# London Dispersion versus Intramolecular Hydrogen Bond in Bis‐Pyridines: How Accurate Is DFT for Competing Noncovalent Interactions in the Condensed Phase?

**DOI:** 10.1002/chem.202502745

**Published:** 2025-10-23

**Authors:** Adélaïde Savoy, Vladimir Gorbachev, Charlotte N. Stindt, Peter Chen

**Affiliations:** ^1^ Laboratorium für Organische Chemie Department of Chemistry and Applied Biosciences ETH Zürich Vladimir‐Prelog‐Weg 2 Zürich 8093 Switzerland

**Keywords:** density functional theory, hydrogen bond, isotope perturbation experiments, London Dispersion, noncovalent interactions

## Abstract

We report a systematic investigation of noncovalent interactions—particularly an intramolecular hydrogen bond and London Dispersion forces—in singly protonated bis‐pyridines, studied across solution and crystalline states. Building on our previous gas‐phase study, we combine variable‐temperature ^1^H NMR spectroscopy, single‐crystal X‐ray diffraction, and density functional theory (DFT) calculations. The measured ^1^H NMR chemical shifts of the acidic proton serve as a solution‐phase structural readout, which we correlate with an independent crystallographic metric. By systematically varying the linker (–CH_2_–, –O–, and –CH_2_CH_2_–) and the pendant substituents (H, methyl, *tert*‐butyl), we examine how increasingly bulky “dispersion energy donors” affect both the intramolecular hydrogen bond and the accessible conformational states. In reference systems, where a single noncovalent interaction governs the geometry, even relatively simple computational models correctly reproduce the experimentally observed structures. However, for molecules featuring two competing noncovalent interactions, the tested, dispersion‐corrected, DFT often fails to predict the relative energies of accessible conformers accurately, highlighting current limitations in predictive accuracy. We briefly discuss broader implications of currently achievable predictive accuracy for homogeneous catalysis.

## Graphical abstract

A test system of 14 singly protonated bis‑pyridine salts, with varying H‑bonded and London dispersion interactions, was designed to systematically study non‑covalent interactions across the gas, solution, and solid phases. A good correlation between the ^1^H chemical shift and the N‐H–N angle from the crystal structures was established. We observed systematic discrepancies in the treatment of these interactions by DFT‑D3/D4 across the phases.

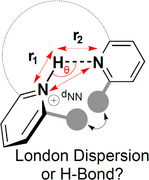

## 1 Introduction

Noncovalent interactions (NCIs) are fundamental in determining the low‐lying conformers of medium‐to‐large organic and organometallic molecules.^[^ ^1^ ^]^ Among the various types of NCIs, this study focuses on two categories: hydrogen bonds and London Dispersion forces.^[^ ^2^, ^3^ ^]^ Hydrogen bonds are well known for their significant role in stabilizing molecular conformations, contributing between 5 and 35 kcal mol^−1^ to a structure's stability.^[^ ^4^ ^]^ The strength of this interaction depends not only on the chemical environment and the nature of the electron donor, but also on the bending of the hydrogen bond, as it is an anisotropic interaction.

In contrast, London Dispersion forces typically involve weaker individual interactions. They have generally been assumed to play a subordinate role due to their small individual magnitudes (e.g., 0.3 kcal mol^−1^ in an Ar_2_ dimer).^[^ ^5^ ^]^ Nevertheless, these forces can become significant due to their additive character, particularly in larger molecules, as the number of atom‐pairwise interactions—one way to treat London Dispersion in large molecules—increases quadratically with the number of atoms. The identified theoretical and practical difficulties in their quantitative prediction by quantum chemical calculations make experimental investigations of these interactions necessary and illuminating. Despite the active reevaluation of London Dispersion in small organic molecules over the last few decades,^[^ ^6^, ^7^ ^]^ challenges remain, particularly in systematically quantifying these interactions across different environments, such as gas phase, solution, and crystal lattice.

In principle, individual NCIs are easier to study in the gas phase, where the absence of solvation or lattice effects simplifies the analysis. In practice, however, most applications rely on the sum of many NCIs operating in solution. We recently reported a gas‐phase study of the ground‐state structures and low‐lying conformations of protonated bis‐pyridines with systematically varied dispersion energy donors (DEDs).^[^ ^8^ ^]^ These studies provided insights into whether dispersion‐corrected density functional theory (DFT) methods can accurately predict the ground‐state structures. In this paper, we extend our investigation to the same protonated bis‐pyridines in solution. Our earlier findings suggested that solvation can attenuate London Dispersion interactions, adding complexity to their interpretation.^[^ ^9^, ^10^ ^]^ The present work, together with our previous gas‐phase studies, offers a basis for testing models that account for solvation effects.

In particular, the present test system features singly protonated bis‐pyridines with varying degrees of flexibility and pendant substituents. Our objective was to explore the bending potential of ionic hydrogen bonds between two equivalent electron donors. In the original experimental design, all studied compounds feature accessible and chemically identical nitrogen atoms, where the hydrogen bond can be rationalized by a symmetric double‐well potential. The height of the energy barrier between the two minima depends on the geometry of the hydrogen bond, which can also be modulated by pendant DEDs. At shorter distances, the barrier between the two minima may become sufficiently low to cause the hydrogen bond to behave like a low‐barrier or single‐well potential.

To differentiate between the two nitrogen atoms, asymmetric ^15^N labeling could, in principle, provide additional insights into the potential energy surface. We employed ^1^H VT‐NMR, isotopic perturbation experiments, single‐crystal XRD, gas‐phase IRMPD spectroscopy,^[^ ^11^ ^]^ and DFT calculations. Given the challenges of modeling charged species in solution, we establish an “experiment‐only” spectroscopic‐structural link by correlating ^1^H VT‐NMR data with the cation geometries obtained from XRD structures, which we then compared with DFT‐calculated geometries. As the ions studied in our current work possess pendant substituents by design, in the discussion section, we intend to accomplish three main objectives:

1. To elucidate the structural information that can be derived from the observed chemical shifts, within a broader context of the investigation of hydrogen bonding;
2. To explore the potential of obtained correlations in the elucidation of the London Dispersion properties;
3. To consider the van der Waals interactions in a broader context and investigate the dispersion and hydrogen bonding contributions in the solution, crystal structures, and compare them to the gas phase.

## 2 Methods

### 2.1 Experimental

^15^N‐pyridine (99 atom % ^15^N) was acquired from Sigma Aldrich. 2‐bromo‐6‐methylpyridine was distilled prior to use. Lutidine was purchased dried and stored in a glovebox. Dry solvents (THF, DCM, Et_2_O, and hexane) were obtained by distillation over drying agents. Other chemicals were purchased from commercial suppliers and used without further purification.

The synthesis of the bipyridines (labeled as **1a**–**14a**) and ^15^N‐tagged bipyridines (**t5a**, **t7a**) is detailed in the Supporting Information. The preparation and characterization of the corresponding tetrakis(3,5‐bis(trifluoromethyl)phenyl)borate (BArF) salts (**1b**–**14b**, **t5b**, and **t7b**), designated by the same numbers as for the neutral bis‐pyridines but with “b” for the protonated version, is also detailed in the Supporting Information.

Variable temperature NMR experiments were performed on a 500 MHz Bruker spectrometer. NMR spectra were recorded every 20 °C from + 10 to −90 °C. The samples for this experiment were prepared in Young tubes in a glovebox under nitrogen atmosphere. CD_2_Cl_2_ was dried by distillation and static drying over highly activated 3 Å molecular sieves and degassed by freeze‐pump‐thaw cycles. Molecular sieves were prepared by overnight drying at 700–800 °C and 10^−^^2^ mbar.

Crystals were prepared by slow cooling in a mixture of DCM/pentane, DCM/hexane, or in absolute ethanol.^[^ ^12^ ^]^ Crystallization in CHCl_3_ was occasionally used but also crystalizes out NaBArF. Dry solvents were sometimes necessary to avoid coordination of water. The crystal data and structure refinement details are documented in the Supporting Information.

### 2.2 Computational Details

Geometry optimization and frequency calculations were performed using Orca (v5.0.4)^[^ ^13^ ^]^ and the BP86^[^ ^14^ ^]^ functional with the Karlsruhe def2‐TZVP^[^ ^15^ ^]^ basis set. Dispersion was treated with the D3^[^ ^16^ ^]^ correction using the Becke–Johnson damping function (D3BJ).^[^ ^17^ ^]^ The obtained structures were used throughout the manuscript for comparison with experimentally determined XRD geometries.

A similar comparative analysis was performed on the most stable conformers from each ensemble generated in our previous work.^[^ ^8^ ^]^ Conformer searches were carried out using the iMTD‐GC workflow,^[^ ^18^ ^]^ as implemented in the CREST (version 2.11)^[^ ^19^, ^20^ ^]^ program using the GFN2‐xTB^[^ ^21^ ^]^ method and default settings. All conformers obtained from CREST were reoptimized with GGA (BP86,^[^ ^14^ ^]^ B97D3,^[^ ^22^ ^]^ PBE^[^ ^23^ ^]^), meta‐GGA (M06‐L,^[^ ^24^ ^]^ TPSS^[^ ^25^ ^]^), or hybrid‐GGA (PBE0,^[^ ^26^ ^]^ B3LYP^[^ ^27^, ^28^, ^29^ ^]^) functionals in combination with the def2‐TZVP^[^ ^15^ ^]^ basis set. London Dispersion was treated using either Grimme's D3^[^ ^16^ ^]^ correction with Becke–Johnson damping^[^ ^17^ ^]^ or the more recent D4 model.^[^ ^30^ ^]^ In the course of the current work, these previously generated data were repurposed to compare the most stable gas‐phase conformers of compounds **7b**, **10b**, and **13b** with their corresponding XRD geometries. However, as these lowest‐energy conformers consistently featured the same effective hydrogen bond across all tested DFT functionals with either D3 or D4 dispersion corrections (vide infra), we limited our discussion to the analysis of a single structure optimized at the BP86‐D3BJ functional with the def2‐TZVP basis set.

^1^H NMR calculations were performed as follows: as described above, initial conformer ensembles were generated using CREST, followed by gas‐phase geometry optimizations with the PBE‐D4 functional and the def2‐TZVP basis set. These structures were then used as starting points for further geometry optimizations and ^1^H NMR chemical shift calculations with the PBE‐D4 functional and the def2‐TZVPD basis set,^[^ ^15^, ^31^, ^32^ ^]^ employing the SMD solvation model^[^ ^33^ ^]^ for dichloromethane (DCM) as implemented in ORCA (version 6.0.1).^[^ ^13^ ^]^ For additional computational details, see Section S6.1 of the Supporting Information. The ^1^H NMR chemical shift for compounds **1b**–**14b** was determined as the difference between the absolute isotropic chemical shielding of the acidic proton in the target bis‐pyridines and the averaged ^1^H isotropic chemical shielding of tetramethylsilane (TMS).

To obtain Boltzmann‐averaged ^1^H NMR values relevant for compounds **7b**, **10b**, and **13b**, different conformer clustering strategies were applied. Clustering was performed either manually, based on relative stabilities and computed ^1^H NMR chemical shifts, or automatically using the hierarchical density‐based spatial clustering of applications with noise (HDBSCAN) algorithm.^[^ ^34^, ^35^ ^]^ The HDBSCAN clustering was implemented via a custom Python script, with various parameters (e.g., minimum cluster size) and input features (combinations of ^1^H NMR shifts, relative stabilities, and RMSD) explored, as detailed in the Supporting Information (Sections S7.1‐7.2). Notably, the different clustering schemes tested did not lead to significantly different results (vide infra). This implementation is based on the *hdbscan* library and utilizes scikit‐learn^[^ ^36^ ^]^ for feature standardization. This enabled efficient and reproducible grouping of conformers according to all spectroscopic, energetic, and structural similarity.

## 3 Results

### 3.1 Experimental Design to Study London Dispersion

**Figure 1:**
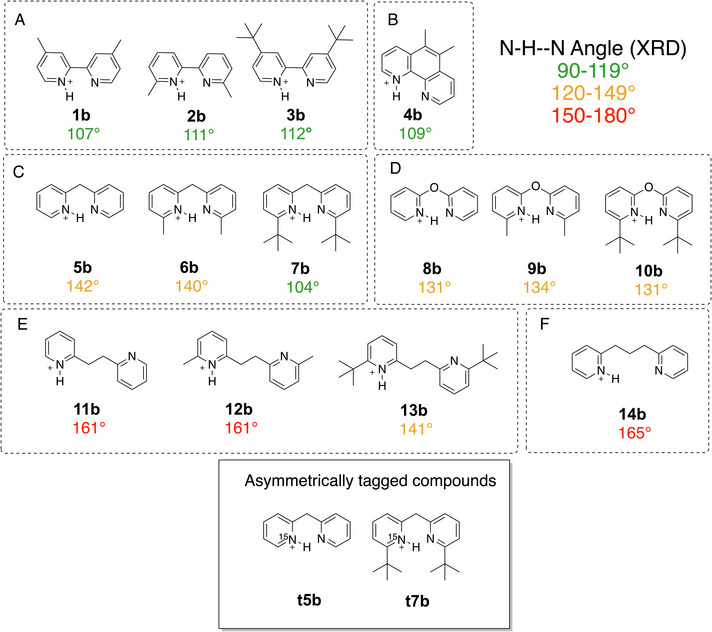
Test system of singly protonated bispyridines, prepared as BArF salts, with the measured N‐H–N angle from XRD structures.

The investigated protonated bis‐pyridine BArF salts along with their N‐H–N angle measured by XRD are depicted in Figure 1. The compounds feature different linkers which ensures different flexibility and distances between the two pyridine moieties, with the plan being that the degree to which the intramolecular hydrogen bond is bent would provide a sensitive readout for the noncovalent interaction between the substituents on the ortho positions of the two pyridine units. The subgroup **A** (Compounds **1b**–**3b**) has no linker but different alkyl substituents, 5,6‐dimethyl phenanthrolinium (**4b**) is a rigid compound of reference. The compounds in subgroups **C**, **D**, and **E** are more flexible due to the different linkers (–CH_2_–, –O–, and –CH_2_CH_2_–), respectively. Each of these 3 subgroups has systematically increased DEDs starting from nonsubstituted, to methyl, to *tert*‐butyl substituted bis‐pyridine. Compound **14b** is the most flexible and has the highest measured N‐H–N angle. Additionally, we synthesized and investigated two asymmetrically ^15^N‐tagged compounds (**t5b** and **7tb**).

### 3.2 Establishing an “Experiment‐only” Spectroscopic‐structural Connection

**Figure 2:**
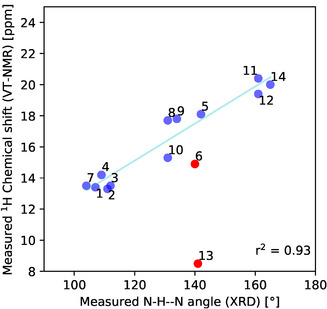
Measured ^1^H‐chemical shift of the acidic proton (VT‐NMR, ‐90 °C) [ppm] of the BArF salts versus measured (XRD) N‐H–N angle [°]. Outliers (in red) are not accounted for in the linear regression and correlation coefficient r^2^.

We seek to establish a correlation of a spectroscopically accessible metric with the structure of the protonated bis‐pyridine, specifically, the distance between the pendant substituents, whose interactions with each other we want to characterize. While a different set of spectroscopic and spectrometric methods apply for the gas phase, we postulate that the ^1^H‐VT‐NMR spectrum in solution may be sensitive to the N‐H–N angle, independently obtained from XRD data analysis. The chemical shift of all protonated bis‐pyridines was measured at ‐90 °C. The numerical values are available in the SI and are presented in Figure 2. Some notable observations from the NMR spectra are that the compound **4b** showed a significantly broadened peak at 8.9 ppm when reducing the temperature. The peak is assigned to the aromatic proton on the carbon neighbouring the nitrogens.

The most plausible explanation for the broadening is that the hydrogen bond has a double‐well potential with a sufficiently high barrier so that exchange of the proton between the two sites slows down at low temperature. It would result in a progressive differentiation of both sites, which can result in a broadening. Compounds **6b** and **13b** show a large, nonlinear variation of the acidic proton chemical shift with respect to temperature. As no precipitation was observed and the elemental analysis of the protonated bis‐pyridine BArF salts confirms a high purity, an equilibrium between at least two conformers with different chemical shifts is the most likely hypothesis. The upfield chemical shift, as well as the XRD Structure of **13b**, suggest that there is an equilibrium in solution between the expected structure, in which there is an intramolecular N‐H–N hydrogen bond, and an alternative conformation with possibly a cation‐π(aryl) interaction. This hypothesis is discussed further below. For the immediate purpose of establishing the correlation of chemical shift to the N‐H–N bond angle, the NMR chemical shifts for these protonated bis‐pyridines represent outliers (in red) that we do not use for the linear regression in Figure 2. They are nevertheless important instances for the overarching aim to determine how interactions between the substituents, i.e., the DEDs, influence the structure of the test molecule.

The analysis of the XRD structures is done, with the caveat, that the positions of H atoms are less precisely localized. The bond lengths involving hydrogen atoms are usually underestimated.^[^ ^37^ ^]^ Therefore, we focus on analysis of potential correlation between the N‐H–N proton in the intramolecular hydrogen bond from ^1^H‐NMR shift from the N‐H–N angle. The linear correlation between ^1^H chemical shift of the proton involved in the hydrogen bonding and the measured N‐H–N angle measured by XRD is shown in Figure 2 and features a reasonably good r^2^ value of 0.93.

### 3.3 Computational Descriptors

**Figure 3:**
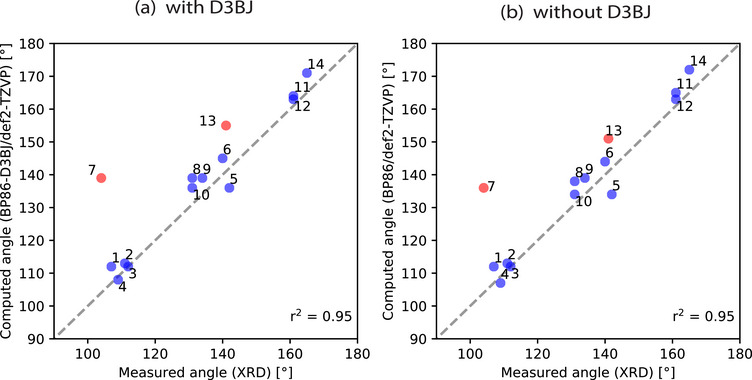
Calculated (BP86/def2‐TZVP) versus experimentally measured (XRD) N‐H–N angle [°] of the BArF salts. Compound **7b** and **13b** (in red) are excluded from the statistical correlation coefficient r^2^.

Having in hand a correlation between condensed phase NMR shift and the XRD data, we seek to establish a computational description. The measured N‐H–N angle was compared to the calculated gas phase geometry obtained using the BP86 functional with the def2‐TZVP basis set, with (Figure 3a) or without the D3BJ dispersion correction (Figure 3b). In all cases, the DFT calculations find a ground‐state structure with an intramolecular N‐H–N hydrogen bond and the two pyridine moieties coplanar or close to coplanar. Both comparisons reveal at least one outlier between the measured (XRD) and calculated N‐H–N angle: compound **7b**. When D3BJ is applied, **13b** shows second‐highest deviation from the linear trend. Remarkably, both compounds have *tert*‐butyl substituents and a relatively flexible linker.

### 3.4 Single‐crystals XRD Analysis

**Figure 4:**
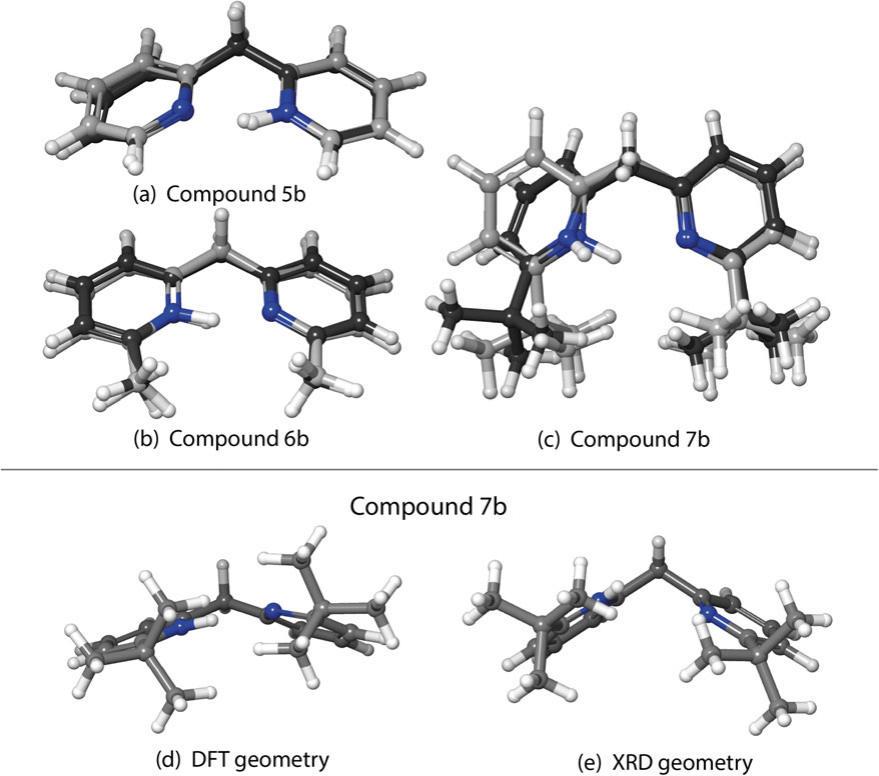
Superposition of the calculated (BP86‐D3BJ/def2‐TZVP) and measured (XRD, without ellipsoids) structures for compounds **5b** a), **6b** b), and **7b** c). Panels d) and e) show the computed (BP86‐D3BJ/def2‐TZVP) and experimental XRD geometry of **7b**, respectively. In superimposed structures (a‐c), the structures measured by XRD have dark grey carbons in contrast with the DFT structures with light grey carbon atoms.

**Figure 5:**
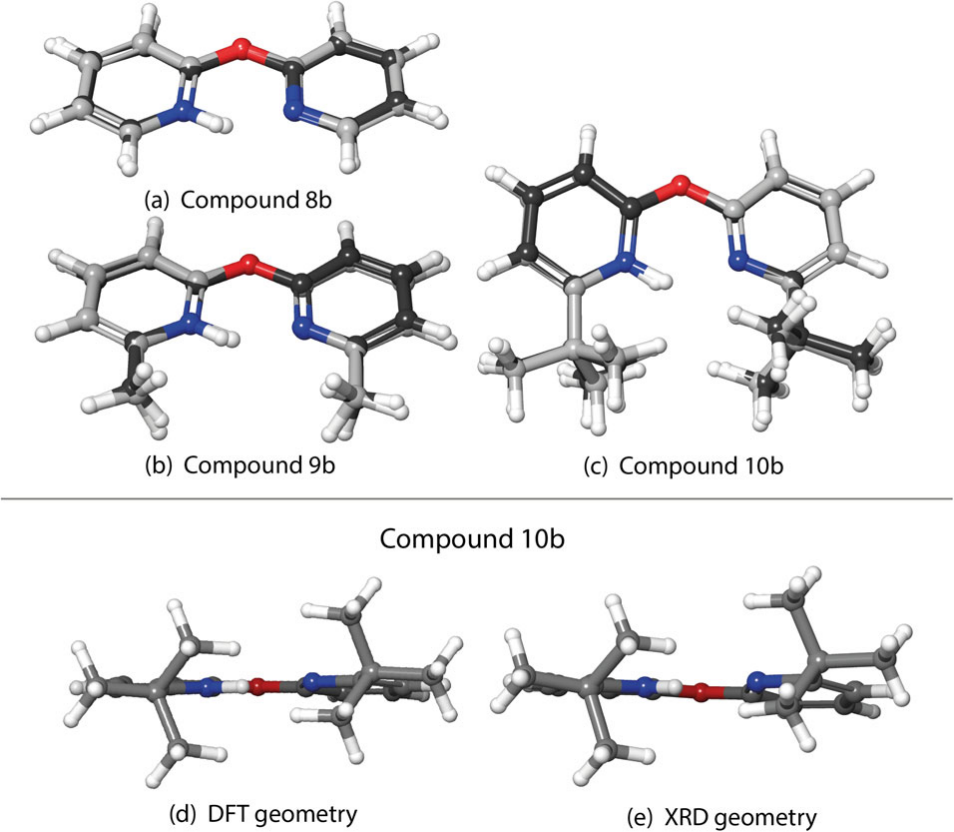
Superposition of the calculated (BP86‐D3BJ/def2‐TZVP) and measured (XRD, without ellipsoids) structures for compounds **8b** a), **9b** b), and **10b** c). Panels d) and e) show the computed (BP86‐D3BJ/def2‐TZVP) and experimental XRD geometry of **10b**, respectively. In superimposed structures (a‐c), the structures measured by XRD have dark grey carbons in contrast with the DFT structures with light grey carbon atoms.

**Figure 6:**
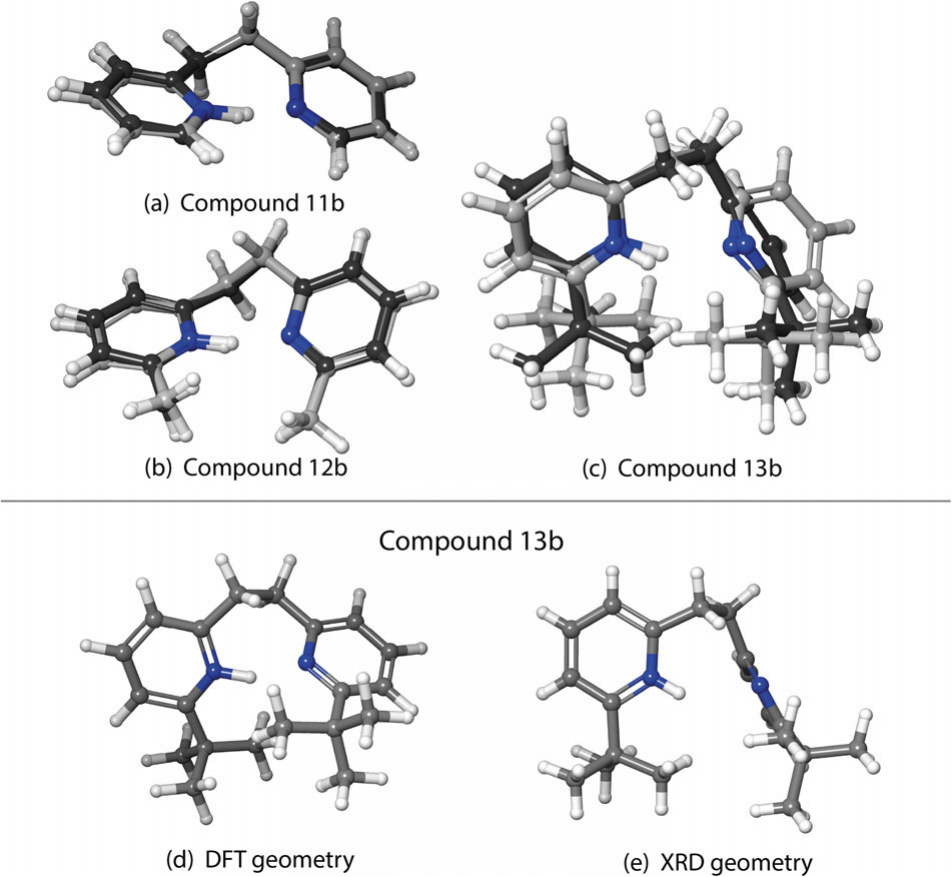
Superposition of the calculated (BP86‐D3BJ/def2‐TZVP) and measured (XRD, without ellipsoids) structures for compounds **11b** a), **12b** b), and **13b** c). Panels d) and e) show the computed (BP86‐D3BJ/def2‐TZVP) and experimental XRD geometry of **13b**, respectively. In superimposed structures (a‐c), the structures measured by XRD have dark grey carbons in contrast with the DFT structures with light grey carbon atoms.

In order to gain insights in the previously mentioned discrepancies, the computed gas phase structures of the protonated bis‐pyridines were compared to the measured XRD structures. The general trend is that the measured XRD geometry resembles the computed one (BP86‐D3BJ/def2‐TZVP) for compounds with hydrogen (Figure 4a) or methyl (Figure 4b) substituents. When investigating the *tert*‐butyl substituted compounds, compound **10b** (Figure 5c‐e) is in reasonable agreement with the gas‐phase computed structure. On the other hand, cations in **7b** (Figure 4c‐e) and **13b** (Figure 6c‐e) show significant differences.

### 3.5 Isotopic Perturbation NMR Study: Probing H‐bond Potential

**Figure 7:**
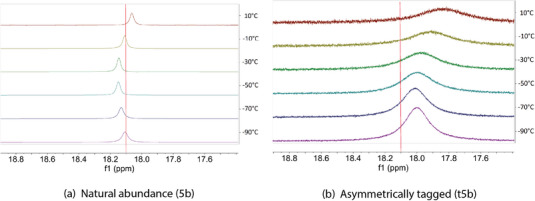
Selected range of the ^1^H VT‐NMR spectra of **5b** and **t5b**.

**Figure 8:**
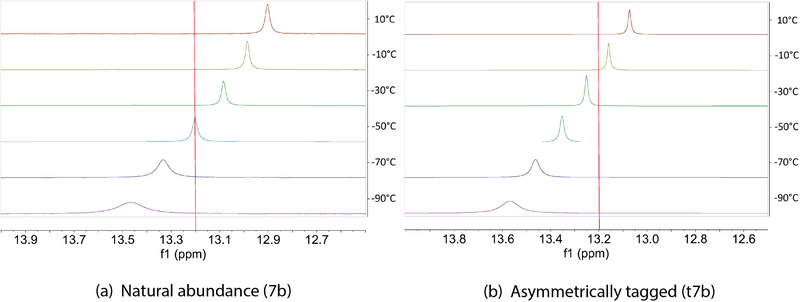
Selected range of the ^1^H VT‐NMR spectra of **7b** and **t7b**.

**^1^H NMR**. The compounds that bear hydrogen or methyl substituents (**5**, **6**, **8**, **9**, **11**, **12** & **14**) have a hydrogen bond with a delocalized proton according to XRD, DFT, and gas‐phase studies.^[^ ^8^ ^]^ Compound **4b** as well as the compounds with *tert*‐butyl substituents (**7b**, **10b** & **13b**), have a proton localized on one of two nitrogen sites in the double‐well potential in the gas phase under the conditions of the experiment. In compounds **4b**, **7b**, and **13b**, the XRD structures suggest that the hydrogen is also localized in solid‐state, which is further supported by solid state FT‐IR (spectra in the SI Section S1.2). However, the assessment of the potential energy surface in solution, by NMR, is challenging. For instance, **4b** is the only compound in the test system for which the barrier in the double‐well potential is undoubtedly high enough to exclude a fast proton exchange on the NMR timescale, due to its rigidity. Compound **5b** has a delocalized proton in the gas phase, and therefore has a single‐well or a double‐well in fast exchange in the condensed phase. On the contrary, compound **7b** has a broken hydrogen bond in the XRD structure and in the gas phase. Both compounds have the same linker and illustrate the two extreme cases of hydrogen bonding, making this comparison relevant. An isotopic perturbation experiment could, in principle, determine whether **7b** has a double or a single‐well potential in the condensed phase. Compound **5b** (Figure 7a) and compound **7b** (Figure 8a) were asymmetrically tagged as compound **t5b** (Figure 7b) and **t7b** (Figure 8b). The chemical shifts of the acidic proton at each temperature are reported in Table 1 (**5b** and **t5b**) and Table 2 (**7b** and **t7b**).

**Table 1:** ^1^H Chemical shifts (δ) in ppm of the acidic proton on the pyridinium of **5b** and **t5b** and their difference (∆(δ))

Temperature [°C]	10	−10	−30	−50	−70	−90
δ 5b	18.06	18.11	18.15	18.15	18.13	18.11
δ t5b	17.79	17.86	17.95	17.98	18.01	18.00
∆(δ)	0.27	0.25	0.20	0.17	0.12	0.11

**^13^C and ^15^N NMR**. The tagging of a nitrogen should result in a change of the chemical shift of the neighboring carbon from singlet to a doublet and a singlet. The analysis of the spectra of both tagged compounds were inconclusive but are discussed in the SI.

^15^N NMR is notably difficult, especially in natural abundance, but even in isotopically enriched species. The broadening tends to make the peaks sink into the baseline. 2D NMR techniques such as ^1^H–^15^N HSQC or ^1^H–^15^N HMBC are often the most practical way to obtain ^15^N shift. The relative ^15^N shift of pyridine (–63.0 ppm) and lutidine (–64.7 ppm) in CDCl_3_ and of pyridinium (–178.6 ppm) and lutidinium (–177.0) in trifluoroacetic acid, referenced to an external standard of DNO_3_, were previously reported.^[^ ^38^ ^]^ The chemical shift of pyridine referenced to ammonia is 318.3 ppm.^[^ ^39^ ^]^ By comparing pyridines and with the corresponding pyridiniums, it is evident that substitution does not affect the ^15^N chemical shift by more than 3 ppm, but that protonation shifts the resonance upfield by about 110 ppm. The tagged compounds synthesized over this study (**t5b** and **t7b**) and their precursors were measured and referenced to ^15^N‐ammonia, as summarized in Table 3.

**Table 2:** ^1^H Chemical shifts (δ) in ppm of the acidic proton on the pyridinium of **7b** and **t7b** and their difference (∆(δ))

Temperature [°C]	10	−10	−30	−50	−70	−90
δ 5b	12.90	12.99	13.08	13.20	13.33	13.46
δ t5b	13.07	13.16	13.25	13.35	13.46	13.57
∆(δ)	0.17	0.17	0.17	0.15	0.13	0.11

**Table 3:** ^1^H ‐^15^N HMBC cross‐peak of ^15^N tagged compounds. **5a** decomposed before the measurement.

Compound	^15^N chemical shift [ppm]
Pyridines	
Pyridine‐N‐oxide^[a]^	292.53
2,6‐dibromopyridine^[a]^	315.62
2,6‐dibromopyridine^[a]^	312.95
Pyridinium BArF salt	
2‐bromo‐6‐tBu‐pyridinium^[b]^	196.28
Bis‐pyridines	
t5a	N/A
t7a^[a]^	312.69
Singly protonated bis‐pyridine BArF salts	
t5b^[b]^	224.38
t7b^[b]^	242.69

[a] in CDCl_3_. [b] in CD_2_Cl_2_.

**^1^H Calculations and Conformer Space Analysis**

**Figure 9:**
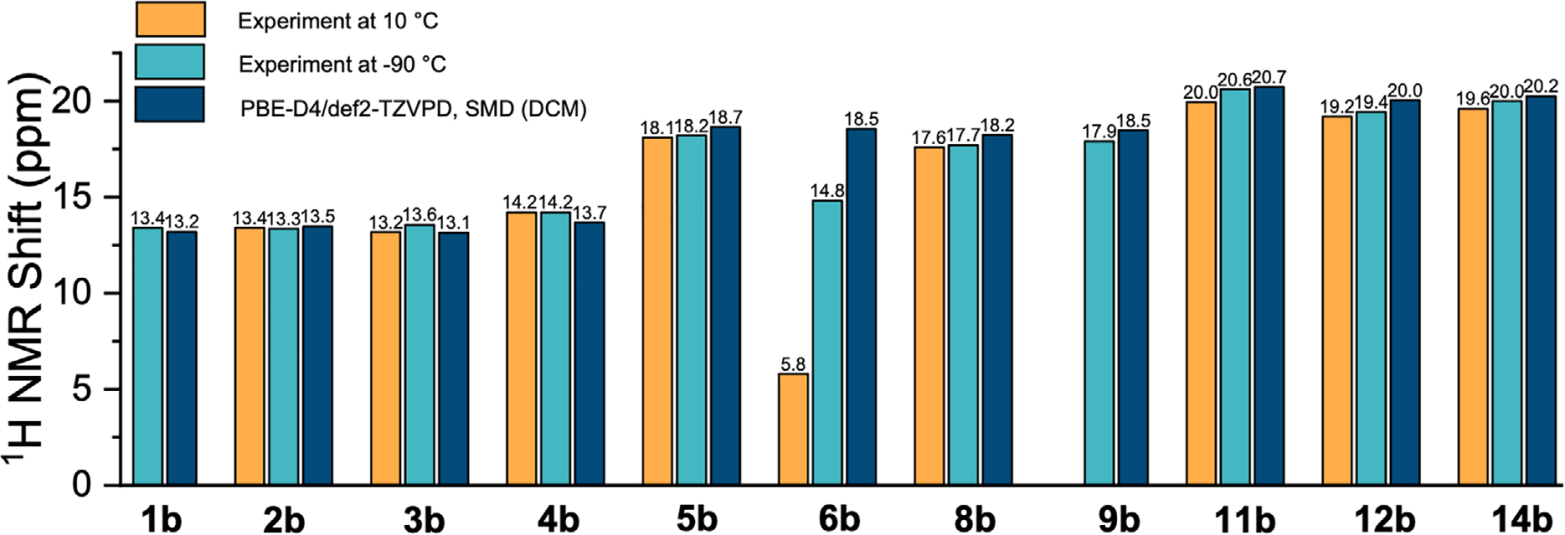
Comparison of experimental and computed ^1^H NMR chemical shifts (in ppm) for protonated bis‐pyridines, excluding *tert*‐butyl‐containing compounds **7b**, **10b**, and **13b**. Experimental data were recorded at 10 °C (orange) and ‐90 °C (cyan), while calculated shifts (dark blue) were obtained using the PBE‐D4 functional with the def2‐TZVPD basis set and the SMD(DCM) solvation model. Excellent agreement is observed across most compounds, with a notable deviation for compound **6b**. The acidic ^1^H signal could not be located for **1b** and **9b** at 10 °C.

In addition to geometry optimizations and comparison with the XRD structures, ^1^H NMR calculations were performed. Initially, to establish a baseline for a later comparison, we consider all compounds except those containing *tert*‐butyl groups. The results are plotted in Figure 9. For most protonated bis‐pyridines, the computed chemical shifts are in excellent agreement with the experimental values, typically much better than 1 ppm—except for compound **6b** (vide infra). For all compounds computed and depicted in Figure 9, the conformational space is relatively simple, usually consisting of 1–5 structures that typically differ by the rotation of substituents (e.g., methyl groups). We note that for these rotameric structures, the PBE‐D4/def2‐TZVPD/SMD(DCM) NMR calculations yield ^1^H chemical shifts that are nearly identical, with deviations of less than 0.1 ppm. These results are provided in Section S6.2 of the Supporting Information.

**Figure 10:**
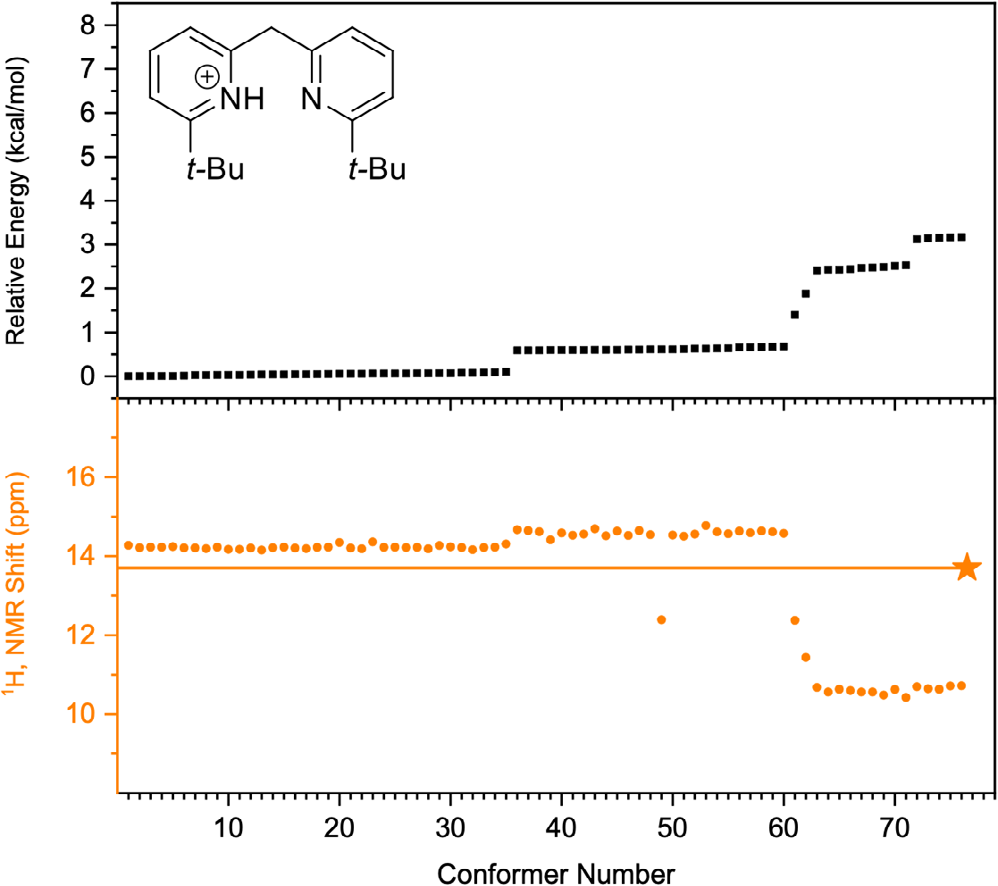
Analysis of the conformer space of bis‐pyridine **7b**, conducted using the PBE‐D4 functional and the def2‐TZVPD basis set with the SMD(DCM) solvation model. The relative energies of the optimized conformers are shown in the top panel, while the computed ^1^H chemical shifts are shown in orange in the bottom panel. The experimental value obtained at −90 °C in DCM is indicated by an orange star.

**Figure 11:**
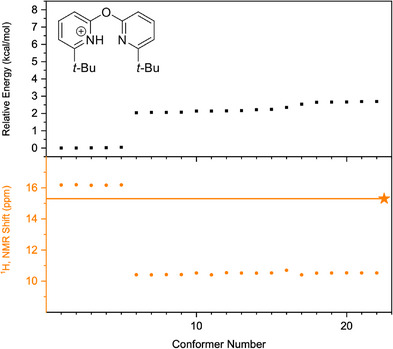
Analysis of the conformer space of bis‐pyridine **10b**, conducted using the PBE‐D4 functional and the def2‐TZVPD basis set with the SMD(DCM) solvation model. The relative energies of the optimized conformers are shown in the top panel, while the computed ^1^H chemical shifts are shown in orange in the bottom panel. The experimental value obtained at −90 °C in DCM is indicated by an orange star.

**Figure 12:**
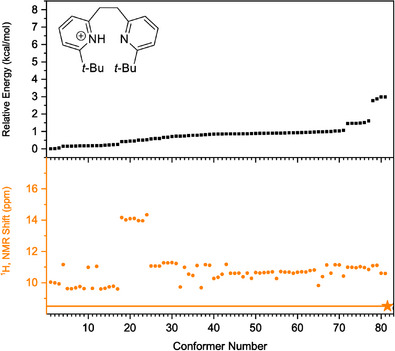
Analysis of the conformer space of bis‐pyridine **13b**, conducted using the PBE‐D4 functional and the def2‐TZVPD basis set with the SMD(DCM) solvation model. The relative energies of the optimized conformers are shown in the top panel, while the computed ^1^H chemical shifts are shown in orange in the bottom panel. The experimental value obtained at −90 °C in DCM is indicated by an orange star.

Next, the conformational spaces of **7b**, **10b**, and **13b** were analyzed and are summarized in Figure 10, 11, 12, respectively. More detailed information is provided in the Supporting Information, Section S6.3. In each Figure, the conformers reoptimized using the PBE‐D4 functional with the def2‐TZVPD basis set and the SMD(DCM) solvation model were found to lie within 3 kcal mol^−^^1^ of the lowest‐energy structure. The numbering of the conformers (horizontal axis in each figure) reflects decreasing relative stability after DFT reoptimization. When multiple conformers show the same energy after reoptimization (indicated on the vertical axis in black in the upper part of each figure)—for example, conformers 1–35 in the top panel of Figure 10—it usually indicates that the starting gas‐phase structures converged to very similar geometries upon full DFT optimization with the solvent model. Using Figure 10 as an example, conformers 1–35 have the same energy but only very slightly different computed ^1^H chemical shift (orange, bottom panel). Conformers 1–35 likely represent the same physical structure, with small differences arising from numerical thresholds in the optimization.

**Figure 13:**
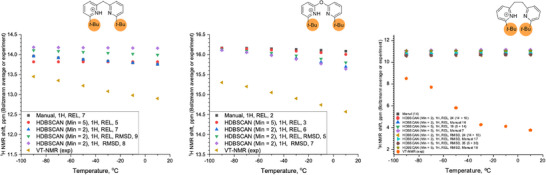
Temperature‐dependent Boltzmann‐averaged ^1^H NMR chemical shifts for conformer ensembles of **7b** (left), **10b** (center), and **13b** (right), compared to experimental values (orange triangles). Different clustering schemes—either manual or HDBSCAN—were applied to prevent overcounting of structurally redundant conformers. For HDBSCAN, various clustering parameters were tested (for details, see Section S7.3 in the Supporting Information). In contrast to the near‐quantitative agreement observed for “simple” bis‐pyridines shown in Figure 9, larger deviations are observed for all bis‐pyridines containing *tert*‐butyl groups. Note that the range of the vertical axis is much larger for **13b** than for either **7b** or **10b**.

To obtain reliable Boltzmann‐averaged ^1^H chemical shifts, all conformers within 3 kcal mol^−^^1^ of the global minimum were considered. In cases where numerous reoptimized structures (e.g., conformers 1–35 in Figure 10) converged to very similar geometries and energies, clustering—either manual or via HDBSCAN—was applied to avoid overcounting equivalent structures. This ensured proper Boltzmann weighting of distinct conformers in each ensemble. Further details on the clustering procedure and conformer selection are provided in the Supporting Information (Section S7), and the Boltzmann‐averaged ^1^H NMR shifts as a function of temperature, compared to the experimental values for **7b**, **10b**, and **13b**, are shown in Figure 13.

## 4 Discussion

London Dispersion, or, more properly, dispersion‐like interactions, as the interaction distances range from long range, where the interaction may rightly be called London Dispersion, to the medium range of a few Å, where some degree of overlap of the electron densities makes the interaction something more complicated than one might get from pure electrostatics, has been re‐examined in the past several years^[^ ^6^, ^7^ ^]^ as a tool to modulate chemical reactivity, and particularly, selectivity.^[^ ^40^, ^41^, ^42^ ^]^ While much of organic reactivity and selectivity has been attributed to, or explained by, steric effects,^[^ ^43^, ^44^, ^45^, ^46^, ^47^ ^]^ specifically a steric repulsion that has been often associated with the Pauli repulsion of two electron densities at ranges below a few Å, recent claims focus on an attractive minimum at the intermediate ranges may be an equally important,^[^ ^6^ ^]^ or useful, design element as the previously employed repulsive wall had been in organic or organometallic structures and reactivity. With the caveat that the attribution of the interaction potential, from the attractive minimum out to its asymptotic limit at long range, cannot be attributed solely to London Dispersion, what are called DEDs^[^ ^6^, ^48^, ^49^ ^]^ have been introduced into a variety of organic and organometallic structures with the aim to facilitate one reaction, or one particular selectivity, over another by the cumulative effect of many weak, NCIs. A variety of experimental approaches have been reported that attempt to place the qualitative ideas on a more quantitative footing, with Schreiner's Gomberg dimers,^[^ ^50^, ^51^, ^52^ ^]^ revisited cyclohexane A‐factors,^[^ ^53^ ^]^ cyclooctatetraenes,^[^ ^54^, ^55^, ^56^, ^57^ ^]^ and bis‐diamondoid hydrocarbons,^[^ ^48^ ^]^ Wegner's DED‐decorated azobenzenes,^[^ ^58^, ^59^, ^60^, ^61^ ^]^ Cockroft's Wilcox‐type balances,^[^ ^62^ ^]^ and Chen's gas‐phase pyridines^[^ ^63^, ^64^ ^]^ representing a selection of the kinds of molecules employed. Each system is designed to probe the magnitude of an (attractive) noncovalent interaction by its additive effect on top of some underlying, structure‐determining, core interaction, which is kept as constant as possible. The different approaches differ in their experimental read‐outs—which physical property that can be measured—but they all have in common that the noncovalent interaction, to be large enough to produce a clear result, demands a “large” molecule with all of the attending synthetic and technical challenges.

Accompanying the revelation that dispersion‐like interactions, when summed together in a sufficiently large molecule, may become large enough to affect structures and reactivity materially, is the development of quantum chemical computational methods for which it has been claimed that they can quantitatively model NCIs accurately.^[^ ^65^ ^]^ To correct for the deficiencies of the Local Density Approximation (LDA) and Generalized Gradient Approximation (GGA) in DFT in capturing dispersion interactions, various methods have been developed, ranging from post‐variational approaches (e.g., D3,^[^ ^16^ ^]^ D4^[^ ^30^ ^]^) to fully nonlocal density functionals (e.g., VV10,^[^ ^64^, ^65^ ^]^ vdW‐DFs^[^ ^68^, ^69^, ^70^, ^71^, ^72^, ^73^ ^]^). Additionally, from the side of wavefunction methods, approximations, such as DLPNO‐CCSD(T)^[^ ^74^, ^75^, ^76^, ^77^, ^78^ ^]^ have been claimed to achieve results close to canonical CCSD(T)^[^ ^79^ ^]^ at a fraction of the computational cost. In all the instances, the benchmarking of the new methods for NCIs in molecules large enough that these NCIs become practically useful for the modulation of structure and reactivity has been hampered by the paucity of reliable experimental data.

The quantification of noncovalent interaction energies can be done, in principle, directly or indirectly. The conceptually simpler, but often technically difficult, experimental direct determination of interaction energies works by measurement of bond dissociation energies in homologous series of gas phase molecules in which the noncovalent interaction varies systematically along the series.^[^ ^80^ ^]^ The indirect method relies on a physical measurement of an equilibrium in which one, target noncovalent interaction is “weighed” against another, perhaps better‐known, noncovalent interaction. A particular embodiment of this experimental approach, in solution, is the Wilcox molecular balance, for which a ^1^H NMR readout encodes the structural information that is the underlying physical basis for an assessment of the strength of the target interaction.^[^ ^81^ ^]^ The Wilcox balance is not the only approach of this kind that can be employed in solution.^[^ ^82^ ^]^

We have designed a test system of singly protonated bis‐pyridines, where dispersion‐like interactions from pendant substituents and an intramolecular hydrogen bond compete against repulsive interactions, i.e., Pauli repulsion, that arise from the same saturated alkyl substituents. This test system was studied experimentally in the crystalline, condensed, and gas phase. Comparing the structures across the phases is the most straightforward approach to understand how NCIs are attenuated in the condensed phase, and is the focus of this work, as well as studying the potential energy surface of the intramolecular hydrogen bonds. The gas phase investigations have been performed in parallel to this study and were recently published.^[^ ^8^ ^]^ In the gas phase, IRMPD spectroscopy (N‐H stretching frequency) was used to probe the degree of intramolecular H‐bonding, and ion mobility spectrometry (collision cross‐section, CCS), were used for experimental readouts sensitive to the structure of the test molecules in the absence of ion pairing and solvation. In condensed phase, one has different experimental readouts. Instead of vibrational spectroscopy for probing the intramolecular H‐bond, we employ ^1^H, ^13^C, and ^15^N NMR spectroscopy. Instead of the CCS from ion‐mobility measurements for the equilibrium structure, we use single‐crystal X‐ray diffraction.

Although some of the control structures, and reference molecules, needed in the present condensed‐phase study differ from those we used in the gas‐phase work, we present, in this work, the same nine protonated bis‐pyridines that we used in the gas phase. We also include, as may be seen in Figure 1, a number of additional structures featuring linkers of variable length. We note that the numbering of the structures does not correspond one‐to‐one with the numbering in the gas‐phase study, given that the condensed‐phase study needed designations for the unprotonated bis‐pyridines, additional reference compounds, and various isotopically‐substituted variants.

### 4.1 What Can We Infer From Experiment Alone?

In an unconstrained system, a hydrogen bond tends to favor a linear geometry, as this arrangement maximizes stabilization. In proton‐bound dimers of pyridines, the resonance for the acidic proton in ^1^H VT‐NMR spectra is significantly shifted downfield (toward 18–22 ppm at low temperature), compared to that for a pyridinium (10‐12 ppm) whose acidic proton is not involved in a H‐bond.^[^ ^9^ ^]^ For intramolecular H‐bonds, as is the case for the test systems, the geometry of the intramolecular H‐bond may be constrained by the molecule's geometry. In our test molecules, it is largely determined by the linker, with increased bending of the H‐bond correspondingly weakening the H‐bond and modifying the chemical shift.

The most easily interpretable indicator for structure in the condensed phase is the single‐crystal X‐ray diffraction structure. There are the usual caveats concerning packing effects,^[^ ^83^, ^84^ ^]^ counter‐ions,^[^ ^85^ ^]^ and the other usual concerns related to extrapolating XRD structures to structures in homogeneous solution. We necessarily need to provide appropriate experimental descriptors in solution that support (or not) the XRD structures. For this purpose, we employ principally ^1^H‐NMR, with some additional experiments with ^13^C or ^15^N.

We hypothesize that the ^1^H NMR chemical shift in homogeneous solution, correlated to an appropriate structural descriptor from the XRD, may provide evidence that the solid‐state structure serves as an adequate surrogate for the structure in solution. Accordingly, Figure 2 shows the chemical shift, at ‐90 °C, of the pyridinium cation's acidic proton for a solution of the BArF salts of the protonated bis‐pyridines **1b** to **14b**, graphed against the N‐H–N angle derived from the XRD structure. Two outliers, to be discussed below, are excluded from the correlation. As a control, the reference compound **4b**, with a wholly rigid geometry, has an essentially completely broken H‐bond, anchoring one end of the correlation. The structures **11b**, **12b**, and **14b**, with the longest linkers, and no other competing interactions, exhibit close‐to‐linear intramolecular H‐bonds in the XRD. Their strongly downshifted ^1^H NMR resonances anchor the other end of the correlation. A total of 12 points gives a good linear fit with r^2^ = 0.93 for an empirical equation:

^1^H Shift = 0.119 × N‐H‐N angle + 0.822

Where the chemical shift is in ppm and the angle in degrees in the range between 90 to 180°. As a reality check, a free pyridinium (at ‐90° C) has a shift of 11.8 ppm^[^ ^9^ ^]^ and a proton bound pyridine dimer (172°)^[^ ^86^ ^]^ of 20.8 ppm^[^ ^9^ ^]^ under the same conditions. The chemical shift extrapolated from our benchmark would be 11.5 and 21.3 ppm, respectively. This is no more than a 2.5% deviation from the experimental values for the two extrema. We surmise from the correlation of an XRD‐derived geometric descriptor with a physical descriptor measured in free solution, that the XRD structures for the 12 protonated bis‐pyridines in solution do indeed represent the ground state structures in solution faithfully.

Two outliers, **6b** and **13b,** were excluded from the correlation in Figure 2. Both behave anomalously in the ^1^H VT‐NMR in two ways. The chemical shift of **13b**, measured at the (standard) ‐90 °C lies significantly off the correlation in Figure 2. While the chemical shift of **6b** lies close to the predicted value in Figure 2, when measured at the lowest temperatures, the chemical shifts for the acidic proton for both **6b** and **13b** show a strong temperature dependence, as seen in the Supporting Information (Section S3.5). For all the other protonated bis‐pyridines, the variation of the ^1^H chemical shift in the range from ‐90 °C to + 10 °C remains below 1 ppm, with the slight variations on the order of 0.1 ppm corresponding to changes in the polarizability of the solvent with temperature. The chemical shift of two, **7b** and **10b**, vary monotonically by about 0.5 ppm between ‐90 °C and + 10 °C, although their chemical shift does nevertheless remain close to the value predicted by the correlation and the N‐H–N angle from the XRD experiment. For **6b** and **13b**, though, the resonance assigned to the pyridinium's acidic proton shifts upfield by 10 and 5 ppm, respectively, going from ‐90 °C to + 10 °C. We can exclude trivial causes, such as precipitation or an interaction with an impurity. As a working hypothesis, the anomalous temperature dependence may be attributed to an equilibrium between at least two, thermally accessible conformers in fast exchange, with the less stable structure having a significantly upfield chemical shift. Given that the acidic proton will have a shift in the range of 10–12 (of a pyridinium) to 20–22 ppm (for a proton involved in an effective intramolecular H‐bond), depending on the extent of H‐bonding, the chemical shifts at + 10 °C of around 5.8 ppm for **6b**, and 3.7 ppm for **13b**, upfield even of a free, non‐H‐bonded pyridinium, suggest a magnetic environment quite different from that in the other structures. Absent a better alternative, a structure, thermally accessible in solution, with the pyridinium's acidic proton in the shielding cone of the opposing aromatic ring, perhaps a cation‐π(aryl) structure, constitutes the most likely explanation. For **6b**, the ground state conformation is likely close to the XRD structure (Figure 4b), as the ^1^H chemical shift, measured at the lowest temperature, ‐90 °C, is not too far from the expectations based on the N‐H–N angle from the XRD structure, the VT‐NMR experiment merely indicating that there is an alternative structure, possibly cation‐π(aryl), at a slightly higher energy. The metastable structure for **13b** must nevertheless lie within a fraction of a kcal mol^−1^ of the ground state. Accordingly, even the two outliers do not invalidate the correlation in Figure 2, from which we may conclude, again, that the XRD structures do indeed represent accessible structures in solution well enough.

### 4.2 What Does DFT Add to the Interpretation of the Experiments?

Now that we establish that the XRD structures are consistent with the condensed phase observation, we want to focus on the compounds **5b** to **13b**, which can be divided into the three series according to their linkers. For each of the linkers, the substituents vary from hydrogen (‐H) to methyl (‐CH_3_), to *tert*‐butyl (‐CMe_3_), to modulate systematically the magnitude of the NCIs (Figure 1, Blocks C‐E). One can make some general observations when comparing their experimental measurements to the gas‐phase DFT‐optimized geometries, as seen in the Figure 4 for the methano‐bridged series, Figure 5 for oxo‐bridged series, and Figure 6 for the ethano‐bridged series. In all three series, the observed XRD structures with small substituents for which the dispersion‐like interactions are also small, i.e., unsubstituted **5b**, **8b**, and **11b**, and methyl‐substituted **6b**, **9b**, and **12b**, the DFT‐optimized structures agree quite acceptably with the XRD structures, with the best agreement appearing consistently for the oxo‐bridged structures for which the structures come close to superimposable. Moreover, for the five, “well‐behaved” cases, **5b**, **8b**, **9b**, **11b** and **12b**, the computational workflow, starting with a CREST conformer search (gas‐phase, 6 kcal mol^−1^ window), then re‐optimization of the ensemble of CREST structures with full DFT (gas‐phase), followed finally with re‐optimization of the gas‐phase structures with full DFT and an implicit solvent model, find only a single, low‐lying conformer, which not only matches the XRD structure closely, but also gives a computed ^1^H chemical shift for the pyridinium proton within much less than 1 ppm of experiment (0.4 ppm compared to observed ^1^H shift at −90 °C). This observation establishes the baseline accuracy for prediction of structure in the solid state, as well as for the prediction of spectroscopic read‐out, in solution, for the cases where dispersion‐like interactions are not large.

Within each series with methano, oxo, or ethano linkers, the largest discrepancies between the structures from DFT versus XRD appear for the largest substituents, *tert*‐butyl, for which the dispersion‐like interactions are also the largest, i.e., **7b**, **10b**, and **13b**. Again, the oxo‐bridged, *tert*‐butyl substituted **10b** (Figure 5c‐e) gives the best agreement, with the principal difference being the minimum C‐H to C‐H distances between the *tert*‐butyl groups, expressed as a different degree of rotation of the *tert*‐butyl groups. For the methano‐bridged*, tert*‐butyl substituted **7b** (Figure 4c‐e), the XRD structure exhibits a much more extremely pronounced puckering than does the DFT structure, which, along with rotation of the *tert*‐butyl groups, significantly increases the C‐H to C‐H distances between the *tert*‐butyl groups relative to that in the DFT structure. For ethano‐bridged, *tert*‐butyl substituted **13b** (Figure 6c‐e), the situation is similar to **7b**, with the deviation from coplanarity of the two rings in the XRD structure being more pronounced than in the DFT‐optimized structure.

One can take the conformational analysis a step further, from a discussion of global minima, either experimentally‐determined or computed by DFT, to an analysis of the ensemble of low‐lying conformations. In contrast to the “well‐behaved” protonated bis‐pyridines above, the same computational workflow yields Figures 10, 11, 12 in which one sees the resulting ensembles of conformations for the protonated bis‐pyridines which show systematic upfield displacement of the ^1^H chemical shifts with increasing temperature. In place of the simple conformational manifolds, in fact, single structures for **5b**, **8b**, **9b**, **11b**, and **12b**, we see a more complicated manifold of structures for **6b** (Figure S5.6), **7b**, **10b**, and **13b** when, for example, a second noncovalent interaction, e.g. dispersion‐like interactions, or something else, becomes comparable to the intramolecular H‐bond.

Starting with the simplest of the complicated cases, **6b**, both experiment and computation had found only a single, intramolecularly H‐bonded structure in the gas phase. Adding the implicit SMD solvent model produced no new structures. Nevertheless, as argued above, from the interpretation of the experimental data alone, the intramolecularly H‐bonded ground state structure of **6b** must necessarily be in fast exchange with a thermally accessible metastable structure, probably cation‐π(aryl), which can lie no more than some tenths of a kcal mol^−1^ above the ground state. The computational workflow finds no metastable conformation within several kcal mol^−1^ of the ground state, and, furthermore, the DFT‐predicted ^1^H chemical shift, based on the computed ground state structure, 18.5 ppm, is about 4 ppm downfield from the observed value of 14.8 ppm at ‐90 °C, which is well outside the baseline accuracy of the prediction by DFT for the simpler cases where there is no question that only one structure is in play. In this case, the dispersion‐like interactions between the methyl groups on **6b** are not large enough to play a material role, but the overall logic, that competing NCIs of comparable magnitude present a difficult challenge for computational chemistry, nevertheless holds, the two competing NCIs, in this case, being presumably the intramolecular H‐bond versus a cation‐π(aryl) interaction.

For the slightly larger test molecules with the *tert*‐butyl substituents, **7b**, **10b**, and **13b**, the situation becomes yet more complicated, as the dispersion‐like interactions become important. Any structure with an intact, intramolecular H‐bond would place the *tert*‐butyl substituents at a distance to each other in which, depending on the rest of the molecule, and the particulars of the computational treatment of the dispersion‐like potential, they could be either pushed up against the repulsive wall, or sit at the bottom of the attractive minimum. For **7b**, **10b**, and **13b**, the computational workflow, starting with CREST in the gas phase, and leading to PBE‐D4/def2‐TZVPD/SMD(DCM)‐optimized structures in solution, produces ensembles of conformations in Figure 10 to Figure 12. We note that many of the 22–81 initial CREST conformers, in the original 6 kcal mol^−1^ windows for each compound, generate, upon re‐optimization with PBE‐D4/def2‐TZVPD/SMD(DCM), a much smaller number of distinguishable conformers. In the SI, we discuss methods for clustering the conformers, starting with manual inspection, and proceeding to RMSD differences, to unsupervised density‐based clustering (HDBSCAN) algorithms, for the purpose of enumerating the physically distinct structures needed for proper Boltzmann‐weighting the computed ^1^H chemical shifts. While the different methods for clustering the hits give slightly different assignments of the hits to distinct conformers, we find fortunately that the range spanned by the Boltzmann‐averaged ^1^H chemical shifts, as one changes the clustering method, stays below 0.5 ppm, which suggests that the different clustering schemes produce largely similar results.

In a first general observation, comparison of the gas‐phase conformer ensembles with those obtained using an implicit solvent model (SI, Section S6.3), indicates that the addition of an implicit solvent model does not appear to change the global minimum for most of the protonated bis‐pyridines. Secondly, the solvent model appears, in general, to compress the range of energies spanned by the ensemble of conformations of bis‐pyridines bearing *tert*‐butyl groups. Thirdly, the different conformational families appear to be stabilized to different degrees by solvation, changing the relative ordering of low‐lying conformations in the cases of **7b**, **10b**, and **13b**.

For the test case with the most flexible covalent linker, the oxo‐bridged **10b**, simultaneous optimization of the intramolecular H‐bond, and the dispersion‐like interaction between the *tert*‐butyl groups, appears to be within reach, or, at least to a reasonable extent, in the solid state, and, by extension, in solution. Interestingly, the most likely interpretation of the IRMPD and CCS results for **10b** in gas phase, especially the deuteration experiments, points to two, almost energetically degenerate, conformations, one with an intramolecular H‐bond distorted enough in length and angle as to weaken it significantly, and a second one wholly lacking an intramolecular H‐bond. Houk and coworkers had indicated that strong, ionic H‐bond strengths in the gas phase are significantly reduced in solution,^[^ ^87^ ^]^ and we had determined that the attractive, dispersion‐like interactions in the gas phase are also significantly attenuated in solution.^[^ ^9^, ^10^ ^]^ While both the intramolecular H‐bond and the attractive, dispersion‐like interaction are attenuated, going from the gas phase to the condensed phase, there is no necessary reason that they are attenuated to the same extent. The XRD structure of **10b** suggests a greater attenuation of the dispersion‐like interaction, resulting in a condensed‐phase structure for **10b** still dominated by the intramolecular H‐bond. The experiment, in the gas phase, indicates that the two conformers, weakly H‐bonded and anti, are nearly degenerate in energy. The ^1^H VT‐NMR experiment for **10b**, in solution, indicates a weakly H‐bonded ground state, with a conformer with an upfield‐shifted ^1^H resonance thermally accessible at + 10 °C. The computational workflow finds for **10b**, in solution, two low‐lying conformers, which examination reveal to be a weakly H‐bonded conformer and a metastable anti structure, but with an energy difference of more than 2 kcal mol^−1^. A metastable anti conformation would show an upfield‐shifted ^1^H chemical shift, which would shift a Boltzmann‐averaged chemical shift in the right direction. The actual Boltzmann‐averaged ^1^H chemical shift, computed by DFT and shown in Figure 13 (center), however, is too far downfield, by 1.5 ppm, and furthermore shows, for some clustering schemes, no large temperature dependence. Most probably, the identities of the computed conformers, weakly‐H‐bonded and anti, are correct, but the energy difference between them, computed with PBE‐D4/def2‐TZVPD/SMD(DCM) is simply too large.

For **7b**, the XRD structure is consistent enough with the most likely interpretation of the IRMPD and CCS data from the gas phase. For **7b**, in the gas phase and in the solid state, the attractive, dispersion‐like interaction of the *tert‐*butyl groups, out‐competes an intramolecular H‐bond weakened by the structural constraint, the strong pucker introduced by the tetrahedral linker in **7b**. In the XRD structure of **7b**, the closest C‐H to C‐H contact at 2.40 Å. It is essentially the same distance as the closest contact in methane crystals (2.405 Å).^[^ ^88^ ^]^ As had been indicated in the previous gas‐phase study, we found that the investigated, dispersion‐corrected, DFT methods placed the attractive minimum of the dispersion‐like potential for the noncovalent interaction of the *tert*‐butyl groups too close, at an unrealistically short distance, found to be about 2.10 Å in the previous work for the structures in the gas phase,^[^ ^8^ ^]^ which would then favor planar, or closer‐to‐planar, structures with an intramolecular H‐bond. For **7b**, Figure 10 shows a complicated, conformational manifold. The conformers, found by PBE‐D4/def2‐TZVPD/SMD(DCM) within about 1 kcal mol^−1^ of the computed minimum energy structure, have predicted ^1^H chemical shifts between 14 and 15 ppm, indicating a partial H‐bond. Some transitional structures right around 2 kcal mol^−1^ show predicted ^1^H chemical shifts around 12 ppm, but the anti structures, with no H‐bond at all, and ^1^H chemical shifts around 10 ppm, are computed to start around 3 kcal mol^−1^ above the minimum energy structure. The Boltzmann‐averaged ^1^H chemical shifts for **7b**, with the different clustering schemes for classifying conformers, are shown in Figure 13 (left). As in **10b**, the Boltzmann‐averaged ^1^H chemical shifts deviate systematically from the experimentally observed ones by 1–2 ppm, and they show essentially no temperature dependence at all, in contradiction with experiment. Accordingly, for **7b**, as was observed for **10b**, the energy differences between the conformations with a partial H‐bond, and the metastable anti conformations, appears to be overestimated by PBE‐D4/def2‐TZVPD/SMD(DCM) with the calculated structures putting the *tert*‐butyl groups too close to each other, allowing a greater extent of intramolecular H‐bonding than actually occurs in the molecules in solution.

The most complicated conformational manifold appears for **13b**, which, in additional to the *tert*‐butyl groups contributing significant NCIs, has a longer and more flexible linker. Depending on the clustering scheme, PBE‐D4/def2‐TZVPD/SMD(DCM) finds up to 14 distinct conformational sub‐families within 3 kcal mol^−1^ of the lowest energy structure, as shown in Figure 12. The lowest energy structure has in common with the XRD structure a nearly orthogonal placement of the two pyridine rings, albeit tipped at a different angle, rendering an intramolecular H‐bond geometrically impossible. Accordingly, the computed ^1^H chemical shift for the putative ground state conformer is close to 10 ppm. Within 0.5 kcal mol^−1^, the computational workflow finds multiple, additional conformations, also with ^1^H chemical shifts between 10 and 10.5 ppm. Similarly to **7b**, PBE‐D4/def2‐TZVPD/SMD(DCM) finds low‐lying conformers with a partial, intramolecular H‐bond, identifiable by ^1^H chemical shifts around 14 ppm. Figure 13 shows the Boltzmann‐averaged ^1^H chemical shift from ‐90 °C to + 10 °C, and one sees, even considering the different clustering schemes, that the computed conformational manifold predicts the ^1^H chemical shift between 10.5 and 11 ppm over the entire temperature range, which deviates from the experimental observation in which it varies systematically from of 8.5 ppm at ‐90°, to 3.6 ppm at + 10 °C. As mentioned earlier, the strong upfield shift, even beyond the 10 ppm expected for a free pyridinium, suggests a complete absence of low‐lying H‐bonded conformers, and a large contribution from one or more conformers in which the acidic pyridium proton has an extraordinarily large upfield chemical shift. While we suggest a cation‐π(aryl) interaction that puts the proton in the shielding cone of the aryl ring, we do note that none of the structures found by the computational workflow have chemical shifts that high, which can mean that either the computational workflow has a serious deficiency, or that the peak assignment is inexplicably wrong despite extensive controls. We presently believe that an as‐yet‐unidentified structure featuring a strong cation–π(aryl) interaction, entirely absent from the computed manifold of conformations, is still the best explanation.

### 4.3 What Else Could be Tried Experimentally?

Having a number of the protonated bis‐pyridines, we found evidence for multiple, energetically similar, conformers in the gas phase and in solution. Especially the VT‐NMR studies found that the ^1^H resonance for the acidic proton in **6b** and **13b** shifted significantly upfield, going from ‐90 °C to + 10 °C, which we interpreted as arising from fast exchange between a ground state conformer with one or multiple other conformers. At least one of these conformers must have a downfield chemical shift and a metastable structure with a significantly upfield chemical shift. The ^1^H chemical shift of **6b** at 90 °C, which is not too far from the expectation, based on the linear correlation in Figure 2 (which itself uses angle from the XRD structure), and the large upfield shift, i.e., 10 ppm, going from ‐90 °C to + 10 °C, could mean, as mentioned above, that a putative cation‐π(aryl) structure, in dichloromethane solution, lies perhaps some tenths of a kcal mol^−1^ above the ground state. For **13b**, the anomalous chemical shift, even at ‐90 °C, suggests an even smaller energy difference. We recapitulate the earlier conclusion because the interpretation of the VT‐NMR spectra suggests that it may be possible to test the interpretation with an isotopic perturbation experiment, of the type first introduced by Saunders for carbocations,^[^ ^89^ ^]^ and subsequently applied by Perrin for H‐bonds.^[^ ^90^, ^91^ ^]^ In an ideal case of two (or more) interconverting structures in fast exchange, the existence of multiple structures in fast equilibrium may be revealed by an isotopic substitution that alters the equilibrium constant via a change in the zero‐point energies, relative to the nonisotopically‐substituted case. The perturbation of the equilibrium becomes visible through the displacement of the chemical shift of a reporter nucleus which is itself the weighted average of the chemical shifts of that nucleus for each of the individual structures in fast exchange, the weighting being the relative populations of the different structures at equilibrium. The isotopic perturbation is most sensitive if three conditions are satisfied: (i) the equilibrium constant is close to unity, (ii) the chemical shift of the reporter nucleus is very different for the two or more individual structures in fast exchange, and (iii) the isotopic substitution changes the relative zero‐point energies, and hence the equilibrium constant, as much as possible. Going through the conditions in reverse order, we judged that, for the protonated bis‐pyridines, the isotopic substitution which could possibly change the equilibrium constant the most would be replacement of one of the two nitrogens with ^15^N, as the presence or absence of an intramolecular H‐bond changes the N‐H vibrational frequency by approximately 1000 cm^−1^. Of course, changing the heavier atom in the bond affects the reduced mass less, but it is nevertheless the largest effect one may achieve in this system. For the second condition, the ^1^H chemical shift of the acidic pyridinium proton shifts from approximately +21 ppm for a proton in an intramolecular H‐bond, to +12 ppm for a fully localized pyridinium N‐H without an intramolecular H‐bond. Alternative structures with a pyridinium proton in the shielding cone of an aromatic ring may have chemical shifts even further upfield. According to the first condition, the isotopic perturbation of the chemical shift is the largest when the equilibrium constant is close to unity. The various structures in our protonated bis‐pyridines are not degenerate by symmetry, and, even with the small energy differences, the populations, at low temperature, are weighted in favor of the ground state structure.

A practical criterion is the availability of the (singly) ^15^N‐substituted molecules. As indicated in the Experimental, we succeeded in the preparation of two (singly) ^15^N‐substituted protonated bis‐pyridines, designated **t5b** and **t7b**, albeit in limited amounts. The former served as a control, as the XRD, the VT‐NMR studies, and the gas‐phase study, all suggested no significant participation of a second conformer in the temperature range under study. The *tert*‐butyl‐substituted **t7b** might be expected to have a more complicated manifold of thermally‐accessible conformations. In hindsight, **10b** might have been preferable as a test case because of evidence for multiple, accessible conformations in the gas phase, or **13b**, because of the VT‐NMR evidence for at least two conformations populated at even the lowest temperatures, but for neither the oxo, nor the ethano, bridges was there a sufficiently efficient cross‐coupling scheme starting from reasonably available ^15^N‐labeled precursors. **6b** would have probably been interesting, but the materials did not suffice to produce enough of it after preparing **t5b** (necessary as a control) and **t7b**.

For the case of **5b**, there is no question for the gas phase, the XRD, and the calculations. All agree that there is an intramolecular H‐bond and, if there is a barrier in an asymmetric, intramolecular H‐bond, it is low. The ^1^H NMR of **5b** shows only a very small temperature dependence, likely due to changes in the solvent polarizability with temperature, and it is not monotonic. Adding the single ^15^N‐substitution for **t5b** shifts the ^1^H resonance by about 0.1 ppm, which is also very small. In contrast, a more planar structure, for **7b**, with an intramolecular H‐bond, is disfavored, as it would place the *tert*‐butyl groups too close. The XRD shows a very strongly puckered molecule, which necessarily compromises the intramolecular H‐bond, weakening it significantly. Nevertheless, conformers with even a weak H‐bond show an observable downfield shift relative to conformers in which the H‐bond is wholly broken. If a structure with a weak H‐bond were to be the only thermally accessible structure in solution, then we would expect to see a ^1^H chemical shift around 14 ppm, and there would be no strong temperature dependence for the chemical shift. The ^1^H chemical shift of **7b** does, however, display a temperature dependence, and it shifts monotonically from + 13.5 ppm to + 13.0 ppm, going from ‐90 °C to + 10 °C. As we had argued, the small, but nevertheless definite, upfield shift suggests that **7b** visits more than one distinguishable conformation in solution, and some of these metastable conformations have an upfield ^1^H chemical shift. Looking at the ^1^H NMR spectra of the (singly) ^15^N‐substituted **t7b**, one sees that the ^1^H chemical shift of the reporter nucleus is indeed displaced, but by about 0.3 ppm, relative to the nonisotopically substituted **7b**. The isotopic perturbation of the chemical shift for the control case, **5b** and **t5b**, was only 0.1 ppm. With the small temperature dependence in the ^1^H VT‐NMR, the small—hardly larger than the control—isotopic perturbation in **t7b** versus **7b** could be consistent with a very minor thermal population of an anti, or even cation‐π(aryl), structure, but the condition (i) above is far from well‐satisfied, making the conclusion less than unambiguous. One should note that hypothetically larger populations of other potential conformations would be invisible to the isotopic perturbation experiment, if the chemical shift of the reporter nucleus would be similar for those conformations because, in such a circumstance, condition (ii) would not be satisfied.

### 4.4 What Did the DFT Calculations Contribute to Our Predictive Ability?

The reference cases, **5b**, **8b**, **9b**, **11b**, and **12b**, for which PBE‐D4/def2‐TZVPD/SMD(DCM) agreed well with the experiment in the gas phase and in condensed phase, neither support nor contradict a claim for chemical accuracy with respect to computed strengths of NCIs. For those structures in which dispersion‐like interactions between the substituents on the rings are small, i.e., the unsubstituted and methyl‐substituted cases (except **6b**), DFT structures, with or without a solvent model, are consistent with experiment, whether it is IRMPD or CCS in the gas phase, or XRD structures and ^1^H NMR chemical shifts in the condensed phase. In these instances, the structures are locked down by a single, dominant, noncovalent interaction, the intramolecular H‐bond. Whether a particular computational method produces a good, absolute interaction energy for the intramolecular H‐bond is largely immaterial for the structure in this indirect test of the prediction, as there is no other, comparably large, noncovalent interaction in competition. The structural constraints of the covalently bridged, protonated bis‐pyridines compel congruence of the DFT with the XRD structures for the simple cases, within a wide range of H‐bond strengths, with the best agreements appearing for **8b** and **9b** because the oxo bridge confers a more flexible angle at the bridge for a modest energetic cost, attributable to the possibility of rehybridization at the oxygen center. The dispersion‐like interactions between the substituents, for the small substituents, is just too small to matter, rendering the accuracy of the quantum chemical calculation of the dispersion‐like interaction between the small substituents moot to the structure. One could venture to claim that any model, ranging from Dreiding models^[^ ^92^ ^]^ to coupled‐cluster theory,^[^ ^93^, ^94^ ^]^ should achieve satisfactory structural predictions for **5b**, **8b**, **9b**, **11b**, and **12b** because the structures are set by the single, strong noncovalent interaction.

For the protonated bis‐pyridines with *tert*‐butyl substituents, which are large enough for dispersion‐like interactions to influence the ground state structure, as well as the conformational manifold, the overall picture of the condensed‐phase structures, as determined by XRD and inferred by NMR, compared to the DFT‐optimized structures, parallels the conclusions drawn from the previously published gas‐phase study on the same series of compounds, which is that the dispersion‐corrected DFT appears to over‐estimate the magnitude of alkyl‐alkyl, attractive interactions, which accordingly leads the computational method to underestimate correspondingly the optimal alkyl‐alkyl nonbonded distances. Putting the *tert*‐butyl groups too close artifactually favors the H‐bonded structures, systematically skewing the manifold of conformations.

Considering the test molecules with the *tert*‐butyl substituents, **7b**, **10b**, and **13b**, as well as the solitary case of **6b**, one may surmise that structures, in which two, or more, NCIs operate in competition with each other, challenge the present generation of accessible quantum chemical methods because the level of absolute accuracy needs to be incomparably higher to achieve even qualitative agreement for the structure of the most stable conformation, or a structure‐dependent experimental readout, such as CCS in the gas phase, or NMR chemical shift in solution. Even a modest increase in the structural complexity of the molecule leads to an incommensurate increase in the complexity of the conformational manifold. An absolute uncertainty of, for example, ±1 kcal mol^−1^ for each of the more‐than‐one NCIs, which is better than is typically claimed as “chemical accuracy” for the individual interactions, spoils the reliability of predictions of the number, order, and spacing of conformations which are chemically relevant. This conclusion applies both for the gas phase and the condensed phase, and it has a considerable consequence for the reliability of the accessible quantum chemical methods for the yet more sterically crowded structures, with many, simultaneous, NCIs, appearing in stereoselective reactions, for example. Of course, it has been amply demonstrated that stereoselectivity can be achieved, and predictions do work in many cases. If one takes the case of asymmetric catalysis by homogeneous organometallic complexes as an example, perhaps the “privileged” status of ligands, such as 2,2′‐bis(diphenylphosphino)‐1,1′‐binaphthyl (BINAP) or bis(oxazoline) ligands (BOX), derives from the conformationally rigid structures achieved when the ligands are coordinated to the appropriate metal center. The sterically congested BINAP complexes, and the well‐defined *d* ^8^‐square‐planar complexes with BOX ligands, do not offer the possibility of chemically‐relevant, alternative conformations, despite the size and complexity of the molecules. In such cases, the “right” structure may be predicted by methods of considerably lower accuracy. It would appear, however, imprudent to extrapolate the success to more flexible structures, either ground state or transition state, based on our present results, without significantly more accurate computational methods.

## 5 Conclusion

Our combined experimental and computational investigation of protonated bis‐pyridines, in which dispersion‐like interactions from pendant substituents and an intramolecular hydrogen bond compete with repulsive interactions arising from the same alkyl substituents, reveals notable limitations in the predictive ability of widely used dispersion‐corrected DFT‐D3 and DFT‐D4 calculations. In reference molecules lacking large alkyl substituents, the intramolecular hydrogen bond remains the dominant interaction, and dispersion‐corrected DFT generally agrees with both XRD and low‐temperature ^1^H NMR measurements. However, upon introducing *tert*‐butyl substituents, the interplay between London dispersion and hydrogen bonding becomes more intricate, and the tested dispersion‐corrected DFT‐D4 methodology struggles to reproduce reliably the experimentally observed ^1^H shifts.

This study complements our earlier gas‐phase work, where computational methods that performed well for the same unsubstituted or methyl‐substituted protonated bis‐pyridines failed to capture the ground states of their tert‐butyl‐substituted analogs. Upon transfer into solution, the manifold of NCIs becomes complicated even further by variable degrees of attenuation, and dispersion‐corrected DFT‐D4 methods do not appear able to reach the required level of chemical accuracy.

Although such accuracy can be achieved in certain organometallic complexes—particularly where a rigid ligand environment significantly limits conformational freedom—extending these methods to more flexible systems, for instance in some classes of asymmetric or peptide‐based catalysts, remains a significant challenge.

## Supporting Information

General materials and methods, synthetic procedures for precursors and neutral/protonated bipyridines, as well as the synthesis of asymmetrically ^15^N‐tagged compounds. Characterization by NMR, HRMS, and solid‐state FT‐IR spectroscopy is also provided, along with additional discussion about ^1^ ^3^C NMR and solid‐state FT‐IR data. The computational protocols, generation of conformer ensembles, and the various clustering schemes applied within the HDBSCAN methodology are detailed as well. (PDF)

XYZ coordinates for all compounds and all conformers of ions **7b**, **10b**, and **13b** (ZIP).

All crystal structures were uploaded to the Cambridge Crystallographic Data Centre database.^[^ ^95^ ^]^ Deposition Numbers https://www.ccdc.cam.ac.uk/services/structures?id = https://doi.org/10.1002/chem.202502745

2 352 861 **1b**

2 352 862 **2b**

2 352 860 **3b**

2 352 865 **4b**

2 352 874 **5b**

2 352 870 **6b**

2 352 857 **7b**

2 352 869 **8b**

2 352 868 **9b**

2 352 867 **10b**

2 352 873 **11b**

2 352 863 **12b**

2 352 871 **13b**

2 352 864 **14b**

2 470 444 Compound **B‐H‐B+**

Contain the supplementary crystallographic data for this paper. These data are provided free of charge by the joint Cambridge Crystallographic Data Centre and Fachinformationszentrum Karlsruhe http://www.ccdc.cam.ac.uk/structures Access Structures service.

The authors have cited additional references within the Supporting Information.^[^ ^96^, ^97^, ^98^, ^99^, ^100^, ^101^, ^102^, ^103^, ^104^, ^105^, ^106^, ^107^, ^108^, ^109^, ^110^, ^111^, ^112^, ^113^, ^114^, ^115^, ^116^ ^]^

## Conflict of Interest

The authors declare that they have no competing interests.

## Supporting information



Supporting Information

Supporting Information

Supporting Information

## Data Availability

All data generated or analysed during this study are included in this published article and its supplementary information files.
