# Supplementary material for: London Dispersion versus Intramolecular Hydrogen Bond in Bis‐Pyridines: How Accurate Is DFT for Competing Noncovalent Interactions in the Condensed Phase?
Source: Chemistry. 2025 Oct 23;31(66):e02745. doi: 10.1002/chem.202502745 (PMC12648470; doi:10.1002/chem.202502745)
Supplement: Supplementary file 1 — Supporting Information [file CHEM-31-e02745-s002.zip › Crystal_structures/10b/c010620_1_1_tables.html]

c010620\_1\_1


# c010620\_1\_1

Table 1 Crystal data and structure refinement for c010620\_1\_1.

| Identification code | c010620\_1\_1 |
| Empirical formula | C50H37BF24N2O |
| Formula weight | 1148.62 |
| Temperature/K | 100.0(1) |
| Crystal system | monoclinic |
| Space group | Cc |
| a/Å | 12.4372(4) |
| b/Å | 23.2521(7) |
| c/Å | 18.3176(6) |
| α/° | 90 |
| β/° | 107.9160(10) |
| γ/° | 90 |
| Volume/Å3 | 5040.4(3) |
| Z | 4 |
| ρcalcg/cm3 | 1.514 |
| μ/mm‑1 | 0.152 |
| F(000) | 2320.0 |
| Crystal size/mm3 | 0.24 × 0.16 × 0.15 |
| Radiation | MoKα (λ = 0.71073) |
| 2Θ range for data collection/° | 3.504 to 56.798 |
| Index ranges | -16 ≤ h ≤ 15, -28 ≤ k ≤ 30, -24 ≤ l ≤ 24 |
| Reflections collected | 21661 |
| Independent reflections | 11543 [Rint = 0.0227, Rsigma = 0.0376] |
| Data/restraints/parameters | 11543/361/779 |
| Goodness-of-fit on F2 | 1.022 |
| Final R indexes [I>=2σ (I)] | R1 = 0.0401, wR2 = 0.0907 |
| Final R indexes [all data] | R1 = 0.0485, wR2 = 0.0951 |
| Largest diff. peak/hole / e Å-3 | 0.40/-0.25 || Flack parameter | 0.11(17) |

Table 2 Fractional Atomic Coordinates (×104) and Equivalent Isotropic Displacement Parameters (Å2×103) for c010620\_1\_1. Ueq is defined as 1/3 of of the trace of the orthogonalised UIJ tensor.

| Atom | *x* | *y* | *z* | U(eq) |
| --- | --- | --- | --- | --- |
| F6 | 9136.8(18) | 2530.2(8) | 6036.9(13) | 38.7(5) |
| F9 | 5289(2) | 2139.7(8) | 7293.8(12) | 42.5(5) |
| F4 | 10010(2) | 2620.0(9) | 5210.7(13) | 45.4(6) |
| F7 | 4875(2) | 2215.3(9) | 6082.1(12) | 48.7(6) |
| F18 | 2800.6(16) | 5617.0(9) | 4915.2(13) | 39.1(5) |
| F20 | 1360.3(18) | 3573.7(9) | 3639.1(15) | 48.3(6) |
| O1B | 3110.8(18) | 6804.0(9) | 2380.4(12) | 24.4(4) |
| F5 | 10701.1(18) | 2978.0(9) | 6331.1(14) | 50.0(6) |
| F15 | 7340.1(19) | 6764.8(8) | 6494.7(15) | 51.7(6) |
| F17 | 3399.7(18) | 6038.3(10) | 4088.6(12) | 45.2(5) |
| F21 | 2174.4(18) | 2763.0(9) | 3850.4(15) | 47.9(6) |
| F16 | 3430.6(19) | 6476.8(10) | 5120.8(16) | 58.9(7) |
| F3 | 10685(8) | 4698(8) | 4577(8) | 48.5(12) |
| F13 | 8475(2) | 6228.1(11) | 6127.8(19) | 65.2(8) |
| F8 | 6570(2) | 2060.3(9) | 6749.0(18) | 58.7(7) |
| F19 | 2227(2) | 3323.2(14) | 4783.6(13) | 63.6(8) |
| F14 | 8082(2) | 6032.0(10) | 7156.0(15) | 60.8(7) |
| N1B | 2076(2) | 5944.9(10) | 2249.5(13) | 17.9(5) |
| F12 | 6545(7) | 4717(3) | 8246(4) | 62.4(19) |
| F2 | 9081(3) | 4841.2(19) | 3745(2) | 44.4(10) |
| N2B | 1781(2) | 6876.4(10) | 3043.7(13) | 19.0(5) |
| F1 | 9559(4) | 5323.4(14) | 4790(3) | 52.4(11) |
| C2 | 8007(2) | 4454.3(12) | 4937.7(16) | 20.1(6) |
| C25 | 5248(2) | 3967.9(10) | 4629.1(15) | 15.9(5) |
| C15 | 5645(3) | 2352.0(12) | 6734.1(17) | 25.2(6) |
| C6 | 8085(2) | 3566.7(11) | 5585.4(15) | 15.9(5) |
| C8 | 9730(3) | 2895.6(13) | 5764.1(17) | 23.4(6) |
| C30 | 5359(2) | 4015.8(11) | 3891.6(16) | 20.0(6) |
| C10B | 2663(2) | 7084.3(12) | 2853.8(15) | 20.5(6) |
| C9 | 6121(2) | 3880.4(11) | 6170.4(15) | 14.5(5) |
| C31 | 2292(3) | 3313.7(14) | 4076.4(18) | 27.5(6) |
| C5B | 1847(2) | 5414.7(12) | 1916.5(16) | 19.2(5) |
| C1 | 7477(2) | 4075.5(11) | 5312.5(15) | 16.3(5) |
| C19 | 6673(2) | 5850.6(12) | 5984.7(17) | 21.8(6) |
| C26 | 4215(2) | 3742.2(11) | 4659.7(15) | 17.3(5) |
| C13 | 6165(3) | 3858.9(13) | 7513.2(16) | 23.4(6) |
| C9B | 2786(2) | 6270.2(12) | 2034.5(16) | 19.8(5) |
| C4 | 9636(3) | 3841.5(13) | 5124.1(17) | 23.7(6) |
| C22 | 4967(2) | 5156.3(11) | 5124.5(15) | 16.9(5) |
| C21 | 4768(2) | 5735.8(11) | 5221.9(16) | 18.4(5) |
| C20 | 5619(3) | 6092.3(11) | 5650.5(16) | 20.5(6) |
| C5 | 9128(2) | 3450.5(11) | 5484.3(16) | 18.5(5) |
| C18 | 6869(2) | 5270.4(12) | 5898.7(16) | 19.6(5) |
| C28 | 3499(3) | 3621.1(12) | 3281.5(16) | 23.8(6) |
| C3 | 9064(3) | 4345.1(12) | 4854.2(17) | 23.1(6) |
| C27 | 3362(2) | 3574.0(11) | 4006.8(17) | 21.6(6) |
| C17 | 6022(2) | 4900.7(10) | 5464.4(14) | 14.8(5) |
| C6B | 2333(3) | 5245.1(13) | 1367.1(18) | 26.9(6) |
| C10 | 5933(2) | 3282.9(11) | 6165.6(15) | 16.0(5) |
| C14 | 6232(2) | 4157.3(12) | 6869.2(16) | 19.4(5) |
| C24 | 3619(3) | 5970.3(12) | 4846.4(18) | 27.3(7) |
| C11 | 5865(2) | 2987.3(11) | 6806.3(16) | 17.7(5) |
| C12 | 5986(2) | 3271.0(12) | 7489.4(16) | 22.0(6) |
| C29 | 4505(3) | 3844.3(12) | 3239.8(16) | 22.5(6) |
| C14B | 1288(3) | 7165.5(12) | 3508.2(16) | 22.0(6) |
| C16 | 6306(6) | 4159(3) | 8258(4) | 35.4(11) |
| C23 | 7635(3) | 6220.3(14) | 6443(2) | 35.3(8) |
| C15B | 238(3) | 6900.3(13) | 3620.3(18) | 25.0(6) |
| C11B | 3140(3) | 7608.7(13) | 3137.0(18) | 26.3(6) |
| C8B | 3309(3) | 6138.9(13) | 1495.1(18) | 26.7(6) |
| C7 | 9606(7) | 4797(3) | 4497(3) | 30.2(11) |
| C3B | 1660(3) | 4940.8(13) | 3076.8(17) | 26.9(6) |
| C13B | 1755(3) | 7681.4(13) | 3800.0(18) | 29.5(7) |
| F10 | 7091(6) | 3936(2) | 8841(2) | 84.3(19) |
| C1B | 1089(3) | 5031.9(12) | 2214.8(17) | 23.5(6) |
| C32 | 4700(3) | 3901.2(14) | 2471.1(17) | 30.3(7) |
| F22 | 4894(7) | 4423.4(18) | 2308(3) | 95(2) |
| F23 | 3796(4) | 3749(3) | 1889.8(18) | 79.2(17) |
| C7B | 3058(3) | 5605.1(15) | 1151.0(19) | 32.4(7) |
| C4B | -66(3) | 5319.1(14) | 2064.1(19) | 28.5(7) |
| F11 | 5369(5) | 4142(4) | 8442(4) | 107(3) |
| F24 | 5489(5) | 3570(3) | 2399(2) | 92(2) |
| C12B | 2673(3) | 7901.6(13) | 3613.3(18) | 31.4(7) |
| C2B | 921(3) | 4440.5(14) | 1825(2) | 34.2(8) |
| B1 | 6222(3) | 4205.5(12) | 5398.4(17) | 14.5(5) |
| C16B | 438(3) | 6272.9(13) | 3868(2) | 30.1(7) |
| C18B | -98(4) | 7226.4(17) | 4243(2) | 42.6(9) |
| C17B | -715(3) | 6945.9(16) | 2859(2) | 37.2(8) |
| C16A | 6116(17) | 4208(8) | 8199(11) | 35.4(11) |
| F12A | 5166(12) | 4442(6) | 8158(8) | 63(4) |
| F11A | 6370(15) | 3891(5) | 8821(6) | 55(4) |
| F10A | 6901(16) | 4613(8) | 8341(14) | 58(5) |
| F22A | 4135(11) | 4293(6) | 2059(6) | 55(4) |
| F23A | 4512(15) | 3435(4) | 2080(6) | 60(4) |
| F24A | 5771(8) | 4031(7) | 2524(5) | 50(3) |
| C7A | 9490(20) | 4740(8) | 4360(12) | 30.2(11) |
| F2A | 9194(13) | 4607(6) | 3630(7) | 44.4(10) |
| F1A | 9146(13) | 5272(5) | 4439(8) | 52.4(11) |
| F3A | 10610(30) | 4770(20) | 4620(30) | 48.5(12) |

Table 3 Anisotropic Displacement Parameters (Å2×103) for c010620\_1\_1. The Anisotropic displacement factor exponent takes the form: -2π2[h2a\*2U11+2hka\*b\*U12+…].

| Atom | U11 | U22 | U33 | U23 | U13 | U12 |
| --- | --- | --- | --- | --- | --- | --- |
| F6 | 38.8(11) | 19.8(9) | 61.1(13) | 10.4(9) | 20.5(10) | 8.5(8) |
| F9 | 69.7(16) | 26.8(10) | 36.2(11) | 8.6(8) | 23.9(11) | -13.4(10) |
| F4 | 63.5(15) | 33.7(11) | 45.0(12) | -0.8(9) | 25.5(11) | 25.8(10) |
| F7 | 75.1(17) | 28.2(11) | 30.5(10) | 4.7(8) | -1.9(10) | -27.3(11) |
| F18 | 20.7(10) | 37.5(11) | 56.2(13) | 10.2(10) | 7.8(9) | 6.4(8) |
| F20 | 24.4(11) | 35.8(12) | 74.7(16) | 4.0(10) | 0.3(10) | -0.1(8) |
| O1B | 25.0(12) | 21.7(10) | 29.8(11) | -7.6(8) | 13.2(9) | -7.2(8) |
| F5 | 35.4(13) | 30.7(11) | 61.0(14) | -2.3(10) | -18.9(10) | 4.6(9) |
| F15 | 39.6(13) | 18.3(10) | 81.6(17) | -19.0(10) | -4.4(11) | -1.5(8) |
| F17 | 35.7(12) | 58.0(14) | 32.2(11) | 18.7(10) | -3.6(9) | 9.0(10) |
| F21 | 36.2(13) | 23.7(10) | 79.8(17) | -4.0(10) | 11.7(11) | -6.8(9) |
| F16 | 36.5(13) | 34.5(12) | 83.1(18) | -29.3(12) | -14.7(12) | 19.1(10) |
| F3 | 31.8(17) | 70(5) | 47(2) | 16.5(18) | 17.6(17) | -13.5(14) |
| F13 | 35.3(14) | 47.6(14) | 116(2) | -30.1(14) | 28.0(14) | -23.7(11) |
| F8 | 54.4(15) | 20.3(10) | 111(2) | 7.2(12) | 39.3(15) | 11.3(9) |
| F19 | 40.7(14) | 111(2) | 39.9(12) | -15.4(13) | 14.2(11) | -40.1(14) |
| F14 | 53.2(16) | 41.6(13) | 59.1(15) | -7.5(11) | -24.6(12) | -9.0(11) |
| N1B | 18.4(12) | 17.0(11) | 18.2(11) | -0.9(8) | 5.4(9) | 1.6(9) |
| F12 | 127(5) | 33.5(19) | 31(2) | -11.9(15) | 30(3) | -4(3) |
| F2 | 45.8(17) | 58(3) | 29.7(16) | 14.1(17) | 11.4(12) | -13(2) |
| N2B | 22.9(13) | 13.4(10) | 19.8(11) | -1.8(8) | 5.2(10) | 1.5(9) |
| F1 | 82(3) | 30.9(13) | 58(3) | -6.5(17) | 42(2) | -25.1(16) |
| C2 | 23.8(15) | 16.7(13) | 21.8(13) | 3.4(10) | 10.1(12) | 1.7(11) |
| C25 | 21.1(14) | 6.9(11) | 20.0(13) | 1.4(9) | 6.8(11) | 3.5(9) |
| C15 | 31.0(17) | 18.9(14) | 25.8(15) | 6.9(11) | 8.8(13) | -1.3(12) |
| C6 | 17.0(13) | 11.4(11) | 19.5(12) | -0.9(9) | 5.8(10) | -1.3(9) |
| C8 | 19.8(15) | 22.8(15) | 24.3(14) | -3.7(11) | 1.9(12) | 1.5(11) |
| C30 | 24.4(16) | 15.1(12) | 20.4(13) | 2.2(10) | 6.6(11) | 5.6(11) |
| C10B | 24.1(15) | 20.1(13) | 16.2(13) | -0.6(10) | 4.5(11) | 0.1(11) |
| C9 | 11.4(13) | 13.4(12) | 18.2(12) | 1.8(9) | 3.8(10) | 0.5(9) |
| C31 | 22.5(16) | 28.7(16) | 27.2(15) | -5.8(12) | 1.7(12) | -1.9(12) |
| C5B | 18.7(14) | 17.1(13) | 20.0(13) | -0.9(10) | 3.2(11) | 0.8(10) |
| C1 | 18.0(14) | 13.9(12) | 17.5(12) | -0.9(9) | 5.9(10) | -1.0(10) |
| C19 | 20.4(15) | 17.1(13) | 26.1(14) | -3.4(11) | 4.8(12) | -3.3(10) |
| C26 | 21.4(14) | 13.5(12) | 16.3(12) | -2.1(9) | 4.8(11) | 2.4(10) |
| C13 | 26.8(16) | 26.8(15) | 16.8(13) | -2.7(11) | 7.3(12) | -4.1(12) |
| C9B | 19.1(14) | 18.6(13) | 21.3(13) | -2.9(10) | 5.5(11) | -1.9(10) |
| C4 | 21.7(15) | 26.4(15) | 26.1(14) | -2.0(11) | 11.9(12) | 1.3(11) |
| C22 | 21.4(14) | 12.0(11) | 16.9(12) | 0.1(9) | 5.3(11) | -0.2(10) |
| C21 | 20.2(14) | 13.6(12) | 19.8(13) | 1.9(10) | 4.0(11) | 2.6(10) |
| C20 | 26.9(16) | 10.7(12) | 23.0(13) | -1.1(10) | 6.2(11) | 2.5(10) |
| C5 | 19.3(14) | 16.2(12) | 19.7(13) | -4.2(10) | 5.6(11) | 0.0(10) |
| C18 | 16.4(14) | 17.1(13) | 24.7(14) | 0.7(10) | 5.1(11) | 0.9(10) |
| C28 | 28.6(17) | 20.6(14) | 17.1(13) | -3.3(10) | -0.8(12) | 3.1(11) |
| C3 | 25.8(16) | 21.9(14) | 25.5(14) | 1.5(11) | 13.6(12) | -1.5(11) |
| C27 | 22.0(15) | 14.2(12) | 25.8(14) | -2.7(11) | 3.0(12) | 1.3(10) |
| C17 | 20.0(14) | 9.3(11) | 16.6(12) | 0.7(9) | 7.9(10) | 1.5(9) |
| C6B | 29.6(17) | 23.8(15) | 29.1(15) | -8.4(12) | 11.8(13) | -2.8(12) |
| C10 | 14.8(13) | 15.0(12) | 17.9(12) | 0.3(9) | 4.6(10) | 1.2(9) |
| C14 | 20.5(14) | 16.6(12) | 20.8(13) | -0.8(10) | 6.2(11) | -1.9(10) |
| C24 | 27.9(17) | 15.9(13) | 31.9(16) | -1.8(11) | 0.0(13) | 3.9(11) |
| C11 | 16.3(14) | 13.5(12) | 22.8(13) | 4.1(10) | 5.3(11) | 0.0(10) |
| C12 | 21.9(15) | 25.0(14) | 18.4(13) | 5.7(11) | 5.1(11) | -0.1(11) |
| C29 | 33.2(17) | 17.0(13) | 16.8(13) | 2.1(10) | 7.1(12) | 8.7(11) |
| C14B | 28.4(16) | 18.6(13) | 18.6(13) | 3.5(10) | 6.8(12) | 10.5(11) |
| C16 | 51(3) | 37(2) | 19.0(16) | -2.9(14) | 11.4(18) | -9.4(18) |
| C23 | 27.1(19) | 21.5(15) | 52(2) | -10.3(14) | 4.3(16) | -3.5(13) |
| C15B | 26.3(16) | 22.3(14) | 29.7(15) | 2.8(11) | 13.6(13) | 9.1(12) |
| C11B | 29.9(17) | 21.5(14) | 25.4(15) | -1.2(11) | 5.5(12) | -5.3(12) |
| C8B | 27.5(16) | 26.5(15) | 30.8(16) | -5.0(12) | 16.0(13) | -8.3(12) |
| C7 | 34(3) | 31(2) | 32(3) | 4.3(18) | 19(2) | -2.4(17) |
| C3B | 35.2(18) | 21.3(14) | 25.1(15) | 2.5(11) | 10.6(13) | 1.9(12) |
| C13B | 42(2) | 20.8(14) | 25.5(15) | -3.3(12) | 10.4(14) | 6.9(13) |
| F10 | 127(5) | 73(3) | 26.0(18) | -10.1(17) | -17(3) | 23(3) |
| C1B | 27.5(16) | 18.1(13) | 27.2(15) | -2.5(11) | 11.7(12) | -3.9(11) |
| C32 | 39.2(19) | 31.3(16) | 18.7(13) | 1.8(11) | 6.2(13) | 4.4(13) |
| F22 | 206(7) | 51(2) | 43(2) | -2.6(18) | 63(3) | -40(3) |
| F23 | 58(3) | 158(5) | 18.2(15) | -13(2) | 7.6(15) | -26(3) |
| C7B | 36.9(19) | 35.3(18) | 31.5(17) | -12.8(14) | 20.2(15) | -5.0(14) |
| C4B | 24.8(17) | 28.1(16) | 34.0(17) | -1.3(13) | 10.9(13) | -7.5(12) |
| F11 | 102(4) | 162(6) | 91(4) | -88(4) | 79(4) | -66(4) |
| F24 | 97(4) | 151(5) | 43(2) | 39(3) | 43(3) | 87(4) |
| C12B | 43(2) | 18.2(14) | 28.5(16) | -4.5(12) | 4.1(14) | -2.5(13) |
| C2B | 49(2) | 22.7(15) | 34.7(17) | -6.4(13) | 17.6(16) | -12.2(14) |
| B1 | 18.9(15) | 8.3(12) | 17.8(13) | 0.3(10) | 7.7(12) | 0.5(10) |
| C16B | 33.5(19) | 20.5(15) | 43.5(18) | 7.2(13) | 22.3(15) | 7.2(12) |
| C18B | 52(2) | 37(2) | 50(2) | -5.7(16) | 32(2) | 11.3(17) |
| C17B | 23.5(18) | 42(2) | 45(2) | 13.3(16) | 8.0(15) | 3.7(14) |
| C16A | 51(3) | 37(2) | 19.0(16) | -2.9(14) | 11.4(18) | -9.4(18) |
| F12A | 77(7) | 67(8) | 48(7) | -21(5) | 24(5) | 18(6) |
| F11A | 97(10) | 58(6) | 13(4) | 3(4) | 21(6) | 15(7) |
| F10A | 67(8) | 63(10) | 49(9) | -36(7) | 26(7) | -42(8) |
| F22A | 60(7) | 74(8) | 37(6) | 33(5) | 21(5) | 47(6) |
| F23A | 115(10) | 38(5) | 32(5) | -22(4) | 31(6) | -25(6) |
| F24A | 38(5) | 102(9) | 14(4) | -9(5) | 15(3) | -10(5) |
| C7A | 34(3) | 31(2) | 32(3) | 4.3(18) | 19(2) | -2.4(17) |
| F2A | 45.8(17) | 58(3) | 29.7(16) | 14.1(17) | 11.4(12) | -13(2) |
| F1A | 82(3) | 30.9(13) | 58(3) | -6.5(17) | 42(2) | -25.1(16) |
| F3A | 31.8(17) | 70(5) | 47(2) | 16.5(18) | 17.6(17) | -13.5(14) |

Table 4 Bond Lengths for c010620\_1\_1.

| Atom | Atom | Length/Å |  | Atom | Atom | Length/Å |
| --- | --- | --- | --- | --- | --- | --- |
| F6 | C8 | 1.320(4) |  | C13 | C14 | 1.393(4) |
| F9 | C15 | 1.331(3) |  | C13 | C12 | 1.384(4) |
| F4 | C8 | 1.333(3) |  | C13 | C16 | 1.495(7) |
| F7 | C15 | 1.320(4) |  | C13 | C16A | 1.513(19) |
| F18 | C24 | 1.343(4) |  | C9B | C8B | 1.374(4) |
| F20 | C31 | 1.334(4) |  | C4 | C5 | 1.385(4) |
| O1B | C10B | 1.336(3) |  | C4 | C3 | 1.381(4) |
| O1B | C9B | 1.396(3) |  | C22 | C21 | 1.391(3) |
| F5 | C8 | 1.342(4) |  | C22 | C17 | 1.400(4) |
| F15 | C23 | 1.329(4) |  | C21 | C20 | 1.383(4) |
| F17 | C24 | 1.339(4) |  | C21 | C24 | 1.486(4) |
| F21 | C31 | 1.340(4) |  | C18 | C17 | 1.402(4) |
| F16 | C24 | 1.329(3) |  | C28 | C27 | 1.395(4) |
| F3 | C7 | 1.324(8) |  | C28 | C29 | 1.378(4) |
| F13 | C23 | 1.341(4) |  | C3 | C7 | 1.503(7) |
| F8 | C15 | 1.328(4) |  | C3 | C7A | 1.50(2) |
| F19 | C31 | 1.323(4) |  | C17 | B1 | 1.646(4) |
| F14 | C23 | 1.326(5) |  | C6B | C7B | 1.375(4) |
| N1B | C5B | 1.366(3) |  | C10 | C11 | 1.385(4) |
| N1B | C9B | 1.312(4) |  | C11 | C12 | 1.381(4) |
| F12 | C16 | 1.332(8) |  | C29 | C32 | 1.506(4) |
| F2 | C7 | 1.334(7) |  | C14B | C15B | 1.515(4) |
| N2B | C10B | 1.338(4) |  | C14B | C13B | 1.368(4) |
| N2B | C14B | 1.368(3) |  | C16 | F10 | 1.312(7) |
| F1 | C7 | 1.345(6) |  | C16 | F11 | 1.309(8) |
| C2 | C1 | 1.400(4) |  | C15B | C16B | 1.525(4) |
| C2 | C3 | 1.392(4) |  | C15B | C18B | 1.530(4) |
| C25 | C30 | 1.404(4) |  | C15B | C17B | 1.531(5) |
| C25 | C26 | 1.405(4) |  | C11B | C12B | 1.369(4) |
| C25 | B1 | 1.645(4) |  | C8B | C7B | 1.383(4) |
| C15 | C11 | 1.501(4) |  | C3B | C1B | 1.534(4) |
| C6 | C1 | 1.410(4) |  | C13B | C12B | 1.386(5) |
| C6 | C5 | 1.392(4) |  | C1B | C4B | 1.531(4) |
| C8 | C5 | 1.501(4) |  | C1B | C2B | 1.534(4) |
| C30 | C29 | 1.390(4) |  | C32 | F22 | 1.291(5) |
| C10B | C11B | 1.385(4) |  | C32 | F23 | 1.336(5) |
| C9 | C10 | 1.408(4) |  | C32 | F24 | 1.286(5) |
| C9 | C14 | 1.401(4) |  | C32 | F22A | 1.252(8) |
| C9 | B1 | 1.642(4) |  | C32 | F23A | 1.281(8) |
| C31 | C27 | 1.503(4) |  | C32 | F24A | 1.340(9) |
| C5B | C6B | 1.381(4) |  | C16A | F12A | 1.282(17) |
| C5B | C1B | 1.515(4) |  | C16A | F11A | 1.312(18) |
| C1 | B1 | 1.645(4) |  | C16A | F10A | 1.323(18) |
| C19 | C20 | 1.384(4) |  | C7A | F2A | 1.309(17) |
| C19 | C18 | 1.388(4) |  | C7A | F1A | 1.334(18) |
| C19 | C23 | 1.502(4) |  | C7A | F3A | 1.325(19) |
| C26 | C27 | 1.389(4) |  |  |  |  |

Table 5 Bond Angles for c010620\_1\_1.

| Atom | Atom | Atom | Angle/˚ |  | Atom | Atom | Atom | Angle/˚ |
| --- | --- | --- | --- | --- | --- | --- | --- | --- |
| C10B | O1B | C9B | 127.5(2) |  | F16 | C24 | F18 | 106.3(3) |
| C9B | N1B | C5B | 116.9(2) |  | F16 | C24 | F17 | 106.5(3) |
| C10B | N2B | C14B | 122.9(2) |  | F16 | C24 | C21 | 114.0(3) |
| C3 | C2 | C1 | 122.8(3) |  | C10 | C11 | C15 | 118.1(2) |
| C30 | C25 | C26 | 115.5(2) |  | C12 | C11 | C15 | 121.1(2) |
| C30 | C25 | B1 | 122.2(2) |  | C12 | C11 | C10 | 120.8(2) |
| C26 | C25 | B1 | 122.2(2) |  | C11 | C12 | C13 | 118.2(2) |
| F9 | C15 | C11 | 113.1(3) |  | C30 | C29 | C32 | 118.5(3) |
| F7 | C15 | F9 | 106.7(3) |  | C28 | C29 | C30 | 121.8(3) |
| F7 | C15 | F8 | 106.2(3) |  | C28 | C29 | C32 | 119.6(3) |
| F7 | C15 | C11 | 112.3(2) |  | N2B | C14B | C15B | 117.0(3) |
| F8 | C15 | F9 | 106.6(3) |  | C13B | C14B | N2B | 117.2(3) |
| F8 | C15 | C11 | 111.6(3) |  | C13B | C14B | C15B | 125.7(3) |
| C5 | C6 | C1 | 122.4(2) |  | F12 | C16 | C13 | 113.8(6) |
| F6 | C8 | F4 | 106.6(2) |  | F10 | C16 | F12 | 106.5(6) |
| F6 | C8 | F5 | 105.9(3) |  | F10 | C16 | C13 | 114.0(5) |
| F6 | C8 | C5 | 113.8(2) |  | F11 | C16 | F12 | 104.8(6) |
| F4 | C8 | F5 | 105.9(3) |  | F11 | C16 | C13 | 110.9(5) |
| F4 | C8 | C5 | 111.9(2) |  | F11 | C16 | F10 | 106.2(6) |
| F5 | C8 | C5 | 112.2(2) |  | F15 | C23 | F13 | 106.6(3) |
| C29 | C30 | C25 | 121.9(3) |  | F15 | C23 | C19 | 113.3(3) |
| O1B | C10B | N2B | 122.1(2) |  | F13 | C23 | C19 | 111.0(3) |
| O1B | C10B | C11B | 117.1(3) |  | F14 | C23 | F15 | 106.6(3) |
| N2B | C10B | C11B | 120.8(3) |  | F14 | C23 | F13 | 106.4(3) |
| C10 | C9 | B1 | 120.4(2) |  | F14 | C23 | C19 | 112.5(3) |
| C14 | C9 | C10 | 115.4(2) |  | C14B | C15B | C16B | 111.2(2) |
| C14 | C9 | B1 | 124.3(2) |  | C14B | C15B | C18B | 110.1(3) |
| F20 | C31 | F21 | 104.8(2) |  | C14B | C15B | C17B | 107.9(2) |
| F20 | C31 | C27 | 113.3(3) |  | C16B | C15B | C18B | 108.0(3) |
| F21 | C31 | C27 | 112.0(3) |  | C16B | C15B | C17B | 110.3(3) |
| F19 | C31 | F20 | 106.1(3) |  | C18B | C15B | C17B | 109.4(3) |
| F19 | C31 | F21 | 106.5(3) |  | C12B | C11B | C10B | 117.4(3) |
| F19 | C31 | C27 | 113.5(2) |  | C9B | C8B | C7B | 115.9(3) |
| N1B | C5B | C6B | 120.4(3) |  | F3 | C7 | F2 | 106.6(7) |
| N1B | C5B | C1B | 115.9(2) |  | F3 | C7 | F1 | 106.3(6) |
| C6B | C5B | C1B | 123.7(3) |  | F3 | C7 | C3 | 113.9(12) |
| C2 | C1 | C6 | 114.8(2) |  | F2 | C7 | F1 | 105.9(5) |
| C2 | C1 | B1 | 122.4(2) |  | F2 | C7 | C3 | 111.1(5) |
| C6 | C1 | B1 | 122.8(2) |  | F1 | C7 | C3 | 112.4(5) |
| C20 | C19 | C18 | 121.3(3) |  | C14B | C13B | C12B | 120.7(3) |
| C20 | C19 | C23 | 119.9(3) |  | C5B | C1B | C3B | 108.1(3) |
| C18 | C19 | C23 | 118.8(3) |  | C5B | C1B | C4B | 109.4(2) |
| C27 | C26 | C25 | 122.5(3) |  | C5B | C1B | C2B | 111.7(2) |
| C14 | C13 | C16 | 121.3(4) |  | C4B | C1B | C3B | 110.9(2) |
| C14 | C13 | C16A | 117.7(8) |  | C4B | C1B | C2B | 108.8(3) |
| C12 | C13 | C14 | 121.1(3) |  | C2B | C1B | C3B | 108.0(3) |
| C12 | C13 | C16 | 117.6(4) |  | F22 | C32 | C29 | 113.2(3) |
| C12 | C13 | C16A | 120.6(8) |  | F22 | C32 | F23 | 103.7(4) |
| N1B | C9B | O1B | 120.3(2) |  | F23 | C32 | C29 | 112.6(3) |
| N1B | C9B | C8B | 126.8(3) |  | F24 | C32 | C29 | 113.0(3) |
| C8B | C9B | O1B | 112.9(2) |  | F24 | C32 | F22 | 109.7(5) |
| C3 | C4 | C5 | 117.8(3) |  | F24 | C32 | F23 | 103.9(4) |
| C21 | C22 | C17 | 122.2(3) |  | F22A | C32 | C29 | 114.0(5) |
| C22 | C21 | C24 | 118.7(3) |  | F22A | C32 | F23A | 107.9(8) |
| C20 | C21 | C22 | 121.2(3) |  | F22A | C32 | F24A | 103.9(8) |
| C20 | C21 | C24 | 120.1(2) |  | F23A | C32 | C29 | 113.2(5) |
| C21 | C20 | C19 | 117.6(2) |  | F23A | C32 | F24A | 103.7(8) |
| C6 | C5 | C8 | 120.6(2) |  | F24A | C32 | C29 | 113.2(4) |
| C4 | C5 | C6 | 121.1(3) |  | C6B | C7B | C8B | 119.4(3) |
| C4 | C5 | C8 | 118.3(3) |  | C11B | C12B | C13B | 121.1(3) |
| C19 | C18 | C17 | 122.2(3) |  | C25 | B1 | C17 | 107.7(2) |
| C29 | C28 | C27 | 117.5(3) |  | C9 | B1 | C25 | 110.6(2) |
| C2 | C3 | C7 | 119.0(4) |  | C9 | B1 | C1 | 108.9(2) |
| C2 | C3 | C7A | 118.7(11) |  | C9 | B1 | C17 | 109.6(2) |
| C4 | C3 | C2 | 121.0(3) |  | C1 | B1 | C25 | 109.1(2) |
| C4 | C3 | C7 | 119.8(4) |  | C1 | B1 | C17 | 110.9(2) |
| C4 | C3 | C7A | 119.6(10) |  | F12A | C16A | C13 | 116.8(15) |
| C26 | C27 | C31 | 120.2(3) |  | F12A | C16A | F11A | 105.1(15) |
| C26 | C27 | C28 | 120.9(3) |  | F12A | C16A | F10A | 108.8(16) |
| C28 | C27 | C31 | 118.9(3) |  | F11A | C16A | C13 | 111.0(14) |
| C22 | C17 | C18 | 115.4(2) |  | F11A | C16A | F10A | 105.3(16) |
| C22 | C17 | B1 | 121.8(2) |  | F10A | C16A | C13 | 109.2(17) |
| C18 | C17 | B1 | 122.7(2) |  | F2A | C7A | C3 | 115.4(17) |
| C7B | C6B | C5B | 120.6(3) |  | F2A | C7A | F1A | 109.3(16) |
| C11 | C10 | C9 | 122.5(2) |  | F2A | C7A | F3A | 108.8(19) |
| C13 | C14 | C9 | 122.0(3) |  | F1A | C7A | C3 | 108.3(15) |
| F18 | C24 | C21 | 112.4(2) |  | F3A | C7A | C3 | 110(4) |
| F17 | C24 | F18 | 104.5(3) |  | F3A | C7A | F1A | 104.8(19) |
| F17 | C24 | C21 | 112.5(3) |  |  |  |  |  |

Table 6 Torsion Angles for c010620\_1\_1.

| A | B | C | D | Angle/˚ |  | A | B | C | D | Angle/˚ |
| --- | --- | --- | --- | --- | --- | --- | --- | --- | --- | --- |
| F6 | C8 | C5 | C6 | 5.7(4) |  | C22 | C21 | C20 | C19 | -0.6(4) |
| F6 | C8 | C5 | C4 | -174.3(3) |  | C22 | C21 | C24 | F18 | 43.1(4) |
| F9 | C15 | C11 | C10 | -162.5(3) |  | C22 | C21 | C24 | F17 | -74.6(3) |
| F9 | C15 | C11 | C12 | 16.9(4) |  | C22 | C21 | C24 | F16 | 164.0(3) |
| F4 | C8 | C5 | C6 | 126.6(3) |  | C22 | C17 | B1 | C25 | 23.8(3) |
| F4 | C8 | C5 | C4 | -53.4(4) |  | C22 | C17 | B1 | C9 | -96.6(3) |
| F7 | C15 | C11 | C10 | -41.8(4) |  | C22 | C17 | B1 | C1 | 143.1(2) |
| F7 | C15 | C11 | C12 | 137.7(3) |  | C21 | C22 | C17 | C18 | -1.0(4) |
| F20 | C31 | C27 | C26 | 127.5(3) |  | C21 | C22 | C17 | B1 | 175.3(2) |
| F20 | C31 | C27 | C28 | -55.1(4) |  | C20 | C19 | C18 | C17 | 0.8(4) |
| O1B | C10B | C11B | C12B | 179.1(3) |  | C20 | C19 | C23 | F15 | 1.7(5) |
| O1B | C9B | C8B | C7B | 177.6(3) |  | C20 | C19 | C23 | F13 | -118.2(3) |
| F5 | C8 | C5 | C6 | -114.5(3) |  | C20 | C19 | C23 | F14 | 122.7(3) |
| F5 | C8 | C5 | C4 | 65.5(4) |  | C20 | C21 | C24 | F18 | -137.7(3) |
| F21 | C31 | C27 | C26 | -114.2(3) |  | C20 | C21 | C24 | F17 | 104.6(3) |
| F21 | C31 | C27 | C28 | 63.2(4) |  | C20 | C21 | C24 | F16 | -16.8(4) |
| F8 | C15 | C11 | C10 | 77.3(3) |  | C5 | C6 | C1 | C2 | -0.4(4) |
| F8 | C15 | C11 | C12 | -103.3(3) |  | C5 | C6 | C1 | B1 | 177.5(2) |
| F19 | C31 | C27 | C26 | 6.4(4) |  | C5 | C4 | C3 | C2 | -0.4(4) |
| F19 | C31 | C27 | C28 | -176.1(3) |  | C5 | C4 | C3 | C7 | 176.7(4) |
| N1B | C5B | C6B | C7B | 0.4(5) |  | C5 | C4 | C3 | C7A | -170.9(10) |
| N1B | C5B | C1B | C3B | -59.3(3) |  | C18 | C19 | C20 | C21 | -0.5(4) |
| N1B | C5B | C1B | C4B | 61.6(3) |  | C18 | C19 | C23 | F15 | -179.0(3) |
| N1B | C5B | C1B | C2B | -177.9(3) |  | C18 | C19 | C23 | F13 | 61.1(4) |
| N1B | C9B | C8B | C7B | -0.5(5) |  | C18 | C19 | C23 | F14 | -58.0(4) |
| N2B | C10B | C11B | C12B | -0.1(4) |  | C18 | C17 | B1 | C25 | -160.1(2) |
| N2B | C14B | C15B | C16B | 51.9(4) |  | C18 | C17 | B1 | C9 | 79.5(3) |
| N2B | C14B | C15B | C18B | 171.6(3) |  | C18 | C17 | B1 | C1 | -40.8(3) |
| N2B | C14B | C15B | C17B | -69.1(3) |  | C28 | C29 | C32 | F22 | 120.9(5) |
| N2B | C14B | C13B | C12B | 1.4(4) |  | C28 | C29 | C32 | F23 | 3.7(5) |
| C2 | C1 | B1 | C25 | 86.4(3) |  | C28 | C29 | C32 | F24 | -113.7(5) |
| C2 | C1 | B1 | C9 | -152.8(2) |  | C28 | C29 | C32 | F22A | 72.1(10) |
| C2 | C1 | B1 | C17 | -32.1(3) |  | C28 | C29 | C32 | F23A | -51.7(10) |
| C2 | C3 | C7 | F3 | 167.7(5) |  | C28 | C29 | C32 | F24A | -169.4(8) |
| C2 | C3 | C7 | F2 | -71.8(6) |  | C3 | C2 | C1 | C6 | -1.4(4) |
| C2 | C3 | C7 | F1 | 46.7(7) |  | C3 | C2 | C1 | B1 | -179.2(3) |
| C2 | C3 | C7A | F2A | -87.6(18) |  | C3 | C4 | C5 | C6 | -1.3(4) |
| C2 | C3 | C7A | F1A | 35(2) |  | C3 | C4 | C5 | C8 | 178.6(3) |
| C2 | C3 | C7A | F3A | 149.1(16) |  | C27 | C28 | C29 | C30 | 0.2(4) |
| C25 | C30 | C29 | C28 | 0.4(4) |  | C27 | C28 | C29 | C32 | 179.6(3) |
| C25 | C30 | C29 | C32 | -179.0(2) |  | C17 | C22 | C21 | C20 | 1.4(4) |
| C25 | C26 | C27 | C31 | 177.6(3) |  | C17 | C22 | C21 | C24 | -179.4(3) |
| C25 | C26 | C27 | C28 | 0.2(4) |  | C6B | C5B | C1B | C3B | 118.0(3) |
| C15 | C11 | C12 | C13 | -178.8(3) |  | C6B | C5B | C1B | C4B | -121.1(3) |
| C6 | C1 | B1 | C25 | -91.3(3) |  | C6B | C5B | C1B | C2B | -0.7(4) |
| C6 | C1 | B1 | C9 | 29.5(3) |  | C10 | C9 | C14 | C13 | 0.2(4) |
| C6 | C1 | B1 | C17 | 150.2(2) |  | C10 | C9 | B1 | C25 | 42.9(3) |
| C30 | C25 | C26 | C27 | 0.4(4) |  | C10 | C9 | B1 | C1 | -76.9(3) |
| C30 | C25 | B1 | C9 | -157.9(2) |  | C10 | C9 | B1 | C17 | 161.6(2) |
| C30 | C25 | B1 | C1 | -38.2(3) |  | C10 | C11 | C12 | C13 | 0.6(4) |
| C30 | C25 | B1 | C17 | 82.3(3) |  | C14 | C9 | C10 | C11 | -0.2(4) |
| C30 | C29 | C32 | F22 | -59.7(6) |  | C14 | C9 | B1 | C25 | -138.4(3) |
| C30 | C29 | C32 | F23 | -176.9(4) |  | C14 | C9 | B1 | C1 | 101.8(3) |
| C30 | C29 | C32 | F24 | 65.7(5) |  | C14 | C9 | B1 | C17 | -19.8(4) |
| C30 | C29 | C32 | F22A | -108.4(9) |  | C14 | C13 | C12 | C11 | -0.6(5) |
| C30 | C29 | C32 | F23A | 127.7(10) |  | C14 | C13 | C16 | F12 | -2.9(8) |
| C30 | C29 | C32 | F24A | 10.1(8) |  | C14 | C13 | C16 | F10 | -125.3(6) |
| C10B | O1B | C9B | N1B | -10.6(4) |  | C14 | C13 | C16 | F11 | 115.0(7) |
| C10B | O1B | C9B | C8B | 171.3(3) |  | C14 | C13 | C16A | F12A | 77.2(16) |
| C10B | N2B | C14B | C15B | 175.1(3) |  | C14 | C13 | C16A | F11A | -162.4(12) |
| C10B | N2B | C14B | C13B | -1.9(4) |  | C14 | C13 | C16A | F10A | -46.7(17) |
| C10B | C11B | C12B | C13B | -0.3(5) |  | C24 | C21 | C20 | C19 | -179.8(3) |
| C9 | C10 | C11 | C15 | 179.2(3) |  | C12 | C13 | C14 | C9 | 0.1(5) |
| C9 | C10 | C11 | C12 | -0.3(4) |  | C12 | C13 | C16 | F12 | 176.0(5) |
| C5B | N1B | C9B | O1B | -176.4(2) |  | C12 | C13 | C16 | F10 | 53.6(7) |
| C5B | N1B | C9B | C8B | 1.5(4) |  | C12 | C13 | C16 | F11 | -66.1(7) |
| C5B | C6B | C7B | C8B | 0.7(5) |  | C12 | C13 | C16A | F12A | -93.7(16) |
| C1 | C2 | C3 | C4 | 1.8(5) |  | C12 | C13 | C16A | F11A | 26.7(18) |
| C1 | C2 | C3 | C7 | -175.3(4) |  | C12 | C13 | C16A | F10A | 142.4(13) |
| C1 | C2 | C3 | C7A | 172.4(9) |  | C29 | C28 | C27 | C31 | -177.9(3) |
| C1 | C6 | C5 | C8 | -178.2(3) |  | C29 | C28 | C27 | C26 | -0.5(4) |
| C1 | C6 | C5 | C4 | 1.8(4) |  | C14B | N2B | C10B | O1B | -177.9(3) |
| C19 | C18 | C17 | C22 | -0.1(4) |  | C14B | N2B | C10B | C11B | 1.2(4) |
| C19 | C18 | C17 | B1 | -176.4(3) |  | C14B | C13B | C12B | C11B | -0.4(5) |
| C26 | C25 | C30 | C29 | -0.7(4) |  | C16 | C13 | C14 | C9 | 178.9(4) |
| C26 | C25 | B1 | C9 | 26.9(3) |  | C16 | C13 | C12 | C11 | -179.4(4) |
| C26 | C25 | B1 | C1 | 146.6(2) |  | C23 | C19 | C20 | C21 | 178.8(3) |
| C26 | C25 | B1 | C17 | -92.9(3) |  | C23 | C19 | C18 | C17 | -178.4(3) |
| C9B | O1B | C10B | N2B | -1.8(4) |  | C15B | C14B | C13B | C12B | -175.2(3) |
| C9B | O1B | C10B | C11B | 179.1(3) |  | C13B | C14B | C15B | C16B | -131.4(3) |
| C9B | N1B | C5B | C6B | -1.4(4) |  | C13B | C14B | C15B | C18B | -11.8(4) |
| C9B | N1B | C5B | C1B | 175.9(3) |  | C13B | C14B | C15B | C17B | 107.5(3) |
| C9B | C8B | C7B | C6B | -0.7(5) |  | C1B | C5B | C6B | C7B | -176.7(3) |
| C4 | C3 | C7 | F3 | -9.4(7) |  | B1 | C25 | C30 | C29 | -176.2(2) |
| C4 | C3 | C7 | F2 | 111.0(6) |  | B1 | C25 | C26 | C27 | 175.9(2) |
| C4 | C3 | C7 | F1 | -130.4(5) |  | B1 | C9 | C10 | C11 | 178.6(2) |
| C4 | C3 | C7A | F2A | 83(2) |  | B1 | C9 | C14 | C13 | -178.5(3) |
| C4 | C3 | C7A | F1A | -154.0(12) |  | C16A | C13 | C14 | C9 | -170.8(9) |
| C4 | C3 | C7A | F3A | -40.2(18) |  | C16A | C13 | C12 | C11 | 170.1(9) |

Table 7 Hydrogen Atom Coordinates (Å×104) and Isotropic Displacement Parameters (Å2×103) for c010620\_1\_1.

| Atom | *x* | *y* | *z* | U(eq) |
| --- | --- | --- | --- | --- |
| H2B | 1506.39 | 6538.75 | 2861.74 | 23 |
| H2 | 7629.96 | 4800.6 | 4731.46 | 24 |
| H6 | 7771.72 | 3293.37 | 5847.81 | 19 |
| H30 | 6037.74 | 4169.77 | 3836.37 | 24 |
| H26 | 4095.64 | 3703.21 | 5145.22 | 21 |
| H4 | 10355.53 | 3765.7 | 5064.71 | 28 |
| H22 | 4366.4 | 4926.22 | 4816.88 | 20 |
| H20 | 5485.14 | 6488.8 | 5713.18 | 25 |
| H18 | 7600.64 | 5118.85 | 6142.52 | 24 |
| H28 | 2920.87 | 3503.79 | 2833.2 | 29 |
| H6B | 2165.01 | 4876.48 | 1136.62 | 32 |
| H10 | 5848.83 | 3074.73 | 5705.11 | 19 |
| H14 | 6357.8 | 4560.75 | 6904.87 | 23 |
| H12 | 5947.13 | 3068.1 | 7930.75 | 26 |
| H11B | 3766.37 | 7759.13 | 3005.91 | 32 |
| H8B | 3811.3 | 6398.77 | 1366.17 | 32 |
| H3BA | 1755.39 | 5312.76 | 3340.66 | 40 |
| H3BB | 2400.93 | 4761.59 | 3159.52 | 40 |
| H3BC | 1187.65 | 4689.82 | 3281.03 | 40 |
| H13B | 1447.37 | 7890.9 | 4134.12 | 35 |
| H7B | 3383.77 | 5488.45 | 768.84 | 39 |
| H4BA | -522.62 | 5097.81 | 2315.91 | 43 |
| H4BB | -452.13 | 5331.95 | 1510.35 | 43 |
| H4BC | 35.52 | 5711.41 | 2269.47 | 43 |
| H12B | 2982.5 | 8261.71 | 3819.29 | 38 |
| H2BA | 1658.74 | 4259.26 | 1899.7 | 51 |
| H2BB | 533.95 | 4486.79 | 1275.15 | 51 |
| H2BC | 464.11 | 4197.51 | 2052.02 | 51 |
| H16A | 727.55 | 6063.52 | 3503.53 | 45 |
| H16B | -275.74 | 6099.51 | 3877.97 | 45 |
| H16C | 989.81 | 6252.65 | 4381.06 | 45 |
| H18A | 517.16 | 7202.88 | 4728.33 | 64 |
| H18B | -782.8 | 7054.73 | 4306.53 | 64 |
| H18C | -241.99 | 7630.38 | 4092 | 64 |
| H17A | -818.77 | 7349.48 | 2698.11 | 56 |
| H17B | -1417.78 | 6798.11 | 2924.64 | 56 |
| H17C | -519.78 | 6719.46 | 2466.65 | 56 |

Table 8 Atomic Occupancy for c010620\_1\_1.

| Atom | *Occupancy* |  | Atom | *Occupancy* |  | Atom | *Occupancy* |
| --- | --- | --- | --- | --- | --- | --- | --- |
| F3 | 0.756(6) |  | F12 | 0.756(6) |  | F2 | 0.756(6) |
| F1 | 0.756(6) |  | C16 | 0.756(6) |  | C7 | 0.756(6) |
| F10 | 0.756(6) |  | F22 | 0.756(6) |  | F23 | 0.756(6) |
| F11 | 0.756(6) |  | F24 | 0.756(6) |  | C16A | 0.244(6) |
| F12A | 0.244(6) |  | F11A | 0.244(6) |  | F10A | 0.244(6) |
| F22A | 0.244(6) |  | F23A | 0.244(6) |  | F24A | 0.244(6) |
| C7A | 0.244(6) |  | F2A | 0.244(6) |  | F1A | 0.244(6) |
| F3A | 0.244(6) |  |  |  |  |  |

Experimental

Single crystals of C50H37BF24N2O
[c010620\_1\_1]
were
[].
A suitable crystal was selected and
[]
on a
Bruker APEX-II Duo (Mo)
diffractometer. The crystal was kept at 100.0(1) K during data collection.
Using Olex2 [1], the structure was solved with the
SHELXT
[2] structure solution program using
Intrinsic Phasing
and refined with the
SHELXL
[3] refinement package using
Least Squares
minimisation.

1. Dolomanov, O.V., Bourhis, L.J., Gildea, R.J, Howard, J.A.K. & Puschmann, H.
   (2009), J. Appl. Cryst. 42, 339-341.
2. Sheldrick, G.M. (2015). Acta Cryst. A71, 3-8.
3. Sheldrick, G.M. (2015). Acta Cryst. C71, 3-8.

Crystal structure determination of
[c010620\_1\_1]

**Crystal Data**
for C50H37BF24N2O (*M*=1148.62 g/mol):
monoclinic, space group Cc (no. 9),
*a* = 12.4372(4) Å, *b* = 23.2521(7) Å, *c* = 18.3176(6) Å, *β* = 107.9160(10)°,
*V*= 5040.4(3) Å3,
*Z* = 4,
*T* = 100.0(1) K,
μ(MoKα) = 0.152 mm-1,
*Dcalc* = 1.514 g/cm3,
21661 reflections measured (3.504° ≤ 2Θ ≤ 56.798°),
11543 unique (*R*int = 0.0227, Rsigma = 0.0376) which were used in all calculations.
The final *R*1 was 0.0401
(I > 2σ(I)) and *wR*2 was 0.0951 (all data).

Refinement model description

Number of restraints - 361,
number of constraints - unknown.

Details:

```
1. Fixed Uiso
```

This report has been created with Olex2, compiled on
2020.02.04 svn.rd84adfe8 for OlexSys. Please
let us know
if there are any errors or if you would like to have additional features.
