# Supplementary material for: London Dispersion versus Intramolecular Hydrogen Bond in Bis‐Pyridines: How Accurate Is DFT for Competing Noncovalent Interactions in the Condensed Phase?
Source: Chemistry. 2025 Oct 23;31(66):e02745. doi: 10.1002/chem.202502745 (PMC12648470; doi:10.1002/chem.202502745)

checkCIF/PLATON report

Structure factors have been supplied for datablock(s) c010620_1_1

THIS REPORT IS FOR GUIDANCE ONLY. IF USED AS PART OF A REVIEW PROCEDURE FOR PUBLICATION, IT SHOULD NOT REPLACE THE EXPERTISE OF AN EXPERIENCED CRYSTALLOGRAPHIC REFEREE.

No syntax errors found. CIF dictionary Interpreting this report

Datablock: c010620_1_1

```
Bond precision:  C-C = 0.0043 A          Wavelength=0.71073

Cell:            a=12.4372(4)    b=23.2521(7)        c=18.3176(6)
                 alpha=90        beta=107.916(1)     gamma=90
Temperature:     100 K
```

	Calculated	Reported
Volume	5040.4(3)	5040.4(3)
Space group	C c	C 1 c 1
Hall group	C -2yc	C -2yc
Moiety formula	C32 H12 B F24, C18 H25 N2 O	C32 H12 B F24, C18 H25 N2 O
Sum formula	C50 H37 B F24 N2 O	C50 H37 B F24 N2 O
Mr	1148.63	1148.62
Dx,g cm-3	1.514	1.514
Z	4	4
Mu (mm-1)	0.152	0.152
F000	2320.0	2320.0
F000’	2322.11	
h,k,lmax	16,31,24	16,30,24
Nref	12661[6338]	11543
Tmin,Tmax	0.971,0.977	0.594,0.746
Tmin’	0.964	

```
Correction method= # Reported T Limits: Tmin=0.594 Tmax=0.746
AbsCorr = MULTI-SCAN

Data completeness= 1.82/0.91      Theta(max)= 28.399

R(reflections)= 0.0401( 10160)    wR2(reflections)= 0.0951( 11543)

S = 1.022                  Npar= 779
```

The following ALERTS were generated. Each ALERT has the format
test-name_ALERT_alert-type_alert-level.
Click on the hyperlinks for more details of the test.

Alert level C

```
PLAT213_ALERT_2_C Atom F11           has ADP max/min Ratio .....        3.8 prolat
PLAT213_ALERT_2_C Atom F22           has ADP max/min Ratio .....        3.2 prolat
PLAT213_ALERT_2_C Atom F23           has ADP max/min Ratio .....        3.1 prolat
PLAT213_ALERT_2_C Atom F11A          has ADP max/min Ratio .....        3.3 prolat
PLAT213_ALERT_2_C Atom F23A          has ADP max/min Ratio .....        3.1 prolat
PLAT213_ALERT_2_C Atom F24A          has ADP max/min Ratio .....        3.5 prolat
PLAT340_ALERT_3_C Low Bond Precision on  C-C Bonds ...............    0.00426 Ang.
PLAT911_ALERT_3_C Missing FCF Refl Between Thmin & STh/L=    0.600         47 Report
PLAT915_ALERT_3_C No Flack x Check Done: Low Friedel Pair Coverage         84 %
```

Alert level G

```
PLAT002_ALERT_2_G Number of Distance or Angle Restraints on AtSite         25 Note
PLAT003_ALERT_2_G Number of Uiso or Uij Restrained non-H Atoms ...         15 Report
PLAT007_ALERT_5_G Number of Unrefined Donor-H Atoms ..............          1 Report
PLAT171_ALERT_4_G The CIF-Embedded .res File Contains EADP Records          5 Report
PLAT176_ALERT_4_G The CIF-Embedded .res File Contains SADI Records          8 Report
PLAT186_ALERT_4_G The CIF-Embedded .res File Contains ISOR Records          1 Report
PLAT187_ALERT_4_G The CIF-Embedded .res File Contains RIGU Records          3 Report
PLAT242_ALERT_2_G Low    'MainMol' Ueq as Compared to Neighbors of        C15 Check
PLAT242_ALERT_2_G Low    'MainMol' Ueq as Compared to Neighbors of        C23 Check
PLAT242_ALERT_2_G Low    'MainMol' Ueq as Compared to Neighbors of        C24 Check
PLAT242_ALERT_2_G Low    'MainMol' Ueq as Compared to Neighbors of        C31 Check
PLAT242_ALERT_2_G Low    'MainMol' Ueq as Compared to Neighbors of        C32 Check
PLAT301_ALERT_3_G Main Residue  Disorder ..............(Resd  1  )        19% Note
PLAT367_ALERT_2_G Long?  C(sp?)-C(sp?) Bond  C29      - C32      .       1.51 Ang.
PLAT720_ALERT_4_G Number of Unusual/Non-Standard Labels ..........          9 Note
PLAT860_ALERT_3_G Number of Least-Squares Restraints .............        361 Note
PLAT912_ALERT_4_G Missing # of FCF Reflections Above STh/L=  0.600         65 Note
PLAT941_ALERT_3_G Average HKL Measurement Multiplicity ...........        3.5 Low
PLAT978_ALERT_2_G Number C-C Bonds with Positive Residual Density.          6 Info
```

---

0 **ALERT level A** = Most likely a serious problem - resolve or explain
0 **ALERT level B** = A potentially serious problem, consider carefully
9 **ALERT level C** = Check. Ensure it is not caused by an omission or oversight
19 **ALERT level G** = General information/check it is not something unexpected

0 ALERT type 1 CIF construction/syntax error, inconsistent or missing data
15 ALERT type 2 Indicator that the structure model may be wrong or deficient
6 ALERT type 3 Indicator that the structure quality may be low
6 ALERT type 4 Improvement, methodology, query or suggestion
1 ALERT type 5 Informative message, check

It is advisable to attempt to resolve as many as possible of the alerts in all categories. Often the minor alerts point to easily fixed oversights, errors and omissions in your CIF or refinement strategy, so attention to these fine details can be worthwhile. In order to resolve some of the more serious problems it may be necessary to carry out additional measurements or structure refinements. However, the purpose of your study may justify the reported deviations and the more serious of these should normally be commented upon in the discussion or experimental section of a paper or in the "special_details" fields of the CIF. checkCIF was carefully designed to identify outliers and unusual parameters, but every test has its limitations and alerts that are not important in a particular case may appear. Conversely, the absence of alerts does not guarantee there are no aspects of the results needing attention. It is up to the individual to critically assess their own results and, if necessary, seek expert advice.

Publication of your CIF in IUCr journals

A basic structural check has been run on your CIF. These basic checks will be run on all CIFs submitted for publication in IUCr journals (*Acta Crystallographica*, *Journal of Applied Crystallography*, *Journal of Synchrotron Radiation*); however, if you intend to submit to *Acta Crystallographica Section C* or *E* or *IUCrData*, you should make sure that full publication checks are run on the final version of your CIF prior to submission.

Publication of your CIF in other journals

Please refer to the *Notes for Authors* of the relevant journal for any special instructions relating to CIF submission.

PLATON version of 22/04/2020; check.def file version of 09/03/2020

Datablock c010620_1_1 - ellipsoid plot

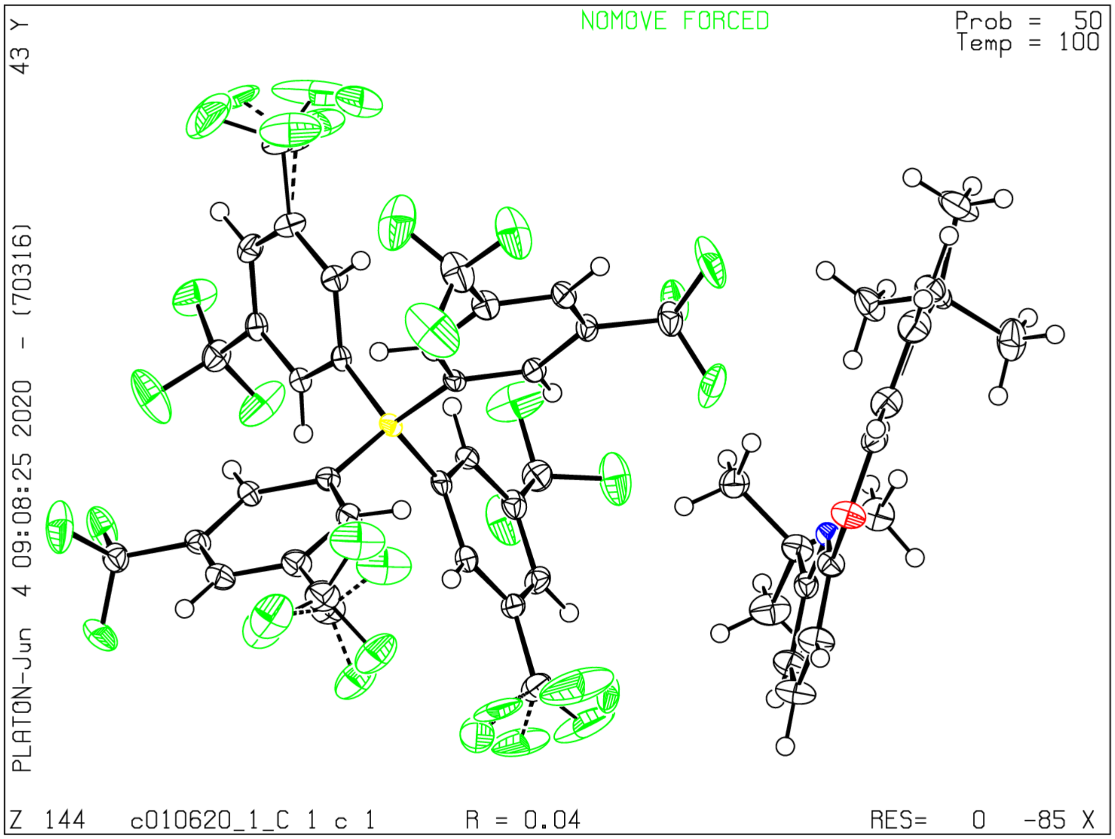

Supplement: Supplementary file 1 — Supporting Information [file CHEM-31-e02745-s002.zip › Crystal_structures/10b/checkcif.pdf]
