# Supplementary material for: London Dispersion versus Intramolecular Hydrogen Bond in Bis‐Pyridines: How Accurate Is DFT for Competing Noncovalent Interactions in the Condensed Phase?
Source: Chemistry. 2025 Oct 23;31(66):e02745. doi: 10.1002/chem.202502745 (PMC12648470; doi:10.1002/chem.202502745)
Supplement: Supplementary file 1 — Supporting Information [file CHEM-31-e02745-s002.zip › Crystal_structures/11b/c200123_1_2_tables.html]

c200123\_1\_2


# c200123\_1\_2

b"\n \n \n "

Table 1 Crystal data and structure refinement for c200123\_1\_2.

| Identification code | c200123\_1\_2 |
| Empirical formula | C44H25BF24N2 |
| Formula weight | 1048.47 |
| Temperature/K | 100.0(1) |
| Crystal system | monoclinic |
| Space group | P21/c |
| a/Å | 20.35010(10) |
| b/Å | 12.77770(10) |
| c/Å | 16.88380(10) |
| α/° | 90 |
| β/° | 103.7410(10) |
| γ/° | 90 |
| Volume/Å3 | 4264.60(5) |
| Z | 4 |
| ρcalcg/cm3 | 1.633 |
| μ/mm‑1 | 1.530 |
| F(000) | 2096.0 |
| Crystal size/mm3 | 0.321 × 0.249 × 0.188 |
| Radiation | Cu Kα (λ = 1.54184) |
| 2Θ range for data collection/° | 8.24 to 159.57 |
| Index ranges | -25 ≤ h ≤ 25, -16 ≤ k ≤ 15, -21 ≤ l ≤ 21 |
| Reflections collected | 149387 |
| Independent reflections | 9238 [Rint = 0.0349, Rsigma = 0.0118] |
| Data/restraints/parameters | 9238/467/708 |
| Goodness-of-fit on F2 | 1.025 |
| Final R indexes [I>=2σ (I)] | R1 = 0.0390, wR2 = 0.0922 |
| Final R indexes [all data] | R1 = 0.0407, wR2 = 0.0934 |
| Largest diff. peak/hole / e Å-3 | 0.47/-0.35 |

Table 2 Fractional Atomic Coordinates (×104) and Equivalent Isotropic Displacement Parameters (Å2×103) for c200123\_1\_2. Ueq is defined as 1/3 of the trace of the orthogonalised UIJ tensor.

| Atom | *x* | *y* | *z* | U(eq) |
| --- | --- | --- | --- | --- |
| F1 | 10270.6(12) | 1304(3) | 3502.9(14) | 59.3(9) |
| F2 | 9420.9(15) | 511.9(18) | 3695(3) | 85.4(17) |
| F3 | 9934(2) | 1663(3) | 4545.9(17) | 62.3(12) |
| F4 | 9056.3(5) | 4375.4(8) | 1108.7(6) | 37.1(2) |
| F5 | 9258.4(6) | 2752.9(9) | 919.1(7) | 47.5(3) |
| F6 | 8236.5(5) | 3311.5(8) | 709.7(5) | 37.5(2) |
| F7 | 9150.3(8) | 7465.5(17) | 3196.3(19) | 69.3(10) |
| F8 | 8483.8(9) | 8749.1(11) | 3021.7(14) | 53.4(7) |
| F9 | 8530.5(13) | 7804(2) | 2025.2(10) | 68.3(10) |
| F10 | 6054.9(5) | 8180.8(8) | 2717.5(7) | 39.5(2) |
| F11 | 5692.7(4) | 6630.4(8) | 2816.6(7) | 39.0(2) |
| F12 | 6143.0(5) | 7501.1(11) | 3897.2(6) | 51.0(3) |
| F13 | 9780.3(5) | 5940.5(9) | 5700.6(7) | 44.2(3) |
| F14 | 10074.3(5) | 4424.9(9) | 6154.9(8) | 46.5(3) |
| F15 | 9717.6(5) | 5523.0(12) | 6907.3(6) | 57.9(4) |
| F16 | 6776.0(5) | 3667.2(8) | 6172.0(6) | 34.5(2) |
| F17 | 7630.7(5) | 3602.4(10) | 7211.2(6) | 45.7(3) |
| F18 | 7171.3(5) | 5069.4(8) | 6795.1(6) | 36.5(2) |
| F19 | 5192.0(5) | 3723.4(9) | 1557.9(6) | 40.9(2) |
| F20 | 5975.9(5) | 3442.9(8) | 931.1(5) | 34.1(2) |
| F21 | 5334.5(4) | 2178.1(7) | 1126.4(5) | 31.3(2) |
| F22 | 7029.1(5) | -6.4(7) | 3741.2(6) | 34.7(2) |
| F23 | 5961.3(5) | 170.3(7) | 3625.8(6) | 35.9(2) |
| F24 | 6667.2(5) | 809.9(7) | 4661.8(5) | 33.4(2) |
| C1 | 8368.6(6) | 3564.6(10) | 3243.2(8) | 16.3(2) |
| C2 | 8408.0(6) | 3731.3(10) | 2437.7(8) | 18.2(3) |
| C3 | 8851.5(7) | 3171.8(11) | 2082.0(9) | 20.5(3) |
| C4 | 9273.8(7) | 2414.6(11) | 2517.9(9) | 23.8(3) |
| C5 | 9237.7(7) | 2227.7(12) | 3314.5(9) | 24.8(3) |
| C6 | 8796.4(7) | 2785.6(11) | 3670.6(8) | 20.5(3) |
| C7 | 8860.1(8) | 3395.2(12) | 1213.8(10) | 27.0(3) |
| C8 | 9722(3) | 1433(4) | 3772(4) | 28.4(11) |
| C9 | 7629.1(6) | 5332.6(10) | 3321.7(7) | 15.9(2) |
| C10 | 7005.1(6) | 5799.9(10) | 3315.8(8) | 17.4(2) |
| C11 | 6875.3(7) | 6854.6(11) | 3129.3(8) | 19.1(3) |
| C12 | 7373.1(7) | 7500.9(11) | 2958.7(8) | 20.8(3) |
| C13 | 7998.5(7) | 7059.5(11) | 2973.1(8) | 20.2(3) |
| C14 | 8123.0(6) | 5999.8(10) | 3143.5(8) | 17.5(2) |
| C15 | 6195.1(7) | 7290.0(12) | 3140.0(9) | 24.5(3) |
| C16 | 8551.3(8) | 7740.4(12) | 2803.6(9) | 27.4(3) |
| C17 | 8038.0(6) | 4229.7(10) | 4622.6(8) | 15.9(2) |
| C18 | 7611.1(6) | 4082.1(10) | 5154.2(8) | 17.0(2) |
| C19 | 7828.8(7) | 4271.9(10) | 5989.3(8) | 18.2(3) |
| C20 | 8479.5(7) | 4624.2(11) | 6330.2(8) | 19.2(3) |
| C21 | 8907.2(7) | 4786.3(10) | 5813.1(8) | 18.8(3) |
| C22 | 8691.6(6) | 4589.7(10) | 4980.4(8) | 17.2(2) |
| C23 | 7357.7(7) | 4136.2(12) | 6539.8(8) | 23.0(3) |
| C24 | 9615.5(7) | 5169.8(12) | 6149.0(9) | 24.1(3) |
| C25 | 7134.4(6) | 3353.5(10) | 3327.5(8) | 15.6(2) |
| C26 | 6653.9(6) | 3518.1(10) | 2591.7(8) | 16.9(2) |
| C27 | 6151.0(6) | 2791.8(11) | 2273.2(8) | 17.5(3) |
| C28 | 6098.9(6) | 1861.5(11) | 2678.4(8) | 18.2(3) |
| C29 | 6570.2(6) | 1678.5(10) | 3407.8(8) | 17.3(2) |
| C30 | 7077.6(6) | 2403.9(10) | 3722.8(8) | 16.8(2) |
| C31 | 5665.7(7) | 3028.0(12) | 1477.5(9) | 23.4(3) |
| C32 | 6556.2(7) | 667.7(11) | 3850.6(9) | 22.0(3) |
| B1 | 7790.6(7) | 4121.3(11) | 3628.1(9) | 14.9(3) |
| N1B | 7204.9(7) | 7435.6(10) | 5496.1(7) | 24.4(3) |
| N2B | 5994.8(7) | 6820.8(11) | 5708.3(8) | 29.4(3) |
| C1B | 7690.8(8) | 6937.3(12) | 5224.2(9) | 26.5(3) |
| C2B | 8238.3(8) | 7475.4(13) | 5092.4(9) | 28.4(3) |
| C3B | 8276.0(8) | 8546.1(13) | 5239.1(10) | 31.3(3) |
| C4B | 7768.9(8) | 9039.3(12) | 5524.3(9) | 29.1(3) |
| C5B | 7229.2(8) | 8472.2(11) | 5657.4(8) | 24.6(3) |
| C6B | 6673.6(8) | 8944.8(12) | 5991.4(9) | 27.3(3) |
| C7B | 5937.5(8) | 8723.0(13) | 5525.1(9) | 29.1(3) |
| C8B | 5631.6(8) | 7701.6(13) | 5707.7(9) | 26.9(3) |
| C9B | 4984.8(8) | 7664.4(14) | 5846.3(9) | 32.2(3) |
| C10B | 4705.5(8) | 6714.2(15) | 5976.0(10) | 35.4(4) |
| C11B | 5075.3(8) | 5810.1(14) | 5964.3(11) | 35.1(4) |
| C12B | 5717.1(8) | 5902.0(14) | 5827.5(11) | 34.2(4) |
| F7A | 8967(3) | 7168(4) | 2419(4) | 58.2(19) |
| F9A | 8350(3) | 8463(5) | 2303(5) | 79(3) |
| F8A | 8956(4) | 8065(7) | 3440(3) | 80(3) |
| C8A | 9592(5) | 1314(8) | 3839(6) | 34(2) |
| F3A | 10105(3) | 1669(5) | 4411(5) | 91(3) |
| F1A | 9856(4) | 654(5) | 3420(2) | 93(3) |
| F2A | 9257.2(19) | 764(3) | 4268(3) | 62.2(13) |

Table 3 Anisotropic Displacement Parameters (Å2×103) for c200123\_1\_2. The Anisotropic displacement factor exponent takes the form: -2π2[h2a\*2U11+2hka\*b\*U12+…].

| Atom | U11 | U22 | U33 | U23 | U13 | U12 |
| --- | --- | --- | --- | --- | --- | --- |
| F1 | 41.2(12) | 77.7(19) | 61.2(13) | 21.3(12) | 16.1(10) | 42.3(12) |
| F2 | 57.4(16) | 21.3(11) | 148(4) | 26.8(16) | -34(2) | -6.1(11) |
| F3 | 87(2) | 73(2) | 22.2(10) | 6.5(10) | 4.8(13) | 58.3(19) |
| F4 | 49.5(6) | 28.0(5) | 38.9(5) | -0.6(4) | 21.0(5) | -11.5(4) |
| F5 | 68.7(7) | 42.1(6) | 44.7(6) | 0.3(5) | 39.6(6) | 17.2(5) |
| F6 | 45.7(6) | 43.9(6) | 22.4(4) | -4.3(4) | 6.8(4) | -12.1(5) |
| F7 | 19.5(7) | 52.2(13) | 127(3) | 53.4(15) | -1.1(10) | -8.1(7) |
| F8 | 57.2(11) | 21.4(7) | 91.7(17) | -11.7(8) | 37.7(11) | -16.8(7) |
| F9 | 88.0(18) | 92(2) | 31.1(8) | -11.0(10) | 27.1(9) | -63.1(17) |
| F10 | 35.4(5) | 29.6(5) | 51.5(6) | 7.7(4) | 6.3(4) | 15.6(4) |
| F11 | 18.8(4) | 35.3(5) | 59.6(7) | -7.3(5) | 2.8(4) | 4.3(4) |
| F12 | 37.1(5) | 91.2(9) | 24.3(5) | -7.5(5) | 6.2(4) | 32.5(6) |
| F13 | 30.1(5) | 42.6(6) | 53.0(6) | 12.0(5) | -3.6(4) | -19.8(4) |
| F14 | 17.5(4) | 40.8(6) | 75.1(8) | -3.0(5) | -1.4(5) | 1.5(4) |
| F15 | 34.8(5) | 107.1(11) | 31.6(5) | -32.9(6) | 7.4(4) | -31.2(6) |
| F16 | 30.6(5) | 43.7(6) | 33.7(5) | -4.5(4) | 16.5(4) | -14.6(4) |
| F17 | 35.5(5) | 73.0(8) | 33.9(5) | 29.8(5) | 18.9(4) | 18.5(5) |
| F18 | 31.7(5) | 38.7(5) | 44.4(5) | -11.3(4) | 19.6(4) | 2.4(4) |
| F19 | 28.3(5) | 47.5(6) | 39.5(5) | -1.4(5) | -6.5(4) | 16.2(4) |
| F20 | 32.0(5) | 46.0(6) | 20.5(4) | 9.7(4) | -1.0(3) | -13.8(4) |
| F21 | 28.2(4) | 36.6(5) | 23.8(4) | -0.9(4) | -4.2(3) | -12.9(4) |
| F22 | 40.3(5) | 22.0(4) | 45.1(5) | 7.0(4) | 16.8(4) | 11.4(4) |
| F23 | 31.4(5) | 24.2(5) | 49.0(6) | 8.1(4) | 3.3(4) | -11.3(4) |
| F24 | 53.4(6) | 25.1(4) | 23.3(4) | 4.8(3) | 12.4(4) | -4.2(4) |
| C1 | 13.0(5) | 15.5(6) | 19.6(6) | -1.8(5) | 2.2(5) | -1.5(5) |
| C2 | 16.4(6) | 16.5(6) | 21.6(6) | -1.1(5) | 4.5(5) | -0.1(5) |
| C3 | 18.8(6) | 18.9(6) | 25.0(7) | -5.3(5) | 7.7(5) | -3.7(5) |
| C4 | 16.6(6) | 22.4(7) | 32.0(7) | -10.1(6) | 4.8(5) | 1.2(5) |
| C5 | 19.5(6) | 22.1(7) | 28.5(7) | -5.4(6) | -2.4(5) | 5.1(5) |
| C6 | 19.3(6) | 19.4(6) | 20.6(6) | -1.6(5) | 0.1(5) | 1.6(5) |
| C7 | 32.7(8) | 21.6(7) | 31.5(8) | -5.3(6) | 17.3(6) | -2.1(6) |
| C8 | 24(2) | 22.6(17) | 37(2) | -1.9(14) | 3.6(15) | 6.6(14) |
| C9 | 17.2(6) | 17.2(6) | 12.7(5) | -1.3(5) | 2.5(4) | -0.1(5) |
| C10 | 16.9(6) | 18.9(6) | 16.8(6) | -1.2(5) | 4.6(5) | -0.3(5) |
| C11 | 20.2(6) | 20.1(6) | 15.9(6) | -2.9(5) | 1.9(5) | 2.9(5) |
| C12 | 26.2(7) | 15.4(6) | 19.0(6) | -0.4(5) | 1.6(5) | 1.5(5) |
| C13 | 21.9(6) | 19.5(6) | 18.1(6) | 1.4(5) | 2.6(5) | -3.0(5) |
| C14 | 16.4(6) | 19.0(6) | 17.2(6) | -0.2(5) | 3.8(5) | 0.4(5) |
| C15 | 24.4(7) | 26.2(7) | 20.9(7) | -3.6(5) | 1.5(5) | 7.0(6) |
| C16 | 28.3(7) | 22.6(7) | 30.4(7) | 3.7(6) | 5.4(6) | -4.9(6) |
| C17 | 16.2(6) | 13.1(6) | 18.2(6) | 0.6(5) | 3.4(5) | 0.5(5) |
| C18 | 15.4(6) | 15.4(6) | 19.8(6) | 0.7(5) | 3.3(5) | -1.0(5) |
| C19 | 20.2(6) | 16.2(6) | 19.4(6) | 2.1(5) | 7.0(5) | 1.6(5) |
| C20 | 21.4(6) | 18.7(6) | 16.9(6) | -0.8(5) | 3.4(5) | 0.9(5) |
| C21 | 16.8(6) | 17.8(6) | 21.0(6) | -1.2(5) | 2.7(5) | -1.1(5) |
| C22 | 15.8(6) | 17.1(6) | 19.1(6) | -0.1(5) | 5.1(5) | -0.9(5) |
| C23 | 21.8(6) | 27.7(7) | 20.4(6) | 2.2(5) | 7.2(5) | 1.2(5) |
| C24 | 19.6(6) | 30.0(8) | 21.3(7) | -3.4(6) | 2.0(5) | -4.6(6) |
| C25 | 13.7(6) | 16.2(6) | 17.4(6) | -1.1(5) | 4.9(5) | 0.4(5) |
| C26 | 15.6(6) | 17.3(6) | 17.9(6) | 0.8(5) | 4.6(5) | 0.1(5) |
| C27 | 14.0(6) | 21.5(6) | 17.0(6) | -1.2(5) | 3.7(5) | 0.6(5) |
| C28 | 14.7(6) | 19.2(6) | 21.2(6) | -3.2(5) | 5.3(5) | -2.7(5) |
| C29 | 17.4(6) | 15.7(6) | 20.1(6) | -0.3(5) | 6.8(5) | 0.5(5) |
| C30 | 15.3(6) | 17.7(6) | 17.2(6) | -0.4(5) | 3.4(5) | 1.3(5) |
| C31 | 17.8(6) | 27.3(7) | 22.9(7) | 1.7(6) | 0.4(5) | -3.1(5) |
| C32 | 22.8(7) | 18.0(6) | 25.2(7) | -0.3(5) | 5.7(5) | -1.5(5) |
| B1 | 12.9(6) | 15.2(6) | 16.5(6) | 0.3(5) | 3.1(5) | -0.5(5) |
| N1B | 27.3(6) | 21.8(6) | 22.1(6) | -1.4(5) | 1.7(5) | 3.6(5) |
| N2B | 28.1(6) | 29.0(7) | 29.7(7) | -0.2(5) | 4.1(5) | 8.6(5) |
| C1B | 29.2(7) | 22.7(7) | 24.3(7) | -2.8(6) | 0.0(6) | 6.9(6) |
| C2B | 27.0(7) | 29.5(8) | 26.4(7) | -1.4(6) | 1.6(6) | 7.2(6) |
| C3B | 28.5(8) | 30.1(8) | 31.9(8) | 2.1(6) | 0.6(6) | 1.1(6) |
| C4B | 33.6(8) | 21.6(7) | 27.8(7) | -0.9(6) | -1.2(6) | 3.2(6) |
| C5B | 32.1(7) | 21.7(7) | 16.1(6) | -0.2(5) | -1.8(5) | 6.7(6) |
| C6B | 40.7(8) | 21.5(7) | 19.4(6) | -0.1(5) | 6.7(6) | 7.6(6) |
| C7B | 35.7(8) | 29.0(8) | 22.9(7) | 3.5(6) | 7.7(6) | 14.8(6) |
| C8B | 29.9(7) | 32.5(8) | 16.8(6) | -1.4(6) | 2.8(5) | 10.6(6) |
| C9B | 31.1(8) | 39.7(9) | 25.4(7) | -3.4(7) | 6.1(6) | 12.8(7) |
| C10B | 27.4(8) | 47.7(10) | 30.4(8) | -8.3(7) | 5.6(6) | 3.7(7) |
| C11B | 29.7(8) | 37.4(9) | 35.3(9) | -4.7(7) | 2.0(7) | -0.4(7) |
| C12B | 28.0(8) | 30.3(8) | 41.5(9) | -1.9(7) | 2.8(7) | 5.6(6) |
| F7A | 53(3) | 47(3) | 91(5) | -3(3) | 51(3) | -20(2) |
| F9A | 52(3) | 65(4) | 120(6) | 70(4) | 19(3) | 3(3) |
| F8A | 72(4) | 114(6) | 53(3) | -23(3) | 14(3) | -69(4) |
| C8A | 20(3) | 44(5) | 36(3) | -1(3) | 0(2) | 18(3) |
| F3A | 57(3) | 56(3) | 119(5) | 26(3) | -59(3) | -16(3) |
| F1A | 145(6) | 86(4) | 55(2) | 15(2) | 36(3) | 92(4) |
| F2A | 65(2) | 44(2) | 80(3) | 37.3(19) | 22(2) | 26.0(16) |

Table 4 Bond Lengths for c200123\_1\_2.

| Atom | Atom | Length/Å |  | Atom | Atom | Length/Å |
| --- | --- | --- | --- | --- | --- | --- |
| F1 | C8 | 1.311(6) |  | C13 | C14 | 1.3949(19) |
| F2 | C8 | 1.320(6) |  | C13 | C16 | 1.5023(19) |
| F3 | C8 | 1.309(6) |  | C16 | F7A | 1.392(5) |
| F4 | C7 | 1.3392(18) |  | C16 | F9A | 1.253(5) |
| F5 | C7 | 1.3305(17) |  | C16 | F8A | 1.259(5) |
| F6 | C7 | 1.3538(19) |  | C17 | C18 | 1.4021(18) |
| F7 | C16 | 1.290(2) |  | C17 | C22 | 1.4015(17) |
| F8 | C16 | 1.356(2) |  | C17 | B1 | 1.6403(19) |
| F9 | C16 | 1.307(2) |  | C18 | C19 | 1.3952(18) |
| F10 | C15 | 1.3377(18) |  | C19 | C20 | 1.3876(19) |
| F11 | C15 | 1.3373(18) |  | C19 | C23 | 1.4953(18) |
| F12 | C15 | 1.3352(17) |  | C20 | C21 | 1.3871(19) |
| F13 | C24 | 1.3327(18) |  | C21 | C22 | 1.3925(18) |
| F14 | C24 | 1.3318(18) |  | C21 | C24 | 1.5003(18) |
| F15 | C24 | 1.3265(17) |  | C25 | C26 | 1.4024(17) |
| F16 | C23 | 1.3405(17) |  | C25 | C30 | 1.4028(18) |
| F17 | C23 | 1.3266(17) |  | C25 | B1 | 1.6373(18) |
| F18 | C23 | 1.3527(17) |  | C26 | C27 | 1.3914(18) |
| F19 | C31 | 1.3416(18) |  | C27 | C28 | 1.3881(19) |
| F20 | C31 | 1.3444(17) |  | C27 | C31 | 1.4969(18) |
| F21 | C31 | 1.3399(17) |  | C28 | C29 | 1.3894(18) |
| F22 | C32 | 1.3367(17) |  | C29 | C30 | 1.3953(18) |
| F23 | C32 | 1.3400(16) |  | C29 | C32 | 1.4960(19) |
| F24 | C32 | 1.3466(17) |  | N1B | C1B | 1.3451(19) |
| C1 | C2 | 1.3976(18) |  | N1B | C5B | 1.3509(19) |
| C1 | C6 | 1.4040(18) |  | N2B | C8B | 1.3463(19) |
| C1 | B1 | 1.6355(18) |  | N2B | C12B | 1.339(2) |
| C2 | C3 | 1.3943(18) |  | C1B | C2B | 1.372(2) |
| C3 | C4 | 1.384(2) |  | C2B | C3B | 1.389(2) |
| C3 | C7 | 1.498(2) |  | C3B | C4B | 1.389(2) |
| C4 | C5 | 1.385(2) |  | C4B | C5B | 1.378(2) |
| C5 | C6 | 1.390(2) |  | C5B | C6B | 1.505(2) |
| C5 | C8 | 1.496(6) |  | C6B | C7B | 1.544(2) |
| C5 | C8A | 1.537(9) |  | C7B | C8B | 1.509(2) |
| C9 | C10 | 1.4011(18) |  | C8B | C9B | 1.391(2) |
| C9 | C14 | 1.4041(18) |  | C9B | C10B | 1.380(3) |
| C9 | B1 | 1.6403(19) |  | C10B | C11B | 1.382(2) |
| C10 | C11 | 1.3948(19) |  | C11B | C12B | 1.384(2) |
| C11 | C12 | 1.389(2) |  | C8A | F3A | 1.323(10) |
| C11 | C15 | 1.4960(19) |  | C8A | F1A | 1.298(10) |
| C12 | C13 | 1.387(2) |  | C8A | F2A | 1.310(9) |

Table 5 Bond Angles for c200123\_1\_2.

| Atom | Atom | Atom | Angle/˚ |  | Atom | Atom | Atom | Angle/˚ |
| --- | --- | --- | --- | --- | --- | --- | --- | --- |
| C2 | C1 | C6 | 115.87(12) |  | C21 | C22 | C17 | 122.25(12) |
| C2 | C1 | B1 | 122.23(11) |  | F16 | C23 | F18 | 104.94(11) |
| C6 | C1 | B1 | 121.39(12) |  | F16 | C23 | C19 | 112.96(12) |
| C3 | C2 | C1 | 122.25(13) |  | F17 | C23 | F16 | 108.09(12) |
| C2 | C3 | C7 | 118.88(13) |  | F17 | C23 | F18 | 105.85(12) |
| C4 | C3 | C2 | 120.87(13) |  | F17 | C23 | C19 | 112.99(12) |
| C4 | C3 | C7 | 120.24(13) |  | F18 | C23 | C19 | 111.45(12) |
| C3 | C4 | C5 | 117.86(13) |  | F13 | C24 | C21 | 112.05(12) |
| C4 | C5 | C6 | 121.35(13) |  | F14 | C24 | F13 | 105.17(12) |
| C4 | C5 | C8 | 115.9(3) |  | F14 | C24 | C21 | 112.23(12) |
| C4 | C5 | C8A | 124.4(4) |  | F15 | C24 | F13 | 107.10(13) |
| C6 | C5 | C8 | 122.6(3) |  | F15 | C24 | F14 | 106.69(13) |
| C6 | C5 | C8A | 113.7(4) |  | F15 | C24 | C21 | 113.09(12) |
| C5 | C6 | C1 | 121.78(13) |  | C26 | C25 | C30 | 115.60(12) |
| F4 | C7 | F6 | 104.78(13) |  | C26 | C25 | B1 | 122.04(11) |
| F4 | C7 | C3 | 112.41(12) |  | C30 | C25 | B1 | 121.61(11) |
| F5 | C7 | F4 | 107.45(12) |  | C27 | C26 | C25 | 122.44(12) |
| F5 | C7 | F6 | 106.23(13) |  | C26 | C27 | C31 | 118.70(12) |
| F5 | C7 | C3 | 113.47(13) |  | C28 | C27 | C26 | 121.06(12) |
| F6 | C7 | C3 | 111.92(12) |  | C28 | C27 | C31 | 120.24(12) |
| F1 | C8 | F2 | 105.6(4) |  | C27 | C28 | C29 | 117.61(12) |
| F1 | C8 | C5 | 114.7(4) |  | C28 | C29 | C30 | 121.24(12) |
| F2 | C8 | C5 | 108.7(4) |  | C28 | C29 | C32 | 119.81(12) |
| F3 | C8 | F1 | 105.6(4) |  | C30 | C29 | C32 | 118.90(12) |
| F3 | C8 | F2 | 109.5(5) |  | C29 | C30 | C25 | 122.03(12) |
| F3 | C8 | C5 | 112.5(4) |  | F19 | C31 | F20 | 105.90(12) |
| C10 | C9 | C14 | 115.69(12) |  | F19 | C31 | C27 | 112.30(12) |
| C10 | C9 | B1 | 121.18(11) |  | F20 | C31 | C27 | 112.23(11) |
| C14 | C9 | B1 | 122.72(11) |  | F21 | C31 | F19 | 106.47(11) |
| C11 | C10 | C9 | 122.41(12) |  | F21 | C31 | F20 | 106.55(12) |
| C10 | C11 | C15 | 118.86(13) |  | F21 | C31 | C27 | 112.89(12) |
| C12 | C11 | C10 | 120.86(12) |  | F22 | C32 | F23 | 106.68(11) |
| C12 | C11 | C15 | 120.26(13) |  | F22 | C32 | F24 | 105.97(11) |
| C13 | C12 | C11 | 117.77(12) |  | F22 | C32 | C29 | 112.81(11) |
| C12 | C13 | C14 | 121.29(13) |  | F23 | C32 | F24 | 105.90(11) |
| C12 | C13 | C16 | 119.12(13) |  | F23 | C32 | C29 | 113.03(12) |
| C14 | C13 | C16 | 119.60(13) |  | F24 | C32 | C29 | 111.91(11) |
| C13 | C14 | C9 | 121.96(12) |  | C1 | B1 | C9 | 113.22(10) |
| F10 | C15 | C11 | 113.01(13) |  | C1 | B1 | C17 | 111.63(10) |
| F11 | C15 | F10 | 105.84(12) |  | C1 | B1 | C25 | 103.11(10) |
| F11 | C15 | C11 | 112.37(12) |  | C17 | B1 | C9 | 103.47(10) |
| F12 | C15 | F10 | 106.46(12) |  | C25 | B1 | C9 | 112.34(10) |
| F12 | C15 | F11 | 106.94(13) |  | C25 | B1 | C17 | 113.41(10) |
| F12 | C15 | C11 | 111.77(11) |  | C1B | N1B | C5B | 122.56(14) |
| F7 | C16 | F8 | 105.28(18) |  | C12B | N2B | C8B | 118.81(14) |
| F7 | C16 | F9 | 109.5(2) |  | N1B | C1B | C2B | 120.63(14) |
| F7 | C16 | C13 | 113.77(14) |  | C1B | C2B | C3B | 118.51(15) |
| F8 | C16 | C13 | 111.71(14) |  | C2B | C3B | C4B | 119.63(16) |
| F9 | C16 | F8 | 103.43(18) |  | C5B | C4B | C3B | 120.30(15) |
| F9 | C16 | C13 | 112.38(14) |  | N1B | C5B | C4B | 118.37(14) |
| F7A | C16 | C13 | 110.5(2) |  | N1B | C5B | C6B | 118.31(14) |
| F9A | C16 | C13 | 114.5(3) |  | C4B | C5B | C6B | 123.31(14) |
| F9A | C16 | F7A | 102.2(4) |  | C5B | C6B | C7B | 117.37(12) |
| F9A | C16 | F8A | 112.0(5) |  | C8B | C7B | C6B | 116.87(12) |
| F8A | C16 | C13 | 113.3(3) |  | N2B | C8B | C7B | 118.05(14) |
| F8A | C16 | F7A | 103.0(4) |  | N2B | C8B | C9B | 120.82(16) |
| C18 | C17 | B1 | 123.90(11) |  | C9B | C8B | C7B | 121.10(14) |
| C22 | C17 | C18 | 115.93(12) |  | C10B | C9B | C8B | 119.91(15) |
| C22 | C17 | B1 | 119.87(11) |  | C9B | C10B | C11B | 119.15(16) |
| C19 | C18 | C17 | 121.82(12) |  | C10B | C11B | C12B | 118.05(17) |
| C18 | C19 | C23 | 120.76(12) |  | N2B | C12B | C11B | 123.24(16) |
| C20 | C19 | C18 | 121.22(12) |  | F3A | C8A | C5 | 109.9(7) |
| C20 | C19 | C23 | 117.99(12) |  | F1A | C8A | C5 | 112.2(7) |
| C21 | C20 | C19 | 117.84(12) |  | F1A | C8A | F3A | 105.3(8) |
| C20 | C21 | C22 | 120.95(12) |  | F1A | C8A | F2A | 106.9(8) |
| C20 | C21 | C24 | 120.06(12) |  | F2A | C8A | C5 | 119.5(6) |
| C22 | C21 | C24 | 118.99(12) |  | F2A | C8A | F3A | 101.8(7) |

Table 6 Torsion Angles for c200123\_1\_2.

| A | B | C | D | Angle/˚ |  | A | B | C | D | Angle/˚ |
| --- | --- | --- | --- | --- | --- | --- | --- | --- | --- | --- |
| C1 | C2 | C3 | C4 | 0.3(2) |  | C18 | C19 | C23 | F18 | -107.65(14) |
| C1 | C2 | C3 | C7 | 179.74(12) |  | C19 | C20 | C21 | C22 | -0.7(2) |
| C2 | C1 | C6 | C5 | 0.87(19) |  | C19 | C20 | C21 | C24 | 179.82(13) |
| C2 | C1 | B1 | C9 | -38.62(16) |  | C20 | C19 | C23 | F16 | -171.88(12) |
| C2 | C1 | B1 | C17 | -154.90(12) |  | C20 | C19 | C23 | F17 | -48.78(18) |
| C2 | C1 | B1 | C25 | 83.02(14) |  | C20 | C19 | C23 | F18 | 70.26(16) |
| C2 | C3 | C4 | C5 | 0.4(2) |  | C20 | C21 | C22 | C17 | 0.5(2) |
| C2 | C3 | C7 | F4 | 63.39(17) |  | C20 | C21 | C24 | F13 | -134.28(14) |
| C2 | C3 | C7 | F5 | -174.43(13) |  | C20 | C21 | C24 | F14 | 107.65(15) |
| C2 | C3 | C7 | F6 | -54.22(17) |  | C20 | C21 | C24 | F15 | -13.1(2) |
| C3 | C4 | C5 | C6 | -0.5(2) |  | C22 | C17 | C18 | C19 | -0.71(19) |
| C3 | C4 | C5 | C8 | -177.7(3) |  | C22 | C17 | B1 | C1 | 45.19(16) |
| C3 | C4 | C5 | C8A | 170.7(5) |  | C22 | C17 | B1 | C9 | -76.89(14) |
| C4 | C3 | C7 | F4 | -117.21(15) |  | C22 | C17 | B1 | C25 | 161.13(11) |
| C4 | C3 | C7 | F5 | 5.0(2) |  | C22 | C21 | C24 | F13 | 46.23(18) |
| C4 | C3 | C7 | F6 | 125.19(14) |  | C22 | C21 | C24 | F14 | -71.83(17) |
| C4 | C5 | C6 | C1 | -0.2(2) |  | C22 | C21 | C24 | F15 | 167.41(14) |
| C4 | C5 | C8 | F1 | 24.6(5) |  | C23 | C19 | C20 | C21 | -177.67(12) |
| C4 | C5 | C8 | F2 | -93.3(4) |  | C24 | C21 | C22 | C17 | 179.95(13) |
| C4 | C5 | C8 | F3 | 145.3(4) |  | C25 | C26 | C27 | C28 | 0.5(2) |
| C4 | C5 | C8A | F3A | 108.2(7) |  | C25 | C26 | C27 | C31 | -179.14(12) |
| C4 | C5 | C8A | F1A | -8.5(9) |  | C26 | C25 | C30 | C29 | -0.78(18) |
| C4 | C5 | C8A | F2A | -134.8(6) |  | C26 | C25 | B1 | C1 | -90.62(14) |
| C6 | C1 | C2 | C3 | -0.96(19) |  | C26 | C25 | B1 | C9 | 31.61(16) |
| C6 | C1 | B1 | C9 | 149.96(12) |  | C26 | C25 | B1 | C17 | 148.50(12) |
| C6 | C1 | B1 | C17 | 33.68(16) |  | C26 | C27 | C28 | C29 | -0.61(19) |
| C6 | C1 | B1 | C25 | -88.41(14) |  | C26 | C27 | C31 | F19 | -76.98(16) |
| C6 | C5 | C8 | F1 | -152.6(3) |  | C26 | C27 | C31 | F20 | 42.20(18) |
| C6 | C5 | C8 | F2 | 89.6(5) |  | C26 | C27 | C31 | F21 | 162.64(12) |
| C6 | C5 | C8 | F3 | -31.9(5) |  | C27 | C28 | C29 | C30 | 0.07(19) |
| C6 | C5 | C8A | F3A | -80.0(8) |  | C27 | C28 | C29 | C32 | -177.28(12) |
| C6 | C5 | C8A | F1A | 163.3(6) |  | C28 | C27 | C31 | F19 | 103.41(15) |
| C6 | C5 | C8A | F2A | 37.0(9) |  | C28 | C27 | C31 | F20 | -137.41(13) |
| C7 | C3 | C4 | C5 | -178.98(13) |  | C28 | C27 | C31 | F21 | -16.97(18) |
| C8 | C5 | C6 | C1 | 176.8(3) |  | C28 | C29 | C30 | C25 | 0.7(2) |
| C9 | C10 | C11 | C12 | -1.6(2) |  | C28 | C29 | C32 | F22 | 101.65(15) |
| C9 | C10 | C11 | C15 | -179.81(12) |  | C28 | C29 | C32 | F23 | -19.49(18) |
| C10 | C9 | C14 | C13 | 0.09(18) |  | C28 | C29 | C32 | F24 | -138.95(13) |
| C10 | C9 | B1 | C1 | 155.34(11) |  | C30 | C25 | C26 | C27 | 0.24(18) |
| C10 | C9 | B1 | C17 | -83.65(13) |  | C30 | C25 | B1 | C1 | 79.04(14) |
| C10 | C9 | B1 | C25 | 39.04(16) |  | C30 | C25 | B1 | C9 | -158.73(11) |
| C10 | C11 | C12 | C13 | 0.59(19) |  | C30 | C25 | B1 | C17 | -41.84(16) |
| C10 | C11 | C15 | F10 | -160.29(12) |  | C30 | C29 | C32 | F22 | -75.76(16) |
| C10 | C11 | C15 | F11 | -40.60(18) |  | C30 | C29 | C32 | F23 | 163.10(12) |
| C10 | C11 | C15 | F12 | 79.64(17) |  | C30 | C29 | C32 | F24 | 43.64(17) |
| C11 | C12 | C13 | C14 | 0.7(2) |  | C31 | C27 | C28 | C29 | 178.99(12) |
| C11 | C12 | C13 | C16 | -179.10(12) |  | C32 | C29 | C30 | C25 | 178.02(12) |
| C12 | C11 | C15 | F10 | 21.52(18) |  | B1 | C1 | C2 | C3 | -172.83(12) |
| C12 | C11 | C15 | F11 | 141.21(13) |  | B1 | C1 | C6 | C5 | 172.82(12) |
| C12 | C11 | C15 | F12 | -98.55(16) |  | B1 | C9 | C10 | C11 | 174.08(12) |
| C12 | C13 | C14 | C9 | -1.1(2) |  | B1 | C9 | C14 | C13 | -172.62(12) |
| C12 | C13 | C16 | F7 | 147.5(2) |  | B1 | C17 | C18 | C19 | -174.28(12) |
| C12 | C13 | C16 | F8 | 28.5(2) |  | B1 | C17 | C22 | C21 | 174.09(12) |
| C12 | C13 | C16 | F9 | -87.2(2) |  | B1 | C25 | C26 | C27 | 170.48(12) |
| C12 | C13 | C16 | F7A | -147.8(3) |  | B1 | C25 | C30 | C29 | -171.07(12) |
| C12 | C13 | C16 | F9A | -33.1(5) |  | N1B | C1B | C2B | C3B | -0.6(2) |
| C12 | C13 | C16 | F8A | 97.1(5) |  | N1B | C5B | C6B | C7B | -51.61(18) |
| C14 | C9 | C10 | C11 | 1.25(18) |  | N2B | C8B | C9B | C10B | -0.8(2) |
| C14 | C9 | B1 | C1 | -32.35(16) |  | C1B | N1B | C5B | C4B | 1.1(2) |
| C14 | C9 | B1 | C17 | 88.66(14) |  | C1B | N1B | C5B | C6B | -177.57(13) |
| C14 | C9 | B1 | C25 | -148.65(12) |  | C1B | C2B | C3B | C4B | 0.9(2) |
| C14 | C13 | C16 | F7 | -32.3(3) |  | C2B | C3B | C4B | C5B | -0.3(2) |
| C14 | C13 | C16 | F8 | -151.34(16) |  | C3B | C4B | C5B | N1B | -0.7(2) |
| C14 | C13 | C16 | F9 | 92.9(2) |  | C3B | C4B | C5B | C6B | 177.91(14) |
| C14 | C13 | C16 | F7A | 32.3(4) |  | C4B | C5B | C6B | C7B | 129.81(15) |
| C14 | C13 | C16 | F9A | 147.1(5) |  | C5B | N1B | C1B | C2B | -0.5(2) |
| C14 | C13 | C16 | F8A | -82.7(5) |  | C5B | C6B | C7B | C8B | 83.10(17) |
| C15 | C11 | C12 | C13 | 178.74(12) |  | C6B | C7B | C8B | N2B | -48.14(19) |
| C16 | C13 | C14 | C9 | 178.74(12) |  | C6B | C7B | C8B | C9B | 133.84(15) |
| C17 | C18 | C19 | C20 | 0.5(2) |  | C7B | C8B | C9B | C10B | 177.12(14) |
| C17 | C18 | C19 | C23 | 178.34(12) |  | C8B | N2B | C12B | C11B | -1.1(2) |
| C18 | C17 | C22 | C21 | 0.24(19) |  | C8B | C9B | C10B | C11B | -0.1(2) |
| C18 | C17 | B1 | C1 | -141.48(12) |  | C9B | C10B | C11B | C12B | 0.5(2) |
| C18 | C17 | B1 | C9 | 96.45(14) |  | C10B | C11B | C12B | N2B | 0.1(3) |
| C18 | C17 | B1 | C25 | -25.53(17) |  | C12B | N2B | C8B | C7B | -176.60(14) |
| C18 | C19 | C20 | C21 | 0.2(2) |  | C12B | N2B | C8B | C9B | 1.4(2) |
| C18 | C19 | C23 | F16 | 10.21(19) |  | C8A | C5 | C6 | C1 | -172.3(4) |
| C18 | C19 | C23 | F17 | 133.31(14) |  |  |  |  |  |  |

Table 7 Hydrogen Atom Coordinates (Å×104) and Isotropic Displacement Parameters (Å2×103) for c200123\_1\_2.

| Atom | *x* | *y* | *z* | U(eq) |
| --- | --- | --- | --- | --- |
| H2 | 8122.85 | 4243.44 | 2121.7 | 22 |
| H4 | 9578.63 | 2035.17 | 2278.33 | 29 |
| H6 | 8783.98 | 2635.68 | 4218 | 25 |
| H10 | 6657.65 | 5383.19 | 3443.63 | 21 |
| H12 | 7288.07 | 8221.07 | 2836.26 | 25 |
| H14 | 8554.93 | 5721.23 | 3138.85 | 21 |
| H18 | 7161.16 | 3846.48 | 4939.73 | 20 |
| H20 | 8627.24 | 4750.42 | 6899.36 | 23 |
| H22 | 8998.05 | 4703.51 | 4643.34 | 21 |
| H26 | 6672.35 | 4148.77 | 2299.86 | 20 |
| H28 | 5753.28 | 1367.65 | 2464.63 | 22 |
| H30 | 7394.29 | 2249.84 | 4220.29 | 20 |
| H1B | 6828(8) | 7083(14) | 5569(11) | 29 |
| H1BA | 7654.36 | 6205.88 | 5122.47 | 32 |
| H2B | 8583.94 | 7123.79 | 4905.04 | 34 |
| H3B | 8646.51 | 8938.82 | 5145 | 38 |
| H4B | 7794.32 | 9770.71 | 5627.93 | 35 |
| H6BA | 6739.14 | 9712.88 | 6016.5 | 33 |
| H6BB | 6729.54 | 8693.99 | 6558.3 | 33 |
| H7BA | 5649.5 | 9299.47 | 5642.1 | 35 |
| H7BB | 5918.22 | 8744.29 | 4933.89 | 35 |
| H9B | 4735.87 | 8292.25 | 5851.66 | 39 |
| H10B | 4264.51 | 6682.25 | 6072.13 | 42 |
| H11B | 4894.49 | 5144.78 | 6047.73 | 42 |
| H12B | 5973.09 | 5282.43 | 5817.68 | 41 |

Table 8 Atomic Occupancy for c200123\_1\_2.

| Atom | *Occupancy* |  | Atom | *Occupancy* |  | Atom | *Occupancy* |
| --- | --- | --- | --- | --- | --- | --- | --- |
| F1 | 0.617(5) |  | F2 | 0.617(5) |  | F3 | 0.617(5) |
| F7 | 0.734(4) |  | F8 | 0.734(4) |  | F9 | 0.734(4) |
| C8 | 0.617(5) |  | F7A | 0.266(4) |  | F9A | 0.266(4) |
| F8A | 0.266(4) |  | C8A | 0.383(5) |  | F3A | 0.383(5) |
| F1A | 0.383(5) |  | F2A | 0.383(5) |  |  |  |

Experimental

Single crystals of C44H25BF24N2
[c200123\_1\_2]
were
[].
A suitable crystal was selected and
[]
on a
XtaLAB Synergy, Dualflex, Pilatus 200K
diffractometer. The crystal was kept at 100.0(1) K during data collection.
Using Olex2 [1], the structure was solved with the
SHELXT
[2] structure solution program using
Intrinsic Phasing
and refined with the
SHELXL
[3] refinement package using
Least Squares
minimisation.

1. Dolomanov, O.V., Bourhis, L.J., Gildea, R.J, Howard, J.A.K. & Puschmann, H.
   (2009), J. Appl. Cryst. 42, 339-341.
2. Sheldrick, G.M. (2015). Acta Cryst. A71, 3-8.
3. Sheldrick, G.M. (2015). Acta Cryst. C71, 3-8.

Crystal structure determination of
[c200123\_1\_2]

**Crystal Data**
for C44H25BF24N2 (*M*=1048.47 g/mol):
monoclinic, space group P21/c (no. 14),
*a* = 20.35010(10) Å, *b* = 12.77770(10) Å, *c* = 16.88380(10) Å, *β* = 103.7410(10)°,
*V*= 4264.60(5) Å3,
*Z* = 4,
*T* = 100.0(1) K,
μ(Cu Kα) = 1.530 mm-1,
*Dcalc* = 1.633 g/cm3,
149387 reflections measured (8.24° ≤ 2Θ ≤ 159.57°),
9238 unique (*R*int = 0.0349, Rsigma = 0.0118) which were used in all calculations.
The final *R*1 was 0.0390
(I > 2σ(I)) and *wR*2 was 0.0934 (all data).

Refinement model description

Number of restraints - 467,
number of constraints - unknown.

Details:

```
1. Fixed Uiso
```

This report has been created with Olex2, compiled on
2022.04.07 svn.rca3783a0 for OlexSys. Please
let us know
if there are any errors or if you would like to have additional features.
