# Supplementary material for: London Dispersion versus Intramolecular Hydrogen Bond in Bis‐Pyridines: How Accurate Is DFT for Competing Noncovalent Interactions in the Condensed Phase?
Source: Chemistry. 2025 Oct 23;31(66):e02745. doi: 10.1002/chem.202502745 (PMC12648470; doi:10.1002/chem.202502745)

checkCIF/PLATON report

Structure factors have been supplied for datablock(s) c200123_1_2

THIS REPORT IS FOR GUIDANCE ONLY. IF USED AS PART OF A REVIEW PROCEDURE FOR PUBLICATION, IT SHOULD NOT REPLACE THE EXPERTISE OF AN EXPERIENCED CRYSTALLOGRAPHIC REFEREE.

No syntax errors found. CIF dictionary Interpreting this report

Datablock: c200123_1_2

Bond precision:	C-C = 0.0020 A		Wavelength=1.54184
Cell:	a=20.3501(1)	b=12.7777(1)	c=16.8838(1)
	alpha=90	beta=103.741(1)	gamma=90
Temperature:	100 K		

	Calculated	Reported
Volume	4264.60(5)	4264.60(5)
Space group	P 21/c	P 1 21/c 1
Hall group	-P 2ybc	-P 2ybc
Moiety formula	C32 H12 B F24, C12 H13 N2	C32 H12 B F24, C12 H13 N2
Sum formula	C44 H25 B F24 N2	C44 H25 B F24 N2
Mr	1048.47	1048.47
Dx,g cm-3	1.633	1.633
Z	4	4
Mu (mm-1)	1.530	1.530
F000	2096.0	2096.0
F000'	2106.20	
h,k,lmax	25,16,21	25,16,21
Nref	9293	9238
Tmin,Tmax	0.665,0.750	0.382,1.000
Tmin'	0.583	

Correction method= # Reported T Limits: Tmin=0.382 Tmax=1.000
AbsCorr = GAUSSIAN

Data completeness= 0.994 Theta(max)= 79.785

R(reflections)= 0.0390(8758) wR2(reflections)= 0.0934(9238)

S = 1.025 Npar= 708

The following ALERTS were generated. Each ALERT has the format
test-name_ALERT_alert-type_alert-level.
Click on the hyperlinks for more details of the test.

Alert level C

```
PLAT213_ALERT_2_C Atom F2             has ADP max/min Ratio .....        3.6 prolat
PLAT213_ALERT_2_C Atom F7             has ADP max/min Ratio .....        3.2 prolat
PLAT213_ALERT_2_C Atom F1A            has ADP max/min Ratio .....        3.5 prolat
PLAT213_ALERT_2_C Atom F3A            has ADP max/min Ratio .....        3.4 prolat
PLAT213_ALERT_2_C Atom F9A            has ADP max/min Ratio .....        3.2 prolat
PLAT242_ALERT_2_C Low    'MainMol' Ueq as Compared to Neighbors of       C24 Check
PLAT906_ALERT_3_C Large K Value in the Analysis of Variance ......     2.327 Check
```

Alert level G

```
PLAT002_ALERT_2_G Number of Distance or Angle Restraints on AtSite        18 Note
PLAT003_ALERT_2_G Number of Uiso or Uij Restrained non-H Atoms ...        16 Report
PLAT142_ALERT_4_G s.u. on b - Axis Small or Missing .............    0.00010 Ang.
PLAT143_ALERT_4_G s.u. on c - Axis Small or Missing .............    0.00010 Ang.
PLAT153_ALERT_1_G The s.u.'s on the Cell Axes   are Equal ..(Note)    0.0001 Ang.
PLAT172_ALERT_4_G The CIF-Embedded .res File Contains DFIX Records         1 Report
PLAT176_ALERT_4_G The CIF-Embedded .res File Contains SADI Records         5 Report
PLAT178_ALERT_4_G The CIF-Embedded .res File Contains SIMU Records         2 Report
PLAT186_ALERT_4_G The CIF-Embedded .res File Contains ISOR Records         1 Report
PLAT187_ALERT_4_G The CIF-Embedded .res File Contains RIGU Records         2 Report
PLAT242_ALERT_2_G Low    'MainMol' Ueq as Compared to Neighbors of        C7 Check
PLAT242_ALERT_2_G Low    'MainMol' Ueq as Compared to Neighbors of       C16 Check
PLAT301_ALERT_3_G Main Residue  Disorder .............(Resd  1  )        12% Note
PLAT434_ALERT_2_G Short Inter HL..HL Contact F4       ..F1A      .      2.71 Ang.
                                          2-x,1/2+y,1/2-z  =      2_755 Check
PLAT720_ALERT_4_G Number of Unusual/Non-Standard Labels .........          5 Note
PLAT860_ALERT_3_G Number of Least-Squares Restraints ............        467 Note
PLAT910_ALERT_3_G Missing # of FCF Reflection(s) Below Theta(Min).         1 Note
PLAT912_ALERT_4_G Missing # of FCF Reflections Above STh/L=  0.600        55 Note
PLAT978_ALERT_2_G Number C-C Bonds with Positive Residual Density.        11 Info
```

0 **ALERT level A** = Most likely a serious problem - resolve or explain
0 **ALERT level B** = A potentially serious problem, consider carefully
7 **ALERT level C** = Check. Ensure it is not caused by an omission or oversight
19 **ALERT level G** = General information/check it is not something unexpected

1 ALERT type 1 CIF construction/syntax error, inconsistent or missing data
12 ALERT type 2 Indicator that the structure model may be wrong or deficient
4 ALERT type 3 Indicator that the structure quality may be low
9 ALERT type 4 Improvement, methodology, query or suggestion
0 ALERT type 5 Informative message, check

It is advisable to attempt to resolve as many as possible of the alerts in all categories. Often the minor alerts point to easily fixed oversights, errors and omissions in your CIF or refinement strategy, so attention to these fine details can be worthwhile. In order to resolve some of the more serious problems it may be necessary to carry out additional measurements or structure refinements. However, the purpose of your study may justify the reported deviations and the more serious of these should normally be commented upon in the discussion or experimental section of a paper or in the "special_details" fields of the CIF. checkCIF was carefully designed to identify outliers and unusual parameters, but every test has its limitations and alerts that are not important in a particular case may appear. Conversely, the absence of alerts does not guarantee there are no aspects of the results needing attention. It is up to the individual to critically assess their own results and, if necessary, seek expert advice.

Publication of your CIF in IUCr journals

A basic structural check has been run on your CIF. These basic checks will be run on all CIFs submitted for publication in IUCr journals (*Acta Crystallographica*, *Journal of Applied Crystallography*, *Journal of Synchrotron Radiation*); however, if you intend to submit to *Acta Crystallographica Section C* or *E* or *IUCrData*, you should make sure that full publication checks are run on the final version of your CIF prior to submission.

Publication of your CIF in other journals

Please refer to the *Notes for Authors* of the relevant journal for any special instructions relating to CIF submission.

PLATON version of 28/11/2022; check.def file version of 28/11/2022

Datablock c200123_1_2 - ellipsoid plot

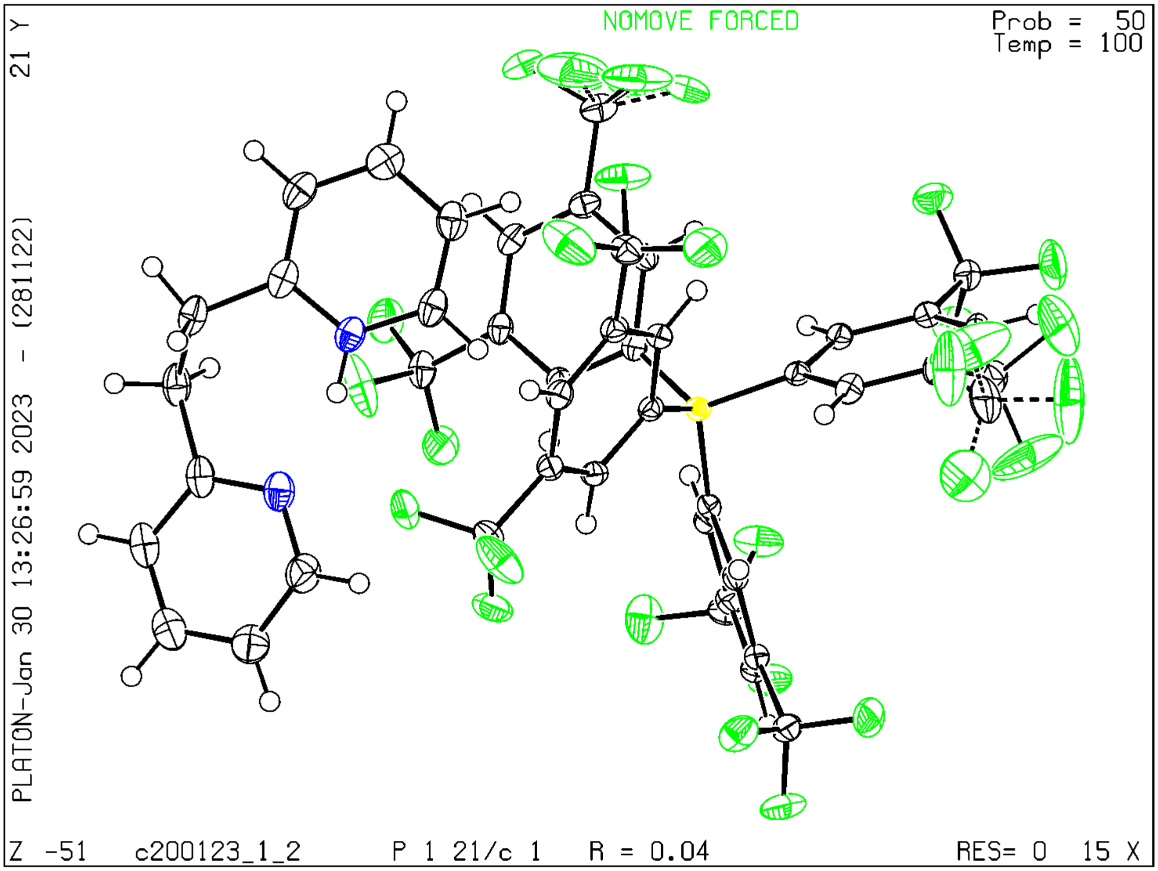

Supplement: Supplementary file 1 — Supporting Information [file CHEM-31-e02745-s002.zip › Crystal_structures/11b/checkcif.pdf]
