# Supplementary material for: London Dispersion versus Intramolecular Hydrogen Bond in Bis‐Pyridines: How Accurate Is DFT for Competing Noncovalent Interactions in the Condensed Phase?
Source: Chemistry. 2025 Oct 23;31(66):e02745. doi: 10.1002/chem.202502745 (PMC12648470; doi:10.1002/chem.202502745)
Supplement: Supplementary file 1 — Supporting Information [file CHEM-31-e02745-s002.zip › Crystal_structures/12b/c020620_1_1_tables.html]

c020620\_1\_1


# c020620\_1\_1

Table 1 Crystal data and structure refinement for c020620\_1\_1.

| Identification code | c020620\_1\_1 |
| Empirical formula | C46H29BF24N2 |
| Formula weight | 1076.47 |
| Temperature/K | 100.0(1) |
| Crystal system | monoclinic |
| Space group | P21/c |
| a/Å | 12.7494(14) |
| b/Å | 21.745(2) |
| c/Å | 16.4749(17) |
| α/° | 90 |
| β/° | 93.000(3) |
| γ/° | 90 |
| Volume/Å3 | 4561.1(8) |
| Z | 4 |
| ρcalcg/cm3 | 1.568 |
| μ/mm‑1 | 0.160 |
| F(000) | 2160.0 |
| Crystal size/mm3 | 0.23 × 0.19 × 0.08 |
| Radiation | MoKα (λ = 0.71073) |
| 2Θ range for data collection/° | 3.104 to 56.686 |
| Index ranges | -17 ≤ h ≤ 17, -28 ≤ k ≤ 29, -21 ≤ l ≤ 21 |
| Reflections collected | 85974 |
| Independent reflections | 11357 [Rint = 0.0370, Rsigma = 0.0210] |
| Data/restraints/parameters | 11357/575/847 |
| Goodness-of-fit on F2 | 1.023 |
| Final R indexes [I>=2σ (I)] | R1 = 0.0388, wR2 = 0.0910 |
| Final R indexes [all data] | R1 = 0.0543, wR2 = 0.0988 |
| Largest diff. peak/hole / e Å-3 | 0.35/-0.27 |

Table 2 Fractional Atomic Coordinates (×104) and Equivalent Isotropic Displacement Parameters (Å2×103) for c020620\_1\_1. Ueq is defined as 1/3 of of the trace of the orthogonalised UIJ tensor.

| Atom | *x* | *y* | *z* | U(eq) |
| --- | --- | --- | --- | --- |
| F1 | 5307.9(9) | 6821.6(6) | 1146.9(8) | 57.1(3) |
| F2 | 6130.7(12) | 7143.0(7) | 136.4(8) | 80.5(5) |
| F3 | 6563.9(12) | 7459.5(5) | 1332.8(11) | 79.7(5) |
| F4 | 9543.5(9) | 5633.8(5) | -418.1(6) | 44.6(3) |
| F5 | 9881.1(9) | 6556.0(4) | -13.9(6) | 43.5(3) |
| F6 | 10467.7(8) | 5788.2(5) | 689.7(6) | 43.3(3) |
| F7 | 8014(6) | 7148(4) | 3805(4) | 40.5(17) |
| F8 | 9591(4) | 6902(4) | 4093(5) | 54(2) |
| F9 | 8515(7) | 6980(4) | 5028(3) | 61(2) |
| F10 | 9158(5) | 4888(4) | 6012(5) | 37.0(12) |
| F11 | 8869(6) | 4124(3) | 5229(4) | 37.2(13) |
| F12 | 7592(5) | 4562(6) | 5777(7) | 58(2) |
| F13 | 11402.1(7) | 4356.5(5) | 2300.5(7) | 42.5(2) |
| F14 | 11086.1(7) | 3736.0(5) | 3259.6(5) | 35.7(2) |
| F15 | 11484.1(7) | 3385.3(5) | 2099.4(7) | 48.5(3) |
| F16 | 8508(4) | 2487(3) | 686(4) | 34.7(11) |
| F17 | 6993(6) | 2699(4) | 1093(6) | 48.2(12) |
| F18 | 7597(7) | 3194(2) | 113(4) | 44.8(13) |
| F19 | 4328.9(9) | 4548.5(5) | 4497.5(6) | 44.8(3) |
| F20 | 4906.1(8) | 3654.4(5) | 4253.2(7) | 45.8(3) |
| F21 | 3345.4(8) | 3896.7(6) | 3858.9(7) | 51.6(3) |
| F22 | 2907(3) | 4369(5) | 967(3) | 70(2) |
| F23 | 3678(5) | 5211(2) | 690(3) | 68.9(12) |
| F24 | 4308(7) | 4347(4) | 329(5) | 71(3) |
| C1 | 7579.1(10) | 5494.4(6) | 1699.8(7) | 16.9(2) |
| C2 | 6861.8(11) | 5977.5(6) | 1565.9(8) | 21.6(3) |
| C3 | 7028.9(12) | 6447.2(7) | 1018.0(9) | 26.5(3) |
| C4 | 7930.1(13) | 6463.2(6) | 583.4(8) | 26.7(3) |
| C5 | 8654.4(11) | 5995.5(6) | 707.2(8) | 22.8(3) |
| C6 | 8476.6(10) | 5518.9(6) | 1245.9(8) | 19.0(3) |
| C7 | 6256.5(16) | 6959.7(8) | 904.0(11) | 43.5(4) |
| C8 | 9630.9(13) | 5991.9(7) | 243.9(9) | 31.5(3) |
| C9 | 7858.3(9) | 5254.3(6) | 3278.8(7) | 14.7(2) |
| C10 | 8038.6(9) | 5881.5(6) | 3414.9(8) | 16.3(2) |
| C11 | 8366.8(10) | 6111.4(6) | 4174.5(8) | 17.8(2) |
| C12 | 8520.1(10) | 5727.3(6) | 4840.8(8) | 19.2(3) |
| C13 | 8360.4(10) | 5103.1(6) | 4721.0(8) | 17.5(2) |
| C14 | 8050.4(9) | 4873.0(6) | 3956.9(7) | 15.9(2) |
| C15 | 8590.6(12) | 6782.2(7) | 4273.0(9) | 26.1(3) |
| C16 | 8479.8(11) | 4668.8(7) | 5422.7(8) | 23.3(3) |
| C17 | 8115.3(9) | 4362.8(6) | 2178.5(7) | 15.1(2) |
| C18 | 9177.6(10) | 4307.9(6) | 2429.0(7) | 17.3(2) |
| C19 | 9794.0(10) | 3821.5(6) | 2183.6(8) | 18.9(3) |
| C20 | 9379.3(10) | 3362.1(6) | 1684.3(8) | 19.4(3) |
| C21 | 8330.2(10) | 3409.9(6) | 1421.4(8) | 18.4(2) |
| C22 | 7715.3(10) | 3898.5(6) | 1662.9(7) | 17.2(2) |
| C23 | 10934.5(11) | 3820.3(7) | 2453.1(9) | 26.4(3) |
| C24 | 7860.7(11) | 2953.5(6) | 829.1(9) | 24.4(3) |
| C25 | 6189.3(9) | 4769.7(6) | 2435.5(8) | 16.0(2) |
| C26 | 5748.1(9) | 4551.9(6) | 3140.4(8) | 16.8(2) |
| C27 | 4720.7(10) | 4335.4(6) | 3144.8(8) | 20.2(3) |
| C28 | 4075.6(10) | 4336.0(7) | 2442.2(9) | 25.6(3) |
| C29 | 4490.4(11) | 4552.5(7) | 1734.5(9) | 27.1(3) |
| C30 | 5517.9(10) | 4762.7(7) | 1731.0(8) | 21.8(3) |
| C31 | 4320.0(11) | 4109.0(7) | 3929.4(9) | 25.2(3) |
| C32 | 3845(6) | 4636(4) | 929(5) | 42.4(17) |
| B1 | 7427.5(10) | 4972.4(6) | 2399.1(8) | 14.6(3) |
| N1B | 6097.8(10) | 1881.6(6) | 2325.0(8) | 27.8(3) |
| N2B | 7884.3(10) | 2236.2(5) | 3091.6(7) | 26.4(3) |
| C1B | 5130.5(14) | 2847.9(8) | 2230.8(12) | 40.1(4) |
| C2B | 5247.5(12) | 2182.7(7) | 2017.2(10) | 32.2(3) |
| C3B | 4521.3(13) | 1868.6(8) | 1525.1(11) | 38.8(4) |
| C4B | 4686.4(14) | 1253.9(9) | 1358.3(11) | 42.8(4) |
| C5B | 5571.3(13) | 961.4(8) | 1681.4(11) | 37.1(4) |
| C6B | 6288.0(13) | 1283.4(7) | 2172.5(10) | 30.7(3) |
| C7B | 7283.5(14) | 1010.0(7) | 2541.6(11) | 36.0(4) |
| C8B | 8293.6(13) | 1331.7(7) | 2289.7(10) | 33.6(3) |
| C9B | 8636.7(13) | 1871.2(7) | 2814.9(9) | 28.0(3) |
| C10B | 9689.8(13) | 1981.8(7) | 3006.7(9) | 31.9(3) |
| C11B | 9962.6(13) | 2472.3(8) | 3505.1(10) | 34.0(3) |
| C12B | 9187.0(13) | 2838.5(7) | 3799.8(9) | 31.4(3) |
| C13B | 8147.0(12) | 2712.4(7) | 3583.8(8) | 26.6(3) |
| C14B | 7261.4(14) | 3086.8(7) | 3881.4(10) | 32.5(3) |
| F9A | 9555(6) | 6926(6) | 4412(9) | 65(4) |
| F7A | 8055(11) | 7019(5) | 4885(6) | 71(4) |
| F8A | 8237(10) | 7107(5) | 3623(6) | 40(2) |
| C32A | 3785(8) | 4495(5) | 995(7) | 34(2) |
| F24A | 4323(7) | 4452(5) | 323(6) | 37.5(18) |
| F23A | 3214(10) | 5005(5) | 908(6) | 74(4) |
| F22A | 3122(7) | 4026(5) | 999(4) | 55(2) |
| F12A | 8902(14) | 4894(6) | 6091(7) | 59(3) |
| F11A | 7522(7) | 4457(7) | 5608(10) | 44(2) |
| F10A | 9000(8) | 4166(5) | 5234(6) | 38.0(18) |
| F8B | 7717(10) | 7105(10) | 4350(20) | 63(8) |
| F7B | 9197(18) | 6906(9) | 4932(10) | 39(5) |
| F9B | 9060(30) | 7025(9) | 3660(9) | 58(7) |
| F16A | 7748(10) | 3185(5) | 81(5) | 50(2) |
| F18A | 6893(9) | 2781(7) | 994(9) | 71(3) |
| F17A | 8393(10) | 2443(5) | 772(8) | 65(3) |
| C32B | 3747(15) | 4507(11) | 1004(11) | 34(5) |
| F23B | 2861(18) | 4797(15) | 1116(15) | 45(6) |
| F22B | 3550(30) | 3924(9) | 830(20) | 91(10) |
| F24B | 4174(17) | 4766(18) | 376(13) | 66(8) |

Table 3 Anisotropic Displacement Parameters (Å2×103) for c020620\_1\_1. The Anisotropic displacement factor exponent takes the form: -2π2[h2a\*2U11+2hka\*b\*U12+…].

| Atom | U11 | U22 | U33 | U23 | U13 | U12 |
| --- | --- | --- | --- | --- | --- | --- |
| F1 | 47.5(6) | 54.9(7) | 69.9(8) | 26.6(6) | 12.7(6) | 26.2(5) |
| F2 | 95.4(10) | 90.3(10) | 57.6(8) | 49.7(7) | 21.9(7) | 54.5(9) |
| F3 | 89.8(10) | 28.4(6) | 121.4(13) | -10.6(7) | 11.4(9) | 19.2(6) |
| F4 | 66.5(7) | 37.4(5) | 32.3(5) | -12.2(4) | 26.3(5) | -10.8(5) |
| F5 | 63.5(7) | 29.6(5) | 40.4(5) | 1.2(4) | 30.2(5) | -14.5(5) |
| F6 | 32.1(5) | 51.4(6) | 48.2(6) | 4.2(5) | 18.6(4) | -6.1(4) |
| F7 | 45(2) | 18.6(14) | 55(4) | -8(2) | -23(3) | 5.1(15) |
| F8 | 28.8(17) | 24.3(15) | 109(6) | 0(3) | 7(3) | -14.0(15) |
| F9 | 127(6) | 24.4(18) | 29.2(13) | -12.5(10) | -3(2) | -11(4) |
| F10 | 51(2) | 36.1(17) | 21.6(19) | 3.2(14) | -18.6(13) | -3.9(13) |
| F11 | 70(3) | 21.0(15) | 19.2(19) | -1.0(12) | -8.9(17) | 4.7(16) |
| F12 | 30.5(17) | 97(5) | 49(4) | 48(3) | 16.0(18) | 12.7(19) |
| F13 | 16.6(4) | 52.2(6) | 58.7(7) | 9.5(5) | 1.7(4) | -5.3(4) |
| F14 | 29.3(5) | 52.1(6) | 24.7(4) | -1.8(4) | -9.2(3) | 2.0(4) |
| F15 | 25.6(5) | 67.2(7) | 51.8(6) | -30.1(5) | -7.2(4) | 23.1(5) |
| F16 | 28.3(17) | 31.3(18) | 44.4(18) | -25.0(13) | 2.5(13) | 4.0(11) |
| F17 | 49(2) | 45.8(19) | 53(2) | -22.8(17) | 27.7(17) | -31.5(16) |
| F18 | 70(3) | 26(2) | 35(2) | -9.1(14) | -26.0(19) | 4.7(19) |
| F19 | 70.3(7) | 34.2(5) | 32.0(5) | -3.9(4) | 23.3(5) | -9.4(5) |
| F20 | 43.5(6) | 44.3(6) | 51.5(6) | 23.7(5) | 20.0(5) | 13.9(5) |
| F21 | 26.4(5) | 77.6(8) | 51.5(6) | 12.1(6) | 8.2(4) | -23.1(5) |
| F22 | 27.0(18) | 149(6) | 32.2(14) | 2(3) | -9.4(12) | -38(3) |
| F23 | 59(3) | 98(3) | 46.9(19) | 20.5(18) | -25.2(17) | 9(2) |
| F24 | 53(3) | 136(6) | 25(3) | -31(3) | -2(2) | -16(3) |
| C1 | 20.2(6) | 18.0(6) | 12.5(5) | -2.1(5) | -0.1(4) | -2.7(5) |
| C2 | 25.3(7) | 22.5(7) | 17.1(6) | 0.2(5) | 1.2(5) | 1.9(5) |
| C3 | 37.6(8) | 21.2(7) | 20.7(7) | 1.2(5) | 0.3(6) | 3.5(6) |
| C4 | 44.2(9) | 19.7(7) | 16.4(6) | 2.2(5) | 3.3(6) | -4.4(6) |
| C5 | 31.6(7) | 21.7(7) | 15.5(6) | -3.0(5) | 5.0(5) | -6.7(5) |
| C6 | 22.8(6) | 18.4(6) | 16.1(6) | -1.3(5) | 2.7(5) | -2.6(5) |
| C7 | 56.4(11) | 33.5(9) | 41.6(10) | 15.0(8) | 11.3(8) | 15.2(8) |
| C8 | 44.8(9) | 25.1(7) | 26.2(7) | -0.5(6) | 16.3(7) | -8.4(7) |
| C9 | 11.4(5) | 17.5(6) | 15.5(5) | -1.4(5) | 2.3(4) | -1.9(4) |
| C10 | 14.5(5) | 17.1(6) | 17.4(6) | 0.3(5) | 1.4(4) | -1.4(4) |
| C11 | 16.5(6) | 16.8(6) | 20.0(6) | -3.3(5) | 1.0(5) | -2.7(5) |
| C12 | 17.9(6) | 22.5(6) | 17.1(6) | -4.4(5) | -0.4(5) | -2.3(5) |
| C13 | 15.5(6) | 20.9(6) | 16.2(6) | 1.2(5) | 1.7(4) | -1.0(5) |
| C14 | 13.7(5) | 16.0(6) | 18.0(6) | -1.7(5) | 1.3(4) | -1.6(4) |
| C15 | 31.8(7) | 20.0(7) | 25.9(7) | -3.7(5) | -4.8(6) | -4.1(6) |
| C16 | 25.2(7) | 26.6(7) | 18.1(6) | 1.2(5) | -0.7(5) | -1.1(5) |
| C17 | 15.7(6) | 16.5(6) | 13.4(5) | 1.5(4) | 3.8(4) | -1.7(5) |
| C18 | 17.5(6) | 20.4(6) | 14.2(6) | -1.1(5) | 1.4(4) | -1.3(5) |
| C19 | 17.0(6) | 25.0(7) | 14.9(6) | 1.4(5) | 2.4(5) | 2.5(5) |
| C20 | 22.4(6) | 20.9(6) | 15.3(6) | 0.9(5) | 5.3(5) | 3.1(5) |
| C21 | 22.1(6) | 18.4(6) | 15.1(6) | -1.3(5) | 4.8(5) | -2.7(5) |
| C22 | 15.9(6) | 19.7(6) | 16.1(6) | -0.2(5) | 2.2(4) | -2.7(5) |
| C23 | 19.4(6) | 35.2(8) | 24.6(7) | -5.4(6) | 0.3(5) | 6.2(6) |
| C24 | 25.6(7) | 22.4(7) | 25.8(7) | -7.7(5) | 6.4(5) | -2.2(5) |
| C25 | 14.7(5) | 15.1(6) | 18.3(6) | -3.5(5) | 0.6(4) | 0.9(4) |
| C26 | 14.7(6) | 15.0(6) | 20.6(6) | -1.9(5) | 0.9(5) | 0.4(4) |
| C27 | 17.1(6) | 16.8(6) | 27.3(7) | -3.7(5) | 4.9(5) | -0.3(5) |
| C28 | 14.3(6) | 30.3(7) | 32.2(7) | -10.2(6) | 2.3(5) | -4.2(5) |
| C29 | 17.5(6) | 39.6(8) | 23.9(7) | -9.8(6) | -2.2(5) | -1.0(6) |
| C30 | 18.0(6) | 29.7(7) | 17.9(6) | -4.2(5) | 1.1(5) | 0.6(5) |
| C31 | 18.7(6) | 24.2(7) | 33.1(8) | 1.0(6) | 6.5(5) | -2.4(5) |
| C32 | 21(2) | 81(4) | 25(2) | -6(2) | -3.6(17) | -17(3) |
| B1 | 14.5(6) | 15.6(6) | 13.8(6) | -1.1(5) | 0.6(5) | -1.1(5) |
| N1B | 33.1(7) | 21.2(6) | 29.8(7) | -3.6(5) | 7.8(5) | -5.0(5) |
| N2B | 36.5(7) | 20.1(6) | 22.8(6) | -0.5(5) | 2.3(5) | 0.2(5) |
| C1B | 37.9(9) | 27.3(8) | 55.6(11) | -1.3(8) | 8.6(8) | 2.0(7) |
| C2B | 31.0(8) | 29.4(8) | 37.4(8) | -1.7(6) | 12.5(6) | -4.0(6) |
| C3B | 29.8(8) | 43.4(10) | 44.1(10) | -4.4(8) | 9.8(7) | -5.7(7) |
| C4B | 35.5(9) | 48.3(10) | 45.9(10) | -17.4(8) | 14.2(8) | -17.8(8) |
| C5B | 39.9(9) | 27.5(8) | 45.7(10) | -12.9(7) | 19.3(8) | -12.0(7) |
| C6B | 39.6(8) | 21.3(7) | 32.6(8) | -4.4(6) | 15.6(7) | -7.2(6) |
| C7B | 51.7(10) | 17.2(7) | 39.7(9) | -3.9(6) | 8.3(7) | 0.5(7) |
| C8B | 39.3(9) | 26.2(8) | 35.7(8) | -8.2(6) | 4.6(7) | 5.0(6) |
| C9B | 39.6(8) | 22.4(7) | 21.8(7) | 2.0(5) | 1.4(6) | 3.2(6) |
| C10B | 37.4(8) | 30.4(8) | 27.6(8) | 3.9(6) | -0.1(6) | 7.0(6) |
| C11B | 36.8(8) | 33.5(8) | 30.7(8) | 5.1(7) | -7.3(6) | 0.2(7) |
| C12B | 44.9(9) | 26.1(7) | 22.4(7) | 1.4(6) | -4.5(6) | -3.1(7) |
| C13B | 41.3(8) | 20.1(7) | 18.5(6) | 2.4(5) | 1.8(6) | -0.8(6) |
| C14B | 45.5(9) | 23.7(7) | 28.7(8) | -5.2(6) | 4.8(7) | -0.7(7) |
| F9A | 44(4) | 25(2) | 122(10) | -2(6) | -47(4) | -12(3) |
| F7A | 135(9) | 22(2) | 60(6) | -17(5) | 53(6) | -6(6) |
| F8A | 74(6) | 11(2) | 33(3) | -1(2) | -17(3) | 1(3) |
| C32A | 20(4) | 49(4) | 32(4) | -4(3) | -1(3) | -4(3) |
| F24A | 24(3) | 67(4) | 21(3) | 5(3) | -2(2) | -13(2) |
| F23A | 72(7) | 90(5) | 55(6) | -17(4) | -39(4) | 46(5) |
| F22A | 39(3) | 96(5) | 30(2) | -10(3) | -5(2) | -39(3) |
| F12A | 124(7) | 34(3) | 17.2(19) | 4.2(15) | -17(4) | -26(5) |
| F11A | 31(2) | 56(3) | 45(5) | 35(3) | 10(2) | -0.3(17) |
| F10A | 30(2) | 38(3) | 46(4) | 16(2) | 4.9(18) | 19(2) |
| F8B | 42(7) | 47(10) | 100(20) | -43(15) | -13(9) | 12(7) |
| F7B | 51(10) | 28(8) | 36(7) | -14(7) | -16(7) | -9(8) |
| F9B | 99(19) | 40(9) | 35(7) | 0(6) | 16(9) | -30(12) |
| F16A | 65(4) | 65(4) | 19(2) | -8(2) | 2(2) | -28(3) |
| F18A | 41(3) | 86(7) | 88(7) | -69(5) | 34(3) | -40(3) |
| F17A | 88(6) | 29(3) | 75(5) | -20(3) | -26(4) | 20(3) |
| C32B | 26(9) | 56(11) | 18(9) | -10(9) | -2(6) | -4(8) |
| F23B | 23(8) | 91(15) | 20(9) | 5(9) | -9(5) | 8(10) |
| F22B | 110(20) | 64(10) | 90(20) | -21(9) | -64(15) | -11(9) |
| F24B | 40(9) | 140(20) | 14(8) | 18(13) | -9(6) | -9(14) |

Table 4 Bond Lengths for c020620\_1\_1.

| Atom | Atom | Length/Å |  | Atom | Atom | Length/Å |
| --- | --- | --- | --- | --- | --- | --- |
| F1 | C7 | 1.328(2) |  | C16 | F11A | 1.355(8) |
| F2 | C7 | 1.328(2) |  | C16 | F10A | 1.324(7) |
| F3 | C7 | 1.344(2) |  | C17 | C18 | 1.4001(17) |
| F4 | C8 | 1.3401(18) |  | C17 | C22 | 1.3985(17) |
| F5 | C8 | 1.3420(17) |  | C17 | B1 | 1.6406(18) |
| F6 | C8 | 1.339(2) |  | C18 | C19 | 1.3904(18) |
| F7 | C15 | 1.307(6) |  | C19 | C20 | 1.3814(19) |
| F8 | C15 | 1.350(5) |  | C19 | C23 | 1.4979(18) |
| F9 | C15 | 1.324(5) |  | C20 | C21 | 1.3880(19) |
| F10 | C16 | 1.353(5) |  | C21 | C22 | 1.3908(18) |
| F11 | C16 | 1.330(5) |  | C21 | C24 | 1.4949(18) |
| F12 | C16 | 1.322(6) |  | C24 | F16A | 1.332(7) |
| F13 | C23 | 1.3394(19) |  | C24 | F18A | 1.331(8) |
| F14 | C23 | 1.3452(17) |  | C24 | F17A | 1.307(8) |
| F15 | C23 | 1.3296(17) |  | C25 | C26 | 1.3993(18) |
| F16 | C24 | 1.337(5) |  | C25 | C30 | 1.4057(18) |
| F17 | C24 | 1.330(5) |  | C25 | B1 | 1.6432(18) |
| F18 | C24 | 1.319(5) |  | C26 | C27 | 1.3923(17) |
| F19 | C31 | 1.3373(18) |  | C27 | C28 | 1.384(2) |
| F20 | C31 | 1.3336(17) |  | C27 | C31 | 1.498(2) |
| F21 | C31 | 1.3249(16) |  | C28 | C29 | 1.387(2) |
| F22 | C32 | 1.335(7) |  | C29 | C30 | 1.3877(19) |
| F23 | C32 | 1.324(8) |  | C29 | C32 | 1.535(7) |
| F24 | C32 | 1.334(8) |  | C29 | C32A | 1.481(10) |
| C1 | C2 | 1.4027(18) |  | C29 | C32B | 1.495(15) |
| C1 | C6 | 1.4004(18) |  | N1B | C2B | 1.343(2) |
| C1 | B1 | 1.6358(18) |  | N1B | C6B | 1.3493(19) |
| C2 | C3 | 1.3867(19) |  | N2B | C9B | 1.3431(19) |
| C3 | C4 | 1.386(2) |  | N2B | C13B | 1.3467(18) |
| C3 | C7 | 1.493(2) |  | C1B | C2B | 1.498(2) |
| C4 | C5 | 1.382(2) |  | C2B | C3B | 1.379(2) |
| C5 | C6 | 1.3908(18) |  | C3B | C4B | 1.383(3) |
| C5 | C8 | 1.494(2) |  | C4B | C5B | 1.378(3) |
| C9 | C10 | 1.3992(17) |  | C5B | C6B | 1.379(2) |
| C9 | C14 | 1.4026(17) |  | C6B | C7B | 1.501(2) |
| C9 | B1 | 1.6415(18) |  | C7B | C8B | 1.541(2) |
| C10 | C11 | 1.3915(18) |  | C8B | C9B | 1.509(2) |
| C11 | C12 | 1.3850(19) |  | C9B | C10B | 1.384(2) |
| C11 | C15 | 1.4933(18) |  | C10B | C11B | 1.380(2) |
| C12 | C13 | 1.3853(18) |  | C11B | C12B | 1.378(2) |
| C13 | C14 | 1.3925(17) |  | C12B | C13B | 1.382(2) |
| C13 | C16 | 1.4943(18) |  | C13B | C14B | 1.495(2) |
| C15 | F9A | 1.278(8) |  | C32A | F24A | 1.337(10) |
| C15 | F7A | 1.349(7) |  | C32A | F23A | 1.329(9) |
| C15 | F8A | 1.342(7) |  | C32A | F22A | 1.325(9) |
| C15 | F8B | 1.329(10) |  | C32B | F23B | 1.316(14) |
| C15 | F7B | 1.326(10) |  | C32B | F22B | 1.319(14) |
| C15 | F9B | 1.315(10) |  | C32B | F24B | 1.321(14) |
| C16 | F12A | 1.297(8) |  |  |  |  |

Table 5 Bond Angles for c020620\_1\_1.

| Atom | Atom | Atom | Angle/˚ |  | Atom | Atom | Atom | Angle/˚ |
| --- | --- | --- | --- | --- | --- | --- | --- | --- |
| C2 | C1 | B1 | 122.04(11) |  | F15 | C23 | F14 | 106.51(12) |
| C6 | C1 | C2 | 115.57(12) |  | F15 | C23 | C19 | 113.39(12) |
| C6 | C1 | B1 | 122.17(11) |  | F16 | C24 | C21 | 112.9(3) |
| C3 | C2 | C1 | 122.20(13) |  | F17 | C24 | F16 | 105.9(5) |
| C2 | C3 | C7 | 120.59(14) |  | F17 | C24 | C21 | 112.0(5) |
| C4 | C3 | C2 | 120.98(13) |  | F18 | C24 | F16 | 105.9(4) |
| C4 | C3 | C7 | 118.40(14) |  | F18 | C24 | F17 | 106.3(5) |
| C5 | C4 | C3 | 118.10(13) |  | F18 | C24 | C21 | 113.3(3) |
| C4 | C5 | C6 | 120.85(13) |  | F16A | C24 | C21 | 111.9(5) |
| C4 | C5 | C8 | 119.79(13) |  | F18A | C24 | C21 | 113.4(7) |
| C6 | C5 | C8 | 119.35(13) |  | F18A | C24 | F16A | 103.8(7) |
| C5 | C6 | C1 | 122.29(13) |  | F17A | C24 | C21 | 114.8(6) |
| F1 | C7 | F3 | 105.64(16) |  | F17A | C24 | F16A | 106.6(6) |
| F1 | C7 | C3 | 113.49(14) |  | F17A | C24 | F18A | 105.4(7) |
| F2 | C7 | F1 | 106.76(17) |  | C26 | C25 | C30 | 115.33(11) |
| F2 | C7 | F3 | 105.97(16) |  | C26 | C25 | B1 | 123.35(11) |
| F2 | C7 | C3 | 112.96(15) |  | C30 | C25 | B1 | 121.16(11) |
| F3 | C7 | C3 | 111.44(16) |  | C27 | C26 | C25 | 122.54(12) |
| F4 | C8 | F5 | 106.53(12) |  | C26 | C27 | C31 | 118.63(12) |
| F4 | C8 | C5 | 112.51(13) |  | C28 | C27 | C26 | 120.87(13) |
| F5 | C8 | C5 | 112.04(13) |  | C28 | C27 | C31 | 120.49(12) |
| F6 | C8 | F4 | 106.51(13) |  | C27 | C28 | C29 | 117.84(12) |
| F6 | C8 | F5 | 106.28(13) |  | C28 | C29 | C30 | 121.11(13) |
| F6 | C8 | C5 | 112.52(12) |  | C28 | C29 | C32 | 123.8(3) |
| C10 | C9 | C14 | 115.35(11) |  | C28 | C29 | C32A | 114.9(5) |
| C10 | C9 | B1 | 123.37(11) |  | C28 | C29 | C32B | 113.6(9) |
| C14 | C9 | B1 | 121.26(11) |  | C30 | C29 | C32 | 114.9(3) |
| C11 | C10 | C9 | 122.24(12) |  | C30 | C29 | C32A | 123.9(5) |
| C10 | C11 | C15 | 119.69(12) |  | C30 | C29 | C32B | 125.3(9) |
| C12 | C11 | C10 | 121.30(12) |  | C29 | C30 | C25 | 122.29(13) |
| C12 | C11 | C15 | 118.99(12) |  | F19 | C31 | C27 | 112.23(12) |
| C11 | C12 | C13 | 117.67(12) |  | F20 | C31 | F19 | 105.41(13) |
| C12 | C13 | C14 | 120.90(12) |  | F20 | C31 | C27 | 112.31(11) |
| C12 | C13 | C16 | 120.05(12) |  | F21 | C31 | F19 | 106.50(12) |
| C14 | C13 | C16 | 119.01(12) |  | F21 | C31 | F20 | 106.26(12) |
| C13 | C14 | C9 | 122.49(12) |  | F21 | C31 | C27 | 113.55(12) |
| F7 | C15 | F8 | 105.2(5) |  | F22 | C32 | C29 | 110.4(5) |
| F7 | C15 | F9 | 106.9(4) |  | F23 | C32 | F22 | 107.1(6) |
| F7 | C15 | C11 | 115.6(4) |  | F23 | C32 | F24 | 107.2(7) |
| F8 | C15 | C11 | 110.0(4) |  | F23 | C32 | C29 | 116.0(6) |
| F9 | C15 | F8 | 105.0(4) |  | F24 | C32 | F22 | 105.1(6) |
| F9 | C15 | C11 | 113.3(4) |  | F24 | C32 | C29 | 110.3(7) |
| F9A | C15 | C11 | 115.8(6) |  | C1 | B1 | C9 | 108.44(10) |
| F9A | C15 | F7A | 107.0(6) |  | C1 | B1 | C17 | 108.69(10) |
| F9A | C15 | F8A | 107.1(7) |  | C1 | B1 | C25 | 111.16(10) |
| F7A | C15 | C11 | 110.6(5) |  | C9 | B1 | C25 | 110.22(10) |
| F8A | C15 | C11 | 111.9(5) |  | C17 | B1 | C9 | 109.83(10) |
| F8A | C15 | F7A | 103.5(5) |  | C17 | B1 | C25 | 108.48(10) |
| F8B | C15 | C11 | 111.7(9) |  | C2B | N1B | C6B | 123.30(14) |
| F7B | C15 | C11 | 112.7(8) |  | C9B | N2B | C13B | 119.95(14) |
| F7B | C15 | F8B | 105.4(9) |  | N1B | C2B | C1B | 118.06(15) |
| F9B | C15 | C11 | 113.7(7) |  | N1B | C2B | C3B | 118.98(15) |
| F9B | C15 | F8B | 106.6(10) |  | C3B | C2B | C1B | 122.96(16) |
| F9B | C15 | F7B | 106.0(9) |  | C2B | C3B | C4B | 119.40(17) |
| F10 | C16 | C13 | 111.6(4) |  | C5B | C4B | C3B | 119.97(16) |
| F11 | C16 | F10 | 104.7(4) |  | C4B | C5B | C6B | 119.75(16) |
| F11 | C16 | C13 | 113.6(3) |  | N1B | C6B | C5B | 118.60(16) |
| F12 | C16 | F10 | 106.0(5) |  | N1B | C6B | C7B | 117.56(14) |
| F12 | C16 | F11 | 106.7(5) |  | C5B | C6B | C7B | 123.83(14) |
| F12 | C16 | C13 | 113.5(5) |  | C6B | C7B | C8B | 114.32(13) |
| F12A | C16 | C13 | 116.1(6) |  | C9B | C8B | C7B | 114.81(13) |
| F12A | C16 | F11A | 106.0(7) |  | N2B | C9B | C8B | 117.61(14) |
| F12A | C16 | F10A | 108.7(7) |  | N2B | C9B | C10B | 121.42(14) |
| F11A | C16 | C13 | 109.5(7) |  | C10B | C9B | C8B | 120.97(14) |
| F10A | C16 | C13 | 111.8(5) |  | C11B | C10B | C9B | 118.78(15) |
| F10A | C16 | F11A | 103.9(7) |  | C12B | C11B | C10B | 119.57(15) |
| C18 | C17 | B1 | 121.58(11) |  | C11B | C12B | C13B | 119.43(15) |
| C22 | C17 | C18 | 115.71(11) |  | N2B | C13B | C12B | 120.83(14) |
| C22 | C17 | B1 | 122.39(11) |  | N2B | C13B | C14B | 116.60(14) |
| C19 | C18 | C17 | 122.14(12) |  | C12B | C13B | C14B | 122.57(14) |
| C18 | C19 | C23 | 118.02(12) |  | F24A | C32A | C29 | 111.8(9) |
| C20 | C19 | C18 | 121.24(12) |  | F23A | C32A | C29 | 108.9(7) |
| C20 | C19 | C23 | 120.71(12) |  | F23A | C32A | F24A | 105.7(9) |
| C19 | C20 | C21 | 117.66(12) |  | F22A | C32A | C29 | 114.9(7) |
| C20 | C21 | C22 | 121.09(12) |  | F22A | C32A | F24A | 107.8(8) |
| C20 | C21 | C24 | 120.03(12) |  | F22A | C32A | F23A | 107.3(8) |
| C22 | C21 | C24 | 118.78(12) |  | F23B | C32B | C29 | 111.6(18) |
| C21 | C22 | C17 | 122.15(12) |  | F23B | C32B | F22B | 109.6(14) |
| F13 | C23 | F14 | 105.17(12) |  | F23B | C32B | F24B | 107.3(13) |
| F13 | C23 | C19 | 112.19(12) |  | F22B | C32B | C29 | 110.0(18) |
| F14 | C23 | C19 | 112.34(11) |  | F22B | C32B | F24B | 108.8(14) |
| F15 | C23 | F13 | 106.68(12) |  | F24B | C32B | C29 | 109.4(17) |

Table 6 Torsion Angles for c020620\_1\_1.

| A | B | C | D | Angle/˚ |  | A | B | C | D | Angle/˚ |
| --- | --- | --- | --- | --- | --- | --- | --- | --- | --- | --- |
| C1 | C2 | C3 | C4 | -0.9(2) |  | C20 | C21 | C24 | F16A | -103.4(6) |
| C1 | C2 | C3 | C7 | -178.84(15) |  | C20 | C21 | C24 | F18A | 139.6(8) |
| C2 | C1 | C6 | C5 | 0.97(19) |  | C20 | C21 | C24 | F17A | 18.3(8) |
| C2 | C1 | B1 | C9 | -81.18(14) |  | C22 | C17 | C18 | C19 | -0.25(18) |
| C2 | C1 | B1 | C17 | 159.46(11) |  | C22 | C17 | B1 | C1 | -86.73(13) |
| C2 | C1 | B1 | C25 | 40.13(16) |  | C22 | C17 | B1 | C9 | 154.79(11) |
| C2 | C3 | C4 | C5 | 0.4(2) |  | C22 | C17 | B1 | C25 | 34.26(15) |
| C2 | C3 | C7 | F1 | -20.6(2) |  | C22 | C21 | C24 | F16 | -176.1(3) |
| C2 | C3 | C7 | F2 | -142.35(17) |  | C22 | C21 | C24 | F17 | -56.6(4) |
| C2 | C3 | C7 | F3 | 98.48(19) |  | C22 | C21 | C24 | F18 | 63.6(4) |
| C3 | C4 | C5 | C6 | 0.7(2) |  | C22 | C21 | C24 | F16A | 72.9(6) |
| C3 | C4 | C5 | C8 | 179.33(13) |  | C22 | C21 | C24 | F18A | -44.1(8) |
| C4 | C3 | C7 | F1 | 161.41(15) |  | C22 | C21 | C24 | F17A | -165.4(8) |
| C4 | C3 | C7 | F2 | 39.7(2) |  | C23 | C19 | C20 | C21 | -176.50(12) |
| C4 | C3 | C7 | F3 | -79.48(19) |  | C24 | C21 | C22 | C17 | -176.31(12) |
| C4 | C5 | C6 | C1 | -1.5(2) |  | C25 | C26 | C27 | C28 | -1.1(2) |
| C4 | C5 | C8 | F4 | -96.52(16) |  | C25 | C26 | C27 | C31 | 179.75(12) |
| C4 | C5 | C8 | F5 | 23.5(2) |  | C26 | C25 | C30 | C29 | -0.2(2) |
| C4 | C5 | C8 | F6 | 143.16(14) |  | C26 | C25 | B1 | C1 | -152.09(11) |
| C6 | C1 | C2 | C3 | 0.22(19) |  | C26 | C25 | B1 | C9 | -31.82(16) |
| C6 | C1 | B1 | C9 | 93.13(13) |  | C26 | C25 | B1 | C17 | 88.47(14) |
| C6 | C1 | B1 | C17 | -26.23(16) |  | C26 | C27 | C28 | C29 | 0.7(2) |
| C6 | C1 | B1 | C25 | -145.56(12) |  | C26 | C27 | C31 | F19 | 61.08(17) |
| C6 | C5 | C8 | F4 | 82.11(17) |  | C26 | C27 | C31 | F20 | -57.47(17) |
| C6 | C5 | C8 | F5 | -157.90(13) |  | C26 | C27 | C31 | F21 | -178.06(12) |
| C6 | C5 | C8 | F6 | -38.21(18) |  | C27 | C28 | C29 | C30 | -0.1(2) |
| C7 | C3 | C4 | C5 | 178.40(15) |  | C27 | C28 | C29 | C32 | -174.3(4) |
| C8 | C5 | C6 | C1 | 179.91(13) |  | C27 | C28 | C29 | C32A | 175.5(4) |
| C9 | C10 | C11 | C12 | -0.79(19) |  | C27 | C28 | C29 | C32B | 177.4(10) |
| C9 | C10 | C11 | C15 | 177.36(12) |  | C28 | C27 | C31 | F19 | -118.07(15) |
| C10 | C9 | C14 | C13 | 2.35(17) |  | C28 | C27 | C31 | F20 | 123.38(15) |
| C10 | C9 | B1 | C1 | 15.92(15) |  | C28 | C27 | C31 | F21 | 2.79(19) |
| C10 | C9 | B1 | C17 | 134.56(12) |  | C28 | C29 | C30 | C25 | -0.1(2) |
| C10 | C9 | B1 | C25 | -105.97(13) |  | C28 | C29 | C32 | F22 | -11.5(8) |
| C10 | C11 | C12 | C13 | 1.57(19) |  | C28 | C29 | C32 | F23 | 110.6(6) |
| C10 | C11 | C15 | F7 | 32.7(4) |  | C28 | C29 | C32 | F24 | -127.3(6) |
| C10 | C11 | C15 | F8 | -86.2(4) |  | C28 | C29 | C32A | F24A | -153.1(8) |
| C10 | C11 | C15 | F9 | 156.6(4) |  | C28 | C29 | C32A | F23A | 90.5(9) |
| C10 | C11 | C15 | F9A | -110.6(8) |  | C28 | C29 | C32A | F22A | -29.8(10) |
| C10 | C11 | C15 | F7A | 127.5(7) |  | C28 | C29 | C32B | F23B | 57(2) |
| C10 | C11 | C15 | F8A | 12.6(6) |  | C28 | C29 | C32B | F22B | -65(2) |
| C10 | C11 | C15 | F8B | 81.7(17) |  | C28 | C29 | C32B | F24B | 175.2(18) |
| C10 | C11 | C15 | F7B | -159.8(13) |  | C30 | C25 | C26 | C27 | 0.80(18) |
| C10 | C11 | C15 | F9B | -39.1(17) |  | C30 | C25 | B1 | C1 | 32.72(16) |
| C11 | C12 | C13 | C14 | -0.38(19) |  | C30 | C25 | B1 | C9 | 152.98(12) |
| C11 | C12 | C13 | C16 | -178.00(12) |  | C30 | C25 | B1 | C17 | -86.73(14) |
| C12 | C11 | C15 | F7 | -149.1(4) |  | C30 | C29 | C32 | F22 | 174.0(6) |
| C12 | C11 | C15 | F8 | 92.0(4) |  | C30 | C29 | C32 | F23 | -63.8(7) |
| C12 | C11 | C15 | F9 | -25.2(5) |  | C30 | C29 | C32 | F24 | 58.2(8) |
| C12 | C11 | C15 | F9A | 67.6(8) |  | C30 | C29 | C32A | F24A | 22.3(11) |
| C12 | C11 | C15 | F7A | -54.3(7) |  | C30 | C29 | C32A | F23A | -94.1(10) |
| C12 | C11 | C15 | F8A | -169.2(6) |  | C30 | C29 | C32A | F22A | 145.6(7) |
| C12 | C11 | C15 | F8B | -100.1(17) |  | C30 | C29 | C32B | F23B | -126.1(19) |
| C12 | C11 | C15 | F7B | 18.4(13) |  | C30 | C29 | C32B | F22B | 112.0(19) |
| C12 | C11 | C15 | F9B | 139.1(17) |  | C30 | C29 | C32B | F24B | -7(2) |
| C12 | C13 | C14 | C9 | -1.65(19) |  | C31 | C27 | C28 | C29 | 179.85(13) |
| C12 | C13 | C16 | F10 | -27.2(4) |  | C32 | C29 | C30 | C25 | 174.5(4) |
| C12 | C13 | C16 | F11 | -145.4(4) |  | B1 | C1 | C2 | C3 | 174.88(12) |
| C12 | C13 | C16 | F12 | 92.4(6) |  | B1 | C1 | C6 | C5 | -173.68(12) |
| C12 | C13 | C16 | F12A | -11.1(9) |  | B1 | C9 | C10 | C11 | 177.56(11) |
| C12 | C13 | C16 | F11A | 108.7(7) |  | B1 | C9 | C14 | C13 | -176.38(11) |
| C12 | C13 | C16 | F10A | -136.7(6) |  | B1 | C17 | C18 | C19 | -173.92(11) |
| C14 | C9 | C10 | C11 | -1.15(17) |  | B1 | C17 | C22 | C21 | 174.27(11) |
| C14 | C9 | B1 | C1 | -165.45(11) |  | B1 | C25 | C26 | C27 | -174.65(12) |
| C14 | C9 | B1 | C17 | -46.81(14) |  | B1 | C25 | C30 | C29 | 175.37(13) |
| C14 | C9 | B1 | C25 | 72.67(14) |  | N1B | C2B | C3B | C4B | -0.2(2) |
| C14 | C13 | C16 | F10 | 155.1(4) |  | N1B | C6B | C7B | C8B | -58.91(19) |
| C14 | C13 | C16 | F11 | 37.0(4) |  | N2B | C9B | C10B | C11B | 1.2(2) |
| C14 | C13 | C16 | F12 | -85.2(6) |  | C1B | C2B | C3B | C4B | -179.86(16) |
| C14 | C13 | C16 | F12A | 171.2(9) |  | C2B | N1B | C6B | C5B | -0.5(2) |
| C14 | C13 | C16 | F11A | -68.9(7) |  | C2B | N1B | C6B | C7B | 178.58(14) |
| C14 | C13 | C16 | F10A | 45.7(6) |  | C2B | C3B | C4B | C5B | -0.2(3) |
| C15 | C11 | C12 | C13 | -176.60(12) |  | C3B | C4B | C5B | C6B | 0.2(3) |
| C16 | C13 | C14 | C9 | 175.99(11) |  | C4B | C5B | C6B | N1B | 0.2(2) |
| C17 | C18 | C19 | C20 | -0.78(19) |  | C4B | C5B | C6B | C7B | -178.92(15) |
| C17 | C18 | C19 | C23 | 177.15(12) |  | C5B | C6B | C7B | C8B | 120.17(16) |
| C18 | C17 | C22 | C21 | 0.66(18) |  | C6B | N1B | C2B | C1B | -179.75(14) |
| C18 | C17 | B1 | C1 | 86.52(13) |  | C6B | N1B | C2B | C3B | 0.6(2) |
| C18 | C17 | B1 | C9 | -31.97(15) |  | C6B | C7B | C8B | C9B | 87.58(18) |
| C18 | C17 | B1 | C25 | -152.50(11) |  | C7B | C8B | C9B | N2B | -37.4(2) |
| C18 | C19 | C20 | C21 | 1.37(19) |  | C7B | C8B | C9B | C10B | 142.27(15) |
| C18 | C19 | C23 | F13 | -50.39(17) |  | C8B | C9B | C10B | C11B | -178.45(14) |
| C18 | C19 | C23 | F14 | 67.84(17) |  | C9B | N2B | C13B | C12B | 1.1(2) |
| C18 | C19 | C23 | F15 | -171.33(13) |  | C9B | N2B | C13B | C14B | -178.54(13) |
| C19 | C20 | C21 | C22 | -0.97(19) |  | C9B | C10B | C11B | C12B | 0.1(2) |
| C19 | C20 | C21 | C24 | 175.24(12) |  | C10B | C11B | C12B | C13B | -0.7(2) |
| C20 | C19 | C23 | F13 | 127.55(14) |  | C11B | C12B | C13B | N2B | 0.2(2) |
| C20 | C19 | C23 | F14 | -114.22(14) |  | C11B | C12B | C13B | C14B | 179.74(14) |
| C20 | C19 | C23 | F15 | 6.61(19) |  | C13B | N2B | C9B | C8B | 177.88(13) |
| C20 | C21 | C22 | C17 | -0.05(19) |  | C13B | N2B | C9B | C10B | -1.7(2) |
| C20 | C21 | C24 | F16 | 7.7(4) |  | C32A | C29 | C30 | C25 | -175.3(5) |
| C20 | C21 | C24 | F17 | 127.1(4) |  | C32B | C29 | C30 | C25 | -177.3(11) |
| C20 | C21 | C24 | F18 | -112.7(4) |  |  |  |  |  |  |

Table 7 Hydrogen Atom Coordinates (Å×104) and Isotropic Displacement Parameters (Å2×103) for c020620\_1\_1.

| Atom | *x* | *y* | *z* | U(eq) |
| --- | --- | --- | --- | --- |
| H2 | 6240.76 | 5983.2 | 1860.25 | 26 |
| H4 | 8046.69 | 6786.63 | 211.02 | 32 |
| H6 | 8981.63 | 5198.85 | 1308.22 | 23 |
| H10 | 7933.32 | 6159.92 | 2973.74 | 20 |
| H12 | 8727.57 | 5886.44 | 5361.77 | 23 |
| H14 | 7965.87 | 4441.36 | 3892.54 | 19 |
| H18 | 9487.21 | 4612.84 | 2778.02 | 21 |
| H20 | 9797.32 | 3025.33 | 1526.5 | 23 |
| H22 | 7000.11 | 3917.4 | 1471.35 | 21 |
| H26 | 6165.72 | 4551.77 | 3635.18 | 20 |
| H28 | 3371.76 | 4192.95 | 2444.51 | 31 |
| H30 | 5776.6 | 4906.81 | 1235.15 | 26 |
| H1B | 6637(11) | 2079(8) | 2630(10) | 33 |
| H1BA | 4918.9 | 2883.32 | 2792.25 | 60 |
| H1BB | 4593.77 | 3036.35 | 1862.76 | 60 |
| H1BC | 5802.25 | 3059.22 | 2177.13 | 60 |
| H3B | 3913.28 | 2072.77 | 1302.66 | 47 |
| H4B | 4189.75 | 1033.46 | 1021.11 | 51 |
| H5B | 5687.46 | 539.43 | 1566.42 | 45 |
| H7BA | 7315.2 | 571.41 | 2383.58 | 43 |
| H7BB | 7260.34 | 1027.28 | 3140.85 | 43 |
| H8BA | 8869.26 | 1025.7 | 2302.17 | 40 |
| H8BB | 8182.87 | 1475.93 | 1721.75 | 40 |
| H10B | 10215.13 | 1724.57 | 2798.9 | 38 |
| H11B | 10680.58 | 2557.05 | 3644.17 | 41 |
| H12B | 9365.43 | 3174.71 | 4148.38 | 38 |
| H14A | 7024.98 | 3381.34 | 3460.27 | 49 |
| H14B | 7500.52 | 3309.89 | 4373.75 | 49 |
| H14C | 6678.33 | 2814.6 | 4005.04 | 49 |

Table 8 Atomic Occupancy for c020620\_1\_1.

| Atom | *Occupancy* |  | Atom | *Occupancy* |  | Atom | *Occupancy* |
| --- | --- | --- | --- | --- | --- | --- | --- |
| F7 | 0.543(11) |  | F8 | 0.543(11) |  | F9 | 0.543(11) |
| F10 | 0.59(2) |  | F11 | 0.59(2) |  | F12 | 0.59(2) |
| F16 | 0.59(2) |  | F17 | 0.59(2) |  | F18 | 0.59(2) |
| F22 | 0.543(11) |  | F23 | 0.543(11) |  | F24 | 0.543(11) |
| C32 | 0.543(11) |  | F9A | 0.378(12) |  | F7A | 0.378(12) |
| F8A | 0.378(12) |  | C32A | 0.378(12) |  | F24A | 0.378(12) |
| F23A | 0.378(12) |  | F22A | 0.378(12) |  | F12A | 0.41(2) |
| F11A | 0.41(2) |  | F10A | 0.41(2) |  | F8B | 0.079(5) |
| F7B | 0.079(5) |  | F9B | 0.079(5) |  | F16A | 0.41(2) |
| F18A | 0.41(2) |  | F17A | 0.41(2) |  | C32B | 0.079(5) |
| F23B | 0.079(5) |  | F22B | 0.079(5) |  | F24B | 0.079(5) |

Experimental

Single crystals of C46H29BF24N2
[c020620\_1\_1]
were
[].
A suitable crystal was selected and
[]
on a
Bruker APEX-II Duo (Mo)
diffractometer. The crystal was kept at 100.0(1) K during data collection.
Using Olex2 [1], the structure was solved with the
SHELXT
[2] structure solution program using
Intrinsic Phasing
and refined with the
SHELXL
[3] refinement package using
Least Squares
minimisation.

1. Dolomanov, O.V., Bourhis, L.J., Gildea, R.J, Howard, J.A.K. & Puschmann, H.
   (2009), J. Appl. Cryst. 42, 339-341.
2. Sheldrick, G.M. (2015). Acta Cryst. A71, 3-8.
3. Sheldrick, G.M. (2015). Acta Cryst. C71, 3-8.

Crystal structure determination of
[c020620\_1\_1]

**Crystal Data**
for C46H29BF24N2 (*M*=1076.47 g/mol):
monoclinic, space group P21/c (no. 14),
*a* = 12.7494(14) Å, *b* = 21.745(2) Å, *c* = 16.4749(17) Å, *β* = 93.000(3)°,
*V*= 4561.1(8) Å3,
*Z* = 4,
*T* = 100.0(1) K,
μ(MoKα) = 0.160 mm-1,
*Dcalc* = 1.568 g/cm3,
85974 reflections measured (3.104° ≤ 2Θ ≤ 56.686°),
11357 unique (*R*int = 0.0370, Rsigma = 0.0210) which were used in all calculations.
The final *R*1 was 0.0388
(I > 2σ(I)) and *wR*2 was 0.0988 (all data).

Refinement model description

Number of restraints - 575,
number of constraints - unknown.

Details:

```
1. Fixed Uiso
```

This report has been created with Olex2, compiled on
2020.02.04 svn.rd84adfe8 for OlexSys. Please
let us know
if there are any errors or if you would like to have additional features.
