# Supplementary material for: London Dispersion versus Intramolecular Hydrogen Bond in Bis‐Pyridines: How Accurate Is DFT for Competing Noncovalent Interactions in the Condensed Phase?
Source: Chemistry. 2025 Oct 23;31(66):e02745. doi: 10.1002/chem.202502745 (PMC12648470; doi:10.1002/chem.202502745)

checkCIF/PLATON report

Structure factors have been supplied for datablock(s) c020620_1_1

THIS REPORT IS FOR GUIDANCE ONLY. IF USED AS PART OF A REVIEW PROCEDURE FOR PUBLICATION, IT SHOULD NOT REPLACE THE EXPERTISE OF AN EXPERIENCED CRYSTALLOGRAPHIC REFEREE.

No syntax errors found. CIF dictionary Interpreting this report

Datablock: c020620_1_1

```
Bond precision:  C-C = 0.0020 A          Wavelength=0.71073

Cell:           a=12.7494(14)    b=21.745(2)       c=16.4749(17)
                alpha=90         beta=93.000(3)    gamma=90
Temperature:    100 K

                Calculated                 Reported
Volume          4561.2(8)                  4561.1(8)
Space group     P 21/c                     P 1 21/c 1
Hall group      -P 2ybc                    -P 2ybc
Moiety formula  C32 H12 B F24, C14 H17 N2  C32 H12 B F24, C14 H17 N2
Sum formula     C46 H29 B F24 N2           C46 H29 B F24 N2
Mr              1076.52                    1076.47
Dx,g cm-3       1.568                      1.568
Z               4                          4
Mu (mm-1)       0.160                      0.160
F000            2160.0                     2160.0
F000'           2162.03
h,k,lmax        17,29,22                   17,29,21
Nref            11376                      11357
Tmin,Tmax       0.964,0.987                0.713,0.746
Tmin'           0.964

Correction method= # Reported T Limits: Tmin=0.713 Tmax=0.746
AbsCorr = MULTI-SCAN

Data completeness= 0.998          Theta(max)= 28.343

R(reflections)= 0.0388( 8972)     wR2(reflections)= 0.0988( 11357)

S = 1.023                  Npar= 847
```

The following ALERTS were generated. Each ALERT has the format
test-name_ALERT_alert-type_alert-level.
Click on the hyperlinks for more details of the test.

Alert level C

```
PLAT213_ALERT_2_C Atom F2              has ADP max/min Ratio .....        3.1 prolat
PLAT213_ALERT_2_C Atom F9              has ADP max/min Ratio .....        3.1 prolat
PLAT213_ALERT_2_C Atom F22             has ADP max/min Ratio .....        3.7 prolat
PLAT213_ALERT_2_C Atom F24             has ADP max/min Ratio .....        3.1 prolat
PLAT213_ALERT_2_C Atom F7A             has ADP max/min Ratio .....        3.5 prolat
PLAT213_ALERT_2_C Atom F9A             has ADP max/min Ratio .....        3.6 prolat
PLAT213_ALERT_2_C Atom F12A            has ADP max/min Ratio .....        3.1 prolat
PLAT213_ALERT_2_C Atom F18A            has ADP max/min Ratio .....        3.4 prolat
PLAT213_ALERT_2_C Atom F24B            has ADP max/min Ratio .....        3.9 prolat
PLAT242_ALERT_2_C Low    'MainMol' Ueq as Compared to Neighbors of        C16 Check
PLAT242_ALERT_2_C Low    'MainMol' Ueq as Compared to Neighbors of        C24 Check
```

Alert level G

```
PLAT002_ALERT_2_G Number of Distance or Angle Restraints on AtSite         39 Note
PLAT003_ALERT_2_G Number of Uiso or Uij Restrained non-H Atoms ...         13 Report
PLAT172_ALERT_4_G The CIF-Embedded .res File Contains DFIX Records          1 Report
PLAT176_ALERT_4_G The CIF-Embedded .res File Contains SADI Records          9 Report
PLAT186_ALERT_4_G The CIF-Embedded .res File Contains ISOR Records          1 Report
PLAT187_ALERT_4_G The CIF-Embedded .res File Contains RIGU Records          4 Report
PLAT242_ALERT_2_G Low    'MainMol' Ueq as Compared to Neighbors of         C7 Check
PLAT242_ALERT_2_G Low    'MainMol' Ueq as Compared to Neighbors of         C8 Check
PLAT242_ALERT_2_G Low    'MainMol' Ueq as Compared to Neighbors of        C15 Check
PLAT242_ALERT_2_G Low    'MainMol' Ueq as Compared to Neighbors of        C23 Check
PLAT242_ALERT_2_G Low    'MainMol' Ueq as Compared to Neighbors of        C31 Check
PLAT301_ALERT_3_G Main Residue  Disorder ..............(Resd  1  )        23% Note
PLAT432_ALERT_2_G Short Inter X...Y Contact  F5       ..C20             2.96 Ang.
                                                2-x,1-y,-z  =      3_765 Check
PLAT432_ALERT_2_G Short Inter X...Y Contact  F7B      ..C12B            2.91 Ang.
                                               2-x,1-y,1-z  =      3_766 Check
PLAT720_ALERT_4_G Number of Unusual/Non-Standard Labels ..........          7 Note
PLAT860_ALERT_3_G Number of Least-Squares Restraints .............        575 Note
PLAT912_ALERT_4_G Missing # of FCF Reflections Above STh/L=  0.600         19 Note
PLAT978_ALERT_2_G Number C-C Bonds with Positive Residual Density.         14 Info
```

```
   0 ALERT level A = Most likely a serious problem - resolve or explain
   0 ALERT level B = A potentially serious problem, consider carefully
  11 ALERT level C = Check. Ensure it is not caused by an omission or oversight
  18 ALERT level G = General information/check it is not something unexpected

   0 ALERT type 1 CIF construction/syntax error, inconsistent or missing data
  21 ALERT type 2 Indicator that the structure model may be wrong or deficient
   2 ALERT type 3 Indicator that the structure quality may be low
   6 ALERT type 4 Improvement, methodology, query or suggestion
   0 ALERT type 5 Informative message, check
```

It is advisable to attempt to resolve as many as possible of the alerts in all categories. Often the minor alerts point to easily fixed oversights, errors and omissions in your CIF or refinement strategy, so attention to these fine details can be worthwhile. In order to resolve some of the more serious problems it may be necessary to carry out additional measurements or structure refinements. However, the purpose of your study may justify the reported deviations and the more serious of these should normally be commented upon in the discussion or experimental section of a paper or in the "special_details" fields of the CIF. checkCIF was carefully designed to identify outliers and unusual parameters, but every test has its limitations and alerts that are not important in a particular case may appear. Conversely, the absence of alerts does not guarantee there are no aspects of the results needing attention. It is up to the individual to critically assess their own results and, if necessary, seek expert advice.

Publication of your CIF in IUCr journals

A basic structural check has been run on your CIF. These basic checks will be run on all CIFs submitted for publication in IUCr journals (*Acta Crystallographica*, *Journal of Applied Crystallography*, *Journal of Synchrotron Radiation*); however, if you intend to submit to *Acta Crystallographica Section C* or *E* or *IUCrData*, you should make sure that full publication checks are run on the final version of your CIF prior to submission.

Publication of your CIF in other journals

Please refer to the *Notes for Authors* of the relevant journal for any special instructions relating to CIF submission.

PLATON version of 22/04/2020; check.def file version of 09/03/2020

Datablock c020620_1_1 - ellipsoid plot

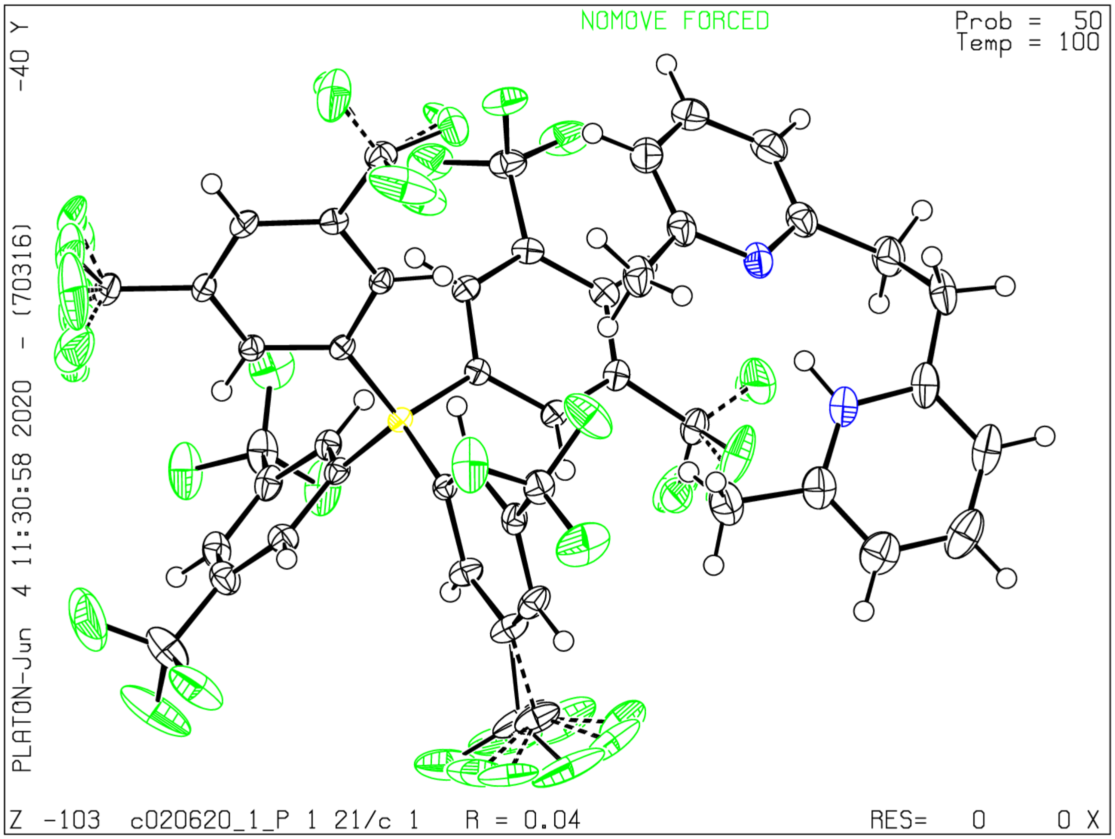

Supplement: Supplementary file 1 — Supporting Information [file CHEM-31-e02745-s002.zip › Crystal_structures/12b/checkcif.pdf]
