# Supplementary material for: London Dispersion versus Intramolecular Hydrogen Bond in Bis‐Pyridines: How Accurate Is DFT for Competing Noncovalent Interactions in the Condensed Phase?
Source: Chemistry. 2025 Oct 23;31(66):e02745. doi: 10.1002/chem.202502745 (PMC12648470; doi:10.1002/chem.202502745)
Supplement: Supplementary file 1 — Supporting Information [file CHEM-31-e02745-s002.zip › Crystal_structures/13b/c240322_3_1_tables.html]

c240322\_3\_1


# c240322\_3\_1

b"\n \n \n "

Table 1 Crystal data and structure refinement for c240322\_3\_1.

| Identification code | c240322\_3\_1 |
| Empirical formula | C53H43BCl2F24N2 |
| Formula weight | 1245.60 |
| Temperature/K | 100.0(1) |
| Crystal system | orthorhombic |
| Space group | Pbca |
| a/Å | 21.3063(2) |
| b/Å | 20.9139(2) |
| c/Å | 24.3031(3) |
| α/° | 90 |
| β/° | 90 |
| γ/° | 90 |
| Volume/Å3 | 10829.4(2) |
| Z | 8 |
| ρcalcg/cm3 | 1.528 |
| μ/mm‑1 | 2.188 |
| F(000) | 5040.0 |
| Crystal size/mm3 | 0.28 × 0.155 × 0.061 |
| Radiation | Cu Kα (λ = 1.54184) |
| 2Θ range for data collection/° | 7.274 to 159.532 |
| Index ranges | -27 ≤ h ≤ 22, -26 ≤ k ≤ 26, -30 ≤ l ≤ 30 |
| Reflections collected | 88264 |
| Independent reflections | 11655 [Rint = 0.0359, Rsigma = 0.0209] |
| Data/restraints/parameters | 11655/970/912 |
| Goodness-of-fit on F2 | 1.060 |
| Final R indexes [I>=2σ (I)] | R1 = 0.0537, wR2 = 0.1352 |
| Final R indexes [all data] | R1 = 0.0613, wR2 = 0.1416 |
| Largest diff. peak/hole / e Å-3 | 0.87/-0.69 |

Table 2 Fractional Atomic Coordinates (×104) and Equivalent Isotropic Displacement Parameters (Å2×103) for c240322\_3\_1. Ueq is defined as 1/3 of the trace of the orthogonalised UIJ tensor.

| Atom | *x* | *y* | *z* | U(eq) |
| --- | --- | --- | --- | --- |
| Cl1B | 5954.8(8) | 7324.7(11) | 3429.1(10) | 104.8(9) |
| Cl2B | 5044.4(12) | 6367.2(10) | 3679.9(12) | 122.3(9) |
| C1B | 5586(4) | 6908(4) | 3937(3) | 88(3) |
| Cl1C | 6083.9(15) | 7793.5(17) | 3946.4(19) | 120.4(16) |
| Cl2C | 5502(2) | 6740(2) | 3364.7(13) | 153(3) |
| C1C | 5670(6) | 7073(4) | 4018(3) | 46(2) |
| F1 | 8354.8(7) | 7599.0(8) | 4206.7(6) | 55.5(4) |
| F2 | 7651.0(7) | 7870.6(6) | 3638.6(6) | 45.8(3) |
| F3 | 8369.1(8) | 7222.6(7) | 3388.9(7) | 56.1(4) |
| F4 | 6208.8(19) | 5443(3) | 3237(2) | 96(2) |
| F5 | 7123(2) | 5413(2) | 2899.7(14) | 71.1(12) |
| F6 | 6874(3) | 4806.1(19) | 3542.1(18) | 65.1(15) |
| F7 | 8240.4(8) | 3901.6(11) | 5176.0(9) | 64.3(7) |
| F8 | 7472.3(10) | 3809.1(9) | 4655.3(7) | 51.3(5) |
| F9 | 7544.4(16) | 3216.2(8) | 5373.7(11) | 85.2(11) |
| F10A | 6896(4) | 4644(4) | 7269(2) | 97(3) |
| F11A | 5959(4) | 4752(4) | 6985(4) | 90(4) |
| F12A | 6414(5) | 3858(4) | 6954(5) | 60(3) |
| F13 | 6801(2) | 7183(2) | 7378.9(17) | 63.3(13) |
| F14 | 7555.2(17) | 7813(2) | 7429.3(13) | 71.4(10) |
| F15 | 6770(3) | 8049(2) | 6924(2) | 106(2) |
| F16 | 9338.8(7) | 7170.1(10) | 6425.8(7) | 71.1(6) |
| F17 | 9145.6(6) | 7448.4(8) | 5594.6(6) | 51.7(4) |
| F18 | 9251.4(6) | 6462.2(8) | 5807.1(8) | 58.9(4) |
| F19 | 4273.5(7) | 5672.6(7) | 4800.2(7) | 50.2(4) |
| F20 | 4576.4(6) | 5179.0(6) | 5529.5(6) | 38.9(3) |
| F21 | 3937.3(6) | 5968.0(7) | 5594.6(8) | 60.8(5) |
| F22 | 5637(4) | 8264(7) | 5811(3) | 54.6(16) |
| F23 | 5366.2(17) | 8254(2) | 4968(2) | 57.8(11) |
| F24 | 4672.6(17) | 8131(2) | 5586(2) | 58.4(12) |
| C1 | 7066.8(8) | 6239.9(8) | 4732.4(7) | 18.3(3) |
| C2 | 7431.7(8) | 6752.4(8) | 4540.7(7) | 20.0(3) |
| C3 | 7592.4(8) | 6818.0(8) | 3988.0(7) | 22.2(3) |
| C4 | 7988.4(9) | 7372.6(9) | 3807.5(8) | 29.3(4) |
| C5 | 7389.4(9) | 6381.1(9) | 3598.1(7) | 24.8(4) |
| C6 | 7009.9(9) | 5881.4(8) | 3774.0(7) | 23.2(4) |
| C7 | 6780.8(10) | 5395.7(10) | 3368.5(8) | 32.4(4) |
| C8 | 6849.4(8) | 5817.0(8) | 4326.2(7) | 20.6(3) |
| C9 | 6918.0(8) | 5405.4(8) | 5586.6(7) | 18.6(3) |
| C10 | 7231.5(8) | 4916.9(8) | 5305.6(7) | 19.9(3) |
| C11 | 7280.0(8) | 4296.9(8) | 5517.2(8) | 23.4(4) |
| C12 | 7611.8(10) | 3793.9(9) | 5189.7(9) | 33.0(4) |
| C13 | 7031.5(9) | 4143.9(9) | 6026.8(8) | 26.3(4) |
| C14 | 6728.6(9) | 4623.3(9) | 6320.4(8) | 26.7(4) |
| C15 | 6455.6(12) | 4484.5(11) | 6873.1(10) | 40.4(5) |
| C16 | 6672.5(8) | 5237.2(8) | 6102.2(7) | 22.6(3) |
| C17 | 7291.9(8) | 6572.5(8) | 5798.8(7) | 18.9(3) |
| C18 | 7044.3(9) | 6896.1(8) | 6253.0(7) | 22.4(3) |
| C19 | 7111.1(12) | 7596.1(11) | 7076.5(9) | 39.7(5) |
| C20 | 8057.9(10) | 7310.1(9) | 6523.7(8) | 29.8(4) |
| C21 | 8319.1(9) | 6973.2(9) | 6088.5(8) | 26.9(4) |
| C22 | 9008.5(10) | 7017.2(11) | 5976.7(10) | 38.0(5) |
| C23 | 7944.6(8) | 6608.6(8) | 5737.6(7) | 22.3(3) |
| C24 | 6122.1(8) | 6396.5(8) | 5400.0(7) | 18.0(3) |
| C25 | 5603.6(8) | 5985.3(8) | 5381.2(8) | 22.3(3) |
| C26 | 4988.7(8) | 6213.3(9) | 5375.2(8) | 24.0(4) |
| C27 | 4447.4(9) | 5760.6(10) | 5326.5(10) | 34.8(5) |
| C28 | 4864.3(8) | 6862.8(8) | 5407.4(7) | 22.5(3) |
| C29 | 5370.5(8) | 7279.5(8) | 5428.5(7) | 21.6(3) |
| C30 | 5253.7(9) | 7981.7(9) | 5470.7(10) | 33.7(4) |
| C31 | 5982.9(8) | 7051.5(8) | 5411.3(7) | 20.3(3) |
| C32 | 7416.0(10) | 7259.1(9) | 6606.9(8) | 27.1(4) |
| B1 | 6854.0(9) | 6150.5(9) | 5375.4(8) | 17.1(3) |
| N1D | 4120.6(8) | 9238.3(7) | 2980.3(6) | 24.4(3) |
| N2D | 4828.4(8) | 10294.3(8) | 3672.1(7) | 27.3(3) |
| C1D | 4206.4(9) | 8861.1(9) | 2533.7(8) | 26.7(4) |
| C2D | 3673.6(10) | 8657.9(9) | 2259.7(8) | 31.0(4) |
| C3D | 3085.6(10) | 8836.1(9) | 2443.6(9) | 31.2(4) |
| C4D | 3023.1(9) | 9224.2(9) | 2902.3(8) | 27.6(4) |
| C5D | 3553.1(9) | 9431.4(9) | 3175.1(8) | 24.8(4) |
| C6D | 4865.7(10) | 8709.5(10) | 2341.1(9) | 34.9(5) |
| C7D | 5362.8(11) | 8909.8(13) | 2754.9(11) | 47.0(6) |
| C8D | 4921.4(14) | 7984.9(13) | 2237.2(14) | 58.2(7) |
| C9D | 4962.6(15) | 9065.6(18) | 1796.6(13) | 64.8(8) |
| C10D | 3546.1(9) | 9881.4(10) | 3652.0(8) | 29.0(4) |
| C11D | 3739.8(9) | 10558.9(9) | 3476.1(9) | 30.8(4) |
| C12D | 4433.8(9) | 10641.5(9) | 3358.4(8) | 26.9(4) |
| C13D | 4642.6(10) | 11079.2(10) | 2974.6(9) | 34.1(4) |
| C14D | 5284.0(11) | 11183.0(10) | 2929.6(10) | 37.5(5) |
| C15D | 5688.8(10) | 10854.6(10) | 3269.7(9) | 33.5(4) |
| C16D | 5448.8(9) | 10405.7(9) | 3637.7(8) | 27.2(4) |
| C17D | 5879.5(10) | 10045.3(10) | 4037.1(9) | 33.3(4) |
| C18D | 5532.5(11) | 9502.5(11) | 4323.8(11) | 42.2(5) |
| C19D | 6452.6(11) | 9774.8(13) | 3732.9(12) | 46.7(6) |
| C20D | 6107.4(12) | 10523.7(12) | 4474.6(11) | 45.7(6) |
| F4A | 6181(3) | 5228(4) | 3467(3) | 73(3) |
| F6A | 7078(4) | 4867(4) | 3386(4) | 97(4) |
| F5A | 6753(3) | 5591(3) | 2860.6(19) | 68(2) |
| F7A | 7970(6) | 3464(6) | 5510(4) | 58(3) |
| F9A | 7151(4) | 3354(5) | 5067(5) | 59(3) |
| F8A | 7883(7) | 3938(7) | 4768(5) | 75(4) |
| F15A | 6499(3) | 7807(3) | 6956(3) | 63(2) |
| F13A | 7005(4) | 7280(3) | 7525(3) | 82(3) |
| F14A | 7363(3) | 8155(3) | 7226(3) | 68.8(16) |
| F10 | 6580(4) | 3937(3) | 7086(4) | 85(3) |
| F11 | 6577(2) | 4933.2(17) | 7242.8(14) | 66.7(11) |
| F12 | 5813(2) | 4510(3) | 6854(3) | 87(2) |
| F24A | 5078(4) | 8266(3) | 5027(3) | 71(2) |
| F23A | 5732(5) | 8310(10) | 5679(6) | 58(3) |
| F22A | 4783(3) | 8098(4) | 5853(3) | 60(2) |

Table 3 Anisotropic Displacement Parameters (Å2×103) for c240322\_3\_1. The Anisotropic displacement factor exponent takes the form: -2π2[h2a\*2U11+2hka\*b\*U12+…].

| Atom | U11 | U22 | U33 | U23 | U13 | U12 |
| --- | --- | --- | --- | --- | --- | --- |
| Cl1B | 59.2(9) | 123.0(17) | 132.3(18) | 38.8(15) | 18.6(10) | 35.9(10) |
| Cl2B | 129.0(17) | 84.0(12) | 154(2) | -11.5(12) | 56.2(16) | 21.8(11) |
| C1B | 93(5) | 131(6) | 39(3) | -26(4) | -23(3) | 87(4) |
| Cl1C | 89(2) | 103(2) | 169(4) | 54(2) | 27(2) | 32.4(17) |
| Cl2C | 185(4) | 202(4) | 72.6(18) | -75(2) | -72(2) | 143(4) |
| C1C | 67(5) | 59(4) | 12(3) | -1(3) | 1(3) | 29(4) |
| F1 | 57.4(9) | 61.0(9) | 48.0(8) | 18.9(7) | -15.4(7) | -40.2(8) |
| F2 | 49.8(8) | 26.4(6) | 61.1(9) | 18.4(6) | 1.6(6) | -2.8(5) |
| F3 | 63.2(9) | 39.3(8) | 65.8(10) | 0.4(7) | 40.9(8) | -12.2(7) |
| F4 | 55(2) | 111(4) | 121(5) | -79(3) | -56(3) | 30(3) |
| F5 | 108(3) | 71(2) | 34.6(16) | -27.2(15) | 23.3(19) | -44(2) |
| F6 | 137(4) | 22.5(14) | 36.2(14) | -3.4(12) | -16.6(19) | -19.5(19) |
| F7 | 36.3(9) | 77.1(14) | 79.5(14) | -32.6(11) | 7.9(9) | 16.4(9) |
| F8 | 64.6(12) | 51.3(10) | 37.9(8) | -19.4(7) | -6.9(8) | 24.6(9) |
| F9 | 151(3) | 17.1(8) | 87.3(16) | 10.4(9) | 71.3(18) | 18.4(11) |
| F10A | 137(6) | 125(7) | 30(2) | 15(4) | -2(3) | -65(5) |
| F11A | 123(7) | 67(4) | 82(6) | 50(4) | 78(5) | 72(5) |
| F12A | 90(5) | 29(2) | 63(7) | 19(3) | 52(4) | 3(3) |
| F13 | 75(2) | 68(2) | 47(2) | -20.8(17) | 30.5(19) | -8.8(18) |
| F14 | 81(2) | 87(3) | 45.8(17) | -42.0(18) | -8.5(15) | -11(2) |
| F15 | 198(6) | 78(3) | 42.4(18) | -9(2) | -9(3) | 105(4) |
| F16 | 39.6(8) | 118.8(16) | 55.0(10) | 18.5(10) | -22.6(7) | -35.9(9) |
| F17 | 39.0(7) | 56.2(9) | 60.0(9) | 30.0(7) | 2.0(6) | -13.5(6) |
| F18 | 24.0(6) | 51.4(9) | 101.2(13) | 20.8(9) | 3.3(7) | 0.9(6) |
| F19 | 44.3(8) | 36.8(7) | 69.4(10) | -3.3(7) | -29.4(7) | -9.1(6) |
| F20 | 29.1(6) | 24.3(6) | 63.1(9) | 1.8(5) | 0.7(6) | -9.1(5) |
| F21 | 23.1(6) | 39.7(8) | 119.5(15) | -16.5(8) | 21.3(8) | -5.8(5) |
| F22 | 54(3) | 31(3) | 78(3) | -27(2) | -17(2) | 6(3) |
| F23 | 58(2) | 30.6(16) | 85(2) | 28.5(14) | -9.6(17) | 0.6(16) |
| F24 | 30.8(13) | 23.4(13) | 121(4) | -8(2) | 6(2) | 9.7(9) |
| C1 | 17.3(7) | 15.1(7) | 22.4(8) | 1.5(6) | -0.4(6) | 1.1(6) |
| C2 | 21.1(8) | 16.2(8) | 22.8(8) | 0.2(6) | -1.5(6) | 0.0(6) |
| C3 | 22.3(8) | 19.4(8) | 25.0(8) | 3.7(7) | 0.1(7) | -0.5(6) |
| C4 | 32.3(10) | 26.8(9) | 28.8(9) | 5.9(7) | 1.6(8) | -3.9(8) |
| C5 | 28.7(9) | 25.3(9) | 20.2(8) | 2.8(7) | 1.2(7) | 2.3(7) |
| C6 | 27.0(9) | 20.9(8) | 21.7(8) | -0.8(7) | -3.2(7) | 0.3(7) |
| C7 | 42.2(11) | 32.1(10) | 22.8(9) | -3.8(8) | -3.8(8) | -4.4(8) |
| C8 | 21.1(8) | 18.9(8) | 21.7(8) | 0.6(6) | -0.9(6) | -1.6(6) |
| C9 | 16.6(7) | 17.3(8) | 22.0(8) | -0.4(6) | -1.6(6) | -2.1(6) |
| C10 | 19.9(8) | 17.9(8) | 22.1(8) | -0.8(6) | 0.2(6) | -1.1(6) |
| C11 | 23.0(8) | 18.8(8) | 28.5(9) | -2.0(7) | -0.8(7) | 0.1(6) |
| C12 | 40.7(11) | 22.8(9) | 35.5(10) | -0.7(8) | 1.4(8) | 7.4(8) |
| C13 | 29.0(9) | 17.2(8) | 32.7(10) | 4.5(7) | 0.7(8) | -0.1(7) |
| C14 | 29.3(9) | 23.7(9) | 27.0(9) | 5.2(7) | 3.9(7) | -0.7(7) |
| C15 | 54.3(13) | 30.3(10) | 36.6(11) | 10.0(9) | 14.2(10) | 2.8(9) |
| C16 | 23.8(8) | 19.9(8) | 24.1(9) | 0.8(7) | 3.4(7) | 1.4(6) |
| C17 | 21.4(8) | 15.2(7) | 20.1(8) | 3.1(6) | -1.4(6) | -0.9(6) |
| C18 | 26.8(9) | 18.9(8) | 21.3(8) | 0.9(6) | -2.2(7) | -0.1(7) |
| C19 | 57.7(14) | 34.2(11) | 27.1(10) | -8.5(8) | -9.1(9) | 1.9(10) |
| C20 | 39.8(11) | 20.6(9) | 29.0(9) | 4.8(7) | -14.6(8) | -10.7(8) |
| C21 | 26.4(9) | 25.1(9) | 29.2(9) | 9.3(7) | -8.0(7) | -8.1(7) |
| C22 | 29.3(10) | 44.0(12) | 40.6(12) | 16.3(10) | -11.5(9) | -16.1(9) |
| C23 | 22.1(8) | 20.9(8) | 23.8(8) | 4.4(7) | -3.0(7) | -2.4(6) |
| C24 | 20.2(8) | 17.5(8) | 16.3(7) | -1.3(6) | -0.1(6) | -1.2(6) |
| C25 | 21.1(8) | 16.8(8) | 28.8(9) | -2.6(7) | 0.3(7) | -1.0(6) |
| C26 | 21.2(8) | 21.1(8) | 29.5(9) | -3.7(7) | -0.2(7) | -2.0(7) |
| C27 | 21.8(9) | 24.8(9) | 57.9(14) | -4.6(9) | -0.2(9) | -0.5(7) |
| C28 | 20.1(8) | 21.7(8) | 25.7(9) | -1.8(7) | -0.1(7) | 2.6(6) |
| C29 | 22.8(8) | 17.8(8) | 24.3(8) | -0.7(6) | -0.4(7) | 1.4(6) |
| C30 | 26.3(9) | 20.6(9) | 54.3(13) | -1.3(8) | -5.1(9) | 1.7(7) |
| C31 | 20.9(8) | 17.3(8) | 22.6(8) | 0.2(6) | -1.2(6) | -1.7(6) |
| C32 | 40.1(11) | 19.5(8) | 21.8(8) | -0.2(7) | -6.9(8) | -0.8(7) |
| B1 | 18.1(8) | 14.8(8) | 18.5(8) | -1.0(6) | 0.2(7) | -1.3(6) |
| N1D | 27.0(8) | 22.2(7) | 23.9(7) | -2.2(6) | 1.2(6) | -2.1(6) |
| N2D | 26.8(8) | 22.3(8) | 32.7(8) | -3.4(6) | -1.8(6) | -0.6(6) |
| C1D | 35.7(10) | 19.5(8) | 24.9(9) | -0.1(7) | 2.2(7) | -2.7(7) |
| C2D | 42.0(11) | 24.0(9) | 27.0(9) | -0.8(7) | -1.5(8) | -6.4(8) |
| C3D | 36.0(10) | 25.1(9) | 32.4(10) | 4.5(8) | -6.5(8) | -8.9(8) |
| C4D | 28.1(9) | 23.6(9) | 31.2(10) | 7.0(7) | -0.7(8) | -3.5(7) |
| C5D | 27.7(9) | 19.8(8) | 26.8(9) | 4.0(7) | 2.2(7) | -0.7(7) |
| C6D | 36.8(11) | 33.8(11) | 34.3(11) | -7.9(8) | 8.5(9) | 0.0(9) |
| C7D | 31.7(11) | 55.5(15) | 53.9(15) | -20.3(12) | 5.1(10) | 3.3(10) |
| C8D | 51.8(15) | 45.3(15) | 77(2) | -28.3(14) | 4.7(14) | 7.5(12) |
| C9D | 55.8(17) | 88(2) | 50.9(16) | 12.9(15) | 21.4(13) | -5.2(16) |
| C10D | 25.7(9) | 32.2(10) | 29.0(9) | -5.2(8) | 2.9(7) | 0.8(7) |
| C11D | 28.1(9) | 25.4(9) | 39.0(11) | -7.7(8) | -1.6(8) | 4.1(7) |
| C12D | 29.6(9) | 20.8(8) | 30.2(9) | -7.1(7) | -1.0(8) | 2.7(7) |
| C13D | 40.3(11) | 28.7(10) | 33.4(11) | -0.2(8) | 2.1(9) | 9.4(8) |
| C14D | 45.1(12) | 28.0(10) | 39.2(12) | 3.8(8) | 14.1(10) | 4.3(9) |
| C15D | 31.9(10) | 26.2(10) | 42.3(12) | -1.2(8) | 8.6(9) | -0.7(8) |
| C16D | 26.2(9) | 21.0(9) | 34.3(10) | -4.3(7) | 0.6(7) | -0.7(7) |
| C17D | 27.4(10) | 29.0(10) | 43.5(12) | 1.4(9) | -5.8(8) | -2.7(8) |
| C18D | 38.8(12) | 35.5(12) | 52.2(14) | 11.0(10) | -11.4(10) | -4.3(9) |
| C19D | 31.8(11) | 48.3(14) | 60.0(16) | 0.8(12) | -4.2(10) | 8.1(10) |
| C20D | 47.3(13) | 40.8(13) | 48.9(14) | -4.0(11) | -14.1(11) | -5.0(10) |
| F4A | 58(3) | 113(6) | 47(3) | -44(3) | 12(3) | -55(4) |
| F6A | 95(5) | 66(5) | 129(8) | -73(4) | -81(5) | 55(4) |
| F5A | 113(5) | 76(4) | 14.9(17) | 5(2) | -15(3) | -52(4) |
| F7A | 61(5) | 49(5) | 64(4) | -23(3) | -22(4) | 36(4) |
| F9A | 52(4) | 44(4) | 83(5) | -42(4) | -10(4) | 10(3) |
| F8A | 85(6) | 72(5) | 69(5) | 8(4) | 25(4) | 10(4) |
| F15A | 54(3) | 79(5) | 56(3) | -45(3) | -19(2) | 35(3) |
| F13A | 147(8) | 70(4) | 31(3) | 17(3) | 30(4) | 73(5) |
| F14A | 77(4) | 57(3) | 73(4) | -49(3) | 8(3) | -20(3) |
| F10 | 149(6) | 51(3) | 53(3) | 35(2) | 48(4) | 49(3) |
| F11 | 112(3) | 61(2) | 27.7(14) | 2.3(13) | 21.9(18) | -16.9(19) |
| F12 | 41.4(15) | 156(6) | 63(3) | 40(3) | 22.3(15) | -6(2) |
| F24A | 127(7) | 24(2) | 61(3) | 3(2) | -39(4) | 18(4) |
| F23A | 31(3) | 22(3) | 122(9) | -27(6) | -13(4) | 1(2) |
| F22A | 61(4) | 29(2) | 91(5) | -6(3) | 39(4) | 13(3) |

Table 4 Bond Lengths for c240322\_3\_1.

| Atom | Atom | Length/Å |  | Atom | Atom | Length/Å |
| --- | --- | --- | --- | --- | --- | --- |
| Cl1B | C1B | 1.703(8) |  | C14 | C16 | 1.394(3) |
| Cl2B | C1B | 1.733(9) |  | C15 | F10 | 1.284(7) |
| Cl1C | C1C | 1.755(7) |  | C15 | F11 | 1.325(4) |
| Cl2C | C1C | 1.771(6) |  | C15 | F12 | 1.370(5) |
| F1 | C4 | 1.332(2) |  | C17 | C18 | 1.398(2) |
| F2 | C4 | 1.330(2) |  | C17 | C23 | 1.401(2) |
| F3 | C4 | 1.338(2) |  | C17 | B1 | 1.646(2) |
| F4 | C7 | 1.264(4) |  | C18 | C32 | 1.394(3) |
| F5 | C7 | 1.353(4) |  | C19 | C32 | 1.490(3) |
| F6 | C7 | 1.319(4) |  | C19 | F15A | 1.407(6) |
| F7 | C12 | 1.359(3) |  | C19 | F13A | 1.295(6) |
| F8 | C12 | 1.333(3) |  | C19 | F14A | 1.337(5) |
| F9 | C12 | 1.296(3) |  | C20 | C21 | 1.387(3) |
| F10A | C15 | 1.385(7) |  | C20 | C32 | 1.387(3) |
| F11A | C15 | 1.227(7) |  | C21 | C22 | 1.497(3) |
| F12A | C15 | 1.329(9) |  | C21 | C23 | 1.395(3) |
| F13 | C19 | 1.313(5) |  | C24 | C25 | 1.401(2) |
| F14 | C19 | 1.355(4) |  | C24 | C31 | 1.402(2) |
| F15 | C19 | 1.250(4) |  | C24 | B1 | 1.643(2) |
| F16 | C22 | 1.338(3) |  | C25 | C26 | 1.394(2) |
| F17 | C22 | 1.327(2) |  | C26 | C27 | 1.497(3) |
| F18 | C22 | 1.336(3) |  | C26 | C28 | 1.386(2) |
| F19 | C27 | 1.344(3) |  | C28 | C29 | 1.388(2) |
| F20 | C27 | 1.341(2) |  | C29 | C30 | 1.493(3) |
| F21 | C27 | 1.339(2) |  | C29 | C31 | 1.390(2) |
| F22 | C30 | 1.303(6) |  | C30 | F24A | 1.287(6) |
| F23 | C30 | 1.369(5) |  | C30 | F23A | 1.331(10) |
| F24 | C30 | 1.308(4) |  | C30 | F22A | 1.388(6) |
| C1 | C2 | 1.404(2) |  | N1D | C1D | 1.354(2) |
| C1 | C8 | 1.404(2) |  | N1D | C5D | 1.360(2) |
| C1 | B1 | 1.638(2) |  | N2D | C12D | 1.347(3) |
| C2 | C3 | 1.393(2) |  | N2D | C16D | 1.345(3) |
| C3 | C4 | 1.500(2) |  | C1D | C2D | 1.383(3) |
| C3 | C5 | 1.386(3) |  | C1D | C6D | 1.514(3) |
| C5 | C6 | 1.389(3) |  | C2D | C3D | 1.381(3) |
| C6 | C7 | 1.497(3) |  | C3D | C4D | 1.385(3) |
| C6 | C8 | 1.392(2) |  | C4D | C5D | 1.379(3) |
| C7 | F4A | 1.348(6) |  | C5D | C10D | 1.493(3) |
| C7 | F6A | 1.275(6) |  | C6D | C7D | 1.520(3) |
| C7 | F5A | 1.302(5) |  | C6D | C8D | 1.541(3) |
| C9 | C10 | 1.399(2) |  | C6D | C9D | 1.532(4) |
| C9 | C16 | 1.403(2) |  | C10D | C11D | 1.537(3) |
| C9 | B1 | 1.646(2) |  | C11D | C12D | 1.516(3) |
| C10 | C11 | 1.399(2) |  | C12D | C13D | 1.381(3) |
| C11 | C12 | 1.497(3) |  | C13D | C14D | 1.388(3) |
| C11 | C13 | 1.384(3) |  | C14D | C15D | 1.378(3) |
| C12 | F7A | 1.291(8) |  | C15D | C16D | 1.394(3) |
| C12 | F9A | 1.378(8) |  | C16D | C17D | 1.534(3) |
| C12 | F8A | 1.215(9) |  | C17D | C18D | 1.523(3) |
| C13 | C14 | 1.390(3) |  | C17D | C19D | 1.535(3) |
| C14 | C15 | 1.492(3) |  | C17D | C20D | 1.539(3) |

Table 5 Bond Angles for c240322\_3\_1.

| Atom | Atom | Atom | Angle/˚ |  | Atom | Atom | Atom | Angle/˚ |
| --- | --- | --- | --- | --- | --- | --- | --- | --- |
| Cl1B | C1B | Cl2B | 112.3(5) |  | C32 | C20 | C21 | 117.89(17) |
| Cl1C | C1C | Cl2C | 110.5(5) |  | C20 | C21 | C22 | 120.06(18) |
| C2 | C1 | C8 | 115.50(15) |  | C20 | C21 | C23 | 120.95(18) |
| C2 | C1 | B1 | 123.86(15) |  | C23 | C21 | C22 | 118.95(19) |
| C8 | C1 | B1 | 120.50(14) |  | F16 | C22 | C21 | 112.5(2) |
| C3 | C2 | C1 | 122.10(16) |  | F17 | C22 | F16 | 107.02(18) |
| C2 | C3 | C4 | 119.77(16) |  | F17 | C22 | F18 | 106.8(2) |
| C5 | C3 | C2 | 121.19(16) |  | F17 | C22 | C21 | 112.64(18) |
| C5 | C3 | C4 | 119.04(16) |  | F18 | C22 | F16 | 104.8(2) |
| F1 | C4 | F3 | 106.37(18) |  | F18 | C22 | C21 | 112.51(17) |
| F1 | C4 | C3 | 113.05(16) |  | C21 | C23 | C17 | 122.17(17) |
| F2 | C4 | F1 | 105.25(17) |  | C25 | C24 | C31 | 115.70(15) |
| F2 | C4 | F3 | 106.05(17) |  | C25 | C24 | B1 | 123.72(15) |
| F2 | C4 | C3 | 113.05(16) |  | C31 | C24 | B1 | 120.49(14) |
| F3 | C4 | C3 | 112.46(16) |  | C26 | C25 | C24 | 122.11(16) |
| C3 | C5 | C6 | 117.86(16) |  | C25 | C26 | C27 | 120.57(16) |
| C5 | C6 | C7 | 119.85(17) |  | C28 | C26 | C25 | 120.95(16) |
| C5 | C6 | C8 | 120.86(16) |  | C28 | C26 | C27 | 118.48(16) |
| C8 | C6 | C7 | 119.28(16) |  | F19 | C27 | C26 | 111.96(18) |
| F4 | C7 | F5 | 107.7(3) |  | F20 | C27 | F19 | 106.40(17) |
| F4 | C7 | F6 | 107.5(4) |  | F20 | C27 | C26 | 112.75(17) |
| F4 | C7 | C6 | 115.3(3) |  | F21 | C27 | F19 | 106.47(18) |
| F5 | C7 | C6 | 111.2(2) |  | F21 | C27 | F20 | 106.34(18) |
| F6 | C7 | F5 | 102.3(3) |  | F21 | C27 | C26 | 112.45(17) |
| F6 | C7 | C6 | 112.0(3) |  | C26 | C28 | C29 | 117.96(16) |
| F4A | C7 | C6 | 111.6(3) |  | C28 | C29 | C30 | 119.40(16) |
| F6A | C7 | C6 | 113.9(4) |  | C28 | C29 | C31 | 120.88(16) |
| F6A | C7 | F4A | 103.9(6) |  | C31 | C29 | C30 | 119.73(16) |
| F6A | C7 | F5A | 109.1(5) |  | F22 | C30 | F23 | 105.5(5) |
| F5A | C7 | C6 | 115.2(3) |  | F22 | C30 | F24 | 110.4(6) |
| F5A | C7 | F4A | 102.0(4) |  | F22 | C30 | C29 | 112.6(6) |
| C6 | C8 | C1 | 122.41(16) |  | F23 | C30 | C29 | 108.6(3) |
| C10 | C9 | C16 | 115.54(15) |  | F24 | C30 | F23 | 104.9(3) |
| C10 | C9 | B1 | 125.35(15) |  | F24 | C30 | C29 | 114.1(3) |
| C16 | C9 | B1 | 119.02(15) |  | F24A | C30 | C29 | 116.5(4) |
| C9 | C10 | C11 | 122.20(16) |  | F24A | C30 | F23A | 107.7(8) |
| C10 | C11 | C12 | 119.40(17) |  | F24A | C30 | F22A | 105.6(5) |
| C13 | C11 | C10 | 121.00(16) |  | F23A | C30 | C29 | 114.0(9) |
| C13 | C11 | C12 | 119.59(17) |  | F23A | C30 | F22A | 102.0(7) |
| F7 | C12 | C11 | 111.23(17) |  | F22A | C30 | C29 | 109.8(4) |
| F8 | C12 | F7 | 101.07(19) |  | C29 | C31 | C24 | 122.30(16) |
| F8 | C12 | C11 | 113.34(17) |  | C18 | C32 | C19 | 118.84(19) |
| F9 | C12 | F7 | 105.8(2) |  | C20 | C32 | C18 | 120.81(18) |
| F9 | C12 | F8 | 109.5(2) |  | C20 | C32 | C19 | 120.35(18) |
| F9 | C12 | C11 | 114.79(19) |  | C1 | B1 | C9 | 112.50(13) |
| F7A | C12 | C11 | 109.5(5) |  | C1 | B1 | C17 | 112.24(13) |
| F7A | C12 | F9A | 101.2(7) |  | C1 | B1 | C24 | 105.18(13) |
| F9A | C12 | C11 | 104.4(4) |  | C17 | B1 | C9 | 105.40(13) |
| F8A | C12 | C11 | 119.9(7) |  | C24 | B1 | C9 | 111.32(13) |
| F8A | C12 | F7A | 111.2(8) |  | C24 | B1 | C17 | 110.32(13) |
| F8A | C12 | F9A | 108.8(8) |  | C1D | N1D | C5D | 124.90(17) |
| C11 | C13 | C14 | 118.04(16) |  | C16D | N2D | C12D | 119.00(17) |
| C13 | C14 | C15 | 120.19(17) |  | N1D | C1D | C2D | 117.00(18) |
| C13 | C14 | C16 | 120.61(17) |  | N1D | C1D | C6D | 119.67(17) |
| C16 | C14 | C15 | 119.21(18) |  | C2D | C1D | C6D | 123.25(18) |
| F10A | C15 | C14 | 108.4(3) |  | C3D | C2D | C1D | 120.38(19) |
| F11A | C15 | F10A | 108.6(6) |  | C2D | C3D | C4D | 120.38(19) |
| F11A | C15 | F12A | 111.1(6) |  | C5D | C4D | C3D | 119.48(19) |
| F11A | C15 | C14 | 116.6(4) |  | N1D | C5D | C4D | 117.85(18) |
| F12A | C15 | F10A | 100.4(6) |  | N1D | C5D | C10D | 117.80(17) |
| F12A | C15 | C14 | 110.5(5) |  | C4D | C5D | C10D | 124.26(18) |
| F10 | C15 | C14 | 117.1(4) |  | C1D | C6D | C7D | 112.60(17) |
| F10 | C15 | F11 | 108.6(4) |  | C1D | C6D | C8D | 109.15(19) |
| F10 | C15 | F12 | 104.8(5) |  | C1D | C6D | C9D | 106.9(2) |
| F11 | C15 | C14 | 113.3(2) |  | C7D | C6D | C8D | 109.0(2) |
| F11 | C15 | F12 | 101.0(3) |  | C7D | C6D | C9D | 110.1(2) |
| F12 | C15 | C14 | 110.5(3) |  | C9D | C6D | C8D | 109.0(2) |
| C14 | C16 | C9 | 122.59(16) |  | C5D | C10D | C11D | 111.26(16) |
| C18 | C17 | C23 | 115.63(16) |  | C12D | C11D | C10D | 114.81(16) |
| C18 | C17 | B1 | 122.63(15) |  | N2D | C12D | C11D | 116.14(18) |
| C23 | C17 | B1 | 121.69(15) |  | N2D | C12D | C13D | 122.58(19) |
| C32 | C18 | C17 | 122.43(17) |  | C13D | C12D | C11D | 121.16(18) |
| F13 | C19 | F14 | 102.6(3) |  | C12D | C13D | C14D | 118.3(2) |
| F13 | C19 | C32 | 109.7(3) |  | C15D | C14D | C13D | 119.4(2) |
| F14 | C19 | C32 | 109.8(2) |  | C14D | C15D | C16D | 119.4(2) |
| F15 | C19 | F13 | 111.8(4) |  | N2D | C16D | C15D | 121.15(19) |
| F15 | C19 | F14 | 109.9(3) |  | N2D | C16D | C17D | 117.61(17) |
| F15 | C19 | C32 | 112.7(3) |  | C15D | C16D | C17D | 121.17(18) |
| F15A | C19 | C32 | 113.1(3) |  | C16D | C17D | C19D | 110.61(19) |
| F13A | C19 | C32 | 118.7(4) |  | C16D | C17D | C20D | 107.86(17) |
| F13A | C19 | F15A | 100.0(5) |  | C18D | C17D | C16D | 111.43(17) |
| F13A | C19 | F14A | 106.7(4) |  | C18D | C17D | C19D | 109.37(19) |
| F14A | C19 | C32 | 116.5(3) |  | C18D | C17D | C20D | 108.8(2) |
| F14A | C19 | F15A | 98.9(4) |  | C19D | C17D | C20D | 108.74(19) |

Table 6 Torsion Angles for c240322\_3\_1.

| A | B | C | D | Angle/˚ |  | A | B | C | D | Angle/˚ |
| --- | --- | --- | --- | --- | --- | --- | --- | --- | --- | --- |
| F13 | C19 | C32 | C18 | 56.0(3) |  | C23 | C17 | B1 | C1 | -42.5(2) |
| F13 | C19 | C32 | C20 | -124.4(3) |  | C23 | C17 | B1 | C9 | 80.33(18) |
| F14 | C19 | C32 | C18 | 168.0(3) |  | C23 | C17 | B1 | C24 | -159.39(15) |
| F14 | C19 | C32 | C20 | -12.4(3) |  | C23 | C21 | C22 | F16 | -157.22(19) |
| F15 | C19 | C32 | C18 | -69.3(4) |  | C23 | C21 | C22 | F17 | 81.7(3) |
| F15 | C19 | C32 | C20 | 110.4(4) |  | C23 | C21 | C22 | F18 | -39.1(3) |
| C1 | C2 | C3 | C4 | -179.46(16) |  | C24 | C25 | C26 | C27 | 176.93(18) |
| C1 | C2 | C3 | C5 | 1.0(3) |  | C24 | C25 | C26 | C28 | -2.3(3) |
| C2 | C1 | C8 | C6 | 3.1(2) |  | C25 | C24 | C31 | C29 | 2.9(3) |
| C2 | C1 | B1 | C9 | -137.66(16) |  | C25 | C24 | B1 | C1 | 98.01(18) |
| C2 | C1 | B1 | C17 | -19.0(2) |  | C25 | C24 | B1 | C9 | -24.1(2) |
| C2 | C1 | B1 | C24 | 101.00(17) |  | C25 | C24 | B1 | C17 | -140.75(16) |
| C2 | C3 | C4 | F1 | 26.0(3) |  | C25 | C26 | C27 | F19 | -92.3(2) |
| C2 | C3 | C4 | F2 | -93.4(2) |  | C25 | C26 | C27 | F20 | 27.7(3) |
| C2 | C3 | C4 | F3 | 146.53(18) |  | C25 | C26 | C27 | F21 | 147.9(2) |
| C2 | C3 | C5 | C6 | 1.2(3) |  | C25 | C26 | C28 | C29 | 2.0(3) |
| C3 | C5 | C6 | C7 | -179.76(17) |  | C26 | C28 | C29 | C30 | -179.22(18) |
| C3 | C5 | C6 | C8 | -1.2(3) |  | C26 | C28 | C29 | C31 | 0.7(3) |
| C4 | C3 | C5 | C6 | -178.37(17) |  | C27 | C26 | C28 | C29 | -177.28(18) |
| C5 | C3 | C4 | F1 | -154.36(18) |  | C28 | C26 | C27 | F19 | 87.0(2) |
| C5 | C3 | C4 | F2 | 86.2(2) |  | C28 | C26 | C27 | F20 | -153.09(18) |
| C5 | C3 | C4 | F3 | -33.9(3) |  | C28 | C26 | C27 | F21 | -32.9(3) |
| C5 | C6 | C7 | F4 | -105.7(4) |  | C28 | C29 | C30 | F22 | 138.9(5) |
| C5 | C6 | C7 | F5 | 17.3(3) |  | C28 | C29 | C30 | F23 | -104.6(3) |
| C5 | C6 | C7 | F6 | 131.0(3) |  | C28 | C29 | C30 | F24 | 12.0(4) |
| C5 | C6 | C7 | F4A | -140.2(4) |  | C28 | C29 | C30 | F24A | -76.2(5) |
| C5 | C6 | C7 | F6A | 102.5(6) |  | C28 | C29 | C30 | F23A | 157.4(7) |
| C5 | C6 | C7 | F5A | -24.6(5) |  | C28 | C29 | C30 | F22A | 43.7(4) |
| C5 | C6 | C8 | C1 | -1.1(3) |  | C28 | C29 | C31 | C24 | -3.3(3) |
| C7 | C6 | C8 | C1 | 177.52(17) |  | C30 | C29 | C31 | C24 | 176.69(17) |
| C8 | C1 | C2 | C3 | -3.0(2) |  | C31 | C24 | C25 | C26 | -0.1(3) |
| C8 | C1 | B1 | C9 | 46.9(2) |  | C31 | C24 | B1 | C1 | -78.41(18) |
| C8 | C1 | B1 | C17 | 165.58(15) |  | C31 | C24 | B1 | C9 | 159.49(15) |
| C8 | C1 | B1 | C24 | -74.44(18) |  | C31 | C24 | B1 | C17 | 42.8(2) |
| C8 | C6 | C7 | F4 | 75.7(4) |  | C31 | C29 | C30 | F22 | -41.0(5) |
| C8 | C6 | C7 | F5 | -161.3(3) |  | C31 | C29 | C30 | F23 | 75.5(3) |
| C8 | C6 | C7 | F6 | -47.6(3) |  | C31 | C29 | C30 | F24 | -167.9(3) |
| C8 | C6 | C7 | F4A | 41.1(5) |  | C31 | C29 | C30 | F24A | 103.8(5) |
| C8 | C6 | C7 | F6A | -76.1(6) |  | C31 | C29 | C30 | F23A | -22.6(7) |
| C8 | C6 | C7 | F5A | 156.8(4) |  | C31 | C29 | C30 | F22A | -136.3(4) |
| C9 | C10 | C11 | C12 | 178.84(17) |  | C32 | C20 | C21 | C22 | 179.33(17) |
| C9 | C10 | C11 | C13 | -1.8(3) |  | C32 | C20 | C21 | C23 | 1.7(3) |
| C10 | C9 | C16 | C14 | -0.7(3) |  | B1 | C1 | C2 | C3 | -178.68(16) |
| C10 | C9 | B1 | C1 | 12.3(2) |  | B1 | C1 | C8 | C6 | 178.92(16) |
| C10 | C9 | B1 | C17 | -110.28(18) |  | B1 | C9 | C10 | C11 | 178.40(16) |
| C10 | C9 | B1 | C24 | 130.10(17) |  | B1 | C9 | C16 | C14 | -177.46(16) |
| C10 | C11 | C12 | F7 | 70.8(2) |  | B1 | C17 | C18 | C32 | -179.24(16) |
| C10 | C11 | C12 | F8 | -42.3(3) |  | B1 | C17 | C23 | C21 | 178.92(16) |
| C10 | C11 | C12 | F9 | -169.2(2) |  | B1 | C24 | C25 | C26 | -176.70(16) |
| C10 | C11 | C12 | F7A | 136.7(7) |  | B1 | C24 | C31 | C29 | 179.56(15) |
| C10 | C11 | C12 | F9A | -115.7(6) |  | N1D | C1D | C2D | C3D | -0.3(3) |
| C10 | C11 | C12 | F8A | 6.4(10) |  | N1D | C1D | C6D | C7D | 11.1(3) |
| C10 | C11 | C13 | C14 | 0.4(3) |  | N1D | C1D | C6D | C8D | 132.3(2) |
| C11 | C13 | C14 | C15 | -179.67(19) |  | N1D | C1D | C6D | C9D | -109.9(2) |
| C11 | C13 | C14 | C16 | 0.7(3) |  | N1D | C5D | C10D | C11D | 73.7(2) |
| C12 | C11 | C13 | C14 | 179.78(18) |  | N2D | C12D | C13D | C14D | -2.9(3) |
| C13 | C11 | C12 | F7 | -108.6(2) |  | N2D | C16D | C17D | C18D | 12.9(3) |
| C13 | C11 | C12 | F8 | 138.3(2) |  | N2D | C16D | C17D | C19D | 134.8(2) |
| C13 | C11 | C12 | F9 | 11.5(3) |  | N2D | C16D | C17D | C20D | -106.4(2) |
| C13 | C11 | C12 | F7A | -42.7(7) |  | C1D | N1D | C5D | C4D | 0.7(3) |
| C13 | C11 | C12 | F9A | 65.0(6) |  | C1D | N1D | C5D | C10D | -176.18(17) |
| C13 | C11 | C12 | F8A | -172.9(9) |  | C1D | C2D | C3D | C4D | 0.5(3) |
| C13 | C14 | C15 | F10A | 94.1(5) |  | C2D | C1D | C6D | C7D | -172.2(2) |
| C13 | C14 | C15 | F11A | -143.1(6) |  | C2D | C1D | C6D | C8D | -51.0(3) |
| C13 | C14 | C15 | F12A | -15.0(6) |  | C2D | C1D | C6D | C9D | 66.8(3) |
| C13 | C14 | C15 | F10 | 8.2(5) |  | C2D | C3D | C4D | C5D | -0.1(3) |
| C13 | C14 | C15 | F11 | 135.8(3) |  | C3D | C4D | C5D | N1D | -0.5(3) |
| C13 | C14 | C15 | F12 | -111.6(4) |  | C3D | C4D | C5D | C10D | 176.15(18) |
| C13 | C14 | C16 | C9 | -0.6(3) |  | C4D | C5D | C10D | C11D | -102.9(2) |
| C15 | C14 | C16 | C9 | 179.80(19) |  | C5D | N1D | C1D | C2D | -0.3(3) |
| C16 | C9 | C10 | C11 | 1.9(2) |  | C5D | N1D | C1D | C6D | 176.70(18) |
| C16 | C9 | B1 | C1 | -171.28(15) |  | C5D | C10D | C11D | C12D | -73.8(2) |
| C16 | C9 | B1 | C17 | 66.10(19) |  | C6D | C1D | C2D | C3D | -177.15(19) |
| C16 | C9 | B1 | C24 | -53.5(2) |  | C10D | C11D | C12D | N2D | -34.5(2) |
| C16 | C14 | C15 | F10A | -86.3(5) |  | C10D | C11D | C12D | C13D | 149.45(19) |
| C16 | C14 | C15 | F11A | 36.5(6) |  | C11D | C12D | C13D | C14D | 172.93(19) |
| C16 | C14 | C15 | F12A | 164.6(6) |  | C12D | N2D | C16D | C15D | -2.5(3) |
| C16 | C14 | C15 | F10 | -172.2(5) |  | C12D | N2D | C16D | C17D | 174.61(17) |
| C16 | C14 | C15 | F11 | -44.6(4) |  | C12D | C13D | C14D | C15D | -0.7(3) |
| C16 | C14 | C15 | F12 | 68.0(4) |  | C13D | C14D | C15D | C16D | 2.5(3) |
| C17 | C18 | C32 | C19 | 179.11(17) |  | C14D | C15D | C16D | N2D | -1.0(3) |
| C17 | C18 | C32 | C20 | -0.5(3) |  | C14D | C15D | C16D | C17D | -177.95(19) |
| C18 | C17 | C23 | C21 | -3.5(2) |  | C15D | C16D | C17D | C18D | -170.0(2) |
| C18 | C17 | B1 | C1 | 140.14(16) |  | C15D | C16D | C17D | C19D | -48.1(3) |
| C18 | C17 | B1 | C9 | -97.07(18) |  | C15D | C16D | C17D | C20D | 70.7(3) |
| C18 | C17 | B1 | C24 | 23.2(2) |  | C16D | N2D | C12D | C11D | -171.54(17) |
| C20 | C21 | C22 | F16 | 25.1(3) |  | C16D | N2D | C12D | C13D | 4.5(3) |
| C20 | C21 | C22 | F17 | -96.0(2) |  | F15A | C19 | C32 | C18 | -33.3(4) |
| C20 | C21 | C22 | F18 | 143.17(19) |  | F15A | C19 | C32 | C20 | 146.4(4) |
| C20 | C21 | C23 | C17 | 1.2(3) |  | F13A | C19 | C32 | C18 | 83.4(5) |
| C21 | C20 | C32 | C18 | -2.0(3) |  | F13A | C19 | C32 | C20 | -97.0(5) |
| C21 | C20 | C32 | C19 | 178.39(18) |  | F14A | C19 | C32 | C18 | -146.9(4) |
| C22 | C21 | C23 | C17 | -176.54(17) |  | F14A | C19 | C32 | C20 | 32.8(5) |
| C23 | C17 | C18 | C32 | 3.2(2) |  |  |  |  |  |  |

Table 7 Hydrogen Atom Coordinates (Å×104) and Isotropic Displacement Parameters (Å2×103) for c240322\_3\_1.

| Atom | *x* | *y* | *z* | U(eq) |
| --- | --- | --- | --- | --- |
| H1BA | 5369.87 | 7211.79 | 4185.18 | 105 |
| H1BB | 5903.92 | 6674.65 | 4156.51 | 105 |
| H1CA | 5272.53 | 7152.4 | 4218.66 | 55 |
| H1CB | 5922.4 | 6766.8 | 4235.91 | 55 |
| H2 | 7573.64 | 7064.42 | 4796.09 | 24 |
| H5 | 7506.14 | 6422.08 | 3222.44 | 30 |
| H8 | 6582.9 | 5474.29 | 4432.33 | 25 |
| H10 | 7417.78 | 5009.34 | 4959.5 | 24 |
| H13 | 7067.12 | 3723.48 | 6171.58 | 32 |
| H16 | 6459.53 | 5554.06 | 6311.03 | 27 |
| H18 | 6606.49 | 6867.55 | 6322.94 | 27 |
| H20 | 8310.84 | 7567.55 | 6757.24 | 36 |
| H23 | 8138.61 | 6377.01 | 5447.52 | 27 |
| H25 | 5673.14 | 5536.73 | 5372.23 | 27 |
| H28 | 4445.26 | 7017.86 | 5414.8 | 27 |
| H31 | 6318.96 | 7350.04 | 5407.08 | 24 |
| H1D | 4464(9) | 9383(11) | 3166(9) | 29 |
| H2D | 3712.17 | 8394.2 | 1943.18 | 37 |
| H3D | 2722.06 | 8691.8 | 2254.44 | 37 |
| H4D | 2618.34 | 9346.62 | 3027.9 | 33 |
| H7DA | 5342.41 | 9373.38 | 2810.66 | 71 |
| H7DB | 5779.03 | 8793.6 | 2615.32 | 71 |
| H7DC | 5287.71 | 8691.49 | 3105.57 | 71 |
| H8DA | 4832.61 | 7752.9 | 2578.81 | 87 |
| H8DB | 5347.62 | 7884.14 | 2112.98 | 87 |
| H8DC | 4619.28 | 7856.84 | 1953.81 | 87 |
| H9DA | 4630.97 | 8945.09 | 1537.16 | 97 |
| H9DB | 5372.48 | 8951.95 | 1641.96 | 97 |
| H9DC | 4946.38 | 9527.62 | 1862.53 | 97 |
| H10A | 3838.19 | 9724.93 | 3938.77 | 35 |
| H10B | 3119.2 | 9894.01 | 3812.48 | 35 |
| H11A | 3500.94 | 10675.9 | 3141.48 | 37 |
| H11B | 3618.4 | 10861.64 | 3770.59 | 37 |
| H13D | 4354.69 | 11303.73 | 2747.12 | 41 |
| H14D | 5442.54 | 11477.75 | 2666.63 | 45 |
| H15D | 6127.48 | 10933.52 | 3253.58 | 40 |
| H18A | 5170.13 | 9675.22 | 4522.81 | 63 |
| H18B | 5815.67 | 9290.55 | 4583.98 | 63 |
| H18C | 5388.38 | 9192.53 | 4049.03 | 63 |
| H19A | 6312.39 | 9476.56 | 3447.19 | 70 |
| H19B | 6723.65 | 9549.45 | 3994.62 | 70 |
| H19C | 6687.39 | 10125.84 | 3562.68 | 70 |
| H20A | 6337.57 | 10871.72 | 4295.14 | 68 |
| H20B | 6384.69 | 10304.74 | 4735.42 | 68 |
| H20C | 5745.27 | 10699.38 | 4671.94 | 68 |

Table 8 Atomic Occupancy for c240322\_3\_1.

| Atom | *Occupancy* |  | Atom | *Occupancy* |  | Atom | *Occupancy* |
| --- | --- | --- | --- | --- | --- | --- | --- |
| Cl1B | 0.617(3) |  | Cl2B | 0.617(3) |  | C1B | 0.617(3) |
| H1BA | 0.617(3) |  | H1BB | 0.617(3) |  | Cl1C | 0.383(3) |
| Cl2C | 0.383(3) |  | C1C | 0.383(3) |  | H1CA | 0.383(3) |
| H1CB | 0.383(3) |  | F4 | 0.617(3) |  | F5 | 0.617(3) |
| F6 | 0.617(3) |  | F7 | 0.859(4) |  | F8 | 0.859(4) |
| F9 | 0.859(4) |  | F10A | 0.383(3) |  | F11A | 0.383(3) |
| F12A | 0.383(3) |  | F13 | 0.617(3) |  | F14 | 0.617(3) |
| F15 | 0.617(3) |  | F22 | 0.617(3) |  | F23 | 0.617(3) |
| F24 | 0.617(3) |  | F4A | 0.383(3) |  | F6A | 0.383(3) |
| F5A | 0.383(3) |  | F7A | 0.141(4) |  | F9A | 0.141(4) |
| F8A | 0.141(4) |  | F15A | 0.383(3) |  | F13A | 0.383(3) |
| F14A | 0.383(3) |  | F10 | 0.617(3) |  | F11 | 0.617(3) |
| F12 | 0.617(3) |  | F24A | 0.383(3) |  | F23A | 0.383(3) |
| F22A | 0.383(3) |  |  |  |  |  |

Experimental

Single crystals of C53H43BCl2F24N2
[c240322\_3\_1]
were
[].
A suitable crystal was selected and
[]
on a
XtaLAB Synergy, Dualflex, Pilatus 200K
diffractometer. The crystal was kept at 100.0(1) K during data collection.
Using Olex2 [1], the structure was solved with the
SHELXT
[2] structure solution program using
Intrinsic Phasing
and refined with the
SHELXL
[3] refinement package using
Least Squares
minimisation.

1. Dolomanov, O.V., Bourhis, L.J., Gildea, R.J, Howard, J.A.K. & Puschmann, H.
   (2009), J. Appl. Cryst. 42, 339-341.
2. Sheldrick, G.M. (2015). Acta Cryst. A71, 3-8.
3. Sheldrick, G.M. (2015). Acta Cryst. C71, 3-8.

Crystal structure determination of
[c240322\_3\_1]

**Crystal Data**
for C53H43BCl2F24N2 (*M*=1245.60 g/mol):
orthorhombic, space group Pbca (no. 61),
*a* = 21.3063(2) Å, *b* = 20.9139(2) Å, *c* = 24.3031(3) Å,
*V*= 10829.4(2) Å3,
*Z* = 8,
*T* = 100.0(1) K,
μ(Cu Kα) = 2.188 mm-1,
*Dcalc* = 1.528 g/cm3,
88264 reflections measured (7.274° ≤ 2Θ ≤ 159.532°),
11655 unique (*R*int = 0.0359, Rsigma = 0.0209) which were used in all calculations.
The final *R*1 was 0.0537
(I > 2σ(I)) and *wR*2 was 0.1416 (all data).

Refinement model description

Number of restraints - 970,
number of constraints - unknown.

Details:

```
1. Fixed Uiso
```

This report has been created with Olex2, compiled on
2022.04.07 svn.rca3783a0 for OlexSys. Please
let us know
if there are any errors or if you would like to have additional features.
