# Supplementary material for: London Dispersion versus Intramolecular Hydrogen Bond in Bis‐Pyridines: How Accurate Is DFT for Competing Noncovalent Interactions in the Condensed Phase?
Source: Chemistry. 2025 Oct 23;31(66):e02745. doi: 10.1002/chem.202502745 (PMC12648470; doi:10.1002/chem.202502745)

checkCIF/PLATON report

Structure factors have been supplied for datablock(s) c240322_3_1

THIS REPORT IS FOR GUIDANCE ONLY. IF USED AS PART OF A REVIEW PROCEDURE FOR PUBLICATION, IT SHOULD NOT REPLACE THE EXPERTISE OF AN EXPERIENCED CRYSTALLOGRAPHIC REFEREE.

No syntax errors found. CIF dictionary Interpreting this report

Datablock: c240322_3_1

Bond precision:	C-C = 0.0027 A		Wavelength=1.54184
Cell:	a=21.3063(2)	b=20.9139(2)	c=24.3031(3)
	alpha=90	beta=90	gamma=90
Temperature:	100 K		

	Calculated	Reported
Volume	10829.4(2)	10829.4(2)
Space group	P b c a	P b c a
Hall group	-P 2ac 2ab	-P 2ac 2ab
Moiety formula	C32 H12 B F24, C20 H29 N2, C H2 Cl2	C H2 Cl2, C32 H12 B F24, C20 H29 N2
Sum formula	C53 H43 B Cl2 F24 N2	C53 H43 B Cl2 F24 N2
Mr	1245.60	1245.60
Dx,g cm-3	1.528	1.528
Z	8	8
Mu (mm-1)	2.188	2.188
F000	5040.0	5040.0
F000’	5067.51	
h,k,lmax	27,26,31	27,26,30
Nref	11792	11655
Tmin,Tmax	0.699,0.875	0.583,1.000
Tmin’	0.516	

Correction method= # Reported T Limits: Tmin=0.583 Tmax=1.000
AbsCorr = GAUSSIAN

Data completeness= 0.988 Theta(max)= 79.766

R(reflections)= 0.0537(10070) wR2(reflections)= 0.1416(11655)

S = 1.060 Npar= 912

The following ALERTS were generated. Each ALERT has the format
test-name_ALERT_alert-type_alert-level.
Click on the hyperlinks for more details of the test.

Alert level C

```
PLAT042_ALERT_1_C Calc. and Reported MoietyFormula Strings  Differ     Please Check
PLAT213_ALERT_2_C Atom F4              has ADP max/min Ratio .....        3.4 prolat
PLAT213_ALERT_2_C Atom F9              has ADP max/min Ratio .....        3.7 prolat
PLAT213_ALERT_2_C Atom F10             has ADP max/min Ratio .....        3.4 prolat
PLAT213_ALERT_2_C Atom F15             has ADP max/min Ratio .....        3.9 prolat
PLAT213_ALERT_2_C Atom F4A             has ADP max/min Ratio .....        3.3 prolat
PLAT213_ALERT_2_C Atom F5A             has ADP max/min Ratio .....        3.5 prolat
PLAT213_ALERT_2_C Atom F6A             has ADP max/min Ratio .....        3.7 prolat
PLAT213_ALERT_2_C Atom F11A            has ADP max/min Ratio .....        3.7 prolat
PLAT213_ALERT_2_C Atom F12A            has ADP max/min Ratio .....        3.4 prolat
PLAT260_ALERT_2_C Large Average Ueq of Residue Including      Cl1B      0.105 Check
PLAT260_ALERT_2_C Large Average Ueq of Residue Including      Cl1C      0.107 Check
PLAT906_ALERT_3_C Large K Value in the Analysis of Variance ......      2.763 Check
PLAT921_ALERT_1_C R1  in the CIF and FCF Differ by ..............     -0.0013 Check
PLAT922_ALERT_1_C wR2 in the CIF and FCF Differ by ..............     -0.0032 Check
PLAT923_ALERT_1_C S    Values in the CIF and FCF Differ by .......     -0.025 Check
```

Alert level G

```
PLAT002_ALERT_2_G Number of Distance or Angle Restraints on AtSite         42 Note
PLAT003_ALERT_2_G Number of Uiso or Uij Restrained non-H Atoms ...         45 Report
PLAT083_ALERT_2_G SHELXL Second Parameter in WGHT  Unusually Large      10.81 Why ?
PLAT142_ALERT_4_G s.u. on b - Axis Small or Missing .............     0.00020 Ang.
PLAT172_ALERT_4_G The CIF-Embedded .res File Contains DFIX Records          5 Report
PLAT173_ALERT_4_G The CIF-Embedded .res File Contains DANG Records          1 Report
PLAT176_ALERT_4_G The CIF-Embedded .res File Contains SADI Records         11 Report
PLAT178_ALERT_4_G The CIF-Embedded .res File Contains SIMU Records          7 Report
PLAT186_ALERT_4_G The CIF-Embedded .res File Contains ISOR Records          2 Report
PLAT187_ALERT_4_G The CIF-Embedded .res File Contains RIGU Records          7 Report
PLAT242_ALERT_2_G Low    'MainMol' Ueq as Compared to Neighbors of         C4 Check
PLAT242_ALERT_2_G Low    'MainMol' Ueq as Compared to Neighbors of         C7 Check
PLAT242_ALERT_2_G Low    'MainMol' Ueq as Compared to Neighbors of        C12 Check
PLAT242_ALERT_2_G Low    'MainMol' Ueq as Compared to Neighbors of        C15 Check
PLAT242_ALERT_2_G Low    'MainMol' Ueq as Compared to Neighbors of        C19 Check
PLAT242_ALERT_2_G Low    'MainMol' Ueq as Compared to Neighbors of        C22 Check
PLAT242_ALERT_2_G Low    'MainMol' Ueq as Compared to Neighbors of        C27 Check
PLAT242_ALERT_2_G Low    'MainMol' Ueq as Compared to Neighbors of        C30 Check
PLAT301_ALERT_3_G Main Residue  Disorder .............(Resd  1  )         26% Note
PLAT302_ALERT_4_G Anion/Solvent/Minor-Residue Disorder (Resd  3  )        100% Note
PLAT302_ALERT_4_G Anion/Solvent/Minor-Residue Disorder (Resd  4  )        100% Note
PLAT304_ALERT_4_G Non-Integer Number of Atoms in ..... (Resd  3  )       3.09 Check
PLAT304_ALERT_4_G Non-Integer Number of Atoms in ..... (Resd  4  )       1.91 Check
PLAT434_ALERT_2_G Short Inter HL..HL Contact F1       ..F9A      .       2.83 Ang.
                                             3/2-x,1/2+y,z  =      8_765 Check
PLAT434_ALERT_2_G Short Inter HL..HL Contact F18      ..F24A     .       2.74 Ang.
                                           1/2+x,3/2-y,1-z  =      4_566 Check
PLAT720_ALERT_4_G Number of Unusual/Non-Standard Labels .........         13 Note
PLAT860_ALERT_3_G Number of Least-Squares Restraints ............        970 Note
PLAT910_ALERT_3_G Missing # of FCF Reflection(s) Below Theta(Min).          1 Note
PLAT912_ALERT_4_G Missing # of FCF Reflections Above STh/L=  0.600        129 Note
```

```
PLAT953_ALERT_1_G Reported (CIF) and Actual (FCF) Hmax Differ by .          1 Units
PLAT978_ALERT_2_G Number C-C Bonds with Positive Residual Density.          0 Info
```

```
   0 ALERT level A = Most likely a serious problem - resolve or explain
   0 ALERT level B = A potentially serious problem, consider carefully
  16 ALERT level C = Check. Ensure it is not caused by an omission or oversight
  31 ALERT level G = General information/check it is not something unexpected

   5 ALERT type 1 CIF construction/syntax error, inconsistent or missing data
  25 ALERT type 2 Indicator that the structure model may be wrong or deficient
   4 ALERT type 3 Indicator that the structure quality may be low
  13 ALERT type 4 Improvement, methodology, query or suggestion
   0 ALERT type 5 Informative message, check
```

It is advisable to attempt to resolve as many as possible of the alerts in all categories. Often the minor alerts point to easily fixed oversights, errors and omissions in your CIF or refinement strategy, so attention to these fine details can be worthwhile. In order to resolve some of the more serious problems it may be necessary to carry out additional measurements or structure refinements. However, the purpose of your study may justify the reported deviations and the more serious of these should normally be commented upon in the discussion or experimental section of a paper or in the "special_details" fields of the CIF. checkCIF was carefully designed to identify outliers and unusual parameters, but every test has its limitations and alerts that are not important in a particular case may appear. Conversely, the absence of alerts does not guarantee there are no aspects of the results needing attention. It is up to the individual to critically assess their own results and, if necessary, seek expert advice.

Publication of your CIF in IUCr journals

A basic structural check has been run on your CIF. These basic checks will be run on all CIFs submitted for publication in IUCr journals (*Acta Crystallographica*, *Journal of Applied Crystallography*, *Journal of Synchrotron Radiation*); however, if you intend to submit to *Acta Crystallographica Section C* or *E* or *IUCrData*, you should make sure that full publication checks are run on the final version of your CIF prior to submission.

Publication of your CIF in other journals

Please refer to the *Notes for Authors* of the relevant journal for any special instructions relating to CIF submission.

PLATON version of 12/09/2022; check.def file version of 09/08/2022

Datablock c240322_3_1 - ellipsoid plot

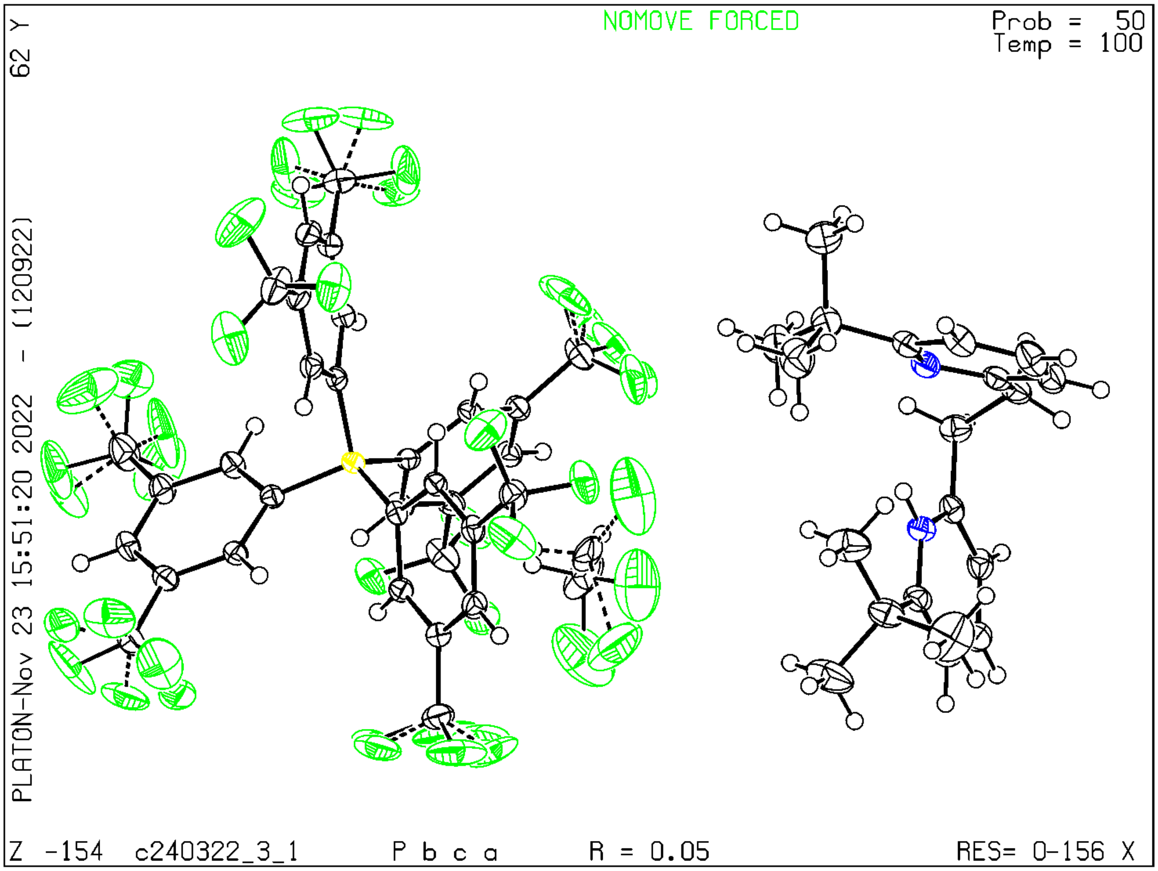

Supplement: Supplementary file 1 — Supporting Information [file CHEM-31-e02745-s002.zip › Crystal_structures/13b/checkcif.pdf]
