# Supplementary material for: London Dispersion versus Intramolecular Hydrogen Bond in Bis‐Pyridines: How Accurate Is DFT for Competing Noncovalent Interactions in the Condensed Phase?
Source: Chemistry. 2025 Oct 23;31(66):e02745. doi: 10.1002/chem.202502745 (PMC12648470; doi:10.1002/chem.202502745)
Supplement: Supplementary file 1 — Supporting Information [file CHEM-31-e02745-s002.zip › Crystal_structures/14b/c050620_2_1_tables.html]

c050620\_2\_1


# c050620\_2\_1

Table 1 Crystal data and structure refinement for c050620\_2\_1.

| Identification code | c050620\_2\_1 |
| Empirical formula | C45H27BF24N2 |
| Formula weight | 1062.49 |
| Temperature/K | 100.0(1) |
| Crystal system | monoclinic |
| Space group | P21/c |
| a/Å | 19.5780(15) |
| b/Å | 13.9630(10) |
| c/Å | 16.6113(13) |
| α/° | 90 |
| β/° | 106.838(2) |
| γ/° | 90 |
| Volume/Å3 | 4346.3(6) |
| Z | 4 |
| ρcalcg/cm3 | 1.624 |
| μ/mm‑1 | 0.167 |
| F(000) | 2128.0 |
| Crystal size/mm3 | 0.24 × 0.16 × 0.1 |
| Radiation | MoKα (λ = 0.71073) |
| 2Θ range for data collection/° | 3.638 to 56.744 |
| Index ranges | -26 ≤ h ≤ 26, -18 ≤ k ≤ 11, -22 ≤ l ≤ 22 |
| Reflections collected | 43270 |
| Independent reflections | 10838 [Rint = 0.0401, Rsigma = 0.0389] |
| Data/restraints/parameters | 10838/388/734 |
| Goodness-of-fit on F2 | 1.010 |
| Final R indexes [I>=2σ (I)] | R1 = 0.0450, wR2 = 0.0955 |
| Final R indexes [all data] | R1 = 0.0749, wR2 = 0.1082 |
| Largest diff. peak/hole / e Å-3 | 0.50/-0.29 |

Table 2 Fractional Atomic Coordinates (×104) and Equivalent Isotropic Displacement Parameters (Å2×103) for c050620\_2\_1. Ueq is defined as 1/3 of of the trace of the orthogonalised UIJ tensor.

| Atom | *x* | *y* | *z* | U(eq) |
| --- | --- | --- | --- | --- |
| F3 | 7703.2(7) | 3139.7(9) | 6137.3(7) | 36.5(3) |
| F23 | 6660.1(7) | 7028.1(9) | 5740.3(7) | 37.3(3) |
| F19 | 5272.6(6) | 8047.2(9) | 9012.9(7) | 36.6(3) |
| F8 | 10224.7(6) | 7839.4(9) | 8877.6(8) | 40.3(3) |
| F21 | 4603.9(6) | 7815.1(10) | 7754.4(7) | 41.9(3) |
| F1 | 6908.8(7) | 3098.6(9) | 6790.1(8) | 42.7(3) |
| F22 | 5850.5(7) | 8068.7(11) | 5671.8(7) | 48.6(4) |
| F15 | 7988.7(7) | 3659.2(9) | 12252.9(7) | 41.5(3) |
| F7 | 9351.8(7) | 8526.7(10) | 8021.2(8) | 46.1(3) |
| F2 | 6978.6(7) | 4312.7(9) | 6032.4(8) | 46.8(4) |
| F5A | 9957(8) | 3883(8) | 9481(5) | 42.6(19) |
| F14 | 8572.6(8) | 4947.9(9) | 12233.0(8) | 49.9(4) |
| F24 | 6953.4(8) | 8422.0(9) | 6223.7(8) | 46.1(3) |
| F20 | 4949.6(7) | 6624.1(10) | 8593.7(9) | 43.9(3) |
| F18A | 5838(6) | 3277(6) | 9038(5) | 47.4(18) |
| F12A | 8581(5) | 7957(6) | 11834(6) | 43.7(17) |
| F13 | 8805.4(7) | 3686.7(11) | 11633.5(9) | 51.0(4) |
| F9 | 9975.3(8) | 9275.0(9) | 9111.6(10) | 57.0(4) |
| F6A | 9908(5) | 3189(4) | 8323(5) | 36.1(13) |
| F11A | 9305(4) | 9059(7) | 11715(7) | 54.6(19) |
| F16A | 5670(5) | 3435(8) | 10240(4) | 60(2) |
| F17A | 5358(4) | 4535(5) | 9319(4) | 45.0(13) |
| N1B | 7320.4(9) | 3430.8(11) | 4195.9(10) | 26.7(3) |
| N2B | 5961.7(9) | 4030.8(12) | 3871.9(11) | 31.9(4) |
| F4A | 10224(4) | 4649(5) | 8492(6) | 51.1(15) |
| F10A | 8181(4) | 9227(6) | 11161(5) | 55.1(16) |
| C17 | 7470.0(9) | 5411.4(11) | 9653.5(10) | 15.3(3) |
| C1 | 8036.6(9) | 5340.2(11) | 8439.6(10) | 15.2(3) |
| C25 | 7015.2(8) | 6668.4(11) | 8361.7(10) | 15.3(3) |
| C10 | 8741.8(9) | 7315.0(11) | 8993.9(10) | 16.6(3) |
| C14 | 8295.0(9) | 7372.4(12) | 10179.2(10) | 16.8(3) |
| C6 | 8751.0(9) | 5050.2(12) | 8711.2(10) | 17.1(3) |
| C2 | 7609.8(9) | 4856.3(11) | 7738.1(10) | 16.6(3) |
| C13 | 8752.8(9) | 8126.3(12) | 10505.4(10) | 19.0(3) |
| C9 | 8279.2(8) | 6932.0(11) | 9415.0(10) | 14.9(3) |
| C29 | 6422.5(9) | 7427.8(12) | 7013.8(10) | 17.6(3) |
| C22 | 6801.3(9) | 4966.6(12) | 9438.6(10) | 17.3(3) |
| C30 | 7003.9(9) | 6966.0(11) | 7551.7(10) | 15.8(3) |
| C11 | 9197.2(9) | 8072.2(12) | 9317.9(11) | 17.1(3) |
| C3 | 7875.9(9) | 4147.0(12) | 7325.4(10) | 18.4(3) |
| C26 | 6407.7(9) | 6905.7(12) | 8605.5(10) | 18.6(3) |
| C18 | 7936.4(9) | 5140.5(12) | 10432.3(10) | 17.8(3) |
| C21 | 6598.8(9) | 4336.0(12) | 9973.2(11) | 19.4(3) |
| C20 | 7062.8(9) | 4093.5(12) | 10743.8(11) | 20.3(4) |
| C5 | 9016.7(9) | 4333.5(12) | 8306.6(11) | 19.0(3) |
| C19 | 7739.9(10) | 4491.6(12) | 10960.3(11) | 20.2(4) |
| C28 | 5809.3(9) | 7604.7(12) | 7253.0(11) | 20.1(3) |
| C12 | 9211.5(9) | 8487.2(12) | 10080.9(11) | 19.0(3) |
| C4 | 8585.0(9) | 3874.4(12) | 7602.5(11) | 20.2(4) |
| C27 | 5812.8(9) | 7340.9(13) | 8057.0(11) | 20.6(4) |
| C32 | 6465.3(10) | 7739.2(13) | 6168.4(11) | 23.2(4) |
| C15 | 9682.3(10) | 8428.9(13) | 8832.5(12) | 23.3(4) |
| C5B | 7737.8(10) | 4184.2(14) | 4165.6(11) | 26.9(4) |
| C24 | 5879.0(10) | 3885.1(14) | 9677.4(12) | 27.5(4) |
| C7 | 7377.3(10) | 3678.7(13) | 6578.7(12) | 26.4(4) |
| C8 | 9779.6(10) | 4043.4(14) | 8662.9(13) | 28.5(4) |
| C31 | 5162.4(10) | 7461.9(15) | 8349.5(12) | 28.6(4) |
| C2B | 8265.3(12) | 2341.6(15) | 4349.4(12) | 33.0(5) |
| C16 | 8732.4(11) | 8601.2(14) | 11304.2(12) | 30.2(4) |
| C23 | 8275.1(11) | 4203.0(14) | 11763.9(12) | 29.4(4) |
| C9B | 5795.5(11) | 4552.3(15) | 3159.0(13) | 31.8(4) |
| C3B | 8707.8(11) | 3094.1(15) | 4309.1(12) | 31.8(4) |
| C4B | 8444.0(11) | 4018.3(15) | 4220.9(13) | 32.1(4) |
| C1B | 7566.7(12) | 2529.5(14) | 4283.0(13) | 33.2(5) |
| C6B | 7387.8(11) | 5151.4(14) | 4046.5(14) | 32.8(5) |
| C8B | 6367.2(11) | 4648.0(17) | 2718.0(13) | 35.0(5) |
| C13B | 5477.6(12) | 3945.3(15) | 4300.1(15) | 37.2(5) |
| C7B | 6962.0(11) | 5359.4(15) | 3131.7(14) | 36.5(5) |
| B1 | 7702.4(10) | 6101.1(13) | 8977.3(11) | 14.9(4) |
| C12B | 4816.8(12) | 4364.9(16) | 4042.2(16) | 40.5(5) |
| C10B | 5135.3(11) | 4982.5(17) | 2861.2(15) | 41.6(5) |
| C11B | 4642.1(12) | 4892.3(18) | 3309.6(17) | 45.9(6) |
| F18 | 5811(6) | 3178(6) | 10215(5) | 44.8(19) |
| F16 | 5361(5) | 4460(6) | 9609(9) | 70(2) |
| F17 | 5783(6) | 3429(7) | 8951(5) | 42.3(19) |
| F10 | 8434(6) | 8115(7) | 11782(7) | 43(2) |
| F11 | 8432(6) | 9458(5) | 11165(6) | 63(2) |
| F12 | 9407(4) | 8786(8) | 11789(7) | 46.9(18) |
| F5 | 9899(9) | 3696(9) | 9455(6) | 34.8(18) |
| F4 | 10231(4) | 4827(5) | 8817(7) | 55.5(19) |
| F6 | 10021(6) | 3428(8) | 8242(7) | 53(2) |

Table 3 Anisotropic Displacement Parameters (Å2×103) for c050620\_2\_1. The Anisotropic displacement factor exponent takes the form: -2π2[h2a\*2U11+2hka\*b\*U12+…].

| Atom | U11 | U22 | U33 | U23 | U13 | U12 |
| --- | --- | --- | --- | --- | --- | --- |
| F3 | 45.7(7) | 37.5(6) | 26.4(6) | -16.1(5) | 10.3(5) | 2.8(6) |
| F23 | 53.5(8) | 41.7(7) | 22.2(6) | 0.8(5) | 19.5(5) | 11.6(6) |
| F19 | 37.1(7) | 50.3(7) | 24.9(6) | -4.8(5) | 12.9(5) | 16.2(6) |
| F8 | 28.9(6) | 51.0(8) | 49.3(8) | 11.7(6) | 24.6(6) | 7.9(6) |
| F21 | 20.3(6) | 76.7(9) | 28.7(6) | 1.4(6) | 7.2(5) | 18.5(6) |
| F1 | 39.9(7) | 45.1(7) | 45.2(7) | -22.2(6) | 15.4(6) | -21.7(6) |
| F22 | 36.8(7) | 85.3(10) | 24.9(6) | 23.4(7) | 11.0(5) | 30.5(7) |
| F15 | 50.8(8) | 45.2(7) | 22.2(6) | 14.3(5) | 0.6(5) | -13.7(6) |
| F7 | 37.5(7) | 70.0(9) | 30.1(7) | 23.5(6) | 8.8(5) | -9.7(7) |
| F2 | 55.5(8) | 35.5(7) | 30.3(7) | -9.5(5) | -17.6(6) | 12.0(6) |
| F5A | 28(3) | 64(5) | 25(2) | -11(2) | -9.2(17) | 23(3) |
| F14 | 63.8(9) | 40.9(7) | 27.9(7) | 5.3(6) | -13.8(6) | -22.1(7) |
| F24 | 64.1(9) | 44.0(7) | 33.3(7) | 6.9(6) | 18.9(6) | -20.6(7) |
| F20 | 35.9(7) | 49.1(8) | 57.9(8) | 3.6(7) | 31.4(6) | -0.9(6) |
| F18A | 38(3) | 42(2) | 58(4) | -26(2) | 8(3) | -12.2(18) |
| F12A | 68(4) | 47(2) | 24.1(19) | -7.3(16) | 26(2) | -1(2) |
| F13 | 39.8(8) | 62.7(9) | 44.8(8) | 20.3(7) | 3.3(6) | 20.0(7) |
| F9 | 78.5(10) | 36.2(7) | 77.3(10) | -24.0(7) | 55.8(9) | -37.5(7) |
| F6A | 26(3) | 39.2(19) | 40(2) | -5.8(16) | 5.9(16) | 17.3(16) |
| F11A | 60(3) | 68(4) | 44(3) | -40(3) | 29(3) | -42(3) |
| F16A | 36(3) | 115(5) | 28(2) | 24(3) | 7.8(15) | -37(3) |
| F17A | 16.7(16) | 51(2) | 61(3) | 11.9(18) | 1.5(16) | -7.7(13) |
| N1B | 33.3(9) | 26.2(8) | 23.2(8) | 3.0(7) | 12.3(7) | 3.3(7) |
| N2B | 36.8(10) | 30.5(9) | 31.0(9) | -4.4(7) | 13.9(8) | -2.5(8) |
| F4A | 18.6(15) | 50(2) | 85(4) | 13(2) | 16(2) | -2.3(15) |
| F10A | 69(3) | 52(3) | 46(2) | -22(2) | 19(2) | 24(2) |
| C17 | 18.0(8) | 14.4(7) | 14.9(8) | -3.3(6) | 6.8(6) | -0.1(6) |
| C1 | 16.9(8) | 15.2(7) | 14.2(8) | 1.8(6) | 5.9(6) | -1.6(6) |
| C25 | 15.2(8) | 13.5(7) | 16.1(8) | -3.1(6) | 3.1(6) | -2.8(6) |
| C10 | 18.2(8) | 16.3(7) | 14.7(8) | -0.4(6) | 3.7(6) | 0.4(6) |
| C14 | 15.9(8) | 18.7(8) | 16.6(8) | -0.4(7) | 5.8(6) | -0.4(6) |
| C6 | 16.5(8) | 18.7(8) | 15.5(8) | 1.4(6) | 3.8(6) | -2.3(7) |
| C2 | 15.1(8) | 16.4(7) | 18.1(8) | 0.6(6) | 4.4(6) | -0.5(6) |
| C13 | 19.4(8) | 19.6(8) | 16.9(8) | -3.5(7) | 3.7(7) | 1.7(7) |
| C9 | 13.3(7) | 14.5(7) | 15.3(8) | 0.8(6) | 1.5(6) | 1.6(6) |
| C29 | 19.0(8) | 17.7(8) | 15.4(8) | -3.0(7) | 4.1(6) | -0.9(7) |
| C22 | 17.6(8) | 19.2(8) | 15.4(8) | -1.7(6) | 5.1(6) | -1.4(7) |
| C30 | 14.1(8) | 15.8(7) | 17.9(8) | -1.9(6) | 5.4(6) | -1.4(6) |
| C11 | 15.9(8) | 15.7(7) | 19.3(8) | 1.7(7) | 4.6(6) | 0.7(6) |
| C3 | 23.7(9) | 15.4(8) | 16.5(8) | 0.1(7) | 6.3(7) | -1.0(7) |
| C26 | 20.5(8) | 20.9(8) | 14.7(8) | -2.4(7) | 5.5(7) | 0.3(7) |
| C18 | 16.9(8) | 16.9(8) | 19.1(8) | -1.1(7) | 4.5(7) | -0.9(6) |
| C21 | 20.2(8) | 20.4(8) | 20.3(8) | -4.5(7) | 10.0(7) | -4.0(7) |
| C20 | 27.9(9) | 17.6(8) | 18.1(8) | -0.9(7) | 10.7(7) | -4.4(7) |
| C5 | 16.8(8) | 21.3(8) | 20.2(8) | 5.1(7) | 7.3(7) | 2.4(7) |
| C19 | 26.0(9) | 17.4(8) | 16.7(8) | -1.3(7) | 5.3(7) | 0.1(7) |
| C28 | 17.5(8) | 22.6(8) | 18.8(8) | -2.3(7) | 2.8(7) | 3.4(7) |
| C12 | 17.3(8) | 15.1(8) | 22.9(9) | -3.1(7) | 3.2(7) | -2.4(6) |
| C4 | 25.2(9) | 18.3(8) | 19.6(8) | 1.7(7) | 10.5(7) | 3.2(7) |
| C27 | 18.4(8) | 24.2(8) | 20.2(9) | -4.7(7) | 7.0(7) | 1.8(7) |
| C32 | 22.0(9) | 26.5(9) | 20.7(9) | 4.6(7) | 5.5(7) | 5.3(7) |
| C15 | 23.0(9) | 21.2(8) | 26.6(9) | 0.0(7) | 8.6(7) | -5.5(7) |
| C5B | 31.9(10) | 26.3(9) | 18.5(9) | -0.5(7) | 0.8(8) | -1.1(8) |
| C24 | 24.6(9) | 33.4(10) | 27.0(10) | 0.7(8) | 11.5(8) | -8.1(8) |
| C7 | 30.2(10) | 23.1(9) | 23.9(9) | -6.2(8) | 4.9(8) | 2.3(8) |
| C8 | 21.5(9) | 32.5(10) | 31.9(10) | 1.0(8) | 8.4(8) | 5.0(8) |
| C31 | 22.9(9) | 38.5(11) | 24.9(9) | -1.5(8) | 7.9(8) | 8.2(8) |
| C2B | 48.3(13) | 27.4(10) | 27.6(10) | 3.3(8) | 18.0(9) | 10.3(9) |
| C16 | 34.2(11) | 30.1(10) | 27.2(10) | -9.8(8) | 10.5(8) | -5.5(8) |
| C23 | 34.7(11) | 26.3(9) | 22.7(9) | 6.1(8) | 1.2(8) | -6.8(8) |
| C9B | 29.1(10) | 34.3(11) | 30.0(11) | -5.9(9) | 5.5(8) | -5.1(9) |
| C3B | 31.5(11) | 36.8(11) | 24.8(10) | -0.3(9) | 4.4(8) | 5.9(9) |
| C4B | 30.4(11) | 30.2(10) | 29.1(10) | 0.4(8) | -1.9(8) | -2.2(9) |
| C1B | 48.2(13) | 24.2(9) | 35.1(11) | 3.2(9) | 24.3(10) | 1.5(9) |
| C6B | 30.5(11) | 23.2(9) | 39.6(12) | 1.0(9) | 2.2(9) | -0.8(8) |
| C8B | 31.5(11) | 46.4(13) | 24.3(10) | 6.9(9) | 3.5(8) | 2.0(10) |
| C13B | 45.1(13) | 31.7(11) | 40.8(12) | -11.1(9) | 22.1(10) | -7.1(10) |
| C7B | 30.0(11) | 34.1(11) | 44.2(13) | 16.2(10) | 9.0(9) | 3.9(9) |
| B1 | 14.8(9) | 15.4(8) | 14.0(8) | -1.7(7) | 3.4(7) | -1.0(7) |
| C12B | 36.6(12) | 35.8(11) | 54.5(15) | -19.8(11) | 21.7(11) | -14.7(10) |
| C10B | 27.5(11) | 49.2(13) | 41.1(13) | -3.1(11) | -1.0(9) | -6.3(10) |
| C11B | 21.6(11) | 47.6(14) | 65.0(17) | -18.0(13) | 6.8(11) | -7.6(10) |
| F18 | 46(4) | 50(3) | 40(2) | 2.3(18) | 14(2) | -31(2) |
| F16 | 28(2) | 42(3) | 145(7) | -15(4) | 35(4) | -3.1(18) |
| F17 | 35(3) | 74(4) | 17.7(19) | -4(2) | 7.5(18) | -37(3) |
| F10 | 46(3) | 56(4) | 38(3) | -29(3) | 29(3) | -26(3) |
| F11 | 99(5) | 39(3) | 51(2) | -17(2) | 22(4) | 24(3) |
| F12 | 42(2) | 63(4) | 32(2) | -30(3) | 4.4(17) | -14(2) |
| F5 | 28(3) | 41(3) | 32(3) | 8(2) | 3(3) | 8(2) |
| F4 | 17.6(17) | 50(3) | 93(5) | 18(3) | 6(3) | -2.7(18) |
| F6 | 35(3) | 88(5) | 40(3) | -11(4) | 16(2) | 28(4) |

Table 4 Bond Lengths for c050620\_2\_1.

| Atom | Atom | Length/Å |  | Atom | Atom | Length/Å |
| --- | --- | --- | --- | --- | --- | --- |
| F3 | C7 | 1.335(2) |  | C13 | C12 | 1.387(2) |
| F23 | C32 | 1.339(2) |  | C13 | C16 | 1.494(2) |
| F19 | C31 | 1.338(2) |  | C9 | B1 | 1.635(2) |
| F8 | C15 | 1.328(2) |  | C29 | C30 | 1.386(2) |
| F21 | C31 | 1.338(2) |  | C29 | C28 | 1.392(2) |
| F1 | C7 | 1.344(2) |  | C29 | C32 | 1.495(2) |
| F22 | C32 | 1.329(2) |  | C22 | C21 | 1.388(2) |
| F15 | C23 | 1.347(2) |  | C11 | C12 | 1.387(2) |
| F7 | C15 | 1.322(2) |  | C11 | C15 | 1.498(2) |
| F2 | C7 | 1.344(2) |  | C3 | C4 | 1.383(2) |
| F5A | C8 | 1.321(7) |  | C3 | C7 | 1.489(2) |
| F14 | C23 | 1.329(2) |  | C26 | C27 | 1.393(2) |
| F24 | C32 | 1.334(2) |  | C18 | C19 | 1.391(2) |
| F20 | C31 | 1.343(2) |  | C21 | C20 | 1.380(2) |
| F18A | C24 | 1.343(6) |  | C21 | C24 | 1.491(2) |
| F12A | C16 | 1.351(6) |  | C20 | C19 | 1.385(2) |
| F13 | C23 | 1.332(2) |  | C5 | C4 | 1.386(2) |
| F9 | C15 | 1.336(2) |  | C5 | C8 | 1.494(2) |
| F6A | C8 | 1.374(6) |  | C19 | C23 | 1.494(3) |
| F11A | C16 | 1.299(7) |  | C28 | C27 | 1.383(2) |
| F16A | C24 | 1.286(6) |  | C27 | C31 | 1.498(2) |
| F17A | C24 | 1.365(6) |  | C5B | C4B | 1.379(3) |
| N1B | C5B | 1.342(2) |  | C5B | C6B | 1.501(3) |
| N1B | C1B | 1.340(2) |  | C24 | F18 | 1.364(7) |
| N2B | C9B | 1.347(3) |  | C24 | F16 | 1.272(7) |
| N2B | C13B | 1.345(3) |  | C24 | F17 | 1.328(7) |
| F4A | C8 | 1.302(6) |  | C8 | F5 | 1.358(8) |
| F10A | C16 | 1.355(6) |  | C8 | F4 | 1.383(7) |
| C17 | C22 | 1.399(2) |  | C8 | F6 | 1.281(8) |
| C17 | C18 | 1.402(2) |  | C2B | C3B | 1.376(3) |
| C17 | B1 | 1.641(2) |  | C2B | C1B | 1.365(3) |
| C1 | C6 | 1.399(2) |  | C16 | F10 | 1.304(8) |
| C1 | C2 | 1.396(2) |  | C16 | F11 | 1.323(6) |
| C1 | B1 | 1.641(2) |  | C16 | F12 | 1.358(7) |
| C25 | C30 | 1.402(2) |  | C9B | C8B | 1.511(3) |
| C25 | C26 | 1.403(2) |  | C9B | C10B | 1.380(3) |
| C25 | B1 | 1.639(2) |  | C3B | C4B | 1.382(3) |
| C10 | C9 | 1.401(2) |  | C6B | C7B | 1.534(3) |
| C10 | C11 | 1.387(2) |  | C8B | C7B | 1.534(3) |
| C14 | C13 | 1.387(2) |  | C13B | C12B | 1.371(3) |
| C14 | C9 | 1.403(2) |  | C12B | C11B | 1.378(4) |
| C6 | C5 | 1.388(2) |  | C10B | C11B | 1.386(3) |
| C2 | C3 | 1.389(2) |  |  |  |  |

Table 5 Bond Angles for c050620\_2\_1.

| Atom | Atom | Atom | Angle/˚ |  | Atom | Atom | Atom | Angle/˚ |
| --- | --- | --- | --- | --- | --- | --- | --- | --- |
| C1B | N1B | C5B | 122.42(18) |  | F17A | C24 | C21 | 112.2(4) |
| C13B | N2B | C9B | 119.01(19) |  | F18 | C24 | C21 | 110.6(5) |
| C22 | C17 | C18 | 115.28(15) |  | F16 | C24 | C21 | 114.6(5) |
| C22 | C17 | B1 | 120.69(14) |  | F16 | C24 | F18 | 106.8(5) |
| C18 | C17 | B1 | 123.66(14) |  | F16 | C24 | F17 | 108.8(6) |
| C6 | C1 | B1 | 122.05(14) |  | F17 | C24 | C21 | 111.9(5) |
| C2 | C1 | C6 | 115.45(15) |  | F17 | C24 | F18 | 103.5(5) |
| C2 | C1 | B1 | 122.08(14) |  | F3 | C7 | F1 | 105.94(15) |
| C30 | C25 | C26 | 115.34(15) |  | F3 | C7 | F2 | 106.08(15) |
| C30 | C25 | B1 | 121.60(14) |  | F3 | C7 | C3 | 113.75(16) |
| C26 | C25 | B1 | 123.05(14) |  | F1 | C7 | C3 | 112.29(15) |
| C11 | C10 | C9 | 122.21(15) |  | F2 | C7 | F1 | 105.46(16) |
| C13 | C14 | C9 | 121.91(15) |  | F2 | C7 | C3 | 112.68(15) |
| C5 | C6 | C1 | 122.16(16) |  | F5A | C8 | F6A | 104.5(6) |
| C3 | C2 | C1 | 122.54(15) |  | F5A | C8 | C5 | 112.8(6) |
| C14 | C13 | C12 | 121.15(15) |  | F6A | C8 | C5 | 110.7(4) |
| C14 | C13 | C16 | 120.22(15) |  | F4A | C8 | F5A | 110.0(6) |
| C12 | C13 | C16 | 118.55(16) |  | F4A | C8 | F6A | 105.1(4) |
| C10 | C9 | C14 | 115.88(15) |  | F4A | C8 | C5 | 113.0(4) |
| C10 | C9 | B1 | 120.79(14) |  | F5 | C8 | C5 | 110.6(7) |
| C14 | C9 | B1 | 123.01(14) |  | F5 | C8 | F4 | 100.5(6) |
| C30 | C29 | C28 | 120.91(15) |  | F4 | C8 | C5 | 111.7(4) |
| C30 | C29 | C32 | 118.81(15) |  | F6 | C8 | C5 | 116.8(6) |
| C28 | C29 | C32 | 120.28(15) |  | F6 | C8 | F5 | 107.6(7) |
| C21 | C22 | C17 | 122.56(16) |  | F6 | C8 | F4 | 108.3(5) |
| C29 | C30 | C25 | 122.57(15) |  | F19 | C31 | F20 | 105.87(16) |
| C10 | C11 | C15 | 118.75(15) |  | F19 | C31 | C27 | 112.73(16) |
| C12 | C11 | C10 | 120.92(15) |  | F21 | C31 | F19 | 106.60(15) |
| C12 | C11 | C15 | 120.33(15) |  | F21 | C31 | F20 | 106.60(16) |
| C2 | C3 | C7 | 118.37(16) |  | F21 | C31 | C27 | 113.04(16) |
| C4 | C3 | C2 | 121.04(16) |  | F20 | C31 | C27 | 111.52(16) |
| C4 | C3 | C7 | 120.59(15) |  | C1B | C2B | C3B | 118.66(19) |
| C27 | C26 | C25 | 122.16(15) |  | F12A | C16 | F10A | 103.4(5) |
| C19 | C18 | C17 | 122.17(16) |  | F12A | C16 | C13 | 110.5(5) |
| C22 | C21 | C24 | 118.37(16) |  | F11A | C16 | F12A | 107.7(6) |
| C20 | C21 | C22 | 121.06(16) |  | F11A | C16 | F10A | 107.3(4) |
| C20 | C21 | C24 | 120.49(16) |  | F11A | C16 | C13 | 116.3(5) |
| C21 | C20 | C19 | 117.75(16) |  | F10A | C16 | C13 | 110.8(4) |
| C6 | C5 | C8 | 118.39(16) |  | F10 | C16 | C13 | 116.2(5) |
| C4 | C5 | C6 | 121.32(16) |  | F10 | C16 | F11 | 108.8(6) |
| C4 | C5 | C8 | 120.27(16) |  | F10 | C16 | F12 | 105.5(6) |
| C18 | C19 | C23 | 119.25(16) |  | F11 | C16 | C13 | 111.8(4) |
| C20 | C19 | C18 | 121.09(16) |  | F11 | C16 | F12 | 103.9(5) |
| C20 | C19 | C23 | 119.64(16) |  | F12 | C16 | C13 | 109.8(5) |
| C27 | C28 | C29 | 117.71(16) |  | F15 | C23 | C19 | 112.58(16) |
| C11 | C12 | C13 | 117.91(15) |  | F14 | C23 | F15 | 106.01(16) |
| C3 | C4 | C5 | 117.48(15) |  | F14 | C23 | F13 | 106.81(17) |
| C26 | C27 | C31 | 117.72(16) |  | F14 | C23 | C19 | 112.81(16) |
| C28 | C27 | C26 | 121.12(15) |  | F13 | C23 | F15 | 105.86(16) |
| C28 | C27 | C31 | 121.07(16) |  | F13 | C23 | C19 | 112.25(16) |
| F23 | C32 | C29 | 112.65(15) |  | N2B | C9B | C8B | 116.73(18) |
| F22 | C32 | F23 | 105.85(15) |  | N2B | C9B | C10B | 120.8(2) |
| F22 | C32 | F24 | 107.29(16) |  | C10B | C9B | C8B | 122.4(2) |
| F22 | C32 | C29 | 113.37(15) |  | C2B | C3B | C4B | 119.8(2) |
| F24 | C32 | F23 | 104.95(15) |  | C5B | C4B | C3B | 120.06(19) |
| F24 | C32 | C29 | 112.14(15) |  | N1B | C1B | C2B | 120.65(19) |
| F8 | C15 | F9 | 105.80(15) |  | C5B | C6B | C7B | 113.39(17) |
| F8 | C15 | C11 | 112.63(15) |  | C9B | C8B | C7B | 114.40(18) |
| F7 | C15 | F8 | 105.59(15) |  | N2B | C13B | C12B | 122.9(2) |
| F7 | C15 | F9 | 107.24(16) |  | C8B | C7B | C6B | 116.44(17) |
| F7 | C15 | C11 | 112.56(15) |  | C17 | B1 | C1 | 103.06(12) |
| F9 | C15 | C11 | 112.50(15) |  | C25 | B1 | C17 | 111.75(13) |
| N1B | C5B | C4B | 118.40(18) |  | C25 | B1 | C1 | 110.97(13) |
| N1B | C5B | C6B | 116.78(17) |  | C9 | B1 | C17 | 113.87(13) |
| C4B | C5B | C6B | 124.80(18) |  | C9 | B1 | C1 | 111.62(13) |
| F18A | C24 | F17A | 102.1(5) |  | C9 | B1 | C25 | 105.72(13) |
| F18A | C24 | C21 | 111.2(5) |  | C13B | C12B | C11B | 118.3(2) |
| F16A | C24 | F18A | 107.9(5) |  | C9B | C10B | C11B | 119.6(2) |
| F16A | C24 | F17A | 106.6(5) |  | C12B | C11B | C10B | 119.3(2) |
| F16A | C24 | C21 | 115.8(4) |  |  |  |  |  |

Table 6 Torsion Angles for c050620\_2\_1.

| A | B | C | D | Angle/˚ |  | A | B | C | D | Angle/˚ |
| --- | --- | --- | --- | --- | --- | --- | --- | --- | --- | --- |
| N1B | C5B | C4B | C3B | -0.3(3) |  | C26 | C25 | C30 | C29 | -2.5(2) |
| N1B | C5B | C6B | C7B | -79.1(2) |  | C26 | C25 | B1 | C17 | 28.0(2) |
| N2B | C9B | C8B | C7B | -76.6(2) |  | C26 | C25 | B1 | C1 | 142.38(15) |
| N2B | C9B | C10B | C11B | 1.3(3) |  | C26 | C25 | B1 | C9 | -96.44(17) |
| N2B | C13B | C12B | C11B | 0.4(3) |  | C26 | C27 | C31 | F19 | 64.1(2) |
| C17 | C22 | C21 | C20 | -1.9(3) |  | C26 | C27 | C31 | F21 | -174.93(16) |
| C17 | C22 | C21 | C24 | -178.82(16) |  | C26 | C27 | C31 | F20 | -54.8(2) |
| C17 | C18 | C19 | C20 | -1.2(3) |  | C18 | C17 | C22 | C21 | 3.0(2) |
| C17 | C18 | C19 | C23 | 177.31(16) |  | C18 | C17 | B1 | C1 | 86.21(18) |
| C1 | C6 | C5 | C4 | 0.7(3) |  | C18 | C17 | B1 | C25 | -154.57(15) |
| C1 | C6 | C5 | C8 | -177.50(15) |  | C18 | C17 | B1 | C9 | -34.9(2) |
| C1 | C2 | C3 | C4 | 0.9(3) |  | C18 | C19 | C23 | F15 | 172.94(16) |
| C1 | C2 | C3 | C7 | -179.57(16) |  | C18 | C19 | C23 | F14 | 53.0(2) |
| C25 | C26 | C27 | C28 | -3.3(3) |  | C18 | C19 | C23 | F13 | -67.7(2) |
| C25 | C26 | C27 | C31 | 173.20(16) |  | C21 | C20 | C19 | C18 | 2.3(2) |
| C10 | C9 | B1 | C17 | 154.04(14) |  | C21 | C20 | C19 | C23 | -176.20(16) |
| C10 | C9 | B1 | C1 | 37.9(2) |  | C20 | C21 | C24 | F18A | -109.4(5) |
| C10 | C9 | B1 | C25 | -82.90(17) |  | C20 | C21 | C24 | F16A | 14.1(6) |
| C10 | C11 | C12 | C13 | -0.5(2) |  | C20 | C21 | C24 | F17A | 136.9(3) |
| C10 | C11 | C15 | F8 | -74.1(2) |  | C20 | C21 | C24 | F18 | -6.7(5) |
| C10 | C11 | C15 | F7 | 45.2(2) |  | C20 | C21 | C24 | F16 | 114.1(7) |
| C10 | C11 | C15 | F9 | 166.45(16) |  | C20 | C21 | C24 | F17 | -121.5(6) |
| C14 | C13 | C12 | C11 | 0.2(3) |  | C20 | C19 | C23 | F15 | -8.5(2) |
| C14 | C13 | C16 | F12A | 34.6(5) |  | C20 | C19 | C23 | F14 | -128.48(18) |
| C14 | C13 | C16 | F11A | 157.8(6) |  | C20 | C19 | C23 | F13 | 110.78(19) |
| C14 | C13 | C16 | F10A | -79.4(5) |  | C28 | C29 | C30 | C25 | -1.1(3) |
| C14 | C13 | C16 | F10 | 19.2(7) |  | C28 | C29 | C32 | F23 | -128.02(17) |
| C14 | C13 | C16 | F11 | -106.4(6) |  | C28 | C29 | C32 | F22 | -7.8(2) |
| C14 | C13 | C16 | F12 | 138.9(5) |  | C28 | C29 | C32 | F24 | 113.85(18) |
| C14 | C9 | B1 | C17 | -32.8(2) |  | C28 | C27 | C31 | F19 | -119.36(19) |
| C14 | C9 | B1 | C1 | -148.94(15) |  | C28 | C27 | C31 | F21 | 1.6(3) |
| C14 | C9 | B1 | C25 | 90.29(18) |  | C28 | C27 | C31 | F20 | 121.71(19) |
| C6 | C1 | C2 | C3 | -1.2(2) |  | C12 | C13 | C16 | F12A | -148.6(5) |
| C6 | C1 | B1 | C17 | -85.76(17) |  | C12 | C13 | C16 | F11A | -25.4(6) |
| C6 | C1 | B1 | C25 | 154.48(14) |  | C12 | C13 | C16 | F10A | 97.4(5) |
| C6 | C1 | B1 | C9 | 36.8(2) |  | C12 | C13 | C16 | F10 | -164.0(6) |
| C6 | C5 | C4 | C3 | -1.0(2) |  | C12 | C13 | C16 | F11 | 70.4(6) |
| C6 | C5 | C8 | F5A | 48.5(6) |  | C12 | C13 | C16 | F12 | -44.3(6) |
| C6 | C5 | C8 | F6A | 165.3(4) |  | C12 | C11 | C15 | F8 | 105.20(19) |
| C6 | C5 | C8 | F4A | -77.1(5) |  | C12 | C11 | C15 | F7 | -135.56(17) |
| C6 | C5 | C8 | F5 | 61.3(6) |  | C12 | C11 | C15 | F9 | -14.3(2) |
| C6 | C5 | C8 | F4 | -49.8(6) |  | C4 | C3 | C7 | F3 | -12.1(2) |
| C6 | C5 | C8 | F6 | -175.3(7) |  | C4 | C3 | C7 | F1 | 108.23(19) |
| C2 | C1 | C6 | C5 | 0.3(2) |  | C4 | C3 | C7 | F2 | -132.86(18) |
| C2 | C1 | B1 | C17 | 86.51(17) |  | C4 | C5 | C8 | F5A | -129.7(6) |
| C2 | C1 | B1 | C25 | -33.2(2) |  | C4 | C5 | C8 | F6A | -13.0(4) |
| C2 | C1 | B1 | C9 | -150.89(15) |  | C4 | C5 | C8 | F4A | 104.7(5) |
| C2 | C3 | C4 | C5 | 0.2(2) |  | C4 | C5 | C8 | F5 | -117.0(6) |
| C2 | C3 | C7 | F3 | 168.42(15) |  | C4 | C5 | C8 | F4 | 131.9(6) |
| C2 | C3 | C7 | F1 | -71.3(2) |  | C4 | C5 | C8 | F6 | 6.5(7) |
| C2 | C3 | C7 | F2 | 47.6(2) |  | C32 | C29 | C30 | C25 | 178.60(15) |
| C13 | C14 | C9 | C10 | -1.4(2) |  | C32 | C29 | C28 | C27 | -177.02(16) |
| C13 | C14 | C9 | B1 | -174.92(15) |  | C15 | C11 | C12 | C13 | -179.76(16) |
| C9 | C10 | C11 | C12 | -0.2(3) |  | C5B | N1B | C1B | C2B | 0.3(3) |
| C9 | C10 | C11 | C15 | 179.12(15) |  | C5B | C6B | C7B | C8B | 57.4(3) |
| C9 | C14 | C13 | C12 | 0.8(3) |  | C24 | C21 | C20 | C19 | 176.04(16) |
| C9 | C14 | C13 | C16 | 177.56(16) |  | C7 | C3 | C4 | C5 | -179.31(16) |
| C29 | C28 | C27 | C26 | -0.5(3) |  | C8 | C5 | C4 | C3 | 177.21(16) |
| C29 | C28 | C27 | C31 | -176.96(17) |  | C2B | C3B | C4B | C5B | -0.6(3) |
| C22 | C17 | C18 | C19 | -1.4(2) |  | C16 | C13 | C12 | C11 | -176.61(16) |
| C22 | C17 | B1 | C1 | -86.54(17) |  | C9B | N2B | C13B | C12B | 0.2(3) |
| C22 | C17 | B1 | C25 | 32.7(2) |  | C9B | C8B | C7B | C6B | 59.4(3) |
| C22 | C17 | B1 | C9 | 152.38(14) |  | C9B | C10B | C11B | C12B | -0.7(3) |
| C22 | C21 | C20 | C19 | -0.8(2) |  | C3B | C2B | C1B | N1B | -1.2(3) |
| C22 | C21 | C24 | F18A | 67.5(5) |  | C4B | C5B | C6B | C7B | 99.0(2) |
| C22 | C21 | C24 | F16A | -169.0(6) |  | C1B | N1B | C5B | C4B | 0.4(3) |
| C22 | C21 | C24 | F17A | -46.2(4) |  | C1B | N1B | C5B | C6B | 178.64(18) |
| C22 | C21 | C24 | F18 | 170.2(5) |  | C1B | C2B | C3B | C4B | 1.3(3) |
| C22 | C21 | C24 | F16 | -69.0(7) |  | C6B | C5B | C4B | C3B | -178.33(19) |
| C22 | C21 | C24 | F17 | 55.5(6) |  | C8B | C9B | C10B | C11B | -177.9(2) |
| C30 | C25 | C26 | C27 | 4.7(2) |  | C13B | N2B | C9B | C8B | 178.25(18) |
| C30 | C25 | B1 | C17 | -153.21(14) |  | C13B | N2B | C9B | C10B | -1.0(3) |
| C30 | C25 | B1 | C1 | -38.8(2) |  | C13B | C12B | C11B | C10B | -0.1(3) |
| C30 | C25 | B1 | C9 | 82.40(17) |  | B1 | C17 | C22 | C21 | 176.29(15) |
| C30 | C29 | C28 | C27 | 2.7(3) |  | B1 | C17 | C18 | C19 | -174.53(15) |
| C30 | C29 | C32 | F23 | 52.2(2) |  | B1 | C1 | C6 | C5 | 173.09(15) |
| C30 | C29 | C32 | F22 | 172.42(16) |  | B1 | C1 | C2 | C3 | -173.91(15) |
| C30 | C29 | C32 | F24 | -65.9(2) |  | B1 | C25 | C30 | C29 | 178.54(15) |
| C11 | C10 | C9 | C14 | 1.1(2) |  | B1 | C25 | C26 | C27 | -176.37(15) |
| C11 | C10 | C9 | B1 | 174.75(15) |  | C10B | C9B | C8B | C7B | 102.6(2) |

Table 7 Hydrogen Atom Coordinates (Å×104) and Isotropic Displacement Parameters (Å2×103) for c050620\_2\_1.

| Atom | *x* | *y* | *z* | U(eq) |
| --- | --- | --- | --- | --- |
| H1B | 6839(6) | 3540(15) | 4128(13) | 32 |
| H10 | 8743.72 | 7047.22 | 8468.78 | 20 |
| H14 | 7983.03 | 7147.79 | 10482.08 | 20 |
| H6 | 9064.47 | 5353.88 | 9188.22 | 20 |
| H2 | 7118.44 | 5018.37 | 7535.12 | 20 |
| H22 | 6473.03 | 5101.53 | 8905.73 | 21 |
| H30 | 7410.61 | 6846.27 | 7364.3 | 19 |
| H26 | 6401.72 | 6764.96 | 9162.98 | 22 |
| H18 | 8402.04 | 5409.11 | 10604.87 | 21 |
| H20 | 6922.67 | 3667.89 | 11113.17 | 24 |
| H28 | 5402.31 | 7896.51 | 6877.23 | 24 |
| H12 | 9525.62 | 9002.57 | 10305.93 | 23 |
| H4 | 8769.07 | 3390.88 | 7320.7 | 24 |
| H2B | 8442.12 | 1704.14 | 4421.86 | 40 |
| H3B | 9193.36 | 2978.65 | 4341.83 | 38 |
| H4B | 8749.63 | 4539.72 | 4198.34 | 39 |
| H1BA | 7252.11 | 2016.51 | 4298.79 | 40 |
| H6BA | 7759.78 | 5648.93 | 4238.34 | 39 |
| H6BB | 7063.02 | 5194.64 | 4404.29 | 39 |
| H8BA | 6140.54 | 4852.8 | 2130.58 | 42 |
| H8BB | 6583.68 | 4010.66 | 2699.21 | 42 |
| H13B | 5598.78 | 3578.05 | 4803.08 | 45 |
| H7BA | 6746.5 | 6003.83 | 3109.83 | 44 |
| H7BB | 7300.56 | 5382.75 | 2790.16 | 44 |
| H12B | 4487.58 | 4293.96 | 4360.56 | 49 |
| H10B | 5019.89 | 5338.09 | 2351.97 | 50 |
| H11B | 4188.01 | 5191.08 | 3114.02 | 55 |

Table 8 Atomic Occupancy for c050620\_2\_1.

| Atom | *Occupancy* |  | Atom | *Occupancy* |  | Atom | *Occupancy* |
| --- | --- | --- | --- | --- | --- | --- | --- |
| F5A | 0.539(16) |  | F18A | 0.539(16) |  | F12A | 0.539(16) |
| F6A | 0.539(16) |  | F11A | 0.539(16) |  | F16A | 0.539(16) |
| F17A | 0.539(16) |  | F4A | 0.539(16) |  | F10A | 0.539(16) |
| F18 | 0.461(16) |  | F16 | 0.461(16) |  | F17 | 0.461(16) |
| F10 | 0.461(16) |  | F11 | 0.461(16) |  | F12 | 0.461(16) |
| F5 | 0.461(16) |  | F4 | 0.461(16) |  | F6 | 0.461(16) |

Experimental

Single crystals of C45H27BF24N2
[c050620\_2\_1]
were
[].
A suitable crystal was selected and
[]
on a
Bruker APEX-II Duo (Mo)
diffractometer. The crystal was kept at 100.0(1) K during data collection.
Using Olex2 [1], the structure was solved with the
XT
[2] structure solution program using
Intrinsic Phasing
and refined with the
SHELXL
[3] refinement package using
Least Squares
minimisation.

1. Dolomanov, O.V., Bourhis, L.J., Gildea, R.J, Howard, J.A.K. & Puschmann, H.
   (2009), J. Appl. Cryst. 42, 339-341.
2. Sheldrick, G.M. (2015). Acta Cryst. A71, 3-8.
3. Sheldrick, G.M. (2015). Acta Cryst. C71, 3-8.

Crystal structure determination of
[c050620\_2\_1]

**Crystal Data**
for C45H27BF24N2 (*M*=1062.49 g/mol):
monoclinic, space group P21/c (no. 14),
*a* = 19.5780(15) Å, *b* = 13.9630(10) Å, *c* = 16.6113(13) Å, *β* = 106.838(2)°,
*V*= 4346.3(6) Å3,
*Z* = 4,
*T* = 100.0(1) K,
μ(MoKα) = 0.167 mm-1,
*Dcalc* = 1.624 g/cm3,
43270 reflections measured (3.638° ≤ 2Θ ≤ 56.744°),
10838 unique (*R*int = 0.0401, Rsigma = 0.0389) which were used in all calculations.
The final *R*1 was 0.0450
(I > 2σ(I)) and *wR*2 was 0.1082 (all data).

Refinement model description

Number of restraints - 388,
number of constraints - unknown.

Details:

```
1. Fixed Uiso
```

This report has been created with Olex2, compiled on
2020.02.04 svn.rd84adfe8 for OlexSys. Please
let us know
if there are any errors or if you would like to have additional features.
