# Supplementary material for: London Dispersion versus Intramolecular Hydrogen Bond in Bis‐Pyridines: How Accurate Is DFT for Competing Noncovalent Interactions in the Condensed Phase?
Source: Chemistry. 2025 Oct 23;31(66):e02745. doi: 10.1002/chem.202502745 (PMC12648470; doi:10.1002/chem.202502745)

checkCIF/PLATON report

Structure factors have been supplied for datablock(s) c050620_2_1

THIS REPORT IS FOR GUIDANCE ONLY. IF USED AS PART OF A REVIEW PROCEDURE FOR PUBLICATION, IT SHOULD NOT REPLACE THE EXPERTISE OF AN EXPERIENCED CRYSTALLOGRAPHIC REFEREE.

No syntax errors found. CIF dictionary Interpreting this report

Datablock: c050620_2_1

```
Bond precision:  C-C = 0.0026 A          Wavelength=0.71073

Cell:           a=19.5780(15)    b=13.963(1)        c=16.6113(13)
                alpha=90         beta=106.838(2)    gamma=90
Temperature:    100 K

                Calculated                 Reported
Volume          4346.3(6)                  4346.3(6)
Space group     P 21/c                     P 1 21/c 1
Hall group      -P 2ybc                    -P 2ybc
Moiety formula  C32 H12 B F24, C13 H15 N2  C32 H12 B F24, C13 H15 N2
Sum formula     C45 H27 B F24 N2           C45 H27 B F24 N2
Mr              1062.50                    1062.49
Dx,g cm-3       1.624                      1.624
Z               4                          4
Mu (mm-1)       0.167                      0.167
F000            2128.0                     2128.0
F000'           2130.02
h,k,lmax        26,18,22                   26,18,22
Nref            10873                      10838
Tmin,Tmax       0.968,0.983                0.691,0.746
Tmin'           0.961

Correction method= # Reported T Limits: Tmin=0.691 Tmax=0.746
AbsCorr = MULTI-SCAN

Data completeness= 0.997          Theta(max)= 28.372

R(reflections)= 0.0450( 7596)     wR2(reflections)= 0.1082( 10838)

S = 1.010                  Npar= 734
```

The following ALERTS were generated. Each ALERT has the format
test-name_ALERT_alert-type_alert-level.
Click on the hyperlinks for more details of the test.

Alert level C

```
PLAT213_ALERT_2_C Atom F16A            has ADP max/min Ratio .....        3.9 prolat
PLAT242_ALERT_2_C Low    'MainMol' Ueq as Compared to Neighbors of        C15 Check
```

Alert level G

```
PLAT002_ALERT_2_G Number of Distance or Angle Restraints on AtSite         23 Note
PLAT003_ALERT_2_G Number of Uiso or Uij Restrained non-H Atoms ...         21 Report
PLAT172_ALERT_4_G The CIF-Embedded .res File Contains DFIX Records          1 Report
PLAT176_ALERT_4_G The CIF-Embedded .res File Contains SADI Records          6 Report
PLAT186_ALERT_4_G The CIF-Embedded .res File Contains ISOR Records          3 Report
PLAT187_ALERT_4_G The CIF-Embedded .res File Contains RIGU Records          3 Report
PLAT242_ALERT_2_G Low    'MainMol' Ueq as Compared to Neighbors of         C7 Check
PLAT242_ALERT_2_G Low    'MainMol' Ueq as Compared to Neighbors of         C8 Check
PLAT242_ALERT_2_G Low    'MainMol' Ueq as Compared to Neighbors of        C16 Check
PLAT242_ALERT_2_G Low    'MainMol' Ueq as Compared to Neighbors of        C23 Check
PLAT242_ALERT_2_G Low    'MainMol' Ueq as Compared to Neighbors of        C24 Check
PLAT242_ALERT_2_G Low    'MainMol' Ueq as Compared to Neighbors of        C31 Check
PLAT301_ALERT_3_G Main Residue  Disorder ..............(Resd  1  )        16% Note
PLAT434_ALERT_2_G Short Inter HL..HL Contact F7       ..F14              2.72 Ang.
                                              x,3/2-y,-1/2+z  =      4_575 Check
PLAT434_ALERT_2_G Short Inter HL..HL Contact F7       ..F6               2.72 Ang.
                                             2-x,1/2+y,3/2-z  =      2_756 Check
PLAT434_ALERT_2_G Short Inter HL..HL Contact F20      ..F18              2.81 Ang.
                                                 1-x,1-y,2-z  =      3_667 Check
PLAT720_ALERT_4_G Number of Unusual/Non-Standard Labels ..........          7 Note
PLAT860_ALERT_3_G Number of Least-Squares Restraints .............        388 Note
PLAT910_ALERT_3_G Missing # of FCF Reflection(s) Below Theta(Min).          1 Note
PLAT912_ALERT_4_G Missing # of FCF Reflections Above STh/L=  0.600         33 Note
PLAT941_ALERT_3_G Average HKL Measurement Multiplicity ...........        4.0 Low
PLAT978_ALERT_2_G Number C-C Bonds with Positive Residual Density.         13 Info
```

---

 0 **ALERT level A** = Most likely a serious problem - resolve or explain
 0 **ALERT level B** = A potentially serious problem, consider carefully
 2 **ALERT level C** = Check. Ensure it is not caused by an omission or oversight
22 **ALERT level G** = General information/check it is not something unexpected

```
 0 ALERT type 1 CIF construction/syntax error, inconsistent or missing data
14 ALERT type 2 Indicator that the structure model may be wrong or deficient
 4 ALERT type 3 Indicator that the structure quality may be low
 6 ALERT type 4 Improvement, methodology, query or suggestion
 0 ALERT type 5 Informative message, check
```

It is advisable to attempt to resolve as many as possible of the alerts in all categories. Often the minor alerts point to easily fixed oversights, errors and omissions in your CIF or refinement strategy, so attention to these fine details can be worthwhile. In order to resolve some of the more serious problems it may be necessary to carry out additional measurements or structure refinements. However, the purpose of your study may justify the reported deviations and the more serious of these should normally be commented upon in the discussion or experimental section of a paper or in the "special_details" fields of the CIF. checkCIF was carefully designed to identify outliers and unusual parameters, but every test has its limitations and alerts that are not important in a particular case may appear. Conversely, the absence of alerts does not guarantee there are no aspects of the results needing attention. It is up to the individual to critically assess their own results and, if necessary, seek expert advice.

Publication of your CIF in IUCr journals

A basic structural check has been run on your CIF. These basic checks will be run on all CIFs submitted for publication in IUCr journals (*Acta Crystallographica*, *Journal of Applied Crystallography*, *Journal of Synchrotron Radiation*); however, if you intend to submit to *Acta Crystallographica Section C* or *E* or *IUCrData*, you should make sure that full publication checks are run on the final version of your CIF prior to submission.

Publication of your CIF in other journals

Please refer to the *Notes for Authors* of the relevant journal for any special instructions relating to CIF submission.

PLATON version of 04/06/2020; check.def file version of 02/06/2020

Datablock c050620_2_1 - ellipsoid plot

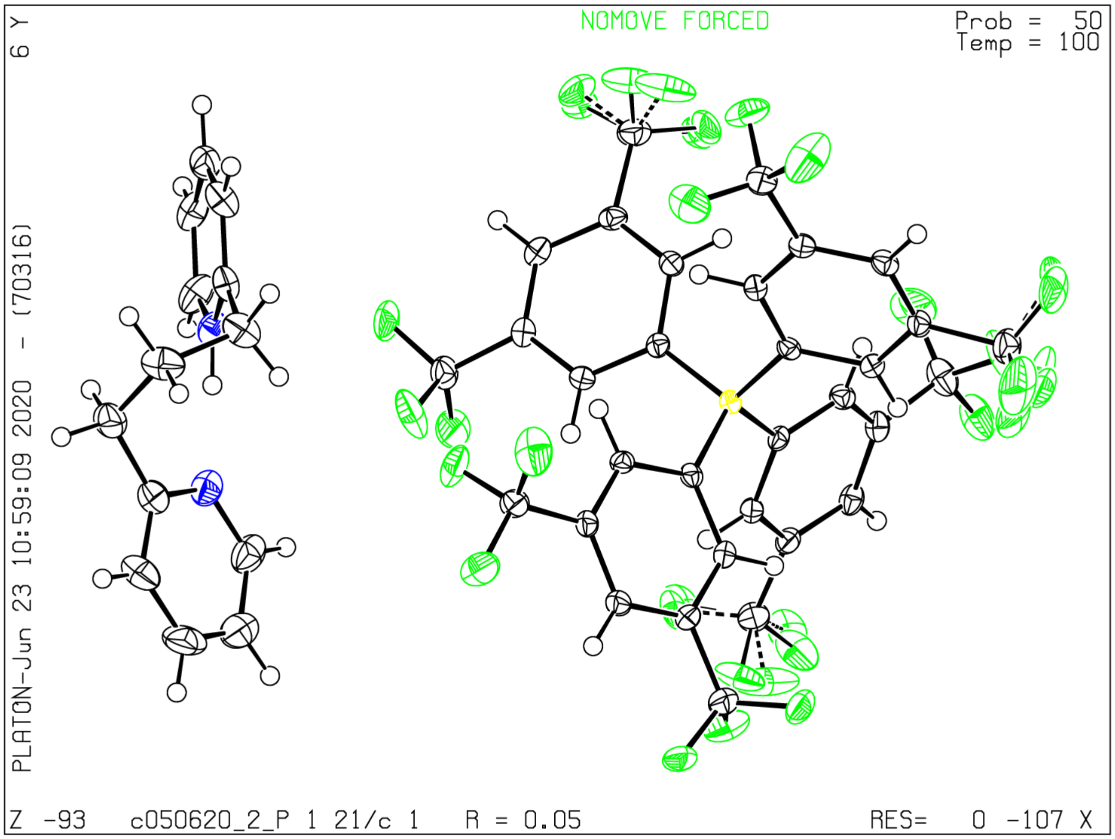

Supplement: Supplementary file 1 — Supporting Information [file CHEM-31-e02745-s002.zip › Crystal_structures/14b/checkcif.pdf]
