# Supplementary material for: London Dispersion versus Intramolecular Hydrogen Bond in Bis‐Pyridines: How Accurate Is DFT for Competing Noncovalent Interactions in the Condensed Phase?
Source: Chemistry. 2025 Oct 23;31(66):e02745. doi: 10.1002/chem.202502745 (PMC12648470; doi:10.1002/chem.202502745)
Supplement: Supplementary file 1 — Supporting Information [file CHEM-31-e02745-s002.zip › Crystal_structures/1b/c041219_2_1_tables.html]

c041219\_2\_1


# c041219\_2\_1

Table 1 Crystal data and structure refinement for c041219\_2\_1.

| Identification code | c041219\_2\_1 |
| Empirical formula | C44H25BF24N2 |
| Formula weight | 1048.47 |
| Temperature/K | 100.0(1) |
| Crystal system | triclinic |
| Space group | P-1 |
| a/Å | 9.34770(10) |
| b/Å | 15.6602(2) |
| c/Å | 16.1982(2) |
| α/° | 73.8830(10) |
| β/° | 76.1990(10) |
| γ/° | 75.6590(10) |
| Volume/Å3 | 2169.99(5) |
| Z | 2 |
| ρcalcg/cm3 | 1.605 |
| μ/mm‑1 | 1.503 |
| F(000) | 1048.0 |
| Crystal size/mm3 | 0.149 × 0.056 × 0.037 |
| Radiation | Cu Kα (λ = 1.54184) |
| 2Θ range for data collection/° | 5.776 to 159.598 |
| Index ranges | -11 ≤ h ≤ 11, -19 ≤ k ≤ 19, -20 ≤ l ≤ 20 |
| Reflections collected | 60128 |
| Independent reflections | 9225 [Rint = 0.0519, Rsigma = 0.0296] |
| Data/restraints/parameters | 9225/358/679 |
| Goodness-of-fit on F2 | 1.085 |
| Final R indexes [I>=2σ (I)] | R1 = 0.0585, wR2 = 0.1650 |
| Final R indexes [all data] | R1 = 0.0690, wR2 = 0.1742 |
| Largest diff. peak/hole / e Å-3 | 1.19/-0.44 |

Table 2 Fractional Atomic Coordinates (×104) and Equivalent Isotropic Displacement Parameters (Å2×103) for c041219\_2\_1. Ueq is defined as 1/3 of of the trace of the orthogonalised UIJ tensor.

| Atom | *x* | *y* | *z* | U(eq) |
| F1A | 8520.0(19) | 4840.6(11) | 1639.4(10) | 44.6(4) |
| F2A | 8507(2) | 5258.8(13) | 2800.5(13) | 62.1(5) |
| F3A | 9851.4(17) | 4007.8(13) | 2571.5(12) | 52.8(4) |
| F4A | 5553(2) | 4052.4(12) | 5574.9(10) | 51.6(4) |
| F5A | 3416.6(19) | 4151.4(12) | 5264.5(11) | 48.3(4) |
| F6A | 4684(2) | 2841.6(10) | 5726.9(9) | 53.3(5) |
| F7A | -155.4(18) | 2986.0(11) | 4577.6(11) | 46.6(4) |
| F8A | -1033.3(17) | 2133.7(12) | 4079.4(13) | 51.3(4) |
| F9A | -536(2) | 1688.8(14) | 5379.1(12) | 64.6(6) |
| F10A | 5420.8(19) | -889.5(10) | 4246.6(14) | 56.9(5) |
| F11A | 3488(2) | -1037.6(11) | 3840.8(12) | 52.6(4) |
| F12A | 3383(2) | -1008.0(11) | 5162.4(11) | 52.8(4) |
| F13A | 4224.3(17) | 5908.7(10) | 750.9(11) | 42.0(4) |
| F14A | 2750.8(18) | 6080.2(10) | -146.8(10) | 40.8(4) |
| F15A | 1837.2(18) | 6120.8(10) | 1195.1(10) | 41.0(4) |
| F16A | 1777.0(19) | 2203.5(11) | 373.0(11) | 44.6(4) |
| F17A | 643(2) | 3543.7(12) | -186.5(11) | 50.5(4) |
| F18A | -124.9(17) | 2888.2(12) | 1141.7(11) | 46.0(4) |
| F19A | 9784(17) | 378(10) | 3369(7) | 54(2) |
| F20A | 11386(9) | 522(11) | 2201(8) | 88(3) |
| F21A | 10257(18) | -548(5) | 2564(9) | 72(3) |
| F22A | 9537(2) | 1345.7(17) | -664.7(11) | 67.2(6) |
| F23A | 7761(2) | 624.6(11) | -318.4(11) | 52.2(4) |
| F24A | 7268(2) | 2068.2(11) | -679.0(10) | 51.5(4) |
| C1A | 5567(2) | 3080.3(13) | 3004.8(13) | 22.0(4) |
| C2A | 5021(3) | 3113.8(14) | 3878.6(14) | 25.2(4) |
| C3A | 5553(3) | 3618.7(15) | 4288.0(14) | 27.8(5) |
| C4A | 6667(3) | 4101.2(15) | 3845.0(15) | 27.9(5) |
| C5A | 7242(2) | 4072.2(14) | 2974.7(14) | 25.8(4) |
| C6A | 6710(2) | 3570.9(14) | 2567.2(14) | 24.2(4) |
| C7A | 4851(3) | 3649.3(17) | 5212.6(16) | 38.3(6) |
| C8A | 8516(3) | 4543.4(17) | 2495.2(16) | 32.2(5) |
| C9A | 3900(2) | 1839.8(14) | 3197.4(13) | 22.9(4) |
| C10A | 2446(3) | 2209.6(15) | 3574.3(14) | 26.5(4) |
| C11A | 1518(3) | 1684.3(16) | 4203.5(14) | 28.5(5) |
| C12A | 1999(3) | 754.7(16) | 4476.1(15) | 30.2(5) |
| C13A | 3422(3) | 371.8(15) | 4101.6(14) | 27.6(4) |
| C14A | 4349(3) | 904.3(15) | 3477.2(14) | 25.5(4) |
| C15A | -41(3) | 2115.0(18) | 4558.7(17) | 37.3(5) |
| C16A | 3928(3) | -634.7(16) | 4338.9(17) | 34.7(5) |
| C17A | 3943(2) | 3199.2(14) | 1791.8(13) | 22.3(4) |
| C18A | 3911(2) | 4130.8(14) | 1503.8(14) | 24.5(4) |
| C19A | 3024(2) | 4690.0(14) | 910.4(14) | 25.9(4) |
| C20A | 2127(3) | 4341.7(16) | 568.5(14) | 28.4(5) |
| C21A | 2136(3) | 3419.0(16) | 842.3(14) | 27.7(5) |
| C22A | 3022(2) | 2863.1(15) | 1442.9(14) | 26.3(4) |
| C23A | 2966(3) | 5693.8(16) | 672.4(16) | 31.8(5) |
| C24A | 1124(3) | 3016.1(18) | 540.4(17) | 35.9(5) |
| C25A | 6454(2) | 1919.2(13) | 1986.7(13) | 22.1(4) |
| C26A | 7605(2) | 1408.5(14) | 2436.6(14) | 25.6(4) |
| C27A | 8887(3) | 894.1(15) | 2039.5(15) | 27.5(4) |
| C28A | 9077(3) | 885.3(15) | 1165.3(15) | 28.9(5) |
| C29A | 7959(3) | 1389.2(14) | 703.9(14) | 26.7(4) |
| C30A | 6666(2) | 1895.1(14) | 1107.3(14) | 24.4(4) |
| C31A | 10052(13) | 316(7) | 2548(8) | 39(3) |
| C32A | 8131(3) | 1367.9(17) | -230.9(16) | 34.1(5) |
| B1A | 4964(3) | 2507.4(16) | 2493.0(15) | 22.3(4) |
| N1 | 6348(4) | 4073(2) | 7186.1(18) | 62.0(8) |
| N2 | 4178(4) | 3194(2) | 7887(2) | 68.3(8) |
| C1 | 10886(5) | 2946(3) | 6447(3) | 79.1(12) |
| C2 | 4692(6) | 302(3) | 8438(3) | 92.4(15) |
| C3 | 7269(6) | 4623(2) | 6835(2) | 70.0(11) |
| C4 | 8759(6) | 4277(3) | 6580(3) | 69.9(10) |
| C5 | 9267(5) | 3338(2) | 6707(2) | 59.6(8) |
| C6 | 8232(4) | 2770(2) | 7079(2) | 51.0(7) |
| C7 | 6732(4) | 3149(2) | 7331(2) | 49.1(7) |
| C8 | 5518(4) | 2645(2) | 7710.4(19) | 52.7(7) |
| C9 | 5720(4) | 1724(2) | 7872(2) | 53.1(7) |
| C10 | 4503(4) | 1307(3) | 8237(2) | 65.2(9) |
| C11 | 3093(5) | 1880(4) | 8413(3) | 78.8(12) |
| C12 | 3015(5) | 2794(4) | 8232(3) | 83.4(12) |
| C31B | 10140(11) | 407(7) | 2534(7) | 36(2) |
| F21B | 10808(15) | -369(9) | 2318(8) | 81(3) |
| F20B | 11219(12) | 863(9) | 2390(6) | 71(3) |
| F19B | 9678(15) | 204(9) | 3394(6) | 55(2) |

Table 3 Anisotropic Displacement Parameters (Å2×103) for c041219\_2\_1. The Anisotropic displacement factor exponent takes the form: -2π2[h2a\*2U11+2hka\*b\*U12+…].

| Atom | U11 | U22 | U33 | U23 | U13 | U12 |
| F1A | 49.6(9) | 49.5(9) | 35.7(8) | 6.0(7) | -8.9(7) | -28.7(7) |
| F2A | 69.7(12) | 59.8(11) | 71.6(12) | -36.5(10) | 16.0(10) | -43.2(10) |
| F3A | 27.0(8) | 67.7(11) | 53.0(10) | 2.4(8) | -5.0(7) | -10.6(7) |
| F4A | 76.4(12) | 55.6(10) | 32.0(8) | -17.0(7) | -10.9(8) | -21.2(9) |
| F5A | 52.9(10) | 48.4(9) | 40.2(8) | -19.2(7) | 6.8(7) | -8.1(8) |
| F6A | 97.8(14) | 30.8(8) | 24.6(7) | -2.7(6) | -0.8(8) | -14.7(8) |
| F7A | 36.9(8) | 45.5(9) | 53.3(9) | -19.2(7) | 6.1(7) | -5.5(7) |
| F8A | 27.3(8) | 52.4(10) | 75.3(12) | -16.0(9) | -12.4(8) | -5.5(7) |
| F9A | 46.4(10) | 65.8(12) | 46.6(10) | 10.3(8) | 20.9(8) | -2.0(8) |
| F10A | 37.3(9) | 28.0(8) | 90.4(14) | 9.3(8) | -11.4(9) | -5.0(6) |
| F11A | 75.4(12) | 30.2(8) | 58.3(10) | -10.2(7) | -23.8(9) | -10.6(8) |
| F12A | 70.9(12) | 33.6(8) | 40.5(9) | 12.7(7) | -4.7(8) | -15.3(8) |
| F13A | 40.8(8) | 27.7(7) | 58.1(9) | 4.8(6) | -20.7(7) | -13.1(6) |
| F14A | 49.0(9) | 32.7(7) | 34.4(7) | 10.5(6) | -14.8(6) | -10.1(6) |
| F15A | 44.3(8) | 28.6(7) | 45.1(8) | -6.8(6) | -7.2(7) | -0.7(6) |
| F16A | 49.6(9) | 43.9(9) | 50.0(9) | -18.5(7) | -19.5(7) | -8.4(7) |
| F17A | 60.4(11) | 52.4(10) | 46.4(9) | 4.1(7) | -33.3(8) | -19.5(8) |
| F18A | 35.6(8) | 52.8(9) | 54.8(9) | -11.5(8) | -7.8(7) | -19.6(7) |
| F19A | 55(4) | 63(5) | 45(4) | -25(4) | -28(3) | 19(3) |
| F20A | 30(2) | 118(6) | 80(5) | 28(4) | -12(3) | -4(4) |
| F21A | 98(6) | 30(2) | 91(5) | -13(3) | -57(4) | 18(3) |
| F22A | 47.6(10) | 122.7(18) | 34.1(9) | -29.8(10) | 10.5(7) | -25.6(11) |
| F23A | 83.1(13) | 41.8(9) | 39.0(8) | -19.8(7) | -10.7(8) | -14.0(8) |
| F24A | 81.8(13) | 39.5(8) | 27.3(7) | -9.4(6) | -16.2(8) | 6.6(8) |
| C1A | 21.6(10) | 19.2(9) | 23.0(10) | -3.1(7) | -6.5(8) | 0.0(7) |
| C2A | 29.3(11) | 21.4(10) | 23.7(10) | -3.7(8) | -5.0(8) | -4.3(8) |
| C3A | 34.2(12) | 24.1(10) | 24.4(10) | -6.1(8) | -5.8(9) | -3.7(9) |
| C4A | 31.8(12) | 23.0(10) | 30.9(11) | -7.6(8) | -10.8(9) | -2.5(8) |
| C5A | 25.7(11) | 22.7(10) | 28.3(10) | -5.1(8) | -6.6(8) | -2.8(8) |
| C6A | 25.5(10) | 22.6(10) | 23.6(10) | -4.2(8) | -5.8(8) | -3.1(8) |
| C7A | 58.6(17) | 31.5(12) | 27.4(11) | -8.6(9) | -4.1(11) | -15.2(11) |
| C8A | 31.2(12) | 33.7(12) | 34.7(12) | -10.3(10) | -5.4(9) | -10.1(10) |
| C9A | 25.0(10) | 24.5(10) | 20.1(9) | -2.3(8) | -6.1(8) | -7.5(8) |
| C10A | 27.5(11) | 25.9(10) | 24.6(10) | -3.0(8) | -5.3(8) | -5.3(8) |
| C11A | 25.2(11) | 31.6(11) | 26.5(10) | -1.4(9) | -3.8(8) | -8.2(9) |
| C12A | 30.1(11) | 33.3(12) | 26.3(11) | 1.3(9) | -4.3(9) | -14.6(9) |
| C13A | 30.1(11) | 25.2(10) | 26.9(10) | 1.1(8) | -8.9(9) | -9.1(9) |
| C14A | 26.9(11) | 24.8(10) | 24.4(10) | -2.5(8) | -6.1(8) | -6.7(8) |
| C15A | 30.4(12) | 37.4(13) | 36.8(13) | -0.6(10) | 1.7(10) | -9.8(10) |
| C16A | 35.8(13) | 28.9(12) | 37.1(12) | 2.5(9) | -7.4(10) | -12.7(10) |
| C17A | 20.9(10) | 23.9(10) | 19.5(9) | -3.1(7) | -1.0(7) | -4.7(8) |
| C18A | 23.6(10) | 24.8(10) | 24.5(10) | -4.2(8) | -3.7(8) | -5.8(8) |
| C19A | 25.3(10) | 23.7(10) | 25.0(10) | -0.1(8) | -3.4(8) | -5.5(8) |
| C20A | 26.0(11) | 30.7(11) | 24.9(10) | 1.0(8) | -7.8(8) | -4.3(9) |
| C21A | 25.9(11) | 31.3(11) | 25.4(10) | -2.8(8) | -5.9(8) | -7.5(9) |
| C22A | 26.5(11) | 26.0(10) | 25.2(10) | -3.1(8) | -4.5(8) | -6.5(8) |
| C23A | 31.3(12) | 27.9(11) | 32.5(12) | 1.9(9) | -10.4(9) | -4.7(9) |
| C24A | 37.5(13) | 37.3(13) | 35.1(12) | -3.3(10) | -14.9(10) | -9.1(10) |
| C25A | 25.0(10) | 18.5(9) | 22.6(9) | -2.9(7) | -2.7(8) | -7.8(8) |
| C26A | 28.3(11) | 24.0(10) | 23.1(10) | -3.2(8) | -4.2(8) | -5.7(8) |
| C27A | 28.3(11) | 23.4(10) | 28.8(11) | -4.9(8) | -5.1(9) | -3.0(8) |
| C28A | 29.2(11) | 25.1(10) | 30.0(11) | -8.3(9) | -0.1(9) | -3.9(9) |
| C29A | 32.5(12) | 22.6(10) | 24.5(10) | -5.6(8) | -1.8(9) | -7.8(9) |
| C30A | 27.9(11) | 21.6(10) | 24.1(10) | -3.7(8) | -5.8(8) | -6.4(8) |
| C31A | 37(4) | 38(4) | 38(4) | -9(3) | -9(3) | 2(3) |
| C32A | 38.0(13) | 33.9(12) | 28.8(11) | -10.8(9) | -2.2(10) | -4.0(10) |
| B1A | 22.9(11) | 21.5(11) | 21.5(10) | -2.6(8) | -4.1(9) | -5.2(9) |
| N1 | 82(2) | 58.5(16) | 46.5(14) | -22.0(13) | -29.4(14) | 13.3(14) |
| N2 | 55.4(17) | 86(2) | 52.9(16) | -23.7(15) | -1.9(13) | 6.7(15) |
| C1 | 71(2) | 84(3) | 87(3) | -12(2) | -16(2) | -33(2) |
| C2 | 98(3) | 99(3) | 86(3) | -33(3) | 27(3) | -59(3) |
| C3 | 128(3) | 38.1(16) | 49.6(18) | -8.6(14) | -44(2) | -1.7(19) |
| C4 | 109(3) | 50.9(19) | 61(2) | -4.6(16) | -36(2) | -26(2) |
| C5 | 79(2) | 54.0(18) | 52.8(18) | -5.4(14) | -25.7(17) | -20.2(16) |
| C6 | 63.1(19) | 48.0(16) | 45.5(16) | -8.9(13) | -18.9(14) | -10.8(14) |
| C7 | 57.9(17) | 44.1(15) | 47.1(16) | -15.0(12) | -22.9(13) | 4.3(13) |
| C8 | 55.1(17) | 66.3(19) | 37.3(14) | -21.1(14) | -16.4(13) | 5.3(14) |
| C9 | 45.5(16) | 71(2) | 50.3(17) | -28.3(15) | -3.9(13) | -14.0(15) |
| C10 | 63(2) | 88(3) | 49.7(18) | -26.3(18) | 6.6(16) | -29.1(19) |
| C11 | 54(2) | 123(3) | 56(2) | -23(2) | 11.0(17) | -30(2) |
| C12 | 69(3) | 103(3) | 64(2) | -20(2) | 3(2) | -4(2) |
| C31B | 32(3) | 36(3) | 35(3) | -7(3) | -7(3) | 4(2) |
| F21B | 82(5) | 78(5) | 80(5) | -48(4) | -45(4) | 52(3) |
| F20B | 47(4) | 90(5) | 72(4) | 20(3) | -35(3) | -29(3) |
| F19B | 44(3) | 63(4) | 32(3) | 16(2) | -7(2) | 6(3) |

Table 4 Bond Lengths for c041219\_2\_1.

| Atom | Atom | Length/Å |  | Atom | Atom | Length/Å |
| F1A | C8A | 1.334(3) |  | C13A | C14A | 1.393(3) |
| F2A | C8A | 1.342(3) |  | C13A | C16A | 1.496(3) |
| F3A | C8A | 1.332(3) |  | C17A | C18A | 1.398(3) |
| F4A | C7A | 1.330(3) |  | C17A | C22A | 1.400(3) |
| F5A | C7A | 1.372(3) |  | C17A | B1A | 1.642(3) |
| F6A | C7A | 1.330(3) |  | C18A | C19A | 1.390(3) |
| F7A | C15A | 1.350(3) |  | C19A | C20A | 1.387(3) |
| F8A | C15A | 1.335(3) |  | C19A | C23A | 1.501(3) |
| F9A | C15A | 1.339(3) |  | C20A | C21A | 1.387(3) |
| F10A | C16A | 1.336(3) |  | C21A | C22A | 1.395(3) |
| F11A | C16A | 1.337(3) |  | C21A | C24A | 1.490(3) |
| F12A | C16A | 1.331(3) |  | C25A | C26A | 1.398(3) |
| F13A | C23A | 1.343(3) |  | C25A | C30A | 1.401(3) |
| F14A | C23A | 1.337(3) |  | C25A | B1A | 1.643(3) |
| F15A | C23A | 1.348(3) |  | C26A | C27A | 1.393(3) |
| F16A | C24A | 1.339(3) |  | C27A | C28A | 1.388(3) |
| F17A | C24A | 1.342(3) |  | C27A | C31A | 1.487(10) |
| F18A | C24A | 1.348(3) |  | C27A | C31B | 1.503(9) |
| F19A | C31A | 1.319(10) |  | C28A | C29A | 1.382(3) |
| F20A | C31A | 1.319(10) |  | C29A | C30A | 1.396(3) |
| F21A | C31A | 1.312(10) |  | C29A | C32A | 1.493(3) |
| F22A | C32A | 1.332(3) |  | N1 | C3 | 1.291(5) |
| F23A | C32A | 1.345(3) |  | N1 | C7 | 1.369(4) |
| F24A | C32A | 1.334(3) |  | N2 | C8 | 1.349(4) |
| C1A | C2A | 1.396(3) |  | N2 | C12 | 1.319(6) |
| C1A | C6A | 1.407(3) |  | C1 | C5 | 1.492(6) |
| C1A | B1A | 1.642(3) |  | C2 | C10 | 1.491(6) |
| C2A | C3A | 1.399(3) |  | C3 | C4 | 1.371(6) |
| C3A | C4A | 1.379(3) |  | C4 | C5 | 1.401(5) |
| C3A | C7A | 1.494(3) |  | C5 | C6 | 1.394(5) |
| C4A | C5A | 1.393(3) |  | C6 | C7 | 1.390(5) |
| C5A | C6A | 1.392(3) |  | C7 | C8 | 1.460(5) |
| C5A | C8A | 1.498(3) |  | C8 | C9 | 1.365(5) |
| C9A | C10A | 1.402(3) |  | C9 | C10 | 1.380(5) |
| C9A | C14A | 1.395(3) |  | C10 | C11 | 1.415(6) |
| C9A | B1A | 1.642(3) |  | C11 | C12 | 1.366(7) |
| C10A | C11A | 1.390(3) |  | C31B | F21B | 1.322(9) |
| C11A | C12A | 1.389(3) |  | C31B | F20B | 1.316(10) |
| C11A | C15A | 1.497(3) |  | C31B | F19B | 1.329(9) |
| C12A | C13A | 1.385(3) |  |  |  |  |

Table 5 Bond Angles for c041219\_2\_1.

| Atom | Atom | Atom | Angle/˚ |  | Atom | Atom | Atom | Angle/˚ |
| C2A | C1A | C6A | 115.73(19) |  | F15A | C23A | C19A | 111.63(19) |
| C2A | C1A | B1A | 123.98(18) |  | F16A | C24A | F17A | 107.1(2) |
| C6A | C1A | B1A | 120.29(18) |  | F16A | C24A | F18A | 106.0(2) |
| C1A | C2A | C3A | 122.2(2) |  | F16A | C24A | C21A | 112.7(2) |
| C2A | C3A | C7A | 118.4(2) |  | F17A | C24A | F18A | 105.8(2) |
| C4A | C3A | C2A | 121.1(2) |  | F17A | C24A | C21A | 112.6(2) |
| C4A | C3A | C7A | 120.5(2) |  | F18A | C24A | C21A | 112.1(2) |
| C3A | C4A | C5A | 117.8(2) |  | C26A | C25A | C30A | 116.27(19) |
| C4A | C5A | C8A | 118.9(2) |  | C26A | C25A | B1A | 119.87(18) |
| C6A | C5A | C4A | 121.0(2) |  | C30A | C25A | B1A | 123.86(19) |
| C6A | C5A | C8A | 120.0(2) |  | C27A | C26A | C25A | 122.1(2) |
| C5A | C6A | C1A | 122.1(2) |  | C26A | C27A | C31A | 120.9(5) |
| F4A | C7A | F5A | 104.2(2) |  | C26A | C27A | C31B | 120.1(4) |
| F4A | C7A | F6A | 109.6(2) |  | C28A | C27A | C26A | 120.5(2) |
| F4A | C7A | C3A | 113.6(2) |  | C28A | C27A | C31A | 118.5(5) |
| F5A | C7A | C3A | 111.0(2) |  | C28A | C27A | C31B | 119.2(4) |
| F6A | C7A | F5A | 104.1(2) |  | C29A | C28A | C27A | 118.5(2) |
| F6A | C7A | C3A | 113.5(2) |  | C28A | C29A | C30A | 120.8(2) |
| F1A | C8A | F2A | 107.2(2) |  | C28A | C29A | C32A | 119.0(2) |
| F1A | C8A | C5A | 113.09(19) |  | C30A | C29A | C32A | 120.1(2) |
| F2A | C8A | C5A | 112.3(2) |  | C29A | C30A | C25A | 121.7(2) |
| F3A | C8A | F1A | 106.0(2) |  | F19A | C31A | F20A | 106.2(9) |
| F3A | C8A | F2A | 105.3(2) |  | F19A | C31A | C27A | 114.1(10) |
| F3A | C8A | C5A | 112.5(2) |  | F20A | C31A | C27A | 111.6(9) |
| C10A | C9A | B1A | 119.80(18) |  | F21A | C31A | F19A | 106.7(9) |
| C14A | C9A | C10A | 115.75(19) |  | F21A | C31A | F20A | 104.0(8) |
| C14A | C9A | B1A | 124.43(19) |  | F21A | C31A | C27A | 113.4(8) |
| C11A | C10A | C9A | 122.4(2) |  | F22A | C32A | F23A | 104.5(2) |
| C10A | C11A | C15A | 119.8(2) |  | F22A | C32A | F24A | 107.2(2) |
| C12A | C11A | C10A | 120.6(2) |  | F22A | C32A | C29A | 113.2(2) |
| C12A | C11A | C15A | 119.5(2) |  | F23A | C32A | C29A | 112.4(2) |
| C13A | C12A | C11A | 118.0(2) |  | F24A | C32A | F23A | 105.4(2) |
| C12A | C13A | C14A | 121.0(2) |  | F24A | C32A | C29A | 113.44(19) |
| C12A | C13A | C16A | 119.1(2) |  | C1A | B1A | C9A | 109.93(16) |
| C14A | C13A | C16A | 119.8(2) |  | C1A | B1A | C17A | 110.48(16) |
| C13A | C14A | C9A | 122.2(2) |  | C1A | B1A | C25A | 107.08(16) |
| F7A | C15A | C11A | 112.7(2) |  | C9A | B1A | C17A | 107.61(17) |
| F8A | C15A | F7A | 105.4(2) |  | C9A | B1A | C25A | 111.11(17) |
| F8A | C15A | F9A | 107.1(2) |  | C17A | B1A | C25A | 110.64(16) |
| F8A | C15A | C11A | 112.4(2) |  | C3 | N1 | C7 | 125.2(3) |
| F9A | C15A | F7A | 106.0(2) |  | C12 | N2 | C8 | 116.3(4) |
| F9A | C15A | C11A | 112.6(2) |  | N1 | C3 | C4 | 119.2(3) |
| F10A | C16A | F11A | 106.8(2) |  | C3 | C4 | C5 | 120.0(4) |
| F10A | C16A | C13A | 112.78(19) |  | C4 | C5 | C1 | 121.0(4) |
| F11A | C16A | C13A | 111.5(2) |  | C6 | C5 | C1 | 120.0(3) |
| F12A | C16A | F10A | 106.0(2) |  | C6 | C5 | C4 | 119.0(4) |
| F12A | C16A | F11A | 106.1(2) |  | C7 | C6 | C5 | 119.1(3) |
| F12A | C16A | C13A | 113.1(2) |  | N1 | C7 | C6 | 117.6(3) |
| C18A | C17A | C22A | 115.64(19) |  | N1 | C7 | C8 | 117.0(3) |
| C18A | C17A | B1A | 124.48(18) |  | C6 | C7 | C8 | 125.4(3) |
| C22A | C17A | B1A | 119.88(18) |  | N2 | C8 | C7 | 112.2(3) |
| C19A | C18A | C17A | 122.4(2) |  | N2 | C8 | C9 | 124.1(4) |
| C18A | C19A | C23A | 119.7(2) |  | C9 | C8 | C7 | 123.7(3) |
| C20A | C19A | C18A | 121.0(2) |  | C8 | C9 | C10 | 119.6(3) |
| C20A | C19A | C23A | 119.2(2) |  | C9 | C10 | C2 | 120.9(4) |
| C19A | C20A | C21A | 117.9(2) |  | C9 | C10 | C11 | 116.6(4) |
| C20A | C21A | C22A | 120.8(2) |  | C11 | C10 | C2 | 122.5(4) |
| C20A | C21A | C24A | 120.3(2) |  | C12 | C11 | C10 | 119.1(4) |
| C22A | C21A | C24A | 118.9(2) |  | N2 | C12 | C11 | 124.3(4) |
| C21A | C22A | C17A | 122.3(2) |  | F21B | C31B | C27A | 111.6(8) |
| F13A | C23A | F15A | 105.8(2) |  | F21B | C31B | F19B | 106.0(8) |
| F13A | C23A | C19A | 112.74(19) |  | F20B | C31B | C27A | 114.0(8) |
| F14A | C23A | F13A | 106.94(19) |  | F20B | C31B | F21B | 105.3(8) |
| F14A | C23A | F15A | 106.38(19) |  | F20B | C31B | F19B | 106.2(8) |
| F14A | C23A | C19A | 112.8(2) |  | F19B | C31B | C27A | 113.1(9) |

Table 6 Torsion Angles for c041219\_2\_1.

| A | B | C | D | Angle/˚ |  | A | B | C | D | Angle/˚ |
| C1A | C2A | C3A | C4A | -0.9(3) |  | C22A | C17A | B1A | C1A | -164.38(18) |
| C1A | C2A | C3A | C7A | 177.1(2) |  | C22A | C17A | B1A | C9A | -44.4(2) |
| C2A | C1A | C6A | C5A | -1.2(3) |  | C22A | C17A | B1A | C25A | 77.2(2) |
| C2A | C1A | B1A | C9A | -11.0(3) |  | C22A | C21A | C24A | F16A | -42.4(3) |
| C2A | C1A | B1A | C17A | 107.6(2) |  | C22A | C21A | C24A | F17A | -163.7(2) |
| C2A | C1A | B1A | C25A | -131.8(2) |  | C22A | C21A | C24A | F18A | 77.1(3) |
| C2A | C3A | C4A | C5A | 0.2(3) |  | C23A | C19A | C20A | C21A | -176.1(2) |
| C2A | C3A | C7A | F4A | 172.3(2) |  | C24A | C21A | C22A | C17A | -176.7(2) |
| C2A | C3A | C7A | F5A | -70.7(3) |  | C25A | C26A | C27A | C28A | 1.2(3) |
| C2A | C3A | C7A | F6A | 46.2(3) |  | C25A | C26A | C27A | C31A | -176.4(6) |
| C3A | C4A | C5A | C6A | -0.1(3) |  | C25A | C26A | C27A | C31B | 175.8(5) |
| C3A | C4A | C5A | C8A | -176.3(2) |  | C26A | C25A | C30A | C29A | -0.1(3) |
| C4A | C3A | C7A | F4A | -9.7(3) |  | C26A | C25A | B1A | C1A | 48.3(2) |
| C4A | C3A | C7A | F5A | 107.4(3) |  | C26A | C25A | B1A | C9A | -71.7(2) |
| C4A | C3A | C7A | F6A | -135.8(2) |  | C26A | C25A | B1A | C17A | 168.80(18) |
| C4A | C5A | C6A | C1A | 0.6(3) |  | C26A | C27A | C28A | C29A | -0.7(3) |
| C4A | C5A | C8A | F1A | -150.4(2) |  | C26A | C27A | C31A | F19A | -3.2(13) |
| C4A | C5A | C8A | F2A | -29.0(3) |  | C26A | C27A | C31A | F20A | -123.6(12) |
| C4A | C5A | C8A | F3A | 89.6(3) |  | C26A | C27A | C31A | F21A | 119.3(10) |
| C6A | C1A | C2A | C3A | 1.3(3) |  | C26A | C27A | C31B | F21B | 145.9(9) |
| C6A | C1A | B1A | C9A | 168.37(18) |  | C26A | C27A | C31B | F20B | -95.0(9) |
| C6A | C1A | B1A | C17A | -73.0(2) |  | C26A | C27A | C31B | F19B | 26.4(12) |
| C6A | C1A | B1A | C25A | 47.6(2) |  | C27A | C28A | C29A | C30A | -0.1(3) |
| C6A | C5A | C8A | F1A | 33.3(3) |  | C27A | C28A | C29A | C32A | -178.5(2) |
| C6A | C5A | C8A | F2A | 154.8(2) |  | C28A | C27A | C31A | F19A | 179.2(9) |
| C6A | C5A | C8A | F3A | -86.6(3) |  | C28A | C27A | C31A | F20A | 58.8(12) |
| C7A | C3A | C4A | C5A | -177.8(2) |  | C28A | C27A | C31A | F21A | -58.3(11) |
| C8A | C5A | C6A | C1A | 176.8(2) |  | C28A | C27A | C31B | F21B | -39.4(11) |
| C9A | C10A | C11A | C12A | -1.1(3) |  | C28A | C27A | C31B | F20B | 79.8(9) |
| C9A | C10A | C11A | C15A | -178.0(2) |  | C28A | C27A | C31B | F19B | -158.8(8) |
| C10A | C9A | C14A | C13A | -1.0(3) |  | C28A | C29A | C30A | C25A | 0.5(3) |
| C10A | C9A | B1A | C1A | 73.7(2) |  | C28A | C29A | C32A | F22A | -37.8(3) |
| C10A | C9A | B1A | C17A | -46.7(2) |  | C28A | C29A | C32A | F23A | 80.3(3) |
| C10A | C9A | B1A | C25A | -167.96(18) |  | C28A | C29A | C32A | F24A | -160.2(2) |
| C10A | C11A | C12A | C13A | -0.1(3) |  | C30A | C25A | C26A | C27A | -0.8(3) |
| C10A | C11A | C15A | F7A | -31.3(3) |  | C30A | C25A | B1A | C1A | -131.3(2) |
| C10A | C11A | C15A | F8A | 87.7(3) |  | C30A | C25A | B1A | C9A | 108.7(2) |
| C10A | C11A | C15A | F9A | -151.2(2) |  | C30A | C25A | B1A | C17A | -10.8(3) |
| C11A | C12A | C13A | C14A | 0.6(3) |  | C30A | C29A | C32A | F22A | 143.7(2) |
| C11A | C12A | C13A | C16A | -175.9(2) |  | C30A | C29A | C32A | F23A | -98.2(3) |
| C12A | C11A | C15A | F7A | 151.8(2) |  | C30A | C29A | C32A | F24A | 21.3(3) |
| C12A | C11A | C15A | F8A | -89.2(3) |  | C31A | C27A | C28A | C29A | 176.9(6) |
| C12A | C11A | C15A | F9A | 31.9(3) |  | C32A | C29A | C30A | C25A | 178.9(2) |
| C12A | C13A | C14A | C9A | -0.1(3) |  | B1A | C1A | C2A | C3A | -179.3(2) |
| C12A | C13A | C16A | F10A | -154.1(2) |  | B1A | C1A | C6A | C5A | 179.37(19) |
| C12A | C13A | C16A | F11A | 85.7(3) |  | B1A | C9A | C10A | C11A | -177.0(2) |
| C12A | C13A | C16A | F12A | -33.8(3) |  | B1A | C9A | C14A | C13A | 177.5(2) |
| C14A | C9A | C10A | C11A | 1.6(3) |  | B1A | C17A | C18A | C19A | -179.83(19) |
| C14A | C9A | B1A | C1A | -104.8(2) |  | B1A | C17A | C22A | C21A | -179.71(19) |
| C14A | C9A | B1A | C17A | 134.9(2) |  | B1A | C25A | C26A | C27A | 179.62(19) |
| C14A | C9A | B1A | C25A | 13.6(3) |  | B1A | C25A | C30A | C29A | 179.54(19) |
| C14A | C13A | C16A | F10A | 29.3(3) |  | N1 | C3 | C4 | C5 | -0.4(5) |
| C14A | C13A | C16A | F11A | -90.9(3) |  | N1 | C7 | C8 | N2 | -2.1(4) |
| C14A | C13A | C16A | F12A | 149.6(2) |  | N1 | C7 | C8 | C9 | 178.3(3) |
| C15A | C11A | C12A | C13A | 176.8(2) |  | N2 | C8 | C9 | C10 | 0.0(5) |
| C16A | C13A | C14A | C9A | 176.5(2) |  | C1 | C5 | C6 | C7 | 178.8(3) |
| C17A | C18A | C19A | C20A | -0.5(3) |  | C2 | C10 | C11 | C12 | 178.3(4) |
| C17A | C18A | C19A | C23A | 176.1(2) |  | C3 | N1 | C7 | C6 | -0.2(4) |
| C18A | C17A | C22A | C21A | 0.4(3) |  | C3 | N1 | C7 | C8 | -178.0(3) |
| C18A | C17A | B1A | C1A | 15.5(3) |  | C3 | C4 | C5 | C1 | -179.1(4) |
| C18A | C17A | B1A | C9A | 135.5(2) |  | C3 | C4 | C5 | C6 | 1.1(5) |
| C18A | C17A | B1A | C25A | -102.9(2) |  | C4 | C5 | C6 | C7 | -1.4(5) |
| C18A | C19A | C20A | C21A | 0.4(3) |  | C5 | C6 | C7 | N1 | 0.9(4) |
| C18A | C19A | C23A | F13A | 27.4(3) |  | C5 | C6 | C7 | C8 | 178.5(3) |
| C18A | C19A | C23A | F14A | 148.7(2) |  | C6 | C7 | C8 | N2 | -179.7(3) |
| C18A | C19A | C23A | F15A | -91.5(3) |  | C6 | C7 | C8 | C9 | 0.6(5) |
| C19A | C20A | C21A | C22A | 0.0(3) |  | C7 | N1 | C3 | C4 | 0.0(5) |
| C19A | C20A | C21A | C24A | 176.2(2) |  | C7 | C8 | C9 | C10 | 179.6(3) |
| C20A | C19A | C23A | F13A | -156.0(2) |  | C8 | N2 | C12 | C11 | -0.2(6) |
| C20A | C19A | C23A | F14A | -34.7(3) |  | C8 | C9 | C10 | C2 | -178.7(4) |
| C20A | C19A | C23A | F15A | 85.1(3) |  | C8 | C9 | C10 | C11 | 0.8(5) |
| C20A | C21A | C22A | C17A | -0.5(3) |  | C9 | C10 | C11 | C12 | -1.3(6) |
| C20A | C21A | C24A | F16A | 141.4(2) |  | C10 | C11 | C12 | N2 | 1.0(7) |
| C20A | C21A | C24A | F17A | 20.1(3) |  | C12 | N2 | C8 | C7 | -180.0(3) |
| C20A | C21A | C24A | F18A | -99.1(3) |  | C12 | N2 | C8 | C9 | -0.3(5) |
| C22A | C17A | C18A | C19A | 0.0(3) |  | C31B | C27A | C28A | C29A | -175.4(5) |

Table 7 Hydrogen Atom Coordinates (Å×104) and Isotropic Displacement Parameters (Å2×103) for c041219\_2\_1.

| Atom | *x* | *y* | *z* | U(eq) |
| H2A | 4262.71 | 2782.66 | 4205.89 | 30 |
| H4A | 7030.26 | 4442.31 | 4124.31 | 33 |
| H6A | 7132.79 | 3559.8 | 1974.64 | 29 |
| H10A | 2081.2 | 2842.09 | 3393.18 | 32 |
| H12A | 1370.79 | 392.49 | 4906.18 | 36 |
| H14A | 5319.27 | 620.71 | 3233.73 | 31 |
| H18A | 4517.79 | 4391.07 | 1721.8 | 29 |
| H20A | 1526.13 | 4722.83 | 159.78 | 34 |
| H22A | 2998.59 | 2234.49 | 1621.31 | 32 |
| H26A | 7510.6 | 1412.35 | 3032.78 | 31 |
| H28A | 9954.78 | 540.82 | 890.49 | 35 |
| H30A | 5911.56 | 2231.81 | 776.33 | 29 |
| H1 | 5397.81 | 4312.11 | 7344.45 | 74 |
| H1A | 11185.75 | 3130.37 | 5810.67 | 119 |
| H1B | 11043.32 | 2283.22 | 6628.76 | 119 |
| H1C | 11493.72 | 3165.77 | 6729.86 | 119 |
| H2B | 5480.25 | 63.47 | 7993.73 | 139 |
| H2C | 3744.24 | 139.25 | 8438.74 | 139 |
| H2D | 4974.95 | 40.97 | 9014.4 | 139 |
| H3 | 6919.13 | 5258.64 | 6754.55 | 84 |
| H4 | 9447.6 | 4674.55 | 6316.96 | 84 |
| H6 | 8546.41 | 2131.42 | 7159.89 | 61 |
| H9 | 6692.3 | 1373.18 | 7732.91 | 64 |
| H11 | 2211.05 | 1632.6 | 8653.27 | 95 |
| H12 | 2060.25 | 3166.57 | 8363.73 | 100 |

Table 8 Atomic Occupancy for c041219\_2\_1.

| Atom | *Occupancy* |  | Atom | *Occupancy* |  | Atom | *Occupancy* |
| F19A | 0.48(3) |  | F20A | 0.48(3) |  | F21A | 0.48(3) |
| C31A | 0.48(3) |  | C31B | 0.52(3) |  | F21B | 0.52(3) |
| F20B | 0.52(3) |  | F19B | 0.52(3) |  |  |  |

Experimental

Single crystals of C44H25BF24N2
[c041219\_2\_1]
were
[].
A suitable crystal was selected and
[]
on a
XtaLAB Synergy, Dualflex, Pilatus 300K
diffractometer. The crystal was kept at 100.0(1) K during data collection.
Using Olex2 [1], the structure was solved with the
SHELXT
[2] structure solution program using
Intrinsic Phasing
and refined with the
SHELXL
[3] refinement package using
Least Squares
minimisation.

1. Dolomanov, O.V., Bourhis, L.J., Gildea, R.J, Howard, J.A.K. & Puschmann, H.
   (2009), J. Appl. Cryst. 42, 339-341.
2. Sheldrick, G.M. (2015). Acta Cryst. A71, 3-8.
3. Sheldrick, G.M. (2015). Acta Cryst. C71, 3-8.

Crystal structure determination of
[c041219\_2\_1]

**Crystal Data**
for C44H25BF24N2 (*M*=1048.47 g/mol):
triclinic, space group P-1 (no. 2),
*a* = 9.34770(10) Å, *b* = 15.6602(2) Å, *c* = 16.1982(2) Å, *α* = 73.8830(10)°, *β* = 76.1990(10)°, *γ* = 75.6590(10)°,
*V*= 2169.99(5) Å3,
*Z* = 2,
*T* = 100.0(1) K,
μ(Cu Kα) = 1.503 mm-1,
*Dcalc* = 1.605 g/cm3,
60128 reflections measured (5.776° ≤ 2Θ ≤ 159.598°),
9225 unique (*R*int = 0.0519, Rsigma = 0.0296) which were used in all calculations.
The final *R*1 was 0.0585
(I > 2σ(I)) and *wR*2 was 0.1742 (all data).

Refinement model description

Number of restraints - 358,
number of constraints - unknown.

Details:

```
1. Fixed Uiso
```

This report has been created with Olex2, compiled on
Nov 21 2019 18:26:39 for OlexSys. Please
let us know
if there are any errors or if you would like to have additional features.
