# Supplementary material for: London Dispersion versus Intramolecular Hydrogen Bond in Bis‐Pyridines: How Accurate Is DFT for Competing Noncovalent Interactions in the Condensed Phase?
Source: Chemistry. 2025 Oct 23;31(66):e02745. doi: 10.1002/chem.202502745 (PMC12648470; doi:10.1002/chem.202502745)

checkCIF/PLATON report

Structure factors have been supplied for datablock(s) c041219_2_1

THIS REPORT IS FOR GUIDANCE ONLY. IF USED AS PART OF A REVIEW PROCEDURE FOR PUBLICATION, IT SHOULD NOT REPLACE THE EXPERTISE OF AN EXPERIENCED CRYSTALLOGRAPHIC REFEREE.

No syntax errors found. CIF dictionary Interpreting this report

Datablock: c041219_2_1

```
Bond precision:  C-C = 0.0040 A          Wavelength=1.54184

Cell:          a=9.3477(1)       b=15.6602(2)     c=16.1982(2)
               alpha=73.883(1)   beta=76.199(1)   gamma=75.659(1)
Temperature:   100 K

                Calculated                 Reported
Volume          2169.99(5)                 2169.99(5)
Space group     P -1                       P -1
Hall group      -P 1                       -P 1
Moiety formula  C32 H12 B F24, C12 H13 N2  C32 H12 B F24, C12 H13 N2
Sum formula     C44 H25 B F24 N2           C44 H25 B F24 N2
Mr              1048.47                    1048.47
Dx,g cm-3       1.605                      1.605
Z               2                          2
Mu (mm-1)       1.503                      1.503
F000            1048.0                     1048.0
F000'           1053.10
h,k,lmax        11,19,20                   11,19,20
Nref            9452                       9225
Tmin,Tmax       0.904,0.946                0.738,1.000
Tmin'           0.799

Correction method= # Reported T Limits: Tmin=0.738 Tmax=1.000
AbsCorr = GAUSSIAN

Data completeness= 0.976          Theta(max)= 79.799

R(reflections)= 0.0585( 7622)     wR2(reflections)= 0.1742( 9225)

S = 1.085                   Npar= 679
```

The following ALERTS were generated. Each ALERT has the format
test-name_ALERT_alert-type_alert-level.
Click on the hyperlinks for more details of the test.

Alert level B

```
PLAT097_ALERT_2_B Large Reported Max.  (Positive) Residual Density       1.19 eA-3
```

Alert level C

```
DIFMX02_ALERT_1_C  The maximum difference density is > 0.1*ZMAX*0.75
            The relevant atom site should be identified.
PLAT094_ALERT_2_C Ratio of Maximum / Minimum Residual Density ....       2.68 Report
PLAT230_ALERT_2_C Hirshfeld Test Diff for    N1        --C7       .       6.5 s.u.
PLAT230_ALERT_2_C Hirshfeld Test Diff for    C6        --C7       .       5.5 s.u.
PLAT340_ALERT_3_C Low Bond Precision on  C-C Bonds ..............    0.00402 Ang.
PLAT906_ALERT_3_C Large K Value in the Analysis of Variance ......      2.401 Check
PLAT911_ALERT_3_C Missing FCF Refl Between Thmin & STh/L=    0.600          4 Report
```

Alert level G

```
PLAT002_ALERT_2_G Number of Distance or Angle Restraints on AtSite          9 Note
PLAT003_ALERT_2_G Number of Uiso or Uij Restrained non-H Atoms ...          8 Report
PLAT007_ALERT_5_G Number of Unrefined Donor-H Atoms .............          1 Report
PLAT154_ALERT_1_G The s.u.'s on the Cell Angles are Equal ..(Note)      0.001 Degree
PLAT176_ALERT_4_G The CIF-Embedded .res File Contains SADI Records          3 Report
PLAT178_ALERT_4_G The CIF-Embedded .res File Contains SIMU Records          1 Report
PLAT186_ALERT_4_G The CIF-Embedded .res File Contains ISOR Records          1 Report
PLAT187_ALERT_4_G The CIF-Embedded .res File Contains RIGU Records          2 Report
PLAT242_ALERT_2_G Low    MainMol Ueq as Compared to Neighbors of        C7A Check
PLAT242_ALERT_2_G Low    MainMol Ueq as Compared to Neighbors of        C8A Check
PLAT242_ALERT_2_G Low    MainMol Ueq as Compared to Neighbors of       C15A Check
PLAT242_ALERT_2_G Low    MainMol Ueq as Compared to Neighbors of       C16A Check
PLAT242_ALERT_2_G Low    MainMol Ueq as Compared to Neighbors of       C23A Check
PLAT242_ALERT_2_G Low    MainMol Ueq as Compared to Neighbors of       C24A Check
PLAT242_ALERT_2_G Low    MainMol Ueq as Compared to Neighbors of       C32A Check
PLAT301_ALERT_3_G Main Residue  Disorder ..............(Resd  1  )         7% Note
PLAT860_ALERT_3_G Number of Least-Squares Restraints .............        358 Note
PLAT912_ALERT_4_G Missing # of FCF Reflections Above STh/L=  0.600        224 Note
PLAT978_ALERT_2_G Number C-C Bonds with Positive Residual Density.          1 Info
PLAT992_ALERT_5_G Repd & Actual _reflns_number_gt Values Differ by          1 Check
```

```
   0 ALERT level A = Most likely a serious problem - resolve or explain
   1 ALERT level B = A potentially serious problem, consider carefully
   7 ALERT level C = Check. Ensure it is not caused by an omission or oversight
  20 ALERT level G = General information/check it is not something unexpected

   2 ALERT type 1 CIF construction/syntax error, inconsistent or missing data
  14 ALERT type 2 Indicator that the structure model may be wrong or deficient
   5 ALERT type 3 Indicator that the structure quality may be low
   5 ALERT type 4 Improvement, methodology, query or suggestion
   2 ALERT type 5 Informative message, check
```

It is advisable to attempt to resolve as many as possible of the alerts in all categories. Often the minor alerts point to easily fixed oversights, errors and omissions in your CIF or refinement strategy, so attention to these fine details can be worthwhile. In order to resolve some of the more serious problems it may be necessary to carry out additional measurements or structure refinements. However, the purpose of your study may justify the reported deviations and the more serious of these should normally be commented upon in the discussion or experimental section of a paper or in the "special_details" fields of the CIF. checkCIF was carefully designed to identify outliers and unusual parameters, but every test has its limitations and alerts that are not important in a particular case may appear. Conversely, the absence of alerts does not guarantee there are no aspects of the results needing attention. It is up to the individual to critically assess their own results and, if necessary, seek expert advice.

Publication of your CIF in IUCr journals

A basic structural check has been run on your CIF. These basic checks will be run on all CIFs submitted for publication in IUCr journals (*Acta Crystallographica*, *Journal of Applied Crystallography*, *Journal of Synchrotron Radiation*); however, if you intend to submit to *Acta Crystallographica Section C* or *E* or *IUCrData*, you should make sure that full publication checks are run on the final version of your CIF prior to submission.

Publication of your CIF in other journals

Please refer to the *Notes for Authors* of the relevant journal for any special instructions relating to CIF submission.

PLATON version of 22/12/2019; check.def file version of 13/12/2019

Datablock c041219_2_1 - ellipsoid plot

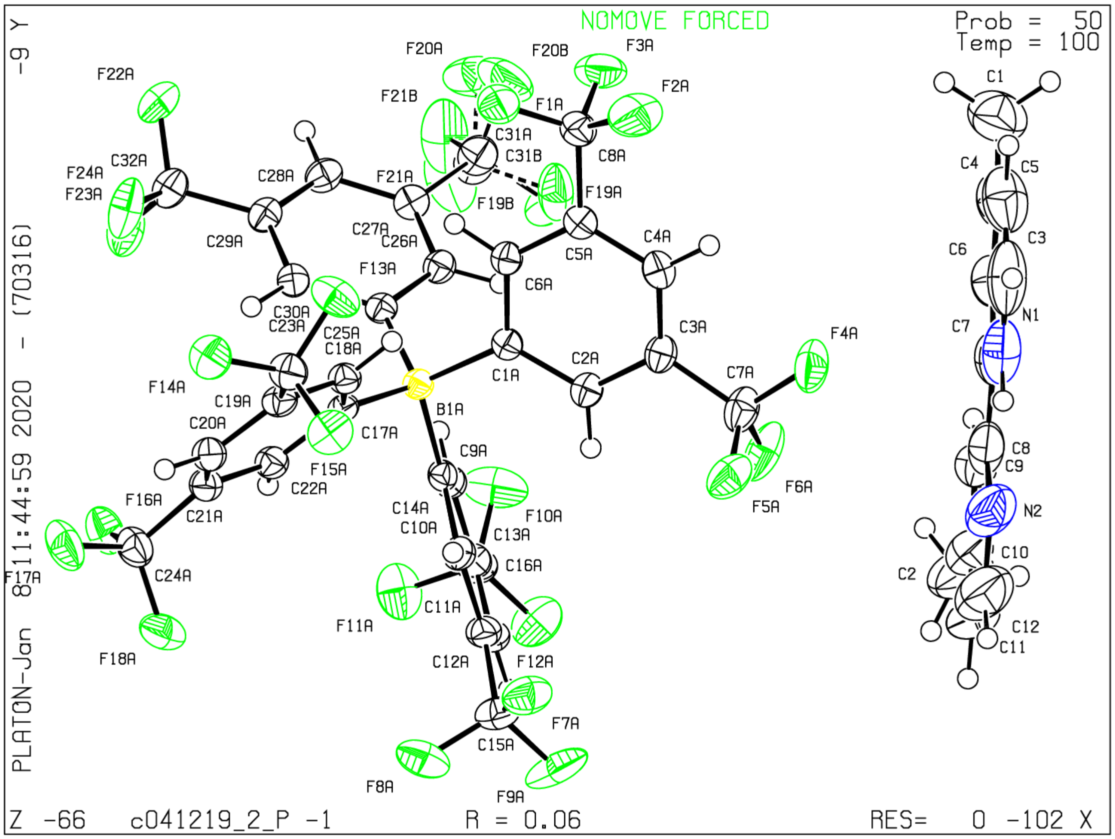

Supplement: Supplementary file 1 — Supporting Information [file CHEM-31-e02745-s002.zip › Crystal_structures/1b/checkcif.pdf]
