# Supplementary material for: London Dispersion versus Intramolecular Hydrogen Bond in Bis‐Pyridines: How Accurate Is DFT for Competing Noncovalent Interactions in the Condensed Phase?
Source: Chemistry. 2025 Oct 23;31(66):e02745. doi: 10.1002/chem.202502745 (PMC12648470; doi:10.1002/chem.202502745)
Supplement: Supplementary file 1 — Supporting Information [file CHEM-31-e02745-s002.zip › Crystal_structures/2b/c030620_1_1_tables.html]

c030620\_1\_1


# c030620\_1\_1

Table 1 Crystal data and structure refinement for c030620\_1\_1.

| Identification code | c030620\_1\_1 |
| Empirical formula | C45H26BCl3F24N2 |
| Formula weight | 1167.84 |
| Temperature/K | 100.0(1) |
| Crystal system | triclinic |
| Space group | P-1 |
| a/Å | 13.3366(15) |
| b/Å | 14.1573(15) |
| c/Å | 15.5099(15) |
| α/° | 66.246(3) |
| β/° | 89.877(4) |
| γ/° | 64.036(3) |
| Volume/Å3 | 2355.2(4) |
| Z | 2 |
| ρcalcg/cm3 | 1.647 |
| μ/mm‑1 | 0.327 |
| F(000) | 1164.0 |
| Crystal size/mm3 | 0.17 × 0.15 × 0.04 |
| Radiation | MoKα (λ = 0.71073) |
| 2Θ range for data collection/° | 2.936 to 55.254 |
| Index ranges | -17 ≤ h ≤ 17, -18 ≤ k ≤ 18, -20 ≤ l ≤ 20 |
| Reflections collected | 35730 |
| Independent reflections | 10885 [Rint = 0.0569, Rsigma = 0.0717] |
| Data/restraints/parameters | 10885/545/808 |
| Goodness-of-fit on F2 | 0.973 |
| Final R indexes [I>=2σ (I)] | R1 = 0.0469, wR2 = 0.1032 |
| Final R indexes [all data] | R1 = 0.0929, wR2 = 0.1198 |
| Largest diff. peak/hole / e Å-3 | 0.39/-0.50 |

Table 2 Fractional Atomic Coordinates (×104) and Equivalent Isotropic Displacement Parameters (Å2×103) for c030620\_1\_1. Ueq is defined as 1/3 of of the trace of the orthogonalised UIJ tensor.

| Atom | *x* | *y* | *z* | U(eq) |
| --- | --- | --- | --- | --- |
| Cl1C | 4325.8(6) | 1972.3(6) | 7469.9(5) | 36.78(16) |
| Cl2C | 6672.1(6) | 277.8(7) | 8341.7(5) | 55.2(2) |
| Cl3C | 5240.4(5) | 1380.4(6) | 9415.3(4) | 32.14(16) |
| C1C | 5294(2) | 858(2) | 8545.0(16) | 27.3(5) |
| F1 | -779(10) | 8835(10) | 3479(5) | 59(2) |
| F2 | -1257(5) | 9032(6) | 4715(4) | 83(2) |
| F3 | -71(6) | 9573(5) | 4072(5) | 60.9(17) |
| F4 | 1049.6(11) | 4613.4(12) | 4644.8(9) | 27.3(3) |
| F5 | 2784.1(11) | 3730.8(12) | 5408.9(10) | 31.5(3) |
| F6 | 1469.0(12) | 3671.0(12) | 6192.6(9) | 29.3(3) |
| F7 | 3214(6) | 9727(4) | 8035(3) | 48.4(14) |
| F8 | 4457(5) | 7965(5) | 8889(5) | 30.4(11) |
| F9 | 4932(9) | 9158(14) | 7841(17) | 41.2(12) |
| F10 | 3895(4) | 10293(4) | 4387(7) | 27.6(13) |
| F11 | 2995(4) | 9548(4) | 3965(3) | 46.7(14) |
| F12 | 4837(3) | 8571(3) | 4471(3) | 37.2(12) |
| F13 | -1188(7) | 8296(8) | 7986(4) | 35.0(14) |
| F14 | -606(6) | 8034(7) | 9385(4) | 60.1(17) |
| F15 | -185(4) | 9059(4) | 8101(4) | 61.8(16) |
| F16 | 2945.3(14) | 2863.1(13) | 10341.4(10) | 40.9(4) |
| F17 | 3145.3(14) | 3718.6(14) | 11139.0(10) | 45.7(4) |
| F18 | 1555.5(14) | 3752.7(16) | 10904.9(12) | 61.5(5) |
| F19 | 6706.6(14) | 4137.5(14) | 5636.1(10) | 44.6(4) |
| F20 | 5657.1(13) | 3343.5(15) | 5727.5(11) | 43.8(4) |
| F21 | 7331.2(13) | 2329.5(14) | 6611.9(10) | 49.3(4) |
| F22 | 6527.8(11) | 3720.4(13) | 10036.2(9) | 32.0(3) |
| F23 | 7908.9(12) | 3411.7(15) | 9297.3(10) | 42.7(4) |
| F24 | 7436.4(13) | 2070.7(13) | 9987.2(10) | 43.4(4) |
| C1 | 2214.4(17) | 6306.1(19) | 6309.1(14) | 15.9(4) |
| C2 | 1340.3(17) | 7401(2) | 5655.0(15) | 20.0(5) |
| C3 | 583.7(18) | 7530(2) | 4950.9(16) | 22.0(5) |
| C4 | 682.6(18) | 6559(2) | 4862.0(16) | 21.5(5) |
| C5 | 1527.4(18) | 5470.9(19) | 5505.8(15) | 17.4(5) |
| C6 | 2264.4(17) | 5341.9(19) | 6224.6(15) | 16.6(4) |
| C7 | -352(2) | 8719(2) | 4301.7(18) | 33.3(6) |
| C8 | 1700.7(19) | 4385(2) | 5435.2(16) | 21.7(5) |
| C9 | 3294.5(17) | 7290.2(18) | 6835.4(15) | 15.7(4) |
| C10 | 3514.5(18) | 7662(2) | 7493.5(16) | 20.3(5) |
| C11 | 3832(2) | 8533(2) | 7230.0(16) | 23.8(5) |
| C12 | 3915.2(19) | 9101(2) | 6291.4(16) | 22.5(5) |
| C13 | 3704.6(18) | 8751.9(19) | 5629.7(15) | 19.6(5) |
| C14 | 3410.5(17) | 7859.9(19) | 5900.6(15) | 17.5(5) |
| C15 | 4120(10) | 8829(8) | 7992(6) | 33.1(10) |
| C16 | 3814(2) | 9309(2) | 4614.4(18) | 30.1(6) |
| C17 | 2398.6(17) | 6041.8(19) | 8060.2(15) | 16.1(4) |
| C18 | 2668.9(18) | 4992(2) | 8869.7(15) | 18.7(5) |
| C19 | 2011.4(19) | 4938(2) | 9572.9(15) | 22.1(5) |
| C20 | 1043.6(19) | 5928(2) | 9482.1(16) | 24.2(5) |
| C21 | 749.4(18) | 6980(2) | 8684.2(16) | 22.5(5) |
| C22 | 1424.0(18) | 7033(2) | 8000.3(15) | 18.3(5) |
| C23 | 2415(2) | 3820(2) | 10475.6(17) | 30.8(6) |
| C24 | -283(2) | 8071(2) | 8557.5(19) | 31.8(6) |
| C25 | 4347.5(17) | 5052.1(18) | 7454.3(14) | 15.0(4) |
| C26 | 4823.5(17) | 4615.9(19) | 6802.3(15) | 15.6(4) |
| C27 | 5945.8(18) | 3740.0(19) | 7020.1(15) | 18.4(5) |
| C28 | 6646.6(18) | 3265.5(19) | 7893.9(15) | 19.6(5) |
| C29 | 6212.9(18) | 3699.1(19) | 8550.9(15) | 17.5(5) |
| C30 | 5090.0(17) | 4568.4(19) | 8332.9(15) | 16.8(4) |
| C31 | 6410.9(19) | 3371(2) | 6260.2(16) | 25.1(5) |
| C32 | 6997.8(19) | 3231(2) | 9463.8(16) | 23.9(5) |
| B1 | 3068(2) | 6158(2) | 7168.7(17) | 15.9(5) |
| N1B | 1492.8(15) | 4996.5(17) | 2617.3(13) | 19.7(4) |
| N2B | 85.0(16) | 7224.2(17) | 1830.6(14) | 25.0(4) |
| C1B | 3523(2) | 3747(2) | 3293.9(18) | 30.2(6) |
| C2B | 2353.6(19) | 3904(2) | 3088.5(16) | 23.1(5) |
| C3B | 2092(2) | 2998(2) | 3351.5(17) | 30.8(6) |
| C4B | 982(2) | 3243(2) | 3121.0(17) | 31.2(6) |
| C5B | 120(2) | 4388(2) | 2626.2(17) | 27.2(6) |
| C6B | 386.3(19) | 5287(2) | 2376.4(16) | 21.2(5) |
| C7B | -395.5(19) | 6555(2) | 1868.2(16) | 22.2(5) |
| C8B | -1527(2) | 7013(2) | 1460.4(17) | 30.7(6) |
| C9B | -2168(2) | 8215(2) | 984.6(18) | 36.6(7) |
| C10B | -1695(2) | 8911(2) | 936.2(18) | 32.6(6) |
| C11B | -557(2) | 8393(2) | 1371.9(17) | 29.7(6) |
| C12B | 18(2) | 9101(2) | 1344(2) | 45.9(7) |
| F13A | -1265(11) | 8011(15) | 8594(15) | 35.0(14) |
| F14A | -257(19) | 8480(20) | 9214(15) | 60.1(17) |
| F15A | -580(20) | 9057(16) | 7701(12) | 61.8(16) |
| F13B | -812(14) | 7815(14) | 9305(8) | 60.1(17) |
| F15B | -1042(13) | 8531(16) | 7795(7) | 45(4) |
| F14B | 30(9) | 8786(9) | 8619(7) | 58(3) |
| F2A | -1334(19) | 8670(30) | 4420(30) | 83(2) |
| F3A | -460(20) | 9570(20) | 4510(20) | 50(2) |
| F1A | -350(30) | 9050(30) | 3385(11) | 46(7) |
| F2B | -1044(15) | 8702(18) | 3727(10) | 64(5) |
| F3B | -938(8) | 9290(9) | 4773(7) | 50(2) |
| F1B | 83(10) | 9389(10) | 3731(8) | 49(3) |
| F11A | 3370(20) | 9100(20) | 3980(20) | 46.7(14) |
| F12A | 4963(11) | 8761(18) | 4638(18) | 37.2(12) |
| F10A | 3478(16) | 10444(11) | 4250(20) | 27.6(13) |
| F11B | 4415(6) | 8597(8) | 4265(7) | 37.2(12) |
| F12B | 4188(11) | 10087(9) | 4479(17) | 27.6(13) |
| F10B | 2736(7) | 9986(9) | 4029(8) | 46.7(14) |
| C15A | 3860(20) | 9000(20) | 7937(15) | 33.1(10) |
| F7A | 2868(19) | 9480(20) | 8180(19) | 79(5) |
| F9A | 4203(18) | 9805(17) | 7574(13) | 48.4(14) |
| F8A | 4620(20) | 8140(20) | 8750(20) | 41.2(12) |
| F7B | 4073(12) | 8300(12) | 8807(11) | 79(5) |
| C15B | 4029(18) | 8988(14) | 7900(11) | 33.1(10) |
| F9B | 3218(12) | 10075(9) | 7689(7) | 48.4(14) |
| F8B | 5016(17) | 9020(30) | 7890(30) | 41.2(12) |

Table 3 Anisotropic Displacement Parameters (Å2×103) for c030620\_1\_1. The Anisotropic displacement factor exponent takes the form: -2π2[h2a\*2U11+2hka\*b\*U12+…].

| Atom | U11 | U22 | U33 | U23 | U13 | U12 |
| --- | --- | --- | --- | --- | --- | --- |
| Cl1C | 37.8(4) | 33.1(4) | 29.2(3) | -9.8(3) | 2.1(3) | -12.3(3) |
| Cl2C | 34.8(4) | 61.9(5) | 44.8(4) | -27.5(4) | 12.0(3) | -0.5(4) |
| Cl3C | 39.0(4) | 39.2(4) | 32.7(3) | -23.0(3) | 14.9(3) | -24.1(3) |
| C1C | 33.1(14) | 24.8(13) | 24.8(13) | -11.2(11) | 9.6(10) | -14.3(11) |
| F1 | 74(7) | 40(4) | 35(3) | -5(3) | -27(3) | -15(4) |
| F2 | 35(3) | 61(5) | 71(5) | 0(2) | 19(3) | 19(2) |
| F3 | 59(4) | 25(2) | 72(5) | -3(3) | -28(3) | -15(2) |
| F4 | 30.6(8) | 40.5(8) | 23.4(7) | -19.9(7) | 6.2(6) | -22.2(7) |
| F5 | 23.1(7) | 36.9(8) | 44.4(9) | -27.3(7) | 10.8(6) | -14.2(6) |
| F6 | 40.5(8) | 34.5(8) | 25.8(7) | -13.7(6) | 11.1(6) | -28.1(7) |
| F7 | 51.6(13) | 48(3) | 55(4) | -43(3) | 14(3) | -13(2) |
| F8 | 43(2) | 49(2) | 25(2) | -23.6(17) | 13.8(16) | -36.7(18) |
| F9 | 52.4(19) | 56(4) | 47(2) | -32(3) | 17(2) | -44(2) |
| F10 | 20(4) | 17(2) | 29(3) | 3(3) | 2(4) | -7(3) |
| F11 | 66(3) | 69(5) | 17.4(11) | -10(3) | 8(2) | -49(3) |
| F12 | 58(2) | 33.9(11) | 26(3) | -17.8(13) | 27(2) | -23.4(19) |
| F13 | 23(2) | 29(3) | 39(3) | -13(3) | 0(2) | -3.2(15) |
| F14 | 45(4) | 79(4) | 39.1(13) | -41.6(15) | 18.6(17) | -1.2(16) |
| F15 | 44(4) | 34(2) | 133(4) | -52(3) | 49(3) | -26(2) |
| F16 | 62.1(11) | 34.4(9) | 26.1(8) | -7.3(7) | 10.1(7) | -28.7(8) |
| F17 | 54.8(10) | 51.2(10) | 22.7(8) | -10.3(7) | -1.4(7) | -24.2(9) |
| F18 | 43.9(10) | 71.6(13) | 44.4(10) | 1.2(9) | 26.7(8) | -32.4(10) |
| F19 | 58.2(10) | 58.6(11) | 35.3(9) | -26.3(8) | 36.2(8) | -38.0(9) |
| F20 | 37.2(9) | 77.1(12) | 46.5(9) | -50.5(9) | 21.6(7) | -30.2(9) |
| F21 | 43.6(9) | 43.5(10) | 33.4(9) | -22.7(8) | 9.3(7) | 6.3(8) |
| F22 | 27.4(8) | 47.3(9) | 18.0(7) | -17.3(7) | 4.1(6) | -12.7(7) |
| F23 | 28.0(8) | 82.0(12) | 32.5(8) | -27.9(9) | 10.7(6) | -36.0(9) |
| F24 | 46.0(10) | 29.6(9) | 29.5(8) | -2.8(7) | -11.1(7) | -7.1(7) |
| C1 | 15.4(10) | 23.0(12) | 14.5(11) | -9.4(9) | 9.4(8) | -12.4(9) |
| C2 | 17.6(11) | 23.5(12) | 21.6(12) | -11.2(10) | 8.4(9) | -11.1(10) |
| C3 | 18.1(11) | 25.6(12) | 21.5(12) | -9.6(10) | 4.8(9) | -10.7(10) |
| C4 | 15.2(11) | 35.2(14) | 18.8(12) | -12.8(11) | 5.7(9) | -15.1(10) |
| C5 | 17.0(11) | 27.0(12) | 18.3(11) | -13.4(10) | 10.3(9) | -15.6(10) |
| C6 | 12.3(10) | 23.0(12) | 17.1(11) | -9.6(9) | 7.5(8) | -9.8(9) |
| C7 | 28.4(14) | 33.1(15) | 30.7(14) | -12.0(12) | -2.7(11) | -10.5(12) |
| C8 | 22.0(12) | 31.5(13) | 18.7(12) | -12.4(10) | 6.9(9) | -17.5(11) |
| C9 | 12.2(10) | 18.9(11) | 17.4(11) | -10.6(9) | 6.0(8) | -6.3(9) |
| C10 | 22.3(12) | 28.3(13) | 19.8(12) | -13.6(10) | 11.1(9) | -17.1(10) |
| C11 | 27.3(13) | 29.9(13) | 26.8(13) | -18.9(11) | 13.0(10) | -18.1(11) |
| C12 | 24.1(12) | 22.4(12) | 29.6(13) | -14.6(11) | 13.5(10) | -15.6(10) |
| C13 | 17.9(11) | 19.5(12) | 20.8(11) | -8.7(10) | 8.5(9) | -8.8(10) |
| C14 | 17.2(11) | 20.5(12) | 16.8(11) | -9.7(10) | 5.9(9) | -9.4(9) |
| C15 | 46(3) | 39(3) | 35(2) | -24(2) | 23.1(19) | -29(2) |
| C16 | 38.0(14) | 25.9(13) | 26.8(13) | -8.3(11) | 12.0(11) | -18.8(12) |
| C17 | 14.5(10) | 25.0(12) | 17.1(11) | -12.5(10) | 6.3(8) | -13.4(9) |
| C18 | 16.7(11) | 26.9(13) | 17.7(11) | -12.0(10) | 6.3(9) | -12.9(10) |
| C19 | 24.7(12) | 35.3(14) | 15.9(11) | -11.9(11) | 8.1(9) | -21.6(11) |
| C20 | 21.2(12) | 42.8(15) | 23.4(12) | -21.0(12) | 13.5(10) | -21.6(11) |
| C21 | 17.3(11) | 35.4(14) | 25.9(13) | -20.9(11) | 9.9(9) | -14.9(10) |
| C22 | 17.4(11) | 23.8(12) | 19.8(11) | -11.8(10) | 6.5(9) | -13.0(10) |
| C23 | 29.5(13) | 45.4(17) | 22.5(13) | -12.1(12) | 12.6(11) | -24.8(13) |
| C24 | 28.5(13) | 41.6(16) | 40.7(15) | -29.1(13) | 19.6(11) | -19.7(12) |
| C25 | 15.8(10) | 19.5(11) | 16.6(11) | -8.7(9) | 7.6(8) | -13.4(9) |
| C26 | 15.3(10) | 21.6(11) | 14.3(10) | -7.6(9) | 6.2(8) | -12.6(9) |
| C27 | 20.5(11) | 23.8(12) | 18.3(11) | -12.3(10) | 10.3(9) | -14.0(10) |
| C28 | 16.1(11) | 22.8(12) | 19.6(12) | -8.4(10) | 7.8(9) | -10.1(10) |
| C29 | 18.6(11) | 22.1(12) | 15.3(11) | -6.7(9) | 5.2(9) | -13.9(10) |
| C30 | 19.8(11) | 22.7(12) | 16.8(11) | -11.3(9) | 9.8(9) | -15.3(10) |
| C31 | 21.1(12) | 31.2(14) | 22.4(13) | -13.4(11) | 10.0(10) | -10.8(11) |
| C32 | 18.7(12) | 30.2(14) | 21.8(12) | -11.4(11) | 5.3(9) | -11.1(11) |
| B1 | 15.9(12) | 19.8(13) | 13.6(12) | -7.7(10) | 5.9(9) | -9.4(10) |
| N1B | 21.7(10) | 21.9(10) | 20.5(10) | -9.8(9) | 9.8(8) | -14.1(9) |
| N2B | 21.4(10) | 27.8(11) | 26.2(11) | -14.2(9) | 7.2(8) | -10.2(9) |
| C1B | 24.4(13) | 26.7(14) | 32.4(14) | -9.1(12) | 6.6(11) | -10.1(11) |
| C2B | 26.1(12) | 24.8(13) | 20.0(12) | -9.8(10) | 10.6(10) | -13.6(11) |
| C3B | 39.0(15) | 24.5(14) | 27.2(14) | -10.3(11) | 12.9(11) | -15.0(12) |
| C4B | 46.3(16) | 36.9(16) | 29.7(14) | -17.6(12) | 22.2(12) | -32.8(14) |
| C5B | 31.4(13) | 41.1(16) | 25.2(13) | -19.4(12) | 16.7(11) | -26.3(13) |
| C6B | 20.1(11) | 31.2(13) | 19.6(12) | -14.6(10) | 9.9(9) | -15.2(10) |
| C7B | 22.0(12) | 32.4(14) | 18.3(12) | -14.8(11) | 11.7(9) | -14.8(11) |
| C8B | 21.7(12) | 44.8(16) | 27.1(13) | -16.0(12) | 9.3(10) | -17.1(12) |
| C9B | 18.0(12) | 50.8(18) | 29.1(14) | -14.7(13) | 7.9(10) | -9.7(13) |
| C10B | 24.8(13) | 31.2(14) | 27.2(14) | -11.7(12) | 7.7(10) | -2.8(11) |
| C11B | 30.1(14) | 26.9(14) | 27.8(14) | -13.4(11) | 7.2(11) | -9.2(11) |
| C12B | 41.4(17) | 24.3(15) | 58(2) | -12.8(14) | -6.1(14) | -9.5(13) |
| F13A | 23(2) | 29(3) | 39(3) | -13(3) | 0(2) | -3.2(15) |
| F14A | 45(4) | 79(4) | 39.1(13) | -41.6(15) | 18.6(17) | -1.2(16) |
| F15A | 44(4) | 34(2) | 133(4) | -52(3) | 49(3) | -26(2) |
| F13B | 45(4) | 79(4) | 39.1(13) | -41.6(15) | 18.6(17) | -1.2(16) |
| F15B | 35(5) | 46(8) | 32(4) | -18(4) | 12(3) | -2(4) |
| F14B | 37(4) | 42(5) | 106(7) | -51(5) | 3(5) | -11(4) |
| F2A | 35(3) | 61(5) | 71(5) | 0(2) | 19(3) | 19(2) |
| F3A | 32(6) | 43(5) | 47(5) | -25(4) | -5(3) | 8(3) |
| F1A | 45(17) | 45(16) | 29(6) | -11(7) | -1(8) | -12(11) |
| F2B | 52(7) | 40(6) | 69(10) | -8(8) | -44(7) | -12(4) |
| F3B | 32(6) | 43(5) | 47(5) | -25(4) | -5(3) | 8(3) |
| F1B | 55(4) | 33(5) | 40(6) | 1(3) | 1(4) | -21(4) |
| F11A | 66(3) | 69(5) | 17.4(11) | -10(3) | 8(2) | -49(3) |
| F12A | 58(2) | 33.9(11) | 26(3) | -17.8(13) | 27(2) | -23.4(19) |
| F10A | 20(4) | 17(2) | 29(3) | 3(3) | 2(4) | -7(3) |
| F11B | 58(2) | 33.9(11) | 26(3) | -17.8(13) | 27(2) | -23.4(19) |
| F12B | 20(4) | 17(2) | 29(3) | 3(3) | 2(4) | -7(3) |
| F10B | 66(3) | 69(5) | 17.4(11) | -10(3) | 8(2) | -49(3) |
| C15A | 46(3) | 39(3) | 35(2) | -24(2) | 23.1(19) | -29(2) |
| F7A | 143(14) | 118(11) | 44(6) | -51(7) | 45(7) | -104(10) |
| F9A | 51.6(13) | 48(3) | 55(4) | -43(3) | 14(3) | -13(2) |
| F8A | 52.4(19) | 56(4) | 47(2) | -32(3) | 17(2) | -44(2) |
| F7B | 143(14) | 118(11) | 44(6) | -51(7) | 45(7) | -104(10) |
| C15B | 46(3) | 39(3) | 35(2) | -24(2) | 23.1(19) | -29(2) |
| F9B | 51.6(13) | 48(3) | 55(4) | -43(3) | 14(3) | -13(2) |
| F8B | 52.4(19) | 56(4) | 47(2) | -32(3) | 17(2) | -44(2) |

Table 4 Bond Lengths for c030620\_1\_1.

| Atom | Atom | Length/Å |  | Atom | Atom | Length/Å |
| --- | --- | --- | --- | --- | --- | --- |
| Cl1C | C1C | 1.745(2) |  | C13 | C16 | 1.491(3) |
| Cl2C | C1C | 1.742(2) |  | C16 | F11A | 1.335(12) |
| Cl3C | C1C | 1.770(2) |  | C16 | F12A | 1.375(12) |
| F1 | C7 | 1.316(6) |  | C16 | F10A | 1.320(12) |
| F2 | C7 | 1.351(5) |  | C16 | F11B | 1.297(8) |
| F3 | C7 | 1.333(5) |  | C16 | F12B | 1.342(10) |
| F4 | C8 | 1.342(2) |  | C16 | F10B | 1.381(8) |
| F5 | C8 | 1.344(2) |  | C17 | C18 | 1.395(3) |
| F6 | C8 | 1.347(2) |  | C17 | C22 | 1.402(3) |
| F7 | C15 | 1.342(8) |  | C17 | B1 | 1.636(3) |
| F8 | C15 | 1.336(8) |  | C18 | C19 | 1.397(3) |
| F9 | C15 | 1.341(8) |  | C19 | C20 | 1.381(3) |
| F10 | C16 | 1.346(5) |  | C19 | C23 | 1.493(3) |
| F11 | C16 | 1.315(4) |  | C20 | C21 | 1.382(3) |
| F12 | C16 | 1.393(4) |  | C21 | C22 | 1.390(3) |
| F13 | C24 | 1.335(6) |  | C21 | C24 | 1.488(3) |
| F14 | C24 | 1.342(5) |  | C24 | F13A | 1.347(11) |
| F15 | C24 | 1.355(5) |  | C24 | F14A | 1.364(11) |
| F16 | C23 | 1.329(3) |  | C24 | F15A | 1.377(12) |
| F17 | C23 | 1.338(3) |  | C24 | F13B | 1.362(9) |
| F18 | C23 | 1.345(3) |  | C24 | F15B | 1.285(10) |
| F19 | C31 | 1.340(3) |  | C24 | F14B | 1.288(8) |
| F20 | C31 | 1.326(3) |  | C25 | C26 | 1.402(3) |
| F21 | C31 | 1.322(3) |  | C25 | C30 | 1.402(3) |
| F22 | C32 | 1.332(2) |  | C25 | B1 | 1.632(3) |
| F23 | C32 | 1.351(3) |  | C26 | C27 | 1.390(3) |
| F24 | C32 | 1.337(3) |  | C27 | C28 | 1.372(3) |
| C1 | C2 | 1.396(3) |  | C27 | C31 | 1.502(3) |
| C1 | C6 | 1.396(3) |  | C28 | C29 | 1.393(3) |
| C1 | B1 | 1.641(3) |  | C29 | C30 | 1.387(3) |
| C2 | C3 | 1.388(3) |  | C29 | C32 | 1.478(3) |
| C3 | C4 | 1.387(3) |  | N1B | C2B | 1.335(3) |
| C3 | C7 | 1.485(3) |  | N1B | C6B | 1.354(3) |
| C4 | C5 | 1.373(3) |  | N2B | C7B | 1.342(3) |
| C5 | C6 | 1.388(3) |  | N2B | C11B | 1.338(3) |
| C5 | C8 | 1.501(3) |  | C1B | C2B | 1.491(3) |
| C7 | F2A | 1.352(13) |  | C2B | C3B | 1.380(3) |
| C7 | F3A | 1.314(12) |  | C3B | C4B | 1.376(3) |
| C7 | F1A | 1.306(12) |  | C4B | C5B | 1.382(3) |
| C7 | F2B | 1.299(10) |  | C5B | C6B | 1.375(3) |
| C7 | F3B | 1.311(8) |  | C6B | C7B | 1.468(3) |
| C7 | F1B | 1.350(8) |  | C7B | C8B | 1.384(3) |
| C9 | C10 | 1.405(3) |  | C8B | C9B | 1.374(4) |
| C9 | C14 | 1.394(3) |  | C9B | C10B | 1.364(4) |
| C9 | B1 | 1.637(3) |  | C10B | C11B | 1.388(3) |
| C10 | C11 | 1.386(3) |  | C11B | C12B | 1.494(4) |
| C11 | C12 | 1.383(3) |  | C15A | F7A | 1.330(14) |
| C11 | C15 | 1.504(8) |  | C15A | F9A | 1.326(14) |
| C11 | C15A | 1.500(15) |  | C15A | F8A | 1.332(14) |
| C11 | C15B | 1.500(13) |  | F7B | C15B | 1.334(13) |
| C12 | C13 | 1.381(3) |  | C15B | F9B | 1.335(12) |
| C13 | C14 | 1.389(3) |  | C15B | F8B | 1.336(13) |

Table 5 Bond Angles for c030620\_1\_1.

| Atom | Atom | Atom | Angle/˚ |  | Atom | Atom | Atom | Angle/˚ |
| --- | --- | --- | --- | --- | --- | --- | --- | --- |
| Cl1C | C1C | Cl3C | 110.90(13) |  | C22 | C21 | C24 | 119.4(2) |
| Cl2C | C1C | Cl1C | 110.50(13) |  | C21 | C22 | C17 | 122.8(2) |
| Cl2C | C1C | Cl3C | 110.48(13) |  | F16 | C23 | F17 | 106.4(2) |
| C2 | C1 | C6 | 115.64(19) |  | F16 | C23 | F18 | 107.0(2) |
| C2 | C1 | B1 | 121.97(19) |  | F16 | C23 | C19 | 113.64(19) |
| C6 | C1 | B1 | 122.25(19) |  | F17 | C23 | F18 | 104.98(19) |
| C3 | C2 | C1 | 122.4(2) |  | F17 | C23 | C19 | 112.1(2) |
| C2 | C3 | C7 | 119.4(2) |  | F18 | C23 | C19 | 112.1(2) |
| C4 | C3 | C2 | 120.6(2) |  | F13 | C24 | F14 | 105.8(4) |
| C4 | C3 | C7 | 120.0(2) |  | F13 | C24 | F15 | 102.6(4) |
| C5 | C4 | C3 | 118.0(2) |  | F13 | C24 | C21 | 112.1(5) |
| C4 | C5 | C6 | 121.3(2) |  | F14 | C24 | F15 | 108.1(4) |
| C4 | C5 | C8 | 120.76(19) |  | F14 | C24 | C21 | 113.6(4) |
| C6 | C5 | C8 | 117.9(2) |  | F15 | C24 | C21 | 113.8(3) |
| C5 | C6 | C1 | 122.0(2) |  | F13A | C24 | C21 | 115.7(7) |
| F1 | C7 | F2 | 104.2(5) |  | F13A | C24 | F14A | 102.3(9) |
| F1 | C7 | F3 | 106.0(5) |  | F13A | C24 | F15A | 101.9(10) |
| F1 | C7 | C3 | 115.3(5) |  | F14A | C24 | C21 | 116.4(10) |
| F2 | C7 | C3 | 111.4(3) |  | F14A | C24 | F15A | 101.5(10) |
| F3 | C7 | F2 | 104.9(4) |  | F15A | C24 | C21 | 116.7(10) |
| F3 | C7 | C3 | 114.2(4) |  | F13B | C24 | C21 | 109.4(8) |
| F2A | C7 | C3 | 106.1(15) |  | F15B | C24 | C21 | 113.8(9) |
| F3A | C7 | C3 | 115.8(12) |  | F15B | C24 | F13B | 105.2(8) |
| F3A | C7 | F2A | 104.8(11) |  | F15B | C24 | F14B | 113.6(8) |
| F1A | C7 | C3 | 114.6(14) |  | F14B | C24 | C21 | 108.9(5) |
| F1A | C7 | F2A | 105.8(12) |  | F14B | C24 | F13B | 105.5(6) |
| F1A | C7 | F3A | 108.7(12) |  | C26 | C25 | C30 | 115.46(19) |
| F2B | C7 | C3 | 112.9(9) |  | C26 | C25 | B1 | 122.54(18) |
| F2B | C7 | F3B | 109.4(9) |  | C30 | C25 | B1 | 121.59(17) |
| F2B | C7 | F1B | 106.3(7) |  | C27 | C26 | C25 | 122.38(19) |
| F3B | C7 | C3 | 112.6(5) |  | C26 | C27 | C31 | 119.04(19) |
| F3B | C7 | F1B | 105.0(6) |  | C28 | C27 | C26 | 120.88(19) |
| F1B | C7 | C3 | 110.1(6) |  | C28 | C27 | C31 | 119.96(19) |
| F4 | C8 | F5 | 106.05(16) |  | C27 | C28 | C29 | 118.4(2) |
| F4 | C8 | F6 | 106.28(17) |  | C28 | C29 | C32 | 117.76(19) |
| F4 | C8 | C5 | 113.36(19) |  | C30 | C29 | C28 | 120.53(19) |
| F5 | C8 | F6 | 105.70(18) |  | C30 | C29 | C32 | 121.67(19) |
| F5 | C8 | C5 | 112.21(17) |  | C29 | C30 | C25 | 122.32(19) |
| F6 | C8 | C5 | 112.65(17) |  | F19 | C31 | C27 | 110.63(19) |
| C10 | C9 | B1 | 122.29(18) |  | F20 | C31 | F19 | 105.26(18) |
| C14 | C9 | C10 | 115.63(19) |  | F20 | C31 | C27 | 113.17(18) |
| C14 | C9 | B1 | 121.69(18) |  | F21 | C31 | F19 | 106.53(18) |
| C11 | C10 | C9 | 122.1(2) |  | F21 | C31 | F20 | 107.25(19) |
| C10 | C11 | C15 | 118.7(5) |  | F21 | C31 | C27 | 113.46(19) |
| C10 | C11 | C15A | 117.6(8) |  | F22 | C32 | F23 | 105.92(18) |
| C10 | C11 | C15B | 123.7(8) |  | F22 | C32 | F24 | 106.88(18) |
| C12 | C11 | C10 | 120.9(2) |  | F22 | C32 | C29 | 113.98(18) |
| C12 | C11 | C15 | 120.4(5) |  | F23 | C32 | C29 | 111.12(18) |
| C12 | C11 | C15A | 120.4(9) |  | F24 | C32 | F23 | 105.12(18) |
| C12 | C11 | C15B | 115.3(8) |  | F24 | C32 | C29 | 113.17(18) |
| C13 | C12 | C11 | 118.2(2) |  | C9 | B1 | C1 | 111.72(17) |
| C12 | C13 | C14 | 120.8(2) |  | C17 | B1 | C1 | 104.14(16) |
| C12 | C13 | C16 | 120.5(2) |  | C17 | B1 | C9 | 111.19(17) |
| C14 | C13 | C16 | 118.7(2) |  | C25 | B1 | C1 | 113.12(17) |
| C13 | C14 | C9 | 122.4(2) |  | C25 | B1 | C9 | 103.50(16) |
| F7 | C15 | C11 | 111.9(8) |  | C25 | B1 | C17 | 113.41(17) |
| F8 | C15 | F7 | 104.0(7) |  | C2B | N1B | C6B | 125.4(2) |
| F8 | C15 | F9 | 106.8(7) |  | C11B | N2B | C7B | 118.6(2) |
| F8 | C15 | C11 | 114.6(7) |  | N1B | C2B | C1B | 118.1(2) |
| F9 | C15 | F7 | 105.5(8) |  | N1B | C2B | C3B | 117.2(2) |
| F9 | C15 | C11 | 113.2(17) |  | C3B | C2B | C1B | 124.7(2) |
| F10 | C16 | F12 | 103.1(4) |  | C4B | C3B | C2B | 119.8(2) |
| F10 | C16 | C13 | 113.5(5) |  | C3B | C4B | C5B | 120.9(2) |
| F11 | C16 | F10 | 108.3(4) |  | C6B | C5B | C4B | 118.9(2) |
| F11 | C16 | F12 | 106.7(3) |  | N1B | C6B | C5B | 117.8(2) |
| F11 | C16 | C13 | 114.3(3) |  | N1B | C6B | C7B | 114.54(19) |
| F12 | C16 | C13 | 110.2(2) |  | C5B | C6B | C7B | 127.6(2) |
| F11A | C16 | C13 | 116.1(14) |  | N2B | C7B | C6B | 114.2(2) |
| F11A | C16 | F12A | 103.2(11) |  | N2B | C7B | C8B | 123.2(2) |
| F12A | C16 | C13 | 105.0(11) |  | C8B | C7B | C6B | 122.6(2) |
| F10A | C16 | C13 | 115.2(13) |  | C9B | C8B | C7B | 117.3(2) |
| F10A | C16 | F11A | 109.2(12) |  | C10B | C9B | C8B | 120.4(2) |
| F10A | C16 | F12A | 106.8(11) |  | C9B | C10B | C11B | 119.3(2) |
| F11B | C16 | C13 | 116.1(5) |  | N2B | C11B | C10B | 121.2(2) |
| F11B | C16 | F12B | 109.7(9) |  | N2B | C11B | C12B | 116.6(2) |
| F11B | C16 | F10B | 106.1(5) |  | C10B | C11B | C12B | 122.2(2) |
| F12B | C16 | C13 | 111.9(11) |  | F7A | C15A | C11 | 114.7(19) |
| F12B | C16 | F10B | 103.7(7) |  | F7A | C15A | F8A | 107.3(13) |
| F10B | C16 | C13 | 108.4(6) |  | F9A | C15A | C11 | 109.3(15) |
| C18 | C17 | C22 | 115.49(19) |  | F9A | C15A | F7A | 108.2(13) |
| C18 | C17 | B1 | 124.79(19) |  | F9A | C15A | F8A | 106.9(14) |
| C22 | C17 | B1 | 119.53(19) |  | F8A | C15A | C11 | 110(2) |
| C17 | C18 | C19 | 122.0(2) |  | F7B | C15B | C11 | 110.9(15) |
| C18 | C19 | C23 | 119.4(2) |  | F7B | C15B | F9B | 107.1(12) |
| C20 | C19 | C18 | 121.0(2) |  | F7B | C15B | F8B | 106.8(13) |
| C20 | C19 | C23 | 119.3(2) |  | F9B | C15B | C11 | 113.4(13) |
| C19 | C20 | C21 | 118.3(2) |  | F9B | C15B | F8B | 106.4(13) |
| C20 | C21 | C22 | 120.3(2) |  | F8B | C15B | C11 | 112(3) |
| C20 | C21 | C24 | 120.3(2) |  |  |  |  |  |

Table 6 Torsion Angles for c030620\_1\_1.

| A | B | C | D | Angle/˚ |  | A | B | C | D | Angle/˚ |
| --- | --- | --- | --- | --- | --- | --- | --- | --- | --- | --- |
| C1 | C2 | C3 | C4 | -1.0(3) |  | C18 | C17 | B1 | C9 | -136.8(2) |
| C1 | C2 | C3 | C7 | 177.8(2) |  | C18 | C17 | B1 | C25 | -20.6(3) |
| C2 | C1 | C6 | C5 | 3.1(3) |  | C18 | C19 | C20 | C21 | -1.0(3) |
| C2 | C1 | B1 | C9 | -32.7(3) |  | C18 | C19 | C23 | F16 | -35.9(3) |
| C2 | C1 | B1 | C17 | 87.4(2) |  | C18 | C19 | C23 | F17 | 84.8(3) |
| C2 | C1 | B1 | C25 | -149.03(19) |  | C18 | C19 | C23 | F18 | -157.5(2) |
| C2 | C3 | C4 | C5 | 1.8(3) |  | C19 | C20 | C21 | C22 | -0.7(3) |
| C2 | C3 | C7 | F1 | 158.2(5) |  | C19 | C20 | C21 | C24 | -179.7(2) |
| C2 | C3 | C7 | F2 | -83.4(5) |  | C20 | C19 | C23 | F16 | 149.4(2) |
| C2 | C3 | C7 | F3 | 35.1(5) |  | C20 | C19 | C23 | F17 | -89.9(2) |
| C2 | C3 | C7 | F2A | -121.9(17) |  | C20 | C19 | C23 | F18 | 27.9(3) |
| C2 | C3 | C7 | F3A | -6.2(17) |  | C20 | C21 | C22 | C17 | 2.0(3) |
| C2 | C3 | C7 | F1A | 121.7(16) |  | C20 | C21 | C24 | F13 | -96.5(4) |
| C2 | C3 | C7 | F2B | -175.2(7) |  | C20 | C21 | C24 | F14 | 23.3(4) |
| C2 | C3 | C7 | F3B | -50.7(6) |  | C20 | C21 | C24 | F15 | 147.6(4) |
| C2 | C3 | C7 | F1B | 66.2(5) |  | C20 | C21 | C24 | F13A | -54.1(10) |
| C3 | C4 | C5 | C6 | -0.1(3) |  | C20 | C21 | C24 | F14A | 66.0(13) |
| C3 | C4 | C5 | C8 | -178.45(19) |  | C20 | C21 | C24 | F15A | -174.0(12) |
| C4 | C3 | C7 | F1 | -23.0(6) |  | C20 | C21 | C24 | F13B | -1.1(6) |
| C4 | C3 | C7 | F2 | 95.4(4) |  | C20 | C21 | C24 | F15B | -118.4(6) |
| C4 | C3 | C7 | F3 | -146.1(4) |  | C20 | C21 | C24 | F14B | 113.7(5) |
| C4 | C3 | C7 | F2A | 56.9(17) |  | C22 | C17 | C18 | C19 | -0.2(3) |
| C4 | C3 | C7 | F3A | 172.6(17) |  | C22 | C17 | B1 | C1 | -72.0(2) |
| C4 | C3 | C7 | F1A | -59.5(17) |  | C22 | C17 | B1 | C9 | 48.4(3) |
| C4 | C3 | C7 | F2B | 3.7(8) |  | C22 | C17 | B1 | C25 | 164.59(18) |
| C4 | C3 | C7 | F3B | 128.2(6) |  | C22 | C21 | C24 | F13 | 84.4(4) |
| C4 | C3 | C7 | F1B | -114.9(5) |  | C22 | C21 | C24 | F14 | -155.8(3) |
| C4 | C5 | C6 | C1 | -2.4(3) |  | C22 | C21 | C24 | F15 | -31.4(4) |
| C4 | C5 | C8 | F4 | 6.8(3) |  | C22 | C21 | C24 | F13A | 126.8(10) |
| C4 | C5 | C8 | F5 | 126.9(2) |  | C22 | C21 | C24 | F14A | -113.0(13) |
| C4 | C5 | C8 | F6 | -113.9(2) |  | C22 | C21 | C24 | F15A | 6.9(13) |
| C6 | C1 | C2 | C3 | -1.4(3) |  | C22 | C21 | C24 | F13B | 179.9(6) |
| C6 | C1 | B1 | C9 | 151.74(18) |  | C22 | C21 | C24 | F15B | 62.6(6) |
| C6 | C1 | B1 | C17 | -88.2(2) |  | C22 | C21 | C24 | F14B | -65.3(5) |
| C6 | C1 | B1 | C25 | 35.4(3) |  | C23 | C19 | C20 | C21 | 173.6(2) |
| C6 | C5 | C8 | F4 | -171.58(17) |  | C24 | C21 | C22 | C17 | -178.9(2) |
| C6 | C5 | C8 | F5 | -51.5(3) |  | C25 | C26 | C27 | C28 | -0.5(3) |
| C6 | C5 | C8 | F6 | 67.7(2) |  | C25 | C26 | C27 | C31 | -176.5(2) |
| C7 | C3 | C4 | C5 | -177.0(2) |  | C26 | C25 | C30 | C29 | -1.3(3) |
| C8 | C5 | C6 | C1 | 175.97(18) |  | C26 | C25 | B1 | C1 | 26.5(3) |
| C9 | C10 | C11 | C12 | 1.9(4) |  | C26 | C25 | B1 | C9 | -94.5(2) |
| C9 | C10 | C11 | C15 | -176.4(5) |  | C26 | C25 | B1 | C17 | 144.86(19) |
| C9 | C10 | C11 | C15A | 170.2(12) |  | C26 | C27 | C28 | C29 | -1.3(3) |
| C9 | C10 | C11 | C15B | 177.4(9) |  | C26 | C27 | C31 | F19 | 79.6(3) |
| C10 | C9 | C14 | C13 | -1.1(3) |  | C26 | C27 | C31 | F20 | -38.3(3) |
| C10 | C9 | B1 | C1 | 149.81(19) |  | C26 | C27 | C31 | F21 | -160.8(2) |
| C10 | C9 | B1 | C17 | 33.9(3) |  | C27 | C28 | C29 | C30 | 1.6(3) |
| C10 | C9 | B1 | C25 | -88.2(2) |  | C27 | C28 | C29 | C32 | -176.3(2) |
| C10 | C11 | C12 | C13 | -1.8(3) |  | C28 | C27 | C31 | F19 | -96.4(2) |
| C10 | C11 | C15 | F7 | -93.9(7) |  | C28 | C27 | C31 | F20 | 145.7(2) |
| C10 | C11 | C15 | F8 | 24.1(10) |  | C28 | C27 | C31 | F21 | 23.2(3) |
| C10 | C11 | C15 | F9 | 146.9(6) |  | C28 | C29 | C30 | C25 | -0.3(3) |
| C10 | C11 | C15A | F7A | -59.5(18) |  | C28 | C29 | C32 | F22 | 177.76(19) |
| C10 | C11 | C15A | F9A | 178.7(11) |  | C28 | C29 | C32 | F23 | 58.2(3) |
| C10 | C11 | C15A | F8A | 61.6(16) |  | C28 | C29 | C32 | F24 | -59.8(3) |
| C10 | C11 | C15B | F7B | 11.9(18) |  | C30 | C25 | C26 | C27 | 1.7(3) |
| C10 | C11 | C15B | F9B | -108.6(13) |  | C30 | C25 | B1 | C1 | -161.17(18) |
| C10 | C11 | C15B | F8B | 131.1(12) |  | C30 | C25 | B1 | C9 | 77.7(2) |
| C11 | C12 | C13 | C14 | 0.4(3) |  | C30 | C25 | B1 | C17 | -42.9(3) |
| C11 | C12 | C13 | C16 | -178.2(2) |  | C30 | C29 | C32 | F22 | -0.2(3) |
| C12 | C11 | C15 | F7 | 87.8(9) |  | C30 | C29 | C32 | F23 | -119.8(2) |
| C12 | C11 | C15 | F8 | -154.2(6) |  | C30 | C29 | C32 | F24 | 122.2(2) |
| C12 | C11 | C15 | F9 | -31.4(9) |  | C31 | C27 | C28 | C29 | 174.7(2) |
| C12 | C11 | C15A | F7A | 108.8(15) |  | C32 | C29 | C30 | C25 | 177.6(2) |
| C12 | C11 | C15A | F9A | -13(2) |  | B1 | C1 | C2 | C3 | -177.23(19) |
| C12 | C11 | C15A | F8A | -130.0(13) |  | B1 | C1 | C6 | C5 | 178.90(18) |
| C12 | C11 | C15B | F7B | -172.2(10) |  | B1 | C9 | C10 | C11 | 172.5(2) |
| C12 | C11 | C15B | F9B | 67.2(17) |  | B1 | C9 | C14 | C13 | -174.03(19) |
| C12 | C11 | C15B | F8B | -53.1(13) |  | B1 | C17 | C18 | C19 | -175.2(2) |
| C12 | C13 | C14 | C9 | 1.1(3) |  | B1 | C17 | C22 | C21 | 173.76(19) |
| C12 | C13 | C16 | F10 | -12.7(4) |  | B1 | C25 | C26 | C27 | 174.45(19) |
| C12 | C13 | C16 | F11 | -137.6(3) |  | B1 | C25 | C30 | C29 | -174.11(19) |
| C12 | C13 | C16 | F12 | 102.3(3) |  | N1B | C2B | C3B | C4B | 0.3(3) |
| C12 | C13 | C16 | F11A | -166.3(12) |  | N1B | C6B | C7B | N2B | -8.0(3) |
| C12 | C13 | C16 | F12A | 80.5(9) |  | N1B | C6B | C7B | C8B | 171.7(2) |
| C12 | C13 | C16 | F10A | -36.8(9) |  | N2B | C7B | C8B | C9B | 1.0(3) |
| C12 | C13 | C16 | F11B | 130.6(4) |  | C1B | C2B | C3B | C4B | -179.7(2) |
| C12 | C13 | C16 | F12B | 3.6(6) |  | C2B | N1B | C6B | C5B | -0.7(3) |
| C12 | C13 | C16 | F10B | -110.2(4) |  | C2B | N1B | C6B | C7B | 179.35(19) |
| C14 | C9 | C10 | C11 | -0.4(3) |  | C2B | C3B | C4B | C5B | 0.1(4) |
| C14 | C9 | B1 | C1 | -37.7(3) |  | C3B | C4B | C5B | C6B | -0.9(3) |
| C14 | C9 | B1 | C17 | -153.61(19) |  | C4B | C5B | C6B | N1B | 1.1(3) |
| C14 | C9 | B1 | C25 | 84.3(2) |  | C4B | C5B | C6B | C7B | -178.9(2) |
| C14 | C13 | C16 | F10 | 168.6(3) |  | C5B | C6B | C7B | N2B | 172.0(2) |
| C14 | C13 | C16 | F11 | 43.7(3) |  | C5B | C6B | C7B | C8B | -8.3(3) |
| C14 | C13 | C16 | F12 | -76.4(3) |  | C6B | N1B | C2B | C1B | 180.0(2) |
| C14 | C13 | C16 | F11A | 15.0(12) |  | C6B | N1B | C2B | C3B | -0.1(3) |
| C14 | C13 | C16 | F12A | -98.2(9) |  | C6B | C7B | C8B | C9B | -178.6(2) |
| C14 | C13 | C16 | F10A | 144.5(9) |  | C7B | N2B | C11B | C10B | -0.4(3) |
| C14 | C13 | C16 | F11B | -48.1(5) |  | C7B | N2B | C11B | C12B | -179.6(2) |
| C14 | C13 | C16 | F12B | -175.1(6) |  | C7B | C8B | C9B | C10B | -0.8(3) |
| C14 | C13 | C16 | F10B | 71.1(4) |  | C8B | C9B | C10B | C11B | 0.0(4) |
| C15 | C11 | C12 | C13 | 176.4(5) |  | C9B | C10B | C11B | N2B | 0.6(4) |
| C16 | C13 | C14 | C9 | 179.8(2) |  | C9B | C10B | C11B | C12B | 179.8(3) |
| C17 | C18 | C19 | C20 | 1.5(3) |  | C11B | N2B | C7B | C6B | 179.24(19) |
| C17 | C18 | C19 | C23 | -173.1(2) |  | C11B | N2B | C7B | C8B | -0.4(3) |
| C18 | C17 | C22 | C21 | -1.5(3) |  | C15A | C11 | C12 | C13 | -169.9(12) |
| C18 | C17 | B1 | C1 | 102.8(2) |  | C15B | C11 | C12 | C13 | -177.8(9) |

Table 7 Hydrogen Atom Coordinates (Å×104) and Isotropic Displacement Parameters (Å2×103) for c030620\_1\_1.

| Atom | *x* | *y* | *z* | U(eq) |
| --- | --- | --- | --- | --- |
| H1C | 5079.96 | 216.5 | 8801.01 | 33 |
| H2 | 1260.19 | 8083.35 | 5692.91 | 24 |
| H4 | 180.68 | 6642.98 | 4370.39 | 26 |
| H6 | 2819.95 | 4573.89 | 6672.95 | 20 |
| H10 | 3443.06 | 7304.16 | 8139.52 | 24 |
| H12 | 4111.48 | 9714.02 | 6106.64 | 27 |
| H14 | 3283.94 | 7630.04 | 5431.73 | 21 |
| H18 | 3319.79 | 4292.14 | 8945.3 | 22 |
| H20 | 591.56 | 5886.43 | 9956.5 | 29 |
| H22 | 1215.75 | 7771.72 | 7469.47 | 22 |
| H26 | 4362.28 | 4930.36 | 6189.14 | 19 |
| H28 | 7408.95 | 2656.04 | 8046.92 | 24 |
| H30 | 4814.43 | 4845.8 | 8795.09 | 20 |
| H1B | 1623(19) | 5616(17) | 2455(16) | 24 |
| H1BA | 3553.81 | 4452.75 | 2844.44 | 45 |
| H1BB | 4077.76 | 3066.69 | 3215.98 | 45 |
| H1BC | 3704.17 | 3619.95 | 3956.41 | 45 |
| H1BD | 3863.74 | 3777.69 | 2729.53 | 30(18) |
| H1BE | 3995.51 | 2985.37 | 3848.39 | 9(15) |
| H1BF | 3476.49 | 4376.32 | 3438.92 | 8(15) |
| H3B | 2677.58 | 2206.11 | 3690.71 | 37 |
| H4B | 805.06 | 2616.32 | 3304.63 | 37 |
| H5B | -643.39 | 4552.81 | 2461.1 | 33 |
| H8B | -1847.47 | 6516.88 | 1507.36 | 37 |
| H9B | -2946 | 8563.2 | 687.72 | 44 |
| H10B | -2138.79 | 9741.94 | 607.47 | 39 |
| H12A | 417.06 | 8822.31 | 1997.09 | 69 |
| H12B | -554.27 | 9929.7 | 1092.86 | 69 |
| H12C | 568.89 | 9014.08 | 925.33 | 69 |

Table 8 Atomic Occupancy for c030620\_1\_1.

| Atom | *Occupancy* |  | Atom | *Occupancy* |  | Atom | *Occupancy* |
| --- | --- | --- | --- | --- | --- | --- | --- |
| F1 | 0.588(3) |  | F2 | 0.588(3) |  | F3 | 0.588(3) |
| F7 | 0.588(3) |  | F8 | 0.588(3) |  | F9 | 0.588(3) |
| F10 | 0.588(3) |  | F11 | 0.588(3) |  | F12 | 0.588(3) |
| F13 | 0.588(3) |  | F14 | 0.588(3) |  | F15 | 0.588(3) |
| C15 | 0.588(3) |  | H1BA | 0.59(3) |  | H1BB | 0.59(3) |
| H1BC | 0.59(3) |  | H1BD | 0.41(3) |  | H1BE | 0.41(3) |
| H1BF | 0.41(3) |  | F13A | 0.0888(19) |  | F14A | 0.0888(19) |
| F15A | 0.0888(19) |  | F13B | 0.323(3) |  | F15B | 0.323(3) |
| F14B | 0.323(3) |  | F2A | 0.0888(19) |  | F3A | 0.0888(19) |
| F1A | 0.0888(19) |  | F2B | 0.323(3) |  | F3B | 0.323(3) |
| F1B | 0.323(3) |  | F11A | 0.0888(19) |  | F12A | 0.0888(19) |
| F10A | 0.0888(19) |  | F11B | 0.323(3) |  | F12B | 0.323(3) |
| F10B | 0.323(3) |  | C15A | 0.0888(19) |  | F7A | 0.0888(19) |
| F9A | 0.0888(19) |  | F8A | 0.0888(19) |  | F7B | 0.323(3) |
| C15B | 0.323(3) |  | F9B | 0.323(3) |  | F8B | 0.323(3) |

Experimental

Single crystals of C45H26BCl3F24N2
[c030620\_1\_1]
were
[].
A suitable crystal was selected and
[]
on a
Bruker APEX-II Duo (Mo)
diffractometer. The crystal was kept at 100.0(1) K during data collection.
Using Olex2 [1], the structure was solved with the
SHELXT
[2] structure solution program using
Intrinsic Phasing
and refined with the
SHELXL
[3] refinement package using
Least Squares
minimisation.

1. Dolomanov, O.V., Bourhis, L.J., Gildea, R.J, Howard, J.A.K. & Puschmann, H.
   (2009), J. Appl. Cryst. 42, 339-341.
2. Sheldrick, G.M. (2015). Acta Cryst. A71, 3-8.
3. Sheldrick, G.M. (2015). Acta Cryst. C71, 3-8.

Crystal structure determination of
[c030620\_1\_1]

**Crystal Data**
for C45H26BCl3F24N2 (*M*=1167.84 g/mol):
triclinic, space group P-1 (no. 2),
*a* = 13.3366(15) Å, *b* = 14.1573(15) Å, *c* = 15.5099(15) Å, *α* = 66.246(3)°, *β* = 89.877(4)°, *γ* = 64.036(3)°,
*V*= 2355.2(4) Å3,
*Z* = 2,
*T* = 100.0(1) K,
μ(MoKα) = 0.327 mm-1,
*Dcalc* = 1.647 g/cm3,
35730 reflections measured (2.936° ≤ 2Θ ≤ 55.254°),
10885 unique (*R*int = 0.0569, Rsigma = 0.0717) which were used in all calculations.
The final *R*1 was 0.0469
(I > 2σ(I)) and *wR*2 was 0.1198 (all data).

Refinement model description

Number of restraints - 545,
number of constraints - unknown.

Details:

```
1. Fixed Uiso
```

This report has been created with Olex2, compiled on
2020.02.04 svn.rd84adfe8 for OlexSys. Please
let us know
if there are any errors or if you would like to have additional features.
