# Supplementary material for: London Dispersion versus Intramolecular Hydrogen Bond in Bis‐Pyridines: How Accurate Is DFT for Competing Noncovalent Interactions in the Condensed Phase?
Source: Chemistry. 2025 Oct 23;31(66):e02745. doi: 10.1002/chem.202502745 (PMC12648470; doi:10.1002/chem.202502745)

checkCIF/PLATON report

Structure factors have been supplied for datablock(s) c030620_1_1

THIS REPORT IS FOR GUIDANCE ONLY. IF USED AS PART OF A REVIEW PROCEDURE FOR PUBLICATION, IT SHOULD NOT REPLACE THE EXPERTISE OF AN EXPERIENCED CRYSTALLOGRAPHIC REFEREE.

No syntax errors found. CIF dictionary Interpreting this report

Datablock: c030620_1_1

```
Bond precision:  C-C = 0.0036 A          Wavelength=0.71073

Cell:          a=13.3366(15)     b=14.1573(15)    c=15.5099(15)
               alpha=66.246(3)   beta=89.877(4)   gamma=64.036(3)
Temperature:   100 K
```

	Calculated	Reported
Volume	2355.2(4)	2355.2(4)
Space group	P -1	P -1
Hall group	-P 1	-P 1
Moiety formula	C32 H12 B F24, C12 H13 N2, C H Cl3	C H Cl3, C32 H12 B F24, C12 H13 N2
Sum formula	C45 H26 B Cl3 F24 N2	C45 H26 B Cl3 F24 N2
Mr	1167.79	1167.84
Dx,g cm-3	1.647	1.647
Z	2	2
Mu (mm-1)	0.327	0.327
F000	1164.0	1164.0
F000’	1165.86	
h,k,lmax	17,18,20	17,18,20
Nref	10958	10885
Tmin,Tmax	0.946,0.987	0.597,0.746
Tmin’	0.946	

```
Correction method= # Reported T Limits: Tmin=0.597 Tmax=0.746
AbsCorr = MULTI-SCAN

Data completeness= 0.993          Theta(max)= 27.627

R(reflections)= 0.0469( 6822)     wR2(reflections)= 0.1198( 10885)

S = 0.973                  Npar= 808
```

The following ALERTS were generated. Each ALERT has the format
test-name_ALERT_alert-type_alert-level.
Click on the hyperlinks for more details of the test.

Alert level C

```
PLAT213_ALERT_2_C Atom F2             has ADP max/min Ratio .....        3.3 prolat
PLAT213_ALERT_2_C Atom F14            has ADP max/min Ratio .....        3.6 prolat
PLAT213_ALERT_2_C Atom F18            has ADP max/min Ratio .....        3.5 prolat
PLAT213_ALERT_2_C Atom F2A            has ADP max/min Ratio .....        3.3 prolat
PLAT213_ALERT_2_C Atom F2B            has ADP max/min Ratio .....        3.4 prolat
PLAT213_ALERT_2_C Atom F13B           has ADP max/min Ratio .....        3.6 prolat
PLAT213_ALERT_2_C Atom F14A           has ADP max/min Ratio .....        3.6 prolat
PLAT220_ALERT_2_C NonSolvent Resd 1  F   Ueq(max) / Ueq(min) Range        3.1 Ratio
PLAT223_ALERT_4_C Solv./Anion  Resd 2  H   Ueq(max)/Ueq(min) Range        8.6 Ratio
PLAT245_ALERT_2_C U(iso) H1BE      Smaller than U(eq) C1B       by      0.021 Ang**2
PLAT245_ALERT_2_C U(iso) H1BF      Smaller than U(eq) C1B       by      0.022 Ang**2
PLAT911_ALERT_3_C Missing FCF Refl Between Thmin & STh/L=    0.600          3 Report
```

Alert level G

```
PLAT002_ALERT_2_G Number of Distance or Angle Restraints on AtSite         45 Note
PLAT042_ALERT_1_G Calc. and Reported MoietyFormula Strings  Differ     Please Check
PLAT171_ALERT_4_G The CIF-Embedded .res File Contains EADP Records         19 Report
PLAT172_ALERT_4_G The CIF-Embedded .res File Contains DFIX Records          1 Report
PLAT176_ALERT_4_G The CIF-Embedded .res File Contains SADI Records          9 Report
PLAT187_ALERT_4_G The CIF-Embedded .res File Contains RIGU Records          3 Report
PLAT230_ALERT_2_G Hirshfeld Test Diff for    F2A      --C7       .        8.8 s.u.
PLAT230_ALERT_2_G Hirshfeld Test Diff for    F11B     --C16      .        5.5 s.u.
PLAT230_ALERT_2_G Hirshfeld Test Diff for    F12A     --C16      .        7.0 s.u.
PLAT230_ALERT_2_G Hirshfeld Test Diff for    F13B     --C24      .        6.0 s.u.
PLAT230_ALERT_2_G Hirshfeld Test Diff for    F15A     --C24      .       10.8 s.u.
PLAT242_ALERT_2_G Low    'MainMol' Ueq as Compared to Neighbors of         C7 Check
PLAT242_ALERT_2_G Low    'MainMol' Ueq as Compared to Neighbors of        C16 Check
PLAT242_ALERT_2_G Low    'MainMol' Ueq as Compared to Neighbors of        C23 Check
PLAT242_ALERT_2_G Low    'MainMol' Ueq as Compared to Neighbors of        C24 Check
PLAT242_ALERT_2_G Low    'MainMol' Ueq as Compared to Neighbors of        C31 Check
PLAT301_ALERT_3_G Main Residue  Disorder ..............(Resd  1  )        23% Note
PLAT432_ALERT_2_G Short Inter X...Y Contact  F10A     ..C3B              2.92 Ang.
                                                    x,1+y,z  =      1_565 Check
PLAT434_ALERT_2_G Short Inter HL..HL Contact Cl1C     ..F9A              3.08 Ang.
                                                   x,-1+y,z  =      1_545 Check
PLAT434_ALERT_2_G Short Inter HL..HL Contact Cl2C     ..F13A             3.05 Ang.
                                                 1+x,-1+y,z  =      1_645 Check
PLAT434_ALERT_2_G Short Inter HL..HL Contact F18      ..F13A             2.50 Ang.
                                                 -x,1-y,2-z  =      2_567 Check
PLAT434_ALERT_2_G Short Inter HL..HL Contact F20      ..F11B             2.79 Ang.
                                                1-x,1-y,1-z  =      2_666 Check
PLAT434_ALERT_2_G Short Inter HL..HL Contact F24      ..F7B              2.82 Ang.
                                                1-x,1-y,2-z  =      2_667 Check
PLAT720_ALERT_4_G Number of Unusual/Non-Standard Labels ..........          6 Note
PLAT860_ALERT_3_G Number of Least-Squares Restraints .............        545 Note
PLAT912_ALERT_4_G Missing # of FCF Reflections Above STh/L=  0.600         70 Note
PLAT941_ALERT_3_G Average HKL Measurement Multiplicity ...........        3.3 Low
PLAT978_ALERT_2_G Number C-C Bonds with Positive Residual Density.          5 Info
PLAT992_ALERT_5_G Repd & Actual _reflns_number_gt Values Differ by          1 Check
```

---

0 **ALERT level A** = Most likely a serious problem - resolve or explain
0 **ALERT level B** = A potentially serious problem, consider carefully
12 **ALERT level C** = Check. Ensure it is not caused by an omission or oversight

```
29 ALERT level G = General information/check it is not something unexpected

 1 ALERT type 1 CIF construction/syntax error, inconsistent or missing data
28 ALERT type 2 Indicator that the structure model may be wrong or deficient
 4 ALERT type 3 Indicator that the structure quality may be low
 7 ALERT type 4 Improvement, methodology, query or suggestion
 1 ALERT type 5 Informative message, check
```

It is advisable to attempt to resolve as many as possible of the alerts in all categories. Often the minor alerts point to easily fixed oversights, errors and omissions in your CIF or refinement strategy, so attention to these fine details can be worthwhile. In order to resolve some of the more serious problems it may be necessary to carry out additional measurements or structure refinements. However, the purpose of your study may justify the reported deviations and the more serious of these should normally be commented upon in the discussion or experimental section of a paper or in the "special_details" fields of the CIF. checkCIF was carefully designed to identify outliers and unusual parameters, but every test has its limitations and alerts that are not important in a particular case may appear. Conversely, the absence of alerts does not guarantee there are no aspects of the results needing attention. It is up to the individual to critically assess their own results and, if necessary, seek expert advice.

Publication of your CIF in IUCr journals

A basic structural check has been run on your CIF. These basic checks will be run on all CIFs submitted for publication in IUCr journals (*Acta Crystallographica*, *Journal of Applied Crystallography*, *Journal of Synchrotron Radiation*); however, if you intend to submit to *Acta Crystallographica Section C* or *E* or *IUCrData*, you should make sure that full publication checks are run on the final version of your CIF prior to submission.

Publication of your CIF in other journals

Please refer to the *Notes for Authors* of the relevant journal for any special instructions relating to CIF submission.

PLATON version of 04/06/2020; check.def file version of 02/06/2020

Datablock c030620_1_1 - ellipsoid plot

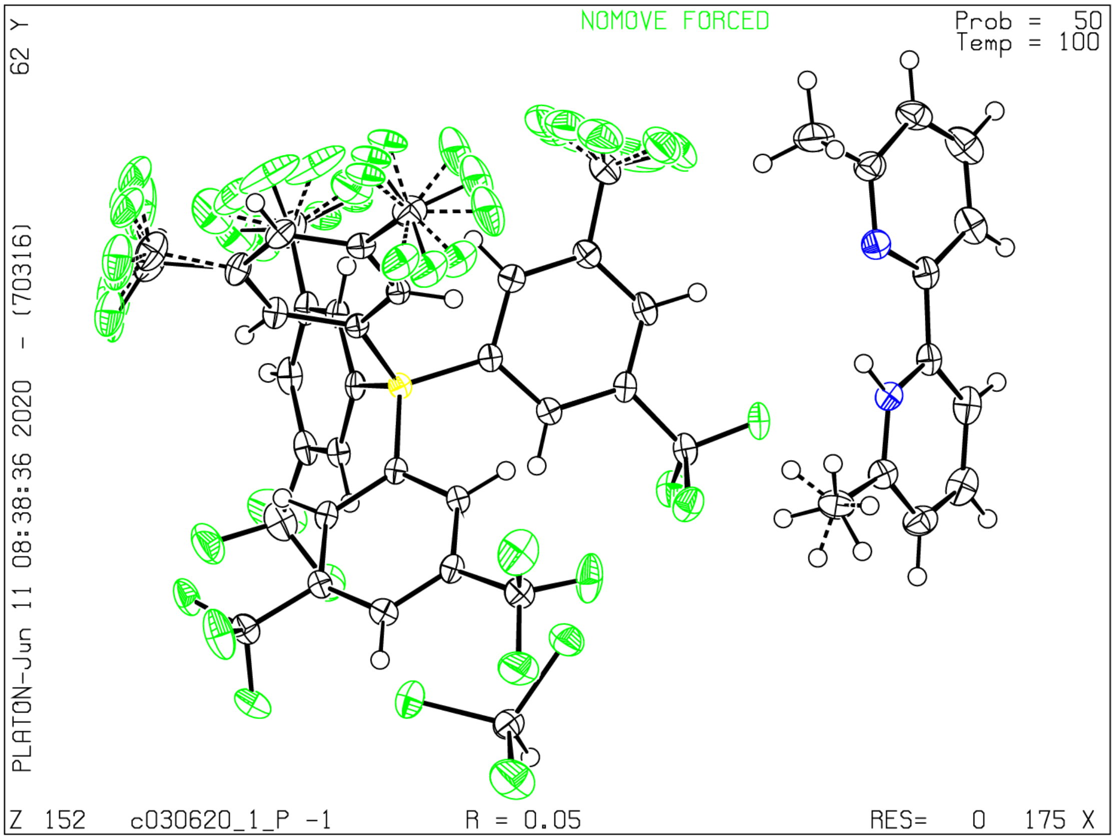

Supplement: Supplementary file 1 — Supporting Information [file CHEM-31-e02745-s002.zip › Crystal_structures/2b/checkcif.pdf]
