# Supplementary material for: London Dispersion versus Intramolecular Hydrogen Bond in Bis‐Pyridines: How Accurate Is DFT for Competing Noncovalent Interactions in the Condensed Phase?
Source: Chemistry. 2025 Oct 23;31(66):e02745. doi: 10.1002/chem.202502745 (PMC12648470; doi:10.1002/chem.202502745)
Supplement: Supplementary file 1 — Supporting Information [file CHEM-31-e02745-s002.zip › Crystal_structures/3b/c031219_2_2_tables.html]

c031219\_2\_2


# c031219\_2\_2

Table 1 Crystal data and structure refinement for c031219\_2\_2.

| Identification code | c031219\_2\_2 |
| Empirical formula | C50H37BF24N2 |
| Formula weight | 1132.62 |
| Temperature/K | 100.0(1) |
| Crystal system | triclinic |
| Space group | P-1 |
| a/Å | 12.98390(10) |
| b/Å | 13.14960(10) |
| c/Å | 16.88170(10) |
| α/° | 97.5750(10) |
| β/° | 110.6430(10) |
| γ/° | 108.0880(10) |
| Volume/Å3 | 2467.80(4) |
| Z | 2 |
| ρcalcg/cm3 | 1.524 |
| μ/mm‑1 | 1.366 |
| F(000) | 1144.0 |
| Crystal size/mm3 | 0.293 × 0.224 × 0.139 |
| Radiation | Cu Kα (λ = 1.54184) |
| 2Θ range for data collection/° | 5.812 to 160.502 |
| Index ranges | -16 ≤ h ≤ 14, -16 ≤ k ≤ 16, -21 ≤ l ≤ 21 |
| Reflections collected | 68819 |
| Independent reflections | 10503 [Rint = 0.0383, Rsigma = 0.0209] |
| Data/restraints/parameters | 10503/2008/995 |
| Goodness-of-fit on F2 | 1.075 |
| Final R indexes [I>=2σ (I)] | R1 = 0.0389, wR2 = 0.0970 |
| Final R indexes [all data] | R1 = 0.0463, wR2 = 0.1037 |
| Largest diff. peak/hole / e Å-3 | 0.33/-0.28 |

Table 2 Fractional Atomic Coordinates (×104) and Equivalent Isotropic Displacement Parameters (Å2×103) for c031219\_2\_2. Ueq is defined as 1/3 of of the trace of the orthogonalised UIJ tensor.

| Atom | *x* | *y* | *z* | U(eq) |
| F8 | 1493.0(8) | 1294.4(8) | 5587.1(7) | 41.6(2) |
| F17 | 8270(6) | 8502(5) | 8977(5) | 46(2) |
| F4A | 3490(20) | 4398(12) | 9726(6) | 12(2) |
| F20 | 10632(9) | 5903(8) | 9462(6) | 37.1(11) |
| F7 | 1784.5(9) | -203.7(8) | 5666.8(8) | 50.2(3) |
| F22 | 9390(5) | 807(7) | 8696(4) | 35.2(9) |
| F6A | 3390(20) | 4842(14) | 8527(12) | 24(3) |
| F10 | 4751(7) | 325(4) | 3861(5) | 37.1(10) |
| F15 | 7664(7) | 7398(5) | 5915(5) | 27.4(17) |
| F9 | 1258.1(9) | 180.5(11) | 4432.0(7) | 60.3(3) |
| F5A | 2202(13) | 3258(15) | 8488(14) | 31(4) |
| F24 | 8425(6) | 434(8) | 7297(4) | 46.5(11) |
| F19 | 11254(5) | 5214(4) | 8581(4) | 41.0(10) |
| F12 | 6418(4) | 1633(7) | 4642(5) | 69.5(19) |
| F21 | 11539(7) | 4822(6) | 9818(4) | 44.2(10) |
| N2C | 6094.1(11) | 1705.7(10) | 2516.5(8) | 25.7(2) |
| F18 | 7332(9) | 7353(9) | 9506(6) | 60.6(16) |
| N1C | 6484.7(11) | 3787.9(10) | 3071.5(8) | 26.6(2) |
| F23 | 7484(5) | 208(6) | 8108(5) | 50.9(11) |
| F2 | 7033(6) | 2586(9) | 10775(6) | 66(3) |
| F16 | 6403(4) | 8072(5) | 8572(6) | 62.2(17) |
| F14 | 5947(6) | 6101(7) | 5115(4) | 43(2) |
| F1 | 6027(6) | 1064(5) | 9778(5) | 53.4(19) |
| C9 | 5219.3(11) | 2532.8(10) | 6540.5(8) | 19.2(2) |
| C6C | 7192.1(12) | 2424.7(11) | 2690.9(8) | 22.1(3) |
| C2 | 5986.6(11) | 2741.5(10) | 8882.5(8) | 20.3(3) |
| C25 | 7415.9(11) | 3296.7(11) | 7838.5(8) | 20.4(3) |
| F11 | 4933(7) | 1950(5) | 3771(4) | 57.4(13) |
| C18 | 6487.7(11) | 5003.2(11) | 6644.6(8) | 21.6(3) |
| C5 | 4250.6(12) | 3525.3(11) | 8882.4(8) | 22.1(3) |
| C1 | 5593.7(11) | 3261.7(10) | 8231.0(8) | 18.5(2) |
| C17 | 6392.2(11) | 4722.5(11) | 7393.0(8) | 19.7(2) |
| C10 | 4001.3(12) | 1966.0(10) | 6305.7(8) | 20.5(3) |
| C14 | 5615.0(12) | 2283.4(11) | 5892.6(9) | 22.1(3) |
| C3 | 5516.5(12) | 2601.8(11) | 9505.5(8) | 23.0(3) |
| F3 | 5254(6) | 1649(7) | 10561(5) | 48.4(16) |
| C6 | 4703.0(11) | 3639.1(10) | 8250.9(8) | 20.4(3) |
| C5C | 7431.5(12) | 3607.6(11) | 3040.0(8) | 23.0(3) |
| C22 | 6671.5(12) | 5617.0(11) | 8091.2(9) | 23.7(3) |
| C7C | 7975.6(12) | 2008.0(11) | 2532.7(9) | 23.6(3) |
| C26 | 8513.5(12) | 4194.2(12) | 8274.7(9) | 24.1(3) |
| C11 | 3233.1(12) | 1218.6(11) | 5482.6(9) | 22.5(3) |
| C12 | 3644.3(13) | 1001.6(11) | 4852.4(9) | 24.4(3) |
| C30 | 7459.5(12) | 2240.7(11) | 7787.9(9) | 23.4(3) |
| C4C | 8555.0(13) | 4436.3(11) | 3330.0(9) | 24.4(3) |
| C13 | 4848.0(13) | 1547.6(11) | 5068.4(9) | 24.0(3) |
| C19 | 6851.3(12) | 6099.7(12) | 6596.8(9) | 24.8(3) |
| C4 | 4652.7(12) | 2995.4(11) | 9517.8(8) | 23.8(3) |
| C8C | 7649.5(12) | 874.2(11) | 2198.3(9) | 24.6(3) |
| C21 | 7011.4(13) | 6710.1(11) | 8038.8(10) | 27.1(3) |
| C29 | 8519.9(13) | 2090.6(13) | 8185.1(9) | 27.4(3) |
| C3C | 8750.0(13) | 5531.2(11) | 3692.7(9) | 25.7(3) |
| F13 | 7510(7) | 5724(6) | 5493(5) | 54(2) |
| C8 | 3342.1(13) | 3988.9(12) | 8900.1(9) | 28.2(3) |
| C10C | 5732.3(13) | 602.5(12) | 2201.4(10) | 30.5(3) |
| C9C | 6493.8(13) | 171.2(12) | 2042.6(9) | 28.4(3) |
| C1C | 6665.1(14) | 4839.4(13) | 3403.8(9) | 29.4(3) |
| C27 | 9577.6(12) | 4048.5(13) | 8655.6(9) | 29.0(3) |
| C20 | 7109.1(13) | 6966.2(12) | 7288.9(10) | 27.2(3) |
| C15 | 1950.3(13) | 628.1(12) | 5288.2(10) | 30.4(3) |
| C2C | 7758.1(14) | 5716.2(12) | 3714.9(9) | 28.8(3) |
| C15C | 8479.1(13) | 399.0(12) | 1979.7(10) | 29.9(3) |
| C11C | 9975.3(14) | 6473.5(12) | 4061.8(10) | 31.7(3) |
| C7 | 5980.5(13) | 2026.7(13) | 10175.5(9) | 31.2(3) |
| C28 | 9595.3(13) | 2996.6(14) | 8626.2(9) | 30.6(3) |
| C16 | 5297.6(15) | 1376.2(12) | 4381.6(10) | 32.4(3) |
| C24 | 7283.2(16) | 7645.8(12) | 8791.4(12) | 37.4(4) |
| C23 | 6991.8(16) | 6333.1(13) | 5787.1(11) | 36.1(4) |
| C12C | 10291.4(16) | 7063.9(13) | 5012.0(10) | 36.7(4) |
| C31 | 10764(6) | 4993(6) | 9140(5) | 27.7(14) |
| C32 | 8467(7) | 896(6) | 8076(6) | 29.1(14) |
| B1 | 6137.2(13) | 3446.6(12) | 7491.7(9) | 18.5(3) |
| C13C | 9934.8(19) | 7299.6(14) | 3499.9(12) | 44.5(4) |
| C17C | 7924.2(17) | -117.6(17) | 980.7(11) | 43.9(4) |
| C14C | 10944.6(16) | 6044.2(15) | 4065.5(15) | 50.8(5) |
| C18C | 8598(2) | -509.3(17) | 2446.9(14) | 50.7(5) |
| C16C | 9715.3(16) | 1285.4(16) | 2250.3(17) | 58.7(6) |
| F1A | 5319(8) | 1115(7) | 10105(7) | 82(4) |
| F2A | 7086(8) | 2167(11) | 10362(8) | 55(3) |
| F3A | 6158(11) | 2707(8) | 11011(4) | 69(2) |
| F1B | 5749(9) | 961(6) | 9799(5) | 46(2) |
| F3B | 5554(9) | 1997(10) | 10772(7) | 51(3) |
| F2B | 7160(6) | 2476(9) | 10606(7) | 29.9(19) |
| F5B | 3278(13) | 4259(11) | 9667(6) | 50(3) |
| F6B | 2219(10) | 3356(12) | 8308(8) | 54(3) |
| F4B | 3593(15) | 4969(9) | 8665(10) | 33(2) |
| F6 | 3181(11) | 4676(8) | 8417(7) | 50(3) |
| F4 | 3614(13) | 4467(9) | 9735(5) | 35(3) |
| F5 | 2274(8) | 3122(9) | 8622(8) | 41(2) |
| F20A | 10553(12) | 6025(11) | 9345(8) | 51(2) |
| C31A | 10668(9) | 5120(9) | 9000(7) | 40(2) |
| F21A | 11566(9) | 5020(9) | 9643(7) | 73(2) |
| F19A | 11102(9) | 5404(9) | 8414(6) | 86(3) |
| F13A | 7793(12) | 7211(9) | 5808(12) | 62(5) |
| F14A | 6885(12) | 5436(8) | 5222(7) | 72(3) |
| F15A | 5894(9) | 6419(11) | 5281(8) | 53(3) |
| F14B | 7905(8) | 6044(8) | 5740(7) | 50(2) |
| F15B | 6084(10) | 5890(12) | 5035(8) | 70(4) |
| F13B | 7438(11) | 7447(7) | 5909(10) | 44(3) |
| F17A | 7601(11) | 7370(10) | 9577(6) | 68(3) |
| F16A | 8249(7) | 8535(6) | 8940(6) | 39(2) |
| F18A | 6417(7) | 7956(8) | 8708(8) | 82(3) |
| F10A | 6436(6) | 1359(5) | 4771(5) | 42.5(11) |
| F11A | 4677(8) | 453(7) | 3761(7) | 57(2) |
| F12A | 5543(9) | 2223(5) | 4052(5) | 59.6(16) |
| F24A | 8330(10) | 432(12) | 7419(7) | 82(3) |
| F23A | 7627(9) | 381(10) | 8381(9) | 88(3) |
| F22A | 9518(8) | 994(10) | 8769(6) | 69(3) |
| C32A | 8499(11) | 993(9) | 8200(9) | 48(3) |

Table 3 Anisotropic Displacement Parameters (Å2×103) for c031219\_2\_2. The Anisotropic displacement factor exponent takes the form: -2π2[h2a\*2U11+2hka\*b\*U12+…].

| Atom | U11 | U22 | U33 | U23 | U13 | U12 |
| F8 | 24.8(4) | 32.5(5) | 60.6(6) | -0.7(4) | 16.4(4) | 9.3(4) |
| F17 | 57(4) | 28(3) | 41(3) | -9(2) | 31(3) | -2(2) |
| F4A | 8(5) | 11(4) | 21(4) | 4(3) | 9(3) | 6(3) |
| F20 | 24.0(19) | 31(2) | 43.7(17) | -9.6(16) | 9.9(15) | 7.2(13) |
| F7 | 40.8(6) | 30.8(5) | 84.8(8) | 21.7(5) | 36.1(6) | 7.4(4) |
| F22 | 35.6(15) | 36.3(19) | 36.8(16) | 13.3(14) | 7.9(13) | 24.8(12) |
| F6A | 15(7) | 50(6) | 19(5) | 12(5) | 9(6) | 23(5) |
| F10 | 56(2) | 25.7(13) | 32.7(19) | -1.4(12) | 25.0(16) | 16.4(14) |
| F15 | 25(2) | 33(2) | 21(2) | 11.3(18) | 12.8(18) | 0.8(18) |
| F9 | 26.3(5) | 81.4(8) | 35.7(5) | -16.6(5) | 4.4(4) | -4.0(5) |
| F5A | 19(4) | 32(6) | 33(7) | -8(6) | 1(4) | 15(4) |
| F24 | 59(2) | 47(2) | 35.3(17) | -0.6(16) | 12.2(15) | 35.1(17) |
| F19 | 27.8(13) | 34.6(13) | 58(2) | -2.5(13) | 29.4(15) | 0.9(10) |
| F12 | 29.6(15) | 107(5) | 39(2) | -23(3) | 20.1(13) | -6(2) |
| F21 | 22.8(14) | 50(2) | 39.8(16) | -5.0(14) | -3.1(12) | 13.4(14) |
| N2C | 25.3(6) | 28.1(6) | 23.7(6) | 4.1(5) | 12.6(5) | 8.6(5) |
| F18 | 107(4) | 24.8(19) | 60(3) | 0(2) | 67(3) | 5(2) |
| N1C | 30.2(6) | 32.0(6) | 24.4(6) | 9.9(5) | 14.4(5) | 16.1(5) |
| F23 | 34.5(15) | 32.3(15) | 90(3) | 26.9(16) | 23.8(15) | 17.0(11) |
| F2 | 53(3) | 60(3) | 41(4) | 30(3) | -13(3) | -7(3) |
| F16 | 37.2(19) | 28.6(16) | 104(4) | -17.0(19) | 25.0(19) | 10.7(14) |
| F14 | 45(3) | 40(4) | 10(2) | 3(2) | 0(2) | -12(2) |
| F1 | 95(5) | 54(3) | 47(3) | 31(2) | 38(3) | 57(4) |
| C9 | 22.1(6) | 17.4(6) | 19.9(6) | 7.2(5) | 9.4(5) | 8.2(5) |
| C6C | 23.6(6) | 25.0(6) | 16.1(6) | 5.7(5) | 7.9(5) | 7.5(5) |
| C2 | 18.9(6) | 19.9(6) | 20.0(6) | 5.1(5) | 7.1(5) | 6.4(5) |
| C25 | 21.2(6) | 24.1(6) | 17.9(6) | 5.9(5) | 10.5(5) | 8.5(5) |
| F11 | 103(3) | 69(2) | 62(2) | 47(2) | 68(3) | 59(2) |
| C18 | 21.7(6) | 22.7(6) | 20.3(6) | 6.6(5) | 8.1(5) | 8.9(5) |
| C5 | 19.7(6) | 23.0(6) | 19.9(6) | 3.2(5) | 8.0(5) | 5.0(5) |
| C1 | 17.3(6) | 16.7(6) | 16.6(5) | 2.8(4) | 5.8(5) | 2.7(5) |
| C17 | 16.8(6) | 20.8(6) | 21.8(6) | 6.3(5) | 8.3(5) | 7.2(5) |
| C10 | 23.4(6) | 19.3(6) | 20.3(6) | 6.6(5) | 10.5(5) | 8.1(5) |
| C14 | 23.0(6) | 20.3(6) | 24.0(6) | 6.2(5) | 11.8(5) | 7.2(5) |
| C3 | 22.5(6) | 23.6(6) | 18.9(6) | 7.1(5) | 7.0(5) | 5.2(5) |
| F3 | 53(3) | 66(4) | 53(4) | 46(3) | 35(3) | 31(3) |
| C6 | 20.6(6) | 20.2(6) | 18.4(6) | 5.9(5) | 7.3(5) | 6.2(5) |
| C5C | 27.3(7) | 25.8(7) | 17.8(6) | 7.0(5) | 9.8(5) | 11.8(5) |
| C22 | 24.7(7) | 22.8(6) | 25.3(6) | 6.5(5) | 14.4(5) | 6.7(5) |
| C7C | 22.4(6) | 23.7(6) | 22.0(6) | 4.2(5) | 8.2(5) | 7.4(5) |
| C26 | 21.9(6) | 26.3(7) | 23.8(6) | 2.8(5) | 11.9(5) | 7.6(5) |
| C11 | 22.9(6) | 18.4(6) | 23.9(6) | 4.7(5) | 8.8(5) | 6.5(5) |
| C12 | 28.7(7) | 19.0(6) | 20.8(6) | 2.5(5) | 8.5(5) | 6.5(5) |
| C30 | 21.5(6) | 25.3(7) | 24.8(6) | 6.8(5) | 11.0(5) | 9.3(5) |
| C4C | 28.3(7) | 24.8(7) | 23.3(6) | 7.4(5) | 12.4(5) | 12.1(5) |
| C13 | 31.3(7) | 20.5(6) | 22.1(6) | 5.1(5) | 13.5(6) | 9.7(5) |
| C19 | 25.4(7) | 26.6(7) | 26.1(7) | 12.4(5) | 11.7(5) | 11.5(5) |
| C4 | 22.9(6) | 27.2(7) | 18.8(6) | 5.7(5) | 10.5(5) | 4.8(5) |
| C8C | 25.7(7) | 24.1(7) | 19.8(6) | 4.3(5) | 6.9(5) | 8.1(5) |
| C21 | 29.4(7) | 21.1(6) | 34.0(7) | 5.9(6) | 19.1(6) | 7.6(5) |
| C29 | 27.9(7) | 33.8(8) | 27.6(7) | 10.0(6) | 14.3(6) | 17.2(6) |
| C3C | 34.1(7) | 23.7(7) | 21.6(6) | 8.1(5) | 12.6(6) | 12.2(6) |
| F13 | 104(6) | 56(4) | 46(5) | 33(3) | 56(5) | 50(5) |
| C8 | 27.3(7) | 31.9(7) | 26.5(7) | 5.4(6) | 14.1(6) | 10.4(6) |
| C10C | 26.7(7) | 28.5(7) | 29.8(7) | 3.4(6) | 12.9(6) | 2.9(6) |
| C9C | 29.6(7) | 22.0(6) | 26.9(7) | 1.6(5) | 10.5(6) | 4.9(6) |
| C1C | 38.2(8) | 34.5(8) | 28.6(7) | 12.7(6) | 18.8(6) | 23.4(7) |
| C27 | 20.3(7) | 36.6(8) | 25.4(7) | -1.4(6) | 10.2(5) | 8.1(6) |
| C20 | 28.6(7) | 20.8(6) | 37.9(8) | 12.4(6) | 18.0(6) | 10.2(5) |
| C15 | 26.4(7) | 24.9(7) | 31.4(7) | -0.8(6) | 10.8(6) | 4.1(6) |
| C2C | 43.0(8) | 26.5(7) | 26.4(7) | 10.0(6) | 18.2(6) | 19.8(6) |
| C15C | 27.8(7) | 24.2(7) | 34.5(8) | 1.7(6) | 11.1(6) | 10.5(6) |
| C11C | 38.5(8) | 21.5(7) | 35.0(8) | 5.9(6) | 17.2(7) | 9.9(6) |
| C7 | 28.1(7) | 39.2(8) | 25.6(7) | 16.8(6) | 10.5(6) | 9.6(6) |
| C28 | 23.6(7) | 44.7(9) | 26.0(7) | 4.9(6) | 10.4(6) | 18.1(6) |
| C16 | 42.1(9) | 27.2(7) | 29.8(7) | 3.5(6) | 21.0(7) | 10.5(6) |
| C24 | 46.8(9) | 21.5(7) | 47.5(9) | 5.0(6) | 31.7(8) | 5.7(7) |
| C23 | 49.1(9) | 30.9(8) | 32.6(8) | 17.6(6) | 19.5(7) | 14.4(7) |
| C12C | 46.0(9) | 28.2(7) | 28.6(7) | 6.4(6) | 9.4(7) | 12.3(7) |
| C31 | 15(2) | 33(2) | 30(3) | -5.1(18) | 8(2) | 9.8(16) |
| C32 | 29(2) | 27(2) | 35(3) | 10(2) | 8.8(18) | 19.8(18) |
| B1 | 18.0(6) | 19.0(6) | 18.1(6) | 4.9(5) | 8.1(5) | 6.1(5) |
| C13C | 64.7(12) | 30.1(8) | 36.8(9) | 10.5(7) | 26.9(8) | 8.5(8) |
| C17C | 44.9(10) | 53.5(11) | 38.0(9) | 5.5(8) | 19.9(8) | 24.8(9) |
| C14C | 33.2(9) | 27.7(8) | 79.3(14) | -4.0(8) | 24.3(9) | 2.7(7) |
| C18C | 61.3(12) | 52.9(11) | 58.2(12) | 25.5(9) | 29.1(10) | 38.7(10) |
| C16C | 31.2(9) | 35.3(9) | 97.8(17) | -9.5(10) | 26.5(10) | 9.3(7) |
| F1A | 73(6) | 54(5) | 65(6) | 46(4) | -4(4) | -16(4) |
| F2A | 51(5) | 88(9) | 58(7) | 50(6) | 28(5) | 48(5) |
| F3A | 115(7) | 89(5) | 23(3) | 25(3) | 22(4) | 68(5) |
| F1B | 56(4) | 17(2) | 26(3) | 13.0(18) | -9(2) | -9(2) |
| F3B | 50(5) | 97(9) | 32(4) | 43(5) | 27(4) | 39(5) |
| F2B | 14.9(18) | 31(3) | 27(5) | 16(3) | -2(2) | -2.1(18) |
| F5B | 55(7) | 75(7) | 50(4) | 30(4) | 42(4) | 35(5) |
| F6B | 22(2) | 46(3) | 75(7) | 0(4) | 4(3) | 14(2) |
| F4B | 32(5) | 36(2) | 39(5) | 16(3) | 14(4) | 22(3) |
| F6 | 55(6) | 84(5) | 55(4) | 46(4) | 38(4) | 53(4) |
| F4 | 32(4) | 39(4) | 31(2) | -5.3(19) | 20(2) | 9(2) |
| F5 | 18.4(18) | 48(3) | 48(4) | -3(2) | 8(2) | 12.9(19) |
| F20A | 23(2) | 34(3) | 75(5) | -4(2) | 9(3) | 5.9(17) |
| C31A | 24(3) | 53(4) | 35(3) | 3(2) | 5(2) | 17(2) |
| F21A | 30(2) | 65(4) | 81(4) | 1(3) | -15(3) | 17(2) |
| F19A | 65(4) | 89(5) | 52(3) | -11(3) | 34(3) | -35(3) |
| F13A | 54(6) | 80(7) | 42(5) | 15(5) | 34(4) | -1(5) |
| F14A | 141(10) | 63(5) | 42(5) | 29(3) | 61(6) | 43(6) |
| F15A | 67(4) | 49(6) | 19(5) | 7(4) | -1(3) | 13(4) |
| F14B | 77(4) | 68(5) | 52(5) | 37(4) | 53(4) | 48(4) |
| F15B | 54(3) | 95(9) | 33(3) | 8(4) | 21(2) | -6(4) |
| F13B | 62(7) | 37(3) | 56(4) | 32(3) | 39(4) | 22(3) |
| F17A | 131(6) | 27(2) | 29(2) | 3.7(16) | 35(3) | 11(4) |
| F16A | 39(3) | 19(3) | 52(4) | 12(3) | 18(3) | 2(2) |
| F18A | 75(4) | 87(5) | 87(4) | -21(3) | 45(3) | 41(3) |
| F10A | 44.0(18) | 42.6(18) | 47(2) | 1.5(14) | 32.8(15) | 12.8(14) |
| F11A | 41(2) | 68(4) | 36(3) | -19(3) | 18.0(18) | 0(3) |
| F12A | 111(5) | 56(2) | 69(3) | 42(2) | 76(3) | 51(3) |
| F24A | 122(6) | 58(4) | 55(4) | -7(3) | 8(3) | 64(4) |
| F23A | 90(5) | 61(5) | 173(8) | 76(5) | 83(5) | 54(4) |
| F22A | 68(4) | 56(5) | 71(4) | 9(2) | 1(2) | 46(3) |
| C32A | 42(4) | 54(4) | 53(5) | 11(3) | 20(3) | 28(3) |

Table 4 Bond Lengths for c031219\_2\_2.

| Atom | Atom | Length/Å |  | Atom | Atom | Length/Å |
| F8 | C15 | 1.3387(18) |  | C30 | C29 | 1.3917(19) |
| F17 | C24 | 1.317(6) |  | C4C | C3C | 1.3941(19) |
| F4A | C8 | 1.349(10) |  | C13 | C16 | 1.4920(19) |
| F20 | C31 | 1.334(7) |  | C19 | C20 | 1.383(2) |
| F7 | C15 | 1.3366(19) |  | C19 | C23 | 1.499(2) |
| F22 | C32 | 1.335(7) |  | C8C | C9C | 1.404(2) |
| F6A | C8 | 1.352(11) |  | C8C | C15C | 1.525(2) |
| F10 | C16 | 1.348(5) |  | C21 | C20 | 1.390(2) |
| F15 | C23 | 1.340(6) |  | C21 | C24 | 1.502(2) |
| F9 | C15 | 1.3321(18) |  | C29 | C28 | 1.387(2) |
| F5A | C8 | 1.347(11) |  | C29 | C32 | 1.534(7) |
| F24 | C32 | 1.350(8) |  | C29 | C32A | 1.439(10) |
| F19 | C31 | 1.328(7) |  | C3C | C2C | 1.395(2) |
| F12 | C16 | 1.276(5) |  | C3C | C11C | 1.526(2) |
| F21 | C31 | 1.331(7) |  | F13 | C23 | 1.343(6) |
| N2C | C6C | 1.3441(18) |  | C8 | F5B | 1.334(9) |
| N2C | C10C | 1.3435(19) |  | C8 | F6B | 1.349(9) |
| F18 | C24 | 1.301(6) |  | C8 | F4B | 1.374(9) |
| N1C | C5C | 1.3394(18) |  | C8 | F6 | 1.310(8) |
| N1C | C1C | 1.3353(19) |  | C8 | F4 | 1.335(7) |
| F23 | C32 | 1.340(7) |  | C8 | F5 | 1.364(7) |
| F2 | C7 | 1.280(7) |  | C10C | C9C | 1.366(2) |
| F16 | C24 | 1.382(6) |  | C1C | C2C | 1.385(2) |
| F14 | C23 | 1.337(6) |  | C27 | C28 | 1.385(2) |
| F1 | C7 | 1.382(5) |  | C27 | C31 | 1.496(7) |
| C9 | C10 | 1.4007(18) |  | C27 | C31A | 1.514(10) |
| C9 | C14 | 1.4051(18) |  | C15C | C17C | 1.537(2) |
| C9 | B1 | 1.6418(18) |  | C15C | C18C | 1.533(2) |
| C6C | C5C | 1.4807(19) |  | C15C | C16C | 1.525(2) |
| C6C | C7C | 1.380(2) |  | C11C | C12C | 1.534(2) |
| C2 | C1 | 1.3990(17) |  | C11C | C13C | 1.534(2) |
| C2 | C3 | 1.3951(18) |  | C11C | C14C | 1.529(2) |
| C25 | C26 | 1.3973(19) |  | C7 | F1A | 1.200(6) |
| C25 | C30 | 1.3998(19) |  | C7 | F2A | 1.303(9) |
| C25 | B1 | 1.6390(19) |  | C7 | F3A | 1.467(6) |
| F11 | C16 | 1.392(4) |  | C7 | F1B | 1.351(7) |
| C18 | C17 | 1.3974(18) |  | C7 | F3B | 1.308(8) |
| C18 | C19 | 1.3940(19) |  | C7 | F2B | 1.327(7) |
| C5 | C6 | 1.3902(18) |  | C16 | F10A | 1.398(6) |
| C5 | C4 | 1.3916(19) |  | C16 | F11A | 1.297(7) |
| C5 | C8 | 1.493(2) |  | C16 | F12A | 1.310(4) |
| C1 | C6 | 1.4015(18) |  | C24 | F17A | 1.376(7) |
| C1 | B1 | 1.6460(18) |  | C24 | F16A | 1.339(7) |
| C17 | C22 | 1.4045(19) |  | C24 | F18A | 1.281(7) |
| C17 | B1 | 1.6493(19) |  | C23 | F13A | 1.279(10) |
| C10 | C11 | 1.3947(18) |  | C23 | F14A | 1.352(8) |
| C14 | C13 | 1.3905(19) |  | C23 | F15A | 1.429(9) |
| C3 | C4 | 1.378(2) |  | C23 | F14B | 1.377(8) |
| C3 | C7 | 1.4974(19) |  | C23 | F15B | 1.290(9) |
| F3 | C7 | 1.337(6) |  | C23 | F13B | 1.353(9) |
| C5C | C4C | 1.387(2) |  | F20A | C31A | 1.329(10) |
| C22 | C21 | 1.3914(19) |  | C31A | F21A | 1.340(9) |
| C7C | C8C | 1.3922(19) |  | C31A | F19A | 1.339(10) |
| C26 | C27 | 1.389(2) |  | F24A | C32A | 1.331(10) |
| C11 | C12 | 1.3832(19) |  | F23A | C32A | 1.328(11) |
| C11 | C15 | 1.493(2) |  | F22A | C32A | 1.333(10) |
| C12 | C13 | 1.387(2) |  |  |  |  |

Table 5 Bond Angles for c031219\_2\_2.

| Atom | Atom | Atom | Angle/˚ |  | Atom | Atom | Atom | Angle/˚ |
| C10C | N2C | C6C | 122.84(13) |  | C16C | C15C | C17C | 108.65(16) |
| C1C | N1C | C5C | 116.27(13) |  | C16C | C15C | C18C | 109.11(16) |
| C10 | C9 | C14 | 115.24(12) |  | C3C | C11C | C12C | 108.87(13) |
| C10 | C9 | B1 | 123.49(11) |  | C3C | C11C | C13C | 109.06(13) |
| C14 | C9 | B1 | 121.26(11) |  | C3C | C11C | C14C | 111.80(12) |
| N2C | C6C | C5C | 115.13(12) |  | C12C | C11C | C13C | 109.64(13) |
| N2C | C6C | C7C | 118.34(12) |  | C14C | C11C | C12C | 108.30(15) |
| C7C | C6C | C5C | 126.53(12) |  | C14C | C11C | C13C | 109.14(15) |
| C3 | C2 | C1 | 122.18(12) |  | F2 | C7 | F1 | 105.3(5) |
| C26 | C25 | C30 | 115.79(12) |  | F2 | C7 | C3 | 115.6(6) |
| C26 | C25 | B1 | 122.97(12) |  | F2 | C7 | F3 | 108.5(5) |
| C30 | C25 | B1 | 120.96(11) |  | F1 | C7 | C3 | 110.6(3) |
| C19 | C18 | C17 | 122.30(12) |  | F3 | C7 | F1 | 102.5(5) |
| C6 | C5 | C4 | 120.55(13) |  | F3 | C7 | C3 | 113.3(4) |
| C6 | C5 | C8 | 120.51(12) |  | F1A | C7 | C3 | 116.9(4) |
| C4 | C5 | C8 | 118.93(12) |  | F1A | C7 | F2A | 116.7(8) |
| C2 | C1 | C6 | 115.47(11) |  | F1A | C7 | F3A | 104.4(6) |
| C2 | C1 | B1 | 123.31(11) |  | F2A | C7 | C3 | 112.6(7) |
| C6 | C1 | B1 | 121.21(11) |  | F2A | C7 | F3A | 98.5(5) |
| C18 | C17 | C22 | 115.61(12) |  | F3A | C7 | C3 | 104.8(3) |
| C18 | C17 | B1 | 121.69(11) |  | F1B | C7 | C3 | 111.8(4) |
| C22 | C17 | B1 | 122.25(11) |  | F3B | C7 | C3 | 114.9(6) |
| C11 | C10 | C9 | 122.35(12) |  | F3B | C7 | F1B | 106.4(6) |
| C13 | C14 | C9 | 122.46(12) |  | F3B | C7 | F2B | 106.6(6) |
| C2 | C3 | C7 | 118.72(13) |  | F2B | C7 | C3 | 112.7(5) |
| C4 | C3 | C2 | 121.14(12) |  | F2B | C7 | F1B | 103.7(6) |
| C4 | C3 | C7 | 120.14(12) |  | C27 | C28 | C29 | 117.92(13) |
| C5 | C6 | C1 | 122.61(12) |  | F10 | C16 | F11 | 100.5(4) |
| N1C | C5C | C6C | 114.08(12) |  | F10 | C16 | C13 | 112.5(4) |
| N1C | C5C | C4C | 123.98(13) |  | F12 | C16 | F10 | 107.1(5) |
| C4C | C5C | C6C | 121.91(12) |  | F12 | C16 | F11 | 108.8(3) |
| C21 | C22 | C17 | 122.22(13) |  | F12 | C16 | C13 | 117.3(3) |
| C6C | C7C | C8C | 121.54(13) |  | F11 | C16 | C13 | 109.2(2) |
| C27 | C26 | C25 | 122.11(13) |  | F10A | C16 | C13 | 109.5(4) |
| C10 | C11 | C15 | 119.19(12) |  | F11A | C16 | C13 | 116.1(6) |
| C12 | C11 | C10 | 121.15(13) |  | F11A | C16 | F10A | 104.9(5) |
| C12 | C11 | C15 | 119.65(12) |  | F11A | C16 | F12A | 110.7(5) |
| C11 | C12 | C13 | 117.74(12) |  | F12A | C16 | C13 | 114.1(3) |
| C29 | C30 | C25 | 122.28(13) |  | F12A | C16 | F10A | 99.8(5) |
| C5C | C4C | C3C | 119.79(13) |  | F17 | C24 | F16 | 104.2(4) |
| C14 | C13 | C16 | 120.26(13) |  | F17 | C24 | C21 | 112.9(3) |
| C12 | C13 | C14 | 121.03(12) |  | F18 | C24 | F17 | 109.0(5) |
| C12 | C13 | C16 | 118.63(12) |  | F18 | C24 | F16 | 106.1(5) |
| C18 | C19 | C23 | 118.84(13) |  | F18 | C24 | C21 | 113.9(5) |
| C20 | C19 | C18 | 121.08(13) |  | F16 | C24 | C21 | 110.1(3) |
| C20 | C19 | C23 | 120.06(13) |  | F17A | C24 | C21 | 111.6(6) |
| C3 | C4 | C5 | 118.03(12) |  | F16A | C24 | C21 | 112.3(4) |
| C7C | C8C | C9C | 117.00(13) |  | F16A | C24 | F17A | 102.5(6) |
| C7C | C8C | C15C | 122.51(13) |  | F18A | C24 | C21 | 114.2(5) |
| C9C | C8C | C15C | 120.47(12) |  | F18A | C24 | F17A | 106.8(6) |
| C22 | C21 | C24 | 120.90(13) |  | F18A | C24 | F16A | 108.7(6) |
| C20 | C21 | C22 | 120.93(13) |  | F15 | C23 | C19 | 112.9(4) |
| C20 | C21 | C24 | 118.17(13) |  | F15 | C23 | F13 | 106.1(5) |
| C30 | C29 | C32 | 117.9(3) |  | F14 | C23 | F15 | 106.6(5) |
| C30 | C29 | C32A | 120.0(5) |  | F14 | C23 | C19 | 112.5(4) |
| C28 | C29 | C30 | 120.70(14) |  | F14 | C23 | F13 | 105.7(5) |
| C28 | C29 | C32 | 121.3(3) |  | F13 | C23 | C19 | 112.4(4) |
| C28 | C29 | C32A | 119.1(5) |  | F13A | C23 | C19 | 122.7(8) |
| C4C | C3C | C2C | 115.97(13) |  | F13A | C23 | F14A | 110.6(8) |
| C4C | C3C | C11C | 122.48(13) |  | F13A | C23 | F15A | 104.2(8) |
| C2C | C3C | C11C | 121.53(13) |  | F14A | C23 | C19 | 113.7(5) |
| F4A | C8 | F6A | 105.6(8) |  | F14A | C23 | F15A | 98.3(6) |
| F4A | C8 | C5 | 112.4(7) |  | F15A | C23 | C19 | 103.4(6) |
| F6A | C8 | C5 | 112.6(9) |  | F14B | C23 | C19 | 107.7(5) |
| F5A | C8 | F4A | 104.9(10) |  | F15B | C23 | C19 | 119.3(7) |
| F5A | C8 | F6A | 105.5(10) |  | F15B | C23 | F14B | 108.8(7) |
| F5A | C8 | C5 | 115.1(10) |  | F15B | C23 | F13B | 108.4(8) |
| F5B | C8 | C5 | 117.9(7) |  | F13B | C23 | C19 | 108.9(7) |
| F5B | C8 | F6B | 106.3(8) |  | F13B | C23 | F14B | 102.5(7) |
| F5B | C8 | F4B | 104.1(8) |  | F20 | C31 | C27 | 110.5(7) |
| F6B | C8 | C5 | 114.6(8) |  | F19 | C31 | F20 | 108.0(6) |
| F6B | C8 | F4B | 103.9(8) |  | F19 | C31 | F21 | 108.0(6) |
| F4B | C8 | C5 | 108.7(8) |  | F19 | C31 | C27 | 108.7(5) |
| F6 | C8 | C5 | 116.4(7) |  | F21 | C31 | F20 | 106.2(6) |
| F6 | C8 | F4 | 110.3(7) |  | F21 | C31 | C27 | 115.3(6) |
| F6 | C8 | F5 | 107.6(6) |  | F22 | C32 | F24 | 106.3(6) |
| F4 | C8 | C5 | 108.7(6) |  | F22 | C32 | F23 | 106.3(6) |
| F4 | C8 | F5 | 105.1(7) |  | F22 | C32 | C29 | 113.3(7) |
| F5 | C8 | C5 | 108.1(7) |  | F24 | C32 | C29 | 113.9(7) |
| N2C | C10C | C9C | 119.84(13) |  | F23 | C32 | F24 | 104.4(6) |
| C10C | C9C | C8C | 120.44(13) |  | F23 | C32 | C29 | 111.9(7) |
| N1C | C1C | C2C | 123.59(14) |  | C9 | B1 | C1 | 110.65(10) |
| C26 | C27 | C31 | 123.3(4) |  | C9 | B1 | C17 | 110.59(10) |
| C26 | C27 | C31A | 113.2(5) |  | C25 | B1 | C9 | 109.51(10) |
| C28 | C27 | C26 | 121.12(13) |  | C25 | B1 | C1 | 108.25(10) |
| C28 | C27 | C31 | 115.5(4) |  | C25 | B1 | C17 | 107.83(10) |
| C28 | C27 | C31A | 125.3(5) |  | C1 | B1 | C17 | 109.93(10) |
| C19 | C20 | C21 | 117.84(13) |  | F20A | C31A | C27 | 116.2(10) |
| F8 | C15 | C11 | 112.66(12) |  | F20A | C31A | F21A | 105.6(9) |
| F7 | C15 | F8 | 105.55(13) |  | F20A | C31A | F19A | 104.6(9) |
| F7 | C15 | C11 | 112.04(13) |  | F21A | C31A | C27 | 109.3(8) |
| F9 | C15 | F8 | 106.69(13) |  | F19A | C31A | C27 | 115.4(8) |
| F9 | C15 | F7 | 106.21(13) |  | F19A | C31A | F21A | 104.7(8) |
| F9 | C15 | C11 | 113.13(13) |  | F24A | C32A | C29 | 110.6(10) |
| C1C | C2C | C3C | 120.36(13) |  | F24A | C32A | F22A | 105.4(9) |
| C8C | C15C | C17C | 108.22(12) |  | F23A | C32A | C29 | 113.4(10) |
| C8C | C15C | C18C | 109.46(14) |  | F23A | C32A | F24A | 106.0(9) |
| C8C | C15C | C16C | 112.33(12) |  | F23A | C32A | F22A | 107.5(9) |
| C18C | C15C | C17C | 109.01(14) |  | F22A | C32A | C29 | 113.4(10) |

Table 6 Torsion Angles for c031219\_2\_2.

| A | B | C | D | Angle/˚ |  | A | B | C | D | Angle/˚ |
| N2C | C6C | C5C | N1C | -4.39(16) |  | C26 | C25 | B1 | C17 | 21.69(16) |
| N2C | C6C | C5C | C4C | 173.72(12) |  | C26 | C27 | C28 | C29 | 1.6(2) |
| N2C | C6C | C7C | C8C | 0.22(19) |  | C26 | C27 | C31 | F20 | 25.4(7) |
| N2C | C10C | C9C | C8C | -0.6(2) |  | C26 | C27 | C31 | F19 | -92.9(5) |
| N1C | C5C | C4C | C3C | 1.0(2) |  | C26 | C27 | C31 | F21 | 145.8(4) |
| N1C | C1C | C2C | C3C | 0.0(2) |  | C26 | C27 | C31A | F20A | 35.7(10) |
| C9 | C10 | C11 | C12 | -0.1(2) |  | C26 | C27 | C31A | F21A | 155.0(7) |
| C9 | C10 | C11 | C15 | 178.70(12) |  | C26 | C27 | C31A | F19A | -87.3(9) |
| C9 | C14 | C13 | C12 | -1.4(2) |  | C11 | C12 | C13 | C14 | 0.3(2) |
| C9 | C14 | C13 | C16 | 175.45(13) |  | C11 | C12 | C13 | C16 | -176.53(13) |
| C6C | N2C | C10C | C9C | -0.2(2) |  | C12 | C11 | C15 | F8 | -139.21(14) |
| C6C | C5C | C4C | C3C | -176.90(12) |  | C12 | C11 | C15 | F7 | 101.93(15) |
| C6C | C7C | C8C | C9C | -1.0(2) |  | C12 | C11 | C15 | F9 | -18.1(2) |
| C6C | C7C | C8C | C15C | 177.20(13) |  | C12 | C13 | C16 | F10 | -36.9(4) |
| C2 | C1 | C6 | C5 | 1.10(18) |  | C12 | C13 | C16 | F12 | -161.9(5) |
| C2 | C1 | B1 | C9 | 102.53(13) |  | C12 | C13 | C16 | F11 | 73.7(4) |
| C2 | C1 | B1 | C25 | -17.47(16) |  | C12 | C13 | C16 | F10A | -143.6(3) |
| C2 | C1 | B1 | C17 | -135.02(12) |  | C12 | C13 | C16 | F11A | -25.0(6) |
| C2 | C3 | C4 | C5 | 0.8(2) |  | C12 | C13 | C16 | F12A | 105.6(5) |
| C2 | C3 | C7 | F2 | 72.5(5) |  | C30 | C25 | C26 | C27 | -1.73(19) |
| C2 | C3 | C7 | F1 | -47.0(4) |  | C30 | C25 | B1 | C9 | -44.33(15) |
| C2 | C3 | C7 | F3 | -161.4(4) |  | C30 | C25 | B1 | C1 | 76.38(14) |
| C2 | C3 | C7 | F1A | -109.5(9) |  | C30 | C25 | B1 | C17 | -164.72(11) |
| C2 | C3 | C7 | F2A | 29.5(6) |  | C30 | C29 | C28 | C27 | -0.2(2) |
| C2 | C3 | C7 | F3A | 135.5(6) |  | C30 | C29 | C32 | F22 | -158.4(5) |
| C2 | C3 | C7 | F1B | -63.2(5) |  | C30 | C29 | C32 | F24 | 79.9(6) |
| C2 | C3 | C7 | F3B | 175.5(5) |  | C30 | C29 | C32 | F23 | -38.2(7) |
| C2 | C3 | C7 | F2B | 53.2(6) |  | C30 | C29 | C32A | F24A | 76.9(10) |
| C25 | C26 | C27 | C28 | -0.6(2) |  | C30 | C29 | C32A | F23A | -42.1(11) |
| C25 | C26 | C27 | C31 | -178.9(4) |  | C30 | C29 | C32A | F22A | -165.1(7) |
| C25 | C26 | C27 | C31A | 173.3(5) |  | C4C | C3C | C2C | C1C | 1.1(2) |
| C25 | C30 | C29 | C28 | -2.3(2) |  | C4C | C3C | C11C | C12C | -125.39(14) |
| C25 | C30 | C29 | C32 | -179.1(4) |  | C4C | C3C | C11C | C13C | 115.01(15) |
| C25 | C30 | C29 | C32A | 172.8(6) |  | C4C | C3C | C11C | C14C | -5.8(2) |
| C18 | C17 | C22 | C21 | 0.5(2) |  | C19 | C18 | C17 | C22 | 0.81(19) |
| C18 | C17 | B1 | C9 | -36.89(16) |  | C19 | C18 | C17 | B1 | -171.66(12) |
| C18 | C17 | B1 | C25 | 82.82(14) |  | C4 | C5 | C6 | C1 | -1.7(2) |
| C18 | C17 | B1 | C1 | -159.37(11) |  | C4 | C5 | C8 | F4A | 35.0(9) |
| C18 | C19 | C20 | C21 | 1.0(2) |  | C4 | C5 | C8 | F6A | 154.0(10) |
| C18 | C19 | C23 | F15 | -161.4(5) |  | C4 | C5 | C8 | F5A | -84.9(11) |
| C18 | C19 | C23 | F14 | 77.8(5) |  | C4 | C5 | C8 | F5B | 24.8(7) |
| C18 | C19 | C23 | F13 | -41.4(4) |  | C4 | C5 | C8 | F6B | -101.4(7) |
| C18 | C19 | C23 | F13A | -145.5(9) |  | C4 | C5 | C8 | F4B | 142.9(7) |
| C18 | C19 | C23 | F14A | -7.9(6) |  | C4 | C5 | C8 | F6 | 165.9(6) |
| C18 | C19 | C23 | F15A | 97.6(5) |  | C4 | C5 | C8 | F4 | 40.7(6) |
| C18 | C19 | C23 | F14B | -64.8(4) |  | C4 | C5 | C8 | F5 | -72.9(6) |
| C18 | C19 | C23 | F15B | 59.7(8) |  | C4 | C3 | C7 | F2 | -106.6(5) |
| C18 | C19 | C23 | F13B | -175.2(6) |  | C4 | C3 | C7 | F1 | 133.9(3) |
| C1 | C2 | C3 | C4 | -1.4(2) |  | C4 | C3 | C7 | F3 | 19.5(4) |
| C1 | C2 | C3 | C7 | 179.47(12) |  | C4 | C3 | C7 | F1A | 71.4(9) |
| C17 | C18 | C19 | C20 | -1.6(2) |  | C4 | C3 | C7 | F2A | -149.6(6) |
| C17 | C18 | C19 | C23 | 176.81(13) |  | C4 | C3 | C7 | F3A | -43.6(6) |
| C17 | C22 | C21 | C20 | -1.1(2) |  | C4 | C3 | C7 | F1B | 117.7(5) |
| C17 | C22 | C21 | C24 | 178.90(14) |  | C4 | C3 | C7 | F3B | -3.6(6) |
| C10 | C9 | C14 | C13 | 1.55(19) |  | C4 | C3 | C7 | F2B | -126.0(6) |
| C10 | C9 | B1 | C25 | 140.61(12) |  | C8 | C5 | C6 | C1 | 177.13(12) |
| C10 | C9 | B1 | C1 | 21.37(16) |  | C8 | C5 | C4 | C3 | -178.15(12) |
| C10 | C9 | B1 | C17 | -100.69(14) |  | C10C | N2C | C6C | C5C | -179.61(12) |
| C10 | C11 | C12 | C13 | 0.4(2) |  | C10C | N2C | C6C | C7C | 0.5(2) |
| C10 | C11 | C15 | F8 | 41.97(18) |  | C9C | C8C | C15C | C17C | 63.68(18) |
| C10 | C11 | C15 | F7 | -76.89(16) |  | C9C | C8C | C15C | C18C | -55.02(19) |
| C10 | C11 | C15 | F9 | 163.08(13) |  | C9C | C8C | C15C | C16C | -176.38(16) |
| C14 | C9 | C10 | C11 | -0.83(18) |  | C1C | N1C | C5C | C6C | 178.27(11) |
| C14 | C9 | B1 | C25 | -40.65(15) |  | C1C | N1C | C5C | C4C | 0.20(19) |
| C14 | C9 | B1 | C1 | -159.89(11) |  | C20 | C19 | C23 | F15 | 17.0(5) |
| C14 | C9 | B1 | C17 | 78.05(14) |  | C20 | C19 | C23 | F14 | -103.8(5) |
| C14 | C13 | C16 | F10 | 146.2(4) |  | C20 | C19 | C23 | F13 | 137.0(4) |
| C14 | C13 | C16 | F12 | 21.2(6) |  | C20 | C19 | C23 | F13A | 32.9(9) |
| C14 | C13 | C16 | F11 | -103.1(4) |  | C20 | C19 | C23 | F14A | 170.5(6) |
| C14 | C13 | C16 | F10A | 39.6(3) |  | C20 | C19 | C23 | F15A | -84.0(5) |
| C14 | C13 | C16 | F11A | 158.1(5) |  | C20 | C19 | C23 | F14B | 113.6(4) |
| C14 | C13 | C16 | F12A | -71.3(6) |  | C20 | C19 | C23 | F15B | -121.9(8) |
| C3 | C2 | C1 | C6 | 0.43(18) |  | C20 | C19 | C23 | F13B | 3.2(7) |
| C3 | C2 | C1 | B1 | 179.55(12) |  | C20 | C21 | C24 | F17 | -45.8(4) |
| C6 | C5 | C4 | C3 | 0.69(19) |  | C20 | C21 | C24 | F18 | -170.9(5) |
| C6 | C5 | C8 | F4A | -143.8(9) |  | C20 | C21 | C24 | F16 | 70.2(4) |
| C6 | C5 | C8 | F6A | -24.8(10) |  | C20 | C21 | C24 | F17A | -156.6(6) |
| C6 | C5 | C8 | F5A | 96.2(11) |  | C20 | C21 | C24 | F16A | -42.2(5) |
| C6 | C5 | C8 | F5B | -154.1(7) |  | C20 | C21 | C24 | F18A | 82.1(7) |
| C6 | C5 | C8 | F6B | 79.7(7) |  | C15 | C11 | C12 | C13 | -178.43(13) |
| C6 | C5 | C8 | F4B | -36.0(7) |  | C2C | C3C | C11C | C12C | 53.38(18) |
| C6 | C5 | C8 | F6 | -12.9(6) |  | C2C | C3C | C11C | C13C | -66.21(18) |
| C6 | C5 | C8 | F4 | -138.1(6) |  | C2C | C3C | C11C | C14C | 173.00(15) |
| C6 | C5 | C8 | F5 | 108.3(6) |  | C15C | C8C | C9C | C10C | -177.04(14) |
| C6 | C1 | B1 | C9 | -78.39(14) |  | C11C | C3C | C2C | C1C | -177.70(13) |
| C6 | C1 | B1 | C25 | 161.61(11) |  | C7 | C3 | C4 | C5 | 179.92(13) |
| C6 | C1 | B1 | C17 | 44.05(15) |  | C28 | C29 | C32 | F22 | 24.9(8) |
| C5C | N1C | C1C | C2C | -0.7(2) |  | C28 | C29 | C32 | F24 | -96.8(6) |
| C5C | C6C | C7C | C8C | -179.72(12) |  | C28 | C29 | C32 | F23 | 145.1(5) |
| C5C | C4C | C3C | C2C | -1.64(19) |  | C28 | C29 | C32A | F24A | -107.9(9) |
| C5C | C4C | C3C | C11C | 177.20(13) |  | C28 | C29 | C32A | F23A | 133.2(8) |
| C22 | C17 | B1 | C9 | 151.14(12) |  | C28 | C29 | C32A | F22A | 10.2(12) |
| C22 | C17 | B1 | C25 | -89.15(14) |  | C28 | C27 | C31 | F20 | -152.9(5) |
| C22 | C17 | B1 | C1 | 28.66(16) |  | C28 | C27 | C31 | F19 | 88.8(5) |
| C22 | C21 | C20 | C19 | 0.4(2) |  | C28 | C27 | C31 | F21 | -32.5(7) |
| C22 | C21 | C24 | F17 | 134.1(4) |  | C28 | C27 | C31A | F20A | -150.6(7) |
| C22 | C21 | C24 | F18 | 9.1(5) |  | C28 | C27 | C31A | F21A | -31.3(10) |
| C22 | C21 | C24 | F16 | -109.9(4) |  | C28 | C27 | C31A | F19A | 86.4(8) |
| C22 | C21 | C24 | F17A | 23.3(6) |  | C24 | C21 | C20 | C19 | -179.66(14) |
| C22 | C21 | C24 | F16A | 137.8(4) |  | C23 | C19 | C20 | C21 | -177.42(14) |
| C22 | C21 | C24 | F18A | -97.9(6) |  | C31 | C27 | C28 | C29 | 180.0(3) |
| C7C | C6C | C5C | N1C | 175.54(13) |  | C32 | C29 | C28 | C27 | 176.5(4) |
| C7C | C6C | C5C | C4C | -6.3(2) |  | B1 | C9 | C10 | C11 | 177.98(12) |
| C7C | C8C | C9C | C10C | 1.2(2) |  | B1 | C9 | C14 | C13 | -177.29(12) |
| C7C | C8C | C15C | C17C | -114.49(15) |  | B1 | C25 | C26 | C27 | 172.17(12) |
| C7C | C8C | C15C | C18C | 126.82(15) |  | B1 | C25 | C30 | C29 | -170.84(12) |
| C7C | C8C | C15C | C16C | 5.5(2) |  | B1 | C1 | C6 | C5 | -178.05(12) |
| C26 | C25 | C30 | C29 | 3.19(19) |  | B1 | C17 | C22 | C21 | 172.95(13) |
| C26 | C25 | B1 | C9 | 142.08(12) |  | C31A | C27 | C28 | C29 | -171.6(5) |
| C26 | C25 | B1 | C1 | -97.21(14) |  | C32A | C29 | C28 | C27 | -175.4(6) |

Table 7 Hydrogen Atom Coordinates (Å×104) and Isotropic Displacement Parameters (Å2×103) for c031219\_2\_2.

| Atom | *x* | *y* | *z* | U(eq) |
| H2C | 5611(14) | 2039(14) | 2617(11) | 31 |
| H2 | 6580.84 | 2480.21 | 8900.95 | 24 |
| H18 | 6302.22 | 4438.73 | 6161.5 | 26 |
| H10 | 3693.72 | 2092.48 | 6713.07 | 25 |
| H14 | 6420.11 | 2623.21 | 6019.7 | 27 |
| H6 | 4402.64 | 3979.25 | 7824.76 | 24 |
| H22 | 6627.91 | 5473.77 | 8605.89 | 28 |
| H7C | 8739.11 | 2495.94 | 2652.34 | 28 |
| H26 | 8532.15 | 4912.06 | 8311.19 | 29 |
| H12 | 3130.99 | 506.4 | 4302.54 | 29 |
| H30 | 6755.48 | 1617.96 | 7478.23 | 28 |
| H4C | 9176.29 | 4262.14 | 3282.75 | 29 |
| H4 | 4347.71 | 2909.45 | 9938.18 | 29 |
| H10C | 4965.18 | 133.67 | 2091.72 | 37 |
| H9C | 6245.14 | -593.1 | 1829.93 | 34 |
| H1C | 6021.51 | 4991.24 | 3427.61 | 35 |
| H20 | 7340.38 | 7696.71 | 7253.68 | 33 |
| H2CA | 7831.07 | 6432.38 | 3939.86 | 35 |
| H28 | 10306.07 | 2900.88 | 8893.97 | 37 |
| H12A | 9730.93 | 7396.33 | 5016.95 | 55 |
| H12B | 11080.6 | 7630.66 | 5261.15 | 55 |
| H12C | 10260.42 | 6534.1 | 5352.37 | 55 |
| H13A | 9738.4 | 6928.52 | 2906.05 | 67 |
| H13B | 10699.78 | 7901.52 | 3734.83 | 67 |
| H13C | 9340.99 | 7585.52 | 3507.25 | 67 |
| H17A | 7153.07 | -691.42 | 803.93 | 66 |
| H17B | 8429.81 | -430.32 | 830.69 | 66 |
| H17C | 7843.01 | 446.1 | 683.55 | 66 |
| H14A | 10965.83 | 5518.76 | 4409.49 | 76 |
| H14B | 11703.97 | 6655.63 | 4315.95 | 76 |
| H14C | 10772.29 | 5688.71 | 3473.55 | 76 |
| H18A | 8961.33 | -189.08 | 3071.83 | 76 |
| H18B | 9085.35 | -836.54 | 2283.93 | 76 |
| H18C | 7822.13 | -1071.15 | 2277.42 | 76 |
| H16A | 9647.51 | 1849.83 | 1951.24 | 88 |
| H16B | 10203.59 | 951.54 | 2096.05 | 88 |
| H16C | 10073.27 | 1613.03 | 2874.16 | 88 |

Table 8 Atomic Occupancy for c031219\_2\_2.

| Atom | *Occupancy* |  | Atom | *Occupancy* |  | Atom | *Occupancy* |
| F17 | 0.544(13) |  | F4A | 0.222(3) |  | F20 | 0.544(13) |
| F22 | 0.544(13) |  | F6A | 0.222(3) |  | F10 | 0.544(13) |
| F15 | 0.453(3) |  | F5A | 0.222(3) |  | F24 | 0.544(13) |
| F19 | 0.544(13) |  | F12 | 0.544(13) |  | F21 | 0.544(13) |
| F18 | 0.544(13) |  | F23 | 0.544(13) |  | F2 | 0.453(3) |
| F16 | 0.544(13) |  | F14 | 0.453(3) |  | F1 | 0.453(3) |
| F11 | 0.544(13) |  | F3 | 0.453(3) |  | F13 | 0.453(3) |
| C31 | 0.544(13) |  | C32 | 0.544(13) |  | F1A | 0.222(3) |
| F2A | 0.222(3) |  | F3A | 0.222(3) |  | F1B | 0.325(3) |
| F3B | 0.325(3) |  | F2B | 0.325(3) |  | F5B | 0.325(3) |
| F6B | 0.325(3) |  | F4B | 0.325(3) |  | F6 | 0.453(3) |
| F4 | 0.453(3) |  | F5 | 0.453(3) |  | F20A | 0.456(13) |
| C31A | 0.456(13) |  | F21A | 0.456(13) |  | F19A | 0.456(13) |
| F13A | 0.222(3) |  | F14A | 0.222(3) |  | F15A | 0.222(3) |
| F14B | 0.325(3) |  | F15B | 0.325(3) |  | F13B | 0.325(3) |
| F17A | 0.456(13) |  | F16A | 0.456(13) |  | F18A | 0.456(13) |
| F10A | 0.456(13) |  | F11A | 0.456(13) |  | F12A | 0.456(13) |
| F24A | 0.456(13) |  | F23A | 0.456(13) |  | F22A | 0.456(13) |
| C32A | 0.456(13) |  |  |  |  |  |

Experimental

Single crystals of C50H37BF24N2
[c031219\_2\_2]
were
[].
A suitable crystal was selected and
[]
on a
XtaLAB Synergy, Dualflex, Pilatus 300K
diffractometer. The crystal was kept at 100.0(1) K during data collection.
Using Olex2 [1], the structure was solved with the
SHELXT
[2] structure solution program using
Intrinsic Phasing
and refined with the
SHELXL
[3] refinement package using
Least Squares
minimisation.

1. Dolomanov, O.V., Bourhis, L.J., Gildea, R.J, Howard, J.A.K. & Puschmann, H.
   (2009), J. Appl. Cryst. 42, 339-341.
2. Sheldrick, G.M. (2015). Acta Cryst. A71, 3-8.
3. Sheldrick, G.M. (2015). Acta Cryst. C71, 3-8.

Crystal structure determination of
[c031219\_2\_2]

**Crystal Data**
for C50H37BF24N2 (*M*=1132.62 g/mol):
triclinic, space group P-1 (no. 2),
*a* = 12.98390(10) Å, *b* = 13.14960(10) Å, *c* = 16.88170(10) Å, *α* = 97.5750(10)°, *β* = 110.6430(10)°, *γ* = 108.0880(10)°,
*V*= 2467.80(4) Å3,
*Z* = 2,
*T* = 100.0(1) K,
μ(Cu Kα) = 1.366 mm-1,
*Dcalc* = 1.524 g/cm3,
68819 reflections measured (5.812° ≤ 2Θ ≤ 160.502°),
10503 unique (*R*int = 0.0383, Rsigma = 0.0209) which were used in all calculations.
The final *R*1 was 0.0389
(I > 2σ(I)) and *wR*2 was 0.1037 (all data).

Refinement model description

Number of restraints - 2008,
number of constraints - unknown.

Details:

```
1. Fixed Uiso
```

This report has been created with Olex2, compiled on
Nov 21 2019 18:26:39 for OlexSys. Please
let us know
if there are any errors or if you would like to have additional features.
