# Supplementary material for: London Dispersion versus Intramolecular Hydrogen Bond in Bis‐Pyridines: How Accurate Is DFT for Competing Noncovalent Interactions in the Condensed Phase?
Source: Chemistry. 2025 Oct 23;31(66):e02745. doi: 10.1002/chem.202502745 (PMC12648470; doi:10.1002/chem.202502745)
Supplement: Supplementary file 1 — Supporting Information [file CHEM-31-e02745-s002.zip › Crystal_structures/4b/c080620_3_2_tables.html]

c080620\_3\_2


# c080620\_3\_2

b"\n \n \n "

Table 1 Crystal data and structure refinement for c080620\_3\_2.

| Identification code | c080620\_3\_2 |
| Empirical formula | C46H25BF24N2 |
| Formula weight | 1072.49 |
| Temperature/K | 100.0(1) |
| Crystal system | triclinic |
| Space group | P-1 |
| a/Å | 12.3378(2) |
| b/Å | 13.3787(2) |
| c/Å | 13.4037(2) |
| α/° | 92.0660(10) |
| β/° | 104.8350(10) |
| γ/° | 90.4690(10) |
| Volume/Å3 | 2136.98(6) |
| Z | 2 |
| ρcalcg/cm3 | 1.667 |
| μ/mm‑1 | 1.543 |
| F(000) | 1072.0 |
| Crystal size/mm3 | 0.327 × 0.279 × 0.092 |
| Radiation | Cu Kα (λ = 1.54184) |
| 2Θ range for data collection/° | 6.828 to 146.442 |
| Index ranges | -15 ≤ h ≤ 14, -15 ≤ k ≤ 16, -16 ≤ l ≤ 16 |
| Reflections collected | 56609 |
| Independent reflections | 8190 [Rint = 0.0442, Rsigma = 0.0242] |
| Data/restraints/parameters | 8190/860/784 |
| Goodness-of-fit on F2 | 1.035 |
| Final R indexes [I>=2σ (I)] | R1 = 0.0341, wR2 = 0.0858 |
| Final R indexes [all data] | R1 = 0.0385, wR2 = 0.0884 |
| Largest diff. peak/hole / e Å-3 | 0.29/-0.29 |

Table 2 Fractional Atomic Coordinates (×104) and Equivalent Isotropic Displacement Parameters (Å2×103) for c080620\_3\_2. Ueq is defined as 1/3 of the trace of the orthogonalised UIJ tensor.

| Atom | *x* | *y* | *z* | U(eq) |
| --- | --- | --- | --- | --- |
| F1 | 8555(7) | 8816(5) | 9993(5) | 59(2) |
| F2 | 8849(8) | 10304(6) | 9564(7) | 58(3) |
| F3 | 8672(7) | 9075(6) | 8489(5) | 43.4(14) |
| F4 | 3900.8(8) | 11459.1(7) | 9218.5(8) | 31.5(2) |
| F5 | 5551.1(8) | 12079.9(7) | 9885.2(7) | 32.4(2) |
| F6 | 4791.9(9) | 12192.5(7) | 8269.3(7) | 33.3(2) |
| F7 | 1634(6) | 8020(8) | 3526(8) | 47.3(19) |
| F8 | 2654(11) | 7823(9) | 2470(7) | 50(3) |
| F9 | 2306(8) | 9294(5) | 2952(7) | 33.0(14) |
| F10 | 7333.7(8) | 7898.1(7) | 4895.7(8) | 32.5(2) |
| F11 | 6652.0(8) | 8820.9(8) | 3622.0(7) | 32.1(2) |
| F12 | 7258.5(8) | 9480.5(7) | 5148.8(8) | 33.7(2) |
| F13 | 4046.3(8) | 3590.3(6) | 6554.6(7) | 32.7(2) |
| F14 | 5567.0(8) | 3874.2(7) | 6097.3(7) | 32.6(2) |
| F15 | 4040.6(8) | 4584.3(6) | 5326.9(7) | 30.2(2) |
| F16 | 6317.2(8) | 4589.0(7) | 10111.6(7) | 34.0(2) |
| F17 | 7509.5(8) | 5739.9(7) | 10007.7(7) | 31.7(2) |
| F18 | 6080.1(9) | 6109.9(7) | 10566.4(7) | 33.8(2) |
| F19 | 1055(8) | 10494(6) | 6273(7) | 59(2) |
| F20 | 266(6) | 10410(5) | 7497(4) | 38.5(13) |
| F21 | -338(7) | 9517(7) | 6121(8) | 48.0(17) |
| F22 | 2040.3(9) | 7118.3(7) | 10019.3(7) | 37.3(2) |
| F23 | 328.0(9) | 7076.9(8) | 9104.5(9) | 43.0(3) |
| F24 | 1490.1(8) | 5993.2(6) | 8812.0(7) | 27.4(2) |
| C1 | 5185.6(12) | 8940.2(10) | 7982.3(10) | 16.3(3) |
| C2 | 6354.1(12) | 8860.8(10) | 8325.0(11) | 18.3(3) |
| C3 | 7036.6(12) | 9616.9(11) | 8907.0(11) | 19.6(3) |
| C4 | 6585.3(12) | 10501.9(11) | 9175.6(11) | 19.7(3) |
| C5 | 5433.7(12) | 10602.5(10) | 8845.6(10) | 17.6(3) |
| C6 | 4751.3(12) | 9838.7(10) | 8273.8(10) | 17.0(3) |
| C7 | 8270.1(13) | 9472.8(12) | 9271.7(13) | 27.6(3) |
| C8 | 4928.3(12) | 11573.4(11) | 9062.3(11) | 19.9(3) |
| C9 | 4429.5(12) | 8270.9(9) | 6030.4(10) | 16.8(3) |
| C10 | 3491.5(12) | 8231.5(10) | 5178.8(11) | 18.1(3) |
| C11 | 3573.3(12) | 8373.6(10) | 4177.8(11) | 19.5(3) |
| C12 | 4601.3(12) | 8551.9(10) | 3973.1(11) | 19.0(3) |
| C13 | 5547.0(12) | 8568.3(10) | 4797.7(11) | 18.2(3) |
| C14 | 5458.6(12) | 8440.5(10) | 5801.1(10) | 17.6(3) |
| C15 | 2552.2(13) | 8352.9(12) | 3291.4(12) | 27.9(3) |
| C16 | 6685.0(12) | 8701.7(10) | 4619.8(11) | 20.0(3) |
| C17 | 4869.9(11) | 6946.0(10) | 7512.3(11) | 16.9(3) |
| C18 | 5451.8(12) | 6702.6(10) | 8512.4(11) | 18.2(3) |
| C19 | 5759.7(12) | 5726.5(10) | 8772.5(11) | 19.4(3) |
| C20 | 5495.7(12) | 4945.2(10) | 8041.4(11) | 20.9(3) |
| C21 | 4929.5(12) | 5168.2(10) | 7038.5(11) | 19.7(3) |
| C22 | 4628.5(12) | 6145.0(10) | 6778.5(11) | 18.3(3) |
| C23 | 6407.2(13) | 5538.5(11) | 9856.5(12) | 23.2(3) |
| C24 | 4651.6(13) | 4321.4(11) | 6251.5(12) | 23.7(3) |
| C25 | 3116.0(11) | 8132.4(10) | 7399.2(10) | 16.5(3) |
| C26 | 2379.7(12) | 8901.4(10) | 6984.7(10) | 17.4(3) |
| C27 | 1362.9(12) | 9039.3(10) | 7232.4(11) | 19.7(3) |
| C28 | 1021.9(12) | 8415.6(11) | 7905.6(11) | 21.2(3) |
| C29 | 1715.8(12) | 7638.3(10) | 8305.1(11) | 19.9(3) |
| C30 | 2734.6(12) | 7497.1(10) | 8049.8(11) | 18.2(3) |
| C31 | 594.8(13) | 9859.3(12) | 6775.1(12) | 25.3(3) |
| C32 | 1395.6(13) | 6966.2(11) | 9052.6(12) | 24.5(3) |
| B1 | 4389.3(13) | 8071.5(11) | 7229.7(12) | 16.3(3) |
| N1B | 8403.9(11) | 4628.0(10) | 4156.0(10) | 26.6(3) |
| N2B | 8191.4(12) | 6249.1(10) | 3021.1(10) | 26.8(3) |
| C1B | 8501.2(14) | 3816.0(12) | 4707.9(12) | 30.1(4) |
| C2B | 9355.5(14) | 3124.0(12) | 4750.3(12) | 30.9(4) |
| C3B | 10142.3(14) | 3280.7(12) | 4213.0(12) | 28.0(3) |
| C4B | 10087.3(13) | 4141.0(11) | 3621.0(11) | 23.8(3) |
| C5B | 10879.2(13) | 4379.9(12) | 3026.3(12) | 26.7(3) |
| C6B | 11830.6(16) | 3664.0(14) | 3065.5(16) | 40.2(4) |
| C7B | 10752.1(13) | 5219.4(12) | 2452.9(12) | 27.1(3) |
| C8B | 11521.6(17) | 5483.9(15) | 1790.6(16) | 41.0(4) |
| C9B | 9827.6(13) | 5882.4(11) | 2450.0(11) | 23.7(3) |
| C10B | 9621.0(15) | 6767.9(12) | 1888.8(12) | 29.6(3) |
| C11B | 8719.2(15) | 7356.2(12) | 1915.6(13) | 32.8(4) |
| C12B | 7997.8(15) | 7082.0(12) | 2493.1(13) | 32.1(4) |
| C13B | 9071.4(13) | 5646.5(11) | 3023.7(11) | 22.6(3) |
| C14B | 9184.4(13) | 4772.0(11) | 3625.9(11) | 22.8(3) |
| F1A | 8487(16) | 8462(9) | 9360(30) | 44(6) |
| F2A | 8688(15) | 9810(20) | 10261(12) | 35(5) |
| F3A | 8908(13) | 9800(20) | 8739(18) | 43(5) |
| F3B | 8669(6) | 8810(5) | 8729(5) | 63(2) |
| F2B | 8865(7) | 10321(5) | 9301(6) | 36.2(13) |
| F1B | 8561(6) | 9199(5) | 10271(4) | 55.9(17) |
| F8B | 2554(15) | 8740(20) | 2453(10) | 61(2) |
| F9B | 1645(10) | 8655(16) | 3620(12) | 27.3(11) |
| F7B | 2248(15) | 7334(9) | 3048(18) | 49.7(19) |
| F8A | 2753(8) | 7920(7) | 2426(5) | 27.3(11) |
| F7A | 1711(6) | 7807(8) | 3478(7) | 61(2) |
| F9A | 2152(8) | 9240(5) | 3019(7) | 49.7(19) |
| F19A | 1097(7) | 10593(5) | 6399(7) | 37.4(14) |
| F21A | -239(9) | 9518(9) | 6005(9) | 63(3) |
| F20A | 124(8) | 10290(7) | 7461(6) | 61(2) |

Table 3 Anisotropic Displacement Parameters (Å2×103) for c080620\_3\_2. The Anisotropic displacement factor exponent takes the form: -2π2[h2a\*2U11+2hka\*b\*U12+…].

| Atom | U11 | U22 | U33 | U23 | U13 | U12 |
| --- | --- | --- | --- | --- | --- | --- |
| F1 | 28.5(19) | 93(5) | 57(4) | 48(3) | 4(3) | 20(3) |
| F2 | 20(2) | 40(3) | 102(7) | -26(3) | -3(3) | 0(2) |
| F3 | 22.8(17) | 62(4) | 49.3(18) | 3(2) | 16.3(13) | 14(2) |
| F4 | 27.4(5) | 23.2(5) | 47.8(6) | -5.0(4) | 17.5(4) | 4.4(4) |
| F5 | 32.7(5) | 29.7(5) | 29.5(5) | -15.1(4) | 0.4(4) | 6.1(4) |
| F6 | 55.6(6) | 20.6(4) | 27.5(5) | 6.3(4) | 16.5(4) | 14.1(4) |
| F7 | 20(2) | 81(4) | 38(4) | 26(3) | -1.8(19) | -12.3(18) |
| F8 | 50(4) | 59(5) | 31(3) | -19(3) | -5(3) | 2(3) |
| F9 | 32(3) | 40(2) | 24(2) | 19.3(17) | -0.5(18) | 6.4(16) |
| F10 | 25.0(5) | 28.1(5) | 48.5(6) | 12.4(4) | 15.0(4) | 11.2(4) |
| F11 | 27.2(5) | 49.8(6) | 22.3(4) | 6.9(4) | 10.9(4) | 0.4(4) |
| F12 | 28.9(5) | 31.7(5) | 42.0(6) | -12.7(4) | 14.1(4) | -9.2(4) |
| F13 | 45.7(6) | 18.0(4) | 37.0(5) | -2.5(4) | 16.2(4) | -7.5(4) |
| F14 | 39.2(5) | 22.7(5) | 38.8(5) | -6.3(4) | 16.1(4) | 8.6(4) |
| F15 | 41.0(5) | 22.4(4) | 24.0(4) | -3.9(3) | 3.2(4) | 0.8(4) |
| F16 | 41.0(6) | 23.2(5) | 33.2(5) | 13.1(4) | -0.5(4) | 2.5(4) |
| F17 | 24.4(5) | 37.5(5) | 30.0(5) | 4.1(4) | 0.9(4) | 1.5(4) |
| F18 | 43.8(6) | 38.8(5) | 18.9(4) | 4.2(4) | 7.7(4) | 15.7(4) |
| F19 | 46(4) | 64(4) | 84(4) | 59(3) | 42(3) | 33(3) |
| F20 | 48(2) | 25.0(17) | 36(2) | -7.7(17) | 0.6(18) | 21.1(14) |
| F21 | 26(2) | 35(3) | 69(4) | -7(3) | -14(2) | 9.5(17) |
| F22 | 56.6(7) | 34.3(5) | 21.7(5) | 1.6(4) | 11.8(4) | -12.0(5) |
| F23 | 34.4(6) | 40.7(6) | 66.4(7) | 23.7(5) | 32.4(5) | 10.9(4) |
| F24 | 31.1(5) | 18.9(4) | 31.4(5) | 5.0(4) | 6.3(4) | -1.2(3) |
| C1 | 20.9(7) | 15.3(6) | 13.6(6) | 3.4(5) | 5.7(5) | 2.7(5) |
| C2 | 21.3(7) | 16.3(7) | 18.6(7) | 2.4(5) | 7.2(6) | 4.3(5) |
| C3 | 18.5(7) | 22.6(7) | 18.3(7) | 3.3(5) | 5.4(5) | 2.3(5) |
| C4 | 21.1(7) | 19.3(7) | 18.2(7) | 0.0(5) | 4.2(6) | -0.6(5) |
| C5 | 22.0(7) | 16.8(7) | 14.9(6) | 2.1(5) | 6.2(5) | 2.7(5) |
| C6 | 17.9(7) | 18.3(7) | 15.1(6) | 2.6(5) | 4.2(5) | 3.2(5) |
| C7 | 21.4(8) | 27.0(8) | 34.0(8) | 2.0(6) | 6.1(6) | 1.8(6) |
| C8 | 22.2(7) | 19.7(7) | 17.6(7) | -1.1(5) | 4.8(6) | 1.4(5) |
| C9 | 22.4(7) | 9.8(6) | 18.6(7) | 1.0(5) | 5.8(5) | 2.9(5) |
| C10 | 19.2(7) | 13.9(6) | 22.0(7) | 0.6(5) | 6.5(6) | 1.5(5) |
| C11 | 21.6(7) | 16.2(7) | 19.6(7) | 0.2(5) | 3.5(6) | 2.2(5) |
| C12 | 25.9(8) | 15.7(7) | 16.5(7) | 1.0(5) | 7.4(6) | 3.1(5) |
| C13 | 21.5(7) | 12.9(6) | 21.7(7) | 1.0(5) | 7.8(6) | 3.1(5) |
| C14 | 20.4(7) | 14.8(6) | 16.9(7) | 0.3(5) | 3.3(5) | 3.1(5) |
| C15 | 25.6(8) | 37.1(9) | 20.5(7) | 0.8(6) | 4.9(6) | -0.3(7) |
| C16 | 23.8(7) | 17.3(7) | 19.3(7) | 1.5(5) | 6.1(6) | 3.1(5) |
| C17 | 16.6(7) | 16.4(7) | 19.5(7) | 1.8(5) | 8.0(5) | 2.7(5) |
| C18 | 20.2(7) | 16.2(7) | 19.4(7) | 0.5(5) | 7.3(5) | 2.9(5) |
| C19 | 19.2(7) | 19.6(7) | 20.7(7) | 4.2(5) | 6.8(6) | 5.3(5) |
| C20 | 22.8(7) | 15.1(7) | 26.9(7) | 4.2(6) | 9.4(6) | 5.5(5) |
| C21 | 21.4(7) | 16.3(7) | 23.2(7) | 0.0(5) | 8.9(6) | 2.2(5) |
| C22 | 19.9(7) | 16.9(7) | 18.6(7) | 1.3(5) | 5.9(5) | 2.3(5) |
| C23 | 25.6(8) | 20.1(7) | 24.0(7) | 4.0(6) | 6.1(6) | 4.9(6) |
| C24 | 29.4(8) | 16.9(7) | 26.3(8) | 0.7(6) | 10.1(6) | 2.3(6) |
| C25 | 18.9(7) | 14.1(6) | 15.4(6) | -2.6(5) | 2.6(5) | 0.0(5) |
| C26 | 19.5(7) | 15.2(6) | 17.0(6) | 0.5(5) | 4.0(5) | 0.6(5) |
| C27 | 19.1(7) | 18.2(7) | 20.6(7) | -0.4(5) | 3.0(6) | 3.1(5) |
| C28 | 18.4(7) | 21.6(7) | 24.5(7) | -0.9(6) | 7.4(6) | 1.9(5) |
| C29 | 20.9(7) | 18.0(7) | 21.4(7) | 0.1(5) | 6.6(6) | -0.4(5) |
| C30 | 20.0(7) | 14.9(6) | 19.3(7) | 0.0(5) | 4.1(5) | 2.4(5) |
| C31 | 20.8(7) | 26.7(8) | 29.6(8) | 5.4(6) | 7.9(6) | 5.9(6) |
| C32 | 24.7(8) | 21.7(7) | 29.0(8) | 2.4(6) | 10.6(6) | 1.2(6) |
| B1 | 18.7(8) | 14.4(7) | 16.2(7) | 1.5(6) | 4.9(6) | 3.3(6) |
| N1B | 27.1(7) | 28.0(7) | 24.6(6) | -1.9(5) | 7.3(5) | -4.4(5) |
| N2B | 28.0(7) | 25.1(7) | 26.8(7) | -0.3(5) | 6.5(6) | 2.8(5) |
| C1B | 33.5(9) | 30.9(8) | 26.2(8) | 0.7(6) | 8.7(7) | -9.9(7) |
| C2B | 35.6(9) | 26.0(8) | 27.6(8) | 4.1(6) | 1.7(7) | -7.2(7) |
| C3B | 28.4(8) | 23.3(8) | 28.3(8) | 1.9(6) | -0.1(6) | -0.7(6) |
| C4B | 23.7(8) | 22.8(7) | 22.2(7) | -0.5(6) | 1.1(6) | -1.8(6) |
| C5B | 23.7(8) | 27.8(8) | 27.0(8) | -0.6(6) | 4.0(6) | 0.7(6) |
| C6B | 32.9(10) | 41.1(10) | 49.3(11) | 9.1(8) | 14.1(8) | 11.7(8) |
| C7B | 25.6(8) | 28.9(8) | 27.2(8) | -1.3(6) | 7.6(6) | -2.4(6) |
| C8B | 40.2(11) | 40.4(10) | 50.7(11) | 8.5(8) | 25.9(9) | 3.4(8) |
| C9B | 25.8(8) | 21.2(7) | 22.2(7) | -1.2(6) | 3.3(6) | -3.3(6) |
| C10B | 36.5(9) | 25.4(8) | 25.6(8) | 1.6(6) | 5.9(7) | -5.6(7) |
| C11B | 44.3(10) | 21.3(8) | 29.4(8) | 3.0(6) | 3.1(7) | 1.9(7) |
| C12B | 36.6(9) | 25.1(8) | 31.9(9) | -1.0(7) | 3.7(7) | 6.9(7) |
| C13B | 24.0(8) | 21.2(7) | 20.6(7) | -3.7(6) | 2.7(6) | -0.8(6) |
| C14B | 24.1(8) | 22.0(7) | 21.1(7) | -2.1(6) | 3.9(6) | -3.6(6) |
| F1A | 29(12) | 32(6) | 70(18) | 3(8) | 13(14) | 8(7) |
| F2A | 29(11) | 43(13) | 27(7) | 1(8) | -3(6) | 16(11) |
| F3A | 36(9) | 46(12) | 53(11) | 0(10) | 22(9) | -4(10) |
| F3B | 20.8(16) | 60(4) | 101(5) | -45(3) | 12(3) | 6(2) |
| F2B | 19.2(16) | 36(2) | 51(2) | 15.8(18) | 2.0(15) | -8.5(16) |
| F1B | 24.2(15) | 90(5) | 52(3) | 48(3) | 1.6(18) | 8(3) |
| F8B | 38(3) | 125(6) | 15(2) | 7(3) | -2(2) | -41(3) |
| F9B | 29(2) | 37(2) | 14.8(19) | -4.3(14) | 4.5(15) | -4.7(15) |
| F7B | 38(3) | 54(3) | 44(3) | -11(2) | -12(2) | 25(2) |
| F8A | 29(2) | 37(2) | 14.8(19) | -4.3(14) | 4.5(15) | -4.7(15) |
| F7A | 38(3) | 125(6) | 15(2) | 7(3) | -2(2) | -41(3) |
| F9A | 38(3) | 54(3) | 44(3) | -11(2) | -12(2) | 25(2) |
| F19A | 28(3) | 15.4(19) | 69(3) | 9.7(18) | 12.7(19) | 6.3(15) |
| F21A | 53(5) | 53(4) | 59(3) | 17(3) | -32(3) | 4(3) |
| F20A | 70(4) | 66(4) | 71(4) | 37(3) | 55(3) | 48(3) |

Table 4 Bond Lengths for c080620\_3\_2.

| Atom | Atom | Length/Å |  | Atom | Atom | Length/Å |
| --- | --- | --- | --- | --- | --- | --- |
| F1 | C7 | 1.312(6) |  | C13 | C16 | 1.494(2) |
| F2 | C7 | 1.309(6) |  | C15 | F8B | 1.256(10) |
| F3 | C7 | 1.364(6) |  | C15 | F9B | 1.363(10) |
| F4 | C8 | 1.3451(17) |  | C15 | F7B | 1.415(11) |
| F5 | C8 | 1.3304(17) |  | C15 | F8A | 1.357(6) |
| F6 | C8 | 1.3484(16) |  | C15 | F7A | 1.345(6) |
| F7 | C15 | 1.328(7) |  | C15 | F9A | 1.316(6) |
| F8 | C15 | 1.321(7) |  | C17 | C18 | 1.4003(19) |
| F9 | C15 | 1.364(6) |  | C17 | C22 | 1.4048(19) |
| F10 | C16 | 1.3506(16) |  | C17 | B1 | 1.6441(19) |
| F11 | C16 | 1.3428(17) |  | C18 | C19 | 1.3943(19) |
| F12 | C16 | 1.3291(17) |  | C19 | C20 | 1.384(2) |
| F13 | C24 | 1.3608(17) |  | C19 | C23 | 1.499(2) |
| F14 | C24 | 1.3408(18) |  | C20 | C21 | 1.391(2) |
| F15 | C24 | 1.3361(18) |  | C21 | C22 | 1.3935(19) |
| F16 | C23 | 1.3385(17) |  | C21 | C24 | 1.495(2) |
| F17 | C23 | 1.3462(18) |  | C25 | C26 | 1.4108(19) |
| F18 | C23 | 1.3423(17) |  | C25 | C30 | 1.4005(19) |
| F19 | C31 | 1.314(6) |  | C25 | B1 | 1.646(2) |
| F20 | C31 | 1.341(5) |  | C26 | C27 | 1.389(2) |
| F21 | C31 | 1.322(7) |  | C27 | C28 | 1.389(2) |
| F22 | C32 | 1.3430(19) |  | C27 | C31 | 1.498(2) |
| F23 | C32 | 1.3457(18) |  | C28 | C29 | 1.386(2) |
| F24 | C32 | 1.3430(17) |  | C29 | C30 | 1.397(2) |
| C1 | C2 | 1.403(2) |  | C29 | C32 | 1.493(2) |
| C1 | C6 | 1.4031(19) |  | C31 | F19A | 1.337(6) |
| C1 | B1 | 1.650(2) |  | C31 | F21A | 1.321(7) |
| C2 | C3 | 1.391(2) |  | C31 | F20A | 1.325(6) |
| C3 | C4 | 1.387(2) |  | N1B | C1B | 1.325(2) |
| C3 | C7 | 1.491(2) |  | N1B | C14B | 1.351(2) |
| C4 | C5 | 1.385(2) |  | N2B | C12B | 1.332(2) |
| C5 | C6 | 1.393(2) |  | N2B | C13B | 1.357(2) |
| C5 | C8 | 1.4964(19) |  | C1B | C2B | 1.400(3) |
| C7 | F1A | 1.385(10) |  | C2B | C3B | 1.368(2) |
| C7 | F2A | 1.351(10) |  | C3B | C4B | 1.414(2) |
| C7 | F3A | 1.276(10) |  | C4B | C5B | 1.451(2) |
| C7 | F3B | 1.305(5) |  | C4B | C14B | 1.404(2) |
| C7 | F2B | 1.341(5) |  | C5B | C6B | 1.513(2) |
| C7 | F1B | 1.359(5) |  | C5B | C7B | 1.371(2) |
| C9 | C10 | 1.401(2) |  | C7B | C8B | 1.505(2) |
| C9 | C14 | 1.399(2) |  | C7B | C9B | 1.450(2) |
| C9 | B1 | 1.652(2) |  | C9B | C10B | 1.416(2) |
| C10 | C11 | 1.391(2) |  | C9B | C13B | 1.393(2) |
| C11 | C12 | 1.385(2) |  | C10B | C11B | 1.375(3) |
| C11 | C15 | 1.493(2) |  | C11B | C12B | 1.376(3) |
| C12 | C13 | 1.386(2) |  | C13B | C14B | 1.433(2) |
| C13 | C14 | 1.393(2) |  |  |  |  |

Table 5 Bond Angles for c080620\_3\_2.

| Atom | Atom | Atom | Angle/˚ |  | Atom | Atom | Atom | Angle/˚ |
| --- | --- | --- | --- | --- | --- | --- | --- | --- |
| C2 | C1 | C6 | 115.11(13) |  | C19 | C20 | C21 | 117.88(13) |
| C2 | C1 | B1 | 122.40(12) |  | C20 | C21 | C22 | 121.15(13) |
| C6 | C1 | B1 | 122.39(12) |  | C20 | C21 | C24 | 117.60(13) |
| C3 | C2 | C1 | 122.67(13) |  | C22 | C21 | C24 | 121.24(13) |
| C2 | C3 | C7 | 119.94(13) |  | C21 | C22 | C17 | 121.90(13) |
| C4 | C3 | C2 | 120.99(13) |  | F16 | C23 | F17 | 106.67(12) |
| C4 | C3 | C7 | 119.05(13) |  | F16 | C23 | F18 | 106.42(12) |
| C5 | C4 | C3 | 117.62(13) |  | F16 | C23 | C19 | 112.77(12) |
| C4 | C5 | C6 | 121.23(13) |  | F17 | C23 | C19 | 111.95(12) |
| C4 | C5 | C8 | 118.93(13) |  | F18 | C23 | F17 | 105.50(12) |
| C6 | C5 | C8 | 119.76(13) |  | F18 | C23 | C19 | 113.00(12) |
| C5 | C6 | C1 | 122.37(13) |  | F13 | C24 | C21 | 111.30(12) |
| F1 | C7 | F3 | 103.3(4) |  | F14 | C24 | F13 | 105.65(11) |
| F1 | C7 | C3 | 113.8(4) |  | F14 | C24 | C21 | 112.68(13) |
| F2 | C7 | F1 | 109.9(5) |  | F15 | C24 | F13 | 105.83(12) |
| F2 | C7 | F3 | 104.9(5) |  | F15 | C24 | F14 | 106.82(12) |
| F2 | C7 | C3 | 113.9(5) |  | F15 | C24 | C21 | 113.97(12) |
| F3 | C7 | C3 | 110.2(4) |  | C26 | C25 | B1 | 121.72(12) |
| F1A | C7 | C3 | 109.5(8) |  | C30 | C25 | C26 | 115.40(13) |
| F2A | C7 | C3 | 112.2(8) |  | C30 | C25 | B1 | 122.54(12) |
| F2A | C7 | F1A | 100.5(10) |  | C27 | C26 | C25 | 122.28(13) |
| F3A | C7 | C3 | 119.2(8) |  | C26 | C27 | C31 | 121.09(13) |
| F3A | C7 | F1A | 105.7(10) |  | C28 | C27 | C26 | 121.03(13) |
| F3A | C7 | F2A | 108.0(10) |  | C28 | C27 | C31 | 117.88(13) |
| F3B | C7 | C3 | 114.6(3) |  | C29 | C28 | C27 | 117.96(13) |
| F3B | C7 | F2B | 107.0(5) |  | C28 | C29 | C30 | 120.91(13) |
| F3B | C7 | F1B | 107.6(4) |  | C28 | C29 | C32 | 119.71(13) |
| F2B | C7 | C3 | 112.9(4) |  | C30 | C29 | C32 | 119.34(13) |
| F2B | C7 | F1B | 103.0(4) |  | C29 | C30 | C25 | 122.37(13) |
| F1B | C7 | C3 | 111.0(3) |  | F19 | C31 | F20 | 105.8(5) |
| F4 | C8 | F6 | 105.20(12) |  | F19 | C31 | F21 | 106.6(7) |
| F4 | C8 | C5 | 112.95(12) |  | F19 | C31 | C27 | 113.3(4) |
| F5 | C8 | F4 | 106.92(11) |  | F20 | C31 | C27 | 112.2(3) |
| F5 | C8 | F6 | 106.02(12) |  | F21 | C31 | F20 | 105.7(5) |
| F5 | C8 | C5 | 112.97(12) |  | F21 | C31 | C27 | 112.7(5) |
| F6 | C8 | C5 | 112.19(11) |  | F19A | C31 | C27 | 114.1(4) |
| C10 | C9 | B1 | 124.53(12) |  | F21A | C31 | C27 | 111.7(5) |
| C14 | C9 | C10 | 115.34(12) |  | F21A | C31 | F19A | 106.3(7) |
| C14 | C9 | B1 | 120.04(12) |  | F21A | C31 | F20A | 106.1(6) |
| C11 | C10 | C9 | 122.39(13) |  | F20A | C31 | C27 | 111.8(4) |
| C10 | C11 | C15 | 120.97(13) |  | F20A | C31 | F19A | 106.4(5) |
| C12 | C11 | C10 | 121.11(13) |  | F22 | C32 | F23 | 106.42(13) |
| C12 | C11 | C15 | 117.91(13) |  | F22 | C32 | C29 | 112.55(12) |
| C11 | C12 | C13 | 117.72(13) |  | F23 | C32 | C29 | 113.13(13) |
| C12 | C13 | C14 | 120.92(13) |  | F24 | C32 | F22 | 105.82(12) |
| C12 | C13 | C16 | 120.28(13) |  | F24 | C32 | F23 | 105.69(12) |
| C14 | C13 | C16 | 118.79(13) |  | F24 | C32 | C29 | 112.64(12) |
| C13 | C14 | C9 | 122.49(13) |  | C1 | B1 | C9 | 107.42(11) |
| F7 | C15 | F9 | 105.3(5) |  | C17 | B1 | C1 | 111.36(11) |
| F7 | C15 | C11 | 113.8(4) |  | C17 | B1 | C9 | 107.87(11) |
| F8 | C15 | F7 | 107.1(6) |  | C17 | B1 | C25 | 108.51(11) |
| F8 | C15 | F9 | 104.9(6) |  | C25 | B1 | C1 | 108.66(11) |
| F8 | C15 | C11 | 114.4(6) |  | C25 | B1 | C9 | 113.06(11) |
| F9 | C15 | C11 | 110.6(4) |  | C1B | N1B | C14B | 116.69(14) |
| F8B | C15 | C11 | 121.6(8) |  | C12B | N2B | C13B | 123.42(15) |
| F8B | C15 | F9B | 110.4(10) |  | N1B | C1B | C2B | 122.98(15) |
| F8B | C15 | F7B | 106.4(10) |  | C3B | C2B | C1B | 119.72(15) |
| F9B | C15 | C11 | 110.1(7) |  | C2B | C3B | C4B | 119.72(15) |
| F9B | C15 | F7B | 99.2(9) |  | C3B | C4B | C5B | 124.17(15) |
| F7B | C15 | C11 | 106.7(7) |  | C14B | C4B | C3B | 115.37(14) |
| F8A | C15 | C11 | 111.8(4) |  | C14B | C4B | C5B | 120.45(14) |
| F7A | C15 | C11 | 112.1(4) |  | C4B | C5B | C6B | 117.16(14) |
| F7A | C15 | F8A | 105.2(5) |  | C7B | C5B | C4B | 120.55(14) |
| F9A | C15 | C11 | 114.6(4) |  | C7B | C5B | C6B | 122.28(15) |
| F9A | C15 | F8A | 105.3(5) |  | C5B | C7B | C8B | 122.86(15) |
| F9A | C15 | F7A | 107.2(5) |  | C5B | C7B | C9B | 119.87(14) |
| F10 | C16 | C13 | 112.10(11) |  | C9B | C7B | C8B | 117.25(14) |
| F11 | C16 | F10 | 105.22(11) |  | C10B | C9B | C7B | 124.46(15) |
| F11 | C16 | C13 | 112.78(12) |  | C13B | C9B | C7B | 119.15(14) |
| F12 | C16 | F10 | 105.87(12) |  | C13B | C9B | C10B | 116.39(14) |
| F12 | C16 | F11 | 106.79(12) |  | C11B | C10B | C9B | 121.22(16) |
| F12 | C16 | C13 | 113.45(12) |  | C10B | C11B | C12B | 119.74(15) |
| C18 | C17 | C22 | 115.75(12) |  | N2B | C12B | C11B | 119.08(16) |
| C18 | C17 | B1 | 122.54(12) |  | N2B | C13B | C9B | 120.15(14) |
| C22 | C17 | B1 | 121.46(12) |  | N2B | C13B | C14B | 117.83(14) |
| C19 | C18 | C17 | 122.42(13) |  | C9B | C13B | C14B | 122.01(14) |
| C18 | C19 | C23 | 118.78(13) |  | N1B | C14B | C4B | 125.50(14) |
| C20 | C19 | C18 | 120.89(13) |  | N1B | C14B | C13B | 116.53(14) |
| C20 | C19 | C23 | 120.31(13) |  | C4B | C14B | C13B | 117.96(14) |

Table 6 Torsion Angles for c080620\_3\_2.

| A | B | C | D | Angle/˚ |  | A | B | C | D | Angle/˚ |
| --- | --- | --- | --- | --- | --- | --- | --- | --- | --- | --- |
| C1 | C2 | C3 | C4 | -0.3(2) |  | C20 | C19 | C23 | F18 | -143.97(14) |
| C1 | C2 | C3 | C7 | 178.29(13) |  | C20 | C21 | C22 | C17 | -0.6(2) |
| C2 | C1 | C6 | C5 | 1.13(19) |  | C20 | C21 | C24 | F13 | 55.97(18) |
| C2 | C1 | B1 | C9 | -82.30(15) |  | C20 | C21 | C24 | F14 | -62.51(17) |
| C2 | C1 | B1 | C17 | 35.61(17) |  | C20 | C21 | C24 | F15 | 175.55(13) |
| C2 | C1 | B1 | C25 | 155.07(12) |  | C22 | C17 | C18 | C19 | -0.9(2) |
| C2 | C3 | C4 | C5 | 0.1(2) |  | C22 | C17 | B1 | C1 | -155.35(12) |
| C2 | C3 | C7 | F1 | -70.1(4) |  | C22 | C17 | B1 | C9 | -37.71(17) |
| C2 | C3 | C7 | F2 | 162.9(5) |  | C22 | C17 | B1 | C25 | 85.10(15) |
| C2 | C3 | C7 | F3 | 45.3(4) |  | C22 | C21 | C24 | F13 | -123.77(14) |
| C2 | C3 | C7 | F1A | -26.6(16) |  | C22 | C21 | C24 | F14 | 117.75(15) |
| C2 | C3 | C7 | F2A | -137.3(16) |  | C22 | C21 | C24 | F15 | -4.2(2) |
| C2 | C3 | C7 | F3A | 95.1(17) |  | C23 | C19 | C20 | C21 | -177.69(13) |
| C2 | C3 | C7 | F3B | 23.2(4) |  | C24 | C21 | C22 | C17 | 179.10(13) |
| C2 | C3 | C7 | F2B | 145.9(4) |  | C25 | C26 | C27 | C28 | -0.2(2) |
| C2 | C3 | C7 | F1B | -99.0(4) |  | C25 | C26 | C27 | C31 | -179.65(13) |
| C3 | C4 | C5 | C6 | 0.7(2) |  | C26 | C25 | C30 | C29 | -2.5(2) |
| C3 | C4 | C5 | C8 | -176.00(12) |  | C26 | C25 | B1 | C1 | 74.70(15) |
| C4 | C3 | C7 | F1 | 108.5(4) |  | C26 | C25 | B1 | C9 | -44.45(17) |
| C4 | C3 | C7 | F2 | -18.5(5) |  | C26 | C25 | B1 | C17 | -164.07(12) |
| C4 | C3 | C7 | F3 | -136.1(4) |  | C26 | C27 | C28 | C29 | -1.4(2) |
| C4 | C3 | C7 | F1A | 152.1(16) |  | C26 | C27 | C31 | F19 | -9.9(5) |
| C4 | C3 | C7 | F2A | 41.3(16) |  | C26 | C27 | C31 | F20 | -129.6(4) |
| C4 | C3 | C7 | F3A | -86.2(17) |  | C26 | C27 | C31 | F21 | 111.3(6) |
| C4 | C3 | C7 | F3B | -158.2(4) |  | C26 | C27 | C31 | F19A | -19.5(4) |
| C4 | C3 | C7 | F2B | -35.4(4) |  | C26 | C27 | C31 | F21A | 101.0(7) |
| C4 | C3 | C7 | F1B | 79.6(4) |  | C26 | C27 | C31 | F20A | -140.3(5) |
| C4 | C5 | C6 | C1 | -1.4(2) |  | C27 | C28 | C29 | C30 | 1.0(2) |
| C4 | C5 | C8 | F4 | -147.44(13) |  | C27 | C28 | C29 | C32 | 178.43(13) |
| C4 | C5 | C8 | F5 | -25.91(18) |  | C28 | C27 | C31 | F19 | 170.6(5) |
| C4 | C5 | C8 | F6 | 93.86(15) |  | C28 | C27 | C31 | F20 | 51.0(4) |
| C6 | C1 | C2 | C3 | -0.31(19) |  | C28 | C27 | C31 | F21 | -68.2(6) |
| C6 | C1 | B1 | C9 | 93.73(15) |  | C28 | C27 | C31 | F19A | 161.0(4) |
| C6 | C1 | B1 | C17 | -148.36(12) |  | C28 | C27 | C31 | F21A | -78.5(7) |
| C6 | C1 | B1 | C25 | -28.90(17) |  | C28 | C27 | C31 | F20A | 40.3(5) |
| C6 | C5 | C8 | F4 | 35.85(18) |  | C28 | C29 | C30 | C25 | 1.0(2) |
| C6 | C5 | C8 | F5 | 157.37(12) |  | C28 | C29 | C32 | F22 | -108.20(16) |
| C6 | C5 | C8 | F6 | -82.85(16) |  | C28 | C29 | C32 | F23 | 12.5(2) |
| C7 | C3 | C4 | C5 | -178.47(13) |  | C28 | C29 | C32 | F24 | 132.27(14) |
| C8 | C5 | C6 | C1 | 175.28(12) |  | C30 | C25 | C26 | C27 | 2.1(2) |
| C9 | C10 | C11 | C12 | 0.7(2) |  | C30 | C25 | B1 | C1 | -98.25(14) |
| C9 | C10 | C11 | C15 | -178.39(13) |  | C30 | C25 | B1 | C9 | 142.60(13) |
| C10 | C9 | C14 | C13 | 0.41(19) |  | C30 | C25 | B1 | C17 | 22.98(17) |
| C10 | C9 | B1 | C1 | -136.13(13) |  | C30 | C29 | C32 | F22 | 69.31(17) |
| C10 | C9 | B1 | C17 | 103.72(14) |  | C30 | C29 | C32 | F23 | -170.02(13) |
| C10 | C9 | B1 | C25 | -16.26(18) |  | C30 | C29 | C32 | F24 | -50.22(18) |
| C10 | C11 | C12 | C13 | 1.0(2) |  | C31 | C27 | C28 | C29 | 178.10(13) |
| C10 | C11 | C15 | F7 | -11.2(6) |  | C32 | C29 | C30 | C25 | -176.43(13) |
| C10 | C11 | C15 | F8 | -134.8(6) |  | B1 | C1 | C2 | C3 | 175.98(13) |
| C10 | C11 | C15 | F9 | 107.1(5) |  | B1 | C1 | C6 | C5 | -175.16(12) |
| C10 | C11 | C15 | F8B | 159.9(16) |  | B1 | C9 | C10 | C11 | -177.88(12) |
| C10 | C11 | C15 | F9B | 28.6(10) |  | B1 | C9 | C14 | C13 | 177.06(12) |
| C10 | C11 | C15 | F7B | -78.1(12) |  | B1 | C17 | C18 | C19 | 173.38(13) |
| C10 | C11 | C15 | F8A | -143.3(5) |  | B1 | C17 | C22 | C21 | -173.05(13) |
| C10 | C11 | C15 | F7A | -25.5(6) |  | B1 | C25 | C26 | C27 | -171.36(12) |
| C10 | C11 | C15 | F9A | 97.0(5) |  | B1 | C25 | C30 | C29 | 170.88(13) |
| C11 | C12 | C13 | C14 | -2.0(2) |  | N1B | C1B | C2B | C3B | 0.7(2) |
| C11 | C12 | C13 | C16 | 176.69(12) |  | N2B | C13B | C14B | N1B | -0.8(2) |
| C12 | C11 | C15 | F7 | 169.7(6) |  | N2B | C13B | C14B | C4B | 179.31(13) |
| C12 | C11 | C15 | F8 | 46.1(6) |  | C1B | N1B | C14B | C4B | -0.6(2) |
| C12 | C11 | C15 | F9 | -72.1(5) |  | C1B | N1B | C14B | C13B | 179.52(13) |
| C12 | C11 | C15 | F8B | -19.2(16) |  | C1B | C2B | C3B | C4B | 0.1(2) |
| C12 | C11 | C15 | F9B | -150.5(10) |  | C2B | C3B | C4B | C5B | 179.80(15) |
| C12 | C11 | C15 | F7B | 102.7(12) |  | C2B | C3B | C4B | C14B | -1.0(2) |
| C12 | C11 | C15 | F8A | 37.5(5) |  | C3B | C4B | C5B | C6B | -1.1(2) |
| C12 | C11 | C15 | F7A | 155.3(6) |  | C3B | C4B | C5B | C7B | 178.52(15) |
| C12 | C11 | C15 | F9A | -82.2(5) |  | C3B | C4B | C14B | N1B | 1.3(2) |
| C12 | C13 | C14 | C9 | 1.3(2) |  | C3B | C4B | C14B | C13B | -178.82(13) |
| C12 | C13 | C16 | F10 | -116.98(14) |  | C4B | C5B | C7B | C8B | -177.65(15) |
| C12 | C13 | C16 | F11 | 1.56(18) |  | C4B | C5B | C7B | C9B | 0.8(2) |
| C12 | C13 | C16 | F12 | 123.15(14) |  | C5B | C4B | C14B | N1B | -179.44(14) |
| C14 | C9 | C10 | C11 | -1.40(19) |  | C5B | C4B | C14B | C13B | 0.4(2) |
| C14 | C9 | B1 | C1 | 47.55(16) |  | C5B | C7B | C9B | C10B | 179.83(15) |
| C14 | C9 | B1 | C17 | -72.60(15) |  | C5B | C7B | C9B | C13B | -0.8(2) |
| C14 | C9 | B1 | C25 | 167.42(11) |  | C6B | C5B | C7B | C8B | 1.9(3) |
| C14 | C13 | C16 | F10 | 61.70(17) |  | C6B | C5B | C7B | C9B | -179.60(15) |
| C14 | C13 | C16 | F11 | -179.75(12) |  | C7B | C9B | C10B | C11B | 179.39(15) |
| C14 | C13 | C16 | F12 | -58.16(17) |  | C7B | C9B | C13B | N2B | -179.12(14) |
| C15 | C11 | C12 | C13 | -179.86(13) |  | C7B | C9B | C13B | C14B | 0.6(2) |
| C16 | C13 | C14 | C9 | -177.39(12) |  | C8B | C7B | C9B | C10B | -1.6(2) |
| C17 | C18 | C19 | C20 | -0.2(2) |  | C8B | C7B | C9B | C13B | 177.75(15) |
| C17 | C18 | C19 | C23 | 178.46(13) |  | C9B | C10B | C11B | C12B | -0.3(2) |
| C18 | C17 | C22 | C21 | 1.3(2) |  | C9B | C13B | C14B | N1B | 179.46(13) |
| C18 | C17 | B1 | C1 | 30.65(18) |  | C9B | C13B | C14B | C4B | -0.4(2) |
| C18 | C17 | B1 | C9 | 148.29(13) |  | C10B | C9B | C13B | N2B | 0.3(2) |
| C18 | C17 | B1 | C25 | -88.90(15) |  | C10B | C9B | C13B | C14B | -179.99(14) |
| C18 | C19 | C20 | C21 | 0.9(2) |  | C10B | C11B | C12B | N2B | 0.3(2) |
| C18 | C19 | C23 | F16 | 158.13(13) |  | C12B | N2B | C13B | C9B | -0.3(2) |
| C18 | C19 | C23 | F17 | -81.57(16) |  | C12B | N2B | C13B | C14B | 179.95(14) |
| C18 | C19 | C23 | F18 | 37.38(19) |  | C13B | N2B | C12B | C11B | 0.0(2) |
| C19 | C20 | C21 | C22 | -0.5(2) |  | C13B | C9B | C10B | C11B | 0.0(2) |
| C19 | C20 | C21 | C24 | 179.72(13) |  | C14B | N1B | C1B | C2B | -0.4(2) |
| C20 | C19 | C23 | F16 | -23.22(19) |  | C14B | C4B | C5B | C6B | 179.77(15) |
| C20 | C19 | C23 | F17 | 97.08(16) |  | C14B | C4B | C5B | C7B | -0.6(2) |

Table 7 Hydrogen Atom Coordinates (Å×104) and Isotropic Displacement Parameters (Å2×103) for c080620\_3\_2.

| Atom | *x* | *y* | *z* | U(eq) |
| --- | --- | --- | --- | --- |
| H2 | 6692.99 | 8266.28 | 8152.55 | 22 |
| H4 | 7049.6 | 11020.45 | 9571.75 | 24 |
| H6 | 3963.28 | 9929.2 | 8074.01 | 20 |
| H10 | 2773.63 | 8103.08 | 5289.32 | 22 |
| H12 | 4656.23 | 8659.38 | 3290.84 | 23 |
| H14 | 6122.16 | 8469.6 | 6350.36 | 21 |
| H18 | 5643.86 | 7221.51 | 9032.85 | 22 |
| H20 | 5694.74 | 4278.11 | 8218.84 | 25 |
| H22 | 4249.07 | 6272.96 | 6084.94 | 22 |
| H26 | 2586.22 | 9340.16 | 6520.03 | 21 |
| H28 | 334.25 | 8518.79 | 8086.43 | 25 |
| H30 | 3184.87 | 6950.49 | 8327.63 | 22 |
| H2B | 7732(15) | 6040(14) | 3399(14) | 32 |
| H1B | 7966.99 | 3698.09 | 5092.12 | 36 |
| H2BA | 9388.48 | 2547.99 | 5150.6 | 37 |
| H3B | 10723.24 | 2813.39 | 4237.44 | 34 |
| H6BA | 12185.34 | 3800.35 | 2507.5 | 60 |
| H6BB | 11536.69 | 2974.81 | 2981.78 | 60 |
| H6BC | 12385.94 | 3753 | 3732.53 | 60 |
| H6BD | 11605.29 | 3156.91 | 2501.99 | 60 |
| H6BE | 12010.71 | 3336.76 | 3728.97 | 60 |
| H6BF | 12491.98 | 4034.49 | 2990.85 | 60 |
| H8BA | 11491.16 | 6204.14 | 1678.89 | 61 |
| H8BB | 11284.05 | 5114.88 | 1123.73 | 61 |
| H8BC | 12290.98 | 5305.06 | 2137.89 | 61 |
| H8BD | 11075.75 | 5688.8 | 1116.32 | 61 |
| H8BE | 11964.48 | 4900.01 | 1695.73 | 61 |
| H8BF | 12025.95 | 6035.27 | 2128.46 | 61 |
| H10B | 10114.37 | 6959.08 | 1486.32 | 35 |
| H11B | 8594.83 | 7949.57 | 1536.95 | 39 |
| H12B | 7370.34 | 7481.16 | 2514.87 | 39 |

Table 8 Atomic Occupancy for c080620\_3\_2.

| Atom | *Occupancy* |  | Atom | *Occupancy* |  | Atom | *Occupancy* |
| --- | --- | --- | --- | --- | --- | --- | --- |
| F1 | 0.450(3) |  | F2 | 0.450(3) |  | F3 | 0.450(3) |
| F7 | 0.450(3) |  | F8 | 0.450(3) |  | F9 | 0.450(3) |
| F19 | 0.517(17) |  | F20 | 0.517(17) |  | F21 | 0.517(17) |
| H6BA | 0.517(17) |  | H6BB | 0.517(17) |  | H6BC | 0.517(17) |
| H6BD | 0.483(17) |  | H6BE | 0.483(17) |  | H6BF | 0.483(17) |
| H8BA | 0.517(17) |  | H8BB | 0.517(17) |  | H8BC | 0.517(17) |
| H8BD | 0.483(17) |  | H8BE | 0.483(17) |  | H8BF | 0.483(17) |
| F1A | 0.0484(15) |  | F2A | 0.0484(15) |  | F3A | 0.0484(15) |
| F3B | 0.502(3) |  | F2B | 0.502(3) |  | F1B | 0.502(3) |
| F8B | 0.0484(15) |  | F9B | 0.0484(15) |  | F7B | 0.0484(15) |
| F8A | 0.502(3) |  | F7A | 0.502(3) |  | F9A | 0.502(3) |
| F19A | 0.483(17) |  | F21A | 0.483(17) |  | F20A | 0.483(17) |

Experimental

Single crystals of C46H25BF24N2
[c080620\_3\_2]
were
[].
A suitable crystal was selected and
[]
on a
XtaLAB Synergy, Dualflex, Pilatus 200K
diffractometer. The crystal was kept at 100.0(1) K during data collection.
Using Olex2 [1], the structure was solved with the
SHELXT
[2] structure solution program using
Intrinsic Phasing
and refined with the
SHELXL
[3] refinement package using
Least Squares
minimisation.

1. Dolomanov, O.V., Bourhis, L.J., Gildea, R.J, Howard, J.A.K. & Puschmann, H.
   (2009), J. Appl. Cryst. 42, 339-341.
2. Sheldrick, G.M. (2015). Acta Cryst. A71, 3-8.
3. Sheldrick, G.M. (2015). Acta Cryst. C71, 3-8.

Crystal structure determination of
[c080620\_3\_2]

**Crystal Data**
for C46H25BF24N2 (*M*=1072.49 g/mol):
triclinic, space group P-1 (no. 2),
*a* = 12.3378(2) Å, *b* = 13.3787(2) Å, *c* = 13.4037(2) Å, *α* = 92.0660(10)°, *β* = 104.8350(10)°, *γ* = 90.4690(10)°,
*V*= 2136.98(6) Å3,
*Z* = 2,
*T* = 100.0(1) K,
μ(Cu Kα) = 1.543 mm-1,
*Dcalc* = 1.667 g/cm3,
56609 reflections measured (6.828° ≤ 2Θ ≤ 146.442°),
8190 unique (*R*int = 0.0442, Rsigma = 0.0242) which were used in all calculations.
The final *R*1 was 0.0341
(I > 2σ(I)) and *wR*2 was 0.0884 (all data).

Refinement model description

Number of restraints - 860,
number of constraints - unknown.

Details:

```
1. Fixed Uiso
```

This report has been created with Olex2, compiled on
2022.04.07 svn.rca3783a0 for OlexSys. Please
let us know
if there are any errors or if you would like to have additional features.
