# Supplementary material for: London Dispersion versus Intramolecular Hydrogen Bond in Bis‐Pyridines: How Accurate Is DFT for Competing Noncovalent Interactions in the Condensed Phase?
Source: Chemistry. 2025 Oct 23;31(66):e02745. doi: 10.1002/chem.202502745 (PMC12648470; doi:10.1002/chem.202502745)

checkCIF/PLATON report

Structure factors have been supplied for datablock(s) c080620_3_2

THIS REPORT IS FOR GUIDANCE ONLY. IF USED AS PART OF A REVIEW PROCEDURE FOR PUBLICATION, IT SHOULD NOT REPLACE THE EXPERTISE OF AN EXPERIENCED CRYSTALLOGRAPHIC REFEREE.

No syntax errors found. CIF dictionary Interpreting this report

Datablock: c080620_3_2

```
Bond precision:   C-C = 0.0021 A          Wavelength=1.54184

Cell:           a=12.3378(2)       b=13.3787(2)       c=13.4037(2)
                alpha=92.066(1)    beta=104.835(1)    gamma=90.469(1)
Temperature:    100 K

                 Calculated                   Reported
Volume           2136.98(6)                   2136.98(6)
Space group      P -1                         P -1
Hall group       -P 1                         -P 1
Moiety formula   C32 H12 B F24, C14 H13 N2    C32 H12 B F24, C14 H13 N2
Sum formula      C46 H25 B F24 N2             C46 H25 B F24 N2
Mr               1072.54                      1072.49
Dx,g cm-3        1.667                        1.667
Z                2                            2
Mu (mm-1)        1.544                        1.543
F000             1072.0                       1072.0
F000'            1077.21
h,k,lmax         15,16,16                     15,16,16
Nref             8582                         8190
Tmin,Tmax        0.634,0.868                  0.281,1.000
Tmin'            0.575

Correction method= # Reported T Limits: Tmin=0.281 Tmax=1.000
AbsCorr = GAUSSIAN

Data completeness= 0.954           Theta(max)= 73.221

R(reflections)= 0.0341( 7298)                        wR2(reflections)=
                                                     0.0884( 8190)
S = 1.035                    Npar= 784
```

The following ALERTS were generated. Each ALERT has the format

test-name_ALERT_alert-type_alert-level.

Click on the hyperlinks for more details of the test.

Alert level C

```
PLAT213_ALERT_2_C Atom F7A            has ADP max/min Ratio .....        3.3 prolat
PLAT213_ALERT_2_C Atom F8B            has ADP max/min Ratio .....        3.3 prolat
PLAT906_ALERT_3_C Large K Value in the Analysis of Variance ......     2.110 Check
PLAT911_ALERT_3_C Missing FCF Refl Between Thmin & STh/L=    0.600        13 Report
```

Alert level G

```
PLAT002_ALERT_2_G Number of Distance or Angle Restraints on AtSite        35 Note
PLAT003_ALERT_2_G Number of Uiso or Uij Restrained non-H Atoms ...        29 Report
PLAT154_ALERT_1_G The s.u.'s on the Cell Angles are Equal ..(Note)     0.001 Degree
PLAT171_ALERT_4_G The CIF-Embedded .res File Contains EADP Records         3 Report
PLAT172_ALERT_4_G The CIF-Embedded .res File Contains DFIX Records         3 Report
PLAT176_ALERT_4_G The CIF-Embedded .res File Contains SADI Records         8 Report
PLAT178_ALERT_4_G The CIF-Embedded .res File Contains SIMU Records         3 Report
PLAT187_ALERT_4_G The CIF-Embedded .res File Contains RIGU Records         3 Report
PLAT230_ALERT_2_G Hirshfeld Test Diff for    F7B      --C15      .       7.7 s.u.
PLAT242_ALERT_2_G Low    'MainMol' Ueq as Compared to Neighbors of        C7 Check
PLAT242_ALERT_2_G Low    'MainMol' Ueq as Compared to Neighbors of       C15 Check
PLAT242_ALERT_2_G Low    'MainMol' Ueq as Compared to Neighbors of       C31 Check
PLAT301_ALERT_3_G Main Residue  Disorder .............(Resd  1  )        16% Note
PLAT412_ALERT_2_G Short Intra XH3 .. XHn     H3B      ..H6BE     .      2.01 Ang.
                                                      x,y,z  =      1_555 Check
PLAT412_ALERT_2_G Short Intra XH3 .. XHn     H8BA     ..H10B     .      1.95 Ang.
                                                      x,y,z  =      1_555 Check
PLAT432_ALERT_2_G Short Inter X...Y Contact  F7B      ..C8B      .      2.94 Ang.
                                                   -1+x,y,z  =      1_455 Check
PLAT432_ALERT_2_G Short Inter X...Y Contact  F8B      ..C4       .      2.87 Ang.
                                                1-x,2-y,1-z  =      2_676 Check
PLAT720_ALERT_4_G Number of Unusual/Non-Standard Labels .........        13 Note
PLAT860_ALERT_3_G Number of Least-Squares Restraints ............       860 Note
PLAT910_ALERT_3_G Missing # of FCF Reflection(s) Below Theta(Min).         1 Note
PLAT912_ALERT_4_G Missing # of FCF Reflections Above STh/L=  0.600       379 Note
PLAT978_ALERT_2_G Number C-C Bonds with Positive Residual Density.        11 Info
PLAT992_ALERT_5_G Repd & Actual _reflns_number_gt Values Differ by         2 Check
```

0 **ALERT level A** = Most likely a serious problem - resolve or explain
0 **ALERT level B** = A potentially serious problem, consider carefully
4 **ALERT level C** = Check. Ensure it is not caused by an omission or oversight
23 **ALERT level G** = General information/check it is not something unexpected

1 ALERT type 1 CIF construction/syntax error, inconsistent or missing data
13 ALERT type 2 Indicator that the structure model may be wrong or deficient
5 ALERT type 3 Indicator that the structure quality may be low
7 ALERT type 4 Improvement, methodology, query or suggestion
1 ALERT type 5 Informative message, check

It is advisable to attempt to resolve as many as possible of the alerts in all categories. Often the minor alerts point to easily fixed oversights, errors and omissions in your CIF or refinement strategy, so attention to these fine details can be worthwhile. In order to resolve some of the more serious problems it may be necessary to carry out additional measurements or structure refinements. However, the purpose of your study may justify the reported deviations and the more serious of these should normally be commented upon in the discussion or experimental section of a paper or in the "special_details" fields of the CIF. checkCIF was carefully designed to identify outliers and unusual parameters, but every test has its limitations and alerts that are not important in a particular case may appear. Conversely, the absence of alerts does not guarantee there are no aspects of the results needing attention. It is up to the individual to critically assess their own results and, if necessary, seek expert advice.

Publication of your CIF in IUCr journals

A basic structural check has been run on your CIF. These basic checks will be run on all CIFs submitted for publication in IUCr journals (*Acta Crystallographica*, *Journal of Applied Crystallography*, *Journal of Synchrotron Radiation*); however, if you intend to submit to *Acta Crystallographica Section C* or *E* or *IUCrData*, you should make sure that full publication checks are run on the final version of your CIF prior to submission.

Publication of your CIF in other journals

Please refer to the *Notes for Authors* of the relevant journal for any special instructions relating to CIF submission.

PLATON version of 12/09/2022; check.def file version of 09/08/2022

Datablock c080620_3_2 - ellipsoid plot

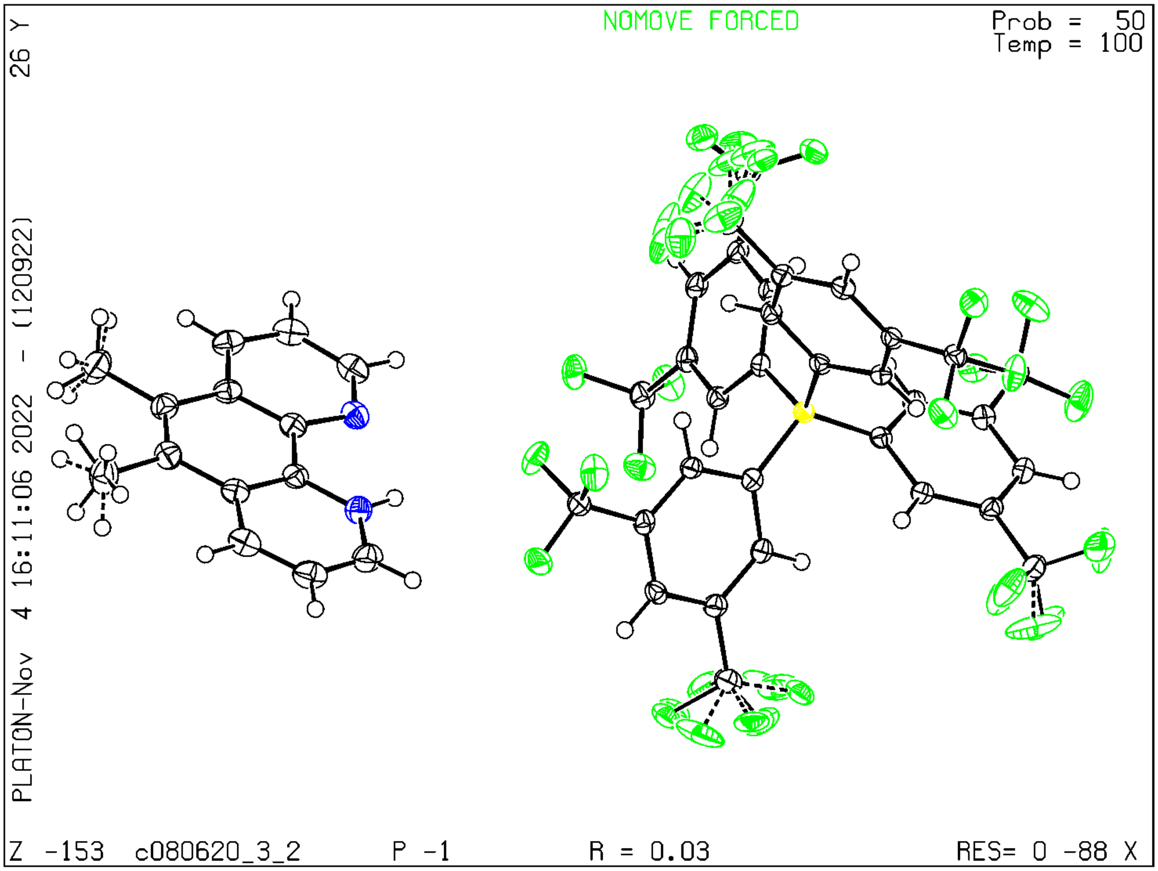

Supplement: Supplementary file 1 — Supporting Information [file CHEM-31-e02745-s002.zip › Crystal_structures/4b/checkcif.pdf]
