# Supplementary material for: London Dispersion versus Intramolecular Hydrogen Bond in Bis‐Pyridines: How Accurate Is DFT for Competing Noncovalent Interactions in the Condensed Phase?
Source: Chemistry. 2025 Oct 23;31(66):e02745. doi: 10.1002/chem.202502745 (PMC12648470; doi:10.1002/chem.202502745)
Supplement: Supplementary file 1 — Supporting Information [file CHEM-31-e02745-s002.zip › Crystal_structures/5b/c231122_1_1_tables.html]

c231122\_1\_1


# c231122\_1\_1

b"\n \n \n "

Table 1 Crystal data and structure refinement for c231122\_1\_1.

| Identification code | c231122\_1\_1 |
| Empirical formula | C43H23BF24N2 |
| Formula weight | 1034.44 |
| Temperature/K | 100.0(1) |
| Crystal system | monoclinic |
| Space group | P21/c |
| a/Å | 22.5581(2) |
| b/Å | 13.06430(10) |
| c/Å | 16.51850(10) |
| α/° | 90 |
| β/° | 90.2900(10) |
| γ/° | 90 |
| Volume/Å3 | 4868.03(6) |
| Z | 4 |
| ρcalcg/cm3 | 1.411 |
| μ/mm‑1 | 1.333 |
| F(000) | 2064.0 |
| Crystal size/mm3 | 0.304 × 0.223 × 0.187 |
| Radiation | Cu Kα (λ = 1.54184) |
| 2Θ range for data collection/° | 7.82 to 159.404 |
| Index ranges | -28 ≤ h ≤ 28, -16 ≤ k ≤ 16, -21 ≤ l ≤ 19 |
| Reflections collected | 72349 |
| Independent reflections | 10464 [Rint = 0.0462, Rsigma = 0.0294] |
| Data/restraints/parameters | 10464/844/770 |
| Goodness-of-fit on F2 | 1.040 |
| Final R indexes [I>=2σ (I)] | R1 = 0.0479, wR2 = 0.1216 |
| Final R indexes [all data] | R1 = 0.0528, wR2 = 0.1250 |
| Largest diff. peak/hole / e Å-3 | 0.40/-0.31 |

Table 2 Fractional Atomic Coordinates (×104) and Equivalent Isotropic Displacement Parameters (Å2×103) for c231122\_1\_1. Ueq is defined as 1/3 of the trace of the orthogonalised UIJ tensor.

| Atom | *x* | *y* | *z* | U(eq) |
| --- | --- | --- | --- | --- |
| F1 | 5767.9(7) | 6061.7(13) | 3068.6(11) | 75.5(5) |
| F2 | 5751.5(7) | 7633.5(12) | 2721.3(10) | 69.5(4) |
| F3 | 6370.7(7) | 6610.4(14) | 2183.0(9) | 70.1(5) |
| F4 | 6342(2) | 9357(6) | 5274(5) | 80(2) |
| F5 | 7166(4) | 9880(6) | 4758(5) | 87(2) |
| F6 | 7165(2) | 8990(5) | 5824(3) | 48.0(10) |
| F7 | 8485.7(5) | 6708.7(9) | 1246.0(6) | 39.3(3) |
| F8 | 9375.6(6) | 7291.9(9) | 1217.9(6) | 44.5(3) |
| F9 | 9213.3(5) | 5696.9(8) | 1457.4(6) | 38.0(2) |
| F10 | 9267(2) | 9299(4) | 4477(4) | 62.2(12) |
| F11 | 9969(4) | 9163(6) | 3599(4) | 82(2) |
| F12 | 9969(3) | 8257(7) | 4670(5) | 44.5(14) |
| F13 | 10081.7(5) | 5660.9(11) | 6124.8(12) | 71.7(5) |
| F14 | 9832.9(7) | 4718.7(17) | 7109.9(8) | 81.2(6) |
| F15 | 9839.4(6) | 4135.0(10) | 5909.5(9) | 54.8(3) |
| F16 | 7240(4) | 6342(7) | 7301(6) | 87(2) |
| F17 | 7935(3) | 6008(6) | 8123(4) | 76(2) |
| F18 | 7438(3) | 4818(3) | 7594(5) | 62.0(15) |
| F19 | 9060(3) | 2949(3) | 2597(3) | 49.3(10) |
| F20 | 9216(4) | 2188(8) | 3714(4) | 50.4(15) |
| F21 | 8614(3) | 1566(4) | 2843(3) | 52.9(10) |
| F22 | 6654(7) | 2598(10) | 5110(4) | 75(2) |
| F23 | 6677(3) | 1681(4) | 4057(5) | 60.1(16) |
| F24 | 6288(3) | 3148(5) | 4007(3) | 47.6(10) |
| C1 | 7454.3(7) | 6563.3(12) | 4257.2(9) | 23.1(3) |
| C2 | 7023.3(7) | 6400.5(12) | 3660.5(10) | 26.8(3) |
| C3 | 6558.9(7) | 7084.6(14) | 3528.3(11) | 31.4(4) |
| C4 | 6118.7(9) | 6857.8(17) | 2878.8(14) | 44.4(5) |
| C5 | 6501.4(8) | 7962.7(14) | 3993.8(12) | 33.7(4) |
| C6 | 6923.9(8) | 8140.6(13) | 4585.7(11) | 30.8(4) |
| C7 | 6894.4(10) | 9096.4(15) | 5081.3(14) | 44.0(5) |
| C8 | 7392.4(7) | 7463.4(12) | 4713.2(10) | 26.7(3) |
| C9 | 8539.0(7) | 6444.1(11) | 3803.4(9) | 21.7(3) |
| C10 | 8592.8(7) | 6296.0(12) | 2968.5(9) | 23.0(3) |
| C11 | 8985.1(7) | 6864.6(12) | 2498.5(9) | 23.3(3) |
| C12 | 9022.4(8) | 6647.1(12) | 1612.6(10) | 28.2(3) |
| C13 | 9339.1(7) | 7614.5(12) | 2845.4(10) | 25.3(3) |
| C14 | 9281.1(7) | 7795.3(12) | 3666.1(10) | 25.2(3) |
| C15 | 9633.6(9) | 8627.7(14) | 4065.5(11) | 36.0(4) |
| C16 | 8892.3(7) | 7222.4(12) | 4134.8(9) | 23.4(3) |
| C17 | 8276.0(7) | 5733.3(11) | 5288.5(9) | 21.5(3) |
| C18 | 8862.9(7) | 5447.5(11) | 5452.1(9) | 21.8(3) |
| C19 | 9069.4(7) | 5267.7(12) | 6236.9(9) | 24.4(3) |
| C20 | 9701.0(8) | 4942.1(14) | 6353.5(10) | 30.7(3) |
| C21 | 8696.3(7) | 5377.3(12) | 6896.4(9) | 25.3(3) |
| C22 | 8111.9(7) | 5649.6(12) | 6750.2(10) | 25.5(3) |
| C23 | 7689.7(8) | 5731.2(14) | 7443.5(11) | 34.2(4) |
| C24 | 7903.9(7) | 5816.8(11) | 5962.9(9) | 23.2(3) |
| C25 | 7932.0(7) | 4660.9(12) | 4068.3(9) | 22.2(3) |
| C26 | 8374.8(7) | 4053.2(12) | 3716.8(9) | 23.7(3) |
| C27 | 8291.0(7) | 3011.3(12) | 3562.1(10) | 27.3(3) |
| C28 | 8793.1(8) | 2412.1(14) | 3205.9(12) | 35.9(4) |
| C29 | 7759.7(8) | 2529.3(12) | 3748.2(10) | 29.3(3) |
| C30 | 7317.9(7) | 3114.7(13) | 4097.1(10) | 28.1(3) |
| C31 | 6740.5(8) | 2631.4(14) | 4330.5(12) | 35.8(4) |
| C32 | 7402.3(7) | 4150.9(12) | 4258.4(10) | 24.9(3) |
| B1 | 8045.5(7) | 5847.0(13) | 4352.5(10) | 21.5(3) |
| N1B | 2187.4(7) | 7462.0(11) | 3646.2(9) | 31.6(3) |
| N2B | 3237.5(8) | 7304.4(14) | 2978.0(11) | 42.1(4) |
| C1B | 1758.6(8) | 6986.4(14) | 4067.2(11) | 32.9(4) |
| C2B | 1334.2(8) | 7543.9(14) | 4454.6(11) | 33.9(4) |
| C3B | 1365.0(8) | 8606.8(15) | 4421.7(12) | 37.2(4) |
| C4B | 1815.2(8) | 9072.7(14) | 3993.8(12) | 36.1(4) |
| C5B | 2230.2(8) | 8486.0(13) | 3596.2(11) | 31.4(4) |
| C6B | 2718.5(9) | 8922.6(15) | 3073.7(12) | 37.4(4) |
| C7B | 3285.8(9) | 8326.6(16) | 3051.5(11) | 38.5(4) |
| C8B | 3827.7(11) | 8815(2) | 3083.4(17) | 57.8(6) |
| C9B | 4333.6(12) | 8225(3) | 3025(2) | 75.9(9) |
| C10B | 4285.5(11) | 7180(2) | 2942.3(19) | 66.0(7) |
| C11B | 3732.8(10) | 6746(2) | 2927.9(15) | 50.9(5) |
| F21A | 8661(4) | 1399(6) | 3181(9) | 114(4) |
| F20A | 9262(5) | 2419(12) | 3697(7) | 56(3) |
| F19A | 8957(5) | 2693(9) | 2517(4) | 100(3) |
| F4A | 6964(6) | 8927(8) | 5839(5) | 93(3) |
| F5A | 6383(3) | 9618(7) | 4963(7) | 74(2) |
| F6A | 7299(4) | 9779(7) | 4836(6) | 59.3(18) |
| F23A | 6626(5) | 1776(6) | 3940(5) | 65(2) |
| F22A | 6744(11) | 2392(15) | 5112(5) | 86(4) |
| F24A | 6269(4) | 3222(7) | 4233(9) | 104(4) |
| F18A | 7336(5) | 4958(7) | 7501(6) | 82(3) |
| F17A | 7964(5) | 5786(9) | 8153(5) | 71(2) |
| F16A | 7360(7) | 6546(9) | 7400(9) | 99(4) |
| F12A | 10103(3) | 8882(7) | 3572(5) | 45.8(15) |
| F11A | 9334(3) | 9478(5) | 4114(6) | 67.8(19) |
| F10A | 9878(5) | 8382(9) | 4756(6) | 50(2) |

Table 3 Anisotropic Displacement Parameters (Å2×103) for c231122\_1\_1. The Anisotropic displacement factor exponent takes the form: -2π2[h2a\*2U11+2hka\*b\*U12+…].

| Atom | U11 | U22 | U33 | U23 | U13 | U12 |
| --- | --- | --- | --- | --- | --- | --- |
| F1 | 56.2(8) | 70.7(10) | 99.2(12) | 4.3(9) | -34.9(8) | -23.3(8) |
| F2 | 57.8(8) | 64.1(9) | 86.1(11) | -11.4(8) | -39.5(8) | 26.8(7) |
| F3 | 56.1(8) | 104.2(13) | 49.6(8) | -20.3(8) | -23.5(6) | 18.3(8) |
| F4 | 47.8(18) | 84(5) | 109(5) | -58(3) | -16(2) | 34(2) |
| F5 | 160(6) | 23.7(16) | 78(3) | 5.1(17) | 14(4) | -4(3) |
| F6 | 57.8(18) | 37.3(16) | 48.7(19) | -16.3(13) | -10.8(13) | 9.1(13) |
| F7 | 44.5(6) | 48.4(6) | 24.8(5) | 0.4(4) | -5.7(4) | 10.1(5) |
| F8 | 65.4(8) | 40.1(6) | 28.2(5) | 0.4(4) | 16.6(5) | -15.3(5) |
| F9 | 55.1(7) | 26.6(5) | 32.3(5) | -3.2(4) | 3.7(5) | 11.9(5) |
| F10 | 64.8(19) | 31.6(19) | 90(3) | -28.2(19) | -15(2) | -0.2(15) |
| F11 | 137(6) | 71(4) | 38.2(18) | 6(2) | 1(3) | -77(4) |
| F12 | 38.0(16) | 53(2) | 43(3) | -6.4(18) | -12.3(17) | -7.7(13) |
| F13 | 26.6(6) | 46.6(8) | 141.9(15) | 15.5(8) | -8.9(7) | -2.1(5) |
| F14 | 58.7(8) | 153.9(17) | 30.8(6) | 7.0(8) | -10.2(6) | 51.7(10) |
| F15 | 43.0(6) | 48.0(7) | 73.2(9) | -24.9(6) | -15.4(6) | 21.0(5) |
| F16 | 81(3) | 133(6) | 46(2) | 28(4) | 33.2(18) | 71(3) |
| F17 | 62(3) | 129(4) | 38(3) | -48(3) | 21.8(18) | -48(3) |
| F18 | 69(3) | 42.2(17) | 76(3) | 13.0(18) | 38(2) | -7.0(16) |
| F19 | 62.0(19) | 37.1(14) | 49(2) | -2.2(11) | 26.4(16) | 9.1(13) |
| F20 | 54(3) | 65(4) | 32.9(19) | -7.8(17) | -6.0(14) | 31(2) |
| F21 | 54.9(17) | 36.2(18) | 67(2) | -32.3(13) | -3.4(14) | 4.6(13) |
| F22 | 59(4) | 127(4) | 38(2) | 6(2) | 6.1(18) | -54(3) |
| F23 | 49(2) | 21.8(15) | 110(5) | 2.5(17) | 7(3) | -10.8(14) |
| F24 | 27.0(17) | 51(2) | 64.3(19) | 14.9(12) | -6.4(12) | -7.1(13) |
| C1 | 23.1(7) | 20.8(7) | 25.3(7) | 2.2(6) | 2.1(6) | -1.6(6) |
| C2 | 25.5(7) | 23.8(7) | 31.0(8) | 0.7(6) | -0.6(6) | -0.1(6) |
| C3 | 24.1(8) | 31.2(8) | 39.0(9) | 2.7(7) | -2.6(7) | 0.7(6) |
| C4 | 33.4(9) | 44.5(11) | 55.0(12) | -2.7(9) | -14.1(9) | 8.0(8) |
| C5 | 27.4(8) | 28.4(8) | 45.3(10) | 2.6(7) | 0.1(7) | 6.2(7) |
| C6 | 30.9(8) | 23.5(8) | 38.2(9) | -0.8(7) | 3.0(7) | 3.3(6) |
| C7 | 49.0(11) | 31.3(9) | 51.8(12) | -8.9(8) | -3.3(9) | 12.1(8) |
| C8 | 27.2(8) | 22.9(7) | 29.9(8) | -0.1(6) | 0.5(6) | 0.2(6) |
| C9 | 21.7(7) | 19.1(7) | 24.2(7) | 1.4(5) | -1.9(6) | 1.5(5) |
| C10 | 24.6(7) | 19.4(7) | 25.0(7) | 0.1(6) | -1.8(6) | 0.2(6) |
| C11 | 26.1(7) | 20.0(7) | 23.7(7) | 2.4(6) | 0.8(6) | 3.5(6) |
| C12 | 34.2(8) | 21.4(7) | 29.1(8) | 1.9(6) | 3.7(6) | 1.5(6) |
| C13 | 24.7(7) | 22.6(7) | 28.7(8) | 6.5(6) | 0.8(6) | -0.4(6) |
| C14 | 26.9(7) | 19.6(7) | 29.0(8) | 2.5(6) | -3.9(6) | -2.3(6) |
| C15 | 41.0(10) | 30.4(9) | 36.7(9) | 1.7(7) | -4.8(7) | -12.7(7) |
| C16 | 27.0(7) | 20.3(7) | 22.7(7) | 1.3(6) | -1.9(6) | 0.3(6) |
| C17 | 26.4(7) | 15.5(6) | 22.8(7) | -0.8(5) | 1.1(6) | -1.8(5) |
| C18 | 26.2(7) | 17.9(7) | 21.5(7) | 0.3(5) | 1.4(6) | -0.1(5) |
| C19 | 30.0(8) | 19.5(7) | 23.6(7) | 0.2(6) | -2.6(6) | -0.1(6) |
| C20 | 34.1(9) | 31.6(8) | 26.4(8) | -1.9(7) | -5.8(6) | 4.7(7) |
| C21 | 35.8(8) | 18.9(7) | 21.2(7) | 0.5(6) | -2.0(6) | -1.8(6) |
| C22 | 34.8(8) | 18.0(7) | 23.9(7) | -2.0(6) | 4.5(6) | -1.6(6) |
| C23 | 39.9(9) | 35.8(9) | 26.9(8) | -1.9(7) | 6.6(7) | -0.3(7) |
| C24 | 26.0(7) | 18.4(7) | 25.2(7) | -0.3(6) | 2.3(6) | -0.1(6) |
| C25 | 24.0(7) | 22.3(7) | 20.2(7) | 1.1(5) | -2.5(5) | -0.4(6) |
| C26 | 24.5(7) | 23.5(7) | 23.2(7) | -1.7(6) | -2.8(6) | 0.2(6) |
| C27 | 31.3(8) | 24.5(8) | 25.9(8) | -4.2(6) | -5.6(6) | 3.4(6) |
| C28 | 39.0(9) | 29.6(9) | 38.9(10) | -12.6(7) | -3.8(7) | 5.3(7) |
| C29 | 37.2(9) | 20.2(7) | 30.3(8) | -1.7(6) | -8.3(7) | -1.5(6) |
| C30 | 30.9(8) | 24.8(8) | 28.4(8) | 3.6(6) | -5.4(6) | -5.3(6) |
| C31 | 35.7(9) | 30.3(9) | 41.2(10) | 4.2(7) | -3.4(7) | -9.5(7) |
| C32 | 26.3(7) | 22.9(7) | 25.5(7) | 1.7(6) | 0.0(6) | -0.4(6) |
| B1 | 22.1(8) | 19.7(8) | 22.7(8) | -0.6(6) | 0.2(6) | -0.7(6) |
| N1B | 35.1(8) | 24.4(7) | 35.3(8) | 1.2(6) | -0.6(6) | -1.5(6) |
| N2B | 37.2(8) | 45.8(10) | 43.3(9) | -2.3(7) | 1.4(7) | -2.4(7) |
| C1B | 38.6(9) | 25.6(8) | 34.6(9) | 4.2(7) | -2.6(7) | -4.1(7) |
| C2B | 35.7(9) | 33.7(9) | 32.3(9) | 1.2(7) | -2.6(7) | -5.3(7) |
| C3B | 33.6(9) | 34.2(9) | 43.6(10) | -5.6(8) | -5.0(8) | 0.6(7) |
| C4B | 37.0(9) | 23.5(8) | 47.7(11) | 1.2(7) | -8.0(8) | -1.4(7) |
| C5B | 34.4(9) | 25.1(8) | 34.5(9) | 3.7(7) | -8.6(7) | -4.4(7) |
| C6B | 45.8(10) | 33.0(9) | 33.3(9) | 4.5(7) | -6.2(8) | -9.0(8) |
| C7B | 37.3(10) | 46.9(11) | 31.5(9) | 5.7(8) | -2.3(7) | -8.3(8) |
| C8B | 43.9(12) | 59.5(15) | 70.1(16) | 5.1(12) | -2.3(11) | -15.1(11) |
| C9B | 37.3(12) | 81(2) | 109(3) | -2.3(18) | -0.3(14) | -12.4(13) |
| C10B | 36.5(12) | 81.6(19) | 80.0(19) | -8.5(15) | -0.9(11) | 4.1(12) |
| C11B | 42.0(11) | 56.9(13) | 53.8(13) | -6.4(11) | 1.7(9) | 3.7(10) |
| F21A | 65(4) | 36(3) | 242(11) | -56(5) | 43(6) | -4(3) |
| F20A | 36(2) | 62(5) | 69(4) | -26(3) | -14(2) | 21(3) |
| F19A | 108(6) | 166(8) | 25(2) | 6(4) | 12(3) | 93(5) |
| F4A | 188(10) | 52(4) | 39(3) | -12(2) | 12(4) | 42(6) |
| F5A | 67(3) | 39(3) | 116(7) | -29(3) | -9(3) | 27(2) |
| F6A | 65(3) | 34(4) | 79(4) | -25(3) | -13(2) | -10(2) |
| F23A | 84(5) | 73(5) | 39(2) | -17(3) | 5(2) | -54(4) |
| F22A | 63(7) | 159(10) | 35(3) | 17(4) | -1(3) | -56(6) |
| F24A | 33(3) | 43(3) | 237(11) | 39(5) | 25(5) | -3(2) |
| F18A | 73(4) | 123(6) | 51(3) | -39(4) | 35(3) | -66(5) |
| F17A | 67(4) | 125(6) | 20(3) | 9(3) | 7(2) | 17(3) |
| F16A | 154(10) | 75(4) | 70(6) | 19(3) | 69(5) | 71(5) |
| F12A | 48(2) | 52(4) | 38(2) | 5(2) | -1.8(14) | -35(2) |
| F11A | 66(3) | 30(2) | 108(6) | -24(3) | -20(3) | -2.1(19) |
| F10A | 72(5) | 57(5) | 20.3(19) | 4(3) | -4(2) | -39(4) |

Table 4 Bond Lengths for c231122\_1\_1.

| Atom | Atom | Length/Å |  | Atom | Atom | Length/Å |
| --- | --- | --- | --- | --- | --- | --- |
| F1 | C4 | 1.345(3) |  | C15 | F12A | 1.380(6) |
| F2 | C4 | 1.334(2) |  | C15 | F11A | 1.303(6) |
| F3 | C4 | 1.325(3) |  | C15 | F10A | 1.304(8) |
| F4 | C7 | 1.333(5) |  | C17 | C18 | 1.401(2) |
| F5 | C7 | 1.308(6) |  | C17 | C24 | 1.403(2) |
| F6 | C7 | 1.375(5) |  | C17 | B1 | 1.635(2) |
| F7 | C12 | 1.353(2) |  | C18 | C19 | 1.395(2) |
| F8 | C12 | 1.3323(19) |  | C19 | C20 | 1.498(2) |
| F9 | C12 | 1.3391(19) |  | C19 | C21 | 1.387(2) |
| F10 | C15 | 1.386(5) |  | C21 | C22 | 1.386(2) |
| F11 | C15 | 1.288(6) |  | C22 | C23 | 1.497(2) |
| F12 | C15 | 1.341(6) |  | C22 | C24 | 1.397(2) |
| F13 | C20 | 1.329(2) |  | C23 | F18A | 1.290(7) |
| F14 | C20 | 1.316(2) |  | C23 | F17A | 1.324(7) |
| F15 | C20 | 1.323(2) |  | C23 | F16A | 1.300(8) |
| F16 | C23 | 1.311(6) |  | C25 | C26 | 1.404(2) |
| F17 | C23 | 1.300(5) |  | C25 | C32 | 1.405(2) |
| F18 | C23 | 1.346(5) |  | C25 | B1 | 1.639(2) |
| F19 | C28 | 1.368(4) |  | C26 | C27 | 1.398(2) |
| F20 | C28 | 1.301(6) |  | C27 | C28 | 1.500(2) |
| F21 | C28 | 1.320(4) |  | C27 | C29 | 1.389(2) |
| F22 | C31 | 1.304(8) |  | C28 | F21A | 1.357(7) |
| F23 | C31 | 1.328(5) |  | C28 | F20A | 1.328(8) |
| F24 | C31 | 1.334(5) |  | C28 | F19A | 1.252(7) |
| C1 | C2 | 1.397(2) |  | C29 | C30 | 1.384(2) |
| C1 | C8 | 1.404(2) |  | C30 | C31 | 1.500(2) |
| C1 | B1 | 1.636(2) |  | C30 | C32 | 1.393(2) |
| C2 | C3 | 1.393(2) |  | C31 | F23A | 1.315(7) |
| C3 | C4 | 1.488(3) |  | C31 | F22A | 1.328(10) |
| C3 | C5 | 1.388(3) |  | C31 | F24A | 1.324(8) |
| C5 | C6 | 1.382(3) |  | N1B | C1B | 1.346(2) |
| C6 | C7 | 1.495(3) |  | N1B | C5B | 1.344(2) |
| C6 | C8 | 1.394(2) |  | N2B | C7B | 1.345(3) |
| C7 | F4A | 1.280(7) |  | N2B | C11B | 1.337(3) |
| C7 | F5A | 1.354(7) |  | C1B | C2B | 1.365(3) |
| C7 | F6A | 1.341(7) |  | C2B | C3B | 1.391(3) |
| C9 | C10 | 1.399(2) |  | C3B | C4B | 1.382(3) |
| C9 | C16 | 1.401(2) |  | C4B | C5B | 1.379(3) |
| C9 | B1 | 1.637(2) |  | C5B | C6B | 1.514(3) |
| C10 | C11 | 1.394(2) |  | C6B | C7B | 1.499(3) |
| C11 | C12 | 1.494(2) |  | C7B | C8B | 1.380(3) |
| C11 | C13 | 1.386(2) |  | C8B | C9B | 1.381(4) |
| C13 | C14 | 1.383(2) |  | C9B | C10B | 1.375(4) |
| C14 | C15 | 1.498(2) |  | C10B | C11B | 1.370(3) |
| C14 | C16 | 1.391(2) |  |  |  |  |

Table 5 Bond Angles for c231122\_1\_1.

| Atom | Atom | Atom | Angle/˚ |  | Atom | Atom | Atom | Angle/˚ |
| --- | --- | --- | --- | --- | --- | --- | --- | --- |
| C2 | C1 | C8 | 115.80(14) |  | C21 | C22 | C24 | 121.13(14) |
| C2 | C1 | B1 | 123.01(14) |  | C24 | C22 | C23 | 119.27(15) |
| C8 | C1 | B1 | 120.71(14) |  | F16 | C23 | F18 | 104.2(5) |
| C3 | C2 | C1 | 122.20(16) |  | F16 | C23 | C22 | 113.6(5) |
| C2 | C3 | C4 | 118.93(17) |  | F17 | C23 | F16 | 108.2(5) |
| C5 | C3 | C2 | 121.05(16) |  | F17 | C23 | F18 | 105.4(4) |
| C5 | C3 | C4 | 120.02(16) |  | F17 | C23 | C22 | 114.2(4) |
| F1 | C4 | C3 | 112.16(18) |  | F18 | C23 | C22 | 110.5(4) |
| F2 | C4 | F1 | 105.51(17) |  | F18A | C23 | C22 | 113.4(4) |
| F2 | C4 | C3 | 113.64(18) |  | F18A | C23 | F17A | 105.2(6) |
| F3 | C4 | F1 | 105.61(19) |  | F18A | C23 | F16A | 107.0(8) |
| F3 | C4 | F2 | 106.56(19) |  | F17A | C23 | C22 | 112.6(5) |
| F3 | C4 | C3 | 112.73(17) |  | F16A | C23 | C22 | 112.4(8) |
| C6 | C5 | C3 | 117.72(16) |  | F16A | C23 | F17A | 105.6(7) |
| C5 | C6 | C7 | 119.72(16) |  | C22 | C24 | C17 | 121.84(15) |
| C5 | C6 | C8 | 121.34(16) |  | C26 | C25 | C32 | 115.59(14) |
| C8 | C6 | C7 | 118.91(17) |  | C26 | C25 | B1 | 122.87(13) |
| F4 | C7 | F6 | 103.0(4) |  | C32 | C25 | B1 | 121.07(13) |
| F4 | C7 | C6 | 112.8(4) |  | C27 | C26 | C25 | 122.04(15) |
| F5 | C7 | F4 | 109.8(5) |  | C26 | C27 | C28 | 118.58(15) |
| F5 | C7 | F6 | 103.7(4) |  | C29 | C27 | C26 | 121.13(15) |
| F5 | C7 | C6 | 114.1(5) |  | C29 | C27 | C28 | 120.28(15) |
| F6 | C7 | C6 | 112.5(3) |  | F19 | C28 | C27 | 110.9(3) |
| F4A | C7 | C6 | 112.7(5) |  | F20 | C28 | F19 | 105.5(5) |
| F4A | C7 | F5A | 109.2(6) |  | F20 | C28 | F21 | 109.1(5) |
| F4A | C7 | F6A | 109.3(6) |  | F20 | C28 | C27 | 114.6(4) |
| F5A | C7 | C6 | 112.4(5) |  | F21 | C28 | F19 | 103.4(3) |
| F6A | C7 | C6 | 111.0(5) |  | F21 | C28 | C27 | 112.6(3) |
| F6A | C7 | F5A | 101.7(5) |  | F21A | C28 | C27 | 110.8(4) |
| C6 | C8 | C1 | 121.88(16) |  | F20A | C28 | C27 | 110.9(6) |
| C10 | C9 | C16 | 115.69(14) |  | F20A | C28 | F21A | 101.5(7) |
| C10 | C9 | B1 | 122.89(13) |  | F19A | C28 | C27 | 115.5(5) |
| C16 | C9 | B1 | 121.08(13) |  | F19A | C28 | F21A | 108.9(6) |
| C11 | C10 | C9 | 122.26(14) |  | F19A | C28 | F20A | 108.4(7) |
| C10 | C11 | C12 | 118.90(14) |  | C30 | C29 | C27 | 117.73(15) |
| C13 | C11 | C10 | 120.81(14) |  | C29 | C30 | C31 | 120.17(15) |
| C13 | C11 | C12 | 120.29(14) |  | C29 | C30 | C32 | 121.25(15) |
| F7 | C12 | C11 | 111.86(13) |  | C32 | C30 | C31 | 118.56(16) |
| F8 | C12 | F7 | 106.22(14) |  | F22 | C31 | F23 | 106.7(6) |
| F8 | C12 | F9 | 107.38(14) |  | F22 | C31 | F24 | 107.1(7) |
| F8 | C12 | C11 | 113.33(14) |  | F22 | C31 | C30 | 113.8(7) |
| F9 | C12 | F7 | 104.91(14) |  | F23 | C31 | F24 | 104.9(4) |
| F9 | C12 | C11 | 112.57(13) |  | F23 | C31 | C30 | 113.4(4) |
| C14 | C13 | C11 | 117.95(14) |  | F24 | C31 | C30 | 110.4(3) |
| C13 | C14 | C15 | 120.19(15) |  | F23A | C31 | C30 | 113.6(5) |
| C13 | C14 | C16 | 121.11(15) |  | F23A | C31 | F22A | 106.0(9) |
| C16 | C14 | C15 | 118.70(15) |  | F23A | C31 | F24A | 106.3(6) |
| F10 | C15 | C14 | 111.0(3) |  | F22A | C31 | C30 | 110.4(11) |
| F11 | C15 | F10 | 107.6(4) |  | F24A | C31 | C30 | 114.9(5) |
| F11 | C15 | F12 | 108.1(5) |  | F24A | C31 | F22A | 104.9(9) |
| F11 | C15 | C14 | 116.3(4) |  | C30 | C32 | C25 | 122.26(15) |
| F12 | C15 | F10 | 101.5(4) |  | C1 | B1 | C9 | 103.33(12) |
| F12 | C15 | C14 | 111.2(5) |  | C1 | B1 | C25 | 112.78(12) |
| F12A | C15 | C14 | 108.8(4) |  | C9 | B1 | C25 | 113.42(12) |
| F11A | C15 | C14 | 111.9(4) |  | C17 | B1 | C1 | 113.45(12) |
| F11A | C15 | F12A | 103.3(5) |  | C17 | B1 | C9 | 110.68(12) |
| F11A | C15 | F10A | 111.9(6) |  | C17 | B1 | C25 | 103.50(12) |
| F10A | C15 | C14 | 115.3(6) |  | C5B | N1B | C1B | 122.90(16) |
| F10A | C15 | F12A | 104.7(6) |  | C11B | N2B | C7B | 118.66(19) |
| C14 | C16 | C9 | 122.13(15) |  | N1B | C1B | C2B | 120.23(17) |
| C18 | C17 | C24 | 115.87(14) |  | C1B | C2B | C3B | 118.62(17) |
| C18 | C17 | B1 | 120.16(13) |  | C4B | C3B | C2B | 119.78(18) |
| C24 | C17 | B1 | 123.72(14) |  | C5B | C4B | C3B | 120.09(17) |
| C19 | C18 | C17 | 122.35(14) |  | N1B | C5B | C4B | 118.36(17) |
| C18 | C19 | C20 | 118.69(14) |  | N1B | C5B | C6B | 117.55(17) |
| C21 | C19 | C18 | 120.74(15) |  | C4B | C5B | C6B | 124.03(16) |
| C21 | C19 | C20 | 120.57(14) |  | C7B | C6B | C5B | 116.23(16) |
| F13 | C20 | C19 | 112.23(14) |  | N2B | C7B | C6B | 116.69(17) |
| F14 | C20 | F13 | 106.45(17) |  | N2B | C7B | C8B | 122.3(2) |
| F14 | C20 | F15 | 107.27(16) |  | C8B | C7B | C6B | 121.0(2) |
| F14 | C20 | C19 | 113.29(15) |  | C7B | C8B | C9B | 118.1(3) |
| F15 | C20 | F13 | 104.54(16) |  | C10B | C9B | C8B | 119.7(2) |
| F15 | C20 | C19 | 112.46(14) |  | C11B | C10B | C9B | 118.9(3) |
| C22 | C21 | C19 | 118.04(15) |  | N2B | C11B | C10B | 122.3(2) |
| C21 | C22 | C23 | 119.56(15) |  |  |  |  |  |

Table 6 Torsion Angles for c231122\_1\_1.

| A | B | C | D | Angle/˚ |  | A | B | C | D | Angle/˚ |
| --- | --- | --- | --- | --- | --- | --- | --- | --- | --- | --- |
| C1 | C2 | C3 | C4 | 179.80(17) |  | C21 | C22 | C23 | F17 | 30.7(5) |
| C1 | C2 | C3 | C5 | -0.5(3) |  | C21 | C22 | C23 | F18 | -87.9(4) |
| C2 | C1 | C8 | C6 | 1.1(2) |  | C21 | C22 | C23 | F18A | -102.9(6) |
| C2 | C1 | B1 | C9 | 92.02(17) |  | C21 | C22 | C23 | F17A | 16.4(6) |
| C2 | C1 | B1 | C17 | -148.09(14) |  | C21 | C22 | C23 | F16A | 135.6(8) |
| C2 | C1 | B1 | C25 | -30.8(2) |  | C21 | C22 | C24 | C17 | -1.0(2) |
| C2 | C3 | C4 | F1 | 70.4(2) |  | C23 | C22 | C24 | C17 | -178.86(15) |
| C2 | C3 | C4 | F2 | -170.05(18) |  | C24 | C17 | C18 | C19 | -0.7(2) |
| C2 | C3 | C4 | F3 | -48.7(3) |  | C24 | C17 | B1 | C1 | 27.0(2) |
| C2 | C3 | C5 | C6 | 0.7(3) |  | C24 | C17 | B1 | C9 | 142.62(14) |
| C3 | C5 | C6 | C7 | 177.63(18) |  | C24 | C17 | B1 | C25 | -95.55(16) |
| C3 | C5 | C6 | C8 | 0.0(3) |  | C24 | C22 | C23 | F16 | -26.7(6) |
| C4 | C3 | C5 | C6 | -179.62(18) |  | C24 | C22 | C23 | F17 | -151.4(4) |
| C5 | C3 | C4 | F1 | -109.3(2) |  | C24 | C22 | C23 | F18 | 90.0(4) |
| C5 | C3 | C4 | F2 | 10.3(3) |  | C24 | C22 | C23 | F18A | 75.0(6) |
| C5 | C3 | C4 | F3 | 131.7(2) |  | C24 | C22 | C23 | F17A | -165.6(5) |
| C5 | C6 | C7 | F4 | 38.8(5) |  | C24 | C22 | C23 | F16A | -46.4(8) |
| C5 | C6 | C7 | F5 | -87.3(5) |  | C25 | C26 | C27 | C28 | -178.36(15) |
| C5 | C6 | C7 | F6 | 154.8(3) |  | C25 | C26 | C27 | C29 | 0.3(2) |
| C5 | C6 | C7 | F4A | 133.6(7) |  | C26 | C25 | C32 | C30 | -1.0(2) |
| C5 | C6 | C7 | F5A | 9.7(5) |  | C26 | C25 | B1 | C1 | 147.73(14) |
| C5 | C6 | C7 | F6A | -103.5(5) |  | C26 | C25 | B1 | C9 | 30.7(2) |
| C5 | C6 | C8 | C1 | -0.9(3) |  | C26 | C25 | B1 | C17 | -89.27(16) |
| C7 | C6 | C8 | C1 | -178.59(16) |  | C26 | C27 | C28 | F19 | -43.5(3) |
| C8 | C1 | C2 | C3 | -0.4(2) |  | C26 | C27 | C28 | F20 | 75.8(5) |
| C8 | C1 | B1 | C9 | -79.69(17) |  | C26 | C27 | C28 | F21 | -158.7(3) |
| C8 | C1 | B1 | C17 | 40.21(19) |  | C26 | C27 | C28 | F21A | 173.1(7) |
| C8 | C1 | B1 | C25 | 157.47(14) |  | C26 | C27 | C28 | F20A | 61.2(7) |
| C8 | C6 | C7 | F4 | -143.5(4) |  | C26 | C27 | C28 | F19A | -62.6(7) |
| C8 | C6 | C7 | F5 | 90.4(5) |  | C26 | C27 | C29 | C30 | -0.4(2) |
| C8 | C6 | C7 | F6 | -27.5(3) |  | C27 | C29 | C30 | C31 | -178.46(15) |
| C8 | C6 | C7 | F4A | -48.7(7) |  | C27 | C29 | C30 | C32 | -0.1(2) |
| C8 | C6 | C7 | F5A | -172.6(5) |  | C28 | C27 | C29 | C30 | 178.19(15) |
| C8 | C6 | C7 | F6A | 74.2(5) |  | C29 | C27 | C28 | F19 | 137.8(3) |
| C9 | C10 | C11 | C12 | 179.03(14) |  | C29 | C27 | C28 | F20 | -102.9(5) |
| C9 | C10 | C11 | C13 | -0.4(2) |  | C29 | C27 | C28 | F21 | 22.6(3) |
| C10 | C9 | C16 | C14 | -1.4(2) |  | C29 | C27 | C28 | F21A | -5.6(8) |
| C10 | C9 | B1 | C1 | -85.35(17) |  | C29 | C27 | C28 | F20A | -117.5(7) |
| C10 | C9 | B1 | C17 | 152.87(14) |  | C29 | C27 | C28 | F19A | 118.8(7) |
| C10 | C9 | B1 | C25 | 37.1(2) |  | C29 | C30 | C31 | F22 | 112.5(7) |
| C10 | C11 | C12 | F7 | 54.84(19) |  | C29 | C30 | C31 | F23 | -9.8(4) |
| C10 | C11 | C12 | F8 | 174.89(14) |  | C29 | C30 | C31 | F24 | -127.2(3) |
| C10 | C11 | C12 | F9 | -63.0(2) |  | C29 | C30 | C31 | F23A | -21.9(5) |
| C10 | C11 | C13 | C14 | -1.4(2) |  | C29 | C30 | C31 | F22A | 97.0(9) |
| C11 | C13 | C14 | C15 | -177.35(15) |  | C29 | C30 | C31 | F24A | -144.6(7) |
| C11 | C13 | C14 | C16 | 1.9(2) |  | C29 | C30 | C32 | C25 | 0.9(2) |
| C12 | C11 | C13 | C14 | 179.10(14) |  | C31 | C30 | C32 | C25 | 179.25(15) |
| C13 | C11 | C12 | F7 | -125.68(16) |  | C32 | C25 | C26 | C27 | 0.4(2) |
| C13 | C11 | C12 | F8 | -5.6(2) |  | C32 | C25 | B1 | C1 | -40.46(19) |
| C13 | C11 | C12 | F9 | 116.48(17) |  | C32 | C25 | B1 | C9 | -157.48(14) |
| C13 | C14 | C15 | F10 | 125.5(3) |  | C32 | C25 | B1 | C17 | 82.54(16) |
| C13 | C14 | C15 | F11 | 2.0(5) |  | C32 | C30 | C31 | F22 | -65.9(7) |
| C13 | C14 | C15 | F12 | -122.3(4) |  | C32 | C30 | C31 | F23 | 171.8(4) |
| C13 | C14 | C15 | F12A | -18.4(4) |  | C32 | C30 | C31 | F24 | 54.5(3) |
| C13 | C14 | C15 | F11A | 95.1(5) |  | C32 | C30 | C31 | F23A | 159.7(5) |
| C13 | C14 | C15 | F10A | -135.6(6) |  | C32 | C30 | C31 | F22A | -81.4(9) |
| C13 | C14 | C16 | C9 | -0.5(2) |  | C32 | C30 | C31 | F24A | 37.0(7) |
| C15 | C14 | C16 | C9 | 178.78(15) |  | B1 | C1 | C2 | C3 | -172.44(15) |
| C16 | C9 | C10 | C11 | 1.8(2) |  | B1 | C1 | C8 | C6 | 173.35(15) |
| C16 | C9 | B1 | C1 | 87.61(16) |  | B1 | C9 | C10 | C11 | 175.13(14) |
| C16 | C9 | B1 | C17 | -34.16(19) |  | B1 | C9 | C16 | C14 | -174.82(14) |
| C16 | C9 | B1 | C25 | -149.97(14) |  | B1 | C17 | C18 | C19 | -175.22(14) |
| C16 | C14 | C15 | F10 | -53.8(3) |  | B1 | C17 | C24 | C22 | 175.82(14) |
| C16 | C14 | C15 | F11 | -177.2(5) |  | B1 | C25 | C26 | C27 | 172.63(14) |
| C16 | C14 | C15 | F12 | 58.5(5) |  | B1 | C25 | C32 | C30 | -173.37(14) |
| C16 | C14 | C15 | F12A | 162.4(4) |  | N1B | C1B | C2B | C3B | -1.7(3) |
| C16 | C14 | C15 | F11A | -84.2(5) |  | N1B | C5B | C6B | C7B | -34.1(2) |
| C16 | C14 | C15 | F10A | 45.1(6) |  | N2B | C7B | C8B | C9B | 0.9(4) |
| C17 | C18 | C19 | C20 | 178.65(14) |  | C1B | N1B | C5B | C4B | -0.1(3) |
| C17 | C18 | C19 | C21 | -0.7(2) |  | C1B | N1B | C5B | C6B | -177.48(16) |
| C18 | C17 | C24 | C22 | 1.6(2) |  | C1B | C2B | C3B | C4B | 0.8(3) |
| C18 | C17 | B1 | C1 | -158.97(13) |  | C2B | C3B | C4B | C5B | 0.4(3) |
| C18 | C17 | B1 | C9 | -43.35(18) |  | C3B | C4B | C5B | N1B | -0.8(3) |
| C18 | C17 | B1 | C25 | 78.48(16) |  | C3B | C4B | C5B | C6B | 176.40(17) |
| C18 | C19 | C20 | F13 | 64.4(2) |  | C4B | C5B | C6B | C7B | 148.67(18) |
| C18 | C19 | C20 | F14 | -175.05(17) |  | C5B | N1B | C1B | C2B | 1.4(3) |
| C18 | C19 | C20 | F15 | -53.2(2) |  | C5B | C6B | C7B | N2B | 43.7(2) |
| C18 | C19 | C21 | C22 | 1.3(2) |  | C5B | C6B | C7B | C8B | -138.0(2) |
| C19 | C21 | C22 | C23 | 177.39(15) |  | C6B | C7B | C8B | C9B | -177.3(2) |
| C19 | C21 | C22 | C24 | -0.5(2) |  | C7B | N2B | C11B | C10B | -0.6(4) |
| C20 | C19 | C21 | C22 | -178.04(15) |  | C7B | C8B | C9B | C10B | -0.3(5) |
| C21 | C19 | C20 | F13 | -116.32(18) |  | C8B | C9B | C10B | C11B | -0.7(5) |
| C21 | C19 | C20 | F14 | 4.3(2) |  | C9B | C10B | C11B | N2B | 1.2(4) |
| C21 | C19 | C20 | F15 | 126.14(17) |  | C11B | N2B | C7B | C6B | 177.75(19) |
| C21 | C22 | C23 | F16 | 155.4(5) |  | C11B | N2B | C7B | C8B | -0.5(3) |

Table 7 Hydrogen Atom Coordinates (Å×104) and Isotropic Displacement Parameters (Å2×103) for c231122\_1\_1.

| Atom | *x* | *y* | *z* | U(eq) |
| --- | --- | --- | --- | --- |
| H2 | 7047.73 | 5803.57 | 3333.91 | 32 |
| H5 | 6182.38 | 8425.69 | 3908.48 | 40 |
| H8 | 7678.1 | 7615.23 | 5120.36 | 32 |
| H10 | 8353.87 | 5790.65 | 2712.76 | 28 |
| H13 | 9613.24 | 7992.56 | 2529.16 | 30 |
| H16 | 8865.44 | 7363.25 | 4697.59 | 28 |
| H18 | 9129.87 | 5373.5 | 5013.1 | 26 |
| H21 | 8837.45 | 5268.99 | 7432.39 | 30 |
| H24 | 7499.12 | 5991.97 | 5882.29 | 28 |
| H26 | 8742.74 | 4359.64 | 3579.73 | 28 |
| H29 | 7701.72 | 1822.2 | 3639.63 | 35 |
| H32 | 7091.02 | 4525.8 | 4505.5 | 30 |
| H1B | 2526(7) | 7103(15) | 3434(13) | 38 |
| H1BA | 1751.28 | 6260.14 | 4094.72 | 40 |
| H2B | 1024.27 | 7213.19 | 4740.91 | 41 |
| H3B | 1077.41 | 9010.86 | 4692.45 | 45 |
| H4B | 1838.92 | 9798.23 | 3973.4 | 43 |
| H6BA | 2565.58 | 8984.17 | 2512.96 | 45 |
| H6BB | 2809.13 | 9622.54 | 3267.84 | 45 |
| H8B | 3852.17 | 9536.93 | 3143.47 | 69 |
| H9B | 4713.36 | 8538.59 | 3042.99 | 91 |
| H10B | 4629.99 | 6766.93 | 2895.47 | 79 |
| H11B | 3700.28 | 6023.68 | 2880.83 | 61 |

Table 8 Atomic Occupancy for c231122\_1\_1.

| Atom | *Occupancy* |  | Atom | *Occupancy* |  | Atom | *Occupancy* |
| --- | --- | --- | --- | --- | --- | --- | --- |
| F4 | 0.581(10) |  | F5 | 0.581(10) |  | F6 | 0.581(10) |
| F10 | 0.581(10) |  | F11 | 0.581(10) |  | F12 | 0.581(10) |
| F16 | 0.581(10) |  | F17 | 0.581(10) |  | F18 | 0.581(10) |
| F19 | 0.581(10) |  | F20 | 0.581(10) |  | F21 | 0.581(10) |
| F22 | 0.581(10) |  | F23 | 0.581(10) |  | F24 | 0.581(10) |
| F21A | 0.419(10) |  | F20A | 0.419(10) |  | F19A | 0.419(10) |
| F4A | 0.419(10) |  | F5A | 0.419(10) |  | F6A | 0.419(10) |
| F23A | 0.419(10) |  | F22A | 0.419(10) |  | F24A | 0.419(10) |
| F18A | 0.419(10) |  | F17A | 0.419(10) |  | F16A | 0.419(10) |
| F12A | 0.419(10) |  | F11A | 0.419(10) |  | F10A | 0.419(10) |

Table 9 Solvent masks information for c231122\_1\_1.

| Number | X | Y | Z | Volume | Electron count | Content |
| --- | --- | --- | --- | --- | --- | --- |
| 1 | 0.500 | -0.777 | -0.607 | 919.0 | 304.2 | ? |

Experimental

Single crystals of C43H23BF24N2
[c231122\_1\_1]
were
[].
A suitable crystal was selected and
[]
on a
XtaLAB Synergy, Dualflex, Pilatus 200K
diffractometer. The crystal was kept at 100.0(1) K during data collection.
Using Olex2 [1], the structure was solved with the
SHELXT
[2] structure solution program using
Intrinsic Phasing
and refined with the
SHELXL
[3] refinement package using
Least Squares
minimisation.

1. Dolomanov, O.V., Bourhis, L.J., Gildea, R.J, Howard, J.A.K. & Puschmann, H.
   (2009), J. Appl. Cryst. 42, 339-341.
2. Sheldrick, G.M. (2015). Acta Cryst. A71, 3-8.
3. Sheldrick, G.M. (2015). Acta Cryst. C71, 3-8.

Crystal structure determination of
[c231122\_1\_1]

**Crystal Data**
for C43H23BF24N2 (*M*=1034.44 g/mol):
monoclinic, space group P21/c (no. 14),
*a* = 22.5581(2) Å, *b* = 13.06430(10) Å, *c* = 16.51850(10) Å, *β* = 90.2900(10)°,
*V*= 4868.03(6) Å3,
*Z* = 4,
*T* = 100.0(1) K,
μ(Cu Kα) = 1.333 mm-1,
*Dcalc* = 1.411 g/cm3,
72349 reflections measured (7.82° ≤ 2Θ ≤ 159.404°),
10464 unique (*R*int = 0.0462, Rsigma = 0.0294) which were used in all calculations.
The final *R*1 was 0.0479
(I > 2σ(I)) and *wR*2 was 0.1250 (all data).

Refinement model description

Number of restraints - 844,
number of constraints - unknown.

Details:

```
1. Fixed Uiso
```

This report has been created with Olex2, compiled on
2022.04.07 svn.rca3783a0 for OlexSys. Please
let us know
if there are any errors or if you would like to have additional features.
