# Supplementary material for: London Dispersion versus Intramolecular Hydrogen Bond in Bis‐Pyridines: How Accurate Is DFT for Competing Noncovalent Interactions in the Condensed Phase?
Source: Chemistry. 2025 Oct 23;31(66):e02745. doi: 10.1002/chem.202502745 (PMC12648470; doi:10.1002/chem.202502745)

checkCIF/PLATON report

Structure factors have been supplied for datablock(s) c231122_1_1

THIS REPORT IS FOR GUIDANCE ONLY. IF USED AS PART OF A REVIEW PROCEDURE FOR PUBLICATION, IT SHOULD NOT REPLACE THE EXPERTISE OF AN EXPERIENCED CRYSTALLOGRAPHIC REFEREE.

No syntax errors found. CIF dictionary Interpreting this report

Datablock: c231122_1_1

Bond precision:	C-C = 0.0024 A		Wavelength=1.54184
Cell:	a=22.5581(2)	b=13.0643(1)	c=16.5185(1)
	alpha=90	beta=90.290(1)	gamma=90
Temperature:	100 K		

	Calculated	Reported
Volume	4868.04(6)	4868.03(6)
Space group	P 21/c	P 1 21/c 1
Hall group	-P 2ybc	-P 2ybc
Moiety formula	C32 H12 B F24, C11 H11 N2 [+ solvent]	C32 H12 B F24, C11 H11 N2
Sum formula	C43 H23 B F24 N2 [+ solvent]	C43 H23 B F24 N2
Mr	1034.44	1034.44
Dx,g cm-3	1.411	1.411
Z	4	4
Mu (mm-1)	1.333	1.333
F000	2064.0	2064.0
F000'	2074.13	
h,k,lmax	28,16,21	28,16,21
Nref	10604	10464
Tmin,Tmax	0.735,0.779	0.390,1.000
Tmin'	0.635	

Correction method= # Reported T Limits: Tmin=0.390 Tmax=1.000
AbsCorr = GAUSSIAN

Data completeness= 0.987 Theta(max)= 79.702

R(reflections)= 0.0479(9330)   wR2(reflections)= 0.1250(10464)

S = 1.040   Npar= 770

The following ALERTS were generated. Each ALERT has the format
test-name_ALERT_alert-type_alert-level.
Click on the hyperlinks for more details of the test.

Alert level C

```
PLAT213_ALERT_2_C Atom F11            has ADP max/min Ratio .....        3.2 prolat
PLAT213_ALERT_2_C Atom F17            has ADP max/min Ratio .....        3.2 prolat
PLAT213_ALERT_2_C Atom F16A           has ADP max/min Ratio .....        3.5 prolat
PLAT213_ALERT_2_C Atom F19A           has ADP max/min Ratio .....        3.3 prolat
PLAT213_ALERT_2_C Atom F21A           has ADP max/min Ratio .....        3.6 prolat
PLAT213_ALERT_2_C Atom F24A           has ADP max/min Ratio .....        3.2 prolat
PLAT906_ALERT_3_C Large K Value in the Analysis of Variance ......      3.860 Check
PLAT911_ALERT_3_C Missing FCF Refl Between Thmin & STh/L=    0.600          4 Report
PLAT921_ALERT_1_C R1  in the CIF and FCF Differ by ..............    -0.0012 Check
PLAT922_ALERT_1_C wR2 in the CIF and FCF Differ by ..............    -0.0028 Check
PLAT923_ALERT_1_C S    Values in the CIF and FCF Differ by .......     -0.024 Check
```

Alert level G

```
PLAT002_ALERT_2_G Number of Distance or Angle Restraints on AtSite         37 Note
PLAT003_ALERT_2_G Number of Uiso or Uij Restrained non-H Atoms ...         47 Report
PLAT142_ALERT_4_G s.u. on b - Axis Small or Missing .............    0.00010 Ang.
PLAT143_ALERT_4_G s.u. on c - Axis Small or Missing .............    0.00010 Ang.
PLAT172_ALERT_4_G The CIF-Embedded .res File Contains DFIX Records          1 Report
PLAT176_ALERT_4_G The CIF-Embedded .res File Contains SADI Records         10 Report
PLAT178_ALERT_4_G The CIF-Embedded .res File Contains SIMU Records          1 Report
PLAT187_ALERT_4_G The CIF-Embedded .res File Contains RIGU Records          5 Report
PLAT230_ALERT_2_G Hirshfeld Test Diff for    F19      --C28      .        7.0 s.u.
PLAT242_ALERT_2_G Low    'MainMol' Ueq as Compared to Neighbors of         C4 Check
PLAT242_ALERT_2_G Low    'MainMol' Ueq as Compared to Neighbors of         C7 Check
PLAT242_ALERT_2_G Low    'MainMol' Ueq as Compared to Neighbors of        C12 Check
PLAT242_ALERT_2_G Low    'MainMol' Ueq as Compared to Neighbors of        C15 Check
PLAT242_ALERT_2_G Low    'MainMol' Ueq as Compared to Neighbors of        C20 Check
PLAT242_ALERT_2_G Low    'MainMol' Ueq as Compared to Neighbors of        C23 Check
PLAT242_ALERT_2_G Low    'MainMol' Ueq as Compared to Neighbors of        C28 Check
PLAT242_ALERT_2_G Low    'MainMol' Ueq as Compared to Neighbors of        C31 Check
PLAT301_ALERT_3_G Main Residue  Disorder .............(Resd  1  )        26% Note
PLAT434_ALERT_2_G Short Inter HL..HL Contact F3       ..F4A      .       2.69 Ang.
                                            x,3/2-y,-1/2+z  =      4_575 Check
PLAT434_ALERT_2_G Short Inter HL..HL Contact F8       ..F10A     .       2.81 Ang.
                                            x,3/2-y,-1/2+z  =      4_575 Check
PLAT434_ALERT_2_G Short Inter HL..HL Contact F9       ..F11      .       2.73 Ang.
                                          2-x,-1/2+y,1/2-z  =      2_745 Check
PLAT434_ALERT_2_G Short Inter HL..HL Contact F9       ..F12A     .       2.83 Ang.
                                          2-x,-1/2+y,1/2-z  =      2_745 Check
PLAT606_ALERT_4_G Solvent Accessible VOID(S) in Structure .......          ! Info
PLAT720_ALERT_4_G Number of Unusual/Non-Standard Labels .........          3 Note
PLAT860_ALERT_3_G Number of Least-Squares Restraints ............        844 Note
PLAT910_ALERT_3_G Missing # of FCF Reflection(s) Below Theta(Min).          1 Note
PLAT912_ALERT_4_G Missing # of FCF Reflections Above STh/L=  0.600        135 Note
```

```
PLAT913_ALERT_3_G Missing # of Very Strong Reflections in FCF ....          1 Note
PLAT978_ALERT_2_G Number C-C Bonds with Positive Residual Density.          4 Info
```

```
  0 ALERT level A = Most likely a serious problem - resolve or explain
  0 ALERT level B = A potentially serious problem, consider carefully
 11 ALERT level C = Check. Ensure it is not caused by an omission or oversight
 29 ALERT level G = General information/check it is not something unexpected

  3 ALERT type 1 CIF construction/syntax error, inconsistent or missing data
 22 ALERT type 2 Indicator that the structure model may be wrong or deficient
  6 ALERT type 3 Indicator that the structure quality may be low
  9 ALERT type 4 Improvement, methodology, query or suggestion
  0 ALERT type 5 Informative message, check
```

It is advisable to attempt to resolve as many as possible of the alerts in all categories. Often the minor alerts point to easily fixed oversights, errors and omissions in your CIF or refinement strategy, so attention to these fine details can be worthwhile. In order to resolve some of the more serious problems it may be necessary to carry out additional measurements or structure refinements. However, the purpose of your study may justify the reported deviations and the more serious of these should normally be commented upon in the discussion or experimental section of a paper or in the "special_details" fields of the CIF. checkCIF was carefully designed to identify outliers and unusual parameters, but every test has its limitations and alerts that are not important in a particular case may appear. Conversely, the absence of alerts does not guarantee there are no aspects of the results needing attention. It is up to the individual to critically assess their own results and, if necessary, seek expert advice.

Publication of your CIF in IUCr journals

A basic structural check has been run on your CIF. These basic checks will be run on all CIFs submitted for publication in IUCr journals (*Acta Crystallographica*, *Journal of Applied Crystallography*, *Journal of Synchrotron Radiation*); however, if you intend to submit to *Acta Crystallographica Section C* or *E* or *IUCrData*, you should make sure that full publication checks are run on the final version of your CIF prior to submission.

Publication of your CIF in other journals

Please refer to the *Notes for Authors* of the relevant journal for any special instructions relating to CIF submission.

PLATON version of 18/11/2022; check.def file version of 18/11/2022

Datablock c231122_1_1 - ellipsoid plot

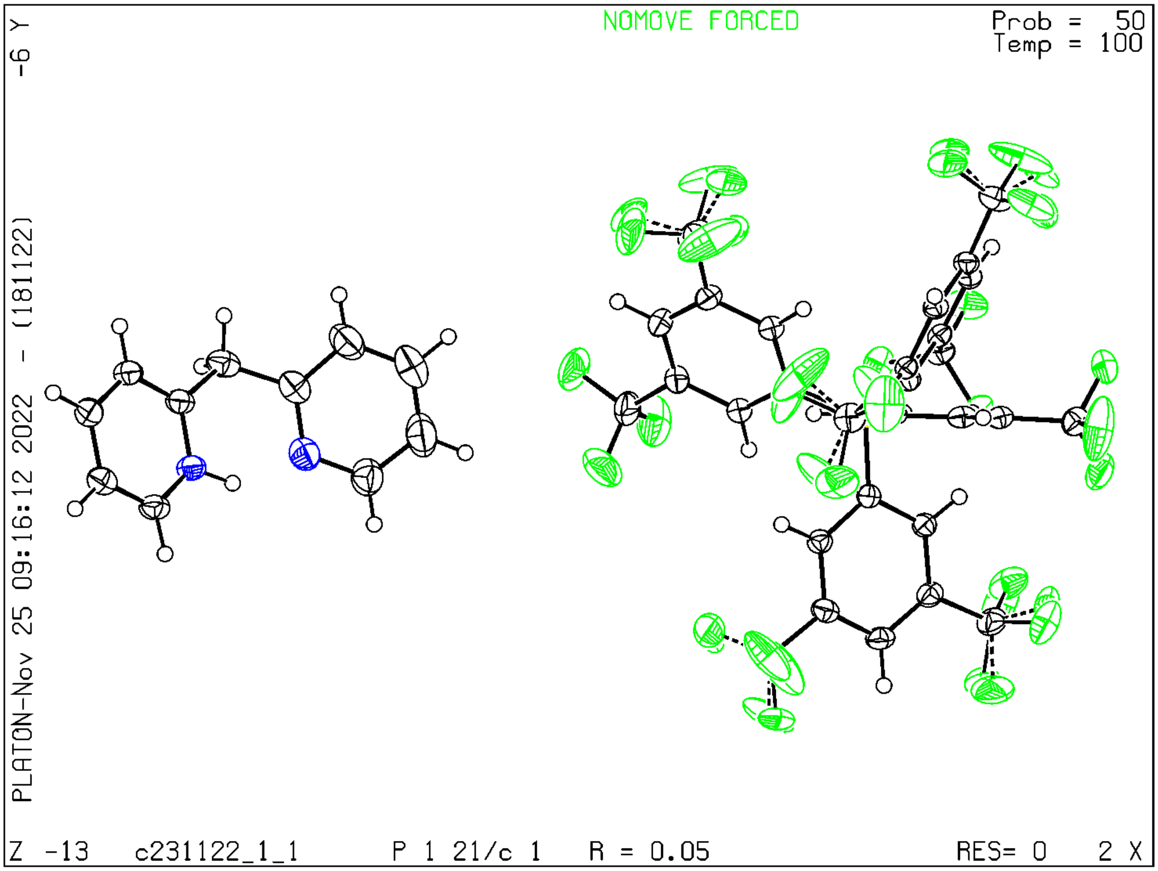

Supplement: Supplementary file 1 — Supporting Information [file CHEM-31-e02745-s002.zip › Crystal_structures/5b/checkcif.pdf]
