# Supplementary material for: London Dispersion versus Intramolecular Hydrogen Bond in Bis‐Pyridines: How Accurate Is DFT for Competing Noncovalent Interactions in the Condensed Phase?
Source: Chemistry. 2025 Oct 23;31(66):e02745. doi: 10.1002/chem.202502745 (PMC12648470; doi:10.1002/chem.202502745)
Supplement: Supplementary file 1 — Supporting Information [file CHEM-31-e02745-s002.zip › Crystal_structures/7b/c040620_1_2_tables.html]

c040620\_1\_2


# c040620\_1\_2

Table 1 Crystal data and structure refinement for c040620\_1\_2.

| Identification code | c040620\_1\_2 |
| Empirical formula | C51H39BF24N2 |
| Formula weight | 1146.65 |
| Temperature/K | 100.0(1) |
| Crystal system | triclinic |
| Space group | P-1 |
| a/Å | 12.9518(7) |
| b/Å | 13.0314(7) |
| c/Å | 17.2798(10) |
| α/° | 97.8190(10) |
| β/° | 107.3230(10) |
| γ/° | 111.3320(10) |
| Volume/Å3 | 2493.6(2) |
| Z | 2 |
| ρcalcg/cm3 | 1.527 |
| μ/mm‑1 | 0.152 |
| F(000) | 1160.0 |
| Crystal size/mm3 | 0.28 × 0.2 × 0.17 |
| Radiation | MoKα (λ = 0.71073) |
| 2Θ range for data collection/° | 3.49 to 61.122 |
| Index ranges | -18 ≤ h ≤ 18, -18 ≤ k ≤ 18, -24 ≤ l ≤ 24 |
| Reflections collected | 59674 |
| Independent reflections | 15264 [Rint = 0.0264, Rsigma = 0.0239] |
| Data/restraints/parameters | 15264/447/804 |
| Goodness-of-fit on F2 | 1.035 |
| Final R indexes [I>=2σ (I)] | R1 = 0.0451, wR2 = 0.1145 |
| Final R indexes [all data] | R1 = 0.0598, wR2 = 0.1238 |
| Largest diff. peak/hole / e Å-3 | 0.62/-0.34 |

Table 2 Fractional Atomic Coordinates (×104) and Equivalent Isotropic Displacement Parameters (Å2×103) for c040620\_1\_2. Ueq is defined as 1/3 of of the trace of the orthogonalised UIJ tensor.

| Atom | *x* | *y* | *z* | U(eq) |
| --- | --- | --- | --- | --- |
| F1 | 2009.2(8) | 3718.0(8) | 4322.8(7) | 41.1(2) |
| F2 | 3666.7(9) | 3630.0(8) | 4897.4(6) | 32.68(19) |
| F3 | 3463.7(11) | 4466.9(9) | 3916.5(6) | 45.8(3) |
| F4A | 3871(2) | 8593.0(18) | 4753.5(14) | 49.6(7) |
| F5A | 5717(3) | 8885(4) | 5296(3) | 53.3(10) |
| F6A | 4913(3) | 9471(3) | 6052.0(16) | 33.4(5) |
| F7 | 8771.3(10) | 9210.9(16) | 9259.5(7) | 49.7(4) |
| F8 | 8841.9(9) | 10460.3(9) | 10229.7(8) | 38.6(3) |
| F9 | 8340.6(10) | 8739.5(11) | 10304.6(8) | 40.4(3) |
| F10A | 5045(4) | 11088(3) | 8342(2) | 74.8(10) |
| F11A | 5479(2) | 11321(2) | 9653.6(18) | 50.7(6) |
| F12A | 3769(2) | 10124(2) | 8869.2(18) | 38.9(5) |
| F13 | 2161(10) | 6452(12) | 9891(8) | 50(3) |
| F14 | 1275(6) | 7383(5) | 9350(6) | 36.7(15) |
| F15 | 301(11) | 5676(11) | 9284(12) | 53.5(10) |
| F16 | -1582.2(7) | 4130.4(9) | 6264.0(6) | 36.0(2) |
| F17 | -536.5(9) | 3438.9(9) | 5805.7(7) | 51.9(3) |
| F18 | -600.0(8) | 4988.3(10) | 5558.6(6) | 42.0(3) |
| F19 | 3325.3(9) | 3684.6(8) | 9587.7(6) | 31.9(2) |
| F20 | 3061.3(10) | 2204.3(8) | 8696.5(7) | 43.1(2) |
| F21 | 4671.6(10) | 3056.1(10) | 9808.1(7) | 45.3(3) |
| F22 | 7587.5(9) | 4108.5(11) | 8476.0(7) | 45.8(3) |
| F23 | 6590.6(10) | 3757.7(10) | 7160.7(7) | 44.6(3) |
| F24 | 7729.8(8) | 5484.1(9) | 7899.6(8) | 43.4(3) |
| C1 | 4035.5(10) | 6403.8(10) | 6688.7(7) | 14.11(19) |
| C2 | 3672.4(10) | 5406.1(10) | 6056.6(7) | 16.2(2) |
| C3 | 3632.4(11) | 5430.5(11) | 5243.4(7) | 18.2(2) |
| C4 | 3964.6(11) | 6454.2(11) | 5025.6(8) | 19.6(2) |
| C5 | 4321.5(11) | 7452.8(11) | 5639.4(8) | 18.9(2) |
| C6 | 4352.0(10) | 7427.4(10) | 6448.9(8) | 16.9(2) |
| C7 | 3196.7(13) | 4323.8(12) | 4596.5(8) | 24.9(3) |
| C8 | 4702.2(13) | 8574.0(12) | 5421.6(9) | 26.0(3) |
| C9 | 4960.9(10) | 7638.7(10) | 8288.1(7) | 14.5(2) |
| C10 | 6159.3(10) | 7946.9(10) | 8775.8(7) | 17.0(2) |
| C11 | 6929.8(10) | 9064.9(11) | 9275.8(8) | 20.4(2) |
| C12 | 6538.3(11) | 9917.9(11) | 9322.5(8) | 23.6(3) |
| C13 | 5355.2(11) | 9633.5(11) | 8849.5(8) | 22.0(2) |
| C14 | 4586.9(10) | 8523.9(10) | 8343.7(8) | 17.9(2) |
| C15 | 8211.3(19) | 9365.1(17) | 9767.3(13) | 22.2(4) |
| C16A | 4896(4) | 10530(4) | 8914(3) | 28.3(8) |
| C17 | 2694.4(10) | 6036.7(9) | 7638.9(7) | 13.36(19) |
| C18 | 2522.2(10) | 6329.2(10) | 8391.4(7) | 15.7(2) |
| C19 | 1388.0(10) | 6019.2(10) | 8416.6(7) | 16.6(2) |
| C20 | 365.9(10) | 5401.1(10) | 7690.0(8) | 17.5(2) |
| C21 | 517.0(10) | 5098.6(10) | 6946.2(7) | 16.1(2) |
| C22 | 1656.4(10) | 5409.9(9) | 6918.9(7) | 14.6(2) |
| C23 | 1236.6(12) | 6354.9(12) | 9226.7(8) | 23.9(3) |
| C24 | -546.5(11) | 4420.3(13) | 6146.9(8) | 25.8(3) |
| C25 | 4496.4(10) | 5411.0(10) | 7939.9(7) | 14.30(19) |
| C26 | 4011.0(11) | 4719.4(10) | 8413.0(7) | 17.0(2) |
| C27 | 4478.8(11) | 3991.5(10) | 8734.0(8) | 18.6(2) |
| C28 | 5453.1(11) | 3909.3(11) | 8595.3(8) | 19.4(2) |
| C29 | 5935.9(11) | 4571.5(11) | 8116.5(8) | 17.9(2) |
| C30 | 5460.7(10) | 5294.1(10) | 7789.5(7) | 16.7(2) |
| C31 | 3893.0(13) | 3243.3(12) | 9208.4(9) | 25.5(3) |
| C32 | 6956.9(12) | 4480.2(13) | 7921.8(9) | 25.3(3) |
| B1 | 4044.2(11) | 6365.4(10) | 7637.4(8) | 13.2(2) |
| N1B | 217.1(10) | -175.2(10) | 2233.0(7) | 21.6(2) |
| N2B | -1464.8(9) | -20.2(9) | 3151.7(7) | 20.0(2) |
| C1B | 992.6(13) | -1652.9(12) | 2181.9(9) | 27.4(3) |
| C2B | 1746(2) | -1810.2(19) | 2984.7(15) | 58.8(6) |
| C3B | -312.0(16) | -2491.3(14) | 1921.6(14) | 42.2(4) |
| C4B | 1408(2) | -1899.4(17) | 1463.9(15) | 54.3(5) |
| C5B | 1176.9(12) | -412.2(12) | 2354.2(8) | 22.5(2) |
| C6B | 2283.0(13) | 516.4(13) | 2618.7(9) | 27.8(3) |
| C7B | 2372.7(13) | 1623.4(13) | 2742.3(9) | 30.2(3) |
| C8B | 1348.8(13) | 1810.1(12) | 2585.4(9) | 26.9(3) |
| C9B | 252.7(12) | 886.2(11) | 2324.4(8) | 22.6(2) |
| C10B | -912.8(14) | 973.2(13) | 2150.8(10) | 28.8(3) |
| C11B | -1277.7(11) | 942.8(11) | 2906.9(8) | 21.6(2) |
| C12B | -1431.7(13) | 1853.0(12) | 3289.1(10) | 30.4(3) |
| C13B | -1818.8(15) | 1744.5(15) | 3953.5(10) | 38.5(4) |
| C14B | -2024.0(15) | 758.2(16) | 4206.2(10) | 36.7(4) |
| C15B | -1835.8(11) | -122.0(12) | 3795.0(8) | 24.3(3) |
| C16B | -2123.3(13) | -1255.2(14) | 4029.6(10) | 32.3(3) |
| C17B | -1571.5(18) | -1955.6(16) | 3660.3(14) | 45.8(4) |
| C18B | -1661.8(16) | -1042.4(18) | 4992.2(11) | 46.1(5) |
| C19B | -3491.1(15) | -1941.3(16) | 3647.8(11) | 40.3(4) |
| F7A | 8481(5) | 8278(5) | 9511(4) | 38.6(3) |
| F9A | 8599(6) | 9532(9) | 10517(4) | 49.7(4) |
| F8A | 8974(6) | 10019(7) | 9508(5) | 46(2) |
| C15A | 8213(12) | 9155(10) | 9673(8) | 22.2(4) |
| F4 | 4571(9) | 9339(9) | 5836(6) | 49(2) |
| F5 | 4168(6) | 8382(5) | 4576(4) | 49.6(7) |
| F6 | 5853(6) | 8919(9) | 5476(6) | 31.5(16) |
| F10 | 4690(8) | 10805(8) | 8001(4) | 60(2) |
| F11 | 5572(6) | 11509(4) | 9324(5) | 41.8(14) |
| C16 | 4907(10) | 10559(9) | 8729(6) | 22.1(19) |
| F12 | 3782(7) | 10172(7) | 8611(5) | 38.9(5) |
| F15A | 803(4) | 7148(3) | 9221(3) | 70.6(11) |
| F14A | 2216(4) | 6773(5) | 9897(3) | 58.2(11) |
| F13A | 420(4) | 5477(4) | 9353(4) | 53.5(10) |

Table 3 Anisotropic Displacement Parameters (Å2×103) for c040620\_1\_2. The Anisotropic displacement factor exponent takes the form: -2π2[h2a\*2U11+2hka\*b\*U12+…].

| Atom | U11 | U22 | U33 | U23 | U13 | U12 |
| --- | --- | --- | --- | --- | --- | --- |
| F1 | 25.8(4) | 32.6(5) | 44.5(6) | -10.1(4) | 2.8(4) | 6.6(4) |
| F2 | 41.6(5) | 31.3(4) | 31.7(4) | 5.8(4) | 15.5(4) | 22.1(4) |
| F3 | 76.4(8) | 38.9(5) | 23.3(4) | 7.2(4) | 28.9(5) | 18.6(5) |
| F4A | 61.6(12) | 28.9(8) | 37.3(9) | 19.4(7) | -8.5(7) | 15.1(6) |
| F5A | 67.8(19) | 44.6(16) | 86(3) | 40.1(16) | 63.8(19) | 28.8(15) |
| F6A | 48.7(13) | 20.5(7) | 31.9(9) | 13.7(6) | 17.1(8) | 12.1(8) |
| F7 | 17.7(5) | 101.4(12) | 22.7(5) | 1.9(6) | 5.6(4) | 24.5(7) |
| F8 | 18.7(5) | 31.4(5) | 42.4(6) | -0.6(5) | -3.7(4) | 1.8(4) |
| F9 | 22.4(5) | 46.1(7) | 41.4(6) | 24.6(6) | -1.1(4) | 8.7(5) |
| F10A | 151(3) | 65.6(17) | 78(2) | 51.4(17) | 75(2) | 83.3(19) |
| F11A | 46.6(10) | 32.6(10) | 53.0(13) | -17.8(9) | 7.0(10) | 15.6(8) |
| F12A | 30.1(5) | 29.2(5) | 58.1(16) | 3.1(10) | 16.5(9) | 16.5(4) |
| F13 | 43(5) | 115(9) | 14(3) | 27(5) | 11(3) | 52(6) |
| F14 | 58(4) | 25.3(18) | 31(2) | 0.5(15) | 27(3) | 16(2) |
| F15 | 57.0(11) | 46.0(17) | 38.3(12) | 2.6(11) | 36.0(10) | -9.7(10) |
| F16 | 11.8(4) | 57.1(6) | 28.3(4) | 12.3(4) | 4.6(3) | 5.2(4) |
| F17 | 31.4(5) | 44.0(6) | 47.3(6) | -23.8(5) | -4.4(4) | 9.2(4) |
| F18 | 24.3(4) | 75.8(7) | 22.2(4) | 21.3(4) | 7.3(3) | 15.9(5) |
| F19 | 47.1(5) | 34.7(4) | 39.3(5) | 22.4(4) | 33.5(4) | 26.2(4) |
| F20 | 62.2(7) | 22.0(4) | 50.9(6) | 14.5(4) | 37.3(5) | 9.9(4) |
| F21 | 58.7(6) | 70.5(7) | 48.3(6) | 48.5(6) | 36.3(5) | 47.7(6) |
| F22 | 44.2(6) | 79.7(8) | 54.4(6) | 44.9(6) | 30.2(5) | 51.4(6) |
| F23 | 48.7(6) | 60.3(7) | 42.0(5) | 8.2(5) | 29.2(5) | 34.1(5) |
| F24 | 29.0(5) | 44.9(6) | 78.0(8) | 31.2(5) | 33.8(5) | 23.0(4) |
| C1 | 10.7(5) | 17.3(5) | 16.3(5) | 7.6(4) | 5.4(4) | 6.8(4) |
| C2 | 15.7(5) | 18.2(5) | 16.8(5) | 7.1(4) | 7.1(4) | 7.7(4) |
| C3 | 16.4(5) | 22.5(5) | 16.0(5) | 5.7(4) | 6.4(4) | 8.3(4) |
| C4 | 15.6(5) | 28.4(6) | 16.3(5) | 11.3(5) | 6.3(4) | 9.2(5) |
| C5 | 15.0(5) | 21.9(5) | 22.5(6) | 13.5(5) | 7.6(4) | 7.7(4) |
| C6 | 14.3(5) | 17.9(5) | 19.1(5) | 8.1(4) | 5.7(4) | 7.0(4) |
| C7 | 29.3(7) | 28.5(6) | 18.1(6) | 6.1(5) | 11.0(5) | 12.2(5) |
| C8 | 27.4(6) | 26.5(6) | 25.5(6) | 15.9(5) | 10.5(5) | 9.5(5) |
| C9 | 12.5(5) | 17.2(5) | 14.8(5) | 6.0(4) | 6.2(4) | 6.0(4) |
| C10 | 14.0(5) | 23.4(5) | 15.6(5) | 8.2(4) | 6.5(4) | 8.3(4) |
| C11 | 12.1(5) | 29.1(6) | 15.0(5) | 6.3(4) | 3.9(4) | 4.5(4) |
| C12 | 17.3(6) | 21.7(6) | 21.2(6) | 0.0(5) | 5.9(5) | 0.2(5) |
| C13 | 18.5(6) | 17.0(5) | 26.7(6) | 1.7(5) | 8.0(5) | 5.4(4) |
| C14 | 12.8(5) | 16.9(5) | 21.2(5) | 3.8(4) | 4.9(4) | 5.2(4) |
| C15 | 14.4(5) | 29.2(10) | 15.8(7) | 3.4(7) | 2.6(5) | 4.9(6) |
| C16A | 31.5(13) | 18.0(11) | 33.0(17) | 1.7(11) | 14.0(12) | 8.2(9) |
| C17 | 13.1(5) | 12.4(4) | 16.6(5) | 5.7(4) | 6.3(4) | 6.5(4) |
| C18 | 14.2(5) | 15.4(5) | 16.4(5) | 3.7(4) | 5.6(4) | 5.4(4) |
| C19 | 17.6(5) | 16.1(5) | 17.6(5) | 4.6(4) | 9.4(4) | 6.6(4) |
| C20 | 13.7(5) | 17.6(5) | 22.1(5) | 5.8(4) | 8.1(4) | 6.4(4) |
| C21 | 12.6(5) | 16.8(5) | 17.6(5) | 5.1(4) | 4.3(4) | 5.9(4) |
| C22 | 14.3(5) | 15.6(5) | 15.5(5) | 4.9(4) | 6.2(4) | 7.5(4) |
| C23 | 21.7(6) | 27.8(6) | 21.2(6) | 3.8(5) | 12.1(5) | 7.2(5) |
| C24 | 14.5(5) | 35.6(7) | 21.1(6) | 3.6(5) | 4.5(5) | 7.5(5) |
| C25 | 15.0(5) | 16.0(5) | 14.0(5) | 5.3(4) | 6.3(4) | 7.9(4) |
| C26 | 19.8(5) | 18.2(5) | 18.8(5) | 7.9(4) | 10.4(4) | 10.9(4) |
| C27 | 24.7(6) | 19.9(5) | 18.2(5) | 9.7(4) | 11.6(5) | 12.7(5) |
| C28 | 25.0(6) | 21.6(5) | 19.4(5) | 9.8(4) | 10.1(5) | 15.5(5) |
| C29 | 18.9(5) | 22.9(5) | 18.7(5) | 8.2(4) | 9.0(4) | 13.7(5) |
| C30 | 17.9(5) | 20.2(5) | 17.4(5) | 8.9(4) | 8.8(4) | 10.9(4) |
| C31 | 36.7(7) | 26.9(6) | 29.0(6) | 17.0(5) | 21.3(6) | 20.2(6) |
| C32 | 26.7(6) | 34.6(7) | 30.7(7) | 17.5(6) | 16.2(5) | 22.7(6) |
| B1 | 12.2(5) | 13.9(5) | 15.3(5) | 6.1(4) | 5.9(4) | 6.3(4) |
| N1B | 22.3(5) | 23.3(5) | 22.7(5) | 10.4(4) | 10.1(4) | 10.9(4) |
| N2B | 17.9(5) | 19.7(5) | 20.5(5) | 3.5(4) | 6.1(4) | 7.5(4) |
| C1B | 32.1(7) | 29.1(7) | 32.1(7) | 14.7(6) | 16.2(6) | 19.5(6) |
| C2B | 62.3(13) | 48.0(11) | 58.9(13) | 29.7(10) | 1.3(10) | 27.9(10) |
| C3B | 37.2(9) | 25.8(7) | 64.9(12) | 7.4(7) | 21.0(8) | 15.1(7) |
| C4B | 86.4(16) | 42.3(10) | 69.7(13) | 23.7(9) | 58.0(13) | 39.3(11) |
| C5B | 26.5(6) | 28.5(6) | 18.8(5) | 10.1(5) | 10.1(5) | 16.0(5) |
| C6B | 22.7(6) | 37.2(7) | 25.6(6) | 8.9(6) | 9.0(5) | 14.9(6) |
| C7B | 25.6(7) | 30.7(7) | 28.1(7) | 5.1(5) | 10.6(6) | 6.1(5) |
| C8B | 33.2(7) | 22.9(6) | 26.7(6) | 7.7(5) | 15.0(6) | 11.0(5) |
| C9B | 28.2(6) | 25.8(6) | 21.8(6) | 12.2(5) | 13.1(5) | 15.0(5) |
| C10B | 32.6(7) | 36.3(7) | 33.3(7) | 19.9(6) | 17.9(6) | 23.3(6) |
| C11B | 18.8(6) | 21.9(6) | 24.4(6) | 5.0(5) | 7.1(5) | 10.4(5) |
| C12B | 28.4(7) | 23.5(6) | 35.5(7) | 1.8(5) | 4.6(6) | 15.1(6) |
| C13B | 40.2(9) | 43.8(9) | 30.1(7) | -6.3(6) | 4.9(7) | 28.8(7) |
| C14B | 36.6(8) | 58.5(10) | 24.0(7) | 6.3(7) | 13.7(6) | 29.7(8) |
| C15B | 16.9(6) | 31.4(7) | 22.4(6) | 5.7(5) | 7.0(5) | 8.8(5) |
| C16B | 25.0(7) | 37.6(8) | 32.0(7) | 15.5(6) | 13.3(6) | 6.7(6) |
| C17B | 51.0(10) | 35.1(8) | 69.0(13) | 31.3(9) | 35.2(10) | 21.1(8) |
| C18B | 31.9(8) | 56.1(11) | 33.2(8) | 21.5(8) | 6.5(7) | 1.8(8) |
| C19B | 27.7(8) | 46.8(9) | 30.1(8) | 8.6(7) | 8.2(6) | 1.5(7) |
| F7A | 18.7(5) | 31.4(5) | 42.4(6) | -0.6(5) | -3.7(4) | 1.8(4) |
| F9A | 17.7(5) | 101.4(12) | 22.7(5) | 1.9(6) | 5.6(4) | 24.5(7) |
| F8A | 13(3) | 47(4) | 63(5) | 34(4) | 1(3) | 0(3) |
| C15A | 14.4(5) | 29.2(10) | 15.8(7) | 3.4(7) | 2.6(5) | 4.9(6) |
| F4 | 73(6) | 40(4) | 76(5) | 38(3) | 53(4) | 40(4) |
| F5 | 61.6(12) | 28.9(8) | 37.3(9) | 19.4(7) | -8.5(7) | 15.1(6) |
| F6 | 17.9(19) | 24(3) | 36(3) | 13.7(19) | 7.9(17) | -8.4(16) |
| F10 | 112(5) | 74(4) | 38(3) | 32(3) | 34(3) | 77(4) |
| F11 | 38(2) | 17.4(18) | 50(3) | -8(2) | 0(2) | 10.6(16) |
| C16 | 24(3) | 18(3) | 24(3) | 2(2) | 11(2) | 10(2) |
| F12 | 30.1(5) | 29.2(5) | 58.1(16) | 3.1(10) | 16.5(9) | 16.5(4) |
| F15A | 128(3) | 94(2) | 58(2) | 34.4(19) | 64(2) | 92(2) |
| F14A | 27.6(12) | 97(3) | 22.3(14) | -12.7(13) | 10.7(9) | 6.3(12) |
| F13A | 57.0(11) | 46.0(17) | 38.3(12) | 2.6(11) | 36.0(10) | -9.7(10) |

Table 4 Bond Lengths for c040620\_1\_2.

| Atom | Atom | Length/Å |  | Atom | Atom | Length/Å |
| --- | --- | --- | --- | --- | --- | --- |
| F1 | C7 | 1.3396(17) |  | C17 | C22 | 1.3968(16) |
| F2 | C7 | 1.3410(17) |  | C17 | B1 | 1.6426(16) |
| F3 | C7 | 1.3379(15) |  | C18 | C19 | 1.3907(16) |
| F4A | C8 | 1.334(2) |  | C19 | C20 | 1.3917(17) |
| F5A | C8 | 1.321(4) |  | C19 | C23 | 1.4996(17) |
| F6A | C8 | 1.368(3) |  | C20 | C21 | 1.3833(16) |
| F7 | C15 | 1.332(2) |  | C21 | C22 | 1.3985(15) |
| F8 | C15 | 1.340(2) |  | C21 | C24 | 1.4951(17) |
| F9 | C15 | 1.329(2) |  | C23 | F15A | 1.343(3) |
| F10A | C16A | 1.323(4) |  | C23 | F14A | 1.303(4) |
| F11A | C16A | 1.329(4) |  | C23 | F13A | 1.342(3) |
| F12A | C16A | 1.332(4) |  | C25 | C26 | 1.4036(16) |
| F13 | C23 | 1.341(8) |  | C25 | C30 | 1.4041(15) |
| F14 | C23 | 1.308(7) |  | C25 | B1 | 1.6395(16) |
| F15 | C23 | 1.255(10) |  | C26 | C27 | 1.3913(16) |
| F16 | C24 | 1.3406(15) |  | C27 | C28 | 1.3903(17) |
| F17 | C24 | 1.3398(18) |  | C27 | C31 | 1.4993(17) |
| F18 | C24 | 1.3363(17) |  | C28 | C29 | 1.3893(17) |
| F19 | C31 | 1.3389(15) |  | C29 | C30 | 1.3944(16) |
| F20 | C31 | 1.3496(18) |  | C29 | C32 | 1.4982(17) |
| F21 | C31 | 1.3325(17) |  | N1B | C5B | 1.3514(17) |
| F22 | C32 | 1.3292(15) |  | N1B | C9B | 1.3528(17) |
| F23 | C32 | 1.3445(18) |  | N2B | C11B | 1.3404(17) |
| F24 | C32 | 1.3412(17) |  | N2B | C15B | 1.3402(17) |
| C1 | C2 | 1.4027(16) |  | C1B | C2B | 1.528(2) |
| C1 | C6 | 1.4045(15) |  | C1B | C3B | 1.524(2) |
| C1 | B1 | 1.6437(17) |  | C1B | C4B | 1.532(2) |
| C2 | C3 | 1.3958(16) |  | C1B | C5B | 1.5184(19) |
| C3 | C4 | 1.3862(17) |  | C5B | C6B | 1.387(2) |
| C3 | C7 | 1.4971(18) |  | C6B | C7B | 1.386(2) |
| C4 | C5 | 1.3881(19) |  | C7B | C8B | 1.389(2) |
| C5 | C6 | 1.3927(17) |  | C8B | C9B | 1.377(2) |
| C5 | C8 | 1.5019(17) |  | C9B | C10B | 1.4986(19) |
| C8 | F4 | 1.240(8) |  | C10B | C11B | 1.5167(19) |
| C8 | F5 | 1.359(6) |  | C11B | C12B | 1.3857(18) |
| C8 | F6 | 1.361(7) |  | C12B | C13B | 1.387(2) |
| C9 | C10 | 1.3986(16) |  | C13B | C14B | 1.373(3) |
| C9 | C14 | 1.4066(16) |  | C14B | C15B | 1.402(2) |
| C9 | B1 | 1.6405(17) |  | C15B | C16B | 1.529(2) |
| C10 | C11 | 1.3968(18) |  | C16B | C17B | 1.529(3) |
| C11 | C12 | 1.382(2) |  | C16B | C18B | 1.537(2) |
| C11 | C15 | 1.492(2) |  | C16B | C19B | 1.538(2) |
| C11 | C15A | 1.550(14) |  | F7A | C15A | 1.329(12) |
| C12 | C13 | 1.3827(18) |  | F9A | C15A | 1.345(12) |
| C13 | C14 | 1.3915(17) |  | F8A | C15A | 1.330(13) |
| C13 | C16A | 1.497(5) |  | F10 | C16 | 1.315(9) |
| C13 | C16 | 1.530(11) |  | F11 | C16 | 1.297(10) |
| C17 | C18 | 1.4090(15) |  | C16 | F12 | 1.296(11) |

Table 5 Bond Angles for c040620\_1\_2.

| Atom | Atom | Atom | Angle/˚ |  | Atom | Atom | Atom | Angle/˚ |
| --- | --- | --- | --- | --- | --- | --- | --- | --- |
| C2 | C1 | C6 | 115.63(10) |  | F16 | C24 | C21 | 112.47(11) |
| C2 | C1 | B1 | 121.90(10) |  | F17 | C24 | F16 | 106.40(12) |
| C6 | C1 | B1 | 122.44(10) |  | F17 | C24 | C21 | 111.76(11) |
| C3 | C2 | C1 | 122.13(11) |  | F18 | C24 | F16 | 106.71(11) |
| C2 | C3 | C7 | 118.87(11) |  | F18 | C24 | F17 | 106.55(12) |
| C4 | C3 | C2 | 121.12(11) |  | F18 | C24 | C21 | 112.53(12) |
| C4 | C3 | C7 | 120.00(11) |  | C26 | C25 | C30 | 115.66(10) |
| C3 | C4 | C5 | 117.81(11) |  | C26 | C25 | B1 | 122.76(10) |
| C4 | C5 | C6 | 121.07(11) |  | C30 | C25 | B1 | 121.42(10) |
| C4 | C5 | C8 | 118.75(11) |  | C27 | C26 | C25 | 122.14(11) |
| C6 | C5 | C8 | 120.17(12) |  | C26 | C27 | C31 | 119.59(11) |
| C5 | C6 | C1 | 122.23(11) |  | C28 | C27 | C26 | 121.28(11) |
| F1 | C7 | F2 | 105.43(12) |  | C28 | C27 | C31 | 119.09(11) |
| F1 | C7 | C3 | 112.33(11) |  | C29 | C28 | C27 | 117.60(11) |
| F2 | C7 | C3 | 112.48(11) |  | C28 | C29 | C30 | 121.09(11) |
| F3 | C7 | F1 | 106.97(12) |  | C28 | C29 | C32 | 119.63(11) |
| F3 | C7 | F2 | 106.01(11) |  | C30 | C29 | C32 | 119.25(11) |
| F3 | C7 | C3 | 113.06(11) |  | C29 | C30 | C25 | 122.19(11) |
| F4A | C8 | F6A | 104.11(19) |  | F19 | C31 | F20 | 105.95(12) |
| F4A | C8 | C5 | 113.15(13) |  | F19 | C31 | C27 | 112.94(10) |
| F5A | C8 | F4A | 108.5(2) |  | F20 | C31 | C27 | 111.85(11) |
| F5A | C8 | F6A | 104.0(2) |  | F21 | C31 | F19 | 106.55(11) |
| F5A | C8 | C5 | 113.6(2) |  | F21 | C31 | F20 | 106.55(12) |
| F6A | C8 | C5 | 112.66(18) |  | F21 | C31 | C27 | 112.52(12) |
| F4 | C8 | C5 | 115.4(5) |  | F22 | C32 | F23 | 106.37(12) |
| F4 | C8 | F5 | 112.6(5) |  | F22 | C32 | F24 | 106.76(12) |
| F4 | C8 | F6 | 111.6(6) |  | F22 | C32 | C29 | 113.12(11) |
| F5 | C8 | C5 | 109.2(3) |  | F23 | C32 | C29 | 112.34(12) |
| F5 | C8 | F6 | 98.4(5) |  | F24 | C32 | F23 | 105.17(12) |
| F6 | C8 | C5 | 108.3(5) |  | F24 | C32 | C29 | 112.52(11) |
| C10 | C9 | C14 | 115.55(11) |  | C9 | B1 | C1 | 108.21(9) |
| C10 | C9 | B1 | 123.83(10) |  | C9 | B1 | C17 | 109.37(9) |
| C14 | C9 | B1 | 120.50(10) |  | C17 | B1 | C1 | 110.03(9) |
| C11 | C10 | C9 | 121.81(11) |  | C25 | B1 | C1 | 110.30(9) |
| C10 | C11 | C15 | 120.08(13) |  | C25 | B1 | C9 | 109.68(9) |
| C10 | C11 | C15A | 109.4(4) |  | C25 | B1 | C17 | 109.23(9) |
| C12 | C11 | C10 | 121.49(11) |  | C5B | N1B | C9B | 125.10(12) |
| C12 | C11 | C15 | 118.42(13) |  | C15B | N2B | C11B | 118.61(11) |
| C12 | C11 | C15A | 129.0(5) |  | C2B | C1B | C4B | 110.32(17) |
| C11 | C12 | C13 | 117.81(12) |  | C3B | C1B | C2B | 109.16(15) |
| C12 | C13 | C14 | 120.94(12) |  | C3B | C1B | C4B | 108.47(16) |
| C12 | C13 | C16A | 118.89(19) |  | C5B | C1B | C2B | 108.17(14) |
| C12 | C13 | C16 | 121.1(4) |  | C5B | C1B | C3B | 112.62(12) |
| C14 | C13 | C16A | 120.13(19) |  | C5B | C1B | C4B | 108.10(12) |
| C14 | C13 | C16 | 116.9(4) |  | N1B | C5B | C1B | 119.27(12) |
| C13 | C14 | C9 | 122.40(11) |  | N1B | C5B | C6B | 116.72(12) |
| F7 | C15 | F8 | 105.59(18) |  | C6B | C5B | C1B | 124.00(12) |
| F7 | C15 | C11 | 111.18(15) |  | C7B | C6B | C5B | 120.48(13) |
| F8 | C15 | C11 | 113.76(15) |  | C6B | C7B | C8B | 120.08(13) |
| F9 | C15 | F7 | 107.44(17) |  | C9B | C8B | C7B | 119.24(13) |
| F9 | C15 | F8 | 105.87(16) |  | N1B | C9B | C8B | 118.33(12) |
| F9 | C15 | C11 | 112.51(17) |  | N1B | C9B | C10B | 117.20(12) |
| F10A | C16A | F11A | 105.8(3) |  | C8B | C9B | C10B | 124.46(13) |
| F10A | C16A | F12A | 111.8(4) |  | C9B | C10B | C11B | 111.64(11) |
| F10A | C16A | C13 | 109.6(3) |  | N2B | C11B | C10B | 115.54(11) |
| F11A | C16A | F12A | 102.4(3) |  | N2B | C11B | C12B | 123.68(13) |
| F11A | C16A | C13 | 113.2(3) |  | C12B | C11B | C10B | 120.75(13) |
| F12A | C16A | C13 | 113.7(3) |  | C11B | C12B | C13B | 117.60(14) |
| C18 | C17 | B1 | 120.95(10) |  | C14B | C13B | C12B | 119.33(14) |
| C22 | C17 | C18 | 115.98(10) |  | C13B | C14B | C15B | 119.85(14) |
| C22 | C17 | B1 | 122.99(10) |  | N2B | C15B | C14B | 120.92(14) |
| C19 | C18 | C17 | 122.12(11) |  | N2B | C15B | C16B | 117.98(12) |
| C18 | C19 | C20 | 120.91(11) |  | C14B | C15B | C16B | 120.99(13) |
| C18 | C19 | C23 | 120.76(11) |  | C15B | C16B | C17B | 111.28(12) |
| C20 | C19 | C23 | 118.33(10) |  | C15B | C16B | C18B | 110.74(14) |
| C21 | C20 | C19 | 117.80(10) |  | C15B | C16B | C19B | 107.25(13) |
| C20 | C21 | C22 | 121.41(11) |  | C17B | C16B | C18B | 109.14(16) |
| C20 | C21 | C24 | 120.33(11) |  | C17B | C16B | C19B | 108.36(15) |
| C22 | C21 | C24 | 118.26(11) |  | C18B | C16B | C19B | 110.02(13) |
| C17 | C22 | C21 | 121.77(10) |  | F7A | C15A | C11 | 122.4(9) |
| F13 | C23 | C19 | 111.0(7) |  | F7A | C15A | F9A | 106.7(10) |
| F14 | C23 | F13 | 103.8(7) |  | F7A | C15A | F8A | 105.9(10) |
| F14 | C23 | C19 | 110.5(4) |  | F9A | C15A | C11 | 109.0(8) |
| F15 | C23 | F13 | 107.0(8) |  | F8A | C15A | C11 | 109.6(9) |
| F15 | C23 | F14 | 109.0(8) |  | F8A | C15A | F9A | 101.1(10) |
| F15 | C23 | C19 | 114.9(9) |  | F10 | C16 | C13 | 119.3(8) |
| F15A | C23 | C19 | 111.9(2) |  | F11 | C16 | C13 | 112.6(7) |
| F14A | C23 | C19 | 114.6(3) |  | F11 | C16 | F10 | 108.4(8) |
| F14A | C23 | F15A | 106.3(3) |  | F12 | C16 | C13 | 110.8(8) |
| F14A | C23 | F13A | 107.6(3) |  | F12 | C16 | F10 | 90.6(8) |
| F13A | C23 | C19 | 112.1(3) |  | F12 | C16 | F11 | 113.5(9) |
| F13A | C23 | F15A | 103.6(3) |  |  |  |  |  |

Table 6 Torsion Angles for c040620\_1\_2.

| A | B | C | D | Angle/˚ |  | A | B | C | D | Angle/˚ |
| --- | --- | --- | --- | --- | --- | --- | --- | --- | --- | --- |
| C1 | C2 | C3 | C4 | 0.65(18) |  | C20 | C19 | C23 | F14 | -93.6(4) |
| C1 | C2 | C3 | C7 | -178.03(11) |  | C20 | C19 | C23 | F15 | 30.2(8) |
| C2 | C1 | C6 | C5 | -0.74(16) |  | C20 | C19 | C23 | F15A | -69.0(3) |
| C2 | C1 | B1 | C9 | 158.74(10) |  | C20 | C19 | C23 | F14A | 169.9(3) |
| C2 | C1 | B1 | C17 | -81.82(12) |  | C20 | C19 | C23 | F13A | 46.9(3) |
| C2 | C1 | B1 | C25 | 38.76(14) |  | C20 | C21 | C22 | C17 | 0.17(17) |
| C2 | C3 | C4 | C5 | -0.94(18) |  | C20 | C21 | C24 | F16 | -2.71(18) |
| C2 | C3 | C7 | F1 | 73.88(15) |  | C20 | C21 | C24 | F17 | -122.32(13) |
| C2 | C3 | C7 | F2 | -44.89(16) |  | C20 | C21 | C24 | F18 | 117.84(13) |
| C2 | C3 | C7 | F3 | -164.93(12) |  | C22 | C17 | C18 | C19 | -0.36(16) |
| C3 | C4 | C5 | C6 | 0.41(18) |  | C22 | C17 | B1 | C1 | 25.79(14) |
| C3 | C4 | C5 | C8 | 179.13(11) |  | C22 | C17 | B1 | C9 | 144.53(10) |
| C4 | C3 | C7 | F1 | -104.82(14) |  | C22 | C17 | B1 | C25 | -95.43(12) |
| C4 | C3 | C7 | F2 | 136.41(12) |  | C22 | C21 | C24 | F16 | 177.04(11) |
| C4 | C3 | C7 | F3 | 16.37(18) |  | C22 | C21 | C24 | F17 | 57.43(16) |
| C4 | C5 | C6 | C1 | 0.45(18) |  | C22 | C21 | C24 | F18 | -62.41(16) |
| C4 | C5 | C8 | F4A | 56.0(2) |  | C23 | C19 | C20 | C21 | 179.85(11) |
| C4 | C5 | C8 | F5A | -68.3(3) |  | C24 | C21 | C22 | C17 | -179.58(11) |
| C4 | C5 | C8 | F6A | 173.74(18) |  | C25 | C26 | C27 | C28 | -0.41(19) |
| C4 | C5 | C8 | F4 | 153.6(5) |  | C25 | C26 | C27 | C31 | -177.83(12) |
| C4 | C5 | C8 | F5 | 25.6(4) |  | C26 | C25 | C30 | C29 | -2.42(17) |
| C4 | C5 | C8 | F6 | -80.5(5) |  | C26 | C25 | B1 | C1 | -142.73(11) |
| C6 | C1 | C2 | C3 | 0.20(16) |  | C26 | C25 | B1 | C9 | 98.18(12) |
| C6 | C1 | B1 | C9 | -23.37(14) |  | C26 | C25 | B1 | C17 | -21.67(15) |
| C6 | C1 | B1 | C17 | 96.08(12) |  | C26 | C27 | C28 | C29 | -0.67(19) |
| C6 | C1 | B1 | C25 | -143.35(10) |  | C26 | C27 | C31 | F19 | -27.61(18) |
| C6 | C5 | C8 | F4A | -125.3(2) |  | C26 | C27 | C31 | F20 | 91.82(15) |
| C6 | C5 | C8 | F5A | 110.4(3) |  | C26 | C27 | C31 | F21 | -148.29(12) |
| C6 | C5 | C8 | F6A | -7.5(2) |  | C27 | C28 | C29 | C30 | 0.16(19) |
| C6 | C5 | C8 | F4 | -27.7(6) |  | C27 | C28 | C29 | C32 | -177.70(12) |
| C6 | C5 | C8 | F5 | -155.6(4) |  | C28 | C27 | C31 | F19 | 154.92(12) |
| C6 | C5 | C8 | F6 | 98.2(5) |  | C28 | C27 | C31 | F20 | -85.65(15) |
| C7 | C3 | C4 | C5 | 177.73(11) |  | C28 | C27 | C31 | F21 | 34.24(18) |
| C8 | C5 | C6 | C1 | -178.26(11) |  | C28 | C29 | C30 | C25 | 1.46(19) |
| C9 | C10 | C11 | C12 | 1.03(18) |  | C28 | C29 | C32 | F22 | -24.04(19) |
| C9 | C10 | C11 | C15 | -178.23(13) |  | C28 | C29 | C32 | F23 | 96.41(15) |
| C9 | C10 | C11 | C15A | -175.1(5) |  | C28 | C29 | C32 | F24 | -145.13(13) |
| C10 | C9 | C14 | C13 | 0.01(18) |  | C30 | C25 | C26 | C27 | 1.90(17) |
| C10 | C9 | B1 | C1 | -95.10(12) |  | C30 | C25 | B1 | C1 | 41.88(14) |
| C10 | C9 | B1 | C17 | 145.03(11) |  | C30 | C25 | B1 | C9 | -77.21(13) |
| C10 | C9 | B1 | C25 | 25.26(14) |  | C30 | C25 | B1 | C17 | 162.94(10) |
| C10 | C11 | C12 | C13 | -0.66(19) |  | C30 | C29 | C32 | F22 | 158.06(13) |
| C10 | C11 | C15 | F7 | 62.0(2) |  | C30 | C29 | C32 | F23 | -81.50(16) |
| C10 | C11 | C15 | F8 | -178.92(14) |  | C30 | C29 | C32 | F24 | 36.96(18) |
| C10 | C11 | C15 | F9 | -58.54(19) |  | C31 | C27 | C28 | C29 | 176.76(12) |
| C10 | C11 | C15A | F7A | 2.2(12) |  | C32 | C29 | C30 | C25 | 179.33(12) |
| C10 | C11 | C15A | F9A | -123.2(8) |  | B1 | C1 | C2 | C3 | 178.23(10) |
| C10 | C11 | C15A | F8A | 127.0(8) |  | B1 | C1 | C6 | C5 | -178.76(10) |
| C11 | C12 | C13 | C14 | 0.0(2) |  | B1 | C9 | C10 | C11 | 175.31(10) |
| C11 | C12 | C13 | C16A | 177.7(2) |  | B1 | C9 | C14 | C13 | -176.11(11) |
| C11 | C12 | C13 | C16 | -168.1(5) |  | B1 | C17 | C18 | C19 | -177.24(10) |
| C12 | C11 | C15 | F7 | -117.25(17) |  | B1 | C17 | C22 | C21 | 177.12(10) |
| C12 | C11 | C15 | F8 | 1.8(2) |  | B1 | C25 | C26 | C27 | -173.73(11) |
| C12 | C11 | C15 | F9 | 122.18(16) |  | B1 | C25 | C30 | C29 | 173.28(11) |
| C12 | C11 | C15A | F7A | -173.5(7) |  | N1B | C5B | C6B | C7B | -0.4(2) |
| C12 | C11 | C15A | F9A | 61.1(11) |  | N1B | C9B | C10B | C11B | 82.61(16) |
| C12 | C11 | C15A | F8A | -48.7(11) |  | N2B | C11B | C12B | C13B | -1.0(2) |
| C12 | C13 | C14 | C9 | 0.3(2) |  | N2B | C15B | C16B | C17B | 17.64(19) |
| C12 | C13 | C16A | F10A | 90.2(3) |  | N2B | C15B | C16B | C18B | 139.23(14) |
| C12 | C13 | C16A | F11A | -27.6(4) |  | N2B | C15B | C16B | C19B | -100.71(15) |
| C12 | C13 | C16A | F12A | -143.9(2) |  | C1B | C5B | C6B | C7B | 178.38(13) |
| C12 | C13 | C16 | F10 | 105.0(8) |  | C2B | C1B | C5B | N1B | -123.46(16) |
| C12 | C13 | C16 | F11 | -23.6(9) |  | C2B | C1B | C5B | C6B | 57.79(19) |
| C12 | C13 | C16 | F12 | -151.9(5) |  | C3B | C1B | C5B | N1B | -2.73(19) |
| C14 | C9 | C10 | C11 | -0.67(17) |  | C3B | C1B | C5B | C6B | 178.52(14) |
| C14 | C9 | B1 | C1 | 80.68(12) |  | C4B | C1B | C5B | N1B | 117.08(16) |
| C14 | C9 | B1 | C17 | -39.19(14) |  | C4B | C1B | C5B | C6B | -61.67(19) |
| C14 | C9 | B1 | C25 | -158.95(10) |  | C5B | N1B | C9B | C8B | -2.1(2) |
| C14 | C13 | C16A | F10A | -92.2(4) |  | C5B | N1B | C9B | C10B | 179.26(12) |
| C14 | C13 | C16A | F11A | 150.1(3) |  | C5B | C6B | C7B | C8B | -1.3(2) |
| C14 | C13 | C16A | F12A | 33.8(4) |  | C6B | C7B | C8B | C9B | 1.4(2) |
| C14 | C13 | C16 | F10 | -63.6(10) |  | C7B | C8B | C9B | N1B | 0.2(2) |
| C14 | C13 | C16 | F11 | 167.9(6) |  | C7B | C8B | C9B | C10B | 178.77(13) |
| C14 | C13 | C16 | F12 | 39.5(8) |  | C8B | C9B | C10B | C11B | -95.98(16) |
| C15 | C11 | C12 | C13 | 178.61(14) |  | C9B | N1B | C5B | C1B | -176.70(12) |
| C16A | C13 | C14 | C9 | -177.3(2) |  | C9B | N1B | C5B | C6B | 2.14(19) |
| C17 | C18 | C19 | C20 | -0.06(18) |  | C9B | C10B | C11B | N2B | -61.99(16) |
| C17 | C18 | C19 | C23 | -179.36(11) |  | C9B | C10B | C11B | C12B | 119.73(14) |
| C18 | C17 | C22 | C21 | 0.31(16) |  | C10B | C11B | C12B | C13B | 177.13(13) |
| C18 | C17 | B1 | C1 | -157.56(10) |  | C11B | N2B | C15B | C14B | -0.08(19) |
| C18 | C17 | B1 | C9 | -38.82(13) |  | C11B | N2B | C15B | C16B | 176.10(12) |
| C18 | C17 | B1 | C25 | 81.23(12) |  | C11B | C12B | C13B | C14B | 0.4(2) |
| C18 | C19 | C20 | C21 | 0.54(17) |  | C12B | C13B | C14B | C15B | 0.3(2) |
| C18 | C19 | C23 | F13 | -28.9(7) |  | C13B | C14B | C15B | N2B | -0.5(2) |
| C18 | C19 | C23 | F14 | 85.7(4) |  | C13B | C14B | C15B | C16B | -176.54(14) |
| C18 | C19 | C23 | F15 | -150.5(8) |  | C14B | C15B | C16B | C17B | -166.18(15) |
| C18 | C19 | C23 | F15A | 110.3(3) |  | C14B | C15B | C16B | C18B | -44.59(19) |
| C18 | C19 | C23 | F14A | -10.7(3) |  | C14B | C15B | C16B | C19B | 75.47(17) |
| C18 | C19 | C23 | F13A | -133.8(3) |  | C15B | N2B | C11B | C10B | -177.38(12) |
| C19 | C20 | C21 | C22 | -0.59(17) |  | C15B | N2B | C11B | C12B | 0.85(19) |
| C19 | C20 | C21 | C24 | 179.15(11) |  | C15A | C11 | C12 | C13 | 174.6(6) |
| C20 | C19 | C23 | F13 | 151.8(7) |  | C16 | C13 | C14 | C9 | 168.9(5) |

Table 7 Hydrogen Atom Coordinates (Å×104) and Isotropic Displacement Parameters (Å2×103) for c040620\_1\_2.

| Atom | *x* | *y* | *z* | U(eq) |
| --- | --- | --- | --- | --- |
| H2 | 3446.42 | 4690.53 | 6186.11 | 19 |
| H4 | 3948.53 | 6471.75 | 4474.43 | 23 |
| H6 | 4595.2 | 8126.12 | 6852.54 | 20 |
| H10 | 6457.51 | 7380.34 | 8766.8 | 20 |
| H12 | 7064.61 | 10674.82 | 9668.3 | 28 |
| H14 | 3780.35 | 8358.21 | 8024.36 | 21 |
| H18 | 3202.86 | 6751.65 | 8897.91 | 19 |
| H20 | -409.93 | 5193.74 | 7704.41 | 21 |
| H22 | 1726.28 | 5189.09 | 6396.6 | 17 |
| H26 | 3341.24 | 4749.36 | 8517.41 | 20 |
| H28 | 5776.99 | 3417.95 | 8819.78 | 23 |
| H30 | 5800.62 | 5721.65 | 7453.67 | 20 |
| H1B | -521(10) | -775(11) | 2089(11) | 26 |
| H2BA | 1500.28 | -1609.65 | 3446.37 | 88 |
| H2BB | 2592.65 | -1311.28 | 3132.52 | 88 |
| H2BC | 1627.05 | -2610.66 | 2890.13 | 88 |
| H3BA | -386.01 | -3277.6 | 1806.37 | 63 |
| H3BB | -801.07 | -2384.04 | 1412.58 | 63 |
| H3BC | -588.15 | -2351.58 | 2378.35 | 63 |
| H4BA | 2256.22 | -1389.14 | 1631.71 | 81 |
| H4BB | 936.93 | -1768.77 | 959.16 | 81 |
| H4BC | 1294.96 | -2697.99 | 1341.29 | 81 |
| H6B | 2984.23 | 392.41 | 2715.66 | 33 |
| H7B | 3135.2 | 2255.43 | 2934.71 | 36 |
| H8B | 1404.29 | 2566.7 | 2657.3 | 32 |
| H10A | -1542.86 | 330.11 | 1662.56 | 35 |
| H10B | -839.35 | 1697.15 | 2002.33 | 35 |
| H12B | -1277.8 | 2527.02 | 3102.87 | 37 |
| H13B | -1940.7 | 2346.26 | 4230.89 | 46 |
| H14B | -2292.87 | 672.8 | 4658.61 | 44 |
| H17A | -1908.05 | -2142.31 | 3043.67 | 69 |
| H17B | -1750.02 | -2665.12 | 3834.37 | 69 |
| H17C | -702.76 | -1508.05 | 3866.38 | 69 |
| H18A | -798.4 | -557.02 | 5234.35 | 69 |
| H18B | -1822.12 | -1776.22 | 5129.49 | 69 |
| H18C | -2070.67 | -658.27 | 5226.58 | 69 |
| H19A | -3860.53 | -1501.62 | 3874.21 | 60 |
| H19B | -3708.94 | -2672.16 | 3792.41 | 60 |
| H19C | -3775.54 | -2087.47 | 3033.28 | 60 |

Table 8 Atomic Occupancy for c040620\_1\_2.

| Atom | *Occupancy* |  | Atom | *Occupancy* |  | Atom | *Occupancy* |
| --- | --- | --- | --- | --- | --- | --- | --- |
| F4A | 0.735(6) |  | F5A | 0.735(6) |  | F6A | 0.735(6) |
| F7 | 0.8563(16) |  | F8 | 0.8563(16) |  | F9 | 0.8563(16) |
| F10A | 0.735(6) |  | F11A | 0.735(6) |  | F12A | 0.735(6) |
| F13 | 0.265(6) |  | F14 | 0.265(6) |  | F15 | 0.265(6) |
| C15 | 0.8563(16) |  | C16A | 0.735(6) |  | F7A | 0.1437(16) |
| F9A | 0.1437(16) |  | F8A | 0.1437(16) |  | C15A | 0.1437(16) |
| F4 | 0.265(6) |  | F5 | 0.265(6) |  | F6 | 0.265(6) |
| F10 | 0.265(6) |  | F11 | 0.265(6) |  | C16 | 0.265(6) |
| F12 | 0.265(6) |  | F15A | 0.735(6) |  | F14A | 0.735(6) |
| F13A | 0.735(6) |  |  |  |  |  |

Experimental

Single crystals of C51H39BF24N2
[c040620\_1\_2]
were
[].
A suitable crystal was selected and
[]
on a
Bruker APEX-II Duo (Mo)
diffractometer. The crystal was kept at 100.0(1) K during data collection.
Using Olex2 [1], the structure was solved with the
SHELXT
[2] structure solution program using
Intrinsic Phasing
and refined with the
SHELXL
[3] refinement package using
Least Squares
minimisation.

1. Dolomanov, O.V., Bourhis, L.J., Gildea, R.J, Howard, J.A.K. & Puschmann, H.
   (2009), J. Appl. Cryst. 42, 339-341.
2. Sheldrick, G.M. (2015). Acta Cryst. A71, 3-8.
3. Sheldrick, G.M. (2015). Acta Cryst. C71, 3-8.

Crystal structure determination of
[c040620\_1\_2]

**Crystal Data**
for C51H39BF24N2 (*M*=1146.65 g/mol):
triclinic, space group P-1 (no. 2),
*a* = 12.9518(7) Å, *b* = 13.0314(7) Å, *c* = 17.2798(10) Å, *α* = 97.8190(10)°, *β* = 107.3230(10)°, *γ* = 111.3320(10)°,
*V*= 2493.6(2) Å3,
*Z* = 2,
*T* = 100.0(1) K,
μ(MoKα) = 0.152 mm-1,
*Dcalc* = 1.527 g/cm3,
59674 reflections measured (3.49° ≤ 2Θ ≤ 61.122°),
15264 unique (*R*int = 0.0264, Rsigma = 0.0239) which were used in all calculations.
The final *R*1 was 0.0451
(I > 2σ(I)) and *wR*2 was 0.1238 (all data).

Refinement model description

Number of restraints - 447,
number of constraints - unknown.

Details:

```
1. Fixed Uiso
```

This report has been created with Olex2, compiled on
2020.02.04 svn.rd84adfe8 for OlexSys. Please
let us know
if there are any errors or if you would like to have additional features.
