# Supplementary material for: London Dispersion versus Intramolecular Hydrogen Bond in Bis‐Pyridines: How Accurate Is DFT for Competing Noncovalent Interactions in the Condensed Phase?
Source: Chemistry. 2025 Oct 23;31(66):e02745. doi: 10.1002/chem.202502745 (PMC12648470; doi:10.1002/chem.202502745)

checkCIF/PLATON report

Structure factors have been supplied for datablock(s) c040620_1_2

THIS REPORT IS FOR GUIDANCE ONLY. IF USED AS PART OF A REVIEW PROCEDURE FOR PUBLICATION, IT SHOULD NOT REPLACE THE EXPERTISE OF AN EXPERIENCED CRYSTALLOGRAPHIC REFEREE.

No syntax errors found.   CIF dictionary   Interpreting this report

Datablock: c040620_1_2

```
Bond precision:  C-C = 0.0021 A          Wavelength=0.71073

Cell:           a=12.9518(7)      b=13.0314(7)      c=17.2798(10)
                alpha=97.819(1)   beta=107.323(1)   gamma=111.332(1)
Temperature:    100 K

                 Calculated                 Reported
Volume           2493.6(2)                  2493.6(2)
Space group      P -1                       P -1
Hall group       -P 1                       -P 1
Moiety formula   C32 H12 B F24, C19 H27 N2  C32 H12 B F24, C19 H27 N2
Sum formula      C51 H39 B F24 N2           C51 H39 B F24 N2
Mr               1146.65                    1146.65
Dx,g cm-3        1.527                      1.527
Z                2                          2
Mu (mm-1)        0.152                      0.152
F000             1160.0                     1160.0
F000’            1161.04
h,k,lmax         18,18,24                   18,18,24
Nref             15293                      15264
Tmin,Tmax        0.964,0.974                0.716,0.746
Tmin’            0.958

Correction method= # Reported T Limits: Tmin=0.716 Tmax=0.746
AbsCorr = MULTI-SCAN

Data completeness= 0.998          Theta(max)= 30.561

R(reflections)= 0.0451( 12019)    wR2(reflections)= 0.1238( 15264)

S = 1.035                 Npar= 804
```

The following ALERTS were generated. Each ALERT has the format
test-name_ALERT_alert-type_alert-level.
Click on the hyperlinks for more details of the test.

Alert level C

```
PLAT213_ALERT_2_C Atom F15A           has ADP max/min Ratio .....         3.2 prolat
PLAT213_ALERT_2_C Atom F6             has ADP max/min Ratio .....         3.4 oblate
PLAT213_ALERT_2_C Atom F13            has ADP max/min Ratio .....         3.9 prolat
PLAT242_ALERT_2_C Low    'MainMol' Ueq as Compared to Neighbors of        C23 Check
PLAT242_ALERT_2_C Low    'MainMol' Ueq as Compared to Neighbors of        C1B Check
PLAT420_ALERT_2_C D-H Without Acceptor       N1B      --H1B      .     Please Check
```

Alert level G

```
PLAT002_ALERT_2_G Number of Distance or Angle Restraints on AtSite         34 Note
PLAT003_ALERT_2_G Number of Uiso or Uij Restrained non-H Atoms ...         15 Report
PLAT154_ALERT_1_G The s.u.'s on the Cell Angles are Equal ..(Note)      0.001 Degree
PLAT171_ALERT_4_G The CIF-Embedded .res File Contains EADP Records          6 Report
PLAT172_ALERT_4_G The CIF-Embedded .res File Contains DFIX Records          1 Report
PLAT176_ALERT_4_G The CIF-Embedded .res File Contains SADI Records         10 Report
PLAT186_ALERT_4_G The CIF-Embedded .res File Contains ISOR Records          2 Report
PLAT187_ALERT_4_G The CIF-Embedded .res File Contains RIGU Records          4 Report
PLAT242_ALERT_2_G Low    'MainMol' Ueq as Compared to Neighbors of         C8 Check
PLAT242_ALERT_2_G Low    'MainMol' Ueq as Compared to Neighbors of        C24 Check
PLAT242_ALERT_2_G Low    'MainMol' Ueq as Compared to Neighbors of        C31 Check
PLAT242_ALERT_2_G Low    'MainMol' Ueq as Compared to Neighbors of        C32 Check
PLAT301_ALERT_3_G Main Residue  Disorder ..............(Resd  1  )        25% Note
PLAT432_ALERT_2_G Short Inter X...Y Contact  F16      ..C4               2.97 Ang.
                                                -x,1-y,1-z  =      2_566 Check
PLAT432_ALERT_2_G Short Inter X...Y Contact  F9A      ..C10B             2.92 Ang.
                                               1+x,1+y,1+z  =      1_666 Check
PLAT434_ALERT_2_G Short Inter HL..HL Contact F18      ..F18              2.81 Ang.
                                                -x,1-y,1-z  =      2_566 Check
PLAT434_ALERT_2_G Short Inter HL..HL Contact F21      ..F11              2.82 Ang.
                                                  x,-1+y,z  =      1_545 Check
PLAT720_ALERT_4_G Number of Unusual/Non-Standard Labels ..........          9 Note
PLAT860_ALERT_3_G Number of Least-Squares Restraints .............        447 Note
PLAT910_ALERT_3_G Missing # of FCF Reflection(s) Below Theta(Min).          1 Note
PLAT912_ALERT_4_G Missing # of FCF Reflections Above STh/L=  0.600         27 Note
PLAT933_ALERT_2_G Number of OMIT Records in Embedded .res File ...          1 Note
PLAT941_ALERT_3_G Average HKL Measurement Multiplicity ...........        3.9 Low
PLAT978_ALERT_2_G Number C-C Bonds with Positive Residual Density.         16 Info
PLAT992_ALERT_5_G Repd & Actual _reflns_number_gt Values Differ by          3 Check
```

```
   0 ALERT level A = Most likely a serious problem - resolve or explain
   0 ALERT level B = A potentially serious problem, consider carefully
   6 ALERT level C = Check. Ensure it is not caused by an omission or oversight
  25 ALERT level G = General information/check it is not something unexpected

   1 ALERT type 1 CIF construction/syntax error, inconsistent or missing data
  18 ALERT type 2 Indicator that the structure model may be wrong or deficient
   4 ALERT type 3 Indicator that the structure quality may be low
   7 ALERT type 4 Improvement, methodology, query or suggestion
   1 ALERT type 5 Informative message, check
```

It is advisable to attempt to resolve as many as possible of the alerts in all categories. Often the minor alerts point to easily fixed oversights, errors and omissions in your CIF or refinement strategy, so attention to these fine details can be worthwhile. In order to resolve some of the more serious problems it may be necessary to carry out additional measurements or structure refinements. However, the purpose of your study may justify the reported deviations and the more serious of these should normally be commented upon in the discussion or experimental section of a paper or in the "special_details" fields of the CIF. checkCIF was carefully designed to identify outliers and unusual parameters, but every test has its limitations and alerts that are not important in a particular case may appear. Conversely, the absence of alerts does not guarantee there are no aspects of the results needing attention. It is up to the individual to critically assess their own results and, if necessary, seek expert advice.

Publication of your CIF in IUCr journals

A basic structural check has been run on your CIF. These basic checks will be run on all CIFs submitted for publication in IUCr journals (*Acta Crystallographica*, *Journal of Applied Crystallography*, *Journal of Synchrotron Radiation*); however, if you intend to submit to *Acta Crystallographica Section C* or *E* or *IUCrData*, you should make sure that full publication checks are run on the final version of your CIF prior to submission.

Publication of your CIF in other journals

Please refer to the *Notes for Authors* of the relevant journal for any special instructions relating to CIF submission.

PLATON version of 04/06/2020; check.def file version of 02/06/2020

Datablock c040620_1_2 - ellipsoid plot

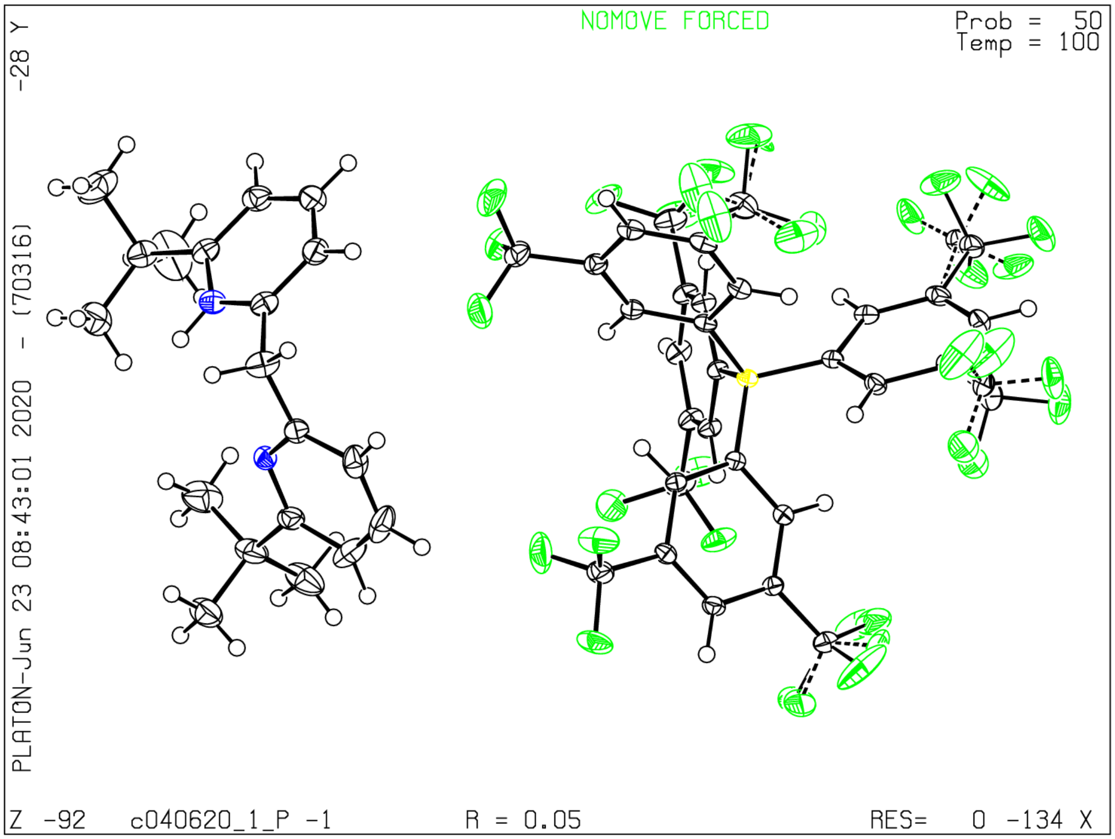

Supplement: Supplementary file 1 — Supporting Information [file CHEM-31-e02745-s002.zip › Crystal_structures/7b/checkcif.pdf]
