# Supplementary material for: London Dispersion versus Intramolecular Hydrogen Bond in Bis‐Pyridines: How Accurate Is DFT for Competing Noncovalent Interactions in the Condensed Phase?
Source: Chemistry. 2025 Oct 23;31(66):e02745. doi: 10.1002/chem.202502745 (PMC12648470; doi:10.1002/chem.202502745)
Supplement: Supplementary file 1 — Supporting Information [file CHEM-31-e02745-s002.zip › Crystal_structures/8b/c250322_1_1_tables.html]

c250322\_1\_1


# c250322\_1\_1

b"\n \n \n "

Table 1 Crystal data and structure refinement for c250322\_1\_1.

| Identification code | c250322\_1\_1 |
| Empirical formula | C42H21BF24N2O |
| Formula weight | 1036.42 |
| Temperature/K | 100.0(1) |
| Crystal system | triclinic |
| Space group | P-1 |
| a/Å | 9.76650(10) |
| b/Å | 12.6456(2) |
| c/Å | 18.0040(2) |
| α/° | 106.5840(10) |
| β/° | 93.7140(10) |
| γ/° | 98.8950(10) |
| Volume/Å3 | 2091.45(5) |
| Z | 2 |
| ρcalcg/cm3 | 1.646 |
| μ/mm‑1 | 1.571 |
| F(000) | 1032.0 |
| Crystal size/mm3 | 0.284 × 0.164 × 0.043 |
| Radiation | Cu Kα (λ = 1.54184) |
| 2Θ range for data collection/° | 5.156 to 159.544 |
| Index ranges | -12 ≤ h ≤ 12, -15 ≤ k ≤ 16, -22 ≤ l ≤ 22 |
| Reflections collected | 57108 |
| Independent reflections | 8889 [Rint = 0.0456, Rsigma = 0.0250] |
| Data/restraints/parameters | 8889/1595/992 |
| Goodness-of-fit on F2 | 1.083 |
| Final R indexes [I>=2σ (I)] | R1 = 0.0421, wR2 = 0.1096 |
| Final R indexes [all data] | R1 = 0.0488, wR2 = 0.1141 |
| Largest diff. peak/hole / e Å-3 | 0.45/-0.39 |

Table 2 Fractional Atomic Coordinates (×104) and Equivalent Isotropic Displacement Parameters (Å2×103) for c250322\_1\_1. Ueq is defined as 1/3 of the trace of the orthogonalised UIJ tensor.

| Atom | *x* | *y* | *z* | U(eq) |
| --- | --- | --- | --- | --- |
| F1 | -1080(11) | 3123(11) | 8313(7) | 45(2) |
| F2 | 778(8) | 4171(6) | 8938(7) | 75(3) |
| F3 | -83(10) | 2695(9) | 9233(4) | 60(3) |
| F4 | -617(4) | -925(5) | 6929(2) | 69.5(13) |
| F5 | 1473(5) | -1226(5) | 6802(4) | 77.5(16) |
| F6 | 386(5) | -705(5) | 5968(2) | 55.8(10) |
| F7A | 6056(18) | 4473(13) | 10419(14) | 73(5) |
| F8A | 5325(13) | 3013(11) | 10747(7) | 87(4) |
| F9A | 7502(13) | 3566(17) | 10764(11) | 63(4) |
| F10 | 6216.6(12) | -654.1(9) | 8939.9(8) | 54.1(3) |
| F11 | 6604.7(14) | -701.6(8) | 7770.3(6) | 57.1(3) |
| F12 | 8221.8(10) | 15.4(8) | 8714.2(7) | 44.8(2) |
| F13 | 2952.6(17) | 811.0(18) | 4429.1(8) | 107.0(7) |
| F14 | 4720.6(18) | 518.7(11) | 3836.1(6) | 78.3(5) |
| F15 | 4121.9(16) | 2103.6(9) | 4100.5(6) | 64.4(4) |
| F16 | 9439.7(13) | 1251.9(12) | 5760.3(8) | 64.3(3) |
| F17 | 9408.1(12) | 2637.2(11) | 5311.7(7) | 56.6(3) |
| F18 | 9647.2(11) | 2913.9(13) | 6540.6(7) | 67.4(4) |
| F19 | 9084(7) | 6088(6) | 7921(5) | 59.8(17) |
| F20 | 8275(6) | 7613(4) | 8248(3) | 70.7(13) |
| F21 | 8317(6) | 6602(4) | 9013(2) | 60.0(10) |
| F22 | 3622(4) | 6629(3) | 6358(2) | 72.9(9) |
| F23 | 2984(4) | 7167(3) | 7487.8(13) | 69.9(12) |
| F24 | 2027(2) | 5591.3(18) | 6692(2) | 64.5(10) |
| C1 | 3125.1(14) | 2243.2(11) | 7428.9(8) | 23.0(3) |
| C2 | 2312.7(14) | 2869.3(12) | 7941.3(8) | 25.7(3) |
| C3 | 988.9(15) | 2414.9(13) | 8057.7(9) | 31.4(3) |
| C4 | 405.6(15) | 1300.0(14) | 7665.4(9) | 33.1(3) |
| C5 | 1187.4(15) | 661.4(12) | 7164.5(8) | 28.9(3) |
| C6 | 2519.2(15) | 1123.2(12) | 7046.9(8) | 25.3(3) |
| C7 | 165(11) | 3114(9) | 8645(6) | 31(2) |
| C8 | 608.6(17) | -547.7(14) | 6738.1(10) | 36.1(3) |
| C9 | 5548.3(13) | 2438.7(11) | 8125.6(7) | 19.5(3) |
| C10 | 5649.2(14) | 3120.8(11) | 8900.7(8) | 22.5(3) |
| C11 | 6132.6(15) | 2783.7(12) | 9525.8(8) | 26.9(3) |
| C12 | 6533.2(15) | 1749.0(13) | 9403.7(8) | 28.8(3) |
| C13 | 6443.3(14) | 1058.9(11) | 8641.1(8) | 23.9(3) |
| C14 | 5966.2(13) | 1396.2(11) | 8016.7(7) | 20.6(3) |
| C15A | 6276(18) | 3442(13) | 10368(7) | 39(3) |
| C16 | 6856.7(16) | -65.4(13) | 8509.8(9) | 30.4(3) |
| C17 | 5369.8(15) | 2248.6(11) | 6575.9(8) | 22.6(3) |
| C18 | 4554.3(16) | 1899.8(11) | 5850.8(8) | 26.9(3) |
| C19 | 5147.7(18) | 1621.2(12) | 5149.5(8) | 32.2(3) |
| C20 | 6579.4(18) | 1707.8(14) | 5147.0(9) | 34.9(3) |
| C21 | 7409.4(17) | 2062.1(13) | 5858.9(9) | 32.5(3) |
| C22 | 6813.2(15) | 2305.5(12) | 6555.8(8) | 25.9(3) |
| C23 | 4241(2) | 1262.7(14) | 4385.0(9) | 39.2(4) |
| C24 | 8967.4(19) | 2200.1(17) | 5869.6(10) | 43.4(4) |
| C25 | 5038.5(15) | 4107.8(11) | 7533.0(8) | 23.4(3) |
| C26 | 6334.3(15) | 4781.5(12) | 7859.4(8) | 25.5(3) |
| C27 | 6645.8(17) | 5906.1(12) | 7883.9(9) | 30.5(3) |
| C28 | 5658.8(18) | 6411.6(12) | 7595.4(9) | 33.4(3) |
| C29 | 4356.3(17) | 5765.3(13) | 7278.3(9) | 31.7(3) |
| C30 | 4058.1(16) | 4637.0(12) | 7241.1(8) | 27.6(3) |
| C31 | 8061(7) | 6558(6) | 8268(5) | 39.5(16) |
| C32 | 3265(2) | 6304.5(14) | 6983.5(10) | 42.1(4) |
| B1 | 4760.4(16) | 2755.3(12) | 7407.8(8) | 21.0(3) |
| C1E | 3492(9) | 4454(7) | 4822(4) | 39.9(14) |
| N1E | 3669(6) | 5127(4) | 4375(3) | 41.1(14) |
| C5E | 2600(8) | 5213(7) | 3907(4) | 48.2(15) |
| C4E | 1298(9) | 4610(9) | 3883(6) | 50.2(17) |
| C3E | 1097(14) | 3888(16) | 4365(10) | 49(2) |
| C2E | 2196(19) | 3860(20) | 4881(10) | 51(3) |
| C7A | 204(14) | 3236(10) | 8533(7) | 37(3) |
| F2A | 556(12) | 3332(10) | 9273(4) | 60(3) |
| F3A | -1156(13) | 2868(15) | 8386(11) | 71(5) |
| F1A | 406(11) | 4257(6) | 8474(6) | 61(2) |
| C7B | 187(12) | 3052(10) | 8650(7) | 40(3) |
| F1B | 991(9) | 3889(8) | 9193(5) | 53(2) |
| F2B | -552(10) | 2464(8) | 9042(6) | 75(3) |
| F3B | -737(10) | 3507(10) | 8328(7) | 85(4) |
| F23A | 3691(6) | 7463(3) | 7205(4) | 74.8(19) |
| F24A | 2146(5) | 6201(6) | 7345(4) | 89(3) |
| F22A | 2992(8) | 6029(6) | 6276(2) | 90(3) |
| C15 | 6044(15) | 3531(11) | 10320(6) | 43(3) |
| F8 | 4750(10) | 3605(11) | 10486(7) | 55(2) |
| F7 | 6664(13) | 4584(9) | 10422(8) | 81(4) |
| F9 | 6654(12) | 3204(12) | 10879(9) | 75(4) |
| C15B | 6152(13) | 3555(10) | 10356(5) | 27(2) |
| F8B | 4922(9) | 3347(9) | 10609(6) | 52(3) |
| F7B | 6401(13) | 4642(8) | 10414(7) | 34.2(16) |
| F9B | 7090(11) | 3386(12) | 10854(10) | 47(3) |
| C1D | -856(9) | 10335(6) | 10469(4) | 22.6(13) |
| C2D | -1525(19) | 10957(13) | 11044(10) | 31(2) |
| C3D | -2690(30) | 10358(18) | 11365(16) | 37(3) |
| C4D | -2817(19) | 9321(16) | 11046(13) | 40(3) |
| C5D | -2231(19) | 8694(14) | 10508(11) | 39(3) |
| N1D | -1197(9) | 9223(5) | 10211(5) | 25.6(14) |
| O1D | 171(2) | 10906.1(18) | 10195.6(14) | 27.5(4) |
| C6D | 1022(9) | 10418(7) | 9659(5) | 21.7(14) |
| N2D | 813(8) | 9311(5) | 9352(4) | 24.9(12) |
| C10D | 1672(18) | 8889(13) | 8838(10) | 27.0(16) |
| C9D | 2600(30) | 9522(16) | 8650(15) | 32(2) |
| C8D | 3002(17) | 10785(13) | 8939(10) | 23.9(15) |
| C7D | 2041(17) | 11198(11) | 9495(8) | 25.6(18) |
| O1E | 4573(4) | 4372(2) | 5295.1(17) | 44.7(7) |
| C6E | 5922(8) | 4931(6) | 5359(4) | 42.7(13) |
| C7E | 6827(8) | 4722(6) | 5874(4) | 45.5(18) |
| C8E | 8188(9) | 5235(9) | 5965(6) | 51.8(17) |
| C9E | 8581(17) | 5948(17) | 5552(12) | 59(3) |
| C10E | 7580(20) | 6160(20) | 5095(13) | 64(4) |
| N2E | 6243(8) | 5607(6) | 4934(4) | 46.2(15) |
| F19A | 9080(13) | 6015(8) | 8032(8) | 50(2) |
| F21A | 8144(12) | 6993(9) | 8955(5) | 97(3) |
| F20A | 8336(11) | 7440(7) | 7917(6) | 78(3) |
| C31A | 8051(13) | 6573(11) | 8188(8) | 40(3) |
| F4A | -95(9) | -753(9) | 6072(5) | 67(2) |
| F6A | 1603(8) | -1184(9) | 6638(6) | 45.3(16) |
| F5A | -195(8) | -1007(7) | 7186(4) | 62.5(19) |

Table 3 Anisotropic Displacement Parameters (Å2×103) for c250322\_1\_1. The Anisotropic displacement factor exponent takes the form: -2π2[h2a\*2U11+2hka\*b\*U12+…].

| Atom | U11 | U22 | U33 | U23 | U13 | U12 |
| --- | --- | --- | --- | --- | --- | --- |
| F1 | 26(4) | 62(6) | 45(3) | 6(4) | 2(2) | 22(3) |
| F2 | 36(4) | 42(4) | 113(9) | -32(4) | 34(5) | -6(3) |
| F3 | 68(6) | 93(8) | 37(3) | 25(5) | 25(3) | 51(5) |
| F4 | 54.9(18) | 53.5(18) | 67(2) | -17.9(17) | 27.1(15) | -28.5(15) |
| F5 | 73(3) | 26.6(15) | 115(4) | 9(2) | -42(2) | 2.3(19) |
| F6 | 79(3) | 35.0(14) | 35.7(10) | -7.6(8) | 1.4(13) | -6.1(18) |
| F7A | 122(14) | 54(7) | 36(4) | -9(5) | -13(8) | 49(8) |
| F8A | 108(9) | 98(8) | 29(5) | -2(4) | 29(6) | -26(7) |
| F9A | 68(6) | 66(7) | 35(6) | -15(4) | -20(5) | 20(5) |
| F10 | 60.4(7) | 41.0(6) | 79.2(8) | 38.8(6) | 25.6(6) | 17.1(5) |
| F11 | 100.5(9) | 29.1(5) | 36.9(5) | -1.1(4) | -17.2(6) | 29.2(6) |
| F12 | 36.9(5) | 38.0(5) | 61.4(6) | 13.4(5) | -0.2(4) | 17.4(4) |
| F13 | 79.2(10) | 172.7(18) | 40.5(7) | 36.8(9) | -24.0(7) | -59.0(11) |
| F14 | 127.7(12) | 64.7(8) | 30.6(5) | -15.8(5) | -20.7(6) | 50.7(8) |
| F15 | 109.9(10) | 38.9(6) | 40.6(6) | 8.4(5) | -21.7(6) | 21.5(6) |
| F16 | 50.1(7) | 72.6(8) | 82.4(9) | 31.9(7) | 21.6(6) | 25.2(6) |
| F17 | 49.8(6) | 75.2(8) | 50.8(6) | 26.5(6) | 26.9(5) | 6.7(6) |
| F18 | 34.9(6) | 105.3(11) | 43.0(6) | 1.1(6) | 12.0(5) | -6.9(6) |
| F19 | 34.0(19) | 64(3) | 67(2) | -1.7(19) | 11.0(17) | 3.8(17) |
| F20 | 58.2(19) | 22.6(11) | 120(3) | 20.5(18) | -25(2) | -9.0(10) |
| F21 | 52.0(14) | 68(2) | 42.5(13) | 5.8(15) | -12.0(10) | -12.8(15) |
| F22 | 92(2) | 86(2) | 69.4(18) | 57.6(18) | 16.5(14) | 34.5(16) |
| F23 | 102(3) | 55.8(18) | 54.4(13) | 0.9(11) | -2.3(13) | 57.3(19) |
| F24 | 54.8(12) | 49.4(12) | 97(3) | 35.3(13) | -14.9(13) | 15.9(9) |
| C1 | 23.1(6) | 22.2(6) | 23.7(6) | 6.6(5) | 1.5(5) | 4.8(5) |
| C2 | 21.9(6) | 23.4(7) | 28.4(7) | 3.1(5) | -0.2(5) | 3.9(5) |
| C3 | 20.3(7) | 33.6(8) | 34.4(8) | 1.3(6) | 1.7(6) | 5.0(6) |
| C4 | 19.8(7) | 35.7(8) | 36.6(8) | 3.0(6) | 2.1(6) | -0.8(6) |
| C5 | 26.0(7) | 26.3(7) | 29.5(7) | 4.1(6) | 0.4(5) | -0.3(6) |
| C6 | 26.0(7) | 24.1(7) | 24.9(6) | 5.9(5) | 2.9(5) | 4.6(5) |
| C7 | 20(5) | 34(5) | 32(5) | -2(4) | 8(3) | 10(4) |
| C8 | 31.4(8) | 30.1(8) | 39.0(8) | 2.4(6) | 4.4(6) | -2.7(6) |
| C9 | 16.6(6) | 19.8(6) | 21.6(6) | 6.0(5) | 5.5(5) | 0.8(5) |
| C10 | 21.7(6) | 20.3(6) | 24.0(6) | 3.6(5) | 6.1(5) | 3.5(5) |
| C11 | 26.8(7) | 29.0(7) | 21.6(6) | 3.2(5) | 3.9(5) | 3.5(6) |
| C12 | 31.3(7) | 31.9(8) | 23.8(7) | 9.8(6) | -0.1(5) | 5.8(6) |
| C13 | 22.6(6) | 22.0(7) | 26.5(7) | 7.4(5) | 1.8(5) | 2.5(5) |
| C14 | 19.5(6) | 19.7(6) | 20.3(6) | 3.8(5) | 4.0(5) | 0.6(5) |
| C15A | 48(6) | 31(6) | 35(5) | 5(4) | 6(4) | 7(5) |
| C16 | 35.0(8) | 26.6(7) | 30.3(7) | 9.9(6) | -0.7(6) | 6.5(6) |
| C17 | 29.9(7) | 15.6(6) | 21.7(6) | 5.2(5) | 4.3(5) | 2.2(5) |
| C18 | 34.8(8) | 20.0(7) | 24.9(7) | 6.9(5) | 2.4(6) | 1.7(6) |
| C19 | 49.2(9) | 23.1(7) | 21.6(7) | 5.9(5) | 1.8(6) | 1.2(6) |
| C20 | 47.3(9) | 34.5(8) | 22.2(7) | 6.7(6) | 12.2(6) | 5.0(7) |
| C21 | 37.2(8) | 33.3(8) | 27.6(7) | 8.9(6) | 11.9(6) | 5.0(6) |
| C22 | 30.5(7) | 25.2(7) | 22.0(6) | 7.0(5) | 6.5(5) | 3.6(6) |
| C23 | 57.0(11) | 31.2(8) | 24.9(7) | 6.6(6) | -0.7(7) | 0.0(7) |
| C24 | 40.2(9) | 57.5(11) | 33.7(8) | 12.9(8) | 16.7(7) | 8.9(8) |
| C25 | 28.6(7) | 21.1(6) | 20.7(6) | 5.2(5) | 6.6(5) | 5.7(5) |
| C26 | 29.2(7) | 20.0(7) | 26.5(6) | 4.7(5) | 6.6(5) | 5.0(5) |
| C27 | 36.9(8) | 20.0(7) | 31.6(7) | 3.5(6) | 9.8(6) | 2.1(6) |
| C28 | 48.9(9) | 18.7(7) | 34.2(8) | 7.9(6) | 13.0(7) | 7.6(6) |
| C29 | 44.4(9) | 26.2(7) | 29.0(7) | 10.3(6) | 9.4(6) | 13.7(7) |
| C30 | 33.3(8) | 24.5(7) | 25.7(7) | 7.1(5) | 5.0(6) | 7.6(6) |
| C31 | 41(3) | 23(3) | 49(3) | 6(3) | 4(2) | 0(2) |
| C32 | 57.2(11) | 31.9(8) | 43.9(9) | 16.5(7) | 6.7(8) | 17.4(8) |
| B1 | 23.2(7) | 18.0(7) | 20.9(7) | 4.4(5) | 3.3(5) | 3.7(6) |
| C1E | 58(3) | 28(2) | 37(3) | 11(2) | 19(3) | 8(2) |
| N1E | 59(4) | 35(2) | 35(5) | 13(3) | 23(3) | 15(3) |
| C5E | 74(5) | 44(3) | 41(4) | 24(3) | 27(3) | 23(3) |
| C4E | 71(5) | 49(4) | 38(4) | 17(3) | 17(3) | 20(4) |
| C3E | 63(6) | 42(5) | 47(3) | 20(4) | 19(4) | 10(4) |
| C2E | 56(5) | 47(6) | 62(5) | 32(4) | 29(3) | 9(4) |
| C7A | 21(5) | 42(5) | 40(5) | 3(4) | 4(3) | -3(4) |
| F2A | 70(7) | 76(8) | 33(3) | 0(4) | 10(4) | 42(5) |
| F3A | 19(4) | 75(8) | 87(10) | -21(6) | 16(4) | -2(4) |
| F1A | 61(5) | 43(3) | 90(5) | 20(4) | 40(4) | 27(3) |
| C7B | 18(5) | 36(5) | 54(7) | 1(4) | -1(4) | -2(4) |
| F1B | 29(2) | 54(6) | 51(3) | -21(3) | 14(2) | -4(3) |
| F2B | 71(6) | 48(3) | 97(8) | 3(4) | 63(5) | -3(3) |
| F3B | 83(8) | 108(10) | 64(3) | -3(5) | -1(5) | 76(7) |
| F23A | 79(3) | 34.7(19) | 111(4) | 20(2) | -15(3) | 24.2(19) |
| F24A | 57(3) | 137(7) | 129(6) | 101(6) | 40(3) | 59(3) |
| F22A | 139(7) | 96(5) | 35.6(19) | -3(3) | -22(3) | 83(5) |
| C15 | 45(5) | 52(6) | 30(4) | 8(4) | -8(4) | 12(4) |
| F8 | 58(3) | 77(5) | 28(4) | -1(3) | 15(2) | 29(2) |
| F7 | 102(7) | 58(5) | 48(4) | -22(3) | 13(4) | -13(4) |
| F9 | 112(10) | 100(8) | 18(2) | 2(4) | -6(6) | 68(7) |
| C15B | 39(5) | 27(5) | 11(4) | -4(3) | 4(4) | 12(4) |
| F8B | 46(4) | 84(9) | 21(4) | 6(4) | 16(3) | 6(4) |
| F7B | 54(4) | 20(3) | 21(3) | -6(2) | -4(3) | 10(3) |
| F9B | 59(7) | 51(5) | 24(4) | -2(2) | -14(6) | 23(6) |
| C1D | 21.7(19) | 24(4) | 19(3) | 3(3) | 1.6(19) | 3(3) |
| C2D | 26(4) | 29(4) | 36(5) | 6(3) | 8(3) | 7(3) |
| C3D | 24(4) | 51(6) | 36(5) | 17(4) | 11(3) | 0(4) |
| C4D | 24(5) | 53(5) | 40(4) | 18(3) | 9(3) | -11(3) |
| C5D | 27(4) | 51(4) | 44(4) | 26(3) | 9(3) | -2(3) |
| N1D | 27(2) | 22(3) | 26(3) | 6(2) | 3.9(19) | 1(2) |
| O1D | 28.8(11) | 21.6(10) | 30.4(10) | 6.0(8) | 8.7(8) | 0.9(8) |
| C6D | 21(3) | 23(4) | 20(3) | 4(3) | 5.1(19) | 2(3) |
| N2D | 25.9(18) | 21(3) | 25(3) | 3.8(19) | 6(2) | 1(2) |
| C10D | 26(3) | 28(4) | 28(4) | 4(3) | 7(2) | 13(2) |
| C9D | 31(6) | 32(3) | 29(5) | 6(3) | 2(4) | 6(3) |
| C8D | 16(3) | 29(3) | 28(3) | 13(2) | 11(2) | -2(3) |
| C7D | 29(4) | 27(3) | 22(3) | 8(2) | 7(2) | 6(3) |
| O1E | 64.4(19) | 35.6(14) | 40.4(15) | 21.2(12) | 13.2(15) | 6.3(15) |
| C6E | 65(3) | 29(2) | 35(3) | 7(2) | 20(2) | 10(2) |
| C7E | 64(5) | 41(3) | 34(5) | 16(3) | 15(3) | 2(4) |
| C8E | 69(5) | 47(3) | 40(4) | 12(3) | 15(3) | 8(4) |
| C9E | 58(5) | 45(5) | 75(7) | 19(5) | 32(5) | -2(4) |
| C10E | 70(6) | 40(5) | 95(7) | 30(5) | 38(5) | 20(5) |
| N2E | 69(4) | 34(2) | 46(4) | 21(3) | 23(3) | 18(3) |
| F19A | 45(4) | 25(3) | 67(5) | 3(3) | -11(3) | -7(2) |
| F21A | 86(6) | 101(7) | 46(4) | -38(3) | 25(3) | -48(4) |
| F20A | 65(3) | 44(4) | 127(7) | 44(4) | 12(5) | -20(3) |
| C31A | 52(6) | 21(5) | 40(5) | 0(4) | 15(4) | -4(4) |
| F4A | 78(5) | 32(2) | 71(4) | 5(3) | -44(3) | -9(3) |
| F6A | 35(2) | 22(2) | 70(3) | -1.0(19) | 21(2) | 2.9(17) |
| F5A | 73(4) | 30.1(19) | 65(4) | -6(3) | 37(3) | -24(3) |

Table 4 Bond Lengths for c250322\_1\_1.

| Atom | Atom | Length/Å |  | Atom | Atom | Length/Å |
| --- | --- | --- | --- | --- | --- | --- |
| F1 | C7 | 1.323(10) |  | C21 | C24 | 1.503(2) |
| F2 | C7 | 1.316(10) |  | C25 | C26 | 1.399(2) |
| F3 | C7 | 1.331(10) |  | C25 | C30 | 1.405(2) |
| F4 | C8 | 1.322(4) |  | C25 | B1 | 1.6364(19) |
| F5 | C8 | 1.315(5) |  | C26 | C27 | 1.395(2) |
| F6 | C8 | 1.342(4) |  | C27 | C28 | 1.387(2) |
| F7A | C15A | 1.332(12) |  | C27 | C31 | 1.506(7) |
| F8A | C15A | 1.327(12) |  | C27 | C31A | 1.475(12) |
| F9A | C15A | 1.317(12) |  | C28 | C29 | 1.389(2) |
| F10 | C16 | 1.3389(18) |  | C29 | C30 | 1.393(2) |
| F11 | C16 | 1.3286(18) |  | C29 | C32 | 1.497(2) |
| F12 | C16 | 1.3402(18) |  | C32 | F23A | 1.391(4) |
| F13 | C23 | 1.316(2) |  | C32 | F24A | 1.316(4) |
| F14 | C23 | 1.322(2) |  | C32 | F22A | 1.221(4) |
| F15 | C23 | 1.322(2) |  | C1E | N1E | 1.329(8) |
| F16 | C24 | 1.318(2) |  | C1E | C2E | 1.397(17) |
| F17 | C24 | 1.341(2) |  | C1E | O1E | 1.349(7) |
| F18 | C24 | 1.343(2) |  | N1E | C5E | 1.336(8) |
| F19 | C31 | 1.339(7) |  | C5E | C4E | 1.369(10) |
| F20 | C31 | 1.329(7) |  | C4E | C3E | 1.430(17) |
| F21 | C31 | 1.332(7) |  | C3E | C2E | 1.385(19) |
| F22 | C32 | 1.352(3) |  | C7A | F2A | 1.322(12) |
| F23 | C32 | 1.284(3) |  | C7A | F3A | 1.321(12) |
| F24 | C32 | 1.364(3) |  | C7A | F1A | 1.311(12) |
| C1 | C2 | 1.4070(19) |  | C7B | F1B | 1.321(11) |
| C1 | C6 | 1.400(2) |  | C7B | F2B | 1.331(11) |
| C1 | B1 | 1.635(2) |  | C7B | F3B | 1.328(11) |
| C2 | C3 | 1.386(2) |  | C15 | F8 | 1.328(11) |
| C3 | C4 | 1.394(2) |  | C15 | F7 | 1.331(12) |
| C3 | C7 | 1.531(9) |  | C15 | F9 | 1.333(11) |
| C3 | C7A | 1.491(12) |  | C15B | F8B | 1.328(10) |
| C3 | C7B | 1.487(11) |  | C15B | F7B | 1.330(10) |
| C4 | C5 | 1.383(2) |  | C15B | F9B | 1.324(11) |
| C5 | C6 | 1.396(2) |  | C1D | C2D | 1.378(10) |
| C5 | C8 | 1.501(2) |  | C1D | N1D | 1.330(6) |
| C8 | F4A | 1.280(7) |  | C1D | O1D | 1.347(9) |
| C8 | F6A | 1.343(7) |  | C2D | C3D | 1.51(3) |
| C8 | F5A | 1.346(6) |  | C3D | C4D | 1.26(3) |
| C9 | C10 | 1.4036(18) |  | C4D | C5D | 1.29(2) |
| C9 | C14 | 1.4044(18) |  | C5D | N1D | 1.348(16) |
| C9 | B1 | 1.6384(19) |  | O1D | C6D | 1.388(9) |
| C10 | C11 | 1.392(2) |  | C6D | N2D | 1.328(6) |
| C11 | C12 | 1.385(2) |  | C6D | C7D | 1.397(15) |
| C11 | C15A | 1.493(12) |  | N2D | C10D | 1.342(10) |
| C11 | C15 | 1.488(11) |  | C10D | C9D | 1.25(3) |
| C11 | C15B | 1.531(9) |  | C9D | C8D | 1.51(3) |
| C12 | C13 | 1.390(2) |  | C8D | C7D | 1.460(19) |
| C13 | C14 | 1.3893(19) |  | O1E | C6E | 1.377(8) |
| C13 | C16 | 1.496(2) |  | C6E | C7E | 1.347(8) |
| C17 | C18 | 1.4004(19) |  | C6E | N2E | 1.317(8) |
| C17 | C22 | 1.404(2) |  | C7E | C8E | 1.364(10) |
| C17 | B1 | 1.6411(19) |  | C8E | C9E | 1.35(2) |
| C18 | C19 | 1.399(2) |  | C9E | C10E | 1.35(2) |
| C19 | C20 | 1.386(2) |  | C10E | N2E | 1.35(2) |
| C19 | C23 | 1.499(2) |  | F19A | C31A | 1.315(12) |
| C20 | C21 | 1.388(2) |  | F21A | C31A | 1.323(12) |
| C21 | C22 | 1.3920(19) |  | F20A | C31A | 1.322(12) |

Table 5 Bond Angles for c250322\_1\_1.

| Atom | Atom | Atom | Angle/˚ |  | Atom | Atom | Atom | Angle/˚ |
| --- | --- | --- | --- | --- | --- | --- | --- | --- |
| C2 | C1 | B1 | 120.42(12) |  | C28 | C27 | C31A | 118.4(6) |
| C6 | C1 | C2 | 115.86(13) |  | C27 | C28 | C29 | 118.15(14) |
| C6 | C1 | B1 | 122.95(12) |  | C28 | C29 | C30 | 120.88(14) |
| C3 | C2 | C1 | 122.37(13) |  | C28 | C29 | C32 | 118.91(14) |
| C2 | C3 | C4 | 120.62(14) |  | C30 | C29 | C32 | 120.21(15) |
| C2 | C3 | C7 | 120.6(4) |  | C29 | C30 | C25 | 122.00(14) |
| C2 | C3 | C7A | 115.0(5) |  | F19 | C31 | C27 | 111.3(6) |
| C2 | C3 | C7B | 122.0(5) |  | F20 | C31 | F19 | 106.5(6) |
| C4 | C3 | C7 | 118.7(4) |  | F20 | C31 | F21 | 106.5(5) |
| C4 | C3 | C7A | 123.9(5) |  | F20 | C31 | C27 | 113.3(5) |
| C4 | C3 | C7B | 117.2(5) |  | F21 | C31 | F19 | 104.5(6) |
| C5 | C4 | C3 | 118.24(14) |  | F21 | C31 | C27 | 114.1(6) |
| C4 | C5 | C6 | 120.98(14) |  | F22 | C32 | F24 | 101.9(2) |
| C4 | C5 | C8 | 120.06(14) |  | F22 | C32 | C29 | 111.80(19) |
| C6 | C5 | C8 | 118.96(13) |  | F23 | C32 | F22 | 107.0(2) |
| C5 | C6 | C1 | 121.93(13) |  | F23 | C32 | F24 | 106.7(2) |
| F1 | C7 | F3 | 105.3(8) |  | F23 | C32 | C29 | 114.75(17) |
| F1 | C7 | C3 | 110.9(10) |  | F24 | C32 | C29 | 113.63(15) |
| F2 | C7 | F1 | 106.2(9) |  | F23A | C32 | C29 | 110.5(2) |
| F2 | C7 | F3 | 108.3(8) |  | F24A | C32 | C29 | 110.3(2) |
| F2 | C7 | C3 | 113.1(8) |  | F24A | C32 | F23A | 102.0(4) |
| F3 | C7 | C3 | 112.5(8) |  | F22A | C32 | C29 | 115.7(3) |
| F4 | C8 | F6 | 104.5(3) |  | F22A | C32 | F23A | 104.7(4) |
| F4 | C8 | C5 | 114.3(3) |  | F22A | C32 | F24A | 112.7(4) |
| F5 | C8 | F4 | 109.1(4) |  | C1 | B1 | C9 | 102.22(10) |
| F5 | C8 | F6 | 104.1(3) |  | C1 | B1 | C17 | 114.61(11) |
| F5 | C8 | C5 | 113.2(3) |  | C1 | B1 | C25 | 112.81(11) |
| F6 | C8 | C5 | 110.7(3) |  | C9 | B1 | C17 | 112.24(11) |
| F4A | C8 | C5 | 116.7(5) |  | C25 | B1 | C9 | 112.13(11) |
| F4A | C8 | F6A | 107.3(6) |  | C25 | B1 | C17 | 103.18(10) |
| F4A | C8 | F5A | 108.0(4) |  | N1E | C1E | C2E | 123.5(9) |
| F6A | C8 | C5 | 112.3(5) |  | N1E | C1E | O1E | 120.6(7) |
| F6A | C8 | F5A | 101.3(5) |  | O1E | C1E | C2E | 115.7(9) |
| F5A | C8 | C5 | 110.0(4) |  | C1E | N1E | C5E | 120.8(6) |
| C10 | C9 | C14 | 116.09(12) |  | N1E | C5E | C4E | 120.4(7) |
| C10 | C9 | B1 | 121.49(11) |  | C5E | C4E | C3E | 119.1(8) |
| C14 | C9 | B1 | 121.74(11) |  | C2E | C3E | C4E | 119.8(11) |
| C11 | C10 | C9 | 122.01(13) |  | C3E | C2E | C1E | 115.9(12) |
| C10 | C11 | C15A | 126.2(7) |  | F2A | C7A | C3 | 107.2(9) |
| C10 | C11 | C15 | 116.5(6) |  | F3A | C7A | C3 | 111.5(13) |
| C10 | C11 | C15B | 118.8(5) |  | F3A | C7A | F2A | 106.9(11) |
| C12 | C11 | C10 | 120.83(13) |  | F1A | C7A | C3 | 118.1(9) |
| C12 | C11 | C15A | 113.0(7) |  | F1A | C7A | F2A | 106.5(10) |
| C12 | C11 | C15 | 122.4(6) |  | F1A | C7A | F3A | 106.0(10) |
| C12 | C11 | C15B | 120.3(5) |  | F1B | C7B | C3 | 112.7(9) |
| C11 | C12 | C13 | 118.23(13) |  | F1B | C7B | F2B | 104.9(9) |
| C12 | C13 | C16 | 118.24(13) |  | F1B | C7B | F3B | 106.1(9) |
| C14 | C13 | C12 | 121.00(13) |  | F2B | C7B | C3 | 116.0(10) |
| C14 | C13 | C16 | 120.76(12) |  | F3B | C7B | C3 | 111.6(10) |
| C13 | C14 | C9 | 121.85(12) |  | F3B | C7B | F2B | 104.7(9) |
| F7A | C15A | C11 | 108.4(15) |  | F8 | C15 | C11 | 114.3(11) |
| F8A | C15A | F7A | 106.5(11) |  | F8 | C15 | F7 | 104.7(10) |
| F8A | C15A | C11 | 112.0(11) |  | F8 | C15 | F9 | 106.4(9) |
| F9A | C15A | F7A | 106.3(11) |  | F7 | C15 | C11 | 112.2(11) |
| F9A | C15A | F8A | 107.2(11) |  | F7 | C15 | F9 | 106.3(10) |
| F9A | C15A | C11 | 116.0(14) |  | F9 | C15 | C11 | 112.3(12) |
| F10 | C16 | F12 | 104.77(12) |  | F8B | C15B | C11 | 109.7(9) |
| F10 | C16 | C13 | 112.49(13) |  | F8B | C15B | F7B | 106.8(9) |
| F11 | C16 | F10 | 107.36(13) |  | F7B | C15B | C11 | 113.8(10) |
| F11 | C16 | F12 | 105.85(13) |  | F9B | C15B | C11 | 112.1(11) |
| F11 | C16 | C13 | 113.48(12) |  | F9B | C15B | F8B | 106.5(9) |
| F12 | C16 | C13 | 112.27(12) |  | F9B | C15B | F7B | 107.5(9) |
| C18 | C17 | C22 | 116.03(12) |  | N1D | C1D | C2D | 121.2(10) |
| C18 | C17 | B1 | 123.58(12) |  | N1D | C1D | O1D | 122.0(6) |
| C22 | C17 | B1 | 119.73(12) |  | O1D | C1D | C2D | 116.8(9) |
| C19 | C18 | C17 | 121.77(14) |  | C1D | C2D | C3D | 118.8(14) |
| C18 | C19 | C23 | 120.18(15) |  | C4D | C3D | C2D | 108.9(18) |
| C20 | C19 | C18 | 120.95(14) |  | C3D | C4D | C5D | 135(2) |
| C20 | C19 | C23 | 118.84(14) |  | C4D | C5D | N1D | 116.5(16) |
| C19 | C20 | C21 | 118.27(13) |  | C1D | N1D | C5D | 119.5(10) |
| C20 | C21 | C22 | 120.66(15) |  | C1D | O1D | C6D | 124.9(4) |
| C20 | C21 | C24 | 119.14(14) |  | O1D | C6D | C7D | 113.4(7) |
| C22 | C21 | C24 | 120.19(14) |  | N2D | C6D | O1D | 120.1(6) |
| C21 | C22 | C17 | 122.27(14) |  | N2D | C6D | C7D | 126.5(8) |
| F13 | C23 | F14 | 106.53(17) |  | C6D | N2D | C10D | 117.3(10) |
| F13 | C23 | F15 | 105.13(18) |  | C9D | C10D | N2D | 120.7(15) |
| F13 | C23 | C19 | 113.15(14) |  | C10D | C9D | C8D | 129.4(16) |
| F14 | C23 | F15 | 105.68(14) |  | C7D | C8D | C9D | 107.6(15) |
| F14 | C23 | C19 | 112.67(16) |  | C6D | C7D | C8D | 118.5(11) |
| F15 | C23 | C19 | 113.02(14) |  | C1E | O1E | C6E | 125.3(5) |
| F16 | C24 | F17 | 106.55(14) |  | C7E | C6E | O1E | 114.8(6) |
| F16 | C24 | F18 | 107.04(16) |  | N2E | C6E | O1E | 120.1(6) |
| F16 | C24 | C21 | 113.62(16) |  | N2E | C6E | C7E | 125.1(7) |
| F17 | C24 | F18 | 105.05(15) |  | C6E | C7E | C8E | 118.3(8) |
| F17 | C24 | C21 | 111.41(15) |  | C9E | C8E | C7E | 119.4(10) |
| F18 | C24 | C21 | 112.60(13) |  | C10E | C9E | C8E | 117.5(13) |
| C26 | C25 | C30 | 115.95(13) |  | C9E | C10E | N2E | 125.0(17) |
| C26 | C25 | B1 | 121.39(12) |  | C6E | N2E | C10E | 113.9(10) |
| C30 | C25 | B1 | 122.27(12) |  | F19A | C31A | C27 | 114.9(11) |
| C27 | C26 | C25 | 122.20(14) |  | F19A | C31A | F21A | 107.1(11) |
| C26 | C27 | C31 | 117.6(3) |  | F19A | C31A | F20A | 105.7(10) |
| C26 | C27 | C31A | 120.7(6) |  | F21A | C31A | C27 | 109.9(10) |
| C28 | C27 | C26 | 120.80(15) |  | F20A | C31A | C27 | 112.6(10) |
| C28 | C27 | C31 | 121.5(3) |  | F20A | C31A | F21A | 106.1(10) |

Table 6 Torsion Angles for c250322\_1\_1.

| A | B | C | D | Angle/˚ |  | A | B | C | D | Angle/˚ |
| --- | --- | --- | --- | --- | --- | --- | --- | --- | --- | --- |
| C1 | C2 | C3 | C4 | 0.2(2) |  | C20 | C21 | C24 | F16 | -81.4(2) |
| C1 | C2 | C3 | C7 | 177.6(5) |  | C20 | C21 | C24 | F17 | 38.9(2) |
| C1 | C2 | C3 | C7A | -171.9(5) |  | C20 | C21 | C24 | F18 | 156.67(16) |
| C1 | C2 | C3 | C7B | 174.7(6) |  | C22 | C17 | C18 | C19 | 0.2(2) |
| C2 | C1 | C6 | C5 | 0.4(2) |  | C22 | C17 | B1 | C1 | -158.09(12) |
| C2 | C1 | B1 | C9 | 84.02(14) |  | C22 | C17 | B1 | C9 | -42.05(16) |
| C2 | C1 | B1 | C17 | -154.30(12) |  | C22 | C17 | B1 | C25 | 78.87(15) |
| C2 | C1 | B1 | C25 | -36.61(17) |  | C22 | C21 | C24 | F16 | 99.62(18) |
| C2 | C3 | C4 | C5 | 0.6(2) |  | C22 | C21 | C24 | F17 | -140.02(16) |
| C2 | C3 | C7 | F1 | 126.1(8) |  | C22 | C21 | C24 | F18 | -22.3(2) |
| C2 | C3 | C7 | F2 | 6.9(11) |  | C23 | C19 | C20 | C21 | 178.73(15) |
| C2 | C3 | C7 | F3 | -116.3(8) |  | C24 | C21 | C22 | C17 | 176.45(15) |
| C2 | C3 | C7A | F2A | -86.7(11) |  | C25 | C26 | C27 | C28 | 1.3(2) |
| C2 | C3 | C7A | F3A | 156.6(9) |  | C25 | C26 | C27 | C31 | 178.4(4) |
| C2 | C3 | C7A | F1A | 33.4(13) |  | C25 | C26 | C27 | C31A | -176.2(6) |
| C2 | C3 | C7B | F1B | -20.3(12) |  | C26 | C25 | C30 | C29 | -0.3(2) |
| C2 | C3 | C7B | F2B | -141.3(8) |  | C26 | C25 | B1 | C1 | 152.35(12) |
| C2 | C3 | C7B | F3B | 99.0(9) |  | C26 | C25 | B1 | C9 | 37.57(17) |
| C3 | C4 | C5 | C6 | -0.8(2) |  | C26 | C25 | B1 | C17 | -83.42(14) |
| C3 | C4 | C5 | C8 | 179.16(15) |  | C26 | C27 | C28 | C29 | -0.3(2) |
| C4 | C3 | C7 | F1 | -56.4(10) |  | C26 | C27 | C31 | F19 | 58.6(7) |
| C4 | C3 | C7 | F2 | -175.7(7) |  | C26 | C27 | C31 | F20 | 178.6(4) |
| C4 | C3 | C7 | F3 | 61.2(10) |  | C26 | C27 | C31 | F21 | -59.4(6) |
| C4 | C3 | C7A | F2A | 101.5(11) |  | C26 | C27 | C31A | F19A | 35.3(13) |
| C4 | C3 | C7A | F3A | -15.1(13) |  | C26 | C27 | C31A | F21A | -85.6(11) |
| C4 | C3 | C7A | F1A | -138.3(9) |  | C26 | C27 | C31A | F20A | 156.4(7) |
| C4 | C3 | C7B | F1B | 154.4(8) |  | C27 | C28 | C29 | C30 | -0.9(2) |
| C4 | C3 | C7B | F2B | 33.4(11) |  | C27 | C28 | C29 | C32 | 178.37(14) |
| C4 | C3 | C7B | F3B | -86.3(9) |  | C28 | C27 | C31 | F19 | -124.3(5) |
| C4 | C5 | C6 | C1 | 0.3(2) |  | C28 | C27 | C31 | F20 | -4.3(7) |
| C4 | C5 | C8 | F4 | -4.3(3) |  | C28 | C27 | C31 | F21 | 117.7(5) |
| C4 | C5 | C8 | F5 | -130.0(3) |  | C28 | C27 | C31A | F19A | -142.2(9) |
| C4 | C5 | C8 | F6 | 113.4(3) |  | C28 | C27 | C31A | F21A | 96.9(10) |
| C4 | C5 | C8 | F4A | 89.5(5) |  | C28 | C27 | C31A | F20A | -21.1(12) |
| C4 | C5 | C8 | F6A | -146.1(4) |  | C28 | C29 | C30 | C25 | 1.3(2) |
| C4 | C5 | C8 | F5A | -34.0(5) |  | C28 | C29 | C32 | F22 | 65.9(3) |
| C6 | C1 | C2 | C3 | -0.6(2) |  | C28 | C29 | C32 | F23 | -56.2(3) |
| C6 | C1 | B1 | C9 | -85.52(14) |  | C28 | C29 | C32 | F24 | -179.4(2) |
| C6 | C1 | B1 | C17 | 36.16(18) |  | C28 | C29 | C32 | F23A | -10.2(4) |
| C6 | C1 | B1 | C25 | 153.85(12) |  | C28 | C29 | C32 | F24A | -122.2(4) |
| C6 | C5 | C8 | F4 | 175.6(3) |  | C28 | C29 | C32 | F22A | 108.5(5) |
| C6 | C5 | C8 | F5 | 49.9(4) |  | C30 | C25 | C26 | C27 | -1.0(2) |
| C6 | C5 | C8 | F6 | -66.6(3) |  | C30 | C25 | B1 | C1 | -35.13(17) |
| C6 | C5 | C8 | F4A | -90.6(5) |  | C30 | C25 | B1 | C9 | -149.91(12) |
| C6 | C5 | C8 | F6A | 33.9(5) |  | C30 | C25 | B1 | C17 | 89.10(14) |
| C6 | C5 | C8 | F5A | 145.9(4) |  | C30 | C29 | C32 | F22 | -114.8(3) |
| C7 | C3 | C4 | C5 | -176.9(5) |  | C30 | C29 | C32 | F23 | 123.1(3) |
| C8 | C5 | C6 | C1 | -179.66(14) |  | C30 | C29 | C32 | F24 | -0.1(3) |
| C9 | C10 | C11 | C12 | -0.1(2) |  | C30 | C29 | C32 | F23A | 169.1(4) |
| C9 | C10 | C11 | C15A | -179.8(8) |  | C30 | C29 | C32 | F24A | 57.1(4) |
| C9 | C10 | C11 | C15 | -173.6(7) |  | C30 | C29 | C32 | F22A | -72.2(6) |
| C9 | C10 | C11 | C15B | -177.4(5) |  | C31 | C27 | C28 | C29 | -177.3(4) |
| C10 | C9 | C14 | C13 | 0.38(19) |  | C32 | C29 | C30 | C25 | -178.01(14) |
| C10 | C9 | B1 | C1 | -83.80(14) |  | B1 | C1 | C2 | C3 | -170.89(13) |
| C10 | C9 | B1 | C17 | 152.90(12) |  | B1 | C1 | C6 | C5 | 170.42(13) |
| C10 | C9 | B1 | C25 | 37.28(16) |  | B1 | C9 | C10 | C11 | 170.53(12) |
| C10 | C11 | C12 | C13 | 0.2(2) |  | B1 | C9 | C14 | C13 | -170.26(12) |
| C10 | C11 | C15A | F7A | -8.8(14) |  | B1 | C17 | C18 | C19 | 170.87(13) |
| C10 | C11 | C15A | F8A | 108.4(11) |  | B1 | C17 | C22 | C21 | -169.23(13) |
| C10 | C11 | C15A | F9A | -128.2(11) |  | B1 | C25 | C26 | C27 | 172.01(13) |
| C10 | C11 | C15 | F8 | 64.9(11) |  | B1 | C25 | C30 | C29 | -173.22(13) |
| C10 | C11 | C15 | F7 | -54.1(11) |  | C1E | N1E | C5E | C4E | 0.4(12) |
| C10 | C11 | C15 | F9 | -173.8(8) |  | C1E | O1E | C6E | C7E | 179.7(7) |
| C10 | C11 | C15B | F8B | 86.7(9) |  | C1E | O1E | C6E | N2E | -0.4(9) |
| C10 | C11 | C15B | F7B | -32.9(11) |  | N1E | C1E | C2E | C3E | 8(3) |
| C10 | C11 | C15B | F9B | -155.2(7) |  | N1E | C1E | O1E | C6E | -1.2(11) |
| C11 | C12 | C13 | C14 | 0.1(2) |  | N1E | C5E | C4E | C3E | 0.0(16) |
| C11 | C12 | C13 | C16 | -179.15(13) |  | C5E | C4E | C3E | C2E | 4(3) |
| C12 | C11 | C15A | F7A | 171.5(9) |  | C4E | C3E | C2E | C1E | -7(3) |
| C12 | C11 | C15A | F8A | -71.3(13) |  | C2E | C1E | N1E | C5E | -4.7(16) |
| C12 | C11 | C15A | F9A | 52.1(13) |  | C2E | C1E | O1E | C6E | -176.7(12) |
| C12 | C11 | C15 | F8 | -108.4(9) |  | C7A | C3 | C4 | C5 | 171.8(6) |
| C12 | C11 | C15 | F7 | 132.6(8) |  | C7B | C3 | C4 | C5 | -174.2(6) |
| C12 | C11 | C15 | F9 | 12.9(13) |  | C15 | C11 | C12 | C13 | 173.3(7) |
| C12 | C11 | C15B | F8B | -90.6(9) |  | C15B | C11 | C12 | C13 | 177.4(5) |
| C12 | C11 | C15B | F7B | 149.9(8) |  | C1D | C2D | C3D | C4D | 2(3) |
| C12 | C11 | C15B | F9B | 27.5(11) |  | C1D | O1D | C6D | N2D | 3.0(10) |
| C12 | C13 | C14 | C9 | -0.4(2) |  | C1D | O1D | C6D | C7D | -177.6(9) |
| C12 | C13 | C16 | F10 | 51.64(18) |  | C2D | C1D | N1D | C5D | 1.1(18) |
| C12 | C13 | C16 | F11 | 173.78(14) |  | C2D | C1D | O1D | C6D | 175.2(11) |
| C12 | C13 | C16 | F12 | -66.24(18) |  | C2D | C3D | C4D | C5D | -3(5) |
| C14 | C9 | C10 | C11 | -0.13(19) |  | C3D | C4D | C5D | N1D | 3(4) |
| C14 | C9 | B1 | C1 | 86.33(14) |  | C4D | C5D | N1D | C1D | -1(2) |
| C14 | C9 | B1 | C17 | -36.96(17) |  | N1D | C1D | C2D | C3D | -2(3) |
| C14 | C9 | B1 | C25 | -152.58(12) |  | N1D | C1D | O1D | C6D | -3.5(9) |
| C14 | C13 | C16 | F10 | -127.58(14) |  | O1D | C1D | C2D | C3D | 179.6(18) |
| C14 | C13 | C16 | F11 | -5.4(2) |  | O1D | C1D | N1D | C5D | 179.8(12) |
| C14 | C13 | C16 | F12 | 114.54(15) |  | O1D | C6D | N2D | C10D | -179.8(11) |
| C15A | C11 | C12 | C13 | 179.9(7) |  | O1D | C6D | C7D | C8D | 179.9(12) |
| C16 | C13 | C14 | C9 | 178.83(12) |  | C6D | N2D | C10D | C9D | -2(3) |
| C17 | C18 | C19 | C20 | -1.6(2) |  | N2D | C6D | C7D | C8D | -1(2) |
| C17 | C18 | C19 | C23 | -179.37(13) |  | N2D | C10D | C9D | C8D | 2(4) |
| C18 | C17 | C22 | C21 | 1.8(2) |  | C10D | C9D | C8D | C7D | -2(4) |
| C18 | C17 | B1 | C1 | 31.61(18) |  | C9D | C8D | C7D | C6D | 1(2) |
| C18 | C17 | B1 | C9 | 147.65(13) |  | C7D | C6D | N2D | C10D | 0.9(17) |
| C18 | C17 | B1 | C25 | -91.43(15) |  | O1E | C1E | N1E | C5E | -179.8(7) |
| C18 | C19 | C20 | C21 | 1.0(2) |  | O1E | C1E | C2E | C3E | -176.5(16) |
| C18 | C19 | C23 | F13 | -26.2(2) |  | O1E | C6E | C7E | C8E | 179.3(7) |
| C18 | C19 | C23 | F14 | -147.14(16) |  | O1E | C6E | N2E | C10E | 175.5(13) |
| C18 | C19 | C23 | F15 | 93.15(19) |  | C6E | C7E | C8E | C9E | 1.3(18) |
| C19 | C20 | C21 | C22 | 1.0(2) |  | C7E | C6E | N2E | C10E | -4.7(17) |
| C19 | C20 | C21 | C24 | -177.90(15) |  | C7E | C8E | C9E | C10E | 3(3) |
| C20 | C19 | C23 | F13 | 155.99(18) |  | C8E | C9E | C10E | N2E | -10(4) |
| C20 | C19 | C23 | F14 | 35.1(2) |  | C9E | C10E | N2E | C6E | 10(3) |
| C20 | C19 | C23 | F15 | -84.7(2) |  | N2E | C6E | C7E | C8E | -0.6(14) |
| C20 | C21 | C22 | C17 | -2.5(2) |  | C31A | C27 | C28 | C29 | 177.2(6) |

Table 7 Hydrogen Atom Coordinates (Å×104) and Isotropic Displacement Parameters (Å2×103) for c250322\_1\_1.

| Atom | *x* | *y* | *z* | U(eq) |
| --- | --- | --- | --- | --- |
| H2 | 2685.35 | 3631.16 | 8217.51 | 31 |
| H4 | -504.93 | 987.04 | 7740.01 | 40 |
| H6 | 3029.39 | 664.18 | 6697.52 | 30 |
| H10 | 5379.55 | 3834.32 | 9001.98 | 27 |
| H12 | 6860.62 | 1517.39 | 9829.95 | 35 |
| H14 | 5921.5 | 908 | 7501.71 | 25 |
| H18 | 3571.76 | 1850.99 | 5834.62 | 32 |
| H20 | 6982.03 | 1529.14 | 4670.42 | 42 |
| H22 | 7403.56 | 2517.39 | 7034.3 | 31 |
| H26 | 7025.08 | 4462.47 | 8071.41 | 31 |
| H28 | 5867.43 | 7178.44 | 7614.04 | 40 |
| H30 | 3164.65 | 4212.12 | 7011.36 | 33 |
| H5E | 2744.13 | 5694.76 | 3589.28 | 58 |
| H4E | 537.74 | 4669.9 | 3549.81 | 60 |
| H3E | 213.2 | 3430.89 | 4332.42 | 58 |
| H2E | 2074.8 | 3456.18 | 5251.73 | 61 |
| H2D | -1257.09 | 11750.35 | 11234.32 | 37 |
| H3D | -3234.7 | 10708.22 | 11747.98 | 44 |
| H4D | -3508.01 | 8898.48 | 11246.48 | 48 |
| H5D | -2513.81 | 7901.35 | 10330.38 | 46 |
| H10D | 1568.92 | 8099.64 | 8615.83 | 32 |
| H9D | 3143.84 | 9156.2 | 8268.49 | 38 |
| H8D | 3742.06 | 11230.5 | 8787.05 | 29 |
| H7D | 2107.1 | 11979.64 | 9740.39 | 31 |
| H7E | 6524.45 | 4229.2 | 6165.63 | 55 |
| H8E | 8854.69 | 5093.03 | 6315.25 | 62 |
| H9E | 9530.56 | 6286.36 | 5581.88 | 71 |
| H10E | 7825.25 | 6748.31 | 4869.04 | 76 |
| H1E | 4560(20) | 5480(30) | 4350(20) | 55 |
| H1D | -570(60) | 8920(50) | 9880(30) | 75 |

Table 8 Atomic Occupancy for c250322\_1\_1.

| Atom | *Occupancy* |  | Atom | *Occupancy* |  | Atom | *Occupancy* |
| --- | --- | --- | --- | --- | --- | --- | --- |
| F1 | 0.386(3) |  | F2 | 0.386(3) |  | F3 | 0.386(3) |
| F4 | 0.646(6) |  | F5 | 0.646(6) |  | F6 | 0.646(6) |
| F7A | 0.258(3) |  | F8A | 0.258(3) |  | F9A | 0.258(3) |
| F19 | 0.646(6) |  | F20 | 0.646(6) |  | F21 | 0.646(6) |
| F22 | 0.646(6) |  | F23 | 0.646(6) |  | F24 | 0.646(6) |
| C7 | 0.386(3) |  | C15A | 0.258(3) |  | C31 | 0.646(6) |
| C1E | 0.5 |  | N1E | 0.5 |  | C5E | 0.5 |
| H5E | 0.5 |  | C4E | 0.5 |  | H4E | 0.5 |
| C3E | 0.5 |  | H3E | 0.5 |  | C2E | 0.5 |
| H2E | 0.5 |  | C7A | 0.258(3) |  | F2A | 0.258(3) |
| F3A | 0.258(3) |  | F1A | 0.258(3) |  | C7B | 0.357(3) |
| F1B | 0.357(3) |  | F2B | 0.357(3) |  | F3B | 0.357(3) |
| F23A | 0.354(6) |  | F24A | 0.354(6) |  | F22A | 0.354(6) |
| C15 | 0.386(3) |  | F8 | 0.386(3) |  | F7 | 0.386(3) |
| F9 | 0.386(3) |  | C15B | 0.357(3) |  | F8B | 0.357(3) |
| F7B | 0.357(3) |  | F9B | 0.357(3) |  | C1D | 0.5 |
| C2D | 0.5 |  | H2D | 0.5 |  | C3D | 0.5 |
| H3D | 0.5 |  | C4D | 0.5 |  | H4D | 0.5 |
| C5D | 0.5 |  | H5D | 0.5 |  | N1D | 0.5 |
| O1D | 0.5 |  | C6D | 0.5 |  | N2D | 0.5 |
| C10D | 0.5 |  | H10D | 0.5 |  | C9D | 0.5 |
| H9D | 0.5 |  | C8D | 0.5 |  | H8D | 0.5 |
| C7D | 0.5 |  | H7D | 0.5 |  | O1E | 0.5 |
| C6E | 0.5 |  | C7E | 0.5 |  | H7E | 0.5 |
| C8E | 0.5 |  | H8E | 0.5 |  | C9E | 0.5 |
| H9E | 0.5 |  | C10E | 0.5 |  | H10E | 0.5 |
| N2E | 0.5 |  | H1E | 0.5 |  | F19A | 0.354(6) |
| F21A | 0.354(6) |  | F20A | 0.354(6) |  | C31A | 0.354(6) |
| F4A | 0.354(6) |  | F6A | 0.354(6) |  | F5A | 0.354(6) |
| H1D | 0.5 |  |  |  |  |  |

Experimental

Single crystals of C42H21BF24N2O
[c250322\_1\_1]
were
[].
A suitable crystal was selected and
[]
on a
XtaLAB Synergy, Dualflex, Pilatus 300K
diffractometer. The crystal was kept at 100.0(1) K during data collection.
Using Olex2 [1], the structure was solved with the
SHELXT
[2] structure solution program using
Intrinsic Phasing
and refined with the
SHELXL
[3] refinement package using
Least Squares
minimisation.

1. Dolomanov, O.V., Bourhis, L.J., Gildea, R.J, Howard, J.A.K. & Puschmann, H.
   (2009), J. Appl. Cryst. 42, 339-341.
2. Sheldrick, G.M. (2015). Acta Cryst. A71, 3-8.
3. Sheldrick, G.M. (2015). Acta Cryst. C71, 3-8.

Crystal structure determination of
[c250322\_1\_1]

**Crystal Data**
for C42H21BF24N2O (*M*=1036.42 g/mol):
triclinic, space group P-1 (no. 2),
*a* = 9.76650(10) Å, *b* = 12.6456(2) Å, *c* = 18.0040(2) Å, *α* = 106.5840(10)°, *β* = 93.7140(10)°, *γ* = 98.8950(10)°,
*V*= 2091.45(5) Å3,
*Z* = 2,
*T* = 100.0(1) K,
μ(Cu Kα) = 1.571 mm-1,
*Dcalc* = 1.646 g/cm3,
57108 reflections measured (5.156° ≤ 2Θ ≤ 159.544°),
8889 unique (*R*int = 0.0456, Rsigma = 0.0250) which were used in all calculations.
The final *R*1 was 0.0421
(I > 2σ(I)) and *wR*2 was 0.1141 (all data).

Refinement model description

Number of restraints - 1595,
number of constraints - unknown.

Details:

```
1. Fixed Uiso
```

This report has been created with Olex2, compiled on
2021.12.09 svn.r5202d8cf for OlexSys. Please
let us know
if there are any errors or if you would like to have additional features.
