# Supplementary material for: London Dispersion versus Intramolecular Hydrogen Bond in Bis‐Pyridines: How Accurate Is DFT for Competing Noncovalent Interactions in the Condensed Phase?
Source: Chemistry. 2025 Oct 23;31(66):e02745. doi: 10.1002/chem.202502745 (PMC12648470; doi:10.1002/chem.202502745)

checkCIF/PLATON report

Structure factors have been supplied for datablock(s) c250322_1_1

THIS REPORT IS FOR GUIDANCE ONLY. IF USED AS PART OF A REVIEW PROCEDURE FOR PUBLICATION, IT SHOULD NOT REPLACE THE EXPERTISE OF AN EXPERIENCED CRYSTALLOGRAPHIC REFEREE.

No syntax errors found. CIF dictionary Interpreting this report

Datablock: c250322_1_1

Bond precision: C-C = 0.0021 A Wavelength=1.54184

Cell:	a=9.7665(1)	b=12.6456(2)	c=18.0040(2)
	alpha=106.584(1)	beta=93.714(1)	gamma=98.895(1)
Temperature:	100 K		

	Calculated	Reported
Volume	2091.46(5)	2091.45(5)
Space group	P -1	P -1
Hall group	-P 1	-P 1
Moiety formula	C32 H12 B F24, C10 H9 N2 O	C32 H12 B F24, C10 H9 N2 O
Sum formula	C42 H21 B F24 N2 O	C42 H21 B F24 N2 O
Mr	1036.56	1036.42
Dx,g cm-3	1.646	1.646
Z	2	2
Mu (mm-1)	1.572	1.571
F000	1032.1	1032.0
F000'	1037.26	
h,k,lmax	12,16,22	12,16,22
Nref	9112	8889
Tmin,Tmax	0.771,0.935	0.423,1.000
Tmin'	0.610	

Correction method= # Reported T Limits: Tmin=0.423 Tmax=1.000
AbsCorr = GAUSSIAN

Data completeness= 0.976 Theta(max)= 79.772

R(reflections)= 0.0421(7586) wR2(reflections)= 0.1141(8889)

S = 1.083 Npar= 992

The following ALERTS were generated. Each ALERT has the format
test-name_ALERT_alert-type_alert-level.
Click on the hyperlinks for more details of the test.

Alert level C

```
PLAT088_ALERT_3_C Poor Data / Parameter Ratio ....................       8.96 Note
PLAT213_ALERT_2_C Atom F9              has ADP max/min Ratio .....        3.1 prolat
PLAT213_ALERT_2_C Atom F21A            has ADP max/min Ratio .....        3.3 prolat
PLAT220_ALERT_2_C NonSolvent   Resd 1  F   Ueq(max)/Ueq(min) Range        3.1 Ratio
PLAT250_ALERT_2_C Large U3/U1 Ratio for Average U(i,j) Tensor ....        2.3 Note
PLAT906_ALERT_3_C Large K Value in the Analysis of Variance ......      2.032 Check
PLAT911_ALERT_3_C Missing FCF Refl Between Thmin & STh/L=    0.600          8 Report
```

Alert level G

```
PLAT002_ALERT_2_G Number of Distance or Angle Restraints on AtSite         55 Note
PLAT003_ALERT_2_G Number of Uiso or Uij Restrained non-H Atoms ...         43 Report
PLAT068_ALERT_1_G Reported F000 Differs from Calcd (or Missing)...     Please Check
PLAT154_ALERT_1_G The s.u.'s on the Cell Angles are Equal ..(Note)      0.001 Degree
PLAT172_ALERT_4_G The CIF-Embedded .res File Contains DFIX Records          2 Report
PLAT176_ALERT_4_G The CIF-Embedded .res File Contains SADI Records         14 Report
PLAT178_ALERT_4_G The CIF-Embedded .res File Contains SIMU Records          4 Report
PLAT187_ALERT_4_G The CIF-Embedded .res File Contains RIGU Records          7 Report
PLAT242_ALERT_2_G Low    'MainMol' Ueq as Compared to Neighbors of         C8 Check
PLAT242_ALERT_2_G Low    'MainMol' Ueq as Compared to Neighbors of        C16 Check
PLAT242_ALERT_2_G Low    'MainMol' Ueq as Compared to Neighbors of        C23 Check
PLAT242_ALERT_2_G Low    'MainMol' Ueq as Compared to Neighbors of        C24 Check
PLAT242_ALERT_2_G Low    'MainMol' Ueq as Compared to Neighbors of        C32 Check
PLAT300_ALERT_4_G Atom Site Occupancy of O1D        Constrained at        0.5 Check
PLAT300_ALERT_4_G Atom Site Occupancy of N1D        Constrained at        0.5 Check
PLAT300_ALERT_4_G Atom Site Occupancy of N2D        Constrained at        0.5 Check
PLAT300_ALERT_4_G Atom Site Occupancy of C1D        Constrained at        0.5 Check
PLAT300_ALERT_4_G Atom Site Occupancy of C2D        Constrained at        0.5 Check
PLAT300_ALERT_4_G Atom Site Occupancy of C3D        Constrained at        0.5 Check
PLAT300_ALERT_4_G Atom Site Occupancy of C4D        Constrained at        0.5 Check
PLAT300_ALERT_4_G Atom Site Occupancy of C5D        Constrained at        0.5 Check
PLAT300_ALERT_4_G Atom Site Occupancy of C6D        Constrained at        0.5 Check
PLAT300_ALERT_4_G Atom Site Occupancy of C7D        Constrained at        0.5 Check
PLAT300_ALERT_4_G Atom Site Occupancy of C8D        Constrained at        0.5 Check
PLAT300_ALERT_4_G Atom Site Occupancy of C9D        Constrained at        0.5 Check
PLAT300_ALERT_4_G Atom Site Occupancy of C10D       Constrained at        0.5 Check
PLAT300_ALERT_4_G Atom Site Occupancy of H1D        Constrained at        0.5 Check
PLAT300_ALERT_4_G Atom Site Occupancy of H2D        Constrained at        0.5 Check
PLAT300_ALERT_4_G Atom Site Occupancy of H3D        Constrained at        0.5 Check
PLAT300_ALERT_4_G Atom Site Occupancy of H4D        Constrained at        0.5 Check
PLAT300_ALERT_4_G Atom Site Occupancy of H5D        Constrained at        0.5 Check
PLAT300_ALERT_4_G Atom Site Occupancy of H7D        Constrained at        0.5 Check
PLAT300_ALERT_4_G Atom Site Occupancy of H8D        Constrained at        0.5 Check
PLAT300_ALERT_4_G Atom Site Occupancy of H9D        Constrained at        0.5 Check
PLAT300_ALERT_4_G Atom Site Occupancy of H10D       Constrained at        0.5 Check
PLAT300_ALERT_4_G Atom Site Occupancy of O1E        Constrained at        0.5 Check
PLAT300_ALERT_4_G Atom Site Occupancy of N1E        Constrained at        0.5 Check
PLAT300_ALERT_4_G Atom Site Occupancy of N2E        Constrained at        0.5 Check
PLAT300_ALERT_4_G Atom Site Occupancy of C1E        Constrained at        0.5 Check
PLAT300_ALERT_4_G Atom Site Occupancy of C2E        Constrained at        0.5 Check
```

```
PLAT300_ALERT_4_G Atom Site Occupancy of C3E       Constrained at        0.5 Check
PLAT300_ALERT_4_G Atom Site Occupancy of C4E       Constrained at        0.5 Check
PLAT300_ALERT_4_G Atom Site Occupancy of C5E       Constrained at        0.5 Check
PLAT300_ALERT_4_G Atom Site Occupancy of C6E       Constrained at        0.5 Check
PLAT300_ALERT_4_G Atom Site Occupancy of C7E       Constrained at        0.5 Check
PLAT300_ALERT_4_G Atom Site Occupancy of C8E       Constrained at        0.5 Check
PLAT300_ALERT_4_G Atom Site Occupancy of C9E       Constrained at        0.5 Check
PLAT300_ALERT_4_G Atom Site Occupancy of C10E      Constrained at        0.5 Check
PLAT300_ALERT_4_G Atom Site Occupancy of H1E       Constrained at        0.5 Check
PLAT300_ALERT_4_G Atom Site Occupancy of H2E       Constrained at        0.5 Check
PLAT300_ALERT_4_G Atom Site Occupancy of H3E       Constrained at        0.5 Check
PLAT300_ALERT_4_G Atom Site Occupancy of H4E       Constrained at        0.5 Check
PLAT300_ALERT_4_G Atom Site Occupancy of H5E       Constrained at        0.5 Check
PLAT300_ALERT_4_G Atom Site Occupancy of H7E       Constrained at        0.5 Check
PLAT300_ALERT_4_G Atom Site Occupancy of H8E       Constrained at        0.5 Check
PLAT300_ALERT_4_G Atom Site Occupancy of H9E       Constrained at        0.5 Check
PLAT300_ALERT_4_G Atom Site Occupancy of H10E      Constrained at        0.5 Check
PLAT301_ALERT_3_G Main Residue  Disorder .............(Resd  1  )        32% Note
PLAT302_ALERT_4_G Anion/Solvent/Minor-Residue Disorder (Resd  2  )       100% Note
PLAT302_ALERT_4_G Anion/Solvent/Minor-Residue Disorder (Resd  3  )       100% Note
PLAT434_ALERT_2_G Short Inter HL..HL Contact F15      ..F22      .       2.83 Ang.
                                                1-x,1-y,1-z  =      2_666 Check
PLAT789_ALERT_4_G Atoms with Negative _atom_site_disorder_group  #         44 Check
PLAT811_ALERT_5_G No ADDSYM Analysis: Too Many Excluded Atoms ....          ! Info
PLAT860_ALERT_3_G Number of Least-Squares Restraints ............        1595 Note
PLAT912_ALERT_4_G Missing # of FCF Reflections Above STh/L=  0.600        215 Note
PLAT978_ALERT_2_G Number C-C Bonds with Positive Residual Density.          7 Info
```

---

0 **ALERT level A** = Most likely a serious problem - resolve or explain
0 **ALERT level B** = A potentially serious problem, consider carefully
7 **ALERT level C** = Check. Ensure it is not caused by an omission or oversight
66 **ALERT level G** = General information/check it is not something unexpected

2 ALERT type 1 CIF construction/syntax error, inconsistent or missing data
13 ALERT type 2 Indicator that the structure model may be wrong or deficient
5 ALERT type 3 Indicator that the structure quality may be low
52 ALERT type 4 Improvement, methodology, query or suggestion
1 ALERT type 5 Informative message, check

It is advisable to attempt to resolve as many as possible of the alerts in all categories. Often the minor alerts point to easily fixed oversights, errors and omissions in your CIF or refinement strategy, so attention to these fine details can be worthwhile. In order to resolve some of the more serious problems it may be necessary to carry out additional measurements or structure refinements. However, the purpose of your study may justify the reported deviations and the more serious of these should normally be commented upon in the discussion or experimental section of a paper or in the "special_details" fields of the CIF. checkCIF was carefully designed to identify outliers and unusual parameters, but every test has its limitations and alerts that are not important in a particular case may appear. Conversely, the absence of alerts does not guarantee there are no aspects of the results needing attention. It is up to the individual to critically assess their own results and, if necessary, seek expert advice.

Publication of your CIF in IUCr journals

A basic structural check has been run on your CIF. These basic checks will be run on all CIFs submitted for publication in IUCr journals (*Acta Crystallographica*, *Journal of Applied Crystallography*, *Journal of Synchrotron Radiation*); however, if you intend to submit to *Acta Crystallographica Section C* or *E* or *IUCrData*, you should make sure that full publication checks are run on the final version of your CIF prior to submission.

Publication of your CIF in other journals

Please refer to the *Notes for Authors* of the relevant journal for any special instructions relating to CIF submission.

PLATON version of 19/02/2022; check.def file version of 19/02/2022

Datablock c250322_1_1 - ellipsoid plot

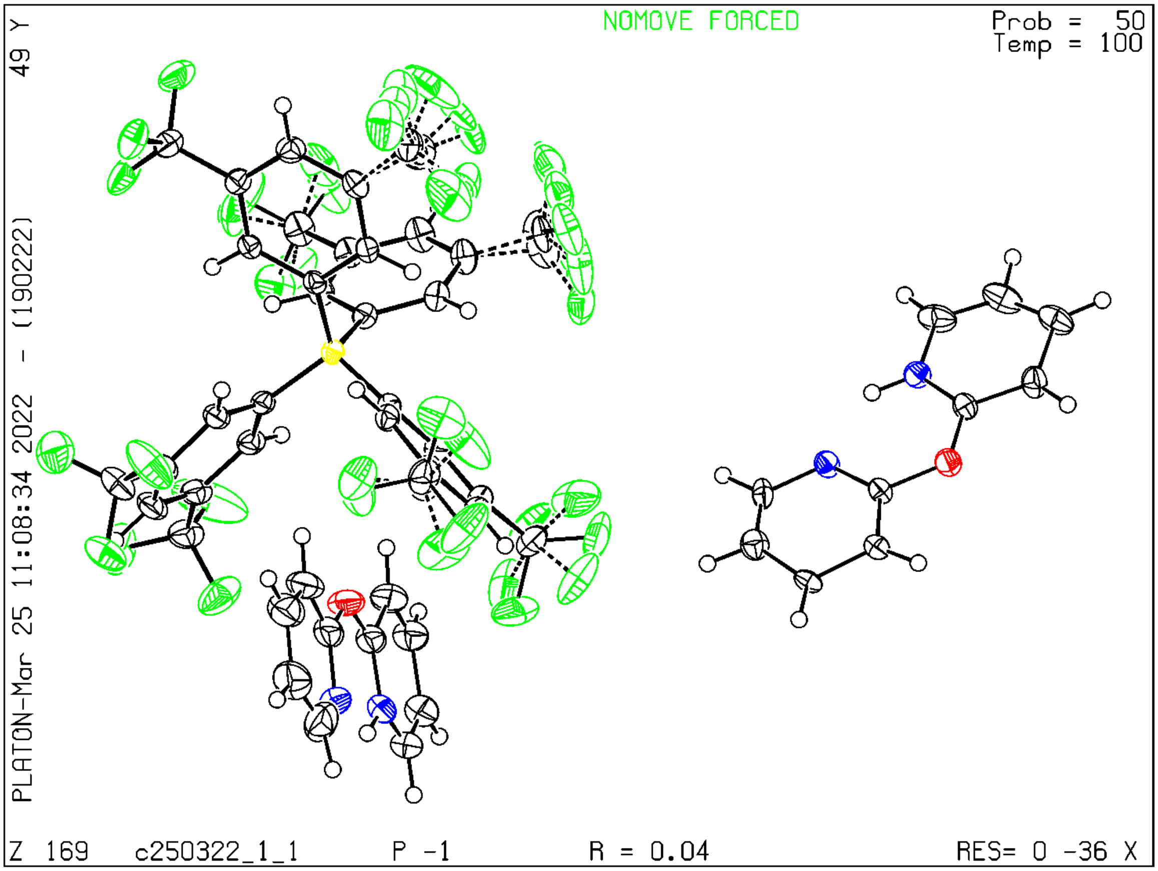

Supplement: Supplementary file 1 — Supporting Information [file CHEM-31-e02745-s002.zip › Crystal_structures/8b/checkcif.pdf]
