# Supplementary material for: London Dispersion versus Intramolecular Hydrogen Bond in Bis‐Pyridines: How Accurate Is DFT for Competing Noncovalent Interactions in the Condensed Phase?
Source: Chemistry. 2025 Oct 23;31(66):e02745. doi: 10.1002/chem.202502745 (PMC12648470; doi:10.1002/chem.202502745)
Supplement: Supplementary file 1 — Supporting Information [file CHEM-31-e02745-s002.zip › Crystal_structures/9b/c041120_1_1_tables.html]

c041120\_1\_1


# c041120\_1\_1

Table 1 Crystal data and structure refinement for c041120\_1\_1.

| Identification code | c041120\_1\_1 |
| Empirical formula | C44H25BF24N2O |
| Formula weight | 1064.47 |
| Temperature/K | 100.0(1) |
| Crystal system | triclinic |
| Space group | P-1 |
| a/Å | 10.25310(10) |
| b/Å | 10.30780(10) |
| c/Å | 21.2778(2) |
| α/° | 80.1710(10) |
| β/° | 84.4690(10) |
| γ/° | 81.6490(10) |
| Volume/Å3 | 2186.36(4) |
| Z | 2 |
| ρcalcg/cm3 | 1.617 |
| μ/mm‑1 | 1.520 |
| F(000) | 1064.0 |
| Crystal size/mm3 | 0.254 × 0.201 × 0.108 |
| Radiation | Cu Kα (λ = 1.54184) |
| 2Θ range for data collection/° | 4.226 to 160.116 |
| Index ranges | -13 ≤ h ≤ 12, -13 ≤ k ≤ 13, -26 ≤ l ≤ 27 |
| Reflections collected | 57226 |
| Independent reflections | 9276 [Rint = 0.0423, Rsigma = 0.0248] |
| Data/restraints/parameters | 9276/718/870 |
| Goodness-of-fit on F2 | 1.054 |
| Final R indexes [I>=2σ (I)] | R1 = 0.0463, wR2 = 0.1167 |
| Final R indexes [all data] | R1 = 0.0544, wR2 = 0.1225 |
| Largest diff. peak/hole / e Å-3 | 0.39/-0.37 |

Table 2 Fractional Atomic Coordinates (×104) and Equivalent Isotropic Displacement Parameters (Å2×103) for c041120\_1\_1. Ueq is defined as 1/3 of of the trace of the orthogonalised UIJ tensor.

| Atom | *x* | *y* | *z* | U(eq) |
| --- | --- | --- | --- | --- |
| F21 | 6831.2(10) | 4751.7(12) | 9301.2(5) | 42.1(3) |
| F12 | -256.2(11) | 219.1(10) | 8551.7(6) | 44.3(3) |
| F10 | -1578.8(11) | 1611.5(11) | 7963.1(5) | 43.4(3) |
| F11 | -1669.3(11) | 1636.8(12) | 8969.7(5) | 46.5(3) |
| F19 | 4730.4(11) | 5247.6(14) | 9294.7(6) | 54.9(3) |
| F20 | 5584.1(15) | 3215.5(13) | 9497.3(5) | 56.4(3) |
| F5A | -9(11) | 5797(12) | 5673(7) | 45(2) |
| F18 | 6856.4(15) | -595.0(12) | 8710.4(7) | 66.6(4) |
| F15 | 3538.7(16) | -3060.1(12) | 6992.5(8) | 67.5(4) |
| F13 | 3214.9(17) | -1395.0(14) | 6249.7(6) | 66.3(4) |
| F16 | 6408.6(16) | -2525.8(12) | 8643.8(8) | 74.4(5) |
| F2 | 6001(4) | 2369(6) | 5088(2) | 68.1(14) |
| F14 | 1830.3(13) | -1609.0(16) | 7046.4(8) | 70.2(4) |
| F9 | 1005.3(17) | 5642.2(15) | 9291.5(8) | 73.8(5) |
| F8 | -774.8(13) | 6217.5(15) | 8830.8(9) | 76.1(5) |
| F3 | 4832(5) | 3597(4) | 4389.0(16) | 63.3(10) |
| F7 | 1014(2) | 6813.5(14) | 8373.8(9) | 88.5(6) |
| N1A | -1287(15) | -308(14) | 10199(8) | 32.5(19) |
| F4A | 1351(8) | 6566(7) | 4932(2) | 36.5(12) |
| F1 | 4282(6) | 1707(4) | 4825(2) | 84.2(16) |
| F23 | 8917.1(19) | 5068(3) | 7095.7(10) | 64.6(9) |
| F17 | 7753.1(16) | -1565(2) | 7942.8(10) | 100.6(6) |
| C9 | 2228.3(16) | 3114.0(15) | 7798.9(7) | 27.4(3) |
| C25 | 4754.1(15) | 3588.1(15) | 7574.6(7) | 26.1(3) |
| C27 | 5802.7(16) | 4274.7(15) | 8429.6(8) | 27.9(3) |
| C17 | 4108.7(15) | 1242.6(15) | 7431.0(7) | 26.8(3) |
| C14 | 1320.5(16) | 2199.0(16) | 7962.7(7) | 27.7(3) |
| C1 | 3340.5(16) | 3426.0(16) | 6594.8(8) | 29.1(3) |
| C26 | 4834.7(16) | 3663.3(16) | 8218.9(8) | 27.6(3) |
| C13 | 154.9(16) | 2458.4(16) | 8343.6(7) | 28.6(3) |
| F6A | 1294(6) | 7139(6) | 5836(3) | 43.3(12) |
| C29 | 6721.6(16) | 4717.0(17) | 7359.1(8) | 31.0(3) |
| C30 | 5731.4(16) | 4136.1(16) | 7151.0(8) | 28.8(3) |
| C28 | 6757.3(16) | 4813.8(16) | 8000.2(8) | 29.6(3) |
| C18 | 3504.1(16) | 402.1(16) | 7132.9(8) | 29.3(3) |
| C12 | -129.3(17) | 3631.1(17) | 8594.8(8) | 32.5(4) |
| C22 | 5123.4(17) | 614.5(16) | 7813.4(8) | 31.5(3) |
| C19 | 3857.9(17) | -968.5(16) | 7218.1(8) | 31.9(3) |
| C10 | 1881.3(16) | 4315.6(16) | 8037.5(8) | 30.4(3) |
| C2 | 4109.6(17) | 2902.1(17) | 6095.0(8) | 32.0(3) |
| C31 | 5747.5(17) | 4374.3(18) | 9124.4(8) | 33.7(4) |
| C16 | -822.8(17) | 1485.6(17) | 8457.4(8) | 32.5(3) |
| C5 | 2199.4(18) | 5003.6(17) | 5769.9(9) | 37.0(4) |
| F22 | 7842(2) | 4797(4) | 6338.5(9) | 72.6(11) |
| C6 | 2376.6(17) | 4498.7(16) | 6410.6(8) | 31.8(3) |
| C11 | 748.2(17) | 4561.2(17) | 8434.8(9) | 33.5(4) |
| C3 | 3931.0(18) | 3398.2(19) | 5452.9(8) | 36.3(4) |
| C21 | 5489.2(18) | -751.2(17) | 7899.0(9) | 37.5(4) |
| C20 | 4859.7(18) | -1566.3(17) | 7604.5(9) | 36.8(4) |
| C4 | 2962.1(19) | 4451.0(19) | 5283.3(9) | 40.1(4) |
| C15 | 501.1(19) | 5805.8(19) | 8721.3(10) | 43.0(4) |
| C23 | 3127(2) | -1769.2(18) | 6881.5(9) | 40.8(4) |
| C32 | 7756.7(19) | 5270(2) | 6888.1(9) | 44.4(4) |
| C8 | 1140(2) | 6135(2) | 5596.8(10) | 45.1(4) |
| C7 | 4763(2) | 2748(2) | 4950.5(9) | 47.3(5) |
| B1 | 3606.0(18) | 2847.7(18) | 7347.2(8) | 26.7(3) |
| F24 | 7449(3) | 6594(2) | 6717.2(18) | 90.3(12) |
| C4A | -3535(12) | -421(14) | 10501(13) | 44(2) |
| C24 | 6620(2) | -1359(2) | 8298.1(12) | 54.2(5) |
| C2A | -2629(11) | 1572(16) | 10569(8) | 45(2) |
| O1A | -405(2) | 1674(2) | 10248.6(12) | 37.1(5) |
| C3A | -3687(4) | 851(4) | 10643.6(19) | 47.1(9) |
| C5A | -2306(4) | -989(4) | 10278.7(16) | 35.3(7) |
| C1A | -1447(3) | 936(4) | 10337.7(16) | 33.7(7) |
| C6A | -2058(8) | -2343(10) | 10109(6) | 43.0(18) |
| C7A | 828(4) | 1290(4) | 10000.6(16) | 34.8(7) |
| C8A | 1741(9) | 2170(11) | 9970(5) | 43.8(18) |
| C9A | 3011(5) | 1753(5) | 9720(2) | 52.9(11) |
| C10A | 3312(12) | 554(14) | 9499(12) | 49(3) |
| C11A | 2356(4) | -268(4) | 9527.1(17) | 40.4(8) |
| N2A | 1113(15) | 125(13) | 9789(7) | 28.6(16) |
| C12A | 2548(13) | -1558(17) | 9299(8) | 51(3) |
| N2B | 443(18) | -347(18) | 4535(6) | 39.1(19) |
| N1B | -479(17) | 334(17) | 5606(6) | 34.2(17) |
| C5B | -1445(4) | 422(4) | 6089(2) | 43.4(9) |
| C1B | 527(4) | 1017(3) | 5564.5(17) | 34.9(7) |
| C6B | -2560(13) | -367(15) | 6096(5) | 60(2) |
| C4B | -1345(10) | 1220(13) | 6531(6) | 46.3(18) |
| O1B | 1475(3) | 957(3) | 5068.0(13) | 41.3(6) |
| C2B | 663(15) | 1852(17) | 5986(7) | 38(2) |
| C3B | -279(4) | 1928(4) | 6485.7(18) | 45.6(9) |
| C7B | 1428(4) | 314(4) | 4565.4(18) | 39.9(8) |
| C8B | 2474(12) | 404(14) | 4098(4) | 51.7(19) |
| C9B | 2427(6) | -258(6) | 3594(2) | 64.1(13) |
| C11B | 375(5) | -1004(4) | 4033(2) | 48.1(10) |
| C10B | 1391(12) | -954(15) | 3558(6) | 63(2) |
| C12B | -798(17) | -1720(20) | 4049(9) | 59(3) |
| F23A | 8704(8) | 4230(6) | 6746(5) | 128(5) |
| F24A | 8488(10) | 6030(11) | 7118(3) | 116(4) |
| F22A | 7425(5) | 5877(8) | 6370(2) | 70(3) |
| F2A | 6048(8) | 2623(13) | 5046(7) | 52(2) |
| F3A | 4556(12) | 3194(11) | 4364(4) | 72(3) |
| F1A | 4561(10) | 1422(8) | 5022(4) | 54.5(19) |
| F6 | 776(5) | 6905(3) | 6079(2) | 86.7(12) |
| F4 | 1396(4) | 6962(5) | 5093(2) | 104.6(17) |
| F5 | -23(5) | 5739(7) | 5532(3) | 57.9(13) |

Table 3 Anisotropic Displacement Parameters (Å2×103) for c041120\_1\_1. The Anisotropic displacement factor exponent takes the form: -2π2[h2a\*2U11+2hka\*b\*U12+…].

| Atom | U11 | U22 | U33 | U23 | U13 | U12 |
| --- | --- | --- | --- | --- | --- | --- |
| F21 | 35.2(5) | 60.8(7) | 35.4(5) | -17.3(5) | -11.3(4) | -5.9(5) |
| F12 | 36.9(6) | 33.2(5) | 62.1(7) | -0.3(5) | -9.3(5) | -6.7(4) |
| F10 | 39.9(6) | 55.1(7) | 38.1(6) | 1.5(5) | -15.9(4) | -17.9(5) |
| F11 | 44.8(6) | 55.5(7) | 39.3(6) | -6.1(5) | 8.5(5) | -15.8(5) |
| F19 | 39.2(6) | 85.7(9) | 41.2(6) | -31.1(6) | -7.7(5) | 15.1(6) |
| F20 | 86.2(9) | 59.9(7) | 27.2(5) | 2.7(5) | -14.8(6) | -27.2(7) |
| F5A | 37(2) | 41(4) | 49(5) | 12(3) | 0(2) | -3(2) |
| F18 | 74.0(9) | 38.7(6) | 91.1(10) | 4.4(6) | -55.9(8) | -5.8(6) |
| F15 | 90.4(10) | 30.5(6) | 88.5(10) | -14.7(6) | -33.9(8) | -7.0(6) |
| F13 | 106.8(12) | 58.5(8) | 42.8(7) | -15.1(6) | -15.2(7) | -26.5(8) |
| F16 | 93.2(11) | 31.3(6) | 100.8(11) | 11.4(6) | -61.2(9) | -5.7(6) |
| F2 | 57(2) | 110(4) | 33.6(16) | -15.5(19) | 0.1(14) | 6.2(19) |
| F14 | 48.1(7) | 81.5(10) | 96.6(11) | -47.6(9) | -3.0(7) | -22.7(7) |
| F9 | 91.3(11) | 62.8(9) | 77.1(10) | -41.8(7) | -36.9(8) | 13.3(8) |
| F8 | 40.5(7) | 68.8(9) | 130.2(14) | -60.7(9) | -13.1(8) | 13.4(6) |
| F3 | 81.5(19) | 82(2) | 25.7(12) | 1.1(12) | 0.6(11) | -23.6(16) |
| F7 | 127.6(15) | 39.5(7) | 99.2(12) | -29.8(8) | 38.3(11) | -22.3(8) |
| N1A | 28(4) | 35(4) | 35(3) | -3(2) | -6(2) | -8(2) |
| F4A | 42(2) | 38(3) | 26.6(17) | 3.5(16) | -11.2(15) | 2.5(19) |
| F1 | 106(3) | 95(3) | 70(3) | -47(2) | 30(2) | -61(3) |
| F23 | 28.5(9) | 121(3) | 42.1(11) | 10.4(14) | -8.8(7) | -28.0(12) |
| F17 | 46.0(8) | 122.9(15) | 119.2(15) | -6.3(12) | -25.0(9) | 31.8(9) |
| C9 | 27.8(8) | 28.6(8) | 25.8(7) | -2.3(6) | -8.9(6) | -1.8(6) |
| C25 | 27.2(7) | 23.3(7) | 27.0(7) | -2.4(6) | -5.8(6) | 0.2(6) |
| C27 | 27.1(8) | 28.2(8) | 28.2(8) | -5.5(6) | -7.8(6) | 2.1(6) |
| C17 | 27.6(8) | 28.4(8) | 23.1(7) | -2.3(6) | -1.1(6) | -2.0(6) |
| C14 | 28.9(8) | 28.6(8) | 26.5(8) | -4.7(6) | -7.8(6) | -2.6(6) |
| C1 | 30.1(8) | 28.0(8) | 31.0(8) | -1.7(6) | -9.0(6) | -8.4(6) |
| C26 | 26.0(7) | 29.2(8) | 26.9(8) | -3.3(6) | -4.5(6) | -1.4(6) |
| C13 | 26.5(8) | 33.3(8) | 26.2(8) | -2.3(6) | -8.9(6) | -2.7(6) |
| F6A | 50(3) | 34(2) | 45(3) | -5.4(19) | -16(2) | 3.8(18) |
| C29 | 28.3(8) | 33.4(8) | 31.0(8) | -0.9(6) | -6.5(6) | -4.5(6) |
| C30 | 29.5(8) | 31.8(8) | 25.1(7) | -3.4(6) | -5.4(6) | -3.3(6) |
| C28 | 26.5(8) | 29.0(8) | 33.9(8) | -3.7(6) | -9.1(6) | -3.1(6) |
| C18 | 30.9(8) | 30.2(8) | 26.7(8) | -3.7(6) | -4.8(6) | -3.0(6) |
| C12 | 27.5(8) | 39.2(9) | 30.6(8) | -7.0(7) | -6.9(6) | 1.0(7) |
| C22 | 30.9(8) | 29.4(8) | 33.9(8) | -2.8(6) | -6.2(7) | -3.7(6) |
| C19 | 35.5(9) | 29.3(8) | 31.4(8) | -6.9(6) | 0.6(7) | -5.8(7) |
| C10 | 27.9(8) | 28.1(8) | 35.9(9) | -4.7(6) | -8.5(6) | -2.6(6) |
| C2 | 34.4(9) | 34.0(8) | 29.2(8) | -2.7(7) | -8.1(6) | -8.4(7) |
| C31 | 29.4(8) | 40.2(9) | 32.3(8) | -8.7(7) | -8.0(6) | -0.5(7) |
| C16 | 30.8(8) | 37.2(9) | 28.9(8) | -1.3(7) | -6.3(6) | -4.2(7) |
| C5 | 38.6(9) | 32.4(8) | 41.4(10) | 5.4(7) | -19.0(8) | -11.9(7) |
| F22 | 60.1(16) | 134(3) | 36.0(10) | -22.8(14) | 12.4(9) | -51.9(18) |
| C6 | 32.8(8) | 29.3(8) | 34.7(9) | -0.7(6) | -12.0(7) | -7.7(7) |
| C11 | 30.2(8) | 33.2(8) | 38.5(9) | -9.8(7) | -9.5(7) | 0.5(7) |
| C3 | 40.5(9) | 43.2(10) | 28.4(8) | -2.3(7) | -7.4(7) | -16.9(8) |
| C21 | 34.4(9) | 31.9(9) | 44.3(10) | -0.6(7) | -9.8(7) | 0.1(7) |
| C20 | 38.4(9) | 25.9(8) | 44.3(10) | -3.6(7) | -4.5(8) | -0.2(7) |
| C4 | 47.3(11) | 46.1(10) | 29.3(9) | 6.4(7) | -16.1(8) | -19.5(8) |
| C15 | 35.8(10) | 40.5(10) | 55.2(12) | -18.0(9) | -5.9(8) | 0.6(8) |
| C23 | 48.6(11) | 34.2(9) | 42.1(10) | -11.4(8) | -3.5(8) | -6.7(8) |
| C32 | 38.3(9) | 60.7(11) | 35.1(9) | 1.3(8) | -6.9(7) | -17.7(8) |
| C8 | 45.0(10) | 41.1(10) | 48.5(10) | 10.1(8) | -22.4(8) | -10.6(8) |
| C7 | 54.7(12) | 60.8(12) | 29.7(9) | -6.3(8) | -4.3(8) | -19.4(10) |
| B1 | 27.8(8) | 26.7(8) | 26.0(8) | -3.6(6) | -6.7(7) | -2.3(7) |
| F24 | 82.9(18) | 62.8(14) | 109(3) | 27.8(14) | 21.8(17) | -22.3(12) |
| C4A | 36(4) | 59(3) | 35(4) | -1(3) | -5(4) | -7(3) |
| C24 | 53.4(12) | 33.0(10) | 75.5(15) | 0.2(9) | -30.2(10) | 3.4(9) |
| C2A | 42(3) | 53(4) | 38(5) | -7(3) | -1(3) | 3(2) |
| O1A | 36.4(13) | 34.9(12) | 41.1(13) | -7.2(10) | -3.7(10) | -5.9(10) |
| C3A | 33.8(19) | 62(2) | 41(2) | -6.7(18) | 4.2(15) | 1.8(17) |
| C5A | 34.0(19) | 43.0(19) | 29.1(17) | 0.1(14) | -7.0(14) | -9.7(15) |
| C1A | 33.8(17) | 37.7(19) | 29.8(16) | -5.1(14) | -5.8(13) | -3.9(14) |
| C6A | 38(4) | 46(3) | 47(4) | -3(3) | -13(3) | -11(3) |
| C7A | 38.9(18) | 38.5(18) | 27.1(16) | -0.8(14) | -6.1(13) | -7.9(15) |
| C8A | 53(4) | 52(3) | 32(3) | -2(2) | -5(3) | -26(3) |
| C9A | 52(3) | 74(3) | 38(2) | -7(2) | 3.3(18) | -35(2) |
| C10A | 38(5) | 75(6) | 35(4) | -4(5) | 2(4) | -20(4) |
| C11A | 32.7(18) | 57(2) | 30.6(17) | -2.9(15) | -3.4(14) | -5.1(16) |
| N2A | 27(3) | 35(4) | 25(2) | 0(2) | -4(2) | -14(2) |
| C12A | 38(3) | 61(4) | 52(7) | -14(4) | -3(3) | 2(3) |
| N2B | 45(3) | 35(3) | 38(5) | -3(3) | -15(3) | -2(2) |
| N1B | 40(3) | 30(3) | 35(4) | -6(3) | -11(3) | -4.3(18) |
| C5B | 43(2) | 51(2) | 35(2) | -0.4(18) | -6.4(16) | -7.6(17) |
| C1B | 39.3(18) | 31.8(17) | 34.4(17) | -2.8(14) | -13.2(14) | -3.8(14) |
| C6B | 58(4) | 66(4) | 61(7) | -7(5) | -5(4) | -23(3) |
| C4B | 47(3) | 60(5) | 32(3) | -4(3) | -9(2) | -9(3) |
| O1B | 42.5(14) | 43.2(14) | 42.6(14) | -14.4(11) | -3.4(11) | -11.6(11) |
| C2B | 44(3) | 36(4) | 39(4) | -8(3) | -17(3) | -6(3) |
| C3B | 52(2) | 51(2) | 35.4(19) | -10.0(16) | -15.5(16) | -2.4(18) |
| C7B | 47(2) | 35.3(18) | 37.8(18) | -3.7(14) | -10.9(15) | -3.6(15) |
| C8B | 57(3) | 54(4) | 42(4) | -2(3) | 1(3) | -10(3) |
| C9B | 88(4) | 61(3) | 39(2) | -6(2) | -1(2) | -3(3) |
| C11B | 71(3) | 34.2(19) | 41(2) | -2.7(16) | -29(2) | 1.9(18) |
| C10B | 94(5) | 58(5) | 38(4) | -12(3) | -18(3) | 1(3) |
| C12B | 70(6) | 43(5) | 69(7) | -6(4) | -43(5) | -1(4) |
| F23A | 88(5) | 73(4) | 182(10) | 22(4) | 88(6) | 10(3) |
| F24A | 134(8) | 183(8) | 61(3) | -22(5) | 10(4) | -130(7) |
| F22A | 45(3) | 112(6) | 44(3) | 29(3) | -9(2) | -24(3) |
| F2A | 43(4) | 56(3) | 61(5) | -5(3) | 4(3) | -27(3) |
| F3A | 96(7) | 91(6) | 24(2) | -15(3) | -17(3) | 24(5) |
| F1A | 58(3) | 63(3) | 51(5) | -36(3) | 8(3) | -15(3) |
| F6 | 116(3) | 47.9(15) | 100(2) | -22.6(16) | -71(2) | 31.8(16) |
| F4 | 46.3(14) | 95(3) | 131(3) | 84(2) | -1(2) | 2.2(18) |
| F5 | 31.0(11) | 72(2) | 73(3) | -14.5(18) | -10.6(14) | -5.9(11) |

Table 4 Bond Lengths for c041120\_1\_1.

| Atom | Atom | Length/Å |  | Atom | Atom | Length/Å |
| --- | --- | --- | --- | --- | --- | --- |
| F21 | C31 | 1.335(2) |  | C2 | C3 | 1.394(2) |
| F12 | C16 | 1.340(2) |  | C5 | C6 | 1.393(2) |
| F10 | C16 | 1.3435(19) |  | C5 | C4 | 1.387(3) |
| F11 | C16 | 1.342(2) |  | C5 | C8 | 1.496(3) |
| F19 | C31 | 1.340(2) |  | F22 | C32 | 1.332(3) |
| F20 | C31 | 1.339(2) |  | C11 | C15 | 1.492(2) |
| F5A | C8 | 1.265(9) |  | C3 | C4 | 1.385(3) |
| F18 | C24 | 1.335(3) |  | C3 | C7 | 1.491(3) |
| F15 | C23 | 1.324(2) |  | C21 | C20 | 1.390(3) |
| F13 | C23 | 1.332(2) |  | C21 | C24 | 1.501(3) |
| F16 | C24 | 1.333(3) |  | C32 | F24 | 1.350(3) |
| F2 | C7 | 1.319(4) |  | C32 | F23A | 1.391(6) |
| F14 | C23 | 1.336(2) |  | C32 | F24A | 1.337(6) |
| F9 | C15 | 1.339(2) |  | C32 | F22A | 1.224(5) |
| F8 | C15 | 1.326(2) |  | C8 | F6 | 1.393(4) |
| F3 | C7 | 1.358(4) |  | C8 | F4 | 1.281(4) |
| F7 | C15 | 1.313(3) |  | C8 | F5 | 1.343(4) |
| N1A | C5A | 1.324(13) |  | C7 | F2A | 1.337(8) |
| N1A | C1A | 1.348(14) |  | C7 | F3A | 1.281(7) |
| F4A | C8 | 1.413(6) |  | C7 | F1A | 1.393(8) |
| F1 | C7 | 1.320(4) |  | C4A | C3A | 1.379(10) |
| F23 | C32 | 1.285(3) |  | C4A | C5A | 1.385(10) |
| F17 | C24 | 1.335(3) |  | C2A | C3A | 1.384(13) |
| C9 | C14 | 1.399(2) |  | C2A | C1A | 1.381(13) |
| C9 | C10 | 1.404(2) |  | O1A | C1A | 1.380(4) |
| C9 | B1 | 1.646(2) |  | O1A | C7A | 1.353(4) |
| C25 | C26 | 1.397(2) |  | C5A | C6A | 1.481(11) |
| C25 | C30 | 1.397(2) |  | C7A | C8A | 1.385(8) |
| C25 | B1 | 1.643(2) |  | C7A | N2A | 1.337(15) |
| C27 | C26 | 1.395(2) |  | C8A | C9A | 1.395(9) |
| C27 | C28 | 1.383(2) |  | C9A | C10A | 1.380(10) |
| C27 | C31 | 1.494(2) |  | C10A | C11A | 1.377(11) |
| C17 | C18 | 1.399(2) |  | C11A | N2A | 1.374(17) |
| C17 | C22 | 1.397(2) |  | C11A | C12A | 1.472(16) |
| C17 | B1 | 1.644(2) |  | N2B | C7B | 1.31(2) |
| C14 | C13 | 1.397(2) |  | N2B | C11B | 1.371(12) |
| C1 | C2 | 1.398(2) |  | N1B | C5B | 1.363(13) |
| C1 | C6 | 1.401(2) |  | N1B | C1B | 1.319(19) |
| C1 | B1 | 1.644(2) |  | C5B | C6B | 1.493(12) |
| C13 | C12 | 1.386(2) |  | C5B | C4B | 1.370(8) |
| C13 | C16 | 1.494(2) |  | C1B | O1B | 1.368(4) |
| F6A | C8 | 1.264(6) |  | C1B | C2B | 1.373(11) |
| C29 | C30 | 1.393(2) |  | C4B | C3B | 1.387(8) |
| C29 | C28 | 1.389(2) |  | O1B | C7B | 1.359(4) |
| C29 | C32 | 1.494(3) |  | C2B | C3B | 1.371(11) |
| C18 | C19 | 1.392(2) |  | C7B | C8B | 1.393(10) |
| C12 | C11 | 1.387(3) |  | C8B | C9B | 1.374(10) |
| C22 | C21 | 1.388(2) |  | C9B | C10B | 1.380(10) |
| C19 | C20 | 1.384(2) |  | C11B | C10B | 1.380(10) |
| C19 | C23 | 1.497(3) |  | C11B | C12B | 1.492(14) |
| C10 | C11 | 1.388(2) |  |  |  |  |

Table 5 Bond Angles for c041120\_1\_1.

| Atom | Atom | Atom | Angle/˚ |  | Atom | Atom | Atom | Angle/˚ |
| --- | --- | --- | --- | --- | --- | --- | --- | --- |
| C5A | N1A | C1A | 119.9(13) |  | F24A | C32 | F23A | 101.5(6) |
| C14 | C9 | C10 | 115.46(15) |  | F22A | C32 | C29 | 118.6(3) |
| C14 | C9 | B1 | 123.62(14) |  | F22A | C32 | F23A | 105.3(5) |
| C10 | C9 | B1 | 120.91(14) |  | F22A | C32 | F24A | 106.5(5) |
| C26 | C25 | B1 | 121.13(14) |  | F5A | C8 | F4A | 105.0(7) |
| C30 | C25 | C26 | 115.66(15) |  | F5A | C8 | C5 | 113.3(6) |
| C30 | C25 | B1 | 123.17(14) |  | F4A | C8 | C5 | 106.5(4) |
| C26 | C27 | C31 | 118.25(15) |  | F6A | C8 | F5A | 116.2(7) |
| C28 | C27 | C26 | 120.53(15) |  | F6A | C8 | F4A | 103.2(4) |
| C28 | C27 | C31 | 121.18(15) |  | F6A | C8 | C5 | 111.4(3) |
| C18 | C17 | B1 | 121.32(14) |  | F6 | C8 | C5 | 113.34(19) |
| C22 | C17 | C18 | 115.30(15) |  | F4 | C8 | C5 | 116.3(3) |
| C22 | C17 | B1 | 123.35(14) |  | F4 | C8 | F6 | 105.2(3) |
| C13 | C14 | C9 | 122.25(15) |  | F4 | C8 | F5 | 107.0(3) |
| C2 | C1 | C6 | 115.68(15) |  | F5 | C8 | C5 | 113.1(3) |
| C2 | C1 | B1 | 121.50(14) |  | F5 | C8 | F6 | 100.4(4) |
| C6 | C1 | B1 | 122.78(15) |  | F2 | C7 | F3 | 105.0(3) |
| C27 | C26 | C25 | 122.67(15) |  | F2 | C7 | F1 | 108.0(4) |
| C14 | C13 | C16 | 119.49(15) |  | F2 | C7 | C3 | 113.7(3) |
| C12 | C13 | C14 | 120.79(16) |  | F3 | C7 | C3 | 111.0(3) |
| C12 | C13 | C16 | 119.66(15) |  | F1 | C7 | F3 | 106.0(3) |
| C30 | C29 | C32 | 119.97(15) |  | F1 | C7 | C3 | 112.6(3) |
| C28 | C29 | C30 | 121.16(16) |  | F2A | C7 | C3 | 111.5(6) |
| C28 | C29 | C32 | 118.86(15) |  | F2A | C7 | F1A | 101.1(6) |
| C29 | C30 | C25 | 122.04(15) |  | F3A | C7 | C3 | 118.2(5) |
| C27 | C28 | C29 | 117.89(15) |  | F3A | C7 | F2A | 111.2(7) |
| C19 | C18 | C17 | 122.95(15) |  | F3A | C7 | F1A | 103.6(5) |
| C13 | C12 | C11 | 118.09(16) |  | F1A | C7 | C3 | 109.5(4) |
| C21 | C22 | C17 | 122.22(16) |  | C25 | B1 | C9 | 109.59(13) |
| C18 | C19 | C23 | 118.18(16) |  | C25 | B1 | C17 | 108.66(12) |
| C20 | C19 | C18 | 120.52(16) |  | C25 | B1 | C1 | 109.96(13) |
| C20 | C19 | C23 | 121.30(16) |  | C17 | B1 | C9 | 109.20(13) |
| C11 | C10 | C9 | 122.57(16) |  | C1 | B1 | C9 | 109.60(13) |
| C3 | C2 | C1 | 122.70(16) |  | C1 | B1 | C17 | 109.80(13) |
| F21 | C31 | F19 | 106.32(14) |  | C3A | C4A | C5A | 119.7(9) |
| F21 | C31 | F20 | 106.22(14) |  | F18 | C24 | C21 | 112.95(17) |
| F21 | C31 | C27 | 113.88(15) |  | F16 | C24 | F18 | 106.5(2) |
| F19 | C31 | C27 | 111.92(13) |  | F16 | C24 | F17 | 107.05(19) |
| F20 | C31 | F19 | 105.89(15) |  | F16 | C24 | C21 | 112.21(18) |
| F20 | C31 | C27 | 112.06(14) |  | F17 | C24 | F18 | 105.5(2) |
| F12 | C16 | F10 | 106.39(14) |  | F17 | C24 | C21 | 112.2(2) |
| F12 | C16 | F11 | 106.29(14) |  | C1A | C2A | C3A | 115.4(11) |
| F12 | C16 | C13 | 113.14(14) |  | C7A | O1A | C1A | 125.9(3) |
| F10 | C16 | C13 | 112.11(13) |  | C4A | C3A | C2A | 121.0(8) |
| F11 | C16 | F10 | 105.59(14) |  | N1A | C5A | C4A | 120.0(9) |
| F11 | C16 | C13 | 112.75(15) |  | N1A | C5A | C6A | 116.8(8) |
| C6 | C5 | C8 | 119.90(18) |  | C4A | C5A | C6A | 123.3(6) |
| C4 | C5 | C6 | 121.27(17) |  | N1A | C1A | C2A | 124.0(9) |
| C4 | C5 | C8 | 118.80(16) |  | N1A | C1A | O1A | 120.9(7) |
| C5 | C6 | C1 | 121.86(17) |  | O1A | C1A | C2A | 115.0(7) |
| C12 | C11 | C10 | 120.73(16) |  | O1A | C7A | C8A | 116.6(6) |
| C12 | C11 | C15 | 119.32(16) |  | N2A | C7A | O1A | 120.3(7) |
| C10 | C11 | C15 | 119.87(16) |  | N2A | C7A | C8A | 123.1(8) |
| C2 | C3 | C7 | 119.15(17) |  | C7A | C8A | C9A | 115.9(8) |
| C4 | C3 | C2 | 120.50(17) |  | C10A | C9A | C8A | 121.4(7) |
| C4 | C3 | C7 | 120.30(16) |  | C11A | C10A | C9A | 120.2(9) |
| C22 | C21 | C20 | 121.33(16) |  | C10A | C11A | C12A | 125.3(7) |
| C22 | C21 | C24 | 119.55(17) |  | N2A | C11A | C10A | 118.5(7) |
| C20 | C21 | C24 | 119.08(16) |  | N2A | C11A | C12A | 116.2(7) |
| C19 | C20 | C21 | 117.66(16) |  | C7A | N2A | C11A | 120.9(9) |
| C3 | C4 | C5 | 117.97(16) |  | C7B | N2B | C11B | 121.7(11) |
| F9 | C15 | C11 | 111.86(16) |  | C1B | N1B | C5B | 120.2(9) |
| F8 | C15 | F9 | 105.06(18) |  | N1B | C5B | C6B | 116.8(8) |
| F8 | C15 | C11 | 112.94(16) |  | N1B | C5B | C4B | 118.9(8) |
| F7 | C15 | F9 | 105.64(18) |  | C4B | C5B | C6B | 124.3(6) |
| F7 | C15 | F8 | 106.41(18) |  | N1B | C1B | O1B | 120.0(5) |
| F7 | C15 | C11 | 114.22(17) |  | N1B | C1B | C2B | 123.4(7) |
| F15 | C23 | F13 | 107.09(16) |  | O1B | C1B | C2B | 116.5(5) |
| F15 | C23 | F14 | 107.08(17) |  | C5B | C4B | C3B | 120.5(6) |
| F15 | C23 | C19 | 113.52(16) |  | C7B | O1B | C1B | 125.0(3) |
| F13 | C23 | F14 | 104.29(17) |  | C3B | C2B | C1B | 117.4(8) |
| F13 | C23 | C19 | 112.35(16) |  | C2B | C3B | C4B | 119.6(7) |
| F14 | C23 | C19 | 111.92(15) |  | N2B | C7B | O1B | 120.7(5) |
| F23 | C32 | C29 | 113.86(17) |  | N2B | C7B | C8B | 123.2(7) |
| F23 | C32 | F22 | 107.8(2) |  | O1B | C7B | C8B | 116.2(6) |
| F23 | C32 | F24 | 107.2(2) |  | C9B | C8B | C7B | 115.9(10) |
| F22 | C32 | C29 | 112.48(18) |  | C8B | C9B | C10B | 121.3(8) |
| F22 | C32 | F24 | 104.1(3) |  | N2B | C11B | C10B | 117.6(9) |
| F24 | C32 | C29 | 110.79(19) |  | N2B | C11B | C12B | 116.8(10) |
| F23A | C32 | C29 | 108.7(3) |  | C10B | C11B | C12B | 125.6(7) |
| F24A | C32 | C29 | 114.4(3) |  | C9B | C10B | C11B | 120.4(7) |

Table 6 Torsion Angles for c041120\_1\_1.

| A | B | C | D | Angle/˚ |  | A | B | C | D | Angle/˚ |
| --- | --- | --- | --- | --- | --- | --- | --- | --- | --- | --- |
| C9 | C14 | C13 | C12 | -2.2(2) |  | C6 | C1 | C2 | C3 | -0.5(2) |
| C9 | C14 | C13 | C16 | 175.12(14) |  | C6 | C1 | B1 | C9 | 28.8(2) |
| C9 | C10 | C11 | C12 | -2.6(3) |  | C6 | C1 | B1 | C25 | -91.72(18) |
| C9 | C10 | C11 | C15 | 174.26(16) |  | C6 | C1 | B1 | C17 | 148.76(15) |
| C17 | C18 | C19 | C20 | -0.9(3) |  | C6 | C5 | C4 | C3 | -1.5(3) |
| C17 | C18 | C19 | C23 | 179.14(16) |  | C6 | C5 | C8 | F5A | -74.8(8) |
| C17 | C22 | C21 | C20 | 0.2(3) |  | C6 | C5 | C8 | F4A | 170.2(3) |
| C17 | C22 | C21 | C24 | 177.90(18) |  | C6 | C5 | C8 | F6A | 58.4(4) |
| C14 | C9 | C10 | C11 | 3.0(2) |  | C6 | C5 | C8 | F6 | 23.9(3) |
| C14 | C9 | B1 | C25 | -147.49(14) |  | C6 | C5 | C8 | F4 | 146.0(4) |
| C14 | C9 | B1 | C17 | -28.6(2) |  | C6 | C5 | C8 | F5 | -89.6(4) |
| C14 | C9 | B1 | C1 | 91.75(17) |  | C20 | C19 | C23 | F15 | -0.1(3) |
| C14 | C13 | C12 | C11 | 2.7(2) |  | C20 | C19 | C23 | F13 | -121.79(19) |
| C14 | C13 | C16 | F12 | 40.3(2) |  | C20 | C19 | C23 | F14 | 121.3(2) |
| C14 | C13 | C16 | F10 | -80.06(19) |  | C20 | C21 | C24 | F18 | -159.69(19) |
| C14 | C13 | C16 | F11 | 160.93(14) |  | C20 | C21 | C24 | F16 | -39.4(3) |
| C1 | C2 | C3 | C4 | -0.1(3) |  | C20 | C21 | C24 | F17 | 81.2(3) |
| C1 | C2 | C3 | C7 | -177.78(16) |  | C4 | C5 | C6 | C1 | 0.9(3) |
| C26 | C25 | C30 | C29 | 0.1(2) |  | C4 | C5 | C8 | F5A | 103.2(8) |
| C26 | C25 | B1 | C9 | 35.71(19) |  | C4 | C5 | C8 | F4A | -11.8(4) |
| C26 | C25 | B1 | C17 | -83.55(17) |  | C4 | C5 | C8 | F6A | -123.5(3) |
| C26 | C25 | B1 | C1 | 156.25(14) |  | C4 | C5 | C8 | F6 | -158.1(3) |
| C26 | C27 | C28 | C29 | -0.4(2) |  | C4 | C5 | C8 | F4 | -35.9(4) |
| C26 | C27 | C31 | F21 | 168.62(14) |  | C4 | C5 | C8 | F5 | 88.4(4) |
| C26 | C27 | C31 | F19 | -70.8(2) |  | C4 | C3 | C7 | F2 | 143.4(3) |
| C26 | C27 | C31 | F20 | 48.0(2) |  | C4 | C3 | C7 | F3 | 25.2(3) |
| C13 | C12 | C11 | C10 | -0.4(2) |  | C4 | C3 | C7 | F1 | -93.4(3) |
| C13 | C12 | C11 | C15 | -177.23(16) |  | C4 | C3 | C7 | F2A | 130.9(6) |
| C30 | C25 | C26 | C27 | 1.6(2) |  | C4 | C3 | C7 | F3A | 0.2(7) |
| C30 | C25 | B1 | C9 | -146.62(14) |  | C4 | C3 | C7 | F1A | -118.0(4) |
| C30 | C25 | B1 | C17 | 94.12(17) |  | C23 | C19 | C20 | C21 | 179.92(17) |
| C30 | C25 | B1 | C1 | -26.1(2) |  | C32 | C29 | C30 | C25 | 179.03(16) |
| C30 | C29 | C28 | C27 | 2.1(2) |  | C32 | C29 | C28 | C27 | -178.91(16) |
| C30 | C29 | C32 | F23 | -141.8(2) |  | C8 | C5 | C6 | C1 | 178.91(16) |
| C30 | C29 | C32 | F22 | -18.7(3) |  | C8 | C5 | C4 | C3 | -179.55(16) |
| C30 | C29 | C32 | F24 | 97.3(3) |  | C7 | C3 | C4 | C5 | 178.78(17) |
| C30 | C29 | C32 | F23A | -80.1(6) |  | B1 | C9 | C14 | C13 | -179.55(14) |
| C30 | C29 | C32 | F24A | 167.2(6) |  | B1 | C9 | C10 | C11 | -178.07(15) |
| C30 | C29 | C32 | F22A | 40.1(6) |  | B1 | C25 | C26 | C27 | 179.47(14) |
| C28 | C27 | C26 | C25 | -1.5(2) |  | B1 | C25 | C30 | C29 | -177.68(14) |
| C28 | C27 | C31 | F21 | -13.6(2) |  | B1 | C17 | C18 | C19 | -176.70(15) |
| C28 | C27 | C31 | F19 | 106.98(18) |  | B1 | C17 | C22 | C21 | 177.02(16) |
| C28 | C27 | C31 | F20 | -134.23(16) |  | B1 | C1 | C2 | C3 | -178.11(15) |
| C28 | C29 | C30 | C25 | -2.0(3) |  | B1 | C1 | C6 | C5 | 177.67(15) |
| C28 | C29 | C32 | F23 | 39.3(3) |  | C24 | C21 | C20 | C19 | -177.34(19) |
| C28 | C29 | C32 | F22 | 162.3(2) |  | O1A | C7A | C8A | C9A | 179.3(6) |
| C28 | C29 | C32 | F24 | -81.7(3) |  | O1A | C7A | N2A | C11A | 179.0(7) |
| C28 | C29 | C32 | F23A | 100.9(6) |  | C3A | C4A | C5A | N1A | 0(3) |
| C28 | C29 | C32 | F24A | -11.8(7) |  | C3A | C4A | C5A | C6A | -179.4(13) |
| C28 | C29 | C32 | F22A | -138.9(5) |  | C3A | C2A | C1A | N1A | -1.2(15) |
| C18 | C17 | C22 | C21 | -1.0(2) |  | C3A | C2A | C1A | O1A | 178.3(8) |
| C18 | C17 | B1 | C9 | 76.55(18) |  | C5A | N1A | C1A | C2A | 0.7(15) |
| C18 | C17 | B1 | C25 | -163.95(14) |  | C5A | N1A | C1A | O1A | -178.9(8) |
| C18 | C17 | B1 | C1 | -43.6(2) |  | C5A | C4A | C3A | C2A | 0(3) |
| C18 | C19 | C20 | C21 | -0.1(3) |  | C1A | N1A | C5A | C4A | 0(2) |
| C18 | C19 | C23 | F15 | 179.90(16) |  | C1A | N1A | C5A | C6A | 179.2(11) |
| C18 | C19 | C23 | F13 | 58.2(2) |  | C1A | C2A | C3A | C4A | 1(2) |
| C18 | C19 | C23 | F14 | -58.7(2) |  | C1A | O1A | C7A | C8A | -178.1(6) |
| C12 | C13 | C16 | F12 | -142.40(15) |  | C1A | O1A | C7A | N2A | 3.1(8) |
| C12 | C13 | C16 | F10 | 97.28(18) |  | C7A | O1A | C1A | N1A | 2.7(9) |
| C12 | C13 | C16 | F11 | -21.7(2) |  | C7A | O1A | C1A | C2A | -176.8(8) |
| C12 | C11 | C15 | F9 | 86.1(2) |  | C7A | C8A | C9A | C10A | 2.2(18) |
| C12 | C11 | C15 | F8 | -32.2(3) |  | C8A | C7A | N2A | C11A | 0.3(14) |
| C12 | C11 | C15 | F7 | -153.96(19) |  | C8A | C9A | C10A | C11A | -1(3) |
| C22 | C17 | C18 | C19 | 1.4(2) |  | C9A | C10A | C11A | N2A | -1(3) |
| C22 | C17 | B1 | C9 | -101.37(17) |  | C9A | C10A | C11A | C12A | 179.1(14) |
| C22 | C17 | B1 | C25 | 18.1(2) |  | C10A | C11A | N2A | C7A | 1(2) |
| C22 | C17 | B1 | C1 | 138.43(16) |  | N2A | C7A | C8A | C9A | -2.0(12) |
| C22 | C21 | C20 | C19 | 0.4(3) |  | C12A | C11A | N2A | C7A | -178.9(12) |
| C22 | C21 | C24 | F18 | 22.5(3) |  | N2B | C7B | C8B | C9B | -0.5(13) |
| C22 | C21 | C24 | F16 | 142.9(2) |  | N2B | C11B | C10B | C9B | 0(2) |
| C22 | C21 | C24 | F17 | -96.6(2) |  | N1B | C5B | C4B | C3B | 0.1(19) |
| C10 | C9 | C14 | C13 | -0.7(2) |  | N1B | C1B | O1B | C7B | 5.4(10) |
| C10 | C9 | B1 | C25 | 33.68(19) |  | N1B | C1B | C2B | C3B | 3(2) |
| C10 | C9 | B1 | C17 | 152.60(14) |  | C5B | N1B | C1B | O1B | -178.1(9) |
| C10 | C9 | B1 | C1 | -87.08(17) |  | C5B | N1B | C1B | C2B | -1(2) |
| C10 | C11 | C15 | F9 | -90.7(2) |  | C5B | C4B | C3B | C2B | 1.6(19) |
| C10 | C11 | C15 | F8 | 150.98(18) |  | C1B | N1B | C5B | C6B | 179.1(13) |
| C10 | C11 | C15 | F7 | 29.2(3) |  | C1B | N1B | C5B | C4B | 0(2) |
| C2 | C1 | C6 | C5 | 0.1(2) |  | C1B | O1B | C7B | N2B | -2.0(10) |
| C2 | C1 | B1 | C9 | -153.80(15) |  | C1B | O1B | C7B | C8B | 178.1(7) |
| C2 | C1 | B1 | C25 | 85.66(18) |  | C1B | C2B | C3B | C4B | -3(2) |
| C2 | C1 | B1 | C17 | -33.8(2) |  | C6B | C5B | C4B | C3B | -179.4(10) |
| C2 | C3 | C4 | C5 | 1.1(3) |  | O1B | C1B | C2B | C3B | 179.9(10) |
| C2 | C3 | C7 | F2 | -39.0(4) |  | O1B | C7B | C8B | C9B | 179.4(7) |
| C2 | C3 | C7 | F3 | -157.1(2) |  | C2B | C1B | O1B | C7B | -171.9(10) |
| C2 | C3 | C7 | F1 | 84.3(3) |  | C7B | N2B | C11B | C10B | 0(2) |
| C2 | C3 | C7 | F2A | -51.4(6) |  | C7B | N2B | C11B | C12B | -179.7(14) |
| C2 | C3 | C7 | F3A | 177.9(6) |  | C7B | C8B | C9B | C10B | 0.6(17) |
| C2 | C3 | C7 | F1A | 59.6(4) |  | C8B | C9B | C10B | C11B | 0(2) |
| C31 | C27 | C26 | C25 | 176.23(14) |  | C11B | N2B | C7B | O1B | -179.9(9) |
| C31 | C27 | C28 | C29 | -178.10(15) |  | C11B | N2B | C7B | C8B | 0.0(17) |
| C16 | C13 | C12 | C11 | -174.61(15) |  | C12B | C11B | C10B | C9B | 179.9(14) |

Table 7 Hydrogen Atom Coordinates (Å×104) and Isotropic Displacement Parameters (Å2×103) for c041120\_1\_1.

| Atom | *x* | *y* | *z* | U(eq) |
| --- | --- | --- | --- | --- |
| H14 | 1499.24 | 1390.46 | 7812.88 | 33 |
| H26 | 4218.17 | 3290.77 | 8519.57 | 33 |
| H30 | 5720.24 | 4112.23 | 6716.63 | 35 |
| H28 | 7402.38 | 5227.86 | 8136.41 | 36 |
| H18 | 2835.61 | 775.97 | 6865.79 | 35 |
| H12 | -886.59 | 3790.11 | 8862.96 | 39 |
| H22 | 5568.98 | 1128.37 | 8017.42 | 38 |
| H10 | 2432.16 | 4972.88 | 7925.06 | 36 |
| H2 | 4767.17 | 2193.49 | 6195.01 | 38 |
| H6 | 1839.83 | 4884.67 | 6724.97 | 38 |
| H20 | 5102.75 | -2481.44 | 7664.81 | 44 |
| H4 | 2826.83 | 4777.6 | 4856.15 | 48 |
| H4A | -4253.52 | -895.17 | 10554.64 | 53 |
| H2A | -2708.65 | 2425.76 | 10668.69 | 54 |
| H3A | -4513.56 | 1230.17 | 10791.68 | 57 |
| H6AA | -2885.09 | -2673.42 | 10105.39 | 64 |
| H6AB | -1529.51 | -2918.77 | 10418.23 | 64 |
| H6AC | -1598.34 | -2312.92 | 9692.64 | 64 |
| H8A | 1520.34 | 2988.62 | 10107.83 | 53 |
| H9A | 3668.53 | 2293.76 | 9702.99 | 63 |
| H10A | 4163.32 | 301.35 | 9329.28 | 59 |
| H2AA | 509.4 | -385.5 | 9816.59 | 34 |
| H12A | 2072.55 | -1486.26 | 8924.63 | 76 |
| H12B | 2225.39 | -2218.58 | 9627.7 | 76 |
| H12C | 3472.5 | -1808.93 | 9195.17 | 76 |
| H2B | -177.41 | -369.19 | 4837.73 | 47 |
| H6BA | -2995.99 | -88.06 | 5706.21 | 90 |
| H6BB | -3178.17 | -226.29 | 6453.65 | 90 |
| H6BC | -2221.75 | -1293.03 | 6130.74 | 90 |
| H4B | -1996.83 | 1286.8 | 6863.68 | 56 |
| H2BA | 1366.2 | 2347.49 | 5933.67 | 46 |
| H3B | -203.91 | 2450.64 | 6792.63 | 55 |
| H8B | 3162.66 | 882.57 | 4125.72 | 62 |
| H9B | 3106.49 | -237.57 | 3271.42 | 77 |
| H10B | 1377.19 | -1390.77 | 3211.42 | 76 |
| H12D | -787.57 | -2415.23 | 4410.79 | 88 |
| H12E | -767.97 | -2088.95 | 3663.11 | 88 |
| H12F | -1591.51 | -1104.07 | 4083.13 | 88 |

Table 8 Atomic Occupancy for c041120\_1\_1.

| Atom | *Occupancy* |  | Atom | *Occupancy* |  | Atom | *Occupancy* |
| --- | --- | --- | --- | --- | --- | --- | --- |
| F5A | 0.294(6) |  | F2 | 0.706(6) |  | F3 | 0.706(6) |
| N1A | 0.5 |  | F4A | 0.294(6) |  | F1 | 0.706(6) |
| F23 | 0.706(6) |  | F6A | 0.294(6) |  | F22 | 0.706(6) |
| F24 | 0.706(6) |  | C4A | 0.5 |  | H4A | 0.5 |
| C2A | 0.5 |  | H2A | 0.5 |  | O1A | 0.5 |
| C3A | 0.5 |  | H3A | 0.5 |  | C5A | 0.5 |
| C1A | 0.5 |  | C6A | 0.5 |  | H6AA | 0.5 |
| H6AB | 0.5 |  | H6AC | 0.5 |  | C7A | 0.5 |
| C8A | 0.5 |  | H8A | 0.5 |  | C9A | 0.5 |
| H9A | 0.5 |  | C10A | 0.5 |  | H10A | 0.5 |
| C11A | 0.5 |  | N2A | 0.5 |  | H2AA | 0.5 |
| C12A | 0.5 |  | H12A | 0.5 |  | H12B | 0.5 |
| H12C | 0.5 |  | N2B | 0.5 |  | H2B | 0.5 |
| N1B | 0.5 |  | C5B | 0.5 |  | C1B | 0.5 |
| C6B | 0.5 |  | H6BA | 0.5 |  | H6BB | 0.5 |
| H6BC | 0.5 |  | C4B | 0.5 |  | H4B | 0.5 |
| O1B | 0.5 |  | C2B | 0.5 |  | H2BA | 0.5 |
| C3B | 0.5 |  | H3B | 0.5 |  | C7B | 0.5 |
| C8B | 0.5 |  | H8B | 0.5 |  | C9B | 0.5 |
| H9B | 0.5 |  | C11B | 0.5 |  | C10B | 0.5 |
| H10B | 0.5 |  | C12B | 0.5 |  | H12D | 0.5 |
| H12E | 0.5 |  | H12F | 0.5 |  | F23A | 0.294(6) |
| F24A | 0.294(6) |  | F22A | 0.294(6) |  | F2A | 0.294(6) |
| F3A | 0.294(6) |  | F1A | 0.294(6) |  | F6 | 0.706(6) |
| F4 | 0.706(6) |  | F5 | 0.706(6) |  |  |  |

Experimental

Single crystals of C44H25BF24N2O
[c041120\_1\_1]
were
[].
A suitable crystal was selected and
[]
on a
XtaLAB Synergy, Dualflex, Pilatus 300K
diffractometer. The crystal was kept at 100.0(1) K during data collection.
Using Olex2 [1], the structure was solved with the
XT
[2] structure solution program using
Intrinsic Phasing
and refined with the
SHELXL
[3] refinement package using
Least Squares
minimisation.

1. Dolomanov, O.V., Bourhis, L.J., Gildea, R.J, Howard, J.A.K. & Puschmann, H.
   (2009), J. Appl. Cryst. 42, 339-341.
2. Sheldrick, G.M. (2015). Acta Cryst. A71, 3-8.
3. Sheldrick, G.M. (2015). Acta Cryst. C71, 3-8.

Crystal structure determination of
[c041120\_1\_1]

**Crystal Data**
for C44H25BF24N2O (*M*=1064.47 g/mol):
triclinic, space group P-1 (no. 2),
*a* = 10.25310(10) Å, *b* = 10.30780(10) Å, *c* = 21.2778(2) Å, *α* = 80.1710(10)°, *β* = 84.4690(10)°, *γ* = 81.6490(10)°,
*V*= 2186.36(4) Å3,
*Z* = 2,
*T* = 100.0(1) K,
μ(Cu Kα) = 1.520 mm-1,
*Dcalc* = 1.617 g/cm3,
57226 reflections measured (4.226° ≤ 2Θ ≤ 160.116°),
9276 unique (*R*int = 0.0423, Rsigma = 0.0248) which were used in all calculations.
The final *R*1 was 0.0463
(I > 2σ(I)) and *wR*2 was 0.1225 (all data).

Refinement model description

Number of restraints - 718,
number of constraints - unknown.

Details:

```
1. Fixed Uiso
```

This report has been created with Olex2, compiled on
2020.07.31 svn.rc2a77460 for OlexSys. Please
let us know
if there are any errors or if you would like to have additional features.
