# Supplementary material for: London Dispersion versus Intramolecular Hydrogen Bond in Bis‐Pyridines: How Accurate Is DFT for Competing Noncovalent Interactions in the Condensed Phase?
Source: Chemistry. 2025 Oct 23;31(66):e02745. doi: 10.1002/chem.202502745 (PMC12648470; doi:10.1002/chem.202502745)

checkCIF/PLATON report

Structure factors have been supplied for datablock(s) c041120_1_1

THIS REPORT IS FOR GUIDANCE ONLY. IF USED AS PART OF A REVIEW PROCEDURE FOR PUBLICATION, IT SHOULD NOT REPLACE THE EXPERTISE OF AN EXPERIENCED CRYSTALLOGRAPHIC REFEREE.

No syntax errors found. CIF dictionary Interpreting this report

Datablock: c041120_1_1

```
Bond precision:  C-C = 0.0023 A          Wavelength=1.54184

Cell:           a=10.2531(1)      b=10.3078(1)     c=21.2778(2)
                alpha=80.171(1)   beta=84.469(1)   gamma=81.649(1)
Temperature:    100 K
```

	Calculated	Reported
Volume	2186.36(4)	2186.36(4)
Space group	P -1	P -1
Hall group	-P 1	-P 1
Moiety formula	C32 H12 B F24, C12 H13 N2 O	C32 H12 B F24, C12 H13 N2 O
Sum formula	C44 H25 B F24 N2 O	C44 H25 B F24 N2 O
Mr	1064.47	1064.47
Dx,g cm-3	1.617	1.617
Z	2	2
Mu (mm-1)	1.520	1.520
F000	1064.0	1064.0
F000’	1069.20	
h,k,lmax	13,13,27	13,13,27
Nref	9526	9276
Tmin,Tmax	0.728,0.849	0.585,1.000
Tmin’	0.647	

```
Correction method= # Reported T Limits: Tmin=0.585 Tmax=1.000
AbsCorr = GAUSSIAN

Data completeness= 0.974          Theta(max)= 80.058

R(reflections)= 0.0463( 7804)     wR2(reflections)= 0.1225( 9276)

S = 1.054                  Npar= 870
```

The following ALERTS were generated. Each ALERT has the format
test-name_ALERT_alert-type_alert-level.
Click on the hyperlinks for more details of the test.

Alert level C

```
PLAT213_ALERT_2_C Atom F4              has ADP max/min Ratio .....        3.3 prolat
PLAT220_ALERT_2_C NonSolvent   Resd 1  F   Ueq(max)/Ueq(min) Range        3.5 Ratio
PLAT906_ALERT_3_C Large K Value in the Analysis of Variance ......      2.955 Check
PLAT911_ALERT_3_C Missing FCF Refl Between Thmin & STh/L=    0.600          5 Report
```

Alert level G

```
PLAT002_ALERT_2_G Number of Distance or Angle Restraints on AtSite         48 Note
PLAT003_ALERT_2_G Number of Uiso or Uij Restrained non-H Atoms ...         20 Report
PLAT007_ALERT_5_G Number of Unrefined Donor-H Atoms .............           2 Report
PLAT154_ALERT_1_G The s.u.’s on the Cell Angles are Equal ..(Note)      0.001 Degree
PLAT174_ALERT_4_G The CIF-Embedded .res File Contains FLAT Records          3 Report
PLAT176_ALERT_4_G The CIF-Embedded .res File Contains SADI Records         14 Report
PLAT186_ALERT_4_G The CIF-Embedded .res File Contains ISOR Records          3 Report
PLAT187_ALERT_4_G The CIF-Embedded .res File Contains RIGU Records          6 Report
PLAT230_ALERT_2_G Hirshfeld Test Diff for    F4A      --C8       .        9.5 s.u.
PLAT242_ALERT_2_G Low    ’MainMol’ Ueq as Compared to Neighbors of         C7 Check
PLAT242_ALERT_2_G Low    ’MainMol’ Ueq as Compared to Neighbors of         C8 Check
PLAT242_ALERT_2_G Low    ’MainMol’ Ueq as Compared to Neighbors of        C15 Check
PLAT242_ALERT_2_G Low    ’MainMol’ Ueq as Compared to Neighbors of        C16 Check
PLAT242_ALERT_2_G Low    ’MainMol’ Ueq as Compared to Neighbors of        C23 Check
PLAT242_ALERT_2_G Low    ’MainMol’ Ueq as Compared to Neighbors of        C24 Check
PLAT242_ALERT_2_G Low    ’MainMol’ Ueq as Compared to Neighbors of        C31 Check
PLAT242_ALERT_2_G Low    ’MainMol’ Ueq as Compared to Neighbors of        C32 Check
PLAT300_ALERT_4_G Atom Site Occupancy of O1A        Constrained at        0.5 Check
PLAT300_ALERT_4_G Atom Site Occupancy of N1A        Constrained at        0.5 Check
PLAT300_ALERT_4_G Atom Site Occupancy of N2A        Constrained at        0.5 Check
PLAT300_ALERT_4_G Atom Site Occupancy of C1A        Constrained at        0.5 Check
PLAT300_ALERT_4_G Atom Site Occupancy of C2A        Constrained at        0.5 Check
PLAT300_ALERT_4_G Atom Site Occupancy of C3A        Constrained at        0.5 Check
PLAT300_ALERT_4_G Atom Site Occupancy of C4A        Constrained at        0.5 Check
PLAT300_ALERT_4_G Atom Site Occupancy of C5A        Constrained at        0.5 Check
PLAT300_ALERT_4_G Atom Site Occupancy of C6A        Constrained at        0.5 Check
PLAT300_ALERT_4_G Atom Site Occupancy of C7A        Constrained at        0.5 Check
PLAT300_ALERT_4_G Atom Site Occupancy of C8A        Constrained at        0.5 Check
PLAT300_ALERT_4_G Atom Site Occupancy of C9A        Constrained at        0.5 Check
PLAT300_ALERT_4_G Atom Site Occupancy of C10A       Constrained at        0.5 Check
PLAT300_ALERT_4_G Atom Site Occupancy of C11A       Constrained at        0.5 Check
PLAT300_ALERT_4_G Atom Site Occupancy of C12A       Constrained at        0.5 Check
PLAT300_ALERT_4_G Atom Site Occupancy of H6AA       Constrained at        0.5 Check
PLAT300_ALERT_4_G Atom Site Occupancy of H6AB       Constrained at        0.5 Check
PLAT300_ALERT_4_G Atom Site Occupancy of H2A        Constrained at        0.5 Check
PLAT300_ALERT_4_G Atom Site Occupancy of H6AC       Constrained at        0.5 Check
PLAT300_ALERT_4_G Atom Site Occupancy of H3A        Constrained at        0.5 Check
PLAT300_ALERT_4_G Atom Site Occupancy of H2AA       Constrained at        0.5 Check
PLAT300_ALERT_4_G Atom Site Occupancy of H4A        Constrained at        0.5 Check
PLAT300_ALERT_4_G Atom Site Occupancy of H8A        Constrained at        0.5 Check
PLAT300_ALERT_4_G Atom Site Occupancy of H9A        Constrained at        0.5 Check
PLAT300_ALERT_4_G Atom Site Occupancy of H10A       Constrained at        0.5 Check
PLAT300_ALERT_4_G Atom Site Occupancy of H12A       Constrained at        0.5 Check
PLAT300_ALERT_4_G Atom Site Occupancy of H12B       Constrained at        0.5 Check
PLAT300_ALERT_4_G Atom Site Occupancy of H12C       Constrained at        0.5 Check
PLAT300_ALERT_4_G Atom Site Occupancy of O1B        Constrained at        0.5 Check
PLAT300_ALERT_4_G Atom Site Occupancy of N1B        Constrained at        0.5 Check
PLAT300_ALERT_4_G Atom Site Occupancy of N2B        Constrained at        0.5 Check
```

```
PLAT300_ALERT_4_G Atom Site Occupancy of C1B        Constrained at        0.5 Check
PLAT300_ALERT_4_G Atom Site Occupancy of C2B        Constrained at        0.5 Check
PLAT300_ALERT_4_G Atom Site Occupancy of C3B        Constrained at        0.5 Check
PLAT300_ALERT_4_G Atom Site Occupancy of C4B        Constrained at        0.5 Check
PLAT300_ALERT_4_G Atom Site Occupancy of C5B        Constrained at        0.5 Check
PLAT300_ALERT_4_G Atom Site Occupancy of C6B        Constrained at        0.5 Check
PLAT300_ALERT_4_G Atom Site Occupancy of C7B        Constrained at        0.5 Check
PLAT300_ALERT_4_G Atom Site Occupancy of C8B        Constrained at        0.5 Check
PLAT300_ALERT_4_G Atom Site Occupancy of C9B        Constrained at        0.5 Check
PLAT300_ALERT_4_G Atom Site Occupancy of C10B       Constrained at        0.5 Check
PLAT300_ALERT_4_G Atom Site Occupancy of C11B       Constrained at        0.5 Check
PLAT300_ALERT_4_G Atom Site Occupancy of C12B       Constrained at        0.5 Check
PLAT300_ALERT_4_G Atom Site Occupancy of H2B        Constrained at        0.5 Check
PLAT300_ALERT_4_G Atom Site Occupancy of H3B        Constrained at        0.5 Check
PLAT300_ALERT_4_G Atom Site Occupancy of H4B        Constrained at        0.5 Check
PLAT300_ALERT_4_G Atom Site Occupancy of H6BA       Constrained at        0.5 Check
PLAT300_ALERT_4_G Atom Site Occupancy of H6BB       Constrained at        0.5 Check
PLAT300_ALERT_4_G Atom Site Occupancy of H6BC       Constrained at        0.5 Check
PLAT300_ALERT_4_G Atom Site Occupancy of H2BA       Constrained at        0.5 Check
PLAT300_ALERT_4_G Atom Site Occupancy of H8B        Constrained at        0.5 Check
PLAT300_ALERT_4_G Atom Site Occupancy of H9B        Constrained at        0.5 Check
PLAT300_ALERT_4_G Atom Site Occupancy of H10B       Constrained at        0.5 Check
PLAT300_ALERT_4_G Atom Site Occupancy of H12D       Constrained at        0.5 Check
PLAT300_ALERT_4_G Atom Site Occupancy of H12E       Constrained at        0.5 Check
PLAT300_ALERT_4_G Atom Site Occupancy of H12F       Constrained at        0.5 Check
PLAT301_ALERT_3_G Main Residue  Disorder ..............(Resd  1  )        16% Note
PLAT302_ALERT_4_G Anion/Solvent/Minor-Residue Disorder (Resd  2  )       100% Note
PLAT302_ALERT_4_G Anion/Solvent/Minor-Residue Disorder (Resd  3  )       100% Note
PLAT380_ALERT_4_G Incorrectly? Oriented X(sp2)-Methyl Moiety .....        C6B Check
PLAT432_ALERT_2_G Short Inter X...Y Contact  F11      ..C1A              2.90 Ang.
                                                      x,y,z  =      1_555 Check
PLAT720_ALERT_4_G Number of Unusual/Non-Standard Labels ..........          8 Note
PLAT789_ALERT_4_G Atoms with Negative _atom_site_disorder_group  #         56 Check
PLAT811_ALERT_5_G No ADDSYM Analysis: Too Many Excluded Atoms ....          ! Info
PLAT860_ALERT_3_G Number of Least-Squares Restraints .............        718 Note
PLAT912_ALERT_4_G Missing # of FCF Reflections Above STh/L=  0.600        246 Note
PLAT978_ALERT_2_G Number C-C Bonds with Positive Residual Density.          2 Info
PLAT992_ALERT_5_G Repd & Actual _reflns_number_gt Values Differ by          2 Check
```

```
   0 ALERT level A = Most likely a serious problem - resolve or explain
   0 ALERT level B = A potentially serious problem, consider carefully
   4 ALERT level C = Check. Ensure it is not caused by an omission or oversight
  85 ALERT level G = General information/check it is not something unexpected

   1 ALERT type 1 CIF construction/syntax error, inconsistent or missing data
  15 ALERT type 2 Indicator that the structure model may be wrong or deficient
   4 ALERT type 3 Indicator that the structure quality may be low
  66 ALERT type 4 Improvement, methodology, query or suggestion
   3 ALERT type 5 Informative message, check
```

It is advisable to attempt to resolve as many as possible of the alerts in all categories. Often the minor alerts point to easily fixed oversights, errors and omissions in your CIF or refinement strategy, so attention to these fine details can be worthwhile. In order to resolve some of the more serious problems it may be necessary to carry out additional measurements or structure refinements. However, the purpose of your study may justify the reported deviations and the more serious of these should normally be commented upon in the discussion or experimental section of a paper or in the "special_details" fields of the CIF. checkCIF was carefully designed to identify outliers and unusual parameters, but every test has its limitations and alerts that are not important in a particular case may appear. Conversely, the absence of alerts does not guarantee there are no aspects of the results needing attention. It is up to the individual to critically assess their own results and, if necessary, seek expert advice.

Publication of your CIF in IUCr journals

A basic structural check has been run on your CIF. These basic checks will be run on all CIFs submitted for publication in IUCr journals (*Acta Crystallographica*, *Journal of Applied Crystallography*, *Journal of Synchrotron Radiation*); however, if you intend to submit to *Acta Crystallographica Section C* or *E* or *IUCrData*, you should make sure that full publication checks are run on the final version of your CIF prior to submission.

Publication of your CIF in other journals

Please refer to the *Notes for Authors* of the relevant journal for any special instructions relating to CIF submission.

PLATON version of 18/09/2020; check.def file version of 20/08/2020

Datablock c041120_1_1 - ellipsoid plot

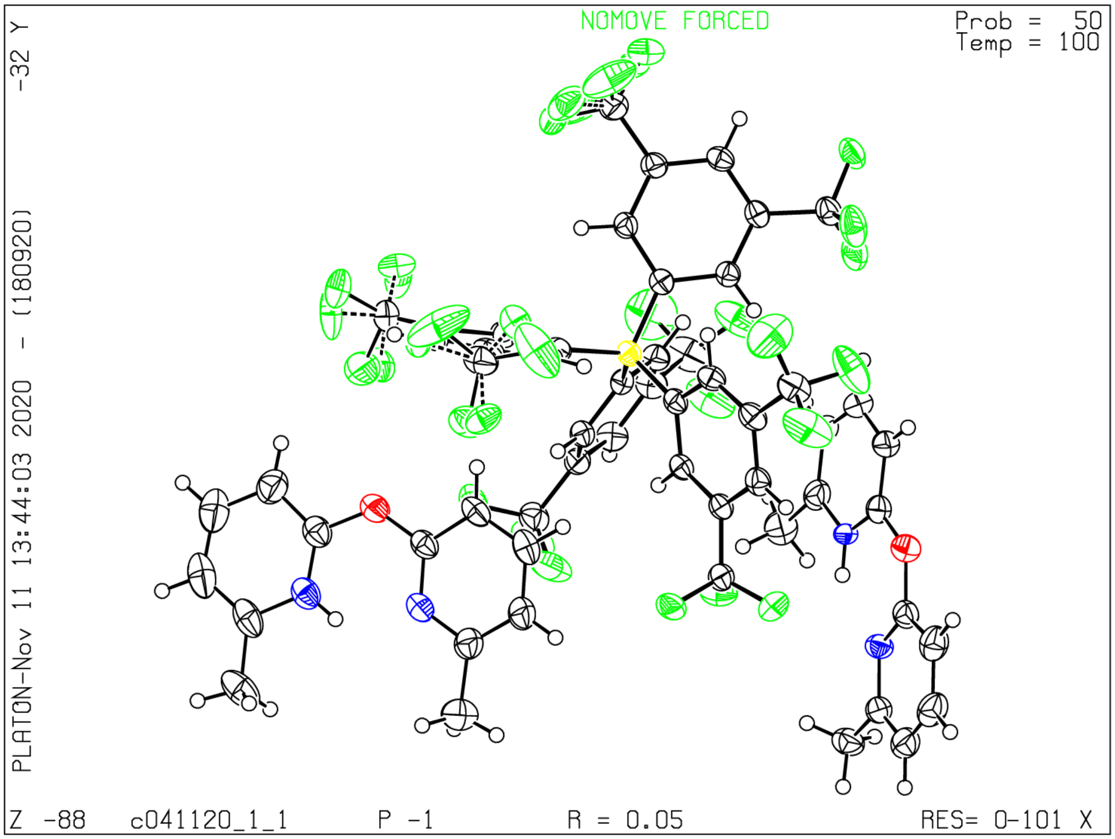

Supplement: Supplementary file 1 — Supporting Information [file CHEM-31-e02745-s002.zip › Crystal_structures/9b/checkcif.pdf]
