# Supplementary material for: London Dispersion versus Intramolecular Hydrogen Bond in Bis‐Pyridines: How Accurate Is DFT for Competing Noncovalent Interactions in the Condensed Phase?
Source: Chemistry. 2025 Oct 23;31(66):e02745. doi: 10.1002/chem.202502745 (PMC12648470; doi:10.1002/chem.202502745)

# checkCIF/PLATON report

Structure factors have been supplied for datablock(s) c280220

THIS REPORT IS FOR GUIDANCE ONLY. IF USED AS PART OF A REVIEW PROCEDURE FOR PUBLICATION, IT SHOULD NOT REPLACE THE EXPERTISE OF AN EXPERIENCED CRYSTALLOGRAPHIC REFEREE.

No syntax errors found. CIF dictionary Interpreting this report

## Datablock: c280220

---

```
Bond precision:  C-C = 0.0023 A          Wavelength=0.71073

Cell:            a=17.9819(17)     b=18.4321(18)       c=22.181(2)
                 alpha=90          beta=110.091(2)     gamma=90
Temperature:     100 K
```

| | Calculated | Reported |
|---|---|---|
| Volume | 6904.4(11) | 6904.5(12) |
| Space group | P 21/c | P 1 21/c 1 |
| Hall group | -P 2ybc | -P 2ybc |
| Moiety formula | C32 H12 B F24, C20 H28 N, C20 H27 N | C32 H12 B F23.999, C20 H28 N, C20 H27 N |
| Sum formula | C72 H67 B F24 N2 | C72 H67 B F24 N2 |
| Mr | 1427.09 | 1427.08 |
| Dx,g cm-3 | 1.373 | 1.373 |
| Z | 4 | 4 |
| Mu (mm-1) | 0.125 | 0.125 |
| F000 | 2936.0 | 2936.0 |
| F000’ | 2938.29 | |
| h,k,lmax | 26,26,32 | 25,26,31 |
| Nref | 22452 | 20844 |
| Tmin,Tmax | 0.933,0.936 | 0.556,0.746 |
| Tmin’ | 0.933 | |

```
Correction method= # Reported T Limits: Tmin=0.556 Tmax=0.746
AbsCorr = MULTI-SCAN

Data completeness= 0.928           Theta(max)= 31.232

R(reflections)= 0.0567( 14546)     wR2(reflections)= 0.1596( 20844)

S = 1.048                  Npar= 1087
```

---

The following ALERTS were generated. Each ALERT has the format
**test-name_ALERT_alert-type_alert-level**.
Click on the hyperlinks for more details of the test.

---

## Alert level B

```
PLAT201_ALERT_2_B Isotropic non-H Atoms in Main Residue(s) .......          2 Report
              C63     C72A
PLAT213_ALERT_2_B Atom F12             has ADP max/min Ratio .....        4.3 prolat
PLAT213_ALERT_2_B Atom F11A            has ADP max/min Ratio .....        4.2 prolat
PLAT353_ALERT_3_B Long   N-H (N0.87,N1.01A)  N1       - H1       .       1.11 Ang.
```

---

## Alert level C

```
PLAT213_ALERT_2_C Atom F8              has ADP max/min Ratio .....        3.7 prolat
PLAT213_ALERT_2_C Atom F22A            has ADP max/min Ratio .....        3.3 prolat
PLAT213_ALERT_2_C Atom F10A            has ADP max/min Ratio .....        3.7 prolat
PLAT213_ALERT_2_C Atom F14A            has ADP max/min Ratio .....        3.4 prolat
PLAT213_ALERT_2_C Atom F14B            has ADP max/min Ratio .....        3.1 prolat
PLAT213_ALERT_2_C Atom F18A            has ADP max/min Ratio .....        3.3 prolat
PLAT213_ALERT_2_C Atom F19A            has ADP max/min Ratio .....        3.4 prolat
PLAT213_ALERT_2_C Atom F20A            has ADP max/min Ratio .....        3.2 prolat
PLAT213_ALERT_2_C Atom F21             has ADP max/min Ratio .....        3.3 prolat
PLAT220_ALERT_2_C NonSolvent Resd 1  F   Ueq(max) / Ueq(min) Range        3.4 Ratio
PLAT906_ALERT_3_C Large K Value in the Analysis of Variance ......      2.562 Check
PLAT934_ALERT_3_C Number of (Iobs-Icalc)/Sigma(W) > 10 Outliers ..          1 Check
```

---

## Alert level G

```
PLAT002_ALERT_2_G Number of Distance or Angle Restraints on AtSite         36 Note
PLAT003_ALERT_2_G Number of Uiso or Uij Restrained non-H Atoms ...         14 Report
PLAT042_ALERT_1_G Calc. and Reported MoietyFormula Strings  Differ     Please Check
PLAT171_ALERT_4_G The CIF-Embedded .res File Contains EADP Records          4 Report
PLAT176_ALERT_4_G The CIF-Embedded .res File Contains SADI Records          9 Report
PLAT186_ALERT_4_G The CIF-Embedded .res File Contains ISOR Records          1 Report
PLAT187_ALERT_4_G The CIF-Embedded .res File Contains RIGU Records          4 Report
PLAT230_ALERT_2_G Hirshfeld Test Diff for    F3A      --C47      .        6.0 s.u.
PLAT230_ALERT_2_G Hirshfeld Test Diff for    F7       --C55      .       10.9 s.u.
PLAT230_ALERT_2_G Hirshfeld Test Diff for    F1       --C47      .        5.3 s.u.
PLAT242_ALERT_2_G Low    MainMol Ueq as Compared to Neighbors of        C47 Check
PLAT242_ALERT_2_G Low    MainMol Ueq as Compared to Neighbors of        C48 Check
PLAT242_ALERT_2_G Low    MainMol Ueq as Compared to Neighbors of        C55 Check
PLAT242_ALERT_2_G Low    MainMol Ueq as Compared to Neighbors of        C64 Check
PLAT242_ALERT_2_G Low    MainMol Ueq as Compared to Neighbors of        C71 Check
PLAT301_ALERT_3_G Main Residue  Disorder .............(Resd  1  )        37% Note
PLAT432_ALERT_2_G Short Inter X...Y Contact  F9       ..C1               2.92 Ang.
                                                      x,y,z  =     1_555 Check
PLAT432_ALERT_2_G Short Inter X...Y Contact  F9A      ..C21              2.71 Ang.
                                                      x,y,z  =     1_555 Check
PLAT720_ALERT_4_G Number of Unusual/Non-Standard Labels ..........          1 Note
PLAT860_ALERT_3_G Number of Least-Squares Restraints .............        286 Note
PLAT912_ALERT_4_G Missing # of FCF Reflections Above STh/L=  0.600       1556 Note
PLAT978_ALERT_2_G Number C-C Bonds with Positive Residual Density.          6 Info
```

---

0 **ALERT level A** = Most likely a serious problem - resolve or explain
4 **ALERT level B** = A potentially serious problem, consider carefully
12 **ALERT level C** = Check. Ensure it is not caused by an omission or oversight
22 **ALERT level G** = General information/check it is not something unexpected

1 ALERT type 1 CIF construction/syntax error, inconsistent or missing data

```
26 ALERT type 2 Indicator that the structure model may be wrong or deficient
 5 ALERT type 3 Indicator that the structure quality may be low
 6 ALERT type 4 Improvement, methodology, query or suggestion
 0 ALERT type 5 Informative message, check
```

---

It is advisable to attempt to resolve as many as possible of the alerts in all categories. Often the minor alerts point to easily fixed oversights, errors and omissions in your CIF or refinement strategy, so attention to these fine details can be worthwhile. In order to resolve some of the more serious problems it may be necessary to carry out additional measurements or structure refinements. However, the purpose of your study may justify the reported deviations and the more serious of these should normally be commented upon in the discussion or experimental section of a paper or in the "special_details" fields of the CIF. checkCIF was carefully designed to identify outliers and unusual parameters, but every test has its limitations and alerts that are not important in a particular case may appear. Conversely, the absence of alerts does not guarantee there are no aspects of the results needing attention. It is up to the individual to critically assess their own results and, if necessary, seek expert advice.

**Publication of your CIF in IUCr journals**

A basic structural check has been run on your CIF. These basic checks will be run on all CIFs submitted for publication in IUCr journals (*Acta Crystallographica*, *Journal of Applied Crystallography*, *Journal of Synchrotron Radiation*); however, if you intend to submit to *Acta Crystallographica Section C* or *E* or *IUCrData*, you should make sure that full publication checks are run on the final version of your CIF prior to submission.

**Publication of your CIF in other journals**

Please refer to the *Notes for Authors* of the relevant journal for any special instructions relating to CIF submission.

---

**PLATON version of 22/12/2019; check.def file version of 13/12/2019**

**Datablock c280220** - ellipsoid plot

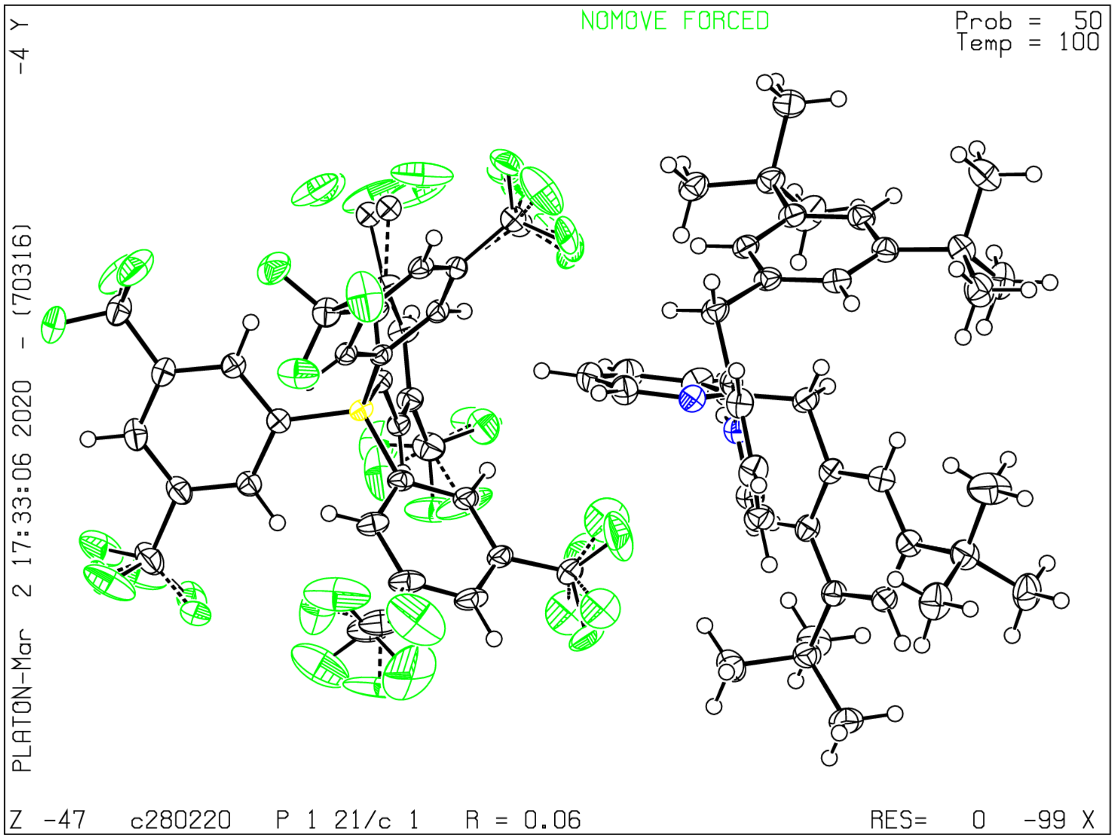

Supplement: Supplementary file 1 — Supporting Information [file CHEM-31-e02745-s002.zip › Crystal_structures/proton_bound_dimer_BHB/c280220_cifreport.pdf]
