# Supplementary material for: London Dispersion versus Intramolecular Hydrogen Bond in Bis‐Pyridines: How Accurate Is DFT for Competing Noncovalent Interactions in the Condensed Phase?
Source: Chemistry. 2025 Oct 23;31(66):e02745. doi: 10.1002/chem.202502745 (PMC12648470; doi:10.1002/chem.202502745)
Supplement: Supplementary file 1 — Supporting Information [file CHEM-31-e02745-s002.zip › Crystal_structures/proton_bound_dimer_BHB/c280220_tables.html]

c280220


# c280220

Table 1 Crystal data and structure refinement for c280220.

| Identification code | c280220 |
| Empirical formula | C72H67BF24N2 |
| Formula weight | 1427.08 |
| Temperature/K | 100.0 |
| Crystal system | monoclinic |
| Space group | P21/c |
| a/Å | 17.9819(17) |
| b/Å | 18.4321(18) |
| c/Å | 22.181(2) |
| α/° | 90 |
| β/° | 110.091(2) |
| γ/° | 90 |
| Volume/Å3 | 6904.5(12) |
| Z | 4 |
| ρcalcg/cm3 | 1.373 |
| μ/mm‑1 | 0.125 |
| F(000) | 2936.0 |
| Crystal size/mm3 | 0.559 × 0.541 × 0.525 |
| Radiation | MoKα (λ = 0.71073) |
| 2Θ range for data collection/° | 2.412 to 62.464 |
| Index ranges | -25 ≤ h ≤ 25, -26 ≤ k ≤ 25, -31 ≤ l ≤ 31 |
| Reflections collected | 91951 |
| Independent reflections | 20844 [Rint = 0.0489, Rsigma = 0.0512] |
| Data/restraints/parameters | 20844/286/1087 |
| Goodness-of-fit on F2 | 1.048 |
| Final R indexes [I>=2σ (I)] | R1 = 0.0567, wR2 = 0.1440 |
| Final R indexes [all data] | R1 = 0.0842, wR2 = 0.1596 |
| Largest diff. peak/hole / e Å-3 | 0.45/-0.41 |

Table 2 Fractional Atomic Coordinates (×104) and Equivalent Isotropic Displacement Parameters (Å2×103) for c280220. Ueq is defined as 1/3 of of the trace of the orthogonalised UIJ tensor.

| Atom | *x* | *y* | *z* | U(eq) |
| --- | --- | --- | --- | --- |
| F1 | 1110(3) | 5407(5) | 1731(2) | 59.2(18) |
| F1A | 1203(2) | 4926(7) | 1684(2) | 86(3) |
| F2 | 289(4) | 5439(5) | 822(3) | 67(2) |
| F2A | 356(4) | 5530(3) | 912(3) | 63.8(17) |
| F3 | 848(6) | 4443(3) | 1186(7) | 87(3) |
| F3A | 587(3) | 4443(2) | 793(3) | 58.6(13) |
| F4 | 1142.0(8) | 5320.7(12) | -1111.1(6) | 81.5(6) |
| F5 | 2221.0(9) | 5859.9(7) | -986.8(5) | 55.2(3) |
| F005 | 4071.5(8) | 8925.1(6) | 353.4(7) | 59.1(4) |
| F6 | 2225.2(11) | 4727.6(8) | -864.9(6) | 74.1(5) |
| F7 | 5397.5(9) | 5636.6(10) | 3990.9(6) | 57.9(5) |
| F7A | 4937(7) | 5869(6) | 4263(6) | 79(4) |
| F8 | 4936.6(10) | 6410.9(16) | 4472.1(7) | 91.8(10) |
| F8A | 5155(6) | 6994(5) | 4299(5) | 63(3) |
| F9 | 5753.0(9) | 6733.5(12) | 3997.8(10) | 73.8(6) |
| F9A | 5771(7) | 6292(7) | 3878(7) | 79(4) |
| F10 | 2353(3) | 8008(2) | 2216(2) | 90(2) |
| F10A | 2127(4) | 7742(5) | 2206(3) | 115(4) |
| F11 | 1903(2) | 7234.2(19) | 2670(3) | 91.1(16) |
| F11A | 2365(5) | 7560(4) | 3177(4) | 99(3) |
| F12 | 2701(3) | 8003(3) | 3224(2) | 127(3) |
| F12A | 3003(3) | 8323.2(19) | 2885(4) | 91(2) |
| F13 | 6558(3) | 6447.1(18) | 1179(6) | 63(2) |
| F13A | 6662(5) | 6528(5) | 1602(10) | 46(3) |
| F13B | 6694(6) | 6795(13) | 1825(3) | 63(6) |
| F14 | 6696(4) | 7444(6) | 1697(3) | 72(3) |
| F14A | 6661(10) | 7656(5) | 1557(7) | 37(3) |
| F14B | 6483(10) | 6461(9) | 883(7) | 55(5) |
| F15 | 6446(2) | 7453(4) | 704(2) | 45.4(12) |
| F15A | 6464(5) | 7059(12) | 702(5) | 54(4) |
| F15B | 6643(6) | 7571(5) | 1111(13) | 74(5) |
| F16 | 3370.3(7) | 8172.5(7) | -352.2(5) | 46.6(3) |
| F17 | 3042.7(7) | 8437.2(6) | 457.0(6) | 44.7(3) |
| F18 | 4361(6) | 3625(5) | 2991(5) | 67(2) |
| F18A | 4691(14) | 3781(6) | 3143(5) | 69(3) |
| F19 | 5375(3) | 3122(4) | 2876(4) | 48.6(13) |
| F19A | 5337(7) | 3055(8) | 2807(8) | 62(3) |
| F20 | 4239(4) | 2780(3) | 2311(3) | 63.6(13) |
| F20A | 4075(8) | 2998(13) | 2475(9) | 84(5) |
| F21 | 5855(8) | 3491(4) | 839(4) | 72.7(10) |
| F21A | 5878(2) | 4359(3) | 630(3) | 73.2(11) |
| F22 | 4845(8) | 3935(8) | 109(6) | 70(4) |
| F22A | 5401(2) | 3291.7(12) | 592.8(13) | 72.7(10) |
| F23 | 5865(10) | 4580(6) | 605(10) | 74(5) |
| F23A | 4719(3) | 4150.0(17) | 17.0(17) | 59.3(8) |
| C41 | 2938.0(8) | 5725.8(7) | 1024.2(7) | 20.3(3) |
| C42 | 2326.2(9) | 5523.6(8) | 1250.7(7) | 23.9(3) |
| C43 | 1594.8(9) | 5289.1(9) | 840.7(8) | 29.1(3) |
| C44 | 1438.2(10) | 5231.0(9) | 184.5(8) | 31.5(3) |
| C45 | 2030.4(10) | 5427.3(8) | -51.9(7) | 26.5(3) |
| C46 | 2759.7(9) | 5674.9(8) | 357.7(7) | 22.5(3) |
| C47 | 954.0(11) | 5093.2(12) | 1100.5(10) | 41.5(4) |
| C48 | 1896.3(11) | 5337.7(10) | -752.4(8) | 33.7(4) |
| C49 | 3832.8(9) | 6254.6(8) | 2185.2(7) | 22.4(3) |
| C50 | 4433.0(10) | 6135.3(9) | 2774.0(7) | 27.2(3) |
| C51 | 4445.1(10) | 6479.8(10) | 3338.4(8) | 32.0(4) |
| C52 | 3857.9(11) | 6963.3(9) | 3337.0(8) | 34.2(4) |
| C53 | 3262.5(11) | 7102.6(9) | 2757.3(9) | 33.1(4) |
| C54 | 3251.0(11) | 6757.0(8) | 2195.1(8) | 28.1(3) |
| C55 | 5114.8(12) | 6329.0(14) | 3947.6(9) | 48.6(6) |
| C56 | 2558(6) | 7592(5) | 2727(4) | 45.5(12) |
| C56A | 2707(9) | 7670(8) | 2754(6) | 45.5(12) |
| C57 | 4247.7(8) | 6531.6(7) | 1200.2(6) | 18.6(3) |
| C58 | 5063.6(9) | 6546.0(8) | 1310.5(7) | 21.5(3) |
| C59 | 5411.4(9) | 7103.4(8) | 1078.5(7) | 23.1(3) |
| C60 | 4970.4(9) | 7682.7(8) | 742.6(7) | 23.4(3) |
| C61 | 4166.8(9) | 7687.4(8) | 641.7(7) | 21.8(3) |
| C62 | 3814.2(9) | 7121.6(7) | 858.8(6) | 20.5(3) |
| C63 | 6258(2) | 7118(2) | 1158(2) | 25.6(5) |
| C63A | 6302(6) | 7073(6) | 1241(6) | 25.6(5) |
| C63B | 6308(6) | 6958(6) | 1224(5) | 25.6(5) |
| C64 | 3672.4(10) | 8305.5(9) | 280.5(8) | 30.0(3) |
| C65 | 4291.9(8) | 5126.0(7) | 1578.3(6) | 19.5(3) |
| C66 | 4313.1(9) | 4612.8(8) | 2051.4(7) | 21.7(3) |
| C67 | 4648.5(9) | 3931.2(8) | 2070.3(7) | 24.5(3) |
| C68 | 4970.4(10) | 3719.2(8) | 1609.7(8) | 28.6(3) |
| C69 | 4940.2(11) | 4209.6(8) | 1129.3(7) | 28.6(3) |
| C70 | 4613.2(9) | 4896.3(8) | 1115.2(7) | 24.1(3) |
| C71 | 4668.0(11) | 3397.9(10) | 2584.1(9) | 34.7(4) |
| C72 | 5407(5) | 4061(3) | 668(2) | 24.4(4) |
| C72A | 5174(3) | 3974.3(19) | 580.8(19) | 24.4(4) |
| B1 | 3833.6(10) | 5909.3(8) | 1505.3(7) | 19.0(3) |
| N1 | 7457.9(8) | 7330.5(7) | 4049.0(6) | 24.8(3) |
| C1 | 7233.2(10) | 7539.6(9) | 4543.3(8) | 29.0(3) |
| C2 | 7120.8(10) | 8265.5(10) | 4639.7(8) | 32.9(4) |
| C3 | 7240.4(11) | 8766.4(9) | 4222.7(8) | 32.7(4) |
| C4 | 7481.9(10) | 8542.4(8) | 3724.1(8) | 28.7(3) |
| C5 | 7593.6(9) | 7810.6(8) | 3639.4(7) | 23.7(3) |
| C6 | 7837.7(10) | 7511.9(9) | 3106.5(8) | 29.7(3) |
| C7 | 8606.1(9) | 7080.0(9) | 3333.3(7) | 25.9(3) |
| C8 | 8632.6(10) | 6397.2(9) | 3071.7(7) | 26.0(3) |
| C9 | 9331.9(10) | 5994.3(9) | 3255.9(7) | 26.4(3) |
| C10 | 10003.0(10) | 6305.7(9) | 3705.3(8) | 27.6(3) |
| C11 | 9994.4(9) | 6989.0(9) | 3979.8(7) | 26.8(3) |
| C12 | 9280.8(10) | 7368.8(8) | 3786.1(7) | 26.9(3) |
| C13 | 9386.3(10) | 5236.3(9) | 2988.2(8) | 30.2(3) |
| C14 | 8585.9(11) | 4969.2(10) | 2530.6(8) | 34.0(4) |
| C15 | 9974.4(12) | 5254.7(11) | 2621.5(10) | 41.3(4) |
| C16 | 9682.6(13) | 4695.7(10) | 3548.6(9) | 41.2(4) |
| C17 | 10757.5(10) | 7288.2(9) | 4467.2(8) | 30.9(3) |
| C18 | 11373.0(11) | 7388.0(11) | 4136.2(10) | 42.7(4) |
| C19 | 11072.1(12) | 6748.3(11) | 5026.1(9) | 40.3(4) |
| C20 | 10633.4(12) | 8019.3(10) | 4744.9(10) | 39.6(4) |
| N2 | 7343.5(8) | 5928.7(7) | 3745.8(6) | 26.5(3) |
| C21 | 6698.8(10) | 5775.3(9) | 3238.6(8) | 31.9(3) |
| C22 | 6494.2(11) | 5079.1(10) | 3016.3(9) | 34.8(4) |
| C23 | 6972.1(12) | 4516.0(9) | 3341.3(9) | 36.4(4) |
| C24 | 7643.0(11) | 4670.9(9) | 3861.6(8) | 31.7(3) |
| C25 | 7825.1(10) | 5386.9(8) | 4054.4(7) | 25.4(3) |
| C26 | 8562.8(10) | 5603.2(9) | 4597.2(7) | 27.7(3) |
| C27 | 8419.1(9) | 5894.7(8) | 5189.8(7) | 23.8(3) |
| C28 | 8824.1(9) | 6510.3(8) | 5503.5(7) | 25.4(3) |
| C29 | 8746.8(9) | 6757.5(8) | 6074.0(8) | 25.9(3) |
| C30 | 8244.8(9) | 6370.8(8) | 6318.6(7) | 26.0(3) |
| C31 | 7830.1(9) | 5755.8(8) | 6019.1(7) | 24.9(3) |
| C32 | 7922.1(9) | 5528.7(8) | 5445.5(7) | 25.2(3) |
| C33 | 9179.6(11) | 7431.3(9) | 6438.6(9) | 33.1(4) |
| C34 | 8559.1(13) | 7998.5(10) | 6449.2(10) | 43.3(4) |
| C35 | 9661.5(12) | 7234.2(11) | 7142.7(10) | 43.1(4) |
| C36 | 9739.6(16) | 7753.4(13) | 6137.2(13) | 62.0(7) |
| C37 | 7280.0(10) | 5333.7(9) | 6289.4(8) | 30.0(3) |
| C38 | 7310.7(13) | 5616.8(12) | 6946.7(9) | 42.5(4) |
| C39 | 7527.0(12) | 4529.2(10) | 6371.4(10) | 39.1(4) |
| C40 | 6425.8(11) | 5398.8(11) | 5818.4(10) | 40.6(4) |

Table 3 Anisotropic Displacement Parameters (Å2×103) for c280220. The Anisotropic displacement factor exponent takes the form: -2π2[h2a\*2U11+2hka\*b\*U12+…].

| Atom | U11 | U22 | U33 | U23 | U13 | U12 |
| --- | --- | --- | --- | --- | --- | --- |
| F1 | 34.0(17) | 114(5) | 36.9(16) | -11(2) | 21.2(14) | -20(2) |
| F1A | 37.0(16) | 181(7) | 38.4(17) | 34(3) | 9.1(12) | -33(3) |
| F2 | 18(2) | 139(6) | 38(2) | 9(3) | -0.1(17) | 3(3) |
| F2A | 58(3) | 32.7(17) | 127(5) | 6.1(19) | 66(3) | 7.7(14) |
| F3 | 99(5) | 45(2) | 159(8) | 6(4) | 99(6) | -11(3) |
| F3A | 54(2) | 42.7(14) | 96(3) | -8.4(18) | 47(2) | -22.7(13) |
| F4 | 39.8(7) | 165.2(18) | 28.9(6) | -14.4(8) | -1.7(5) | -21.1(9) |
| F5 | 79.4(9) | 57.0(7) | 26.6(5) | -0.8(5) | 14.7(6) | -23.1(7) |
| F005 | 56.1(8) | 25.8(5) | 81.4(9) | 20.1(6) | 5.8(7) | -7.9(5) |
| F6 | 133.8(14) | 48.7(8) | 38.0(7) | -5.9(6) | 27.3(8) | 24.8(8) |
| F7 | 57.1(10) | 77.3(12) | 26.4(7) | -1.6(7) | -2.1(6) | 4.6(8) |
| F8 | 52.8(10) | 200(3) | 21.1(7) | -23.6(10) | 10.5(7) | 30.6(13) |
| F9 | 34.0(9) | 81.7(14) | 91.1(14) | -19.6(11) | 3.0(8) | -23.4(8) |
| F10 | 133(4) | 67(2) | 108(4) | 55(3) | 91(3) | 59(2) |
| F10A | 71(4) | 157(7) | 88(5) | -77(5) | -11(3) | 68(4) |
| F11 | 72(2) | 70(2) | 157(4) | 20(2) | 72(3) | 27.9(16) |
| F11A | 142(6) | 88(4) | 129(6) | 49(4) | 125(5) | 65(4) |
| F12 | 132(4) | 131(4) | 105(3) | -81(3) | 25(3) | 54(4) |
| F12A | 96(4) | 25.6(17) | 170(6) | -9(2) | 69(4) | 10.5(18) |
| F13 | 33(2) | 34.5(15) | 133(8) | 12(3) | 43(4) | 8.1(12) |
| F13A | 22(3) | 35(4) | 84(9) | 12(5) | 22(6) | 3(3) |
| F13B | 26(4) | 134(17) | 24(4) | -19(6) | 2(3) | 10(7) |
| F14 | 24.2(15) | 142(7) | 44(2) | -43(4) | 6.7(16) | -16(4) |
| F14A | 22(3) | 25(3) | 56(8) | -22(3) | 4(5) | -13(2) |
| F14B | 31(4) | 94(11) | 49(6) | -33(7) | 23(5) | 6(5) |
| F15 | 28.6(13) | 70(3) | 45.3(19) | 21.0(18) | 22.2(12) | 3.8(15) |
| F15A | 36(3) | 98(10) | 36(4) | -20(5) | 21(3) | -11(5) |
| F15B | 44(5) | 53(5) | 127(12) | 12(7) | 35(7) | -21(4) |
| F16 | 49.8(7) | 60.0(7) | 26.0(5) | 11.3(5) | 8.2(5) | 16.6(6) |
| F17 | 54.5(7) | 36.1(6) | 52.6(7) | 13.5(5) | 30.0(6) | 20.4(5) |
| F18 | 102(4) | 59(3) | 72(3) | 42(2) | 70(3) | 44(2) |
| F18A | 140(9) | 48(3) | 35(3) | 21(2) | 52(4) | 28(4) |
| F19 | 38.5(19) | 66(3) | 36.1(17) | 30.8(16) | 6.3(16) | 5.7(19) |
| F19A | 71(5) | 69(5) | 64(6) | 45(4) | 45(5) | 57(5) |
| F20 | 78(3) | 44.3(18) | 60(2) | 21.6(14) | 12.4(17) | -20.7(15) |
| F20A | 54(4) | 105(9) | 69(6) | 61(6) | -10(4) | -38(5) |
| F21 | 157(3) | 34.8(10) | 45.4(13) | 11.0(9) | 60.1(17) | 45.4(14) |
| F21A | 72.6(18) | 93(3) | 78(2) | -23(2) | 57.0(16) | -5.6(18) |
| F22 | 82(6) | 92(8) | 37(6) | -15(5) | 23(4) | 24(6) |
| F22A | 157(3) | 34.8(10) | 45.4(13) | 11.0(9) | 60.1(17) | 45.4(14) |
| F23 | 122(10) | 43(5) | 102(8) | 6(5) | 98(8) | 5(5) |
| F23A | 101(2) | 57.9(14) | 23.1(10) | 6.1(9) | 26.2(10) | 32.8(13) |
| C41 | 22.5(7) | 15.3(6) | 23.2(7) | 0.3(5) | 7.9(5) | 0.1(5) |
| C42 | 23.6(7) | 23.4(7) | 25.1(7) | 2.9(5) | 8.8(6) | 0.1(5) |
| C43 | 21.7(7) | 31.6(8) | 33.9(8) | 6.9(6) | 9.1(6) | -1.0(6) |
| C44 | 24.3(8) | 31.7(8) | 32.5(8) | 3.0(6) | 2.1(6) | -4.4(6) |
| C45 | 28.5(8) | 24.6(7) | 22.4(7) | -0.7(6) | 3.7(6) | -1.8(6) |
| C46 | 25.3(7) | 18.6(6) | 23.5(7) | -1.3(5) | 8.3(6) | -2.1(5) |
| C47 | 25.3(9) | 52.6(11) | 45.8(11) | 11.9(9) | 11.5(8) | -4.8(8) |
| C48 | 34.2(9) | 35.2(9) | 26.5(8) | -3.3(7) | 3.7(7) | -4.8(7) |
| C49 | 27.2(7) | 20.5(6) | 24.1(7) | -3.9(5) | 14.5(6) | -5.8(5) |
| C50 | 26.7(8) | 33.7(8) | 24.1(7) | -9.1(6) | 12.5(6) | -9.2(6) |
| C51 | 31.2(8) | 44.7(9) | 23.9(7) | -14.0(7) | 14.2(7) | -16.3(7) |
| C52 | 48.6(10) | 31.9(8) | 31.8(8) | -14.4(7) | 26.4(8) | -16.5(7) |
| C53 | 50.7(10) | 21.6(7) | 38.5(9) | -4.8(6) | 29.9(8) | -2.0(7) |
| C54 | 41.0(9) | 20.6(7) | 28.5(8) | -0.7(6) | 19.3(7) | 0.5(6) |
| C55 | 33.8(10) | 85.7(17) | 28.2(9) | -26.3(10) | 13.1(8) | -18.2(10) |
| C56 | 70(4) | 33(2) | 49.4(14) | -1.5(12) | 41(2) | 9.0(18) |
| C56A | 70(4) | 33(2) | 49.4(14) | -1.5(12) | 41(2) | 9.0(18) |
| C57 | 21.7(7) | 19.1(6) | 15.5(6) | -3.0(5) | 7.0(5) | -1.6(5) |
| C58 | 22.3(7) | 22.6(6) | 19.4(6) | -2.5(5) | 6.6(5) | -0.1(5) |
| C59 | 20.6(7) | 28.2(7) | 21.1(7) | -6.7(5) | 8.1(5) | -5.2(5) |
| C60 | 29.4(8) | 22.9(7) | 20.0(6) | -3.4(5) | 11.1(6) | -7.7(6) |
| C61 | 26.9(7) | 19.8(6) | 18.0(6) | -1.0(5) | 7.0(6) | -3.5(5) |
| C62 | 21.7(7) | 21.0(6) | 18.4(6) | -1.6(5) | 6.3(5) | -1.9(5) |
| C64 | 33.4(9) | 25.7(7) | 29.7(8) | 6.1(6) | 9.3(7) | -1.5(6) |
| C65 | 21.7(7) | 19.9(6) | 16.0(6) | -0.7(5) | 5.2(5) | -1.5(5) |
| C66 | 22.1(7) | 23.6(7) | 19.0(6) | 2.3(5) | 6.7(5) | -0.4(5) |
| C67 | 27.4(8) | 25.2(7) | 21.7(7) | 7.0(5) | 9.4(6) | 2.5(6) |
| C68 | 39.0(9) | 21.1(7) | 27.6(7) | 5.1(6) | 13.8(7) | 7.9(6) |
| C69 | 43.1(9) | 23.7(7) | 22.4(7) | 3.1(6) | 15.5(7) | 8.4(6) |
| C70 | 35.5(8) | 19.7(6) | 18.4(6) | 2.1(5) | 11.1(6) | 4.4(6) |
| C71 | 36.4(9) | 35.4(9) | 34.6(9) | 16.2(7) | 15.3(7) | 8.8(7) |
| B1 | 23.3(8) | 17.8(7) | 16.9(7) | -0.9(5) | 8.1(6) | -0.8(6) |
| N1 | 23.8(6) | 21.7(6) | 26.8(6) | 0.6(5) | 6.2(5) | 2.7(5) |
| C1 | 29.7(8) | 28.2(7) | 29.9(8) | 2.1(6) | 11.2(7) | 3.0(6) |
| C2 | 34.1(9) | 35.1(9) | 30.6(8) | -1.8(7) | 12.3(7) | 8.3(7) |
| C3 | 38.0(9) | 23.6(7) | 32.7(8) | -2.7(6) | 7.2(7) | 7.3(6) |
| C4 | 30.0(8) | 22.9(7) | 30.4(8) | 2.6(6) | 6.8(6) | 2.6(6) |
| C5 | 20.5(7) | 23.8(7) | 23.9(7) | 0.9(5) | 4.0(6) | 3.2(5) |
| C6 | 31.5(8) | 33.5(8) | 22.8(7) | 2.4(6) | 7.6(6) | 10.5(7) |
| C7 | 28.5(8) | 28.8(7) | 22.0(7) | 4.4(6) | 10.6(6) | 7.1(6) |
| C8 | 28.3(8) | 30.3(8) | 20.0(7) | 1.4(6) | 9.3(6) | 4.3(6) |
| C9 | 29.8(8) | 28.9(7) | 22.1(7) | 1.9(6) | 11.0(6) | 4.9(6) |
| C10 | 26.5(8) | 29.8(8) | 27.8(7) | 1.8(6) | 11.0(6) | 6.1(6) |
| C11 | 26.7(8) | 28.1(7) | 27.0(7) | 2.0(6) | 11.0(6) | 0.5(6) |
| C12 | 31.4(8) | 25.5(7) | 26.2(7) | 2.3(6) | 13.2(6) | 3.8(6) |
| C13 | 32.8(9) | 31.4(8) | 26.4(7) | -2.1(6) | 10.3(7) | 8.3(6) |
| C14 | 39.1(9) | 33.0(8) | 29.8(8) | -4.3(7) | 11.7(7) | 3.3(7) |
| C15 | 37.4(10) | 49.6(11) | 40.2(10) | -11.3(8) | 17.3(8) | 8.3(8) |
| C16 | 56.5(12) | 28.5(8) | 32.8(9) | -0.6(7) | 7.8(8) | 13.1(8) |
| C17 | 27.4(8) | 30.2(8) | 34.8(8) | 0.7(6) | 10.3(7) | 0.7(6) |
| C18 | 31.9(10) | 46.5(11) | 52.8(12) | -0.4(9) | 18.5(9) | -3.0(8) |
| C19 | 37.6(10) | 38.2(9) | 36.1(9) | 2.9(7) | 1.1(8) | -1.9(8) |
| C20 | 35.9(10) | 34.5(9) | 45.9(10) | -9.8(8) | 10.9(8) | -3.7(7) |
| N2 | 28.9(7) | 22.6(6) | 27.8(6) | -1.1(5) | 9.4(5) | -0.6(5) |
| C21 | 29.6(8) | 31.7(8) | 32.6(8) | -1.4(6) | 8.5(7) | 0.7(6) |
| C22 | 30.7(9) | 38.5(9) | 33.9(9) | -9.2(7) | 9.5(7) | -6.8(7) |
| C23 | 46.1(10) | 26.3(8) | 39.5(9) | -9.0(7) | 18.0(8) | -8.1(7) |
| C24 | 43.7(10) | 21.7(7) | 31.1(8) | 0.8(6) | 14.6(7) | 2.4(7) |
| C25 | 32.5(8) | 23.2(7) | 23.0(7) | 0.0(5) | 12.5(6) | -1.0(6) |
| C26 | 30.4(8) | 30.3(8) | 22.2(7) | -1.7(6) | 9.0(6) | 2.1(6) |
| C27 | 26.2(7) | 22.8(7) | 20.9(7) | -0.4(5) | 6.3(6) | 2.1(6) |
| C28 | 26.9(8) | 23.0(7) | 27.1(7) | 1.1(6) | 10.3(6) | 0.8(6) |
| C29 | 26.9(8) | 21.1(7) | 29.7(8) | -3.7(6) | 9.6(6) | -0.9(6) |
| C30 | 26.7(8) | 25.4(7) | 26.7(7) | -5.3(6) | 10.2(6) | -1.1(6) |
| C31 | 26.2(7) | 23.3(7) | 25.3(7) | -2.3(5) | 8.7(6) | -1.8(6) |
| C32 | 27.8(8) | 22.0(7) | 24.4(7) | -2.9(5) | 7.1(6) | -2.5(6) |
| C33 | 33.4(9) | 26.4(8) | 40.2(9) | -10.5(7) | 13.6(7) | -7.4(6) |
| C34 | 48.6(11) | 28.8(9) | 47.6(11) | -10.2(8) | 10.4(9) | 0.7(8) |
| C35 | 39.9(10) | 39.3(10) | 42.6(10) | -13.7(8) | 4.6(8) | -3.9(8) |
| C36 | 78.5(17) | 53.9(13) | 72.4(15) | -30.8(12) | 50.0(14) | -39.8(12) |
| C37 | 31.1(8) | 31.0(8) | 30.5(8) | -4.6(6) | 14.0(7) | -6.7(6) |
| C38 | 48.3(11) | 50.4(11) | 36.8(9) | -8.7(8) | 24.9(9) | -14.3(9) |
| C39 | 44.5(11) | 29.9(8) | 46.6(10) | 0.9(7) | 20.3(9) | -6.5(7) |
| C40 | 29.1(9) | 46.1(10) | 46.5(11) | -3.6(8) | 12.9(8) | -6.0(8) |

Table 4 Bond Lengths for c280220.

| Atom | Atom | Length/Å |  | Atom | Atom | Length/Å |
| --- | --- | --- | --- | --- | --- | --- |
| F1 | C47 | 1.450(5) |  | C57 | C62 | 1.399(2) |
| F1A | C47 | 1.254(4) |  | C57 | B1 | 1.635(2) |
| F2 | C47 | 1.307(7) |  | C58 | C59 | 1.390(2) |
| F2A | C47 | 1.293(5) |  | C59 | C60 | 1.385(2) |
| F3 | C47 | 1.238(5) |  | C59 | C63 | 1.472(4) |
| F3A | C47 | 1.424(5) |  | C59 | C63A | 1.517(10) |
| F4 | C48 | 1.316(2) |  | C59 | C63B | 1.555(10) |
| F5 | C48 | 1.322(2) |  | C60 | C61 | 1.384(2) |
| F005 | C64 | 1.3288(19) |  | C61 | C62 | 1.3897(19) |
| F6 | C48 | 1.333(2) |  | C61 | C64 | 1.497(2) |
| F7 | C55 | 1.365(3) |  | C65 | C66 | 1.4036(19) |
| F7A | C55 | 1.211(9) |  | C65 | C70 | 1.4057(19) |
| F8 | C55 | 1.317(2) |  | C65 | B1 | 1.643(2) |
| F8A | C55 | 1.441(8) |  | C66 | C67 | 1.388(2) |
| F9 | C55 | 1.341(3) |  | C67 | C68 | 1.392(2) |
| F9A | C55 | 1.244(9) |  | C67 | C71 | 1.496(2) |
| F10 | C56 | 1.312(8) |  | C68 | C69 | 1.384(2) |
| F10A | C56A | 1.308(12) |  | C69 | C70 | 1.392(2) |
| F11 | C56 | 1.317(8) |  | C69 | C72 | 1.556(5) |
| F11A | C56A | 1.302(12) |  | C69 | C72A | 1.482(3) |
| F12 | C56 | 1.290(8) |  | N1 | C1 | 1.349(2) |
| F12A | C56A | 1.307(12) |  | N1 | C5 | 1.349(2) |
| F13 | C63 | 1.344(5) |  | C1 | C2 | 1.381(2) |
| F13A | C63A | 1.308(11) |  | C2 | C3 | 1.375(2) |
| F13B | C63B | 1.311(11) |  | C3 | C4 | 1.383(2) |
| F14 | C63 | 1.327(5) |  | C4 | C5 | 1.386(2) |
| F14A | C63A | 1.323(11) |  | C5 | C6 | 1.500(2) |
| F14B | C63B | 1.294(11) |  | C6 | C7 | 1.523(2) |
| F15 | C63 | 1.321(4) |  | C7 | C8 | 1.394(2) |
| F15A | C63A | 1.326(11) |  | C7 | C12 | 1.388(2) |
| F15B | C63B | 1.342(10) |  | C8 | C9 | 1.395(2) |
| F16 | C64 | 1.342(2) |  | C9 | C10 | 1.398(2) |
| F17 | C64 | 1.341(2) |  | C9 | C13 | 1.534(2) |
| F18 | C71 | 1.280(6) |  | C10 | C11 | 1.401(2) |
| F18A | C71 | 1.415(9) |  | C11 | C12 | 1.394(2) |
| F19 | C71 | 1.315(6) |  | C11 | C17 | 1.529(2) |
| F19A | C71 | 1.296(10) |  | C13 | C14 | 1.530(3) |
| F20 | C71 | 1.393(5) |  | C13 | C15 | 1.540(2) |
| F20A | C71 | 1.250(9) |  | C13 | C16 | 1.538(2) |
| F21 | C72 | 1.299(7) |  | C17 | C18 | 1.535(2) |
| F21A | C72A | 1.422(7) |  | C17 | C19 | 1.538(2) |
| F22 | C72 | 1.323(10) |  | C17 | C20 | 1.530(2) |
| F22A | C72A | 1.320(3) |  | N2 | C21 | 1.339(2) |
| F23 | C72 | 1.300(11) |  | N2 | C25 | 1.345(2) |
| F23A | C72A | 1.279(5) |  | C21 | C22 | 1.379(2) |
| C41 | C42 | 1.408(2) |  | C22 | C23 | 1.382(3) |
| C41 | C46 | 1.404(2) |  | C23 | C24 | 1.383(3) |
| C41 | B1 | 1.633(2) |  | C24 | C25 | 1.391(2) |
| C42 | C43 | 1.386(2) |  | C25 | C26 | 1.507(2) |
| C43 | C44 | 1.388(2) |  | C26 | C27 | 1.522(2) |
| C43 | C47 | 1.499(2) |  | C27 | C28 | 1.398(2) |
| C44 | C45 | 1.387(2) |  | C27 | C32 | 1.387(2) |
| C45 | C46 | 1.391(2) |  | C28 | C29 | 1.396(2) |
| C45 | C48 | 1.497(2) |  | C29 | C30 | 1.398(2) |
| C49 | C50 | 1.396(2) |  | C29 | C33 | 1.539(2) |
| C49 | C54 | 1.403(2) |  | C30 | C31 | 1.394(2) |
| C49 | B1 | 1.637(2) |  | C31 | C32 | 1.402(2) |
| C50 | C51 | 1.397(2) |  | C31 | C37 | 1.533(2) |
| C51 | C52 | 1.381(3) |  | C33 | C34 | 1.535(3) |
| C51 | C55 | 1.495(3) |  | C33 | C35 | 1.547(3) |
| C52 | C53 | 1.385(3) |  | C33 | C36 | 1.509(3) |
| C53 | C54 | 1.394(2) |  | C37 | C38 | 1.531(2) |
| C53 | C56 | 1.538(11) |  | C37 | C39 | 1.541(2) |
| C53 | C56A | 1.445(18) |  | C37 | C40 | 1.537(3) |
| C57 | C58 | 1.402(2) |  |  |  |  |

Table 5 Bond Angles for c280220.

| Atom | Atom | Atom | Angle/˚ |  | Atom | Atom | Atom | Angle/˚ |
| --- | --- | --- | --- | --- | --- | --- | --- | --- |
| C42 | C41 | B1 | 122.54(12) |  | C66 | C65 | B1 | 123.37(12) |
| C46 | C41 | C42 | 115.75(13) |  | C70 | C65 | B1 | 120.72(12) |
| C46 | C41 | B1 | 121.27(12) |  | C67 | C66 | C65 | 122.18(13) |
| C43 | C42 | C41 | 122.00(14) |  | C66 | C67 | C68 | 121.19(13) |
| C42 | C43 | C44 | 121.13(14) |  | C66 | C67 | C71 | 120.92(14) |
| C42 | C43 | C47 | 120.27(15) |  | C68 | C67 | C71 | 117.88(14) |
| C44 | C43 | C47 | 118.59(15) |  | C69 | C68 | C67 | 117.74(14) |
| C45 | C44 | C43 | 118.07(15) |  | C68 | C69 | C70 | 121.06(14) |
| C44 | C45 | C46 | 120.84(14) |  | C68 | C69 | C72 | 120.32(19) |
| C44 | C45 | C48 | 119.51(15) |  | C68 | C69 | C72A | 119.59(17) |
| C46 | C45 | C48 | 119.58(14) |  | C70 | C69 | C72 | 117.68(19) |
| C45 | C46 | C41 | 122.19(14) |  | C70 | C69 | C72A | 118.98(16) |
| F1 | C47 | C43 | 110.8(2) |  | C69 | C70 | C65 | 122.28(13) |
| F1A | C47 | F2A | 116.7(5) |  | F18 | C71 | F19 | 110.9(5) |
| F1A | C47 | F3A | 103.3(4) |  | F18 | C71 | F20 | 105.4(3) |
| F1A | C47 | C43 | 114.0(2) |  | F18 | C71 | C67 | 114.8(3) |
| F2 | C47 | F1 | 96.0(4) |  | F18A | C71 | C67 | 109.0(5) |
| F2 | C47 | C43 | 113.6(4) |  | F19 | C71 | F20 | 101.3(4) |
| F2A | C47 | F3A | 99.9(3) |  | F19 | C71 | C67 | 113.3(3) |
| F2A | C47 | C43 | 112.9(3) |  | F19A | C71 | F18A | 99.5(8) |
| F3 | C47 | F1 | 103.1(4) |  | F19A | C71 | C67 | 112.8(6) |
| F3 | C47 | F2 | 112.3(6) |  | F20 | C71 | C67 | 110.0(3) |
| F3 | C47 | C43 | 118.2(3) |  | F20A | C71 | F18A | 103.3(7) |
| F3A | C47 | C43 | 108.0(2) |  | F20A | C71 | F19A | 113.9(11) |
| F4 | C48 | F5 | 106.41(16) |  | F20A | C71 | C67 | 116.4(4) |
| F4 | C48 | F6 | 107.33(17) |  | F21 | C72 | F22 | 109.2(7) |
| F4 | C48 | C45 | 113.27(15) |  | F21 | C72 | F23 | 105.9(9) |
| F5 | C48 | F6 | 104.85(16) |  | F21 | C72 | C69 | 111.7(4) |
| F5 | C48 | C45 | 113.10(14) |  | F22 | C72 | C69 | 103.7(9) |
| F6 | C48 | C45 | 111.32(14) |  | F23 | C72 | F22 | 109.7(10) |
| C50 | C49 | C54 | 115.49(13) |  | F23 | C72 | C69 | 116.5(10) |
| C50 | C49 | B1 | 123.73(13) |  | F21A | C72A | C69 | 106.7(4) |
| C54 | C49 | B1 | 120.50(13) |  | F22A | C72A | F21A | 102.3(4) |
| C49 | C50 | C51 | 122.38(16) |  | F22A | C72A | C69 | 115.8(2) |
| C50 | C51 | C55 | 119.02(17) |  | F23A | C72A | F21A | 101.7(3) |
| C52 | C51 | C50 | 120.98(16) |  | F23A | C72A | F22A | 110.5(4) |
| C52 | C51 | C55 | 119.99(15) |  | F23A | C72A | C69 | 117.5(3) |
| C51 | C52 | C53 | 117.92(14) |  | C41 | B1 | C49 | 111.70(11) |
| C52 | C53 | C54 | 120.98(16) |  | C41 | B1 | C57 | 111.22(11) |
| C52 | C53 | C56 | 120.8(3) |  | C41 | B1 | C65 | 103.36(11) |
| C52 | C53 | C56A | 116.8(5) |  | C49 | B1 | C65 | 114.20(11) |
| C54 | C53 | C56 | 118.0(3) |  | C57 | B1 | C49 | 104.66(11) |
| C54 | C53 | C56A | 121.9(5) |  | C57 | B1 | C65 | 111.90(11) |
| C53 | C54 | C49 | 122.24(16) |  | C1 | N1 | C5 | 122.28(13) |
| F7 | C55 | C51 | 113.72(15) |  | N1 | C1 | C2 | 120.24(15) |
| F7A | C55 | F8A | 105.0(7) |  | C3 | C2 | C1 | 118.78(15) |
| F7A | C55 | F9A | 118.9(8) |  | C2 | C3 | C4 | 120.14(15) |
| F7A | C55 | C51 | 111.3(6) |  | C3 | C4 | C5 | 119.93(15) |
| F8 | C55 | F7 | 104.0(2) |  | N1 | C5 | C4 | 118.61(14) |
| F8 | C55 | F9 | 108.90(19) |  | N1 | C5 | C6 | 117.36(13) |
| F8 | C55 | C51 | 114.35(17) |  | C4 | C5 | C6 | 124.01(14) |
| F8A | C55 | C51 | 101.9(5) |  | C5 | C6 | C7 | 114.18(13) |
| F9 | C55 | F7 | 103.10(19) |  | C8 | C7 | C6 | 119.45(15) |
| F9 | C55 | C51 | 111.9(2) |  | C12 | C7 | C6 | 120.46(14) |
| F9A | C55 | F8A | 103.9(7) |  | C12 | C7 | C8 | 120.08(14) |
| F9A | C55 | C51 | 113.7(6) |  | C7 | C8 | C9 | 121.00(15) |
| F10 | C56 | F11 | 103.6(6) |  | C8 | C9 | C10 | 117.44(14) |
| F10 | C56 | C53 | 111.3(6) |  | C8 | C9 | C13 | 122.84(15) |
| F11 | C56 | C53 | 114.0(7) |  | C10 | C9 | C13 | 119.72(14) |
| F12 | C56 | F10 | 108.0(8) |  | C9 | C10 | C11 | 122.92(14) |
| F12 | C56 | F11 | 106.6(7) |  | C10 | C11 | C17 | 119.37(14) |
| F12 | C56 | C53 | 112.8(6) |  | C12 | C11 | C10 | 117.64(15) |
| F10A | C56A | C53 | 114.0(10) |  | C12 | C11 | C17 | 122.99(14) |
| F11A | C56A | F10A | 105.2(12) |  | C7 | C12 | C11 | 120.91(15) |
| F11A | C56A | F12A | 104.0(10) |  | C9 | C13 | C15 | 109.32(14) |
| F11A | C56A | C53 | 112.3(10) |  | C9 | C13 | C16 | 109.12(13) |
| F12A | C56A | F10A | 104.3(11) |  | C14 | C13 | C9 | 112.29(13) |
| F12A | C56A | C53 | 116.0(10) |  | C14 | C13 | C15 | 108.47(14) |
| C58 | C57 | B1 | 122.61(12) |  | C14 | C13 | C16 | 108.26(15) |
| C62 | C57 | C58 | 115.76(13) |  | C16 | C13 | C15 | 109.33(15) |
| C62 | C57 | B1 | 121.39(12) |  | C11 | C17 | C18 | 108.93(14) |
| C59 | C58 | C57 | 121.74(14) |  | C11 | C17 | C19 | 109.20(14) |
| C58 | C59 | C63 | 123.2(2) |  | C11 | C17 | C20 | 112.52(14) |
| C58 | C59 | C63A | 117.2(5) |  | C18 | C17 | C19 | 109.70(15) |
| C58 | C59 | C63B | 111.3(4) |  | C20 | C17 | C18 | 108.42(15) |
| C60 | C59 | C58 | 121.45(14) |  | C20 | C17 | C19 | 108.03(15) |
| C60 | C59 | C63 | 115.3(2) |  | C21 | N2 | C25 | 119.41(14) |
| C60 | C59 | C63A | 121.3(5) |  | N2 | C21 | C22 | 123.00(16) |
| C60 | C59 | C63B | 127.2(4) |  | C21 | C22 | C23 | 118.08(16) |
| C61 | C60 | C59 | 117.70(13) |  | C22 | C23 | C24 | 119.22(16) |
| C60 | C61 | C62 | 120.96(14) |  | C23 | C24 | C25 | 119.87(16) |
| C60 | C61 | C64 | 119.11(13) |  | N2 | C25 | C24 | 120.37(15) |
| C62 | C61 | C64 | 119.92(14) |  | N2 | C25 | C26 | 116.51(13) |
| C61 | C62 | C57 | 122.35(13) |  | C24 | C25 | C26 | 123.10(15) |
| F13 | C63 | C59 | 111.9(3) |  | C25 | C26 | C27 | 114.80(13) |
| F14 | C63 | F13 | 106.2(4) |  | C28 | C27 | C26 | 120.17(14) |
| F14 | C63 | C59 | 112.7(4) |  | C32 | C27 | C26 | 120.38(14) |
| F15 | C63 | F13 | 104.6(4) |  | C32 | C27 | C28 | 119.34(14) |
| F15 | C63 | F14 | 104.4(4) |  | C29 | C28 | C27 | 121.27(14) |
| F15 | C63 | C59 | 116.2(3) |  | C28 | C29 | C30 | 117.64(14) |
| F13A | C63A | F14A | 104.5(9) |  | C28 | C29 | C33 | 123.35(14) |
| F13A | C63A | F15A | 108.6(9) |  | C30 | C29 | C33 | 119.00(14) |
| F13A | C63A | C59 | 115.8(8) |  | C31 | C30 | C29 | 122.75(14) |
| F14A | C63A | F15A | 105.9(9) |  | C30 | C31 | C32 | 117.70(14) |
| F14A | C63A | C59 | 112.3(11) |  | C30 | C31 | C37 | 122.53(13) |
| F15A | C63A | C59 | 109.1(8) |  | C32 | C31 | C37 | 119.77(13) |
| F13B | C63B | F15B | 105.6(9) |  | C27 | C32 | C31 | 121.29(14) |
| F13B | C63B | C59 | 113.5(9) |  | C29 | C33 | C35 | 110.36(14) |
| F14B | C63B | F13B | 106.4(9) |  | C34 | C33 | C29 | 108.58(14) |
| F14B | C63B | F15B | 105.8(10) |  | C34 | C33 | C35 | 107.61(15) |
| F14B | C63B | C59 | 116.3(10) |  | C36 | C33 | C29 | 112.25(15) |
| F15B | C63B | C59 | 108.5(8) |  | C36 | C33 | C34 | 109.75(18) |
| F005 | C64 | F16 | 106.96(14) |  | C36 | C33 | C35 | 108.19(18) |
| F005 | C64 | F17 | 106.50(14) |  | C31 | C37 | C39 | 109.63(14) |
| F005 | C64 | C61 | 113.10(14) |  | C31 | C37 | C40 | 108.84(14) |
| F16 | C64 | C61 | 111.92(13) |  | C38 | C37 | C31 | 112.47(14) |
| F17 | C64 | F16 | 105.03(14) |  | C38 | C37 | C39 | 107.52(15) |
| F17 | C64 | C61 | 112.79(13) |  | C38 | C37 | C40 | 108.74(15) |
| C66 | C65 | C70 | 115.53(13) |  | C40 | C37 | C39 | 109.61(15) |

Table 6 Torsion Angles for c280220.

| A | B | C | D | Angle/˚ |  | A | B | C | D | Angle/˚ |
| --- | --- | --- | --- | --- | --- | --- | --- | --- | --- | --- |
| C41 | C42 | C43 | C44 | 1.2(2) |  | C65 | C66 | C67 | C71 | 179.49(15) |
| C41 | C42 | C43 | C47 | -178.76(15) |  | C66 | C65 | C70 | C69 | -0.8(2) |
| C42 | C41 | C46 | C45 | -1.2(2) |  | C66 | C65 | B1 | C41 | -89.24(15) |
| C42 | C41 | B1 | C49 | -30.79(18) |  | C66 | C65 | B1 | C49 | 32.32(19) |
| C42 | C41 | B1 | C57 | -147.33(13) |  | C66 | C65 | B1 | C57 | 150.99(13) |
| C42 | C41 | B1 | C65 | 92.44(15) |  | C66 | C67 | C68 | C69 | -0.4(2) |
| C42 | C43 | C44 | C45 | -1.2(2) |  | C66 | C67 | C71 | F18 | -0.4(7) |
| C42 | C43 | C47 | F1 | 19.2(5) |  | C66 | C67 | C71 | F18A | -28.8(10) |
| C42 | C43 | C47 | F1A | -22.7(7) |  | C66 | C67 | C71 | F19 | -129.2(4) |
| C42 | C43 | C47 | F2 | 125.9(4) |  | C66 | C67 | C71 | F19A | -138.2(9) |
| C42 | C43 | C47 | F2A | 113.6(4) |  | C66 | C67 | C71 | F20 | 118.3(4) |
| C42 | C43 | C47 | F3 | -99.4(8) |  | C66 | C67 | C71 | F20A | 87.5(17) |
| C42 | C43 | C47 | F3A | -136.9(3) |  | C67 | C68 | C69 | C70 | 1.3(3) |
| C43 | C44 | C45 | C46 | 0.0(2) |  | C67 | C68 | C69 | C72 | 169.9(4) |
| C43 | C44 | C45 | C48 | 177.06(15) |  | C67 | C68 | C69 | C72A | -171.7(3) |
| C44 | C43 | C47 | F1 | -160.8(4) |  | C68 | C67 | C71 | F18 | -179.8(6) |
| C44 | C43 | C47 | F1A | 157.4(7) |  | C68 | C67 | C71 | F18A | 151.8(10) |
| C44 | C43 | C47 | F2 | -54.0(5) |  | C68 | C67 | C71 | F19 | 51.4(4) |
| C44 | C43 | C47 | F2A | -66.4(4) |  | C68 | C67 | C71 | F19A | 42.4(9) |
| C44 | C43 | C47 | F3 | 80.7(8) |  | C68 | C67 | C71 | F20 | -61.2(4) |
| C44 | C43 | C47 | F3A | 43.2(3) |  | C68 | C67 | C71 | F20A | -91.9(17) |
| C44 | C45 | C46 | C41 | 1.2(2) |  | C68 | C69 | C70 | C65 | -0.6(3) |
| C44 | C45 | C48 | F4 | 22.6(2) |  | C68 | C69 | C72 | F21 | -6.4(9) |
| C44 | C45 | C48 | F5 | 143.80(16) |  | C68 | C69 | C72 | F22 | 111.1(7) |
| C44 | C45 | C48 | F6 | -98.4(2) |  | C68 | C69 | C72 | F23 | -128.3(9) |
| C46 | C41 | C42 | C43 | 0.0(2) |  | C68 | C69 | C72A | F21A | -113.1(3) |
| C46 | C41 | B1 | C49 | 157.12(12) |  | C68 | C69 | C72A | F22A | 0.0(6) |
| C46 | C41 | B1 | C57 | 40.59(17) |  | C68 | C69 | C72A | F23A | 133.7(4) |
| C46 | C41 | B1 | C65 | -79.64(15) |  | C70 | C65 | C66 | C67 | 1.7(2) |
| C46 | C45 | C48 | F4 | -160.31(17) |  | C70 | C65 | B1 | C41 | 83.31(15) |
| C46 | C45 | C48 | F5 | -39.1(2) |  | C70 | C65 | B1 | C49 | -155.13(13) |
| C46 | C45 | C48 | F6 | 78.6(2) |  | C70 | C65 | B1 | C57 | -36.45(18) |
| C47 | C43 | C44 | C45 | 178.75(16) |  | C70 | C69 | C72 | F21 | 162.7(7) |
| C48 | C45 | C46 | C41 | -175.84(14) |  | C70 | C69 | C72 | F22 | -79.8(8) |
| C49 | C50 | C51 | C52 | -0.4(2) |  | C70 | C69 | C72 | F23 | 40.8(10) |
| C49 | C50 | C51 | C55 | -179.25(16) |  | C70 | C69 | C72A | F21A | 73.9(4) |
| C50 | C49 | C54 | C53 | -1.3(2) |  | C70 | C69 | C72A | F22A | -173.1(4) |
| C50 | C49 | B1 | C41 | 146.60(13) |  | C70 | C69 | C72A | F23A | -39.4(6) |
| C50 | C49 | B1 | C57 | -92.95(16) |  | C71 | C67 | C68 | C69 | 179.00(16) |
| C50 | C49 | B1 | C65 | 29.75(19) |  | C72 | C69 | C70 | C65 | -169.6(4) |
| C50 | C51 | C52 | C53 | -0.8(2) |  | C72A | C69 | C70 | C65 | 172.4(3) |
| C50 | C51 | C55 | F7 | -33.7(2) |  | B1 | C41 | C42 | C43 | -172.46(14) |
| C50 | C51 | C55 | F7A | -96.9(7) |  | B1 | C41 | C46 | C45 | 171.41(13) |
| C50 | C51 | C55 | F8 | -153.0(2) |  | B1 | C49 | C50 | C51 | 175.19(14) |
| C50 | C51 | C55 | F8A | 151.7(5) |  | B1 | C49 | C54 | C53 | -175.27(14) |
| C50 | C51 | C55 | F9 | 82.6(2) |  | B1 | C57 | C58 | C59 | -176.12(13) |
| C50 | C51 | C55 | F9A | 40.6(7) |  | B1 | C57 | C62 | C61 | 174.50(12) |
| C51 | C52 | C53 | C54 | 0.9(2) |  | B1 | C65 | C66 | C67 | 174.59(14) |
| C51 | C52 | C53 | C56 | 175.6(3) |  | B1 | C65 | C70 | C69 | -173.94(15) |
| C51 | C52 | C53 | C56A | -173.1(5) |  | N1 | C1 | C2 | C3 | 0.2(3) |
| C52 | C51 | C55 | F7 | 147.48(17) |  | N1 | C5 | C6 | C7 | -60.4(2) |
| C52 | C51 | C55 | F7A | 84.2(7) |  | C1 | N1 | C5 | C4 | -1.3(2) |
| C52 | C51 | C55 | F8 | 28.1(3) |  | C1 | N1 | C5 | C6 | -179.77(14) |
| C52 | C51 | C55 | F8A | -27.2(5) |  | C1 | C2 | C3 | C4 | -1.1(3) |
| C52 | C51 | C55 | F9 | -96.2(2) |  | C2 | C3 | C4 | C5 | 0.8(3) |
| C52 | C51 | C55 | F9A | -138.3(7) |  | C3 | C4 | C5 | N1 | 0.3(2) |
| C52 | C53 | C54 | C49 | 0.1(2) |  | C3 | C4 | C5 | C6 | 178.68(15) |
| C52 | C53 | C56 | F10 | 141.0(5) |  | C4 | C5 | C6 | C7 | 121.25(17) |
| C52 | C53 | C56 | F11 | -102.3(6) |  | C5 | N1 | C1 | C2 | 1.1(2) |
| C52 | C53 | C56 | F12 | 19.4(7) |  | C5 | C6 | C7 | C8 | 130.83(15) |
| C52 | C53 | C56A | F10A | -175.3(8) |  | C5 | C6 | C7 | C12 | -50.6(2) |
| C52 | C53 | C56A | F11A | -55.9(10) |  | C6 | C7 | C8 | C9 | 178.32(14) |
| C52 | C53 | C56A | F12A | 63.4(10) |  | C6 | C7 | C12 | C11 | -177.67(14) |
| C54 | C49 | C50 | C51 | 1.4(2) |  | C7 | C8 | C9 | C10 | -0.7(2) |
| C54 | C49 | B1 | C41 | -39.91(17) |  | C7 | C8 | C9 | C13 | 179.11(14) |
| C54 | C49 | B1 | C57 | 80.54(16) |  | C8 | C7 | C12 | C11 | 0.9(2) |
| C54 | C49 | B1 | C65 | -156.75(13) |  | C8 | C9 | C10 | C11 | 1.1(2) |
| C54 | C53 | C56 | F10 | -44.2(7) |  | C8 | C9 | C13 | C14 | -2.8(2) |
| C54 | C53 | C56 | F11 | 72.5(6) |  | C8 | C9 | C13 | C15 | 117.61(17) |
| C54 | C53 | C56 | F12 | -165.8(5) |  | C8 | C9 | C13 | C16 | -122.86(17) |
| C54 | C53 | C56A | F10A | 10.7(12) |  | C9 | C10 | C11 | C12 | -0.5(2) |
| C54 | C53 | C56A | F11A | 130.2(8) |  | C9 | C10 | C11 | C17 | -179.90(14) |
| C54 | C53 | C56A | F12A | -110.5(9) |  | C10 | C9 | C13 | C14 | 177.01(14) |
| C55 | C51 | C52 | C53 | 178.04(16) |  | C10 | C9 | C13 | C15 | -62.55(19) |
| C56 | C53 | C54 | C49 | -174.7(3) |  | C10 | C9 | C13 | C16 | 57.0(2) |
| C56A | C53 | C54 | C49 | 173.8(5) |  | C10 | C11 | C12 | C7 | -0.5(2) |
| C57 | C58 | C59 | C60 | 1.9(2) |  | C10 | C11 | C17 | C18 | 62.81(19) |
| C57 | C58 | C59 | C63 | -177.0(2) |  | C10 | C11 | C17 | C19 | -57.0(2) |
| C57 | C58 | C59 | C63A | 178.5(5) |  | C10 | C11 | C17 | C20 | -176.93(15) |
| C57 | C58 | C59 | C63B | -175.7(4) |  | C12 | C7 | C8 | C9 | -0.2(2) |
| C58 | C57 | C62 | C61 | -0.02(19) |  | C12 | C11 | C17 | C18 | -116.54(17) |
| C58 | C57 | B1 | C41 | -147.30(13) |  | C12 | C11 | C17 | C19 | 123.67(17) |
| C58 | C57 | B1 | C49 | 91.93(15) |  | C12 | C11 | C17 | C20 | 3.7(2) |
| C58 | C57 | B1 | C65 | -32.26(17) |  | C13 | C9 | C10 | C11 | -178.72(14) |
| C58 | C59 | C60 | C61 | -0.3(2) |  | C17 | C11 | C12 | C7 | 178.86(14) |
| C58 | C59 | C63 | F13 | 30.1(6) |  | N2 | C21 | C22 | C23 | 1.1(3) |
| C58 | C59 | C63 | F14 | -89.4(6) |  | N2 | C25 | C26 | C27 | 69.80(18) |
| C58 | C59 | C63 | F15 | 150.2(4) |  | C21 | N2 | C25 | C24 | -2.0(2) |
| C58 | C59 | C63A | F13A | -1.6(13) |  | C21 | N2 | C25 | C26 | 176.68(14) |
| C58 | C59 | C63A | F14A | -121.6(10) |  | C21 | C22 | C23 | C24 | -1.7(3) |
| C58 | C59 | C63A | F15A | 121.3(10) |  | C22 | C23 | C24 | C25 | 0.5(3) |
| C58 | C59 | C63B | F13B | -50.5(13) |  | C23 | C24 | C25 | N2 | 1.4(2) |
| C58 | C59 | C63B | F14B | 73.4(14) |  | C23 | C24 | C25 | C26 | -177.21(15) |
| C58 | C59 | C63B | F15B | -167.4(12) |  | C24 | C25 | C26 | C27 | -111.56(17) |
| C59 | C60 | C61 | C62 | -1.3(2) |  | C25 | N2 | C21 | C22 | 0.7(2) |
| C59 | C60 | C61 | C64 | 179.99(13) |  | C25 | C26 | C27 | C28 | -136.23(15) |
| C60 | C59 | C63 | F13 | -148.8(6) |  | C25 | C26 | C27 | C32 | 47.6(2) |
| C60 | C59 | C63 | F14 | 91.6(7) |  | C26 | C27 | C28 | C29 | -175.27(14) |
| C60 | C59 | C63 | F15 | -28.8(6) |  | C26 | C27 | C32 | C31 | 174.93(14) |
| C60 | C59 | C63A | F13A | 175.0(11) |  | C27 | C28 | C29 | C30 | -0.5(2) |
| C60 | C59 | C63A | F14A | 55.0(12) |  | C27 | C28 | C29 | C33 | 179.95(15) |
| C60 | C59 | C63A | F15A | -62.1(12) |  | C28 | C27 | C32 | C31 | -1.3(2) |
| C60 | C59 | C63B | F13B | 132.0(11) |  | C28 | C29 | C30 | C31 | 0.4(2) |
| C60 | C59 | C63B | F14B | -104.0(13) |  | C28 | C29 | C33 | C34 | 117.06(18) |
| C60 | C59 | C63B | F15B | 15.1(13) |  | C28 | C29 | C33 | C35 | -125.22(17) |
| C60 | C61 | C62 | C57 | 1.5(2) |  | C28 | C29 | C33 | C36 | -4.5(3) |
| C60 | C61 | C64 | F005 | -30.1(2) |  | C29 | C30 | C31 | C32 | -0.7(2) |
| C60 | C61 | C64 | F16 | 90.81(17) |  | C29 | C30 | C31 | C37 | -179.98(15) |
| C60 | C61 | C64 | F17 | -151.02(14) |  | C30 | C29 | C33 | C34 | -62.5(2) |
| C62 | C57 | C58 | C59 | -1.67(19) |  | C30 | C29 | C33 | C35 | 55.2(2) |
| C62 | C57 | B1 | C41 | 38.56(17) |  | C30 | C29 | C33 | C36 | 175.98(19) |
| C62 | C57 | B1 | C49 | -82.21(15) |  | C30 | C31 | C32 | C27 | 1.1(2) |
| C62 | C57 | B1 | C65 | 153.60(12) |  | C30 | C31 | C37 | C38 | -5.9(2) |
| C62 | C61 | C64 | F005 | 151.23(15) |  | C30 | C31 | C37 | C39 | -125.45(17) |
| C62 | C61 | C64 | F16 | -87.87(17) |  | C30 | C31 | C37 | C40 | 114.66(17) |
| C62 | C61 | C64 | F17 | 30.3(2) |  | C32 | C27 | C28 | C29 | 0.9(2) |
| C63 | C59 | C60 | C61 | 178.6(2) |  | C32 | C31 | C37 | C38 | 174.81(16) |
| C63A | C59 | C60 | C61 | -176.8(5) |  | C32 | C31 | C37 | C39 | 55.2(2) |
| C63B | C59 | C60 | C61 | 176.9(5) |  | C32 | C31 | C37 | C40 | -64.63(19) |
| C64 | C61 | C62 | C57 | -179.80(13) |  | C33 | C29 | C30 | C31 | 179.94(15) |
| C65 | C66 | C67 | C68 | -1.1(2) |  | C37 | C31 | C32 | C27 | -179.54(15) |

Table 7 Hydrogen Atom Coordinates (Å×104) and Isotropic Displacement Parameters (Å2×103) for c280220.

| Atom | *x* | *y* | *z* | U(eq) |
| --- | --- | --- | --- | --- |
| H42 | 2417.21 | 5548.61 | 1698.18 | 29 |
| H44 | 939.8 | 5061.59 | -94.51 | 38 |
| H46 | 3150.14 | 5814.2 | 180.68 | 27 |
| H50 | 4848.05 | 5808.16 | 2791.36 | 33 |
| H52 | 3861.98 | 7193.18 | 3721.42 | 41 |
| H54 | 2835.05 | 6865.34 | 1805.33 | 34 |
| H58 | 5387.23 | 6164.73 | 1550.07 | 26 |
| H60 | 5211.15 | 8063.71 | 586.94 | 28 |
| H62 | 3259.04 | 7135.77 | 772.22 | 25 |
| H66 | 4090.55 | 4736.01 | 2369.22 | 26 |
| H68 | 5202.89 | 3253.72 | 1624.56 | 34 |
| H70 | 4606.85 | 5221.18 | 780.99 | 29 |
| H1 | 7482(17) | 6740(16) | 3954(14) | 78(9) |
| H1A | 7151.93 | 7186.25 | 4826.26 | 35 |
| H2 | 6963.78 | 8416.13 | 4987.75 | 40 |
| H3 | 7157.04 | 9267.47 | 4277.29 | 39 |
| H4 | 7571.06 | 8889.73 | 3439.86 | 34 |
| H6A | 7898.33 | 7919.68 | 2836.63 | 36 |
| H6B | 7409.29 | 7195.06 | 2833.98 | 36 |
| H8 | 8167.4 | 6203.05 | 2763.32 | 31 |
| H10 | 10486.45 | 6042.51 | 3830.22 | 33 |
| H12 | 9255.83 | 7831.72 | 3966.49 | 32 |
| H14A | 8392.86 | 5301.27 | 2164.73 | 51 |
| H14B | 8204.97 | 4952.88 | 2757.59 | 51 |
| H14C | 8647.15 | 4482.26 | 2376.49 | 51 |
| H15A | 10016.1 | 4769.4 | 2455.48 | 62 |
| H15B | 10495.59 | 5409.17 | 2912.47 | 62 |
| H15C | 9785.7 | 5597.33 | 2263.15 | 62 |
| H16A | 9317.92 | 4694.24 | 3790.75 | 62 |
| H16B | 10211.55 | 4840.05 | 3832.33 | 62 |
| H16C | 9706.98 | 4208.36 | 3379.7 | 62 |
| H18A | 11164.34 | 7721.35 | 3772.78 | 64 |
| H18B | 11486.66 | 6917.47 | 3981.2 | 64 |
| H18C | 11860.98 | 7588.83 | 4443.32 | 64 |
| H19A | 11547.77 | 6948.32 | 5349.42 | 60 |
| H19B | 11203.38 | 6288.45 | 4864.79 | 60 |
| H19C | 10665.51 | 6664.18 | 5220.33 | 60 |
| H20A | 10227.47 | 7969.04 | 4944.9 | 59 |
| H20B | 10461.37 | 8379.8 | 4399.86 | 59 |
| H20C | 11131.71 | 8177.51 | 5068 | 59 |
| H21 | 6367.39 | 6164.04 | 3022.13 | 38 |
| H22 | 6037.76 | 4989.1 | 2650.26 | 42 |
| H23 | 6841.28 | 4028.22 | 3208.79 | 44 |
| H24 | 7978.97 | 4289.16 | 4087.01 | 38 |
| H26A | 8844.1 | 5978.63 | 4438.91 | 33 |
| H26B | 8915.5 | 5175.54 | 4723.77 | 33 |
| H28 | 9158.42 | 6765.34 | 5325.09 | 30 |
| H30 | 8183.81 | 6534.02 | 6705.18 | 31 |
| H32 | 7638.47 | 5116.15 | 5227.72 | 30 |
| H34A | 8215.29 | 7796.22 | 6667.24 | 65 |
| H34B | 8239.14 | 8129.86 | 6008.05 | 65 |
| H34C | 8826.62 | 8431.68 | 6679.17 | 65 |
| H35A | 9916.55 | 7671.52 | 7373.27 | 65 |
| H35B | 10067.03 | 6875.27 | 7151.34 | 65 |
| H35C | 9306.2 | 7031.11 | 7349.6 | 65 |
| H36A | 10005.21 | 8176.5 | 6386.42 | 93 |
| H36B | 9441.34 | 7902.71 | 5696.03 | 93 |
| H36C | 10136.07 | 7390.32 | 6133.36 | 93 |
| H38A | 7112.01 | 6116.16 | 6903.04 | 64 |
| H38B | 7858.77 | 5606.23 | 7244.17 | 64 |
| H38C | 6980.91 | 5309.2 | 7113.33 | 64 |
| H39A | 7167.11 | 4258.5 | 6534.85 | 59 |
| H39B | 8069.35 | 4488.46 | 6675.88 | 59 |
| H39C | 7501.67 | 4328.8 | 5955.54 | 59 |
| H40A | 6387.85 | 5176.2 | 5407.69 | 61 |
| H40B | 6278.81 | 5912.19 | 5750.95 | 61 |
| H40C | 6066.1 | 5149.65 | 5996.35 | 61 |

Table 8 Atomic Occupancy for c280220.

| Atom | *Occupancy* |  | Atom | *Occupancy* |  | Atom | *Occupancy* |
| --- | --- | --- | --- | --- | --- | --- | --- |
| F1 | 0.442(13) |  | F1A | 0.558(13) |  | F2 | 0.442(13) |
| F2A | 0.558(13) |  | F3 | 0.442(13) |  | F3A | 0.558(13) |
| F7 | 0.861(3) |  | F7A | 0.139(3) |  | F8 | 0.861(3) |
| F8A | 0.139(3) |  | F9 | 0.861(3) |  | F9A | 0.139(3) |
| F10 | 0.598(6) |  | F10A | 0.402(6) |  | F11 | 0.598(6) |
| F11A | 0.402(6) |  | F12 | 0.598(6) |  | F12A | 0.402(6) |
| F13 | 0.599(12) |  | F13A | 0.225(12) |  | F13B | 0.176(7) |
| F14 | 0.599(12) |  | F14A | 0.225(12) |  | F14B | 0.176(7) |
| F15 | 0.599(12) |  | F15A | 0.225(12) |  | F15B | 0.176(7) |
| F18 | 0.65(2) |  | F18A | 0.35(2) |  | F19 | 0.65(2) |
| F19A | 0.35(2) |  | F20 | 0.65(2) |  | F20A | 0.35(2) |
| F21 | 0.218(6) |  | F21A | 0.782(6) |  | F22 | 0.218(6) |
| F22A | 0.782(6) |  | F23 | 0.218(6) |  | F23A | 0.782(6) |
| C56 | 0.598(6) |  | C56A | 0.402(6) |  | C63 | 0.599(12) |
| C63A | 0.225(12) |  | C63B | 0.176(7) |  | C72 | 0.427(11) |
| C72A | 0.573(11) |  |  |  |  |  |

c280220


# c280220

Table 1 Crystal data and structure refinement for c280220.

| Identification code | c280220 |
| Empirical formula | C72H67BF24N2 |
| Formula weight | 1427.08 |
| Temperature/K | 100.0 |
| Crystal system | monoclinic |
| Space group | P21/c |
| a/Å | 17.9819(17) |
| b/Å | 18.4321(18) |
| c/Å | 22.181(2) |
| α/° | 90 |
| β/° | 110.091(2) |
| γ/° | 90 |
| Volume/Å3 | 6904.5(12) |
| Z | 4 |
| ρcalcg/cm3 | 1.373 |
| μ/mm‑1 | 0.125 |
| F(000) | 2936.0 |
| Crystal size/mm3 | 0.559 × 0.541 × 0.525 |
| Radiation | MoKα (λ = 0.71073) |
| 2Θ range for data collection/° | 2.412 to 62.464 |
| Index ranges | -25 ≤ h ≤ 25, -26 ≤ k ≤ 25, -31 ≤ l ≤ 31 |
| Reflections collected | 91951 |
| Independent reflections | 20844 [Rint = 0.0489, Rsigma = 0.0512] |
| Data/restraints/parameters | 20844/286/1087 |
| Goodness-of-fit on F2 | 1.048 |
| Final R indexes [I>=2σ (I)] | R1 = 0.0567, wR2 = 0.1440 |
| Final R indexes [all data] | R1 = 0.0842, wR2 = 0.1596 |
| Largest diff. peak/hole / e Å-3 | 0.45/-0.41 |
