# Supplementary figures and images for: London Dispersion versus Intramolecular Hydrogen Bond in Bis‐Pyridines: How Accurate Is DFT for Competing Noncovalent Interactions in the Condensed Phase?
Source: Chemistry. 2025 Oct 23;31(66):e02745. doi: 10.1002/chem.202502745 (PMC12648470; doi:10.1002/chem.202502745)

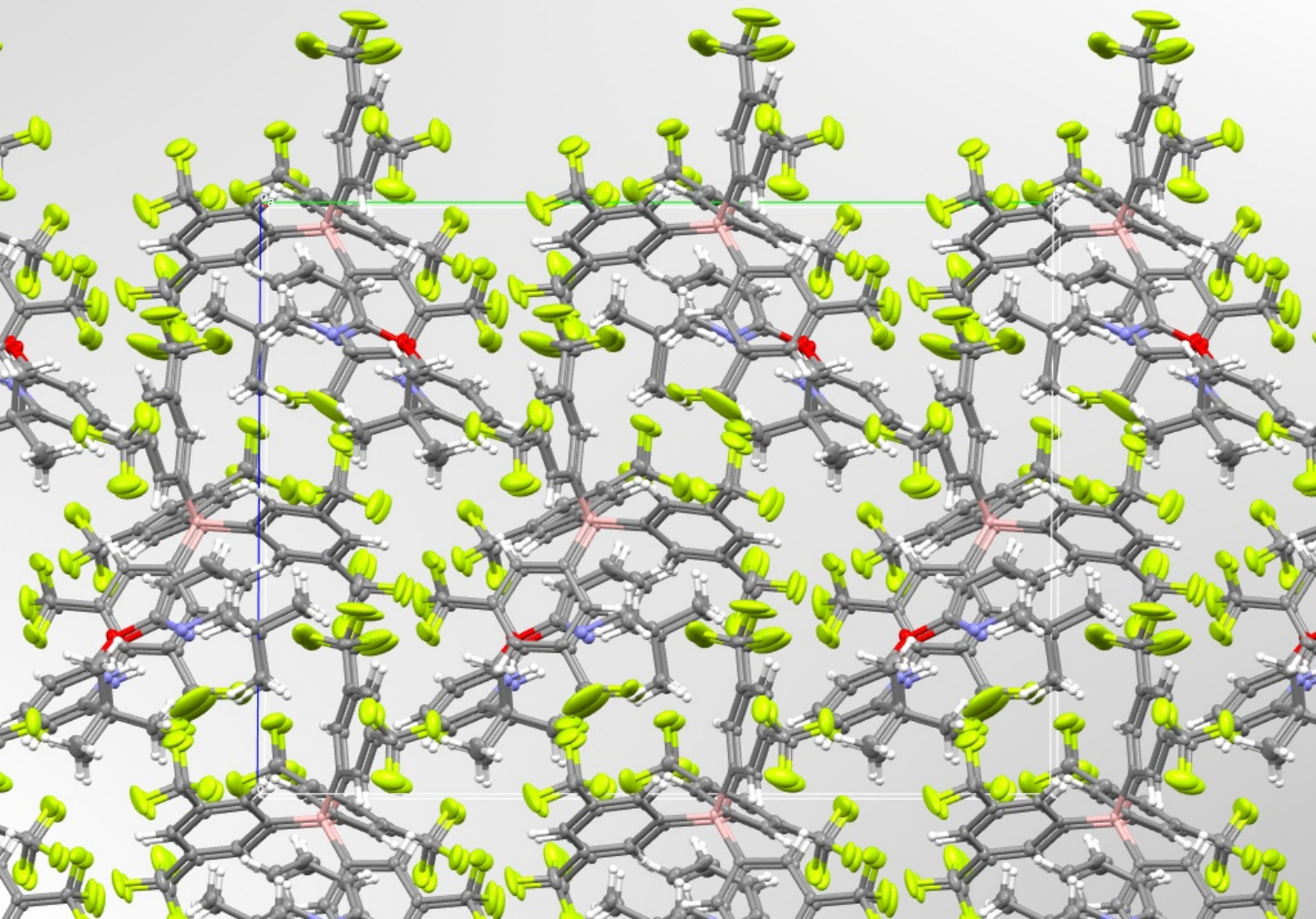

Supplement: Supplementary file 1 — Supporting Information [file CHEM-31-e02745-s002.zip › Crystal_structures/10b/packing.pdf]

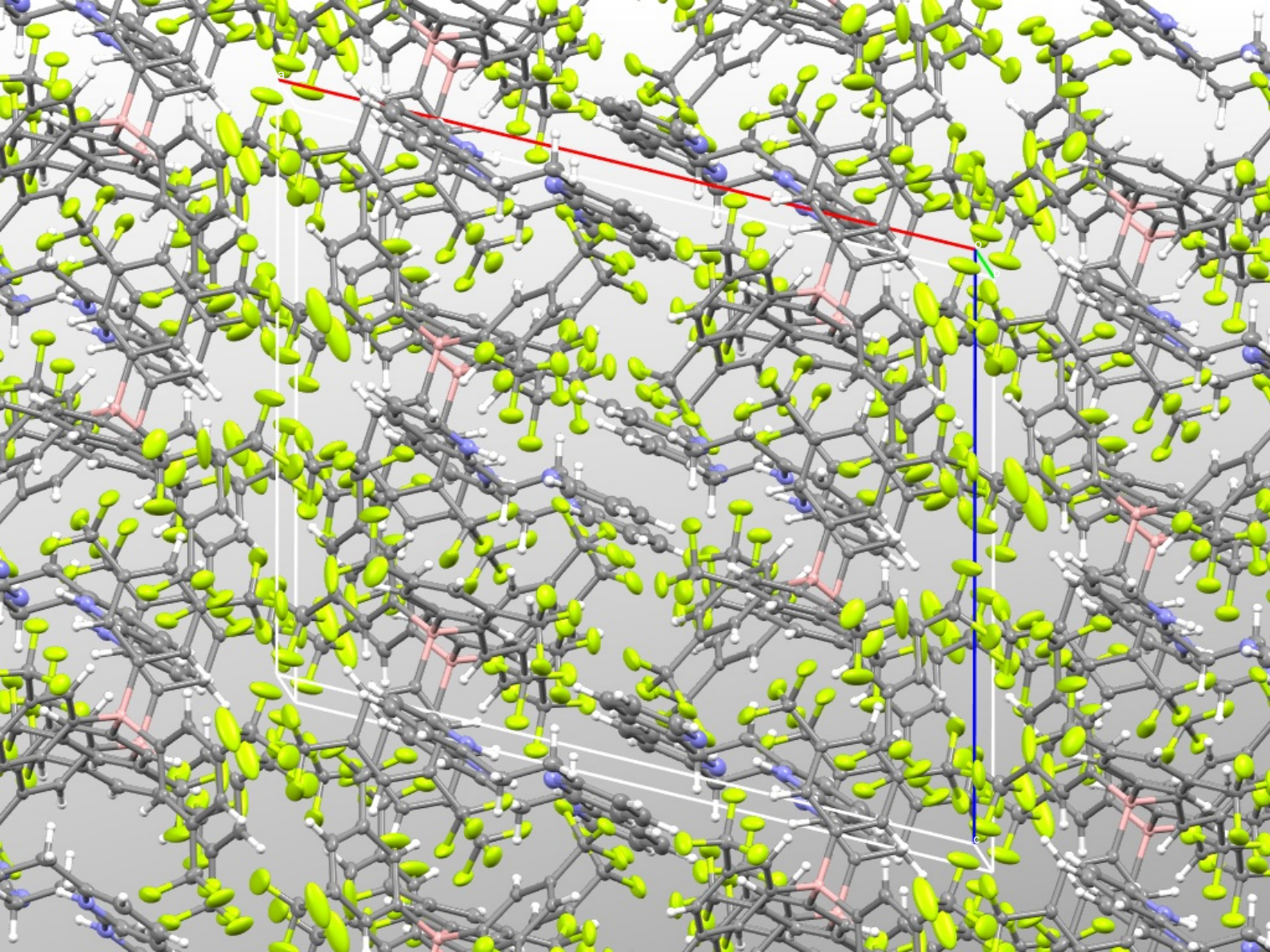

Supplement: Supplementary file 1 — Supporting Information [file CHEM-31-e02745-s002.zip › Crystal_structures/11b/packing.pdf]

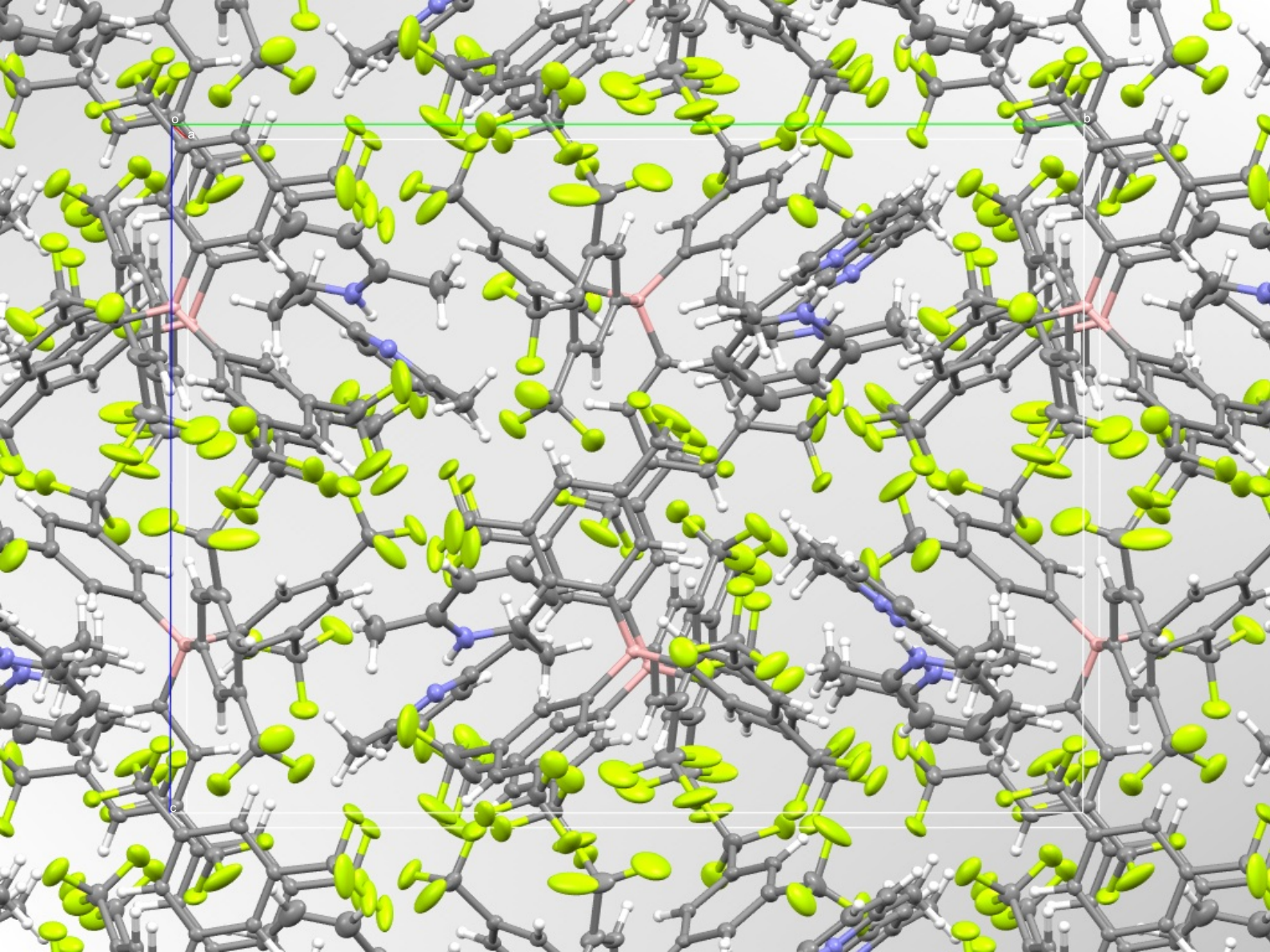

Supplement: Supplementary file 1 — Supporting Information [file CHEM-31-e02745-s002.zip › Crystal_structures/12b/packing.pdf]

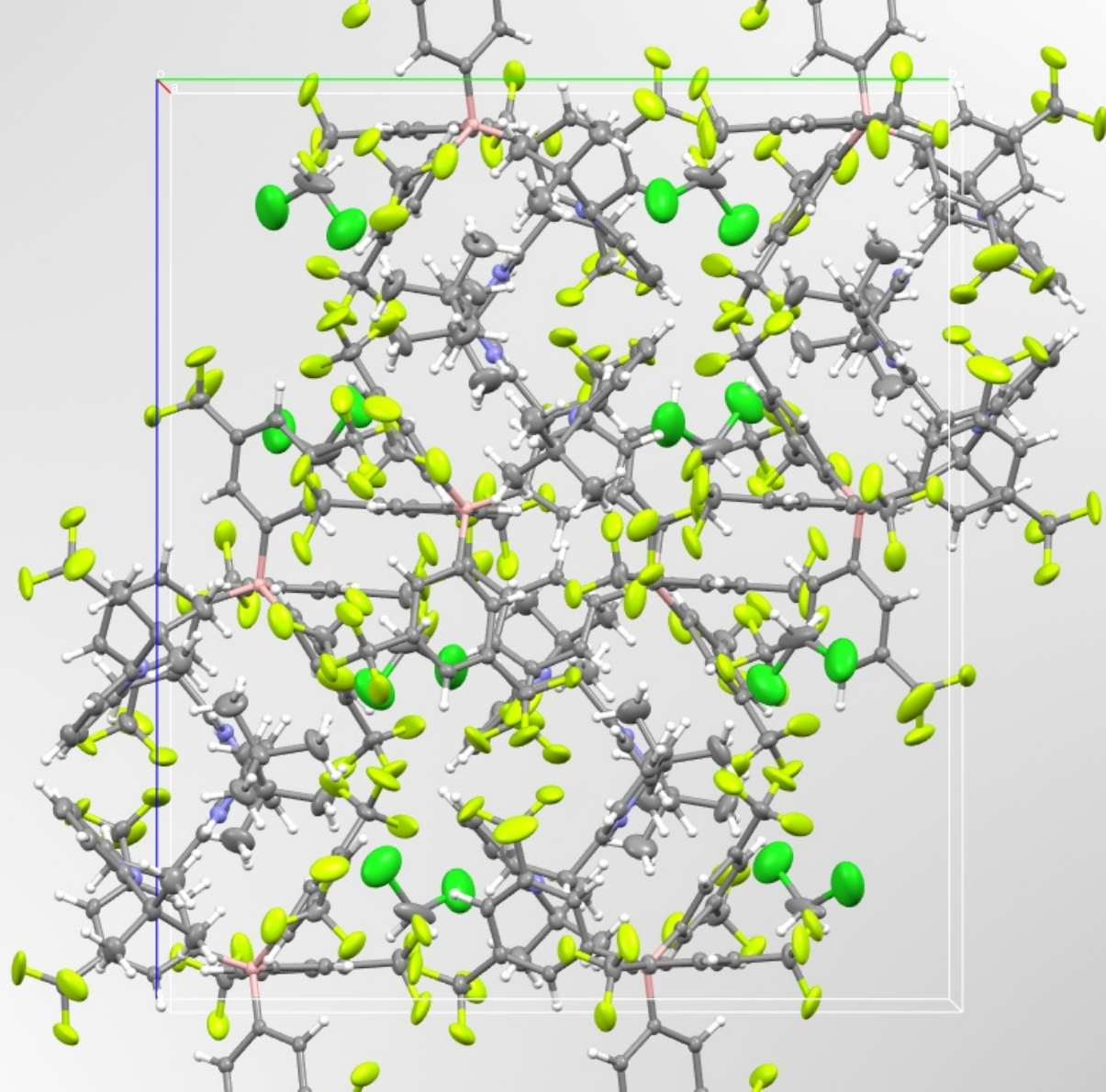

Supplement: Supplementary file 1 — Supporting Information [file CHEM-31-e02745-s002.zip › Crystal_structures/13b/packing.pdf]

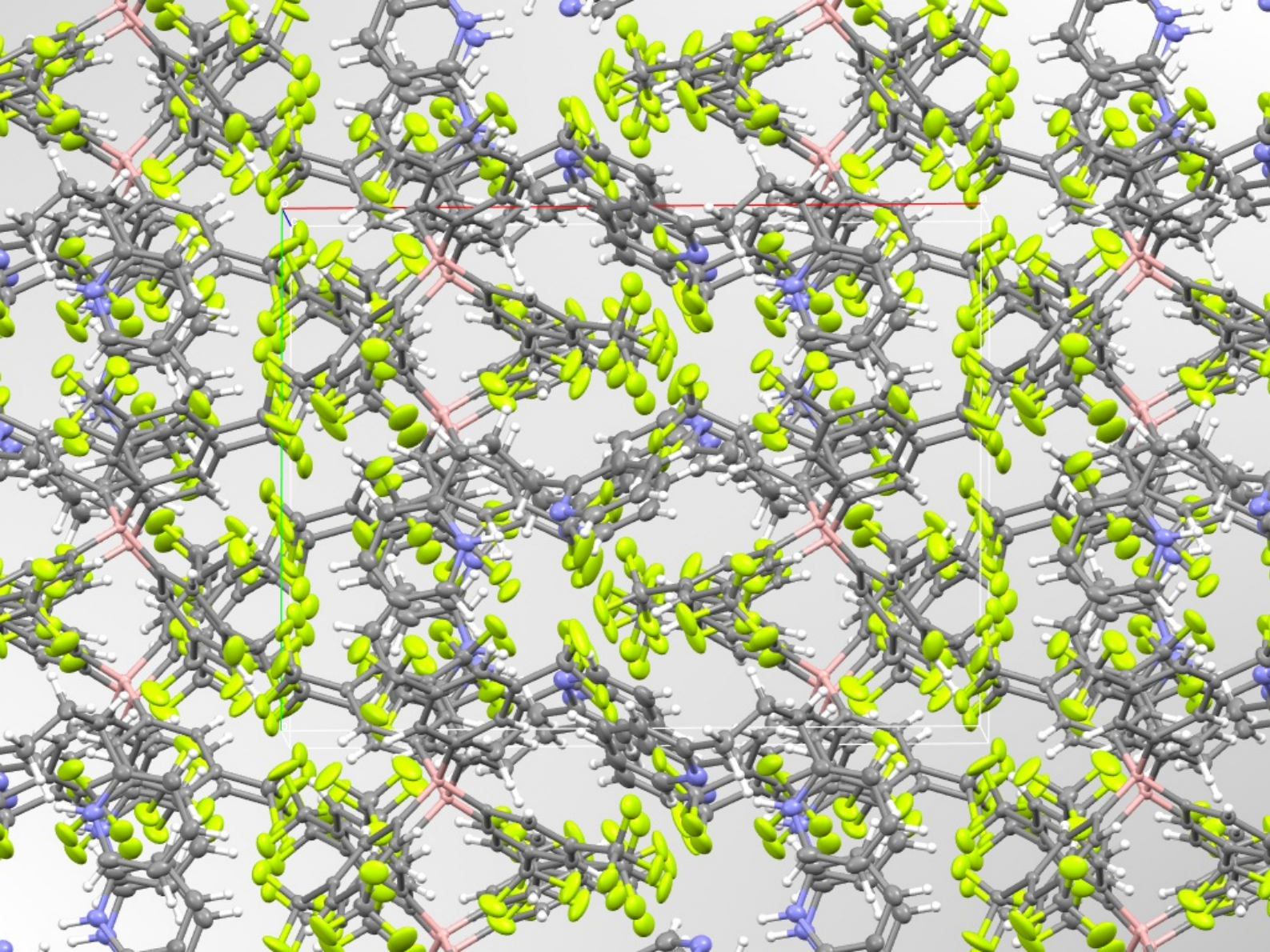

Supplement: Supplementary file 1 — Supporting Information [file CHEM-31-e02745-s002.zip › Crystal_structures/14b/packing.pdf]

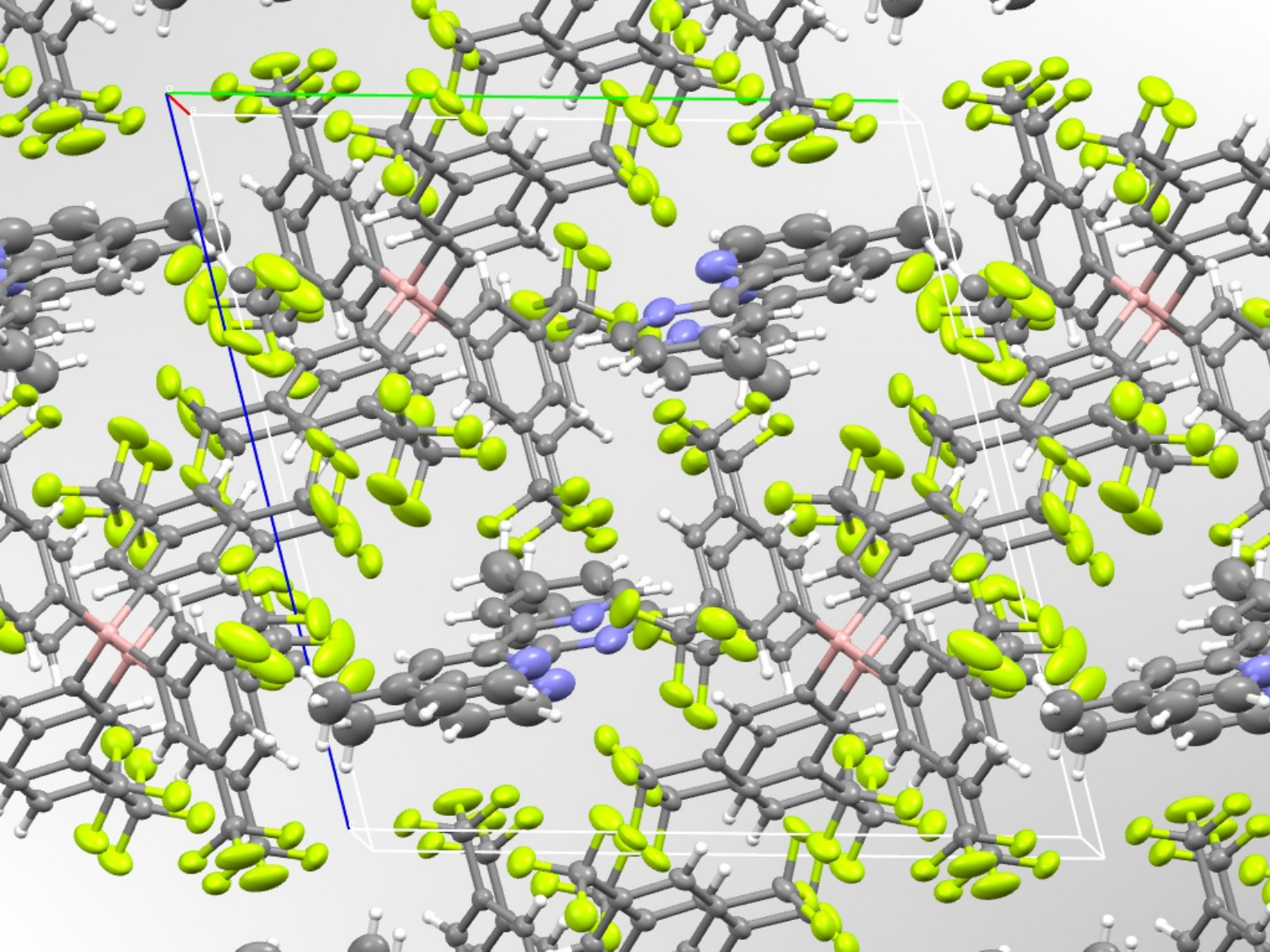

Supplement: Supplementary file 1 — Supporting Information [file CHEM-31-e02745-s002.zip › Crystal_structures/1b/packing.pdf]

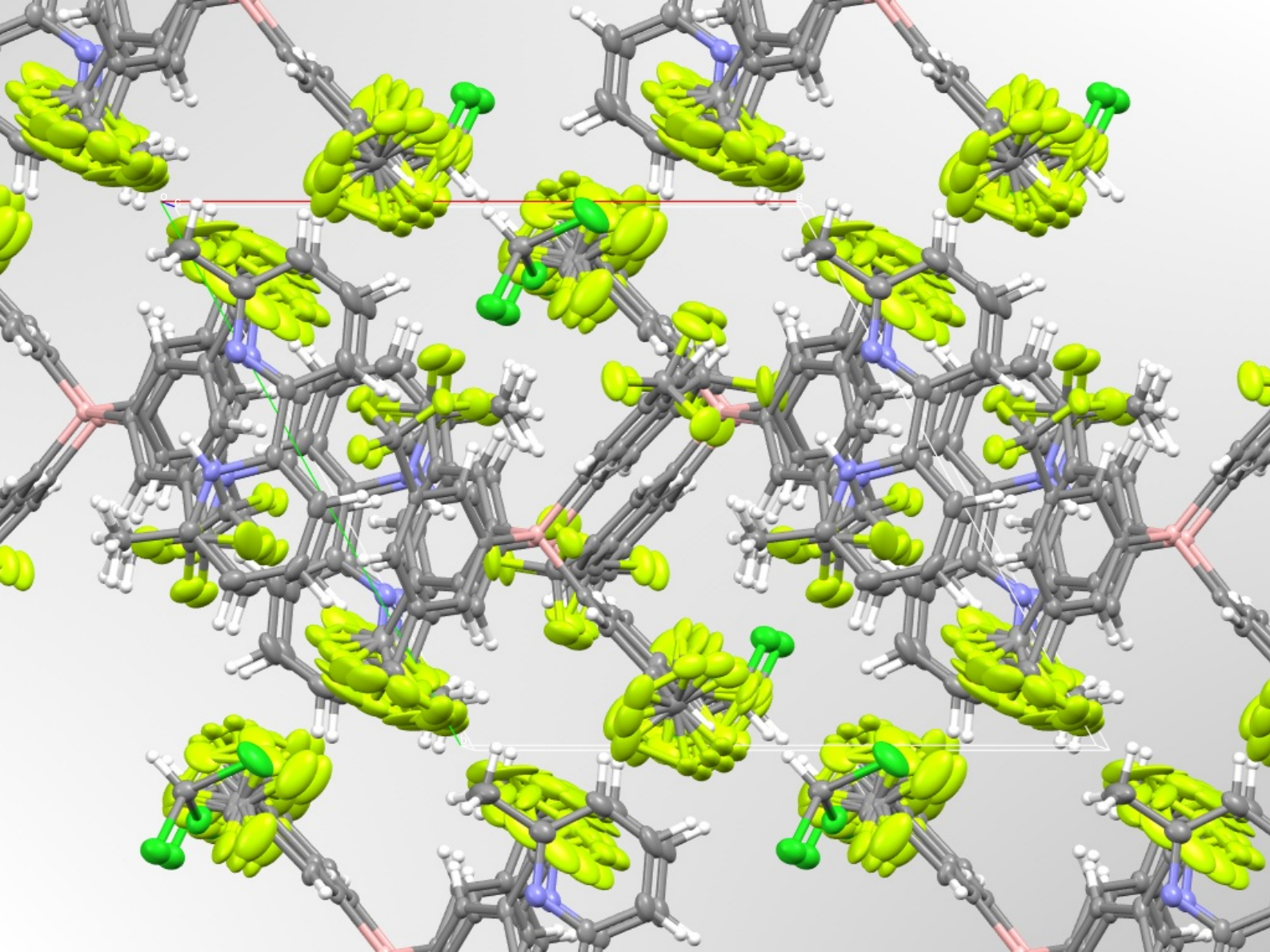

Supplement: Supplementary file 1 — Supporting Information [file CHEM-31-e02745-s002.zip › Crystal_structures/2b/packing.pdf]

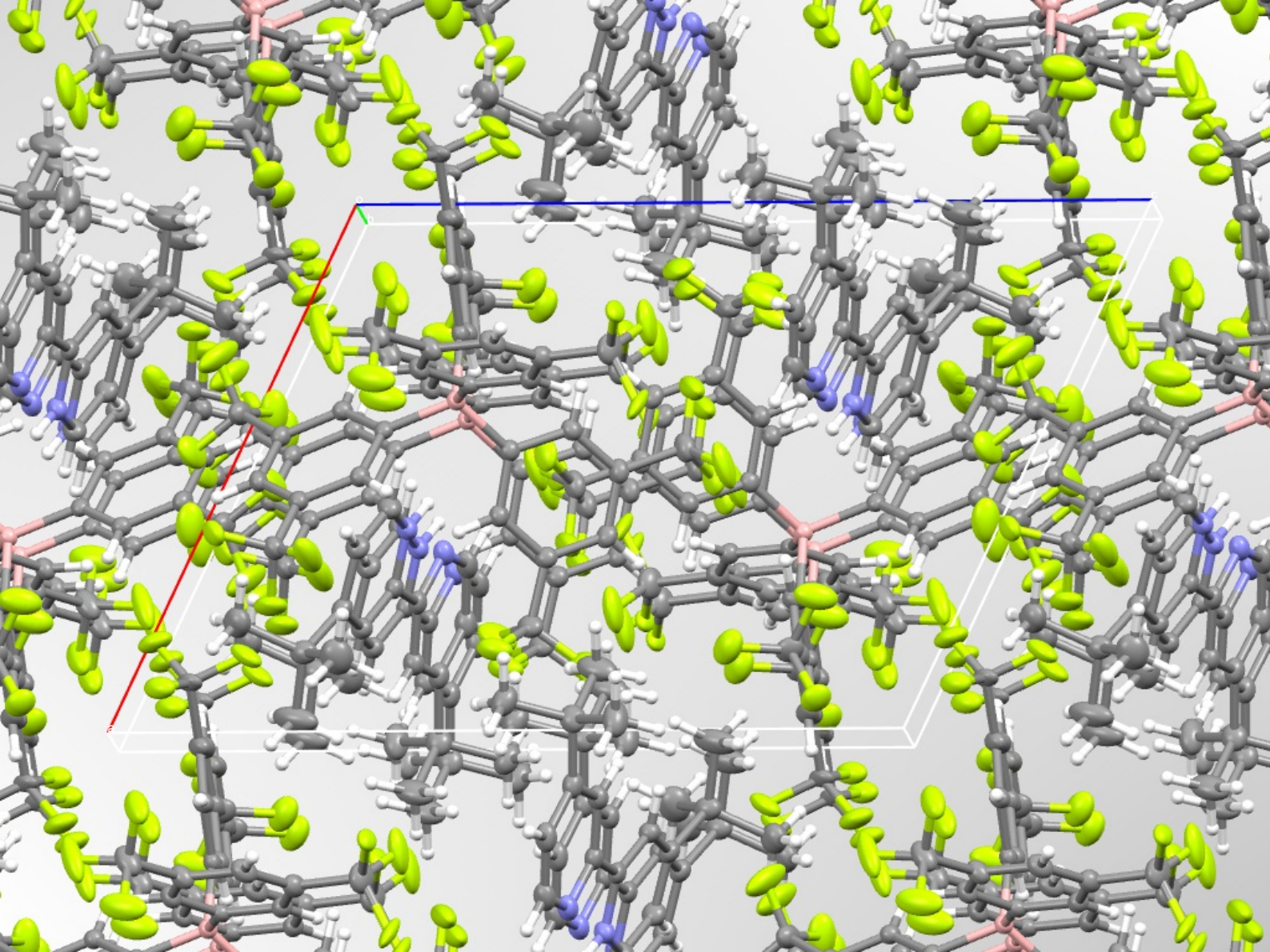

Supplement: Supplementary file 1 — Supporting Information [file CHEM-31-e02745-s002.zip › Crystal_structures/3b/packing.pdf]

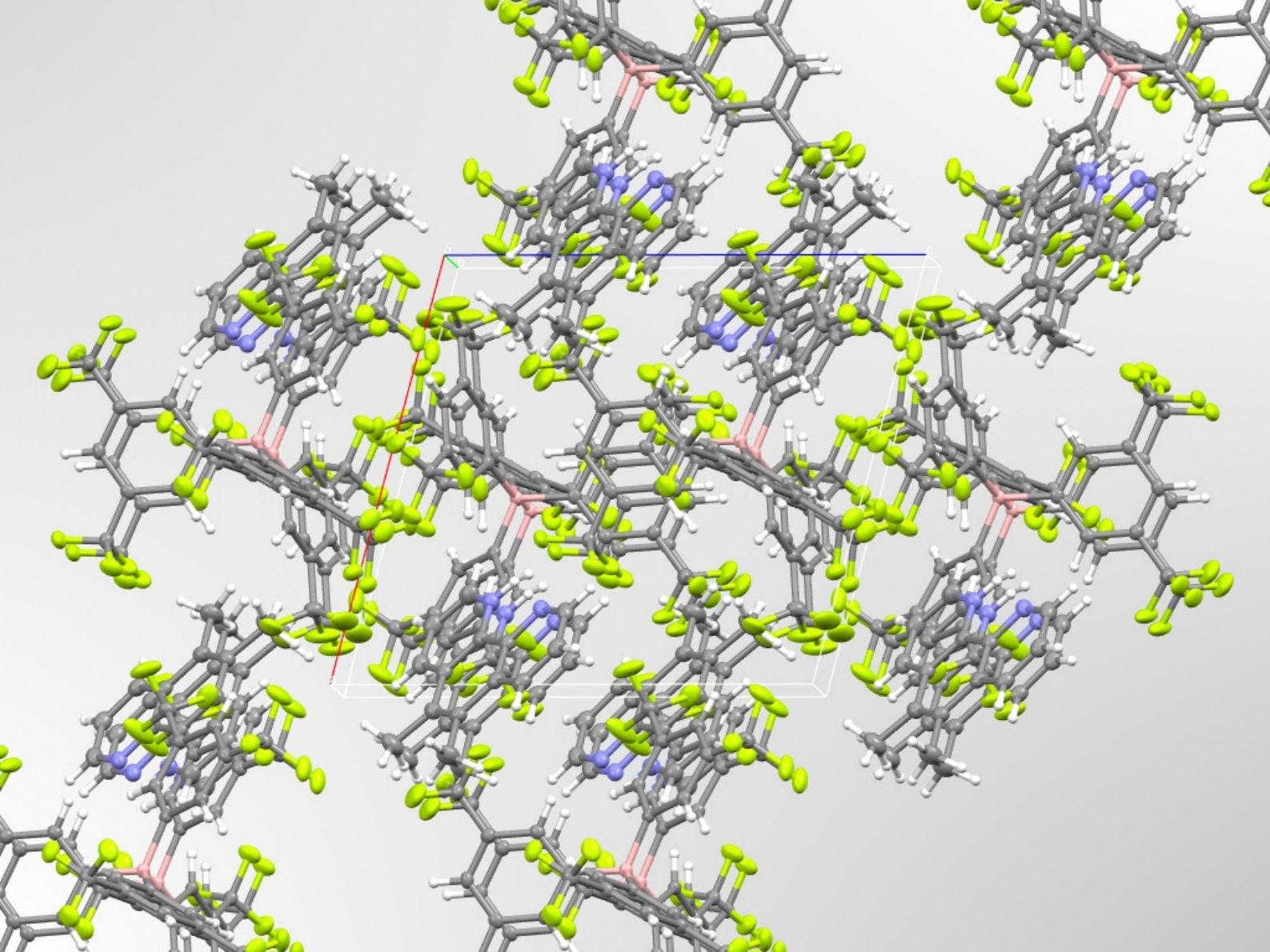

Supplement: Supplementary file 1 — Supporting Information [file CHEM-31-e02745-s002.zip › Crystal_structures/4b/packing.pdf]

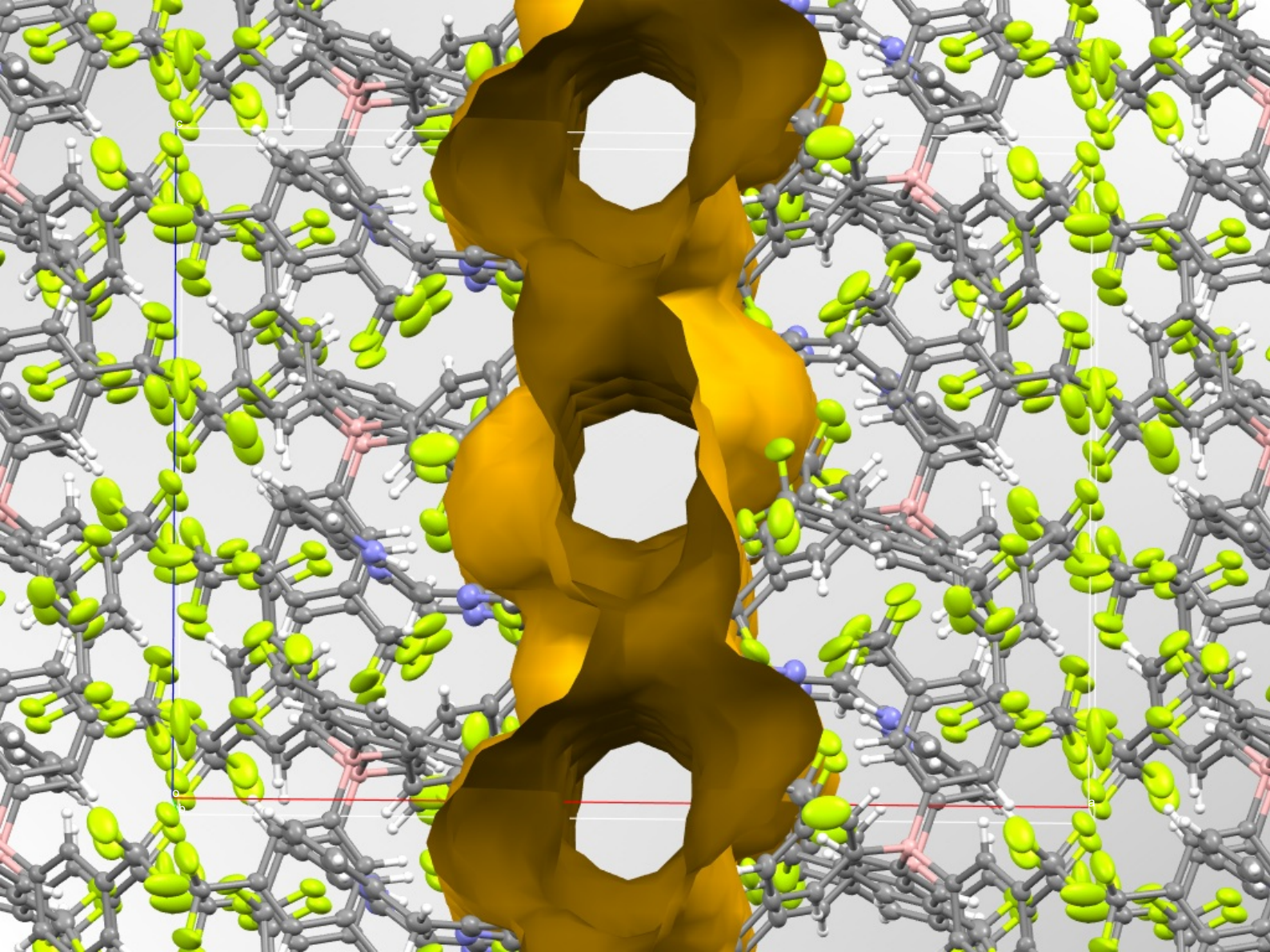

Supplement: Supplementary file 1 — Supporting Information [file CHEM-31-e02745-s002.zip › Crystal_structures/5b/packing.pdf]

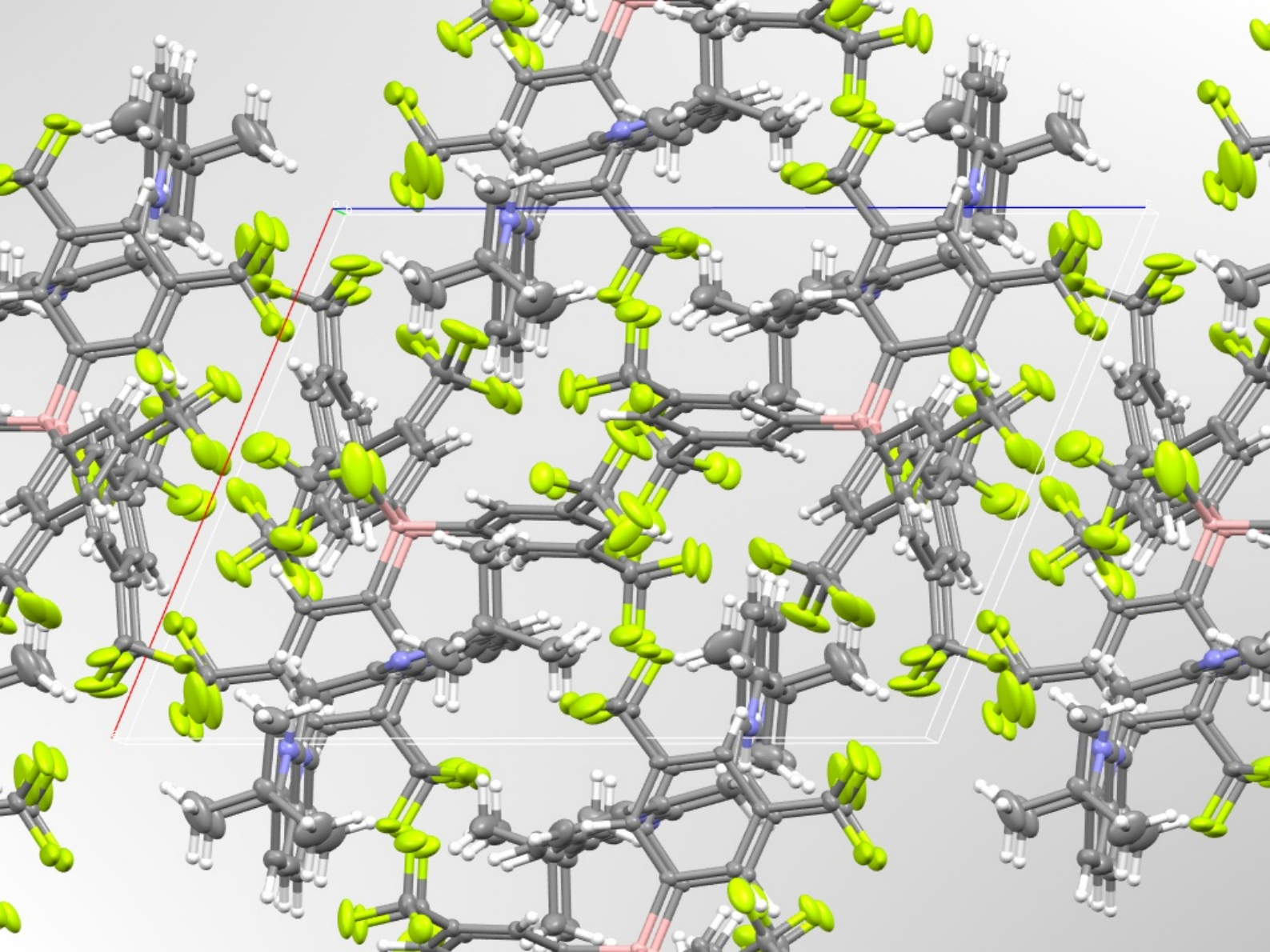

Supplement: Supplementary file 1 — Supporting Information [file CHEM-31-e02745-s002.zip › Crystal_structures/7b/packing.pdf]

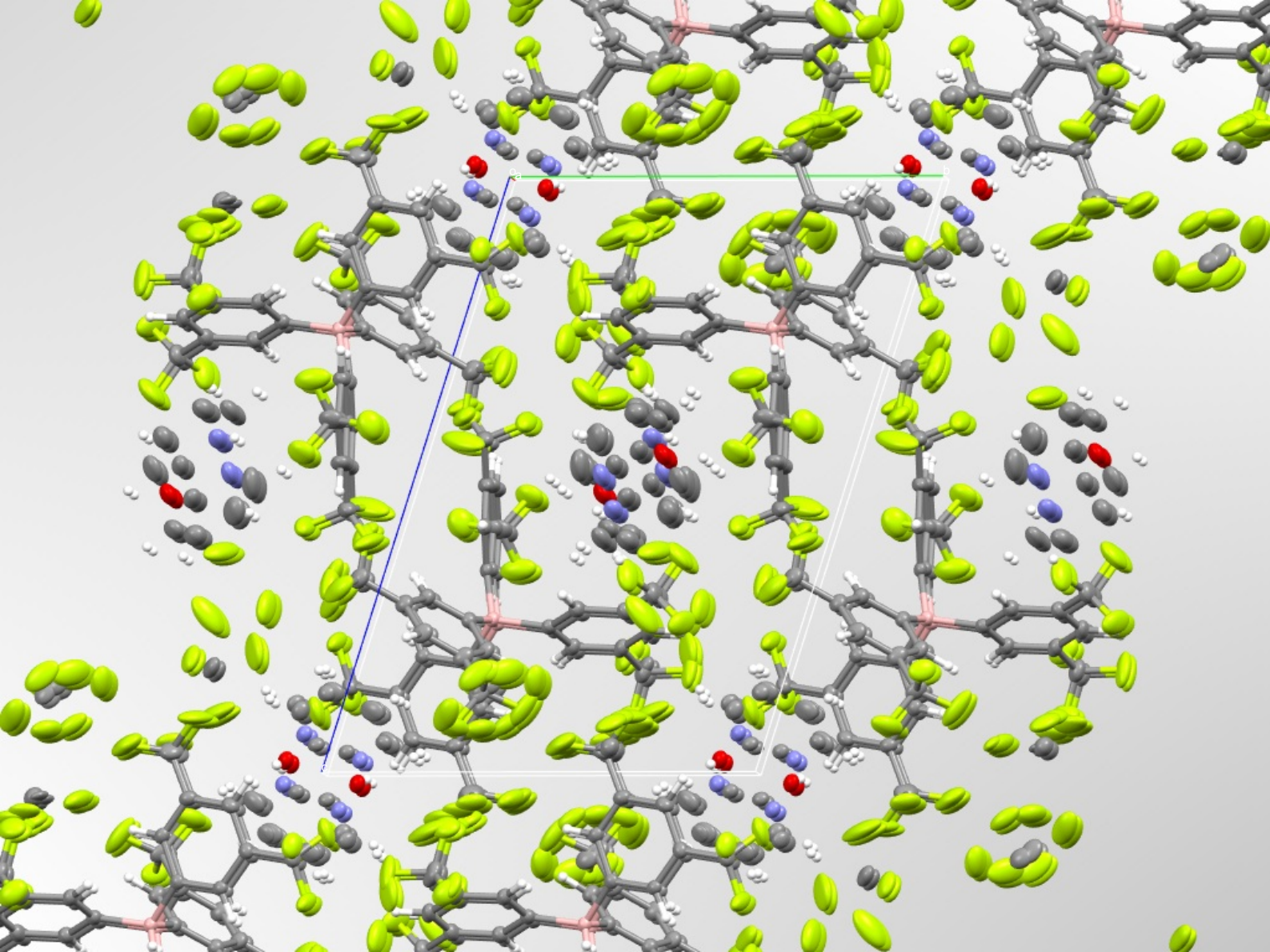

Supplement: Supplementary file 1 — Supporting Information [file CHEM-31-e02745-s002.zip › Crystal_structures/8b/packing.pdf]

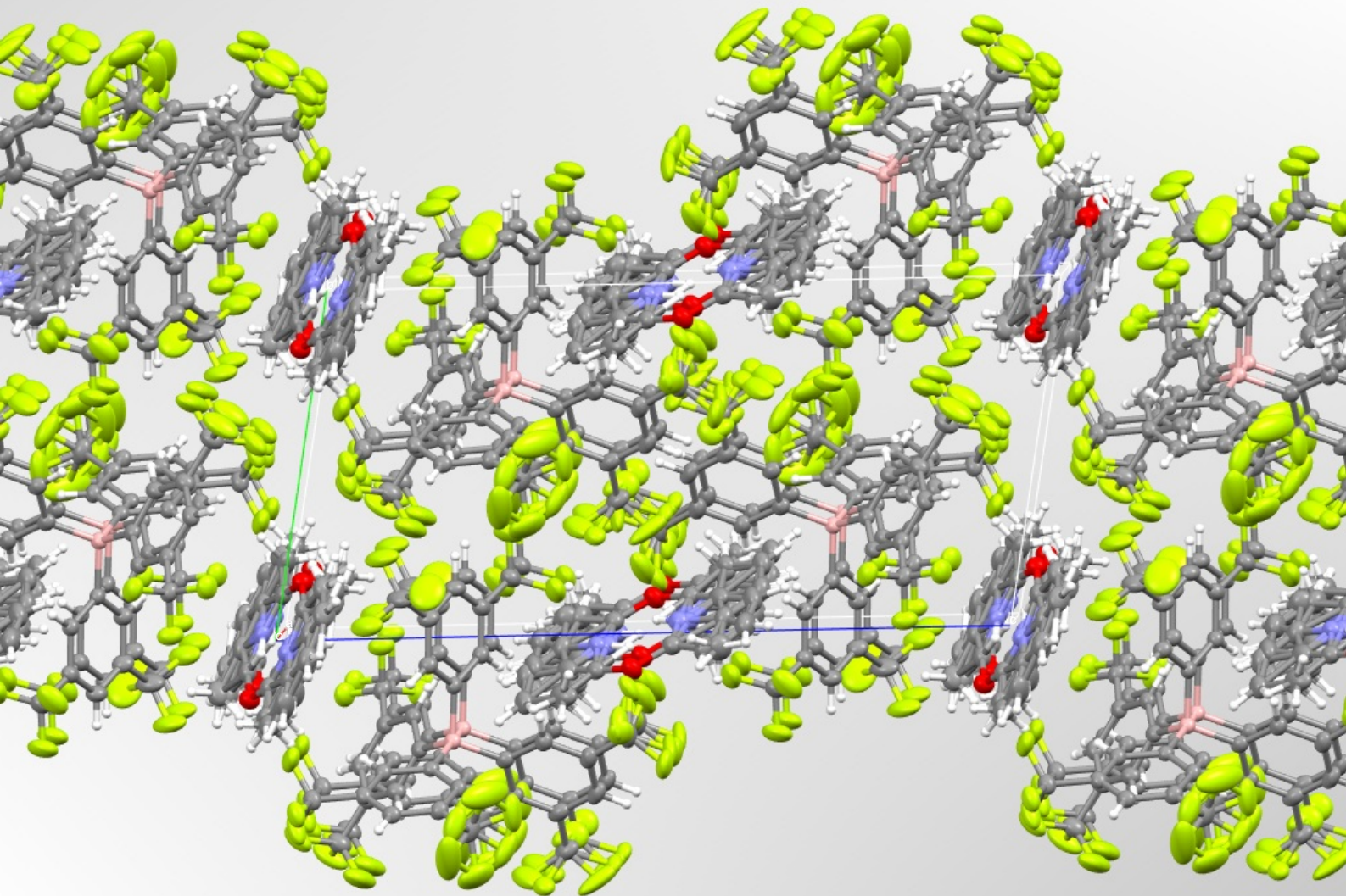

Supplement: Supplementary file 1 — Supporting Information [file CHEM-31-e02745-s002.zip › Crystal_structures/9b/packing.pdf]

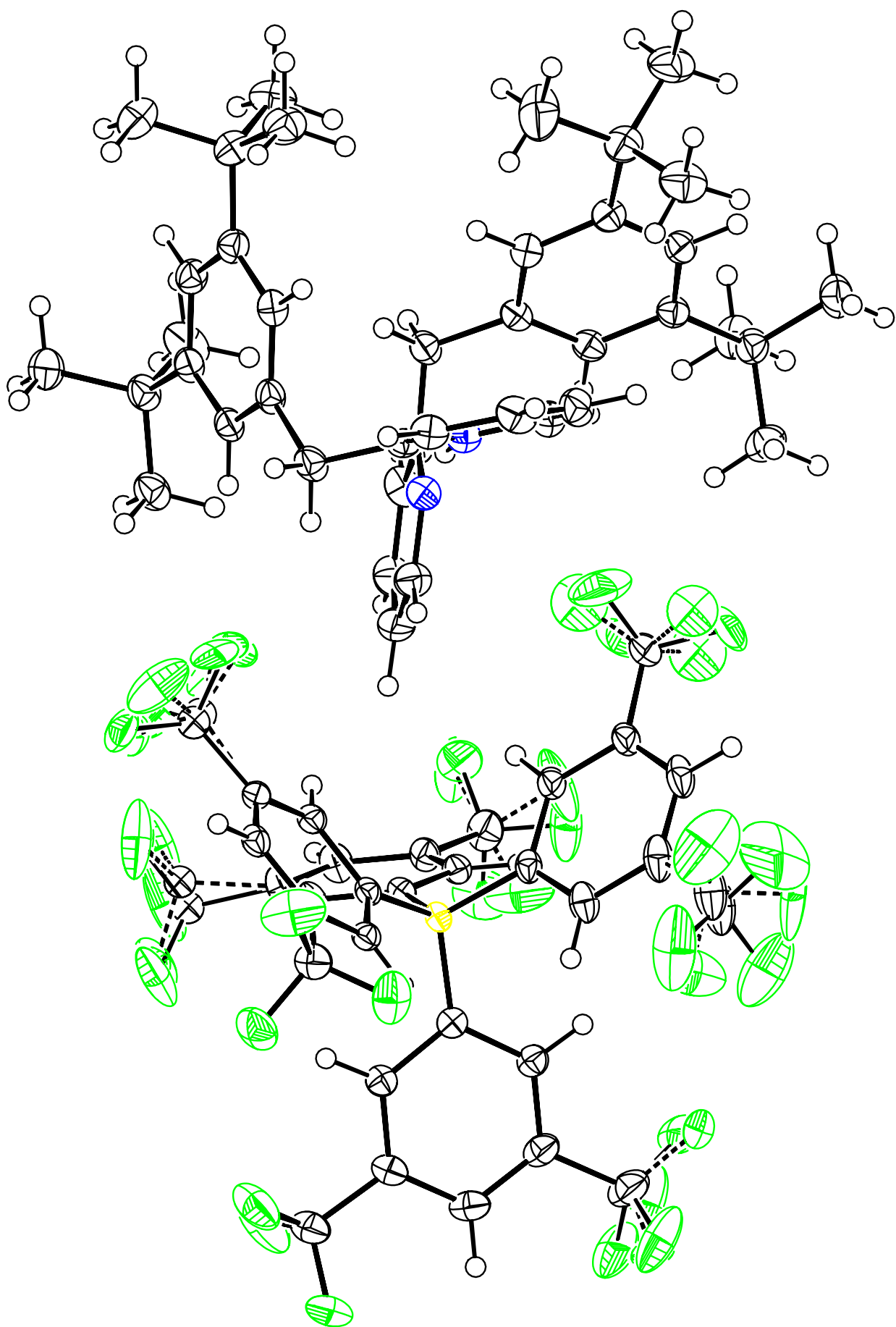

Supplement: Supplementary file 1 — Supporting Information [file CHEM-31-e02745-s002.zip › Crystal_structures/proton_bound_dimer_BHB/c280220.pdf]
