# Supplementary material for: London Dispersion versus Intramolecular Hydrogen Bond in Bis‐Pyridines: How Accurate Is DFT for Competing Noncovalent Interactions in the Condensed Phase?
Source: Chemistry. 2025 Oct 23;31(66):e02745. doi: 10.1002/chem.202502745 (PMC12648470; doi:10.1002/chem.202502745)
Supplement: Supplementary file 2 — Supporting Information [file CHEM-31-e02745-s003.pdf]

London Dispersion vs Intramolecular Hydrogen Bond in Bis-Pyridines: How Accurate Is DFT for Competing Noncovalent Interactions in the Condensed Phase?

Adélaïde Savoy,Vladimir Gorbachev, Charlotte N. Stindt, Peter Chen*

Department of Chemistry and Applied Biosciences, ETH Zürich, Zürich, 8093, Switzerland

*E-mail: peter.chen@org.chem.ethz.ch

Contents

1 Additional discussions 1
1.1 ^{13}C NMR . . . 1
1.2 Solid-state FT-IR spectra . . . 6
1.3 Additional figures . . . 12

2 Experimental procedures 14
2.1 Synthesis of precursors . . . 14
2.2 Synthesis of neutral bipyridines . . . 15
2.3 Synthesis of ^{15}N-tagged species . . . 19
2.4 General procedure for the protonation of bipyridines . . . 22
2.5 Characterisation of BArF salts . . . 22

3 NMR spectra 26
3.1 NMR spectra of precursors . . . 26
3.2 NMR spectra of neutral bipyridines . . . 28
3.3 NMR spectra of protonated bipyridines . . . 37
3.4 NMR spectra of ^{15}N tagged pyridines and bipyridines . . . 51
3.5 VT-NMR . . . 61

4 XRD 70

5 DFT 86

6 ^{1}H NMR Calculations and Conformer Analysis 94
6.1 Computational details . . . 94
6.2 Conformer space and Reference compounds . . . 95
6.3 Conformer space analyses of **7b**, **10b**, and **13b** . . . 97

7 Manual and Unsupervised Clustering 103
7.1 General notes . . . 103
7.2 Python-Based Protocol for HDBSCAN . . . 104
7.3 Influence of Parameter Selection on HDBSCAN Clustering . . . 105

8 References 111

Additional discussions

1.1 ^{13}C NMR

To illustrate the effect of ^{15}N isotopic labeling, the ^{13}C-NMR of pyridine and ^{15}N-pyridine were measured and compared, as shown in Figure 1.1. The coupling with ^{15}N generates a doublet and, overall, all chemical shift are slightly upfield from the natural abundance pyridine.

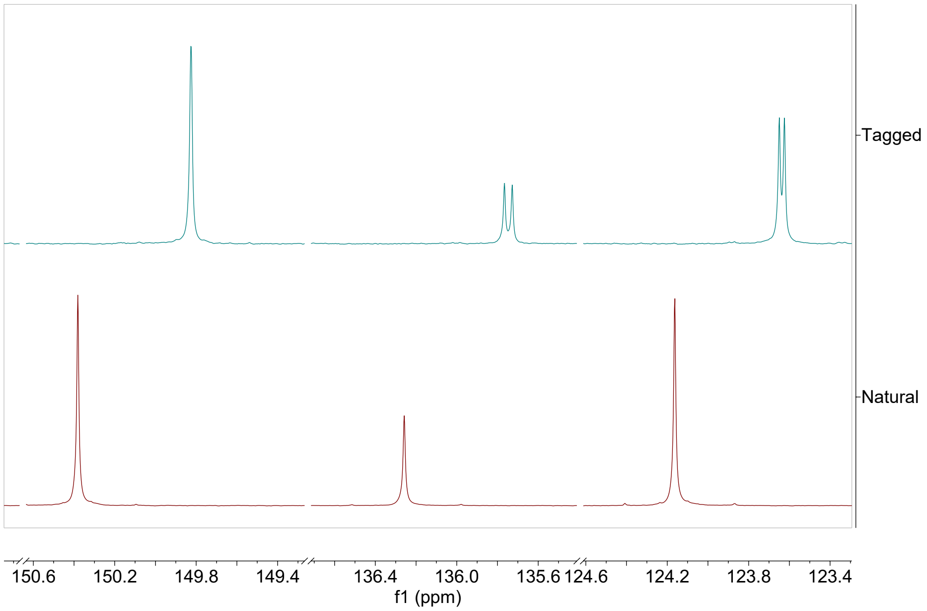

Figure 1.1: Peaks of the ^{13}C NMR corresponding to ^{15}N-pyridine (up) and pyridine at natural abundance (down) in $CDCl_3$.

In the case of **5b-t5b**, it is not possible to distinguish which peaks belong to the C-6 position, as shown in Figure 1.2 and Figure 1.3. The pair **7b-t7b** behave as expected (Figure 1.6 and Figure 1.7). It was not possible to draw any conclusion regarding the localization of the proton from these measurements.

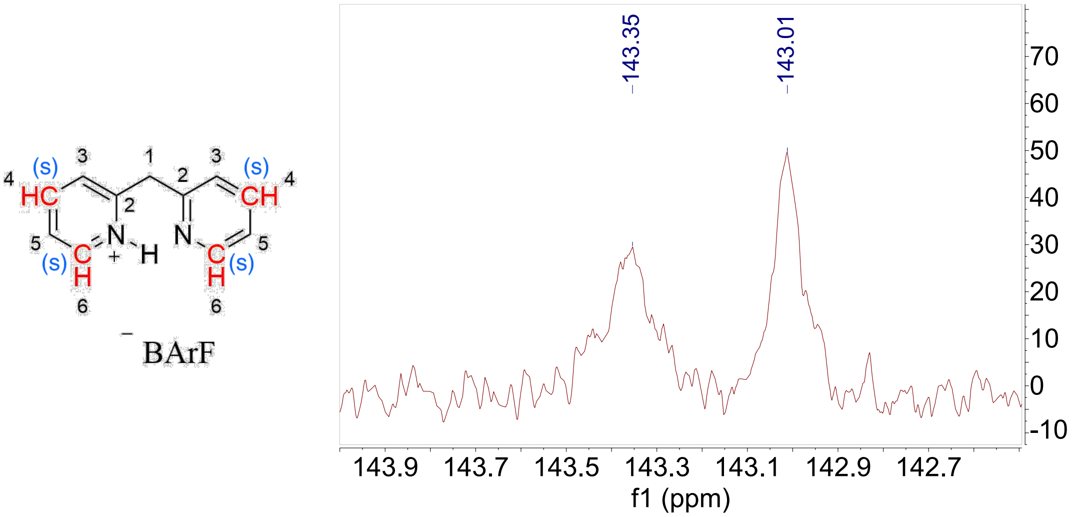

Figure 1.2: Selected region of the the ^{13}C NMR of compound **5b** in $CDCl_3$. The peaks correspond to the carbons in red with their expected multiplicity in blue.

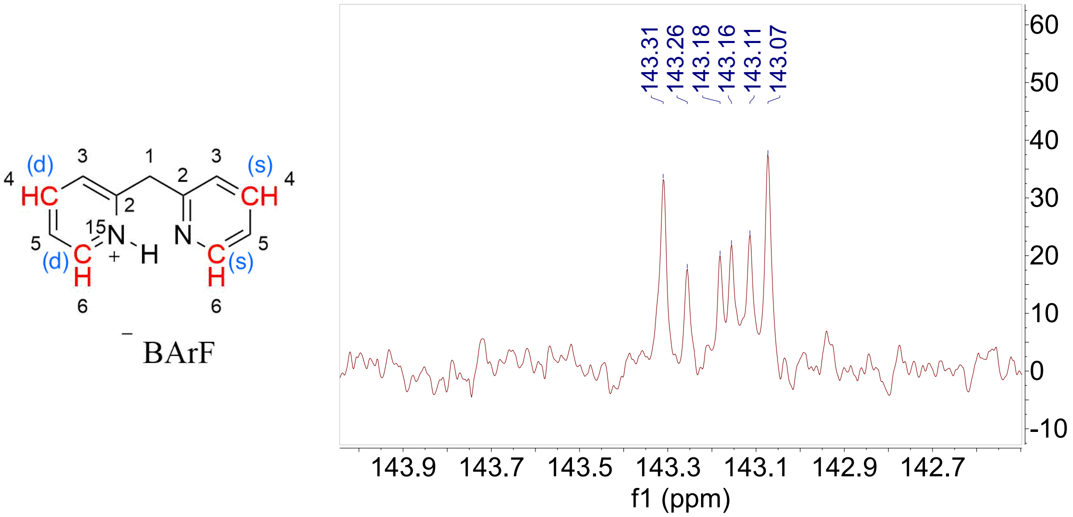

Figure 1.3: Selected region of the ^{13}C NMR of compound **t5b** in $CDCl_3$. The peaks correspond to the carbons in red with their expected multiplicity in blue.

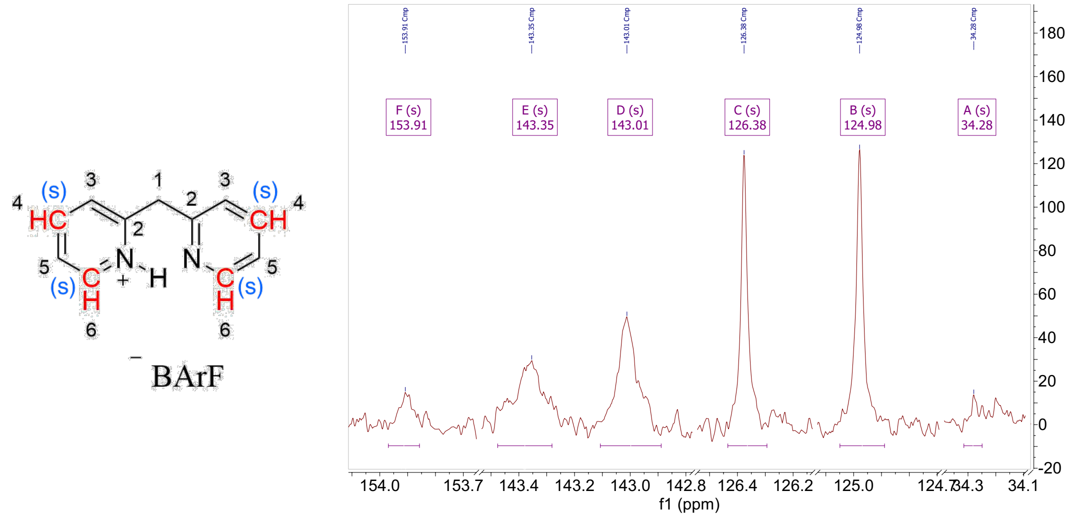

(a) Peaks of the ^{13}C NMR corresponding to compound **5b** in $CDCl_3$.

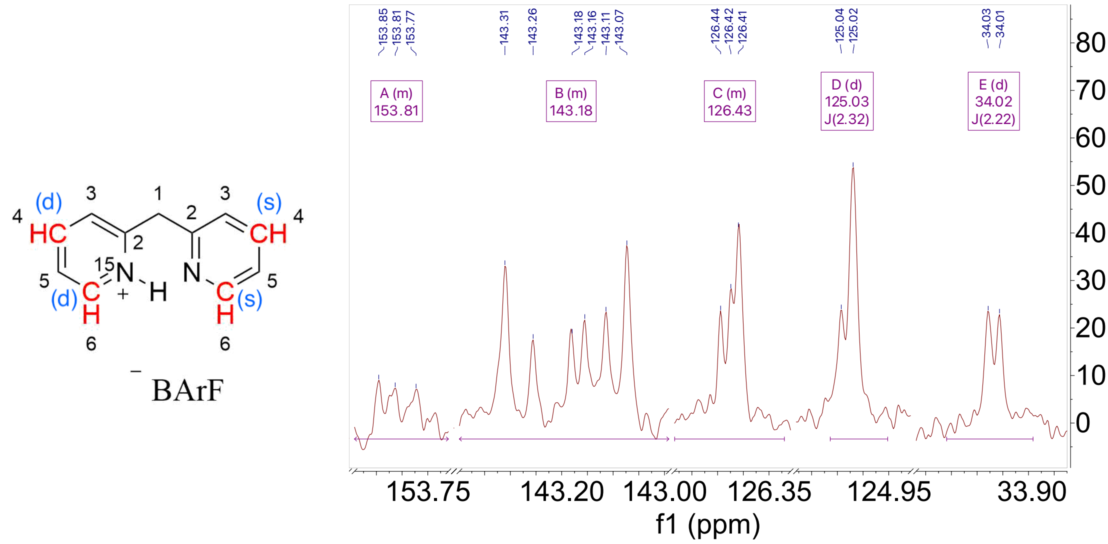

(a) Peaks of the ^{13}C NMR corresponding to compound **t5b** in $CDCl_3$.

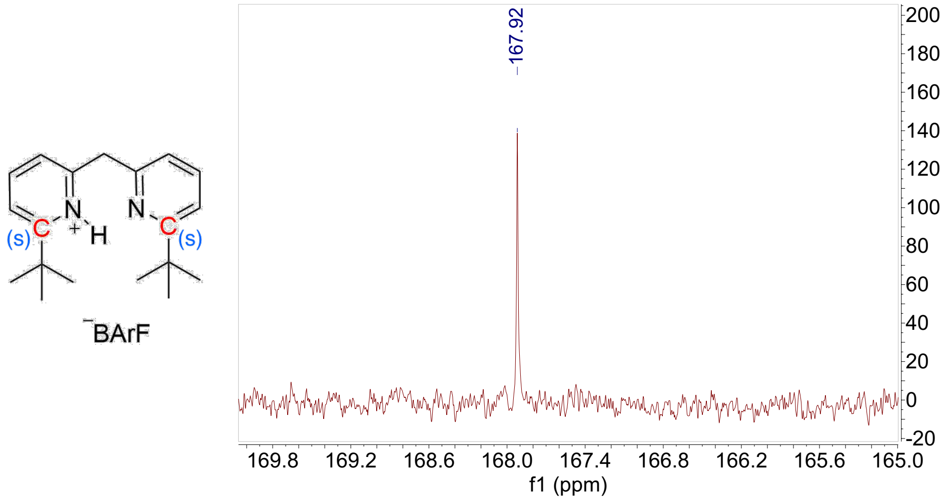

Figure 1.6: Selected region of the ^{13}C NMR of compound **7b** in $CDCl_3$. The peak correspond to the carbons in red with their expected multiplicity in blue.

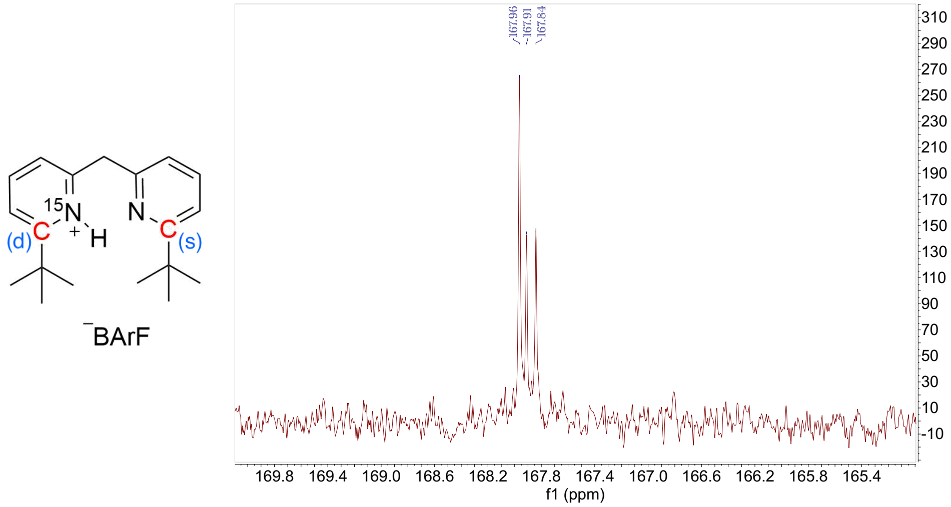

Figure 1.7: Selected region of the ^{13}C NMR of compound **t7b** in $CDCl_3$. The peaks correspond to the carbons in red with their expected multiplicity in blue.

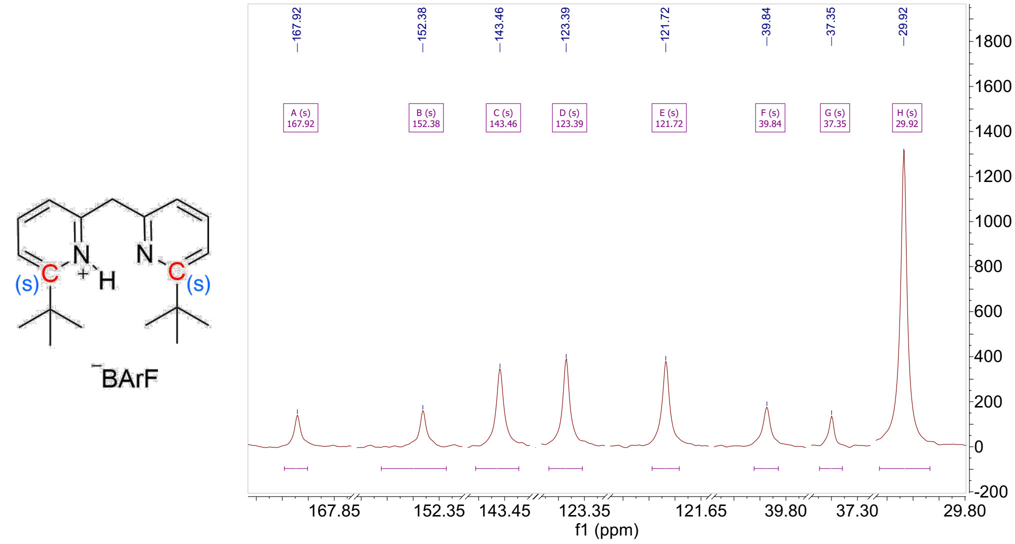

(a) Peaks of the ^{13}C NMR corresponding to compound **7b** in $CDCl_3$.

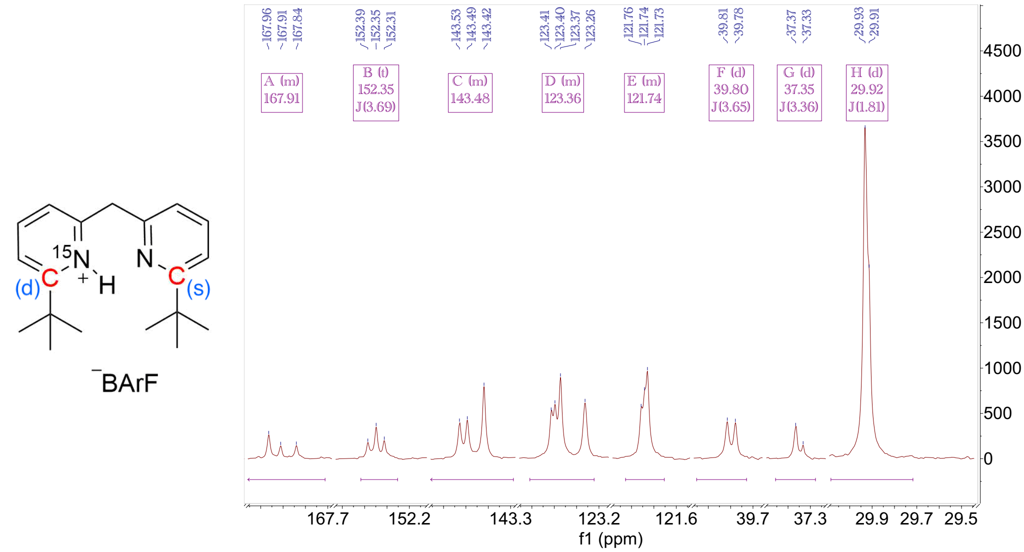

(a) Peaks of the ^{13}C NMR corresponding to compound **t7b** in $CDCl_3$.

1.2 Solid-state FT-IR spectra

The spectra were obtained using a Spectrum Two FT-IR Spectrometer from PerkinElmer with a Universal ATR crystal (Diamond). Scanning parameters are as follows: the number of scans varied between 2 and 6, with a 1 cm^{-1} step size in the range of 450-4000 cm^{-1}.

We note that the diffuse N-H--N stretch is challenging to probe experimentally via solid-state FT-IR analysis. The diffuse, redshifted, broad H-bond is not distinguishable from the baseline, in contrast to gas-phase IRMPD spectra. Therefore, we provide baseline-corrected solid-state FT-IR spectra. For correction, we used the standard procedure implemented in Origin (2019) Software. Adjacent-averaging smoothing was applied, with a smoothing window size of 3, threshold 0.05, and 10–20 points in the baseline. Initial anchor points were identified using the 2nd Derivative (zeroes) method, followed by BSpline interpolation. However, even in this case, the H-bond could not be reliably identified (see Figures 1.10-1.14).

Nevertheless, we do note some findings: compounds **1b**, **2b**, **3b**, and **4b** feature a redshifted N-H stretch, possibly due to Fermi resonance, as we reported previously for *o,o'*-disubstituted and *o*-substituted pyridiniums.[1, 2] Compounds **5b**, **6b**, **8b**, **9b**, **10b**, **11b**, and **12b** do not feature a free H-bond and are consistent with XRD analysis.

Compounds **7b** and **13b** are also consistent with their XRD structures. As the position of N-H cannot be reliably defined in XRD analysis, the FT-IR spectrum of **7b** indicates that it has only a slightly redshifted N-H and does not exhibit an effective H-bond. Compound **13b**, whose crystal structure suggests cation-π(Aryl) interaction, shows a shift typical for cation–π(Aryl) interaction, in line with our previous gas-phase studies.[1, 2]

While compound **14b** exists as an H-bonded structure in the crystal, in the powder, it presumably exists as a polymorph, with other conformations featuring a free N-H and cation-π(Aryl) interaction also accessible, as evidenced by the presence of low-intensity peaks in the 3200-3400 cm^{-1} region in the spectrum depicted in Figure 1.11, bottom.

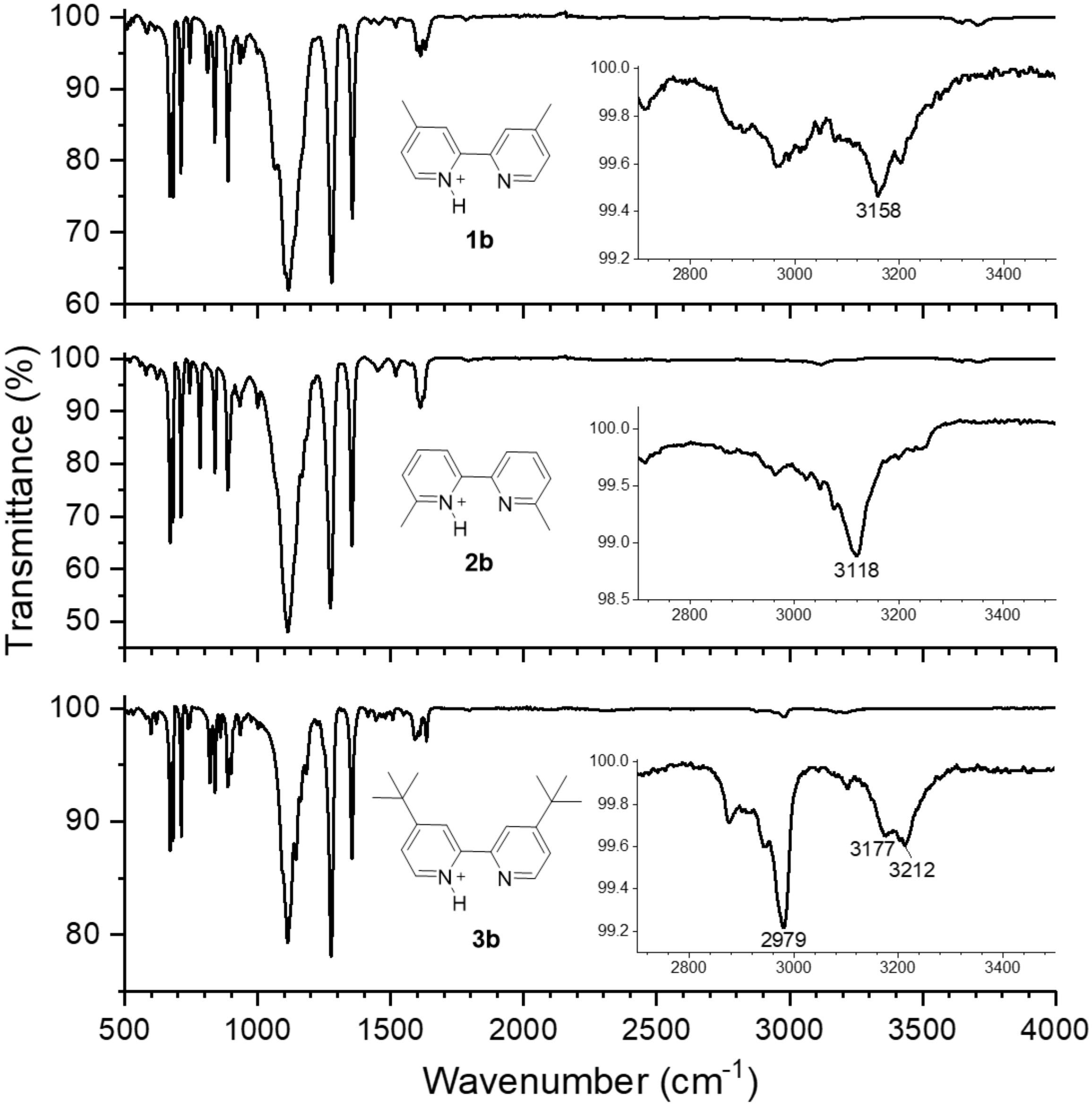

Figure 1.10: Solid-state FT-IR spectra of subgroup A compounds: **1b** (top), **2b** (middle), and **3b** (bottom). The BArF counterion is omitted for clarity. The insets highlight the zoomed-in N-H/C-H stretching region.

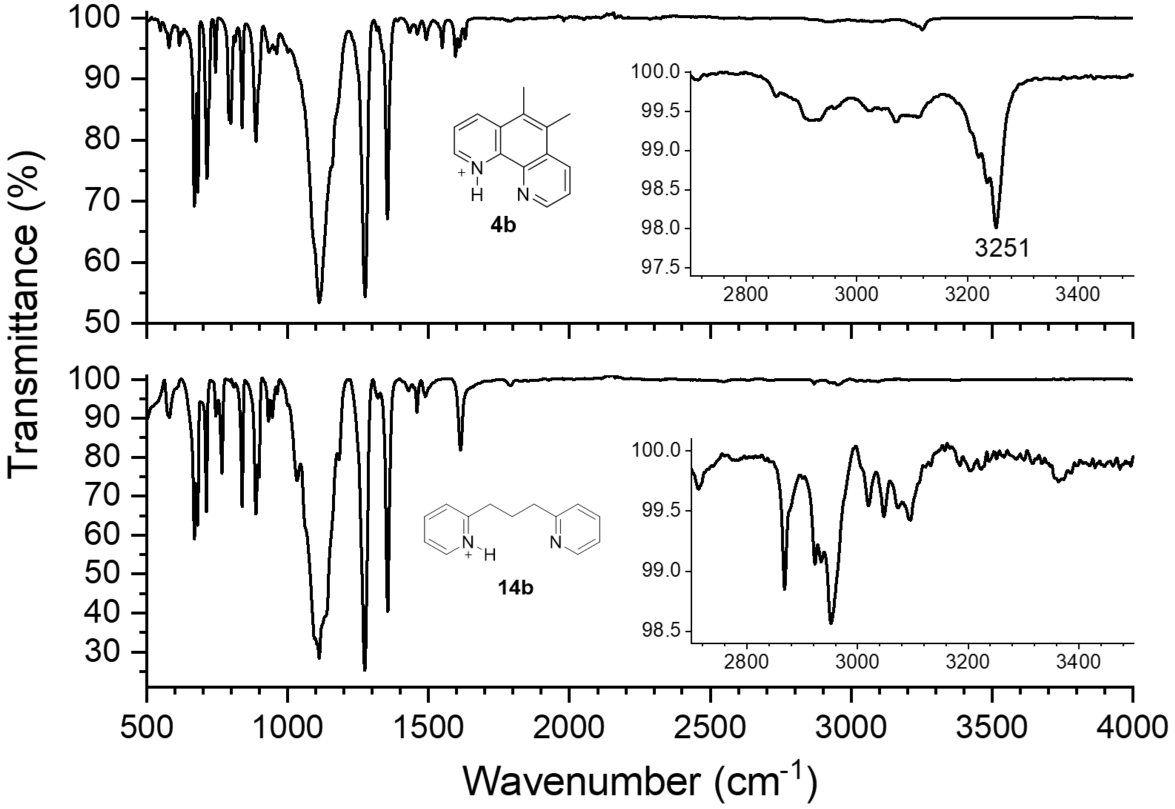

Figure 1.11: Solid-state FT-IR spectra of subgroups B and F compounds: **4b** (top) and **14b** (bottom). The BArF counterion is omitted for clarity. Although the single-crystal structure of **14b** clearly shows the H-bonded conformation, solid-state FT-IR analysis from powder suggests that other conformers with a free N-H and cation-π(Aryl) interaction are also populated in the solid form.

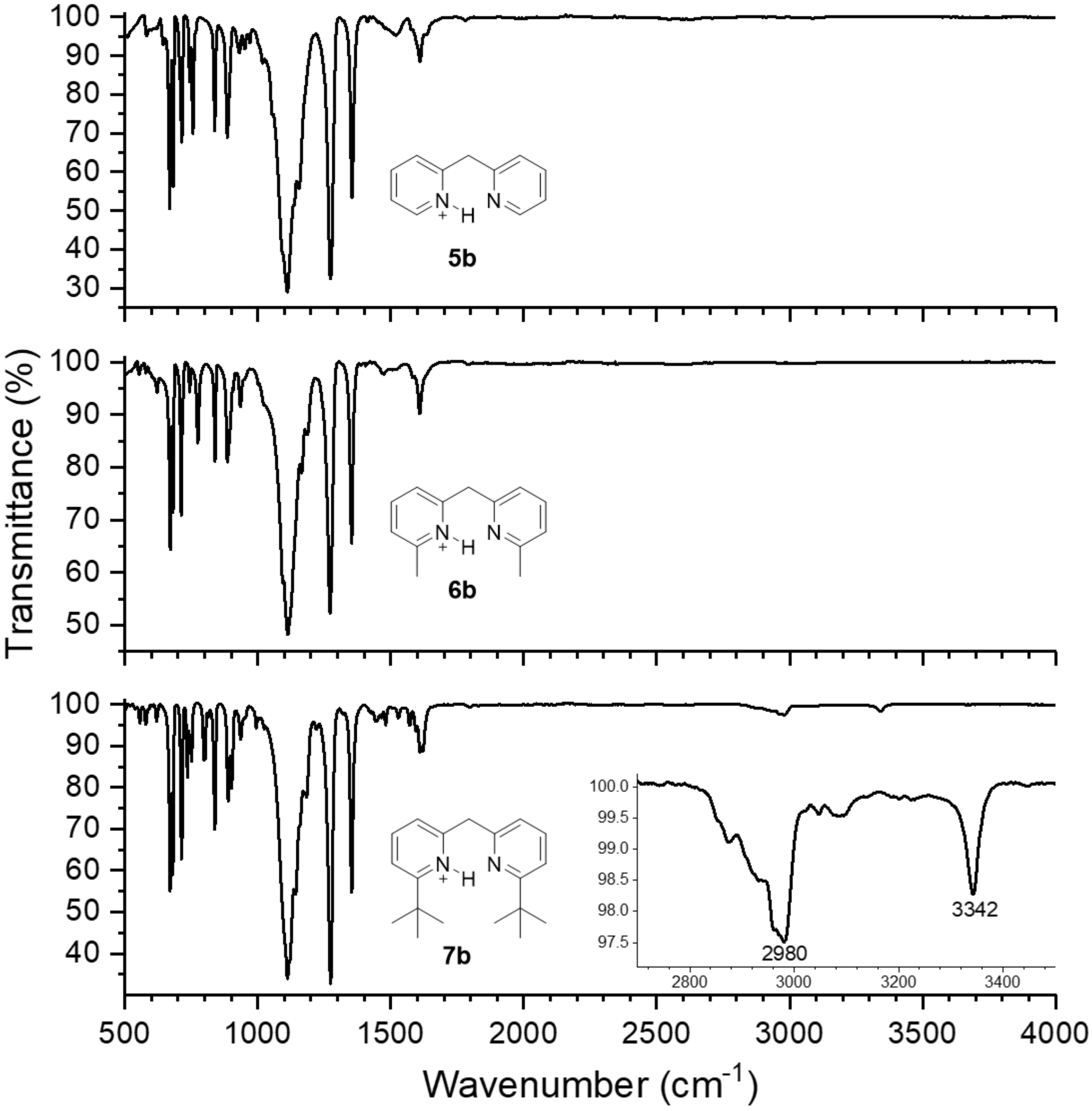

Figure 1.12: Solid-state FT-IR spectra of subgroup C compounds: **5b** (top), **6b** (middle), and **7b** (bottom). The BArF counterion is omitted for clarity. The insets highlight the zoomed-in N-H/C-H stretching region.

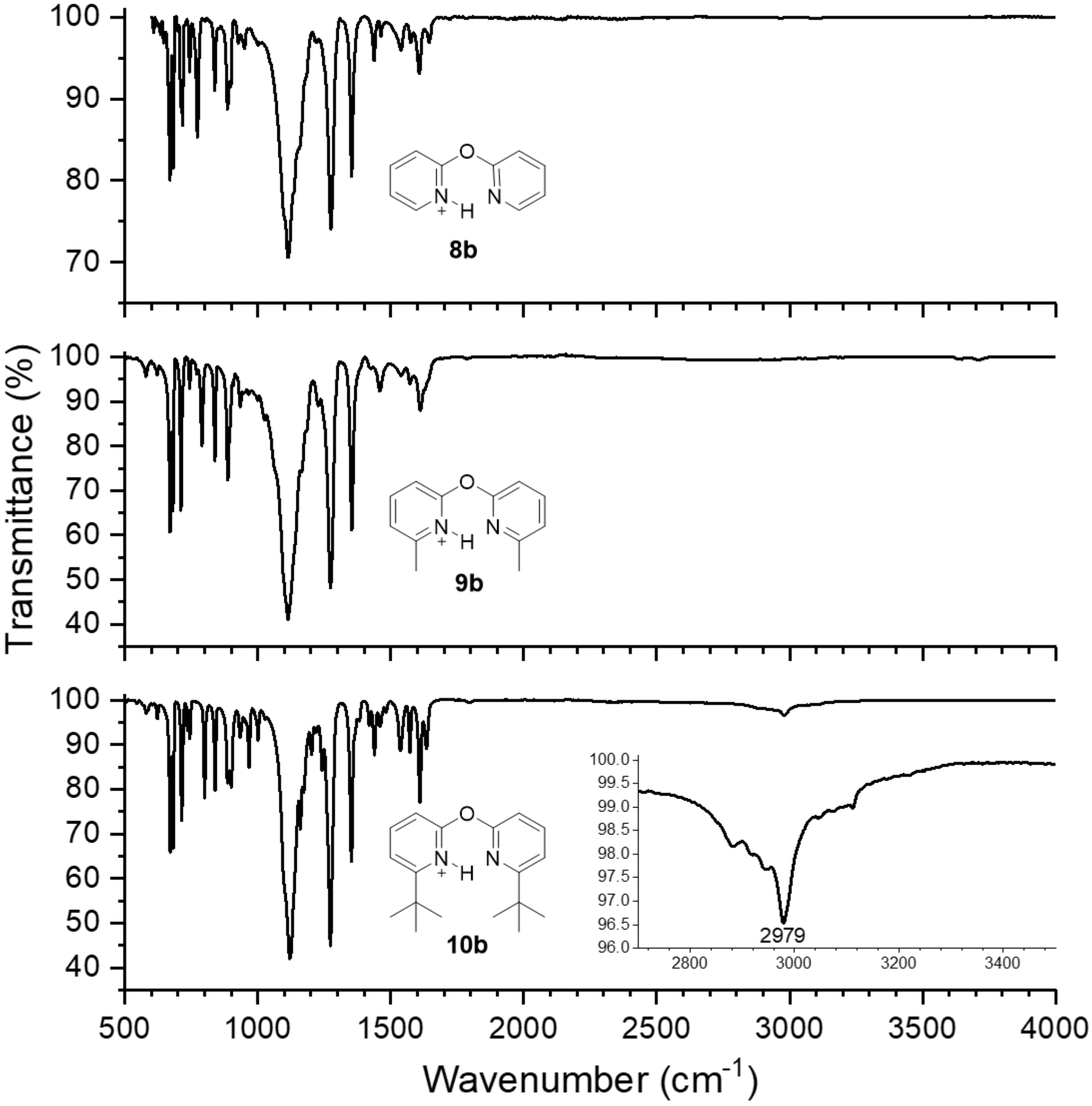

Figure 1.13: Solid-state FT-IR spectra of subgroup D compounds: **8b** (top), **9b** (middle), and **10b** (bottom). The BArF counterion is omitted for clarity. The insets highlight the zoomed-in N-H/C-H stretching region.

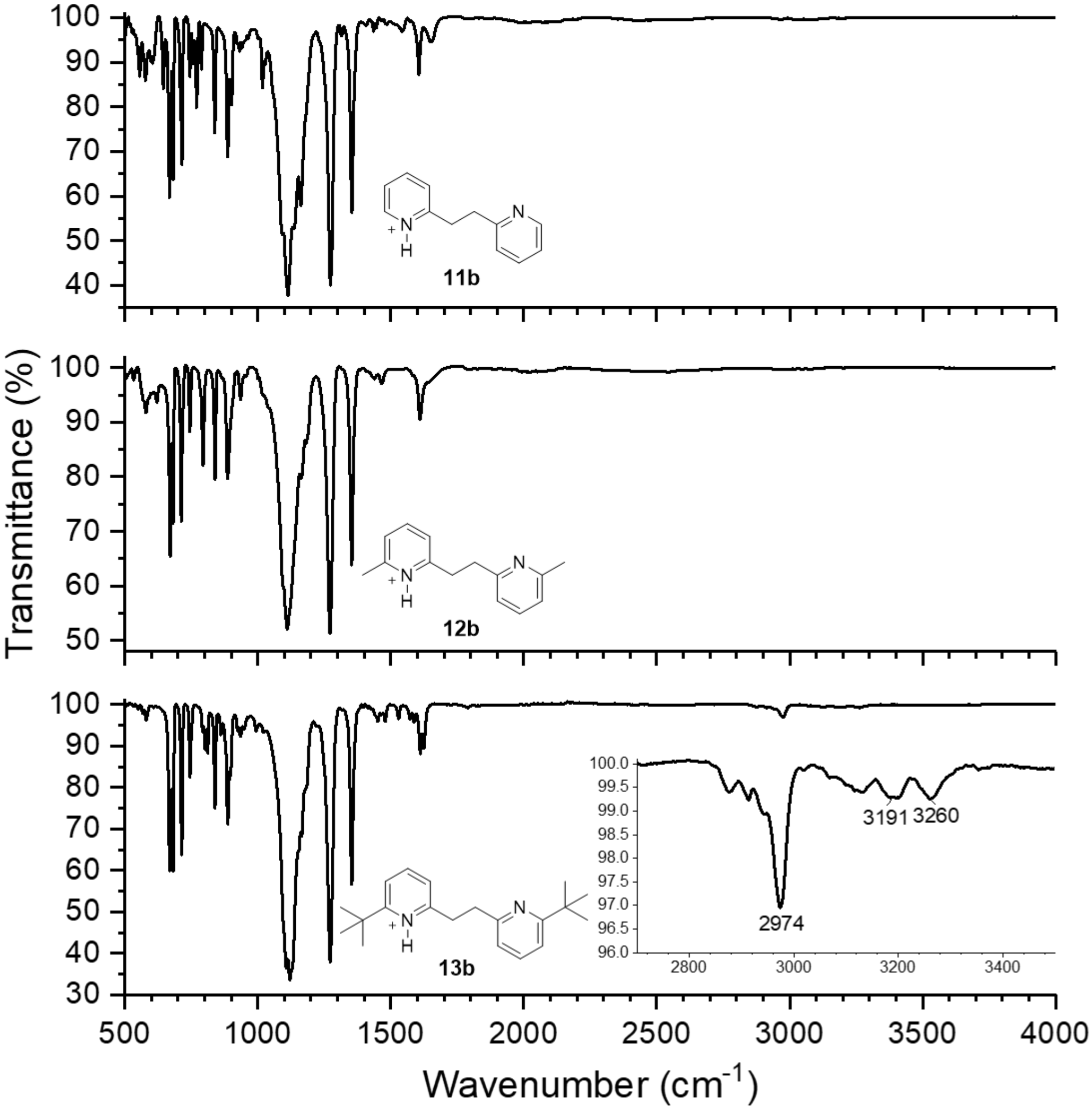

Figure 1.14: Solid-state FT-IR spectra of subgroup D compounds: **11b** (top), **12b** (middle), and **13b** (bottom). The BArF counterion is omitted for clarity. The insets highlight the zoomed-in N-H/C-H stretching region.

1.3 Additional figures

For the sake of completeness, two additional figures are provided here and illustrate the comparison between the calculated N-H-N angle and the measured ^{1}H chemical shift, similarly to the Figure 3 from the main text. The structural outliers **7b** & **13b** still lay off the linear correlation. Compound **6b** could also be removed from the correlation, sililarly to the Figure 2 in the main text, as its chemical shift may be underestimated.

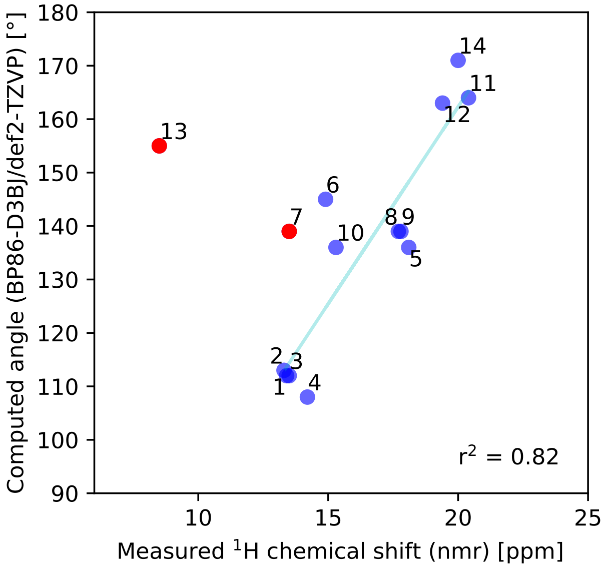

Figure 1.15: Calculated (BP86-d3bj/def2-TZVP) vs experimentally measured (VT-NMR) ^{1}H chemical shift [ppm] of the BArF salts. Compound 7b and 13b (in red) are excluded from the statistical correlation coefficient r2.

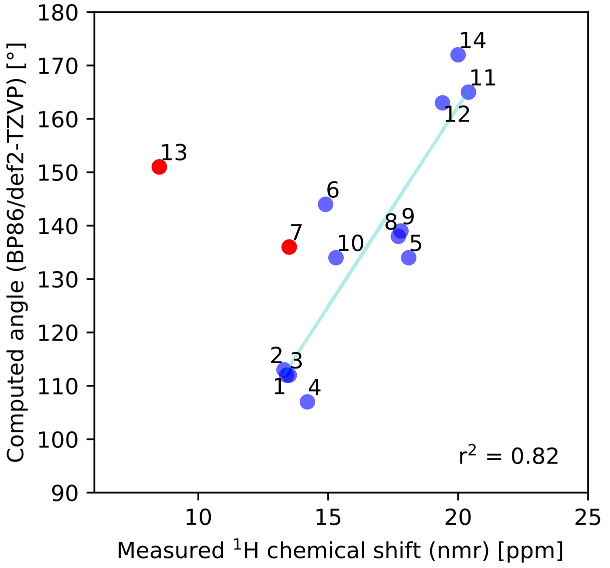

Figure 1.16: Calculated (BP86/def2-TZVP, without D3 correction) vs experimentally measured (VT-NMR) ^{1}H chemical shift [ppm] of the BArF salts. Compound 7b and 13b (in red) are excluded from the statistical correlation coefficient r2.

Experimental procedures

^{15}N-pyridine (99 atom% ^{15}N) was acquired from Sigma-aldrich. 2-bromo-6-methylpyridine was distilled prior to use. Lutidine was purchased dried and stored in a glovebox. Dry solvents (THF, dichloromethane, MeCN, Et_2O and hexanes) were obtained by distillation over drying agents. Degassing of solvents, when mentioned, was done by bubbleing agron for a minimum of 20 min. Water was systematically deionized. Other chemicals were purchased from commercial suppliers and used without further purification.

NMR Spectra were recorded on Bruker spectrometers. The field strengths are indicated for each measurement. Data are reported as follows: chemical shift, multiplicity (s– singlet, d– doublet, t– triplet, q– quartet, m– multiplet, td– triplet of doublets, ddd doublet of double doublets), coupling constants, integration.

High-resolution mass spectrometry (HRMS) was performed on a Bruker Daltonics maXis ESI-QTOF.

2.1 Synthesis of precursors

Synthesis of 2-bromo-6-(*tert*-butyl)pyridine

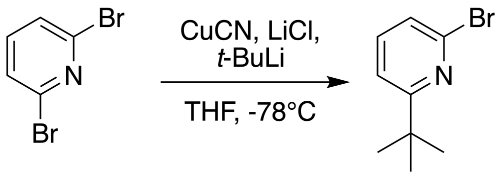

This procedure was adapted from literature.[3] LiCl (1.616 g, 38 mmol, 2.5 equiv., dried overnight at 130°C under reduced pressure), CuCN (1.536 g, 17 mmol, 1.1 equiv.) and THF (110 mL, dry) were inserted in an oven-dried Schlenk tube under argon atmosphere. The mixture was cooled to -78°C before addition of *t*-BuLi (11.5 mL, 1.7 M in pentane, 20 mmol, 1.3 equiv.). The mixture was stirred for 30 min before dropwise addition of a solution of 2,6-dibromopyridine (3.56 g 15 mmol, 1.0 equiv., in 20 mL of dry THF). The mixture was stirred for 30 min and then at room temperature overnight. The black mixture was diluted with Et_2O (200 mL) and washed with ammonia (3x200 mL, 6% (v/v) solution in water). The organic phase was dried over Na_2SO_4, filtrated and concentrated under reduced pressure yielding a yellow liquid (2.674 g, 83% yield). The product was used without further purification. HRMS (ESI+): [M+H]+ calculated 212.0069; found 212.0071. ^{1}H-NMR (400 MHz, $CDCl_3$): δ/ppm= 7.44 (dd, J= 7.97, 7.53 Hz, 2H), 7.27 (q, J= 0.81 Hz ,2H), 7.25 (dd, J= 1.62, 0.82 Hz, 2H), 1.34 (s, 18H). ^{13}C-NMR (101 MHz, $CDCl_3$): δ/ppm= 171.24, 141.20, 138.47, 124.96, 117.80, 37.62, 29.99.

Synthesis of 2-(*tert*-butyl)-6-methylpyridine

This procedure was adapted from literature.[3] $ZnCl_2$ (2.556 g, 20 mmol, 1.5 equiv.) was dissolved in THF (50 mL, dry) in an oven-dried Schlenk flask under argon atmosphere. The solution was cooled down to -78°C before dropwise addition of MeLi (13 mL, 20.8 mmol, 1.6 M in hexanes, 1.6 equiv.). The mixture was stirred for 30 min. $Pd(PPh_3)_4$ (300 mg, 0.25 mmol, 2mol%) was added under argon

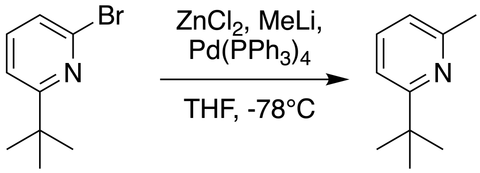

counterflow. 2-bromo-6-(*tert*-butyl)pyridine (2779 mg, 13 mmol, 1.0 equiv.) was added dropwise. The mixture was stirred overnight at room temperature. The mixture was diluted with Et_2O (180 mL) and washed with NH_4Cl (2x150 mL, sat. aqueous solution) and brine (150 mL). The organic phase was dried over Na_2SO_4, filtrated and concentrated under reduced pressure (not lower than 50 mbar to avoid evaporation of the product). After filtration, the product was isolated by silica gel column chromatography, using a gradient from hexanes to dichloromethane to yield a colorless liquid (955 mg, 49% yield). HRMS (ESI+): [M+H]+ calculated 150.1277; found 150.1277. ^{1}H-NMR (400 MHz, $CDCl_3$): δ/ppm= 7.47 (t, J= 7.73 Hz, 2H), 7.11 dd, J= 7.86, 0.43 Hz ,2H), 6.92 (dd, J= 7.60, 0.43 Hz, 2H), 2.52 (s, 3H), 1.35 (s, 18H). ^{13}C-NMR (101 MHz, $CDCl_3$): δ/ppm= 168.65, 157.03, 135.11, 119.91, 115.62, 37.26, 30.24, 24.79.

2.2 Synthesis of neutral bipyridines

1a, 2a, 3a, 4a and 5a are commercially available and were used without any further purification.

Synthesis of bis(6-(methyl)pyridin-2-yl)methane 6a

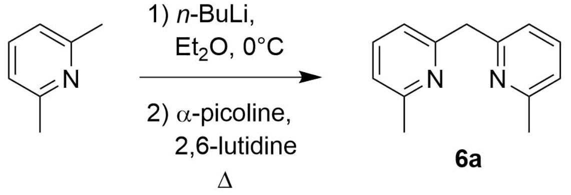

The procedure was adapted from literature.[4] 2,6-lutidine (2.4 mL, 20.6 mmol, 1.0 equiv., dry) and diethylether (20 mL, dry) were introduced in an oven-dried Schlenk tube under Argon atmosphere and cooled to 0°C. *n*-BuLi (14.5 mL, 1.6 M in hexanes, 23.2 mmol, 1.1 equiv.) was added dropwise. The mixture was stirred for 15 min at 0°C and then for 30 min at room temperature. The solvent was removed under reduced pressure, while avoiding contact with air. The resulting orange residue was diluted with 2,6-lutidine (5 mL, dry) before the addition of α-picoline (2 mL, 20.3 mmol, 1.0 equiv.). The mixture was stirred for 1 h at room temperature and at reflux for 4 h before letting it cool down to room temperature overnight. Methanol (12 mL) and water (60 mL) were slowly added. The product was extracted with toluene (3x75 mL). The combined organic phase was dried over $MgSO_4$, filtrated and concentrated under reduced pressure. The crude product was purified by distillation under reduced pressure followed by two successive silica gel column chromatography using EtOAc/hexanes (1:4 to 1:0) and methanol/EtOAc (1:10) as eluent respectively. After drying under high vacuum, a white solid was obtained (215 mg, 5.3% yield). HRMS (ESI+): [M+H]+ calculated 199.123; found 199.1231. ^{1}H-NMR (400 MHz, $CDCl_3$): δ/ppm= 7.46 (t,J= 7.69 Hz, 2H), 6.98 (d, J= 7.52 Hz ,4H), 4.29 (s, 2H), 2.55 (s, 6H). ^{13}C-NMR (101 MHz, $CDCl_3$): δ/ppm= 158.92, 157.91, 136.75, 120.94, 120.41, 47.33, 24.52.

Synthesis of bis(6-(*tert*-butyl)pyridin-2-yl)methane 7a

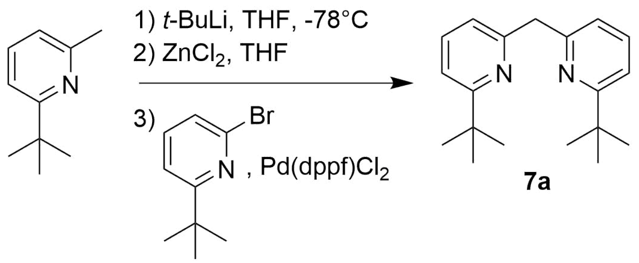

The synthesis was adapted from literature.[3] 2-(*tert*-butyl)-6-methylpyridine (301 mg, 2.02 mmol) and THF (12 mL, dry) were inserted in an oven-dried Schlenk tube under argon atmosphere. The solution was cooled to -78°C and *t*-BuLi (1.7 min pentane, 1.25 mL, 2.21 mmol, 1.1 eq) was added dropwise. The solution was stirred at -78°C for 30 min and subsequently at room temperature for 2 h. The solution was cooled to -78°C and an anhydrous solution of $ZnCl_2$ (398 mg, 2.92 mmol, 1.5 equiv.) in THF (6 mL) was added. 2-bromo-6-(*tert*-butyl)pyridine (417 mg, 1.95 mmol, 1,0 equiv.), Pd(dppf)CL_2 (31.9 mg, 0.04 mmol, 2 mol%) and THF (10 mL, dry) were inserted into an oven-dried 3-neck flask equipped with a condenser. The mixture was heated to reflux. The zinc reagent solution was added dropwise via cannula. The mixture was stirred at reflux overnight then at room temperature for 24 h. The mixture was diluted with Et_2O (30 mL) and was washed with saturated aqueous NH_4Cl (2 x 30 mL, sat. aq. solution) and brine (30 mL). The organic phase was extracted with HCl (4 x 20 mL, 1 M aq. solution). The combined acidic aqueous phase was washed with Et_2O (2 x 30 mL) and basified with NaOH (1 M aqueous solution). The basic aqueous phase was extracted with dichloromethane (3 x 30 mL) and the combined organic phase was dried over anhydrous $MgSO_4$, filtered and concentrated under reduced pressure. The crude product was filtered on a silica gel pad using ethyl acetate/hexanes (1:2) as eluent. The product was obtained as a pale yellow liquid (320 mg, 58% yield). HRMS (ESI+): [M+Na]+ calculated 283.2169 ; found 283.2171. ^{1}H-NMR (300 MHz, $CDCl_3$): δ/ppm= 7.47(t, J= 7.74 Hz, 2H), 7.12 (d, J= 7.84 Hz, 2H), 7.04 (dd, J= 7.67, 0.93 Hz, 2H), 4.29 (s, 2H), 1.35 (s, 18H). ^{13}C-NMR (101 MHz, $CDCl_3$): δ/ppm= 168.52, 158.60, 136.19, 120.12, 119.91, 116.05, 115.63, 47.82, 37.37, 30.21.

Synthesis of 2,2'-oxydipyridine 8a

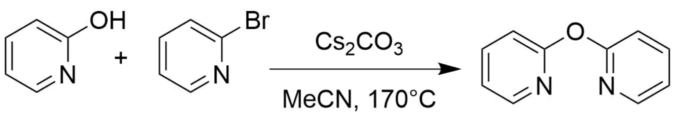

8a

The procedure was adapted from literature.[5] 2-hydroxypyridine (365 mg, 3.85 mmol, 1.4 equiv.) and Cs_2CO_3 (2.5 g, 7.70 mmol, 2.9 equiv.), 2-bromopyridine (0.26 mL, 2.67 mmol, 1.0 equiv.) and acetonitrile (18 mL, dry) were inserted in a pressure reactor under argon atmosphere,. The mixture was heated to 170°C for 5 days. After letting the mixture to cool down to room temperature, it was filtered and concentrated under reduced pressure to yield a brown oil. The crude product was purified by silica gel column chromatography using ethyl-acetate/hexanes (1:1) as eluent to yield a yellow liquid (50 mg, 11% yield). ^{1}H-NMR (300 MHz, $CDCl_3$): δ/ppm= 8.30 (ddd, J= 4.95, 2.03, 0.81 Hz, 2H), 7.76 (ddd, J= 8.17, 7.29, 2.03 Hz, 2H), 7.13-7.06 (m, 4H).^{13}C-NMR (101 MHz, CDCl3): δ/ppm= 161.95, 148.12, 139.59, 119.95, 114.02.

Synthesis of 6,6'-oxybis(2-methylpyridine) 9a

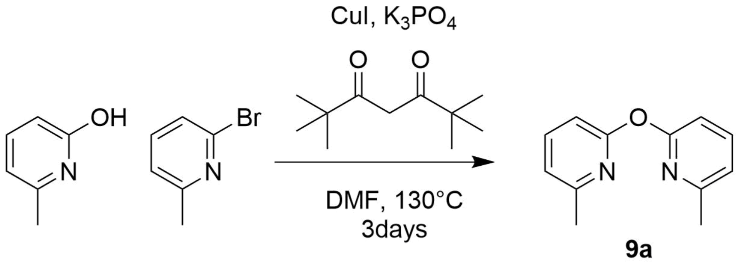

The procedure was adapted from literature.[6] K_3PO_4 (6.24 g, 29.4 mmol, 2.9 equiv., anhydrous) and CuI (773 mg, 4.1 mmol, 0.4 equiv.) were inserted in an oven-dried Schlenk tube and dried under high vacuum for two days. 2,2,6,6-tetramethylheptane-3,5-dione (2.6 mL, 12.4 mmol, 1.2 equiv.) and DMF (25 mL, dry and degassed) were added under argon atmosphere. The mixture was stirred for 10 min before the addition of 2-bromo-6-methylpyridine (1.15 mL, 10.1 mmol, 1.0 equiv.) and 6-methyl-2-pyridone (1092 mg, 10.0 mmol, 1.0 equiv.). The mixture was heated to 130°C for 3 days under argon atmosphere. After cooling down to room temperature, the mixture was diluted with water (50 mL) and DMF (25 mL) and filtrated. The product was extracted with dichloromethane (3x50 mL). The combined organic phase was washed with water (3x50 mL) and concentrated under reduced pressure. The black mixture was then acidified with HCl (65 mL, 0.1 M in water), filtrated and subsequently neutralized with NaOH (15 mL, 1 M in water). After extraction with dichloromethane (3x100 mL), the combined organic phase was dired over Na_2SO_4, filtrated and concentrated under reduced pressure to yield a brown oil (838.8 mg, 42% yield). HRMS (ESI+): [M+H]+ calculated 201.1022; found 201.1021. ^{1}H-NMR (400 MHz, $CDCl_3$): δ/ppm= 7.60 (dd, J= 8.12, 7.41 Hz, 2H), 6.93 (d, J= 7.39 Hz, 2H), 6.82 (dd, J= 8.08, 0.79 Hz, 2H), 2.46 (s, 6H). ^{13}C-NMR (101 MHz, $CDCl_3$): δ/ppm= 161.27, 157.57, 139.54, 119.15, 110.38, 24.18.

Synthesis of 6,6'-oxybis(2-(*tert*-butyl)pyridine) 10a

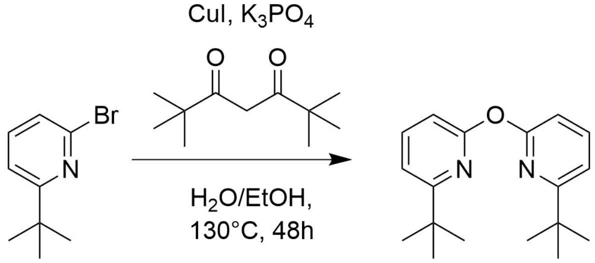

The procedure was adapted from literature.[6] A small dried vial under inert atmosphere topped with a rubber septum was charged with CuI (83.5 mg, 0.44 mmol, 0.44 equiv., anhydrous), K_3PO_4 (1213 mg, 5.7 mmol, 5.6 equiv., anhydrous), 2,2,6,6-tetramethyl-3,5-heptanedione (0.130 mL, 115 mg, 0.62 mmol, 0.62 equiv.), 2-bromo-6-(*tert*-butyl)pyridine (0.335 mL, 433 mg , 2.0 mmol, 2.0 equiv.), water (2 mL, degassed) and EtOH (2 mL, degassed). The septum cap was replaced by a regular cap under an argon blanket. The mixture was stirred at 130°C for 3 days. After cooling down to room temperature, the reaction mixture was diluted with dichloromethane (25 mL) and water (25 mL). The organic phase was separated and washed with NH_4Cl (3 x 20 mL, sat. aq. solution) and HCl (3 x 25 mL, 0.1 M solution in water). The organic phase was dried over Na_2SO_4, filtrated

and concentrated under reduced pressure. The crude product was purified by filtration over a silica pad using hexanes/dichloromethane/triethylamine (30/20/1) as eluent. The product was obtained as a pale yellow oil (65 mg, 18% yield). The product was used without further purification. HRMS (ESI+): [M+H]+ calculated 285.1961; found 285.1957. ^{1}H-NMR (400 MHz, $CDCl_3$): δ/ppm= 7.63 (dd, J= 8.11, 7.60 Hz, 2H), 7.08 (d, J= 7.60, 0.72 Hz, 2H), 6.85 (d, J= 8.10, 0.76 Hz, 2H), 1.29 (s, 18H).^{13}C-NMR (101 MHz, $CDCl_3$): δ/ppm= 168.76, 161.24, 139.21, 114.50, 110.47, 30.12.

Synthesis of 1,2-bis(2-pyridyl)ethane 11a

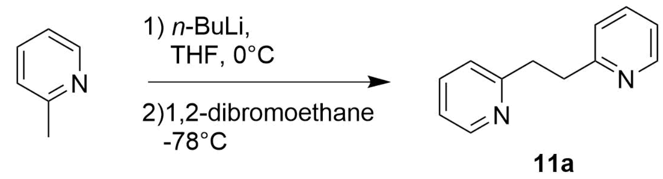

The procedure was adapted from literature.[7] α-picoline (230 μL, 2.6 mmol, 1.0 equiv.) and THF (12 mL, dry) were inserted in an oven-dried Schlenk tube under argon atmosphere. The solution was cooled down to 0°C. n-BuLi (4 mL, 6.4 mmol, 2.5 equiv., 1.6 M in hexanes) was added dropwise. The mixture was stirred for 15 min at 0°C and for 90 min at room temperature. The solution was then cooled down to -78°C before addition of 1,2-dibromoethane (180μL, 2.1 mmol, 0.8 equiv.). The mixture was stirred for 20 min at -78°C, 15 min at 0°C and 3 h at room temperature. The solution was diluted with Et_2O (50 mL) and washed with NH_4Cl (2x50 mL, sat. aq. solution). The organic phase was extracted with HCl (3x50 mL, 0.1 M in water). The combined acidic aqueous phase was basified with NaOH (4 mL, 5 M in water) and extracted with dichloromethane (3x50 mL). The combined organic phase was dried over Na_2SO_4, filtrated and concentrated under reduced pressure. The product was purified over silica gel column chromatography using successively EtOAc, dichloromethane, EtOH and MeOH as eluent. The fractions containing the product were combined and concentrated under reduce pressure yielding a yellow liquid (138 mg, 58% yield). HRMS (ESI+): [M+H]+ calculated 3185.1073; found 185.1074. ^{1}H-NMR (400 MHz, $CDCl_3$): δ/ppm= 8.55 (ddd, J= 4.87, 1.88, 0.98 Hz, 2H), 7.55 (td, J= 7.65, 1.87 Hz, 2H), 7.08-7.14 (m, 4H), 3.24 (s, 4H). ^{13}C-NMR (101 MHz, $CDCl_3$): δ/ppm= 161.15, 149.32, 136.32, 123.04, 121.17, 38.14.

Synthesis of 1,2-bis(6-methylpyridin-2-yl)ethane 12a

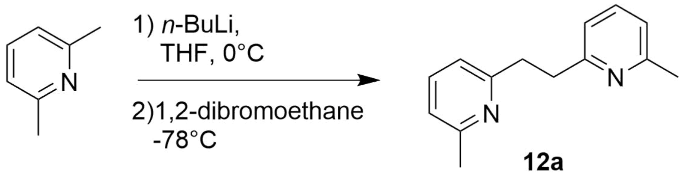

The same procedure as for the synthesis of **11a** was used. The scale was increased by 2.4 and 2-methylpyridine was replaced by 2,6-lutidine. The product was obtained as a yellow crystalline solid (607 mg, 88% yield). HRMS (ESI+): [M+Na]+ calculated 235.1206; found 235.1206. ^{1}H-NMR (400 MHz, $CDCl_3$): δ/ppm= 7.44 (t, J= 7.64 Hz, 2H), 6.94 (dd, J= 12.06, 763 Hz, 4H), 3.17 (s, 4H), 2.54 (s, 6H). ^{13}C-NMR (101 MHz, $CDCl_3$): δ/ppm= 160.64, 157.77, 136.47, 120.60, 119.78, 38.45, 24.58.

Synthesis of 1,2-bis(6-(*tert*-butyl)pyridin-2-yl)ethane 13a

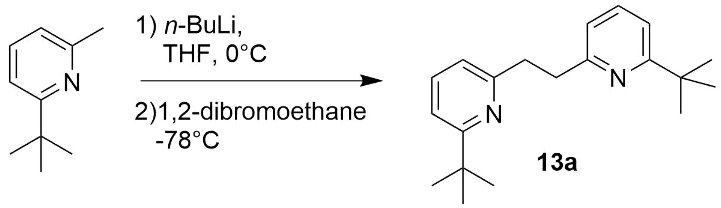

The same procedure as for the synthesis of **11a** was used. The scale was divided by 2.0 and 2-methylpyridine was replaced by 2-(*tert*-butyl)-6-methylpyridine. The product was obtained as a white solid (123 mg, 62% yield). HRMS (ESI+): [M+H]+ calculated 295.2169; found 295.2164. ^{1}H-NMR (400 MHz, $CDCl_3$): δ/ppm= 7.43 (d, J= 7.32 Hz, 2H), 7.08 (dd, J= 7.87, 0.92 Hz, 2H), 6.88 (dd, J= 7.57, 0.98 Hz, 2H), 3.24 (s, 4H), 1.34 (s, 18H). ^{13}C-NMR (101 MHz, $CDCl_3$): δ/ppm= 168.39, 160.11, 135.91, 119.56, 115.69, 37.60, 37.37, 30.20.

Synthesis of 1,3-di(pyridin-2-yl)propane 14a

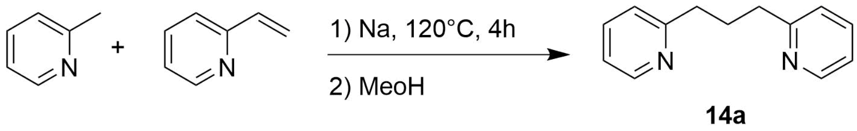

The procedure was adapted from literature.[7] Sodium (40.0 mg, 1.74 mmol, 0.12 equiv) was inserted in an oven-dried two-neck flask under argon atmosphere, followed by 2-methylpyridine (6.63 g, 7.03 mL, 71.2 mmol, 5.0 equiv.). The mixture was stirred for 2 h to allow the dissolution of sodium. The temperature was raised to 120°C and 2-vinylpyridine (1.51 g, 1.55 mL, 14.4 mmol, 1.0 equiv) was added dropwise and the mixture was stirred for 4 h. The mixture was cooled to 0 °C and methanol (25 mL) was added dropwise to quench the remaining sodium. the resulting red solution was stirred over the weekend. The crude mixture was distilled under reduced pressure. The product was found in the fraction distilled at 1 mbar with a 150°C oil bath. The product was obtained as a yellow oil (1.045 g, 37% yield). HRMS (ESI+): [M+Na]+ calculated 221.1049; found 221.1052. ^{1}H-NMR (400 MHz, $CDCl_3$): δ/ppm= 4.14 (ddd, J= 4.90, 1.90, 0.95 Hz, 2H), 7.57 (td, J= 7.67, 1.87 Hz, 2H), 7.15 (d, J= 7.86 Hz, 2H), 7.09 (ddd, J= 7.52, 4.88, 1.18, TODO H), 2.86 (m, 4H), 2.19 (s, 2H). ^{13}C-NMR (101 MHz, $CDCl_3$): δ/ppm= 161.82, 149.25, 136.31, 122.85, 121.02, 37.90, 29.81.

2.3 Synthesis of ^{15}N-tagged species

Synthesis of di(pyridin-2-yl)methane t5a

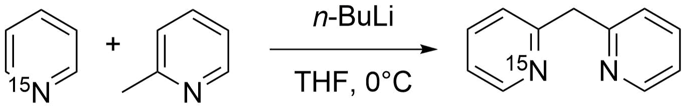

The procedure was adapted from literature.[4] A solution of ^{15}N-pyridine (500 mg, 6.24 mmol, 1.0 equiv.) in THF (8 mL, dry) and a solution of 2-methylpyridine (0.62 mL, 7.05 mmol, 1.1 equiv.) in THF (8 mL, dry) were prepared in oven-dried Schlenk flasks and kept under inert atmosphere. Both

solutions were cooled to 0°C. n-BuLi (4.5 mL, 1.6 M in hexanes, 7.2 mmol, 1.2 equiv.) was added slowly to the 2-methylpyridine solution. After 20 min of stirring, this solution was added dropwise to the pyridine solution via cannula. The mixture was stirred for 24 h at room temperature. The mixture was diluted with cold water (50 mL) and extracted with dichloromethane (2x50 mL). The combined organic phase was dried over $MgSO_4$, filtrated and concentrated under reduced pressure. The resulting brown oil was purified by distillation under reduced pressure. The first fraction was discarded and the second was further purified by silica gel column chromatography using a gradient of hexanes to ethyl acetate as eluent to yield a dark yellow oil (130 mg, 12% yield). HRMS (ESI+): [M+H]+ calculated 172.0887; found 172.0887. ^{1}H-NMR (400 MHz, $CDCl_3$):δ/ppm= 8.55 (qdd, J= 5.77, 1.91, 0.96 Hz, 2H), 7.60 (td, J= 7.64, 1.88 Hz, 2H), 7.26 (d, J= 7.78 Hz, 2H), 7.13 (dd$J$= 7.21, 5.76 Hz, 2H), 4.34(d, J= 2.90 Hz, 2H). ^{13}C-NMR (101 MHz, $CDCl_3$): δ/ppm= 159.61, 159.60, 149.62, 149.60, 136.77, 136.75, 123.76, 123.75, 121.65, 121.63, 47.51, 47.43.

Synthesis of pyridine N-oxide

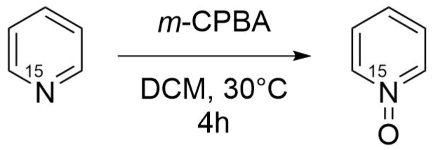

The procedure was adapted from literature.[8] m-chloroperbenzoic acid (6.1 g, 35.3 mmol, 1.4 equiv.), dichloromethane (25 mL) and ^{15}N-pyridine (2.0 g, 25.0 mmol, 1.0 equiv.) were inserted in a round bottom flask and heated to 30°C for 4 h. Sodium sulfite (23 mg) was added. The solution was diluted with dichloromethane and basified with an excess of ammonium bicarbonate. The mixture was filtrated over celite and concentrated under reduced pressure to yield a white crystalline solid (2.17 g, 90% yield) which was used without further purification. HRMS (ESI+): [M+Na]+ calculated 119.0234; found 119.0237. ^{1}H-NMR (400 MHz, $CDCl_3$):δ/ppm= 8.22-8.24 (m, 2H), 7.27-7.31 (m, 3H). ^{13}C-NMR (101 MHz, $CDCl_3$): δ/ppm= 139.64, 139.49, 126.28, 126.27, 126.07, 126.02. ^{15}N-NMR (61 MHz, $CDCl_3$): δ/ppm= 292.53.

Synthesis of 2,6-dibromopyridine

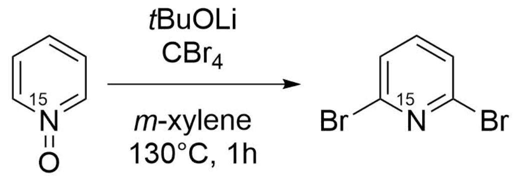

The procedure was adapted from literature.[9] ^{15}N-pyridine oxide (2.13 g, 22 mmol, 1.0 equiv.), CBr_4 (18.40 g, 55 mmol, 2.5 equiv.), t-BuOLi (7.14 g, 89 mmol, 4.0 equiv.) and m-xylene (50 mL) were inserted in a round bottom flask equipped with a condenser and heated to 130°C for 1 h. The mixture was let to cool down to room temperature before purification by silica gel column chromatography. The product was further purified by recrystalization in ethanol to yield a off-white crystalline solid (829 mg, 16% yield). HRMS (ESI+): [M+H]+ calculated 236.8675; found 236.8672. ^{1}H-NMR (400 MHz, $CDCl_3$):δ/ppm= 7.48-7.45 (m, 2H), 7.40 (ddd, J= 8.87, 6.28, 0.68 Hz, 1H). ^{13}C-NMR (101 MHz, $CDCl_3$): δ/ppm= 141.04, 141.02, 140.24, 140.22, 127.21, 127.19. ^{15}N-NMR (61 MHz, $CDCl_3$): δ/ppm= 315.72.

Synthesis of 2-bromo-6-(*tert*-butyl)-pyridine

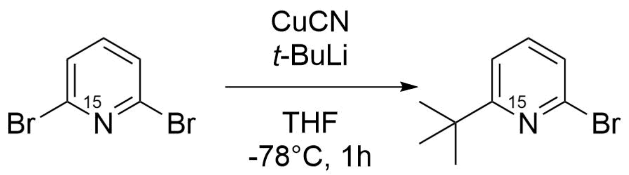

The same procedure as for the natural abundance compound (section 1.1) was used at a reduced scale (620 mg of ^{15}N-2,6-dibromopyridine). Purification by silica gel chromatography was necessary and yielded the product as a yellow liquid (196 mg, 35% yield). 126 mg of the starting material were recovered. HRMS (ESI+): [M+H]+ calculated 215.0196; found 215.0192. ^{1}H-NMR (400 MHz, $CDCl_3$):δ/ppm= 7.44 (ddd, J= 8.10, 7.55, 0.66 Hz, 1H), 7.27 (q, J= 1.17 Hz, 1H), 7.25(dq, J= 1.43. 0.87 Hz, 1H), 1.34 (s, 9H). ^{13}C-NMR (101 MHz, $CDCl_3$): δ/ppm= 171.36, 141.34, 138.62, 138.60, 125.11, 125.08, 117.94, 117.92, 30.13, 30.12. ^{15}N-NMR (61 MHz, $CDCl_3$): δ/ppm= 313.13.

Synthesis of bis(6-(*tert*-butyl)pyridin-2-yl)methane t7a

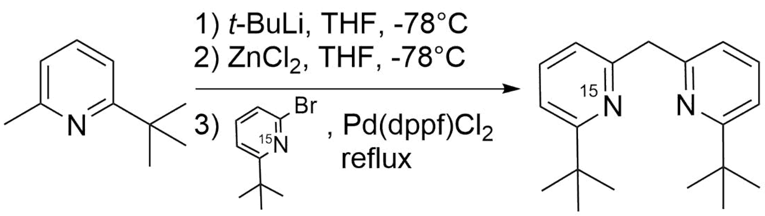

The same procedure as for the natural abundance compound **7a** (section 1.2) was used at a reduced scale (100.6 mg of ^{15}N-2-bromo-6-(*tert*-butyl)-pyridine) to yield the product as a pale yellow liquid (87 mg , 65% yield). HRMS (ESI+): [M+H]+ calculated 284.2139; found 284.2135. ^{1}H-NMR (400 MHz, $CDCl_3$):δ/ppm= 7.50 (t, J= 7.74 Hz, 2H), 7.15 (d, J= 7.87 Hz, 2H), 7.06 (d, J= 7.68 Hz, 2H), 4.31 (d, J= 2.82 Hz, 2H), 1.38 (s, 18H). ^{13}C-NMR (101 MHz, $CDCl_3$): δ/ppm= 168.66, 158.74, 136.34, 136.32, 120.26, 116.19, 48.00, 47.91, 37.51, 30.35. ^{15}N-NMR (61 MHz, $CDCl_3$): δ/ppm= 312.69.

2.4 General procedure for the protonation of bipyridines

The neutral bipyrdine (1 mmol, 1.0 equiv.) was disolved in dichloromethane (15 mL). Water (60 mL), NaBArF (886 mg, 1 mmol, 1.0 equiv.) and HCl (37%, 0.43 mL, 5 mmol, 5.0 equiv.) were added. The reaction was stirred overnight. The mixture was then dilluted with dichloromethane (50 mL). The organic phase was washed with water (2x50 mL), dried over Na_2SO_4, filtrated and concentrated under reduced pressure. The resulting solid was then purified by repeated dissolution and precipitation in dichloromethane and pentane. An execss of NaBArF can be removed by dissolution in a minimum of $CHCl_3$ and filtration. Crystalisation was usually reached by slow cooling in $CHCl_3$, dichloromethane/hexanes or dichloromethane/pentane. This procedure was scaled up/down when necessary.

2.5 Characterisation of BArF salts

Characterisation of 1b

HRMS (ESI+): [M]+ calculated 185.1073; found 185.107. Elemental analysis [CHNF]: Calculated 50.41%C, 2.40%H, 1.03%B, 43.49%F, 2.67%N; found 50.41%C, 2.22%H, 2.53%N, 40.80%F. ^{1}H-NMR (400 MHz, $CDCl_3$): δ/ppm= 8.31 (dd, J= 5.54, 0.73 Hz, 2H), 8.01 (s, broad, 2H), 7.69 (s, broad, 8H), 7.48 (s, broad, 4H), 7.46 (dd, J= 5.55, 0.83 Hz, 2H), 2.54 (s, 6H). ^{13}C-NMR (101 MHz, $CDCl_3$): δ/ppm= 162.55, 162.06, 161.56, 161.07, 157.82, 144.86, 144.56, 134.86, 129.28, 128.94, 128.71, 128.66, 125.95, 123.24, 123.06, 120.53, 117.64, 22.10.

Characterisation of 2b

HRMS (ESI+): [M]+ calculated 185.1073; found 185.1073. Elemental analysis [CHNF]: Calculated 50.41%C, 2.40%H, 1.03%B, 43.49%F, 2.67%N; found 50.19%C, 2.18%H, 2.62%N, 41.12%. ^{1}H-NMR (400 MHz, $CDCl_3$): δ/ppm= 7.99 (t,J= 7.91, 2H), 7.84 (d, J= 7.37, 2H), 7.69 (s, broad, 8H), 7.48 (m, broad, 6H), 2.72 (s, 6H). ^{13}C-NMR (101 MHz, $CDCl_3$, remaining peaks not observed): δ/ppm= 157.10, 144.51, 143.77, 134.86, 128.20, 119.70, 117.65, 22.34.

Characterisation of 3b

HRMS (ESI+): [M]+ calculated 269.2012; found 269.2009. Elemental analysis [CHNF]: Calculated 53.02%C, 3.29%H, 0.95%B, 40.26%F, 2.47%N; found 52.77%C, 3.01%H, 2.43%N, 36.60%F. ^{1}H-NMR (400 MHz, $CDCl_3$): δ/ppm= 8.33 (dd, J= 5.78,0.69 Hz, 2H), 8.18 (dd, J= 1.82, 0.71, 2H), 7.70 (dd, J= 5.82, 1.78, 2H), 7.69 (s, broad, 8H), 7.48 (s, broad, 4H), 1.42 (s, 18H). ^{13}C-NMR (101 MHz, $CDCl_3$, remaining peaks not observed): δ/ppm= 170.51, 145.31, 144.79, 134.87, 125.29, 119.20, 117.67, 36.66, 30.19.

Characterisation of 4b

HRMS (ESI+): [M]+ calculated 209.1073; found 209.1074. Elemental analysis [CHN]: Calculated 51.52%C, 2.35%H, 1.01%B, 42.51%F, 2.61%N; found 51.79%C, 2.49%H, 2.75%N. ^{1}H-NMR (400 MHz, $CDCl_3$): δ/ppm= 8.84 (dd, J= 8.54, 1.37, 2H), 8.78 (dd, J= 4.92, 1.38, 2H), 7.92 (dd, J= 8.54, 4.95, 2H), 7.70 (s, broad, 8H), 7.44 (s, broad, 4H), 2.78 (s, 6H). ^{13}C-NMR (101 MHz, $CDCl_3$, remaining peaks not observed): δ/ppm= 144.65, 138.94, 134.87, 130.68, 125.92, 125.86, 123.21, 117.61, 15.46.

Characterisation of 5b

HRMS (ESI+): [M]+ calculated 171.0917; found 171.0914 Elemental analysis [CHN]: Calculated 49.93%C, 2.24%H, 1.05%B, 44.08%F, 2.71%N; found 49.83%C, 2.33%H, 2.75%N. ^{1}H-NMR (400 MHz, $CDCl_3$): δ/ppm= 8.40 (dt, J= 5.47, 1.34, 2H), 8.00 (td, J= 7.88, 1.70, 2H), 7.69 (s, broad, 8H), 7.51 (d, J= 6.80, 2H), 7.48 (s, broad, 4H), 7.44 (d, J= 8.03, 2H), 4.35 (s, 2H). ^{13}C-NMR (101 MHz, $CDCl_3$): δ/ppm= 162.59, 162.09, 161.60, 161.11, 153.91, 143.35, 143.01, 134.85, 129.31, 129.00, 128.65, 126.38, 125.94, 124.98, 123.23, 120.52, 117.67, 34.28.

Characterisation of 6b

HRMS (ESI+): [M]+ calculated 199.123; found 199.123. Elemental analysis [CHN]: Calculated 50.87%C, 2.56%H, 1.02%B, 42.91%F, 2.64%N; found 50.75%C, 2.12%H, 3.04%N. ^{1}H-NMR (400 MHz, $CDCl_3$): δ/ppm= 7.86 (t, J= 7.90, 2H), 7.70 (s, broad, 8H), 7.50 (s, broad, 4H), 7.35 (d, J= 7.90, 2H), 7.25 (d, J= 7.87, 2H), 4.26 (s, 2H), 2.71 (s, 6H). ^{13}C-NMR (101 MHz, $CDCl_3$): δ/ppm= 162.41, 161.91, 161.42, 160.92, 154.36, 152.98, 142.81, 134.72, 129.13, 128.79, 128.51, 125.80, 124.66, 123.12, 120.38, 117.47, 33.50, 21.73.

Characterisation of 7b

HRMS (ESI+): [M]+ calculated 283.2169; found 283.2173. Elemental analysis [CHN]: Calculated 53.42%C, 3.43%H, 0.94%B, 39.76%F, 2.44%N; found 53.43%C, 3.47%H, 2.49%N. ^{1}H-NMR (400 MHz, $CDCl_3$): δ/ppm= 7.80 (t, J= 7.99, 2H), 7.66 (s, broad, 8H), 7.59 (d, J= 8.17, 2H), 7.49 (s, broad, 4H), 7.31 (d, J= 7.78, 2H), 5.31 (s, broad, 2H), 1.60 (s, 18H). ^{13}C-NMR (101 MHz, $CDCl_3$):δ/ppm= 167.85, 162.55, 162.05, 161.56, 161.06, 143.49, 134.87, 129.26, 128.97, 128.65, 125.94, 123.23, 120.52, 117.68, 37.43, 29.86, 27.07.

Characterisation of 8b

HRMS (ESI+): [M]+ calculated 173.0709; found 173.0706. Elemental analysis [CHN]: Calculated 48.67%C, 2.04%H, 1.04%B, 43.99%F, 2.70%N, 1.54%O; found 48.75%C, 2.13%H, 2.93%N. ^{1}H-NMR (400 MHz, $CDCl_3$): δ/ppm= 8.14 (ddd, J= 8.65, 7.45, 1.91, 2H), 8.02 (ddd, J= 5.68, 1.94, 0.74, 2H) 7.66 (s, broad, 8H), 7.49 (s, broad, 4H), 7.42 (ddd, J= 7.49, 5.65, 1.02, 2H), 7.28 (d, J= 8.63, 2H). ^{13}C-NMR (101 MHz, $CDCl_3$, remaining peaks not observed): δ/ppm= 160.01, 146.29, 140.27, 125.94, 123.23, 122.77, 115.80.

Characterisation of 9b

HRMS (ESI+): [M]+ calculated 201.1022; found 201.1024. Elemental analysis [CHN]: Calculated 49.65%C, 2.27%H, 1.02%B, 42.83%F, 2.63%N, 1.50%O; found 48.51%C, 1.80%H, 2.13%N. ^{1}H-NMR (400 MHz, $CDCl_3$): δ/ppm= 7.97 (dd, J= 8.56, 7.62, 2H) 7.68 (s, broad, 8H), 7.48 (s, broad, 4H), 7.22 (d, J= 7.62, 2H), 7.05 (d, J= 8.52, 2H), 2.62 (s, 6H). ^{13}C-NMR (101 MHz, $CDCl_3$): δ/ppm= 162.55, 162.06, 161.56, 161.07, 159.98, 152.11, 146.09, 134.86, 129.26, 128.89, 128.66, 125.96, 123.25, 122.14, 117.61, 112.32, 76.84, 21.76.

Characterisation of 10b

HRMS (ESI+): [M]+ calculated 285.1961; found 285.1966. Elemental analysis [CHNF]: Calculated 52.28%C, 3.25%H, 0.94%B, 39.70%F, 2.44%N, 1.39%O; found 52.43%C, 2.97%H, 2.51%N, 37.10%F. ^{1}H-NMR (400 MHz, $CDCl_3$): δ/ppm= 7.98 (dd, J= 8.43, 7.83, 2H), 7.68 (s, broad, 8H), 7.49 (s,

broad, 4H), 7.44 (dd, J= 7.83, 0.89, 2H), 7.00 (dd, J= 8.43, 0.88, 2H), 1.48 (s, 18H). ^{13}C-NMR (101 MHz, $CDCl_3$): δ/ppm= 164.69, 158.80, 146.22, 134.87, 125.97, 123.26, 118.49, 117.67, 112.97, 37.24, 29.88.

Characterisation of 11b

HRMS (ESI+): [M]+ calculated 185.1073; found 185.1074 Elemental analysis [CHN]: Calculated 50.41%C, 2.40%H, 1.03%B, 43.49%F, 2.67%N; found 50.15%C, 2.49%H, 2.73%N. ^{1}H-NMR (400 MHz, $CDCl_3$): δ/ppm= 8.42 (dt, J= 5.29, 1.43, 2H), 8.00 (td, J= 7.81, 1.68, 2H), 7.69 (s, broad, 8H), 7.49 (s, broad, 4H), 7.46 (d, J= 5.47, 2H), 3.30 (s, 4H). ^{13}C-NMR (101 MHz, $CDCl_3$): 162.57, 162.08, 161.58, 161.09, 157.23, 143.50, 143.47, 134.87, 129.25, 128.99, 128.66, 126.52, 125.95, 124.44, 123.24, 117.65, 32.80.

Characterisation of 12b

HRMS (ESI+): [M]+ calculated 213.1386; found 213.1388 Elemental analysis [CHNF]: Calculated 51.32%C, 2.72%H, 1.00%B, 42.36%F, 2.60%N; found 51.30%C, 2.40%H, 2.58%N, 39.60%F. ^{1}H-NMR (400 MHz, $CDCl_3$): δ/ppm= 7.84 (t, J= 7.84, 2H), 7.68 (s, broad, 8H), 7.49 (s, broad, 4H), 7.29 (d, J= 7.91, 2H), 7.24 (d, J= 9.00, 2H), 3.23 (s, 4H), 2.73 (s, 6H). ^{13}C-NMR (101 MHz, $CDCl_3$, remaining peaks not observed): δ/ppm= 157.20, 155.16, 143.16, 134.90, 124.64, 123.46, 117.64, 33.01, 22.13.

Characterisation of 13b

HRMS (ESI+): [M]+ calculated 297.2325; found 297.2329. Elemental analysis [CHN]: Calculated 53.81%C, 3.56%H, 0.93%B, 39.28%F, 2.41%N; found 53.42%C, 3.40%H, 2.59%N. ^{1}H-NMR (400 MHz, $CDCl_3$): δ/ppm= 10.42 (s, broad, 1H), 7.82 (t, J= 7.92, 2H), 7.69 (s, broad, 8H), 7.51 (s, broad, 4H), 7.45 (dd, J= 8.10, 1.03, 2H), 7.19 (dd, J= 7.73, 1.04, 2H), 3.43 (s, 4H), 1.23 (s, 18H). ^{13}C-NMR (101 MHz, $CDCl_3$): δ/ppm= 167.37, 162.57, 162.07, 161.58, 161.08, 156.24, 143.13, 134.89, 129.24, 128.90, 128.69, 125.98, 123.22, 120.90, 117.62, 37.27, 32.71, 29.48.

Characterisation of 14b

HRMS (ESI+): [M]+ calculated 199.123; found 199.123. Elemental analysis [CHNF]: Calculated 50.87%C, 2.56%H, 1.02%B, 42.91%F, 2.64%N; found 50.74%C, 2.26%H, 2.47%N, 40.60%F. ^{1}H-NMR (400 MHz, $CDCl_3$): δ/ppm= 8.41 (dd, J= 6.08, 1.67, 2H), 8.05 (td, J= 7.84, 1.72, 2H), 7.69 (s, broad, 8H), 7.49 (s, broad, 4H), 7.48 (d, J= 8.16, 4H), 2.80 (s, 4H), 2.12 (s, 2H). ^{13}C-NMR (101 MHz, $CDCl_3$): δ/ppm=156.83, 143.79, 142.97, 134.90, 127.20, 124.30, 117.71, 30.61, 28.63.

Characterisation of t5b

HRMS (ESI+): [M]+ calculated 172.0887; found 172.0886. Elemental analysis [CHN]: Calculated 49.93%C, 2.24%H, 1.05%B, 44.08%F, 2.71%N; found 49.64%C, 2.23%H, 2.67%N. ^{1}H-NMR (400 MHz, $CDCl_3$): δ/ppm= 10.62 (s, broad, 1H), 8.40 (m, 2H), 8.01 (td, J= 7.89, 1.54 Hz, 2H), 7.69 (s, broad, 10H instead of 8H), 7.52 (d, J= 5.70 Hz, 2H), 7.48 (s, broad, 5H instead of 4H), 7.44 (dd, J= 8.02, 0.99 Hz, 2H), 4.35 (d, J= 2.54 Hz, 2H) . ^{13}C-NMR (51 MHz, $CDCl_3$): 162.59, 162.09, 161.60, 161.10, 153.85, 153.81, 143.31, 143.26, 143.11, 143.07, 134.85, 129.59, 129.30, 129.02, 128.65, 126.44, 126.41, 125.94, 125.04, 125.02, 123.23, 120.52, 117.68, 34.03, 34.01. ^{15}N-NMR (51 MHz, CD_2Cl_2): δ/ppm= 244.40.

Characterisation of t7b

HRMS (ESI+): [M]+ calculated 284.2139; found 284.2134. Elemental analysis [CHN]: Calculated 53.38%C, 3.43%H, 0.94%B, 39.73%F, 2.53%N; found 53.32%C, 3.48%H, 2.50%N. ^{1}H-NMR (400 MHz, $CDCl_3$): δ/ppm= 7.50 (t, J= 7.74 Hz, 2H), 7.15 (s, J= 7.87 Hz, 2H), 7.06 (d, J= 7.68 Hz, 2H), 4.31 (d, J= 2.82 Hz, 2H), 1.38 (s, 18H). ^{13}C-NMR (51 MHz, $CDCl_3$): 168.66, 158.74, 136.34, 136.32, 120.26, 116.19, 48.00, 47.91, 37.51, 30.35 ^{15}NN-NMR (61 MHz, CD_2Cl_2): δ/ppm= 242.59.

NMR spectra

3.1 NMR spectra of precursors

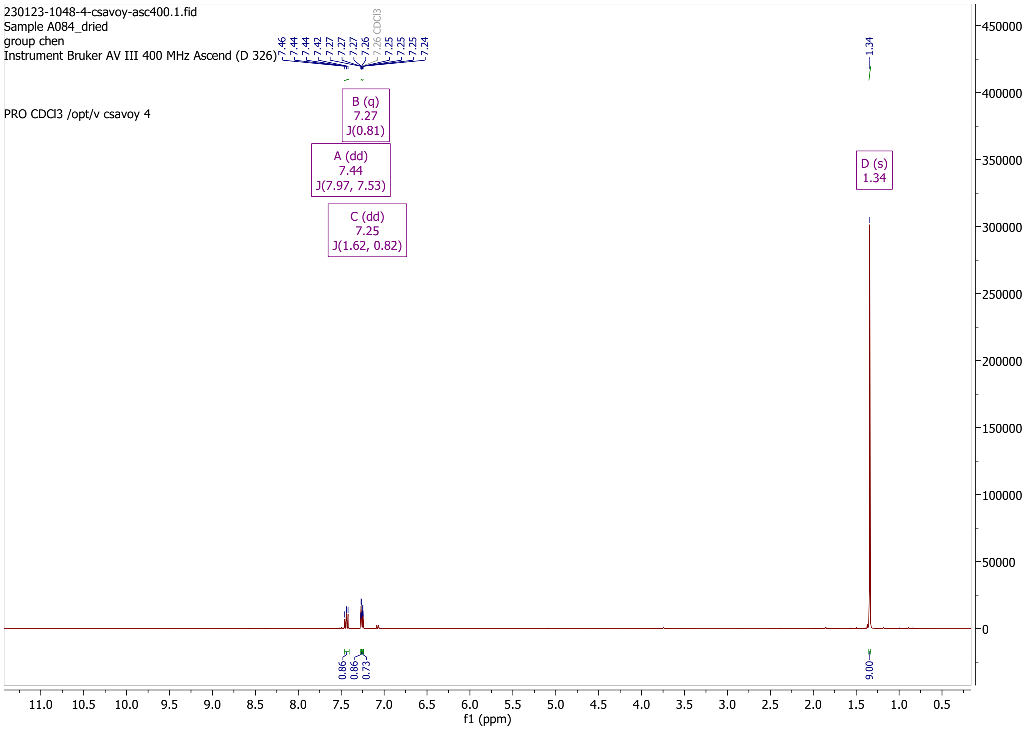

^{1}H-NMR of 2-bromo-6-(*tert*-butyl)pyridine in $CDCl_3$

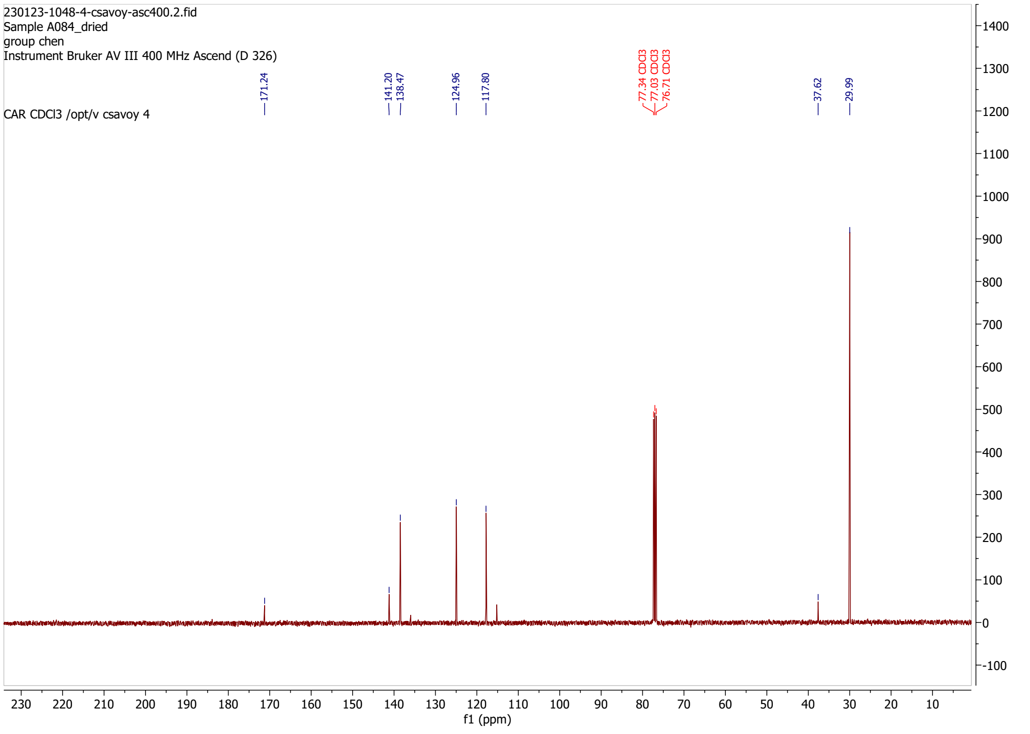

^{13}C-NMR of 2-bromo-6-(*tert*-butyl)pyridine in $CDCl_3$

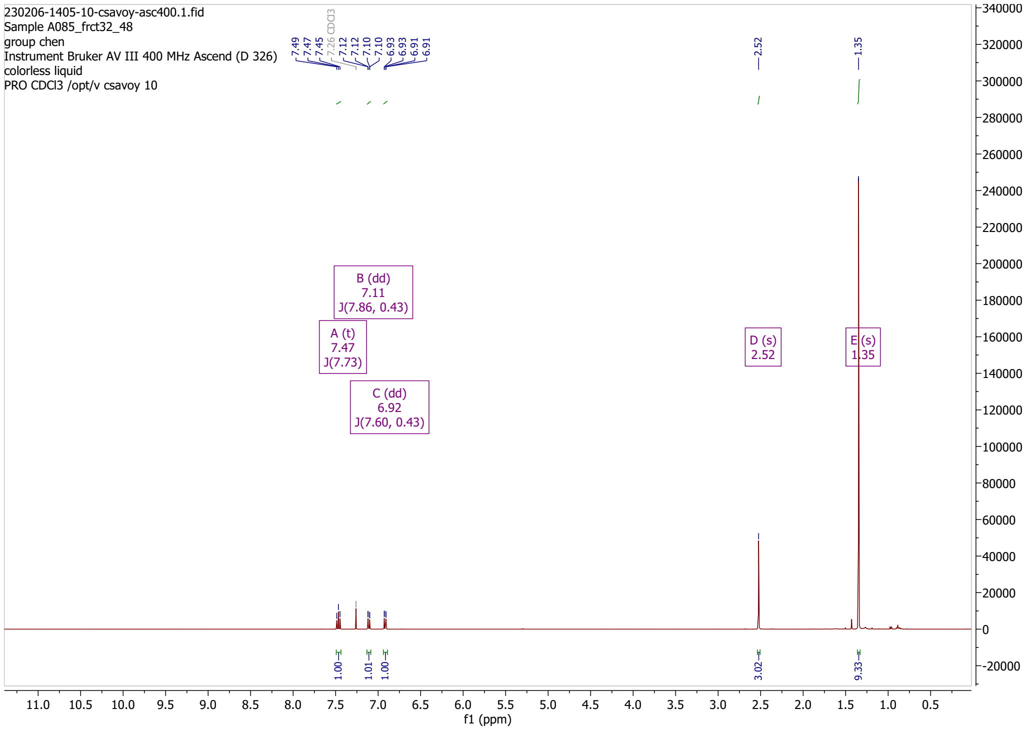

^{1}H-NMR of 2-(*tert*-butyl)-6-methylpyridine in $CDCl_3$

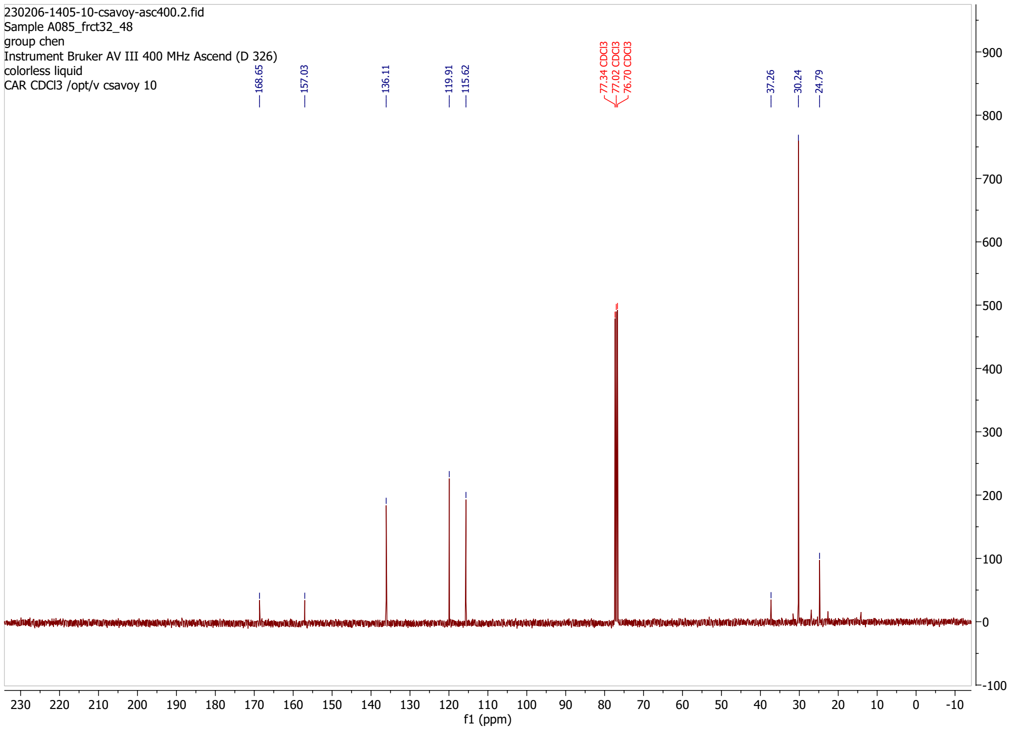

^{13}C-NMR of 2-(*tert*-butyl)-6-methylpyridine in $CDCl_3$

3.2 NMR spectra of neutral bipyridines

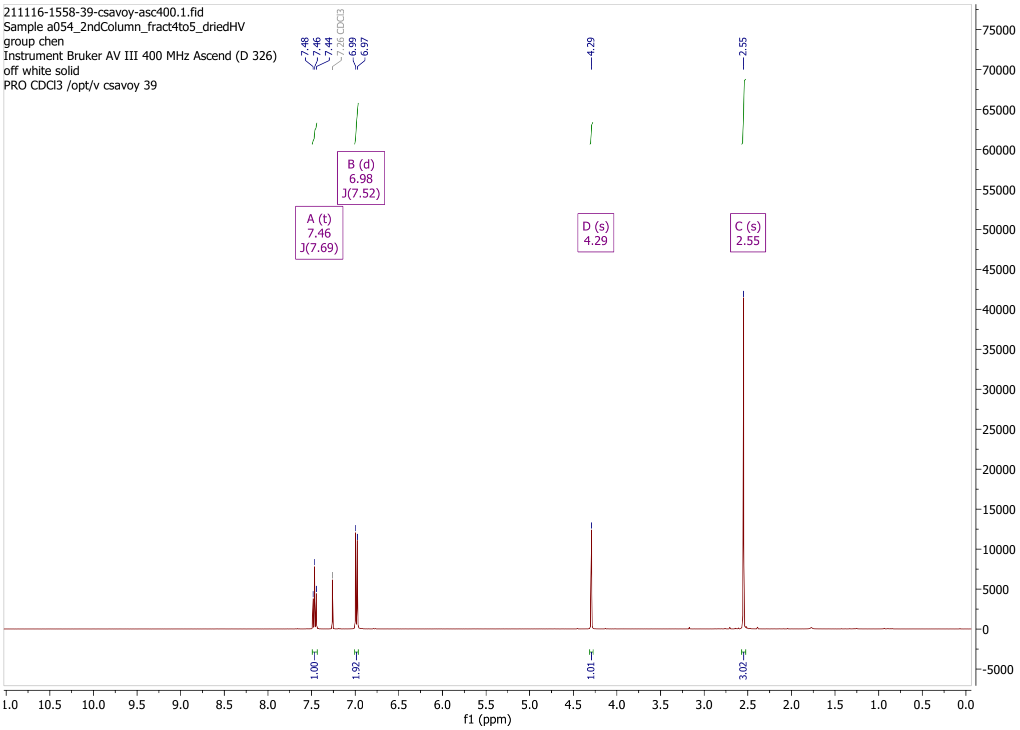

^{1}H-NMR of **6a** in $CDCl_3$

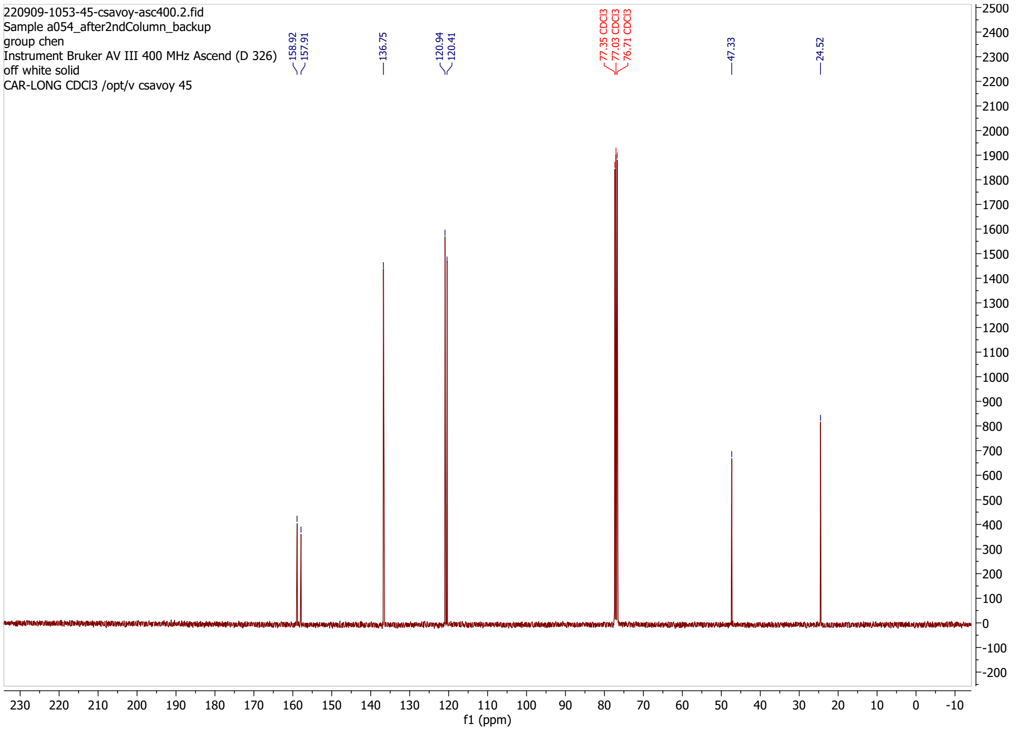

^{13}C-NMR of **6a** in $CDCl_3$

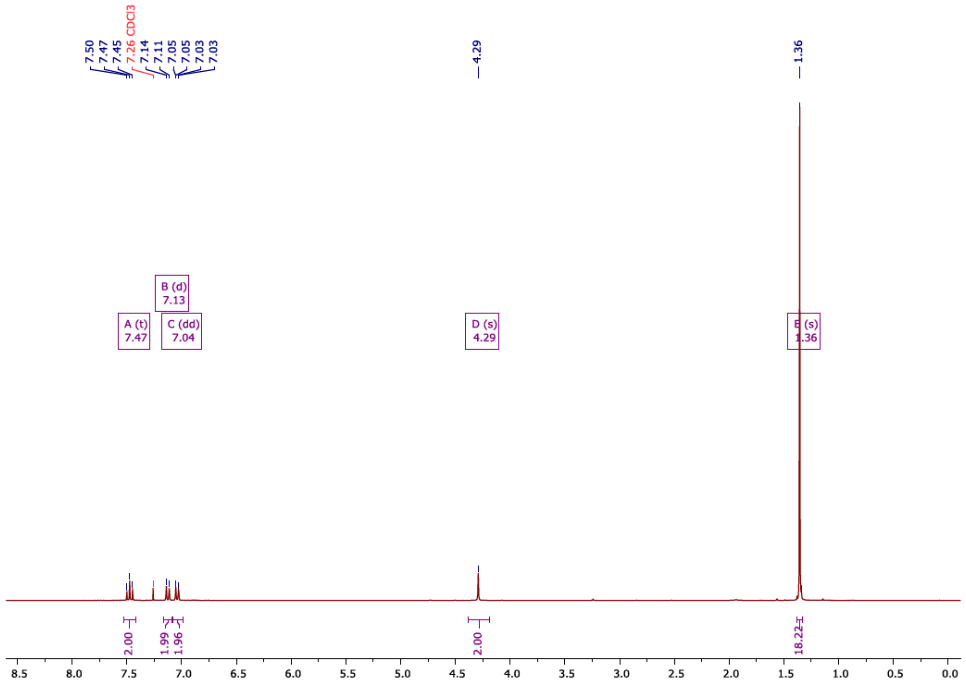

^{1}H-NMR of **7a** in $CDCl_3$

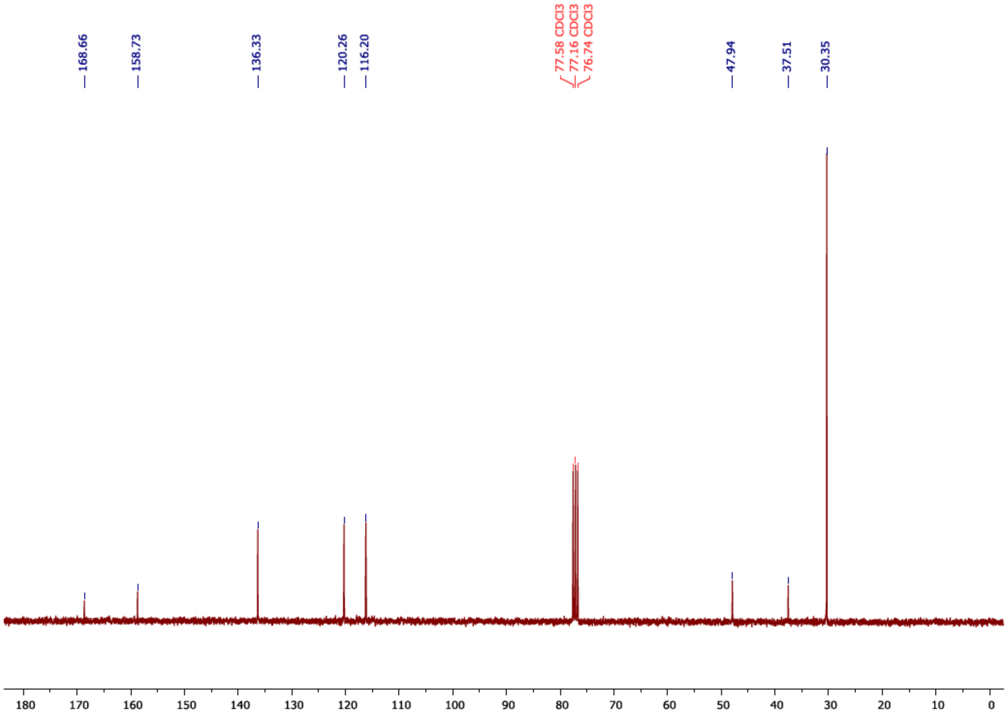

^{13}C-NMR of **7a** in $CDCl_3$

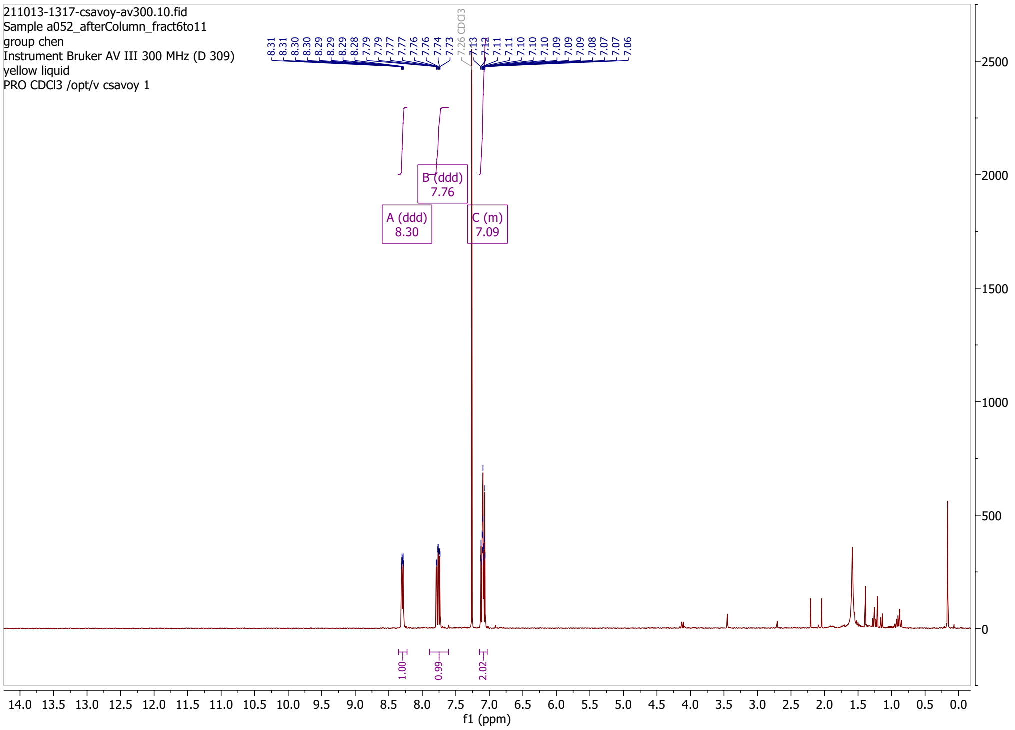

^{1}H-NMR of **8a** in $CDCl_3$

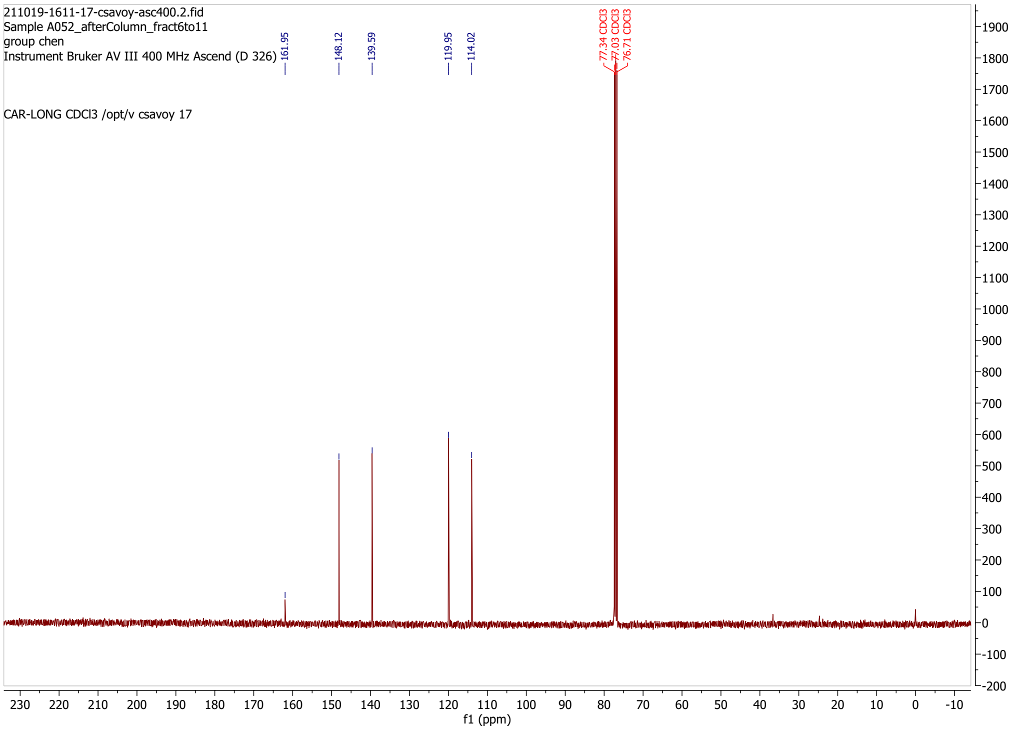

^{13}C-NMR of **8a** in $CDCl_3$

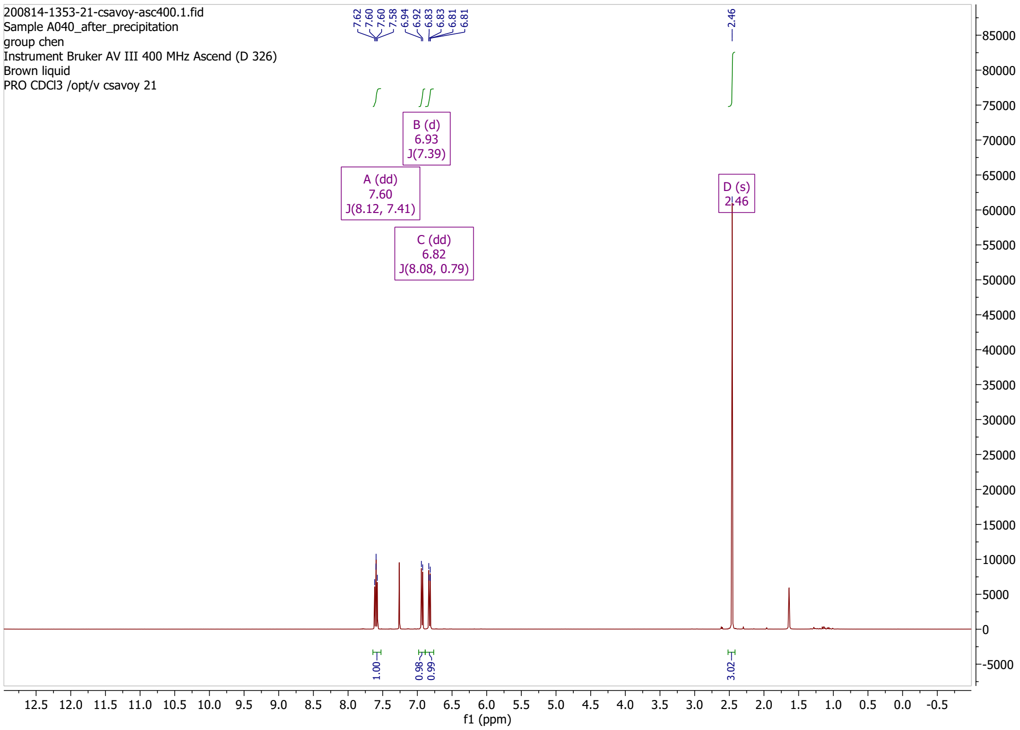

^{1}H-NMR of **9a** in $CDCl_3$

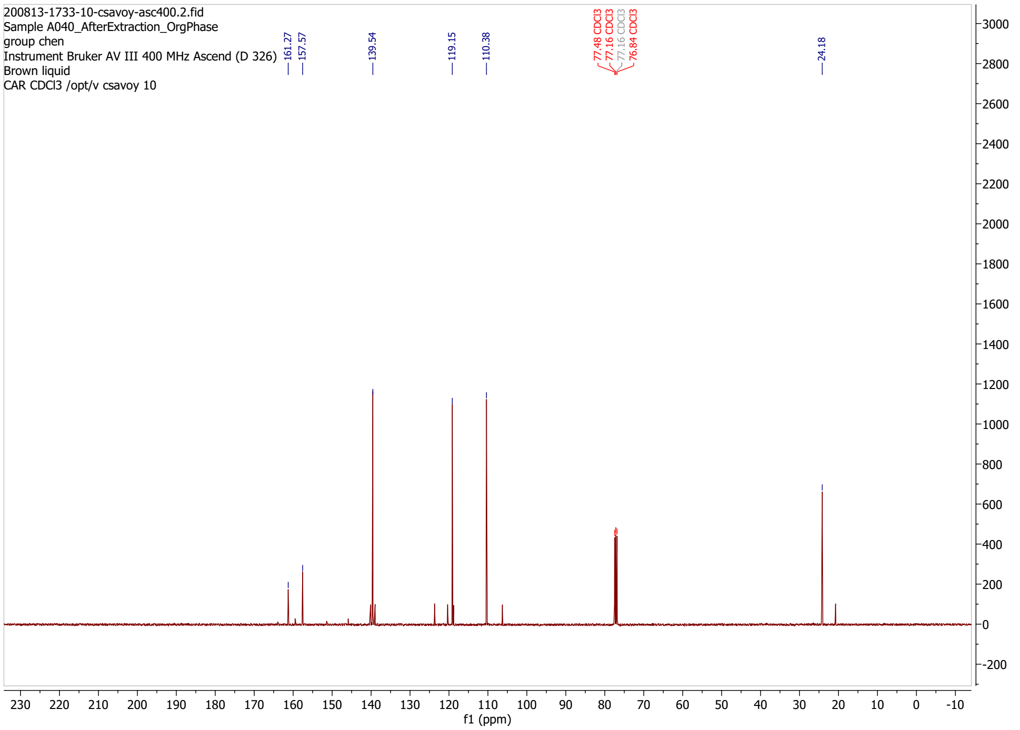

^{13}C-NMR of **9a** in $CDCl_3$

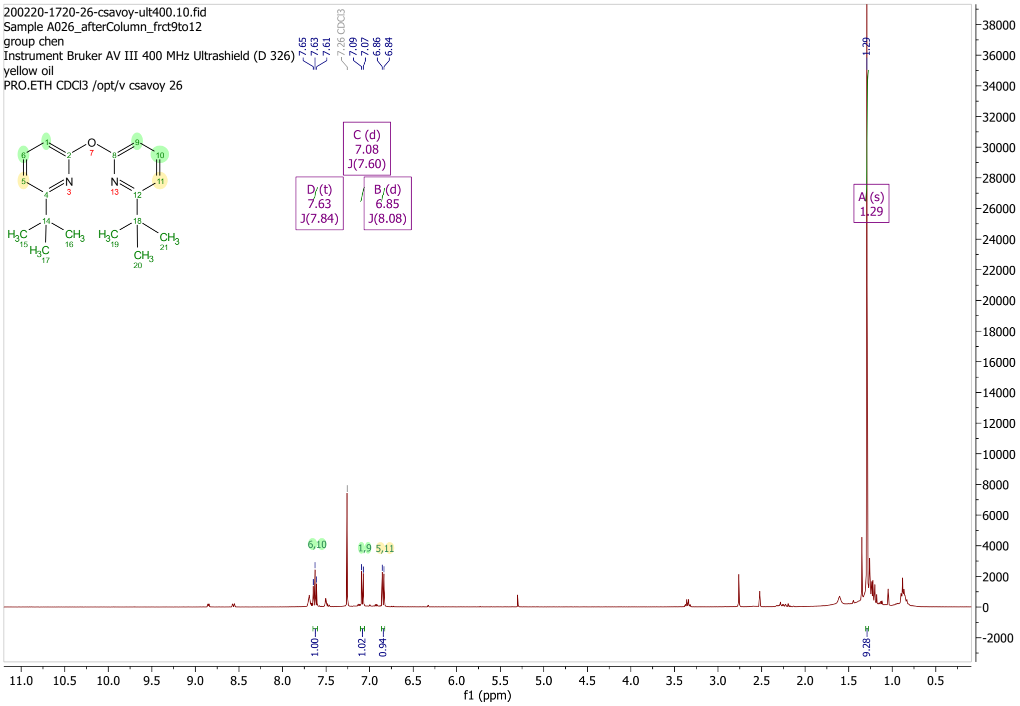

^{1}H-NMR of **10a** in $CDCl_3$

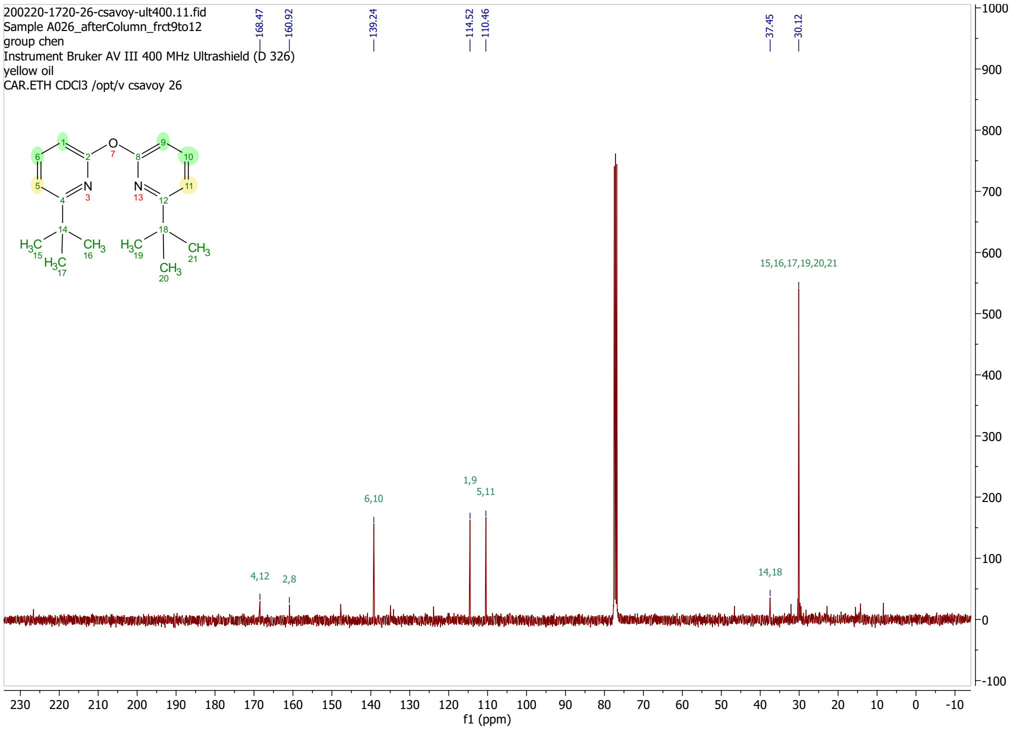

^{13}C-NMR of **10a** in $CDCl_3$

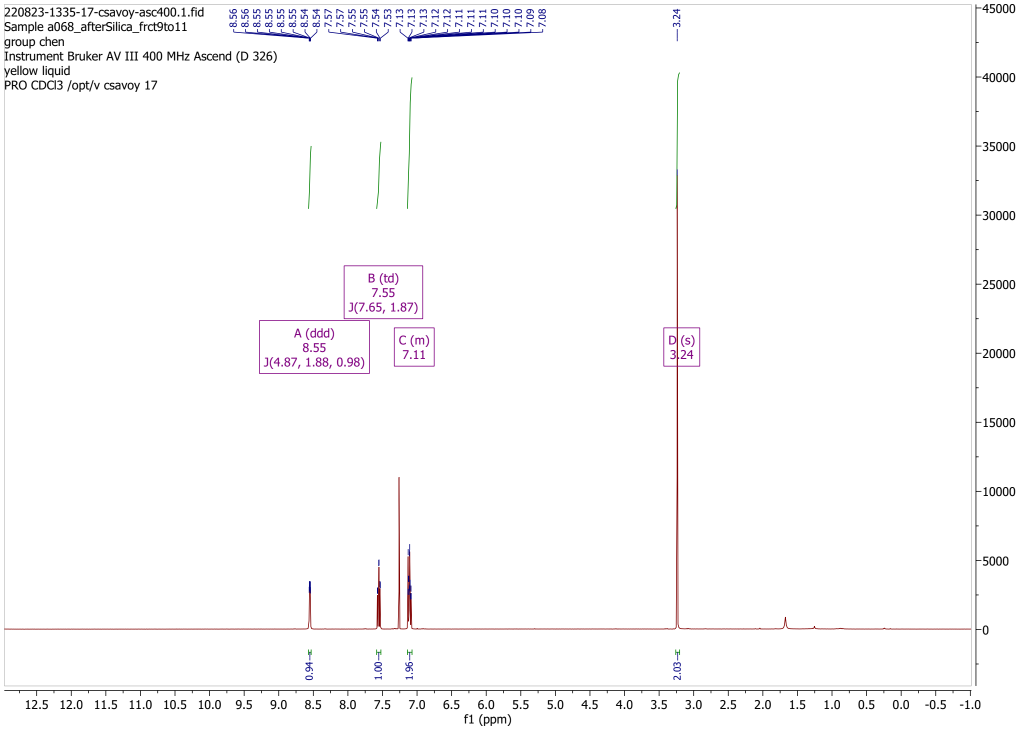

^{1}H-NMR of **11a** in $CDCl_3$

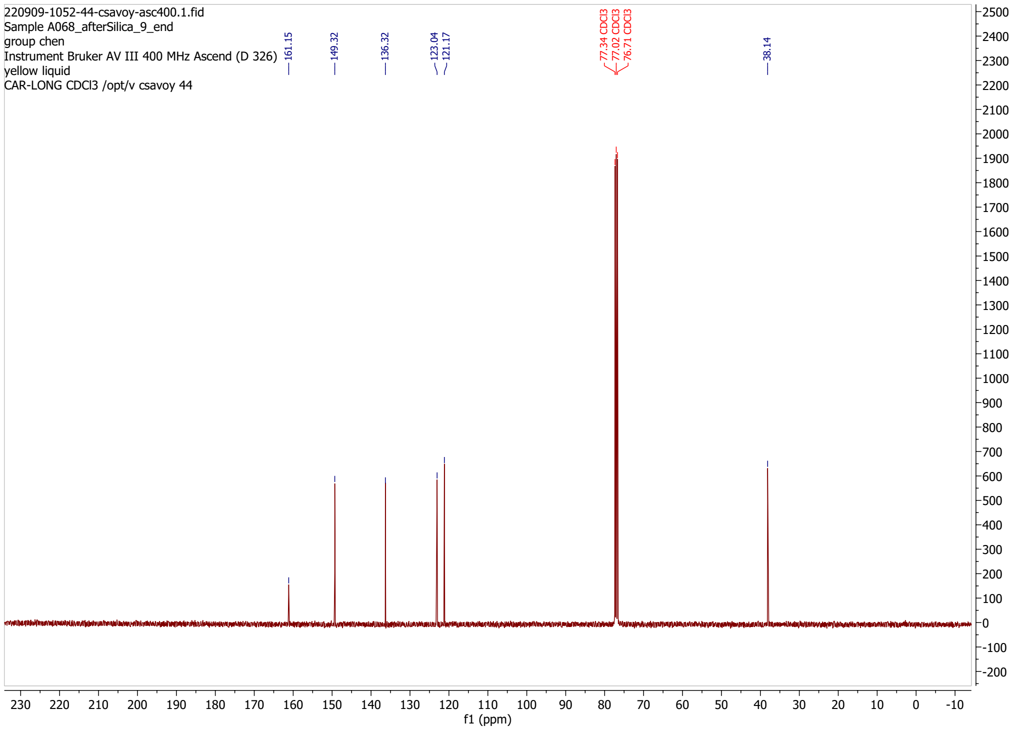

^{13}C-NMR of **11a** in $CDCl_3$

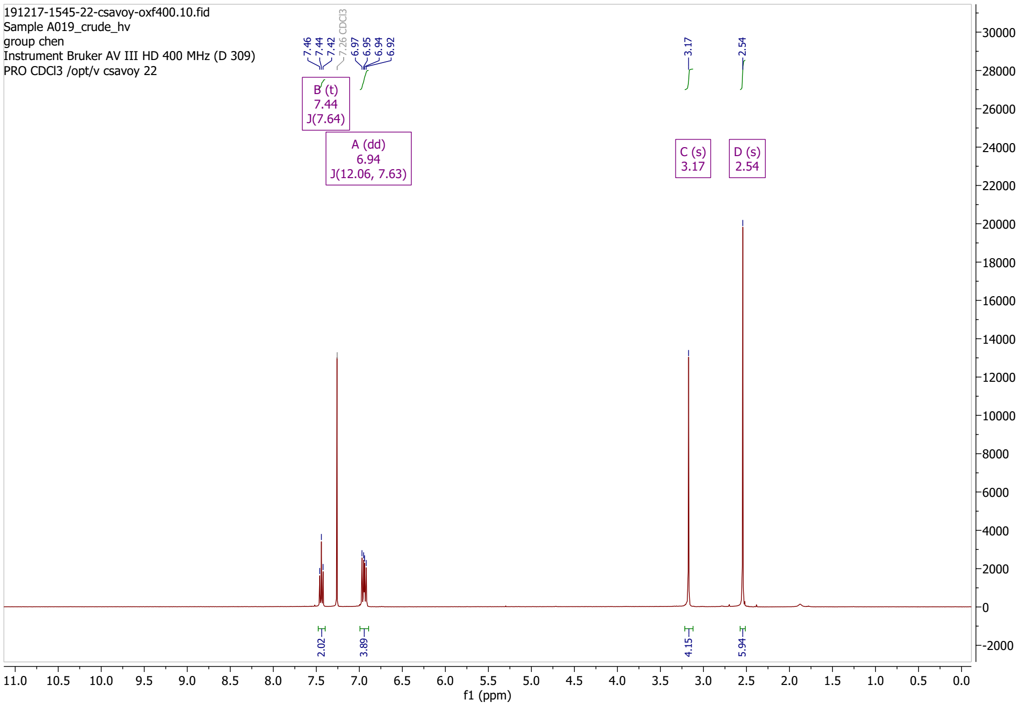

^{1}H-NMR of **12a** in $CDCl_3$

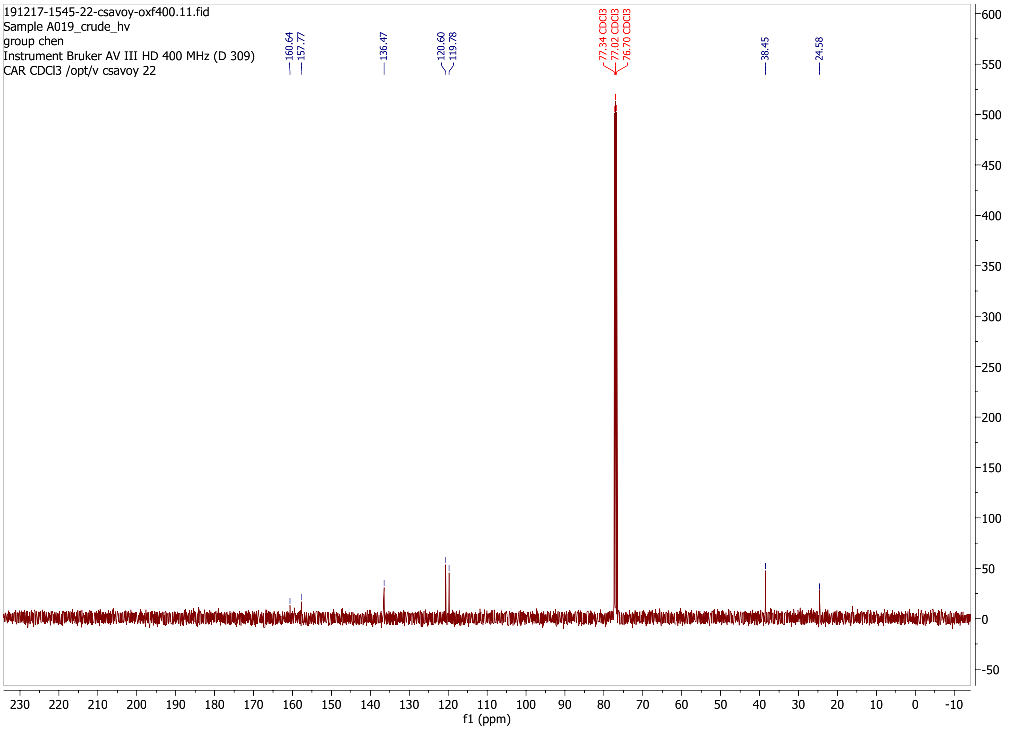

^{13}C-NMR of **12a** in $CDCl_3$

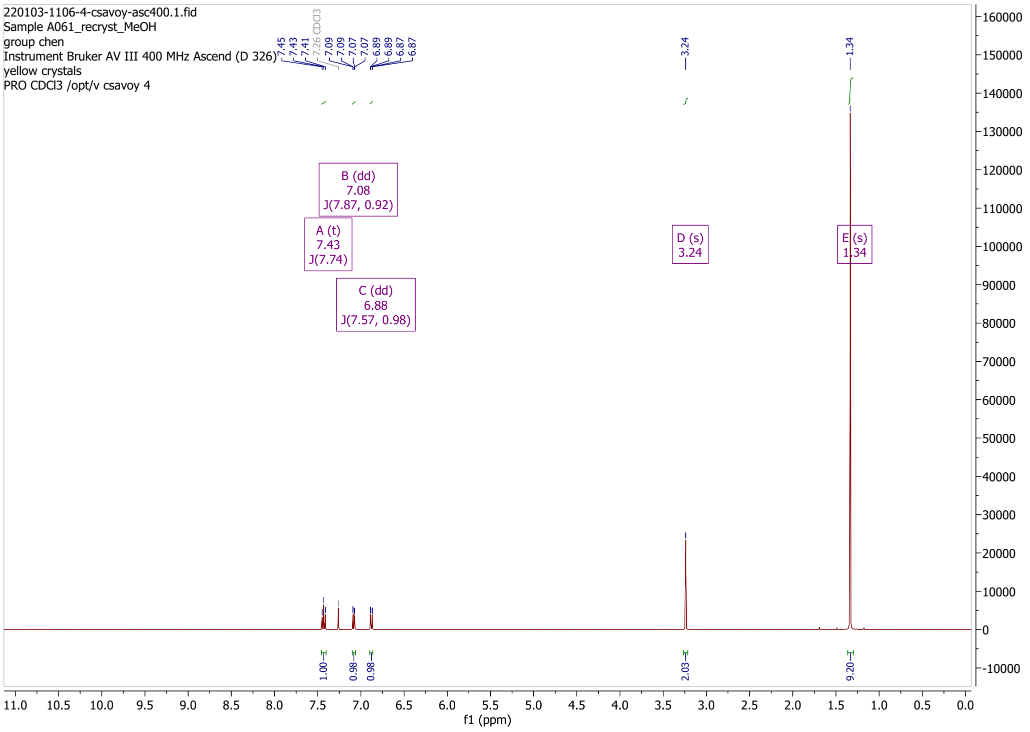

^{1}H-NMR of **13a** in $CDCl_3$

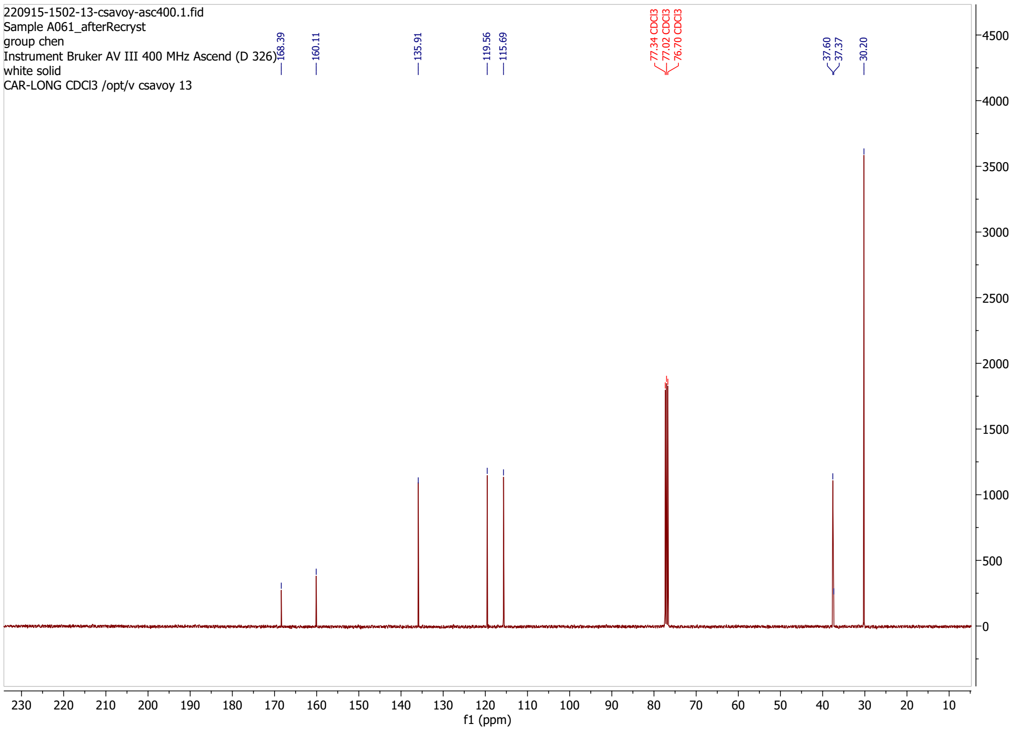

^{13}C-NMR of **13a** in $CDCl_3$

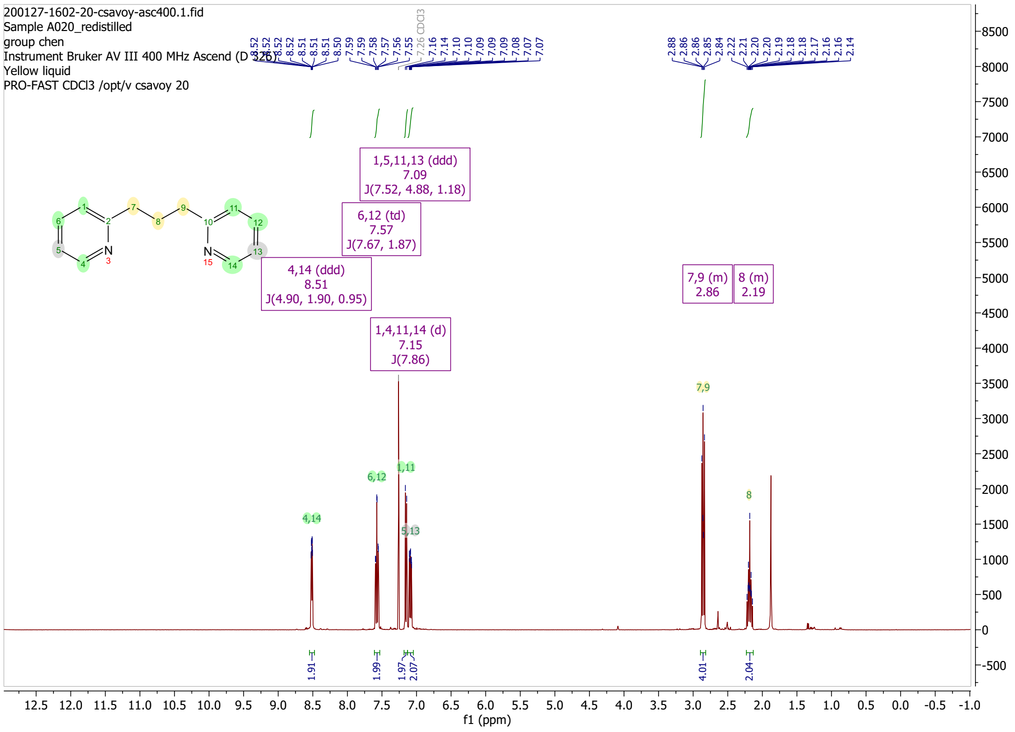

^{1}H-NMR of **14a** in $CDCl_3$

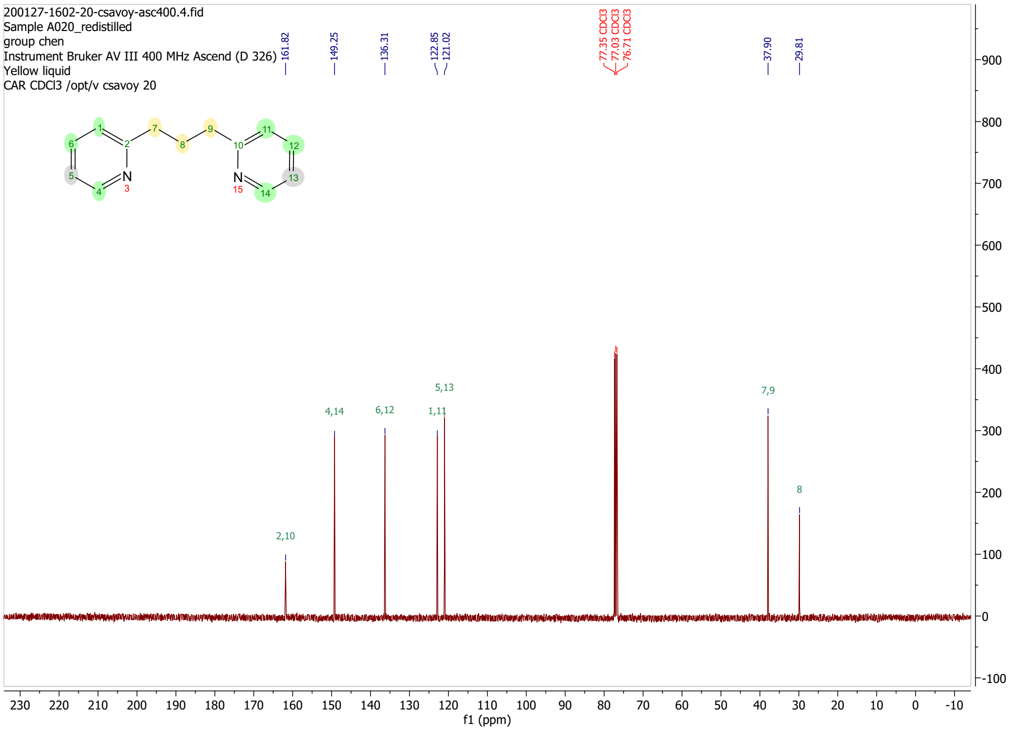

^{13}C-NMR of **14a** in $CDCl_3$

3.3 NMR spectra of protonated bipyridines

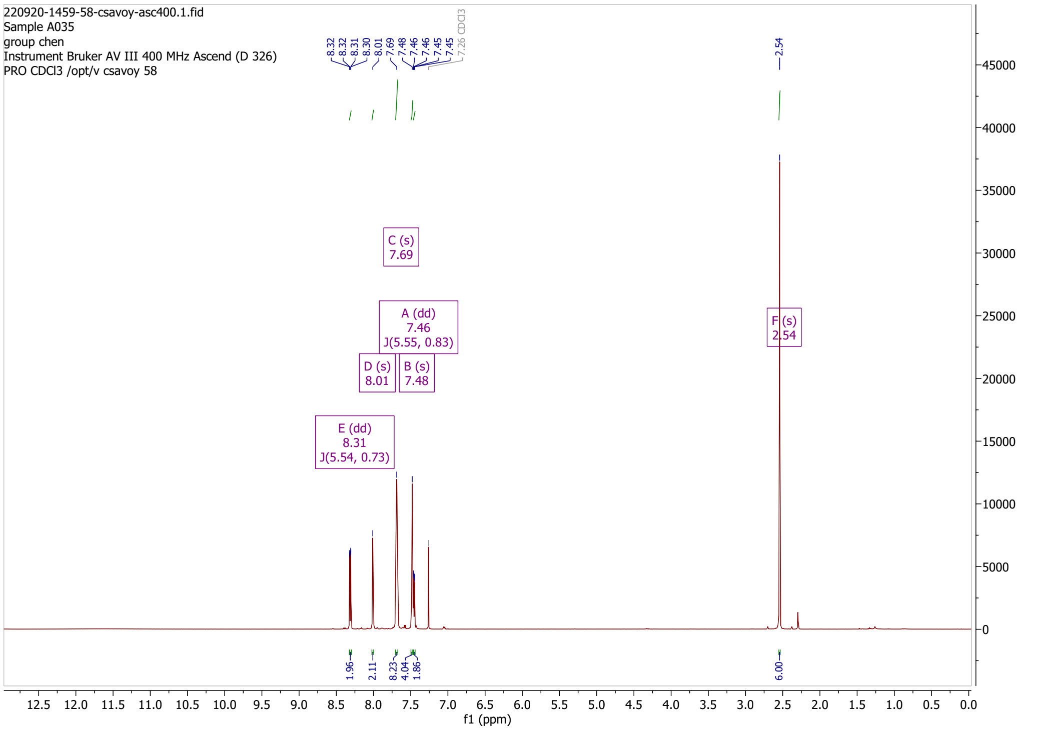

^{1}H-NMR of **1b** in $CDCl_3$

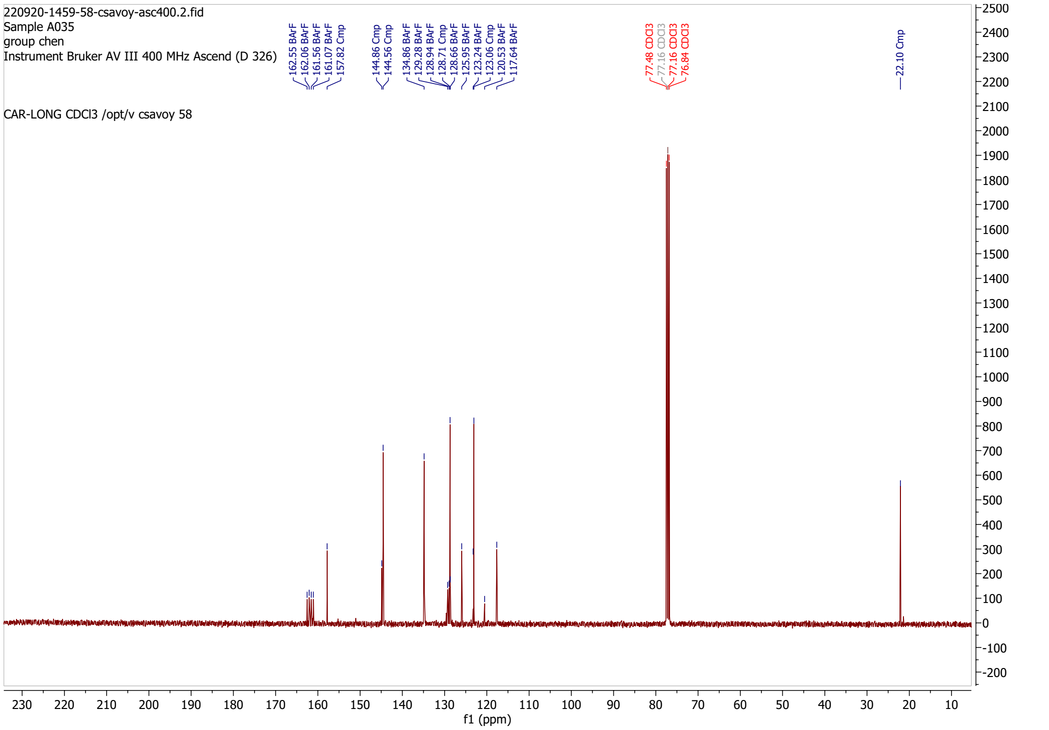

^{13}C-NMR of **1b** in $CDCl_3$

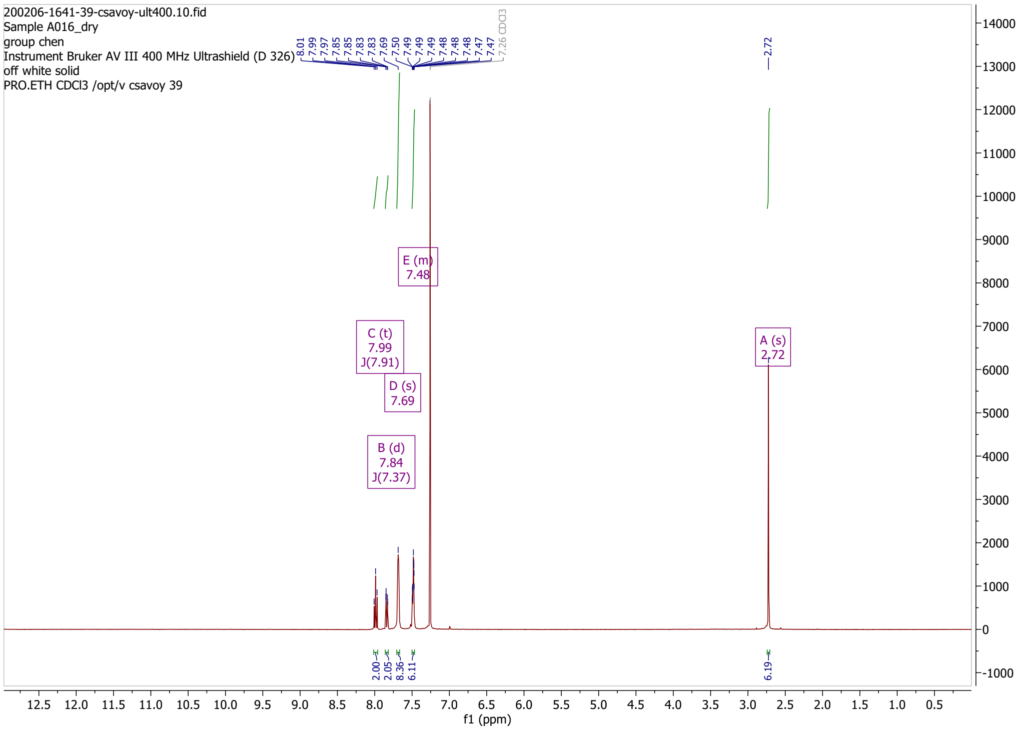

^{1}H-NMR of **2b** in $CDCl_3$

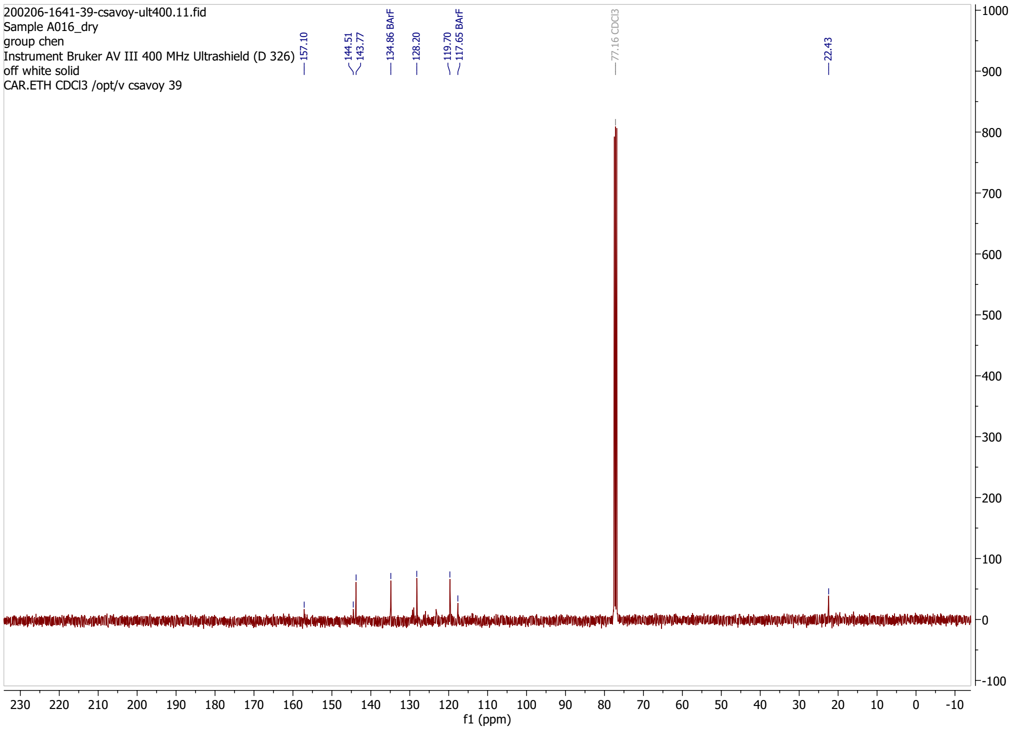

^{13}C-NMR of **2b** in $CDCl_3$

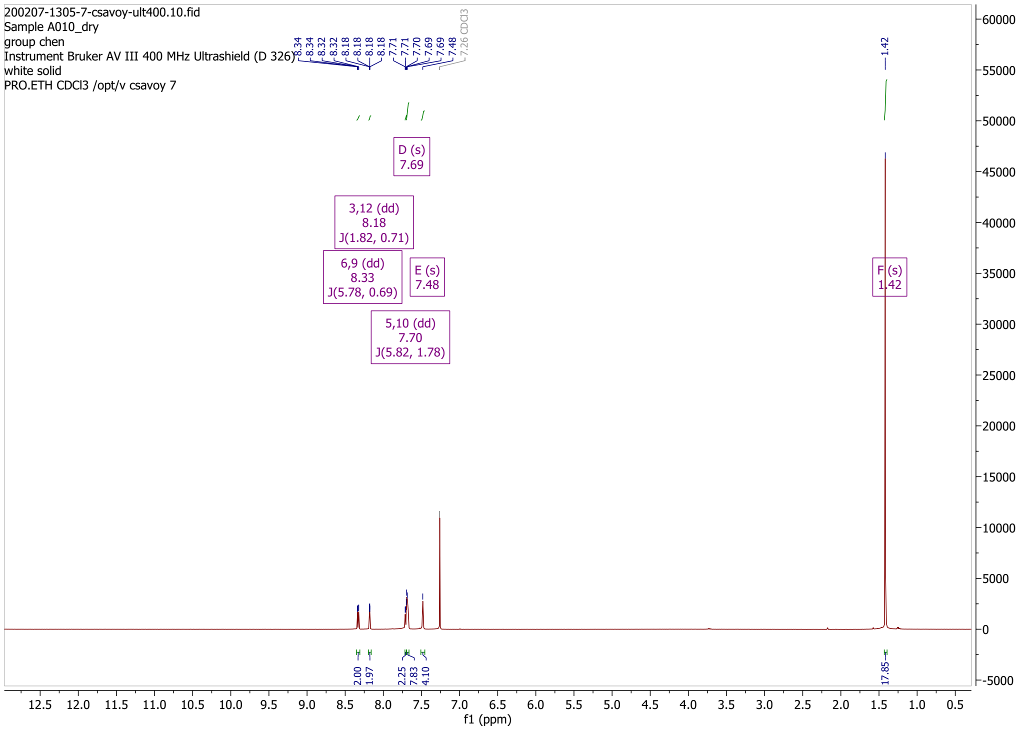

^{1}H-NMR of **3b** in $CDCl_3$

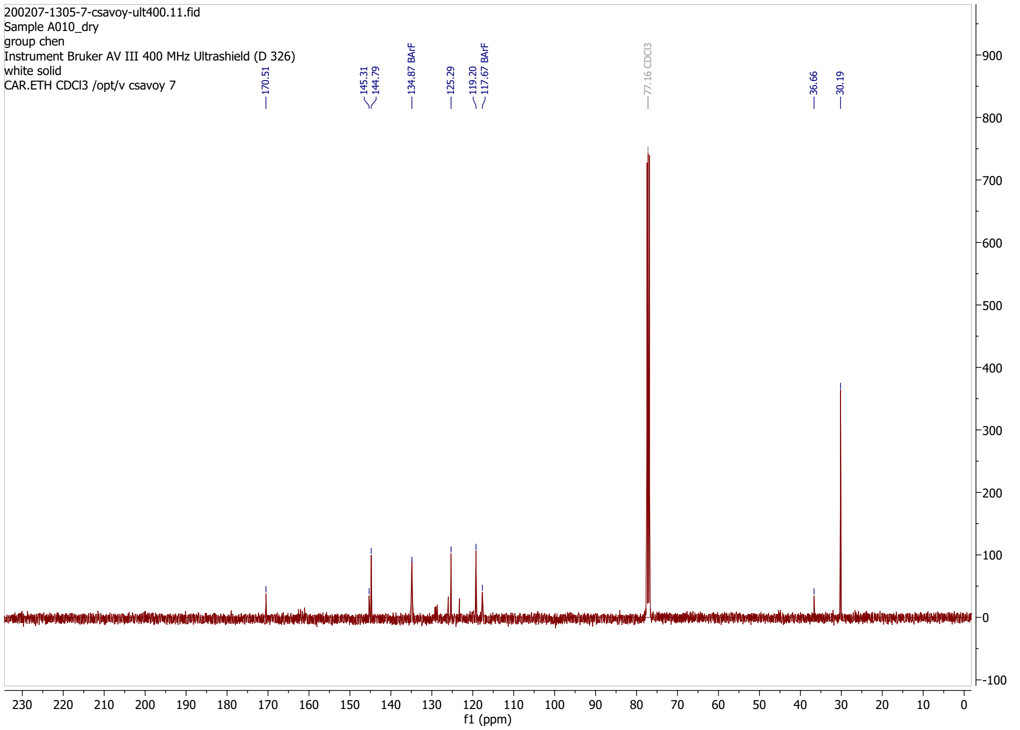

^{13}C-NMR of **3b** in $CDCl_3$

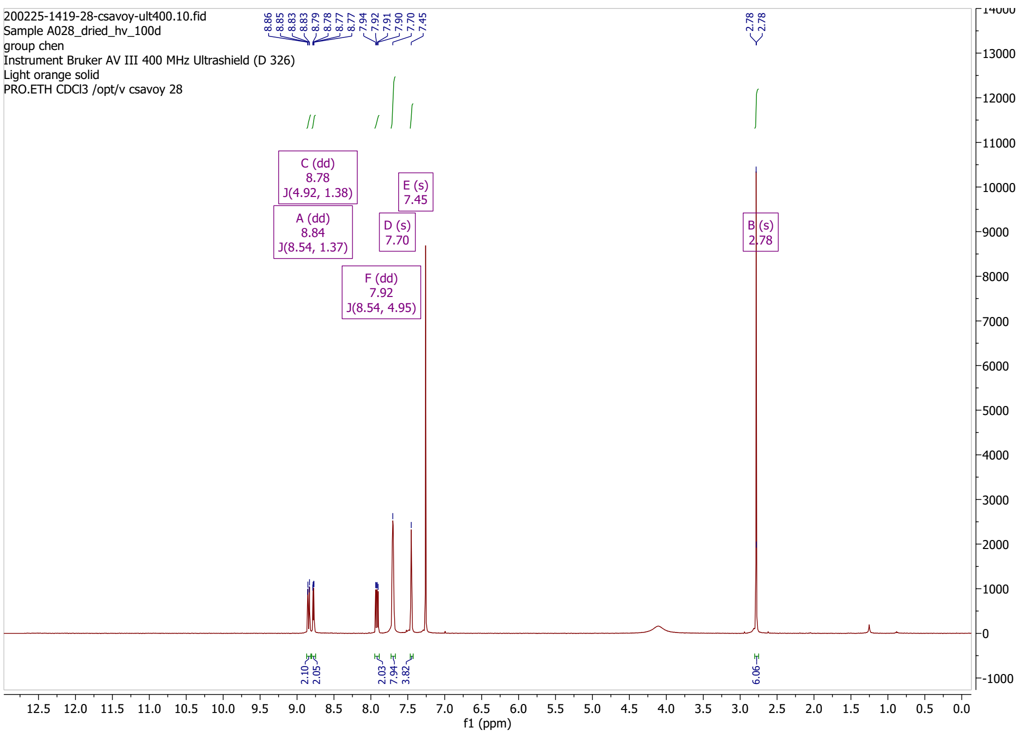

^{1}H-NMR of **4b** in $CDCl_3$

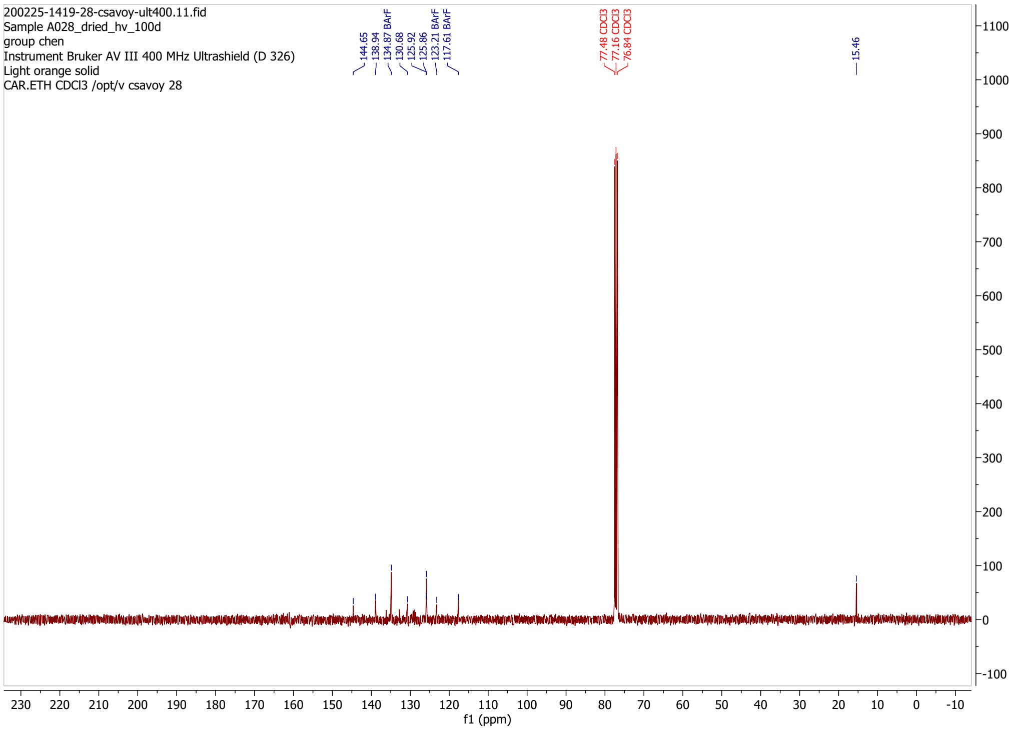

^{13}C-NMR of **4b** in $CDCl_3$

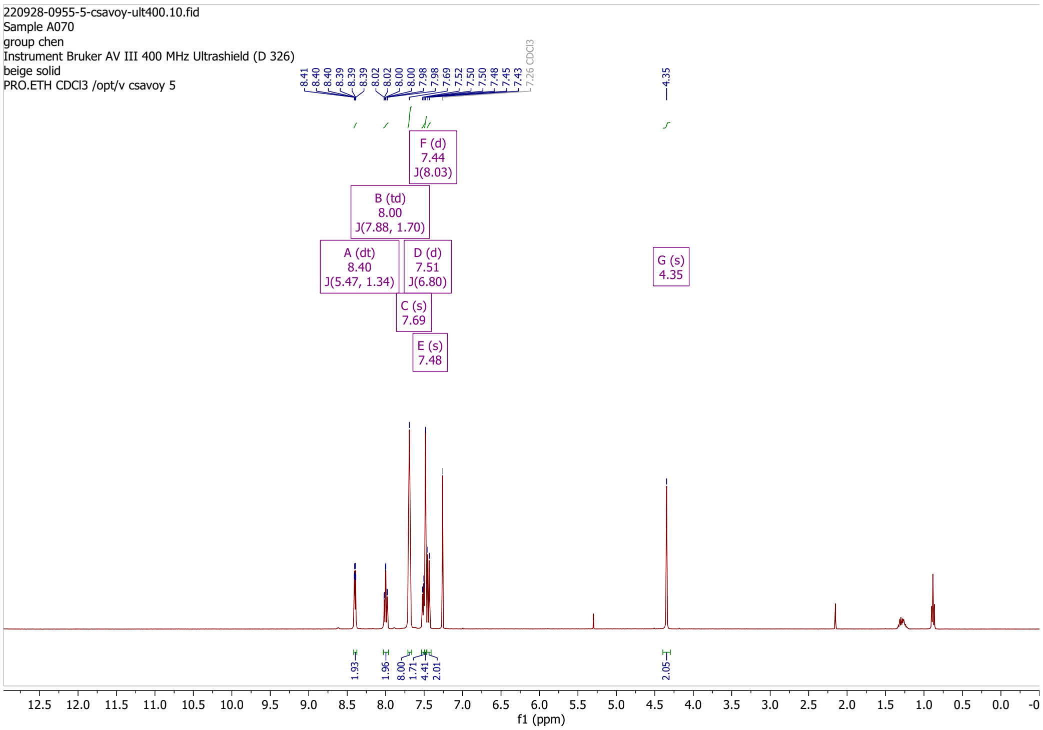

^{1}H-NMR of **5b** in $CDCl_3$

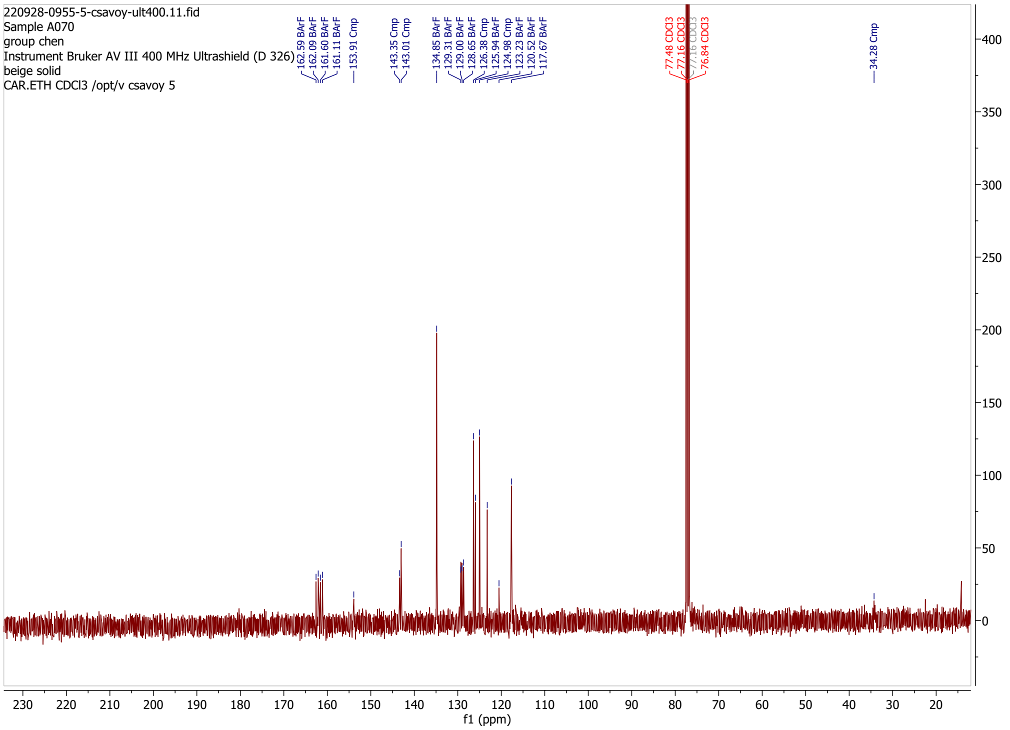

^{13}C-NMR of **5b** in $CDCl_3$

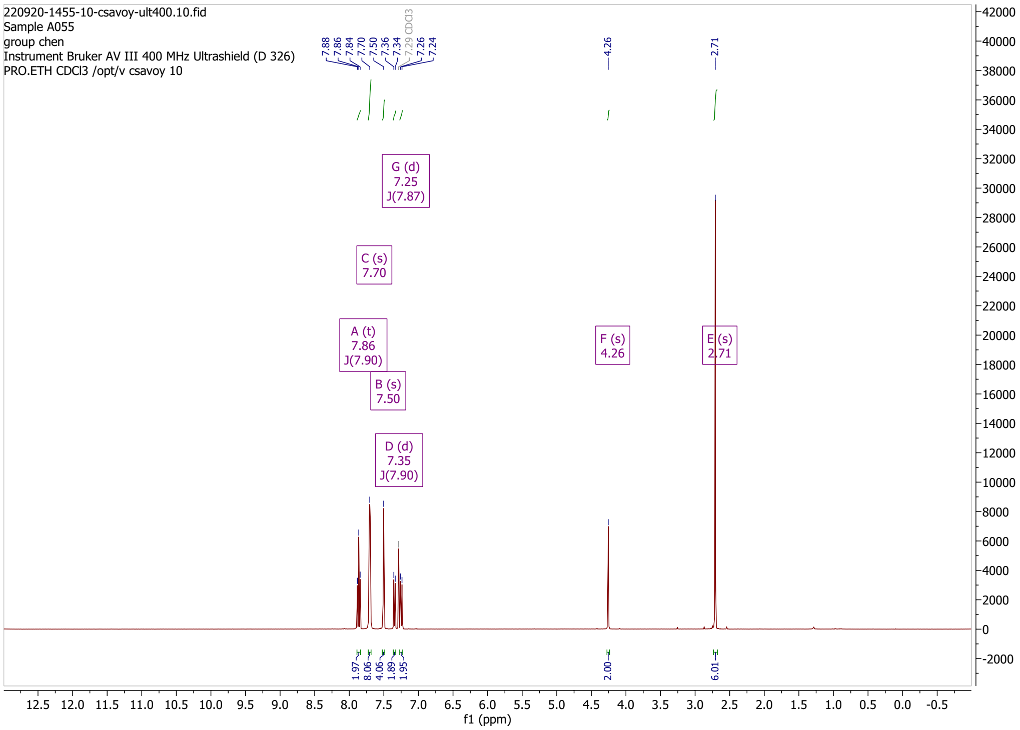

^{1}H-NMR of **6b** in $CDCl_3$

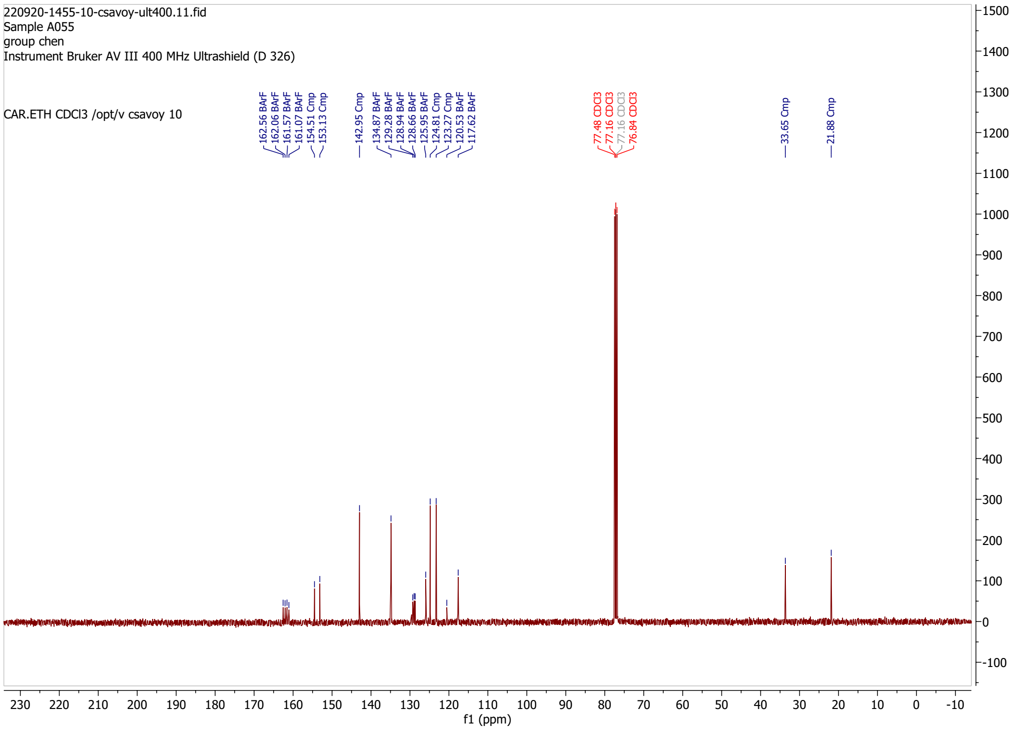

^{13}C-NMR of **6b** in $CDCl_3$

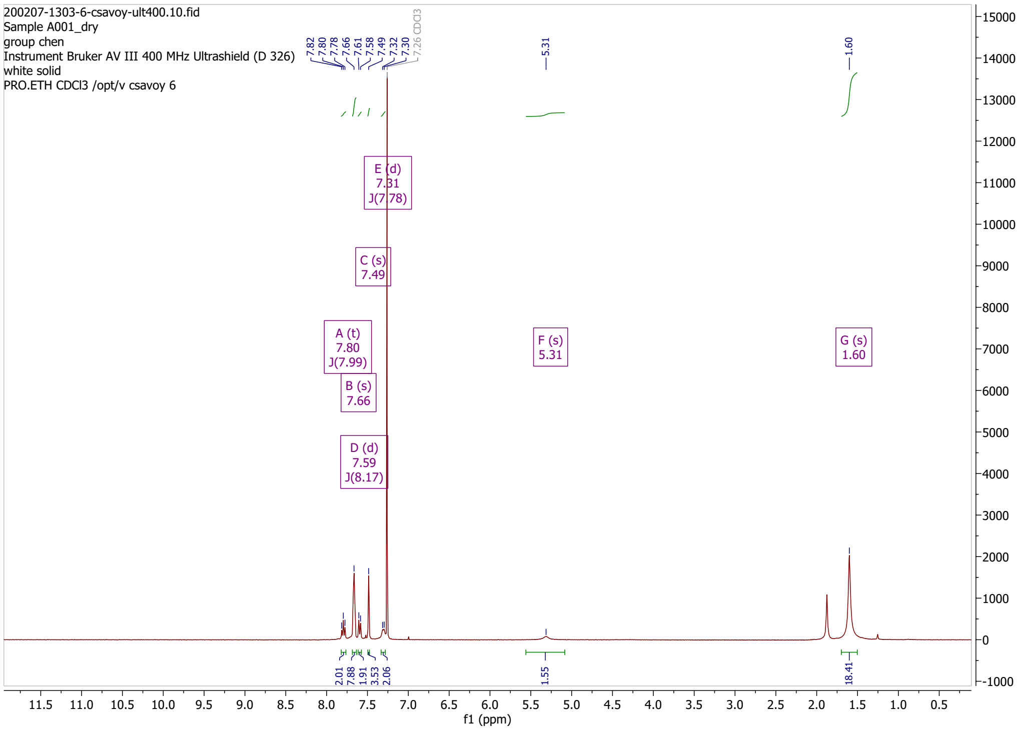

^{1}H-NMR of **7b** in $CDCl_3$

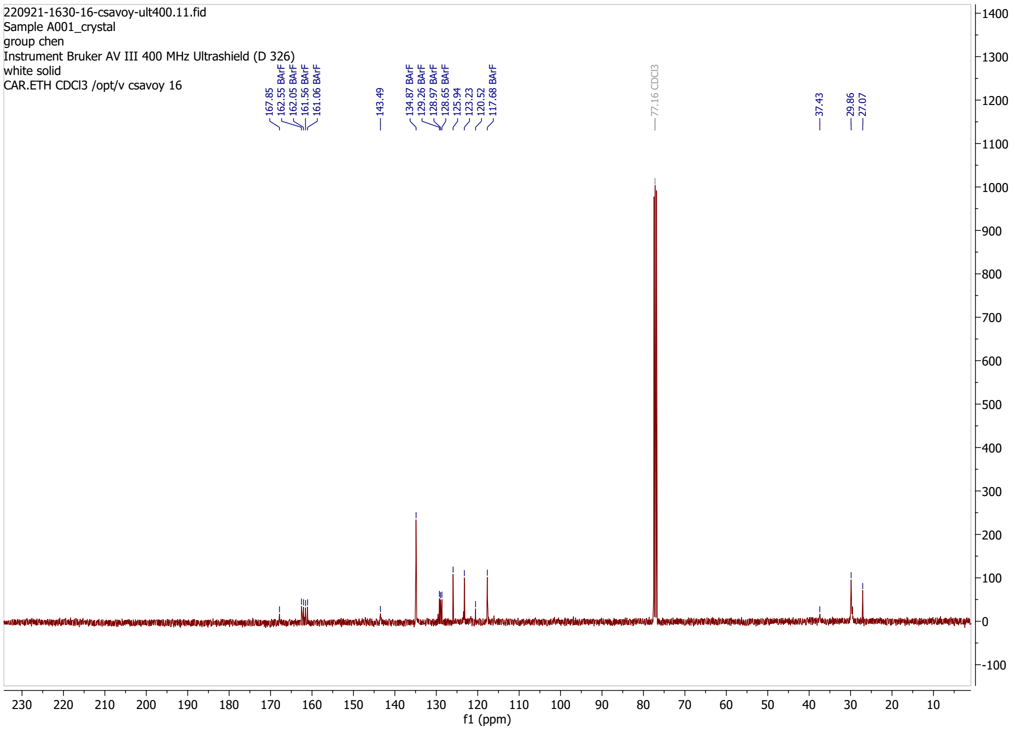

^{13}C-NMR of **7b** in $CDCl_3$

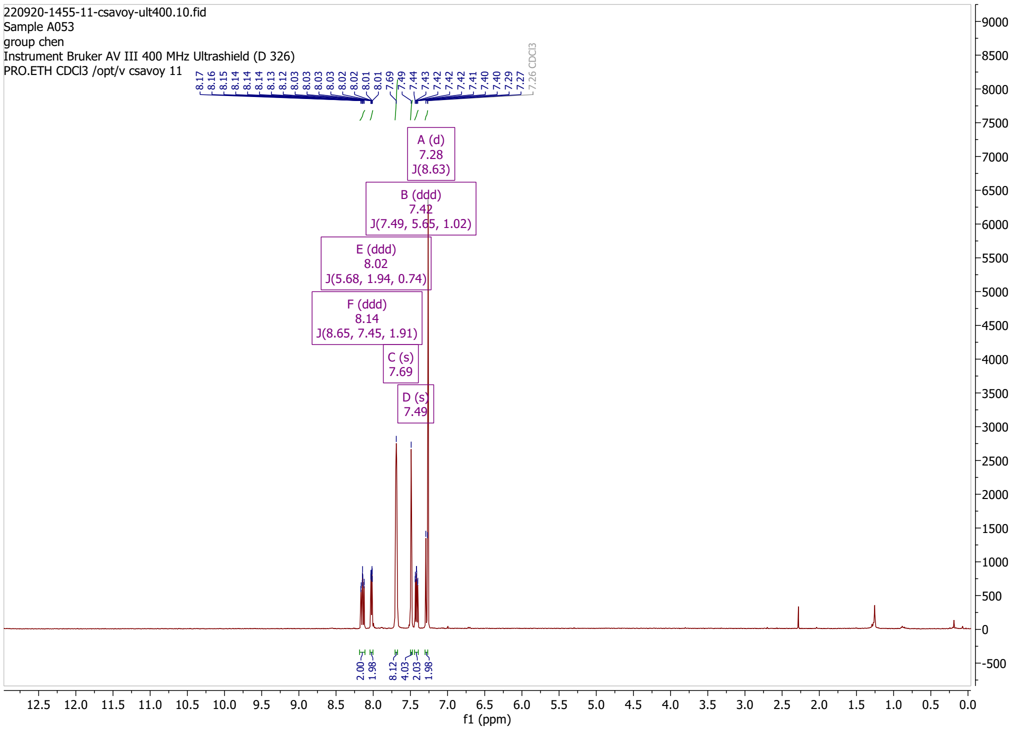

^{1}H-NMR of **8b** in $CDCl_3$

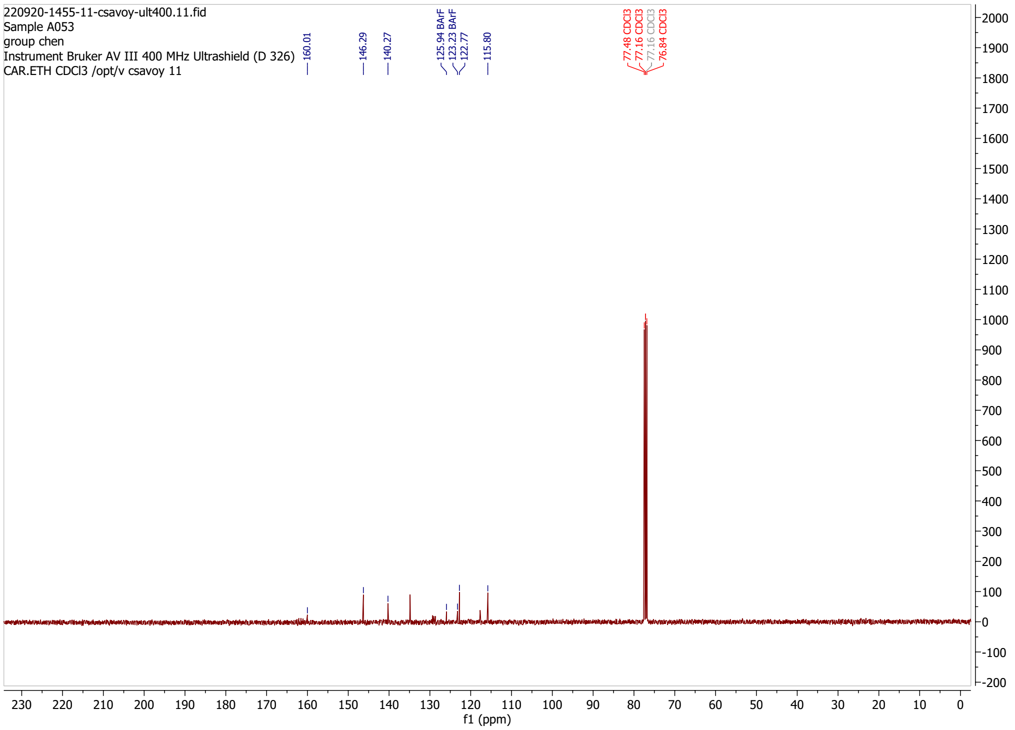

^{13}C-NMR of **8b** in $CDCl_3$

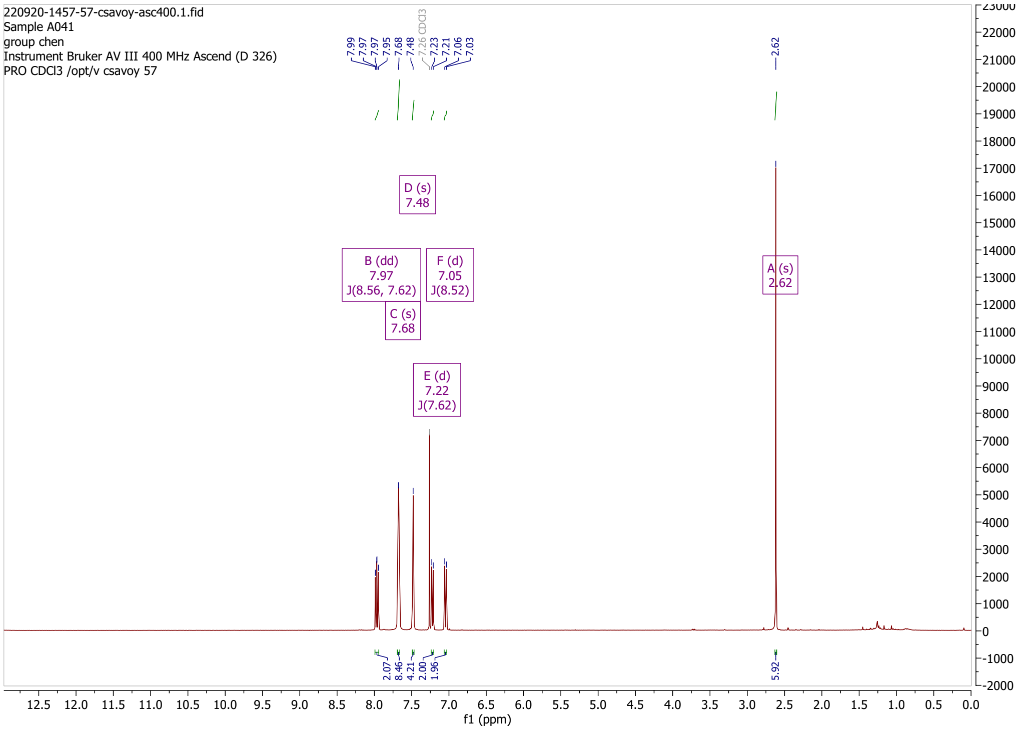

^{1}H-NMR of **9b** in $CDCl_3$

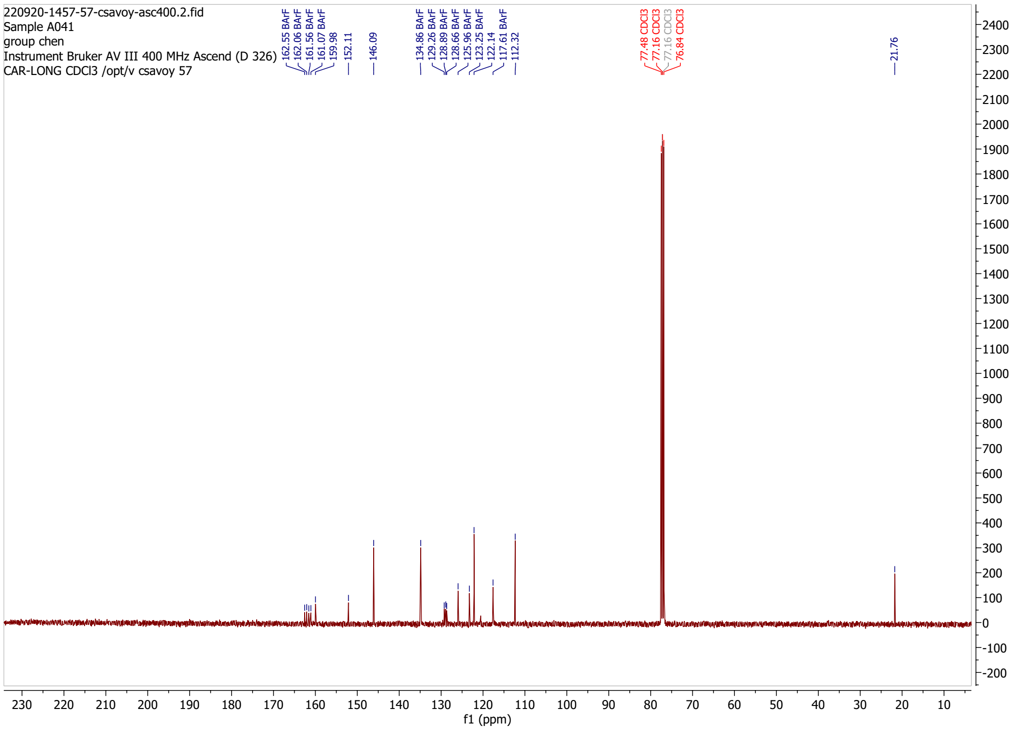

^{13}C-NMR of **9b** in $CDCl_3$

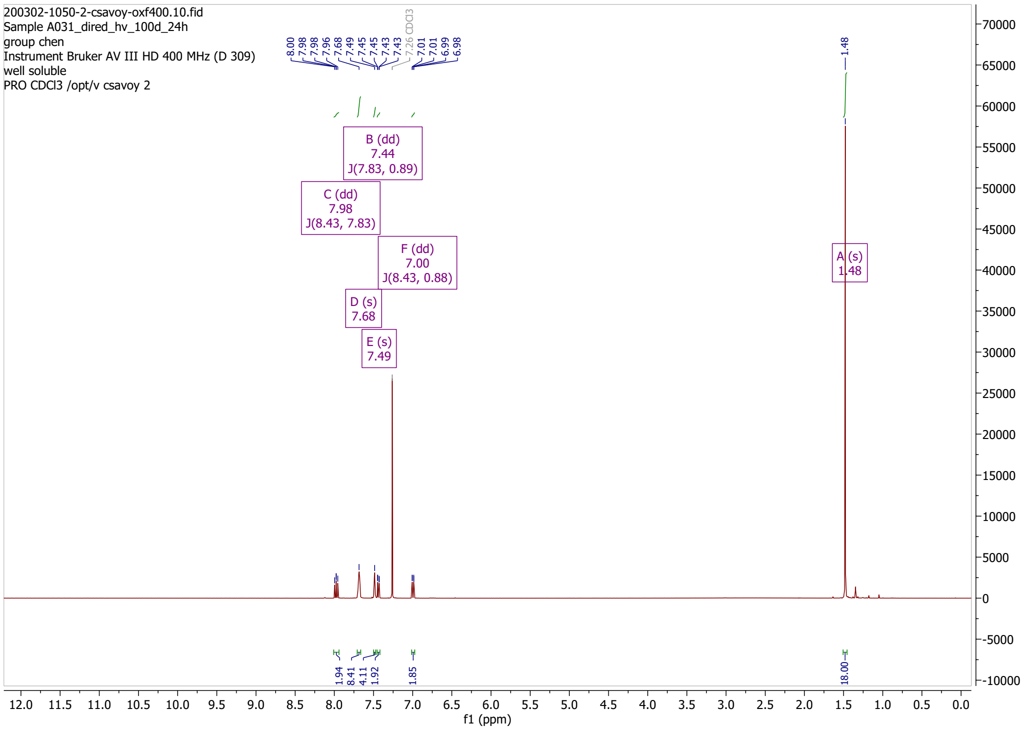

^{1}H-NMR of **10b** in $CDCl_3$

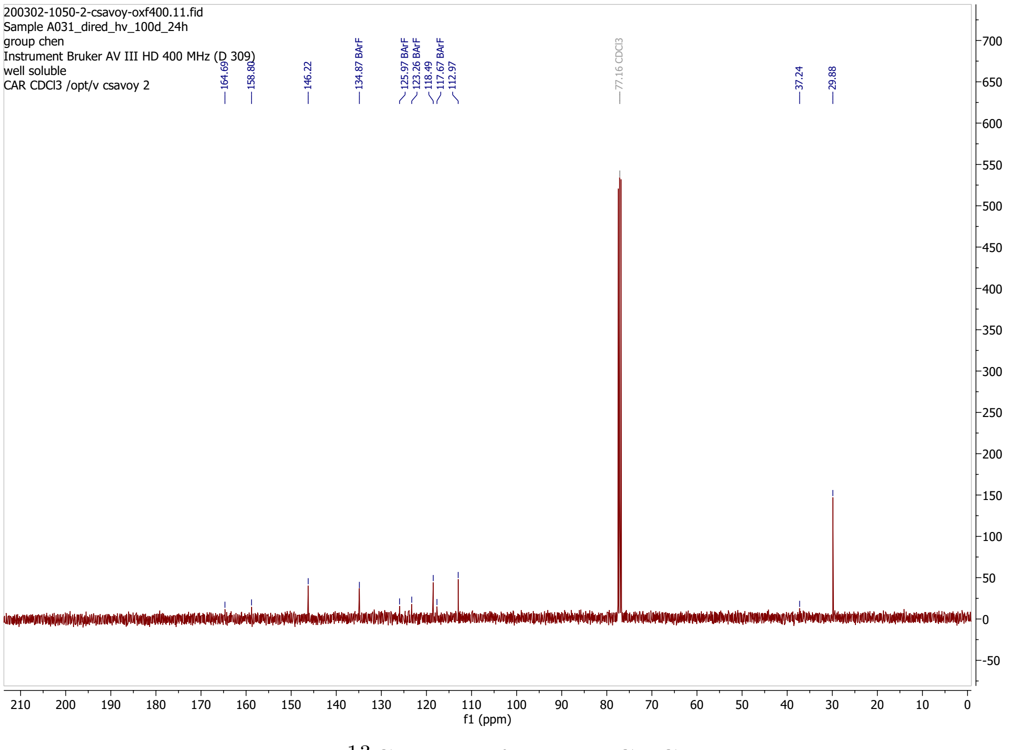

^{13}C-NMR of **10b** in $CDCl_3$

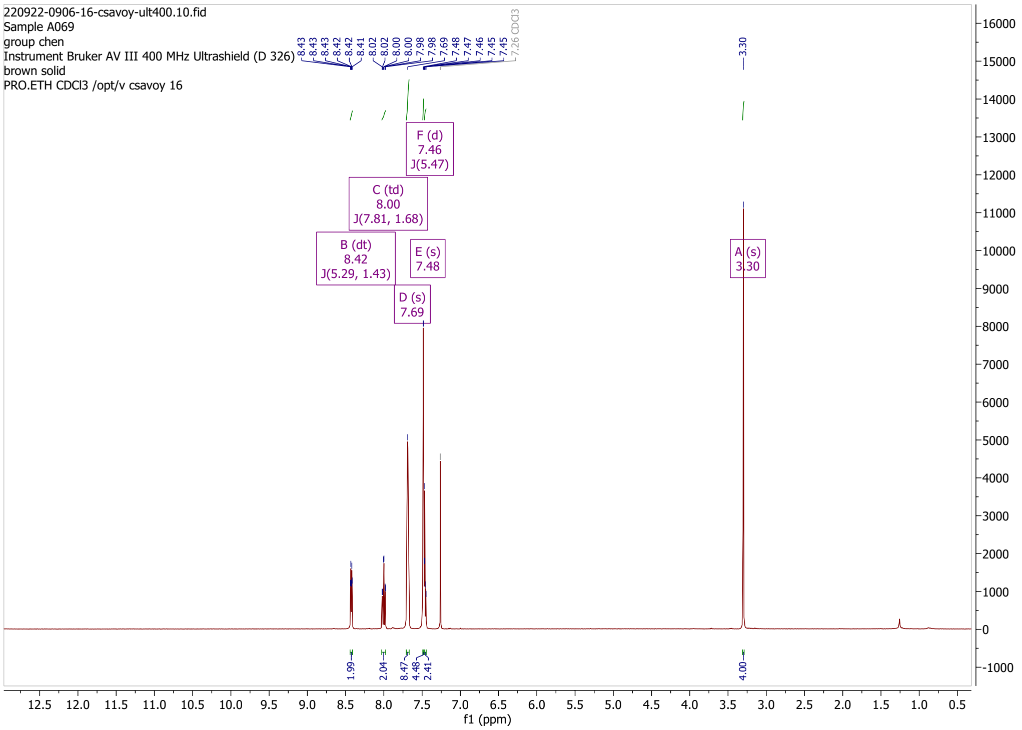

^{1}H-NMR of **11b** in $CDCl_3$

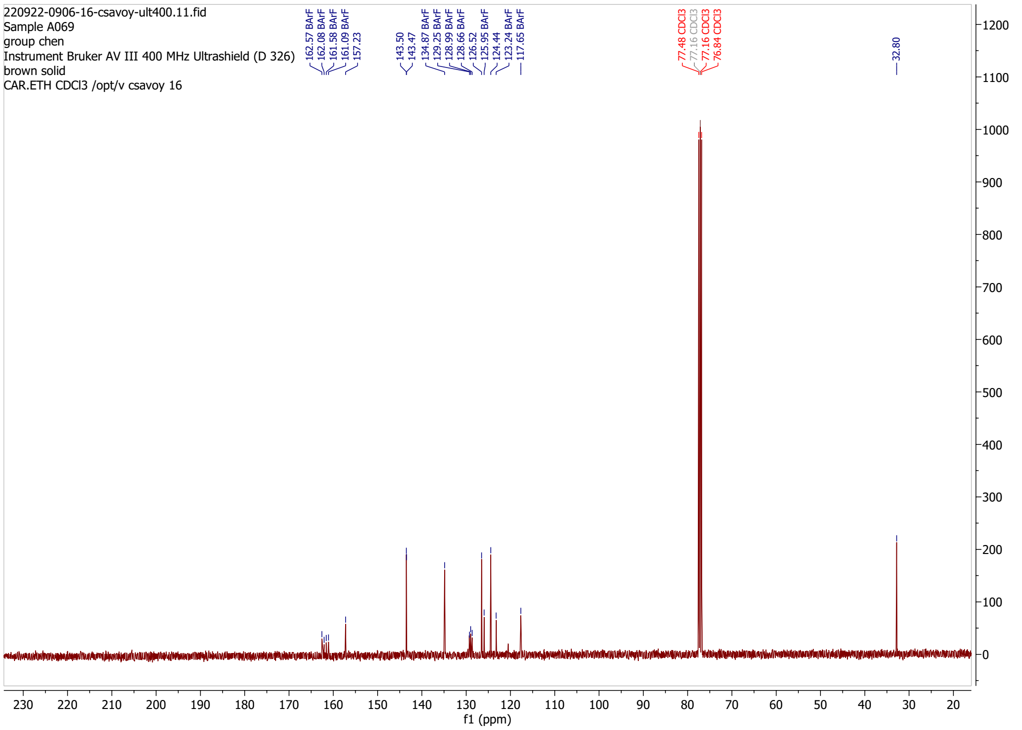

^{13}C-NMR of **11b** in $CDCl_3$

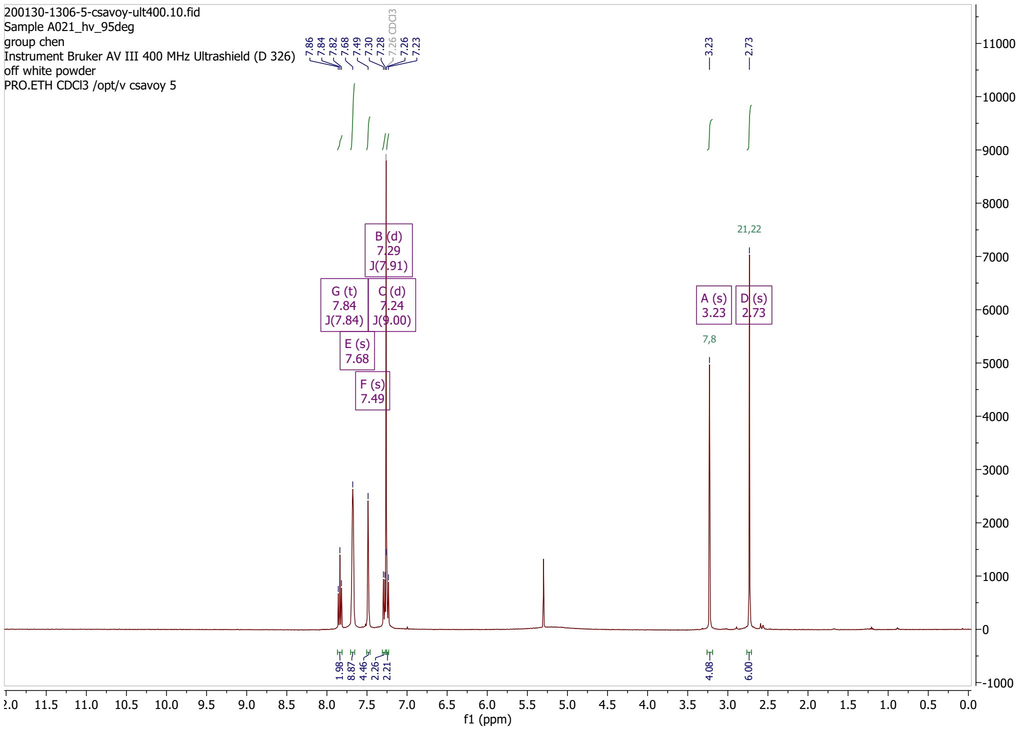

^{1}H-NMR of **12b** in $CDCl_3$

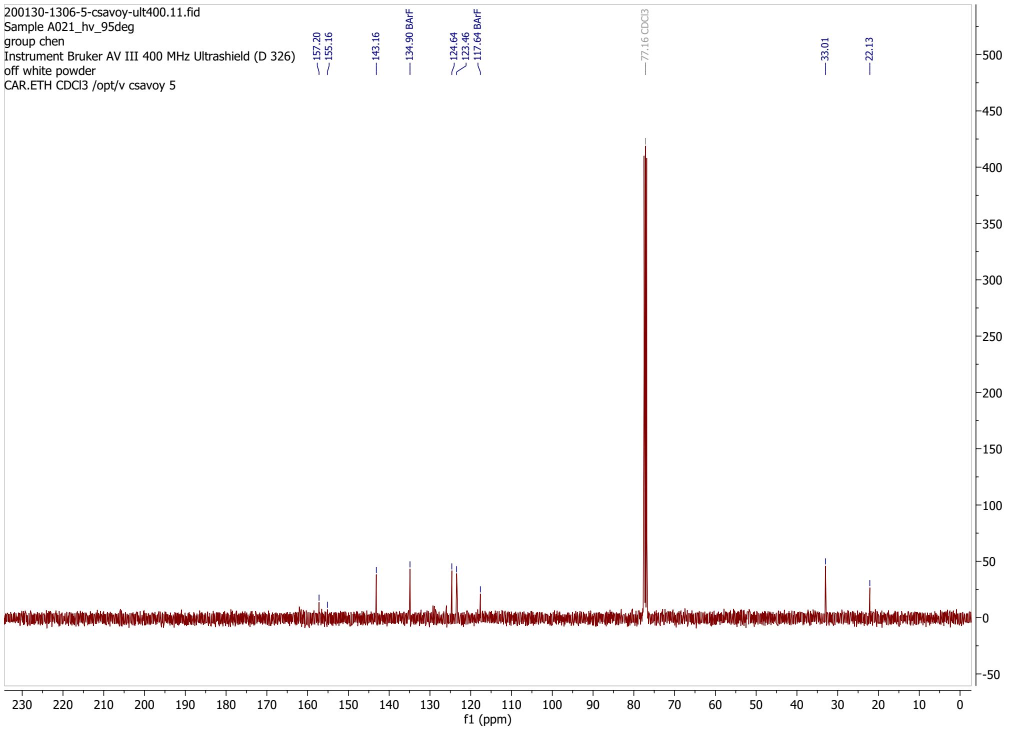

^{13}C-NMR of **12b** in $CDCl_3$

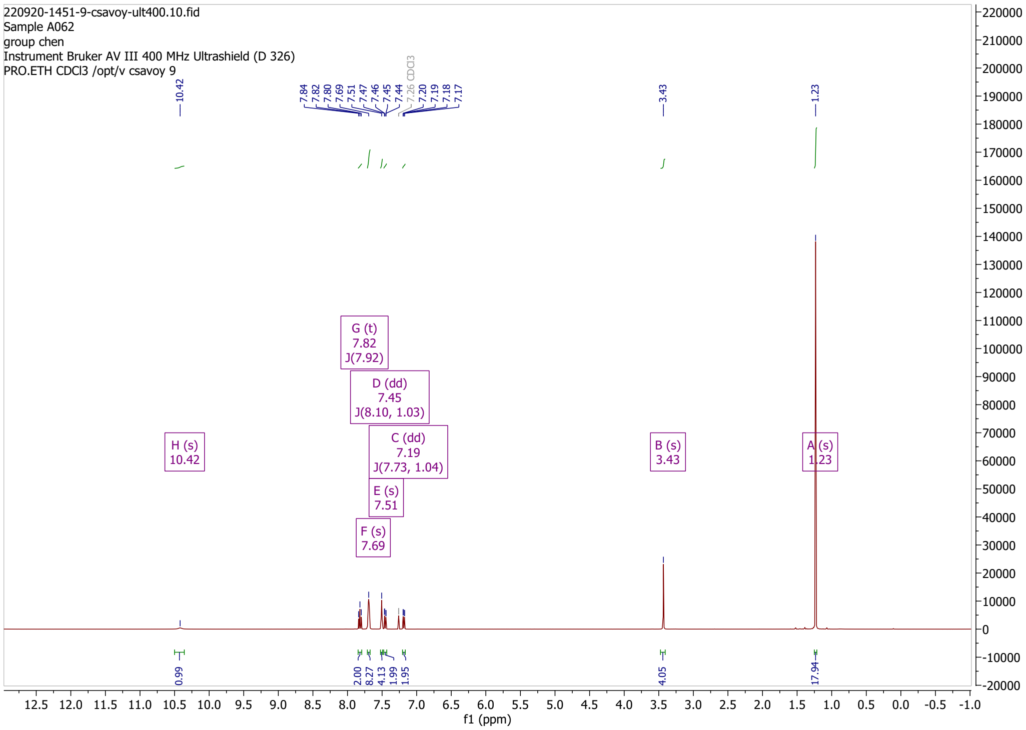

^{1}H-NMR of **13b** in $CDCl_3$

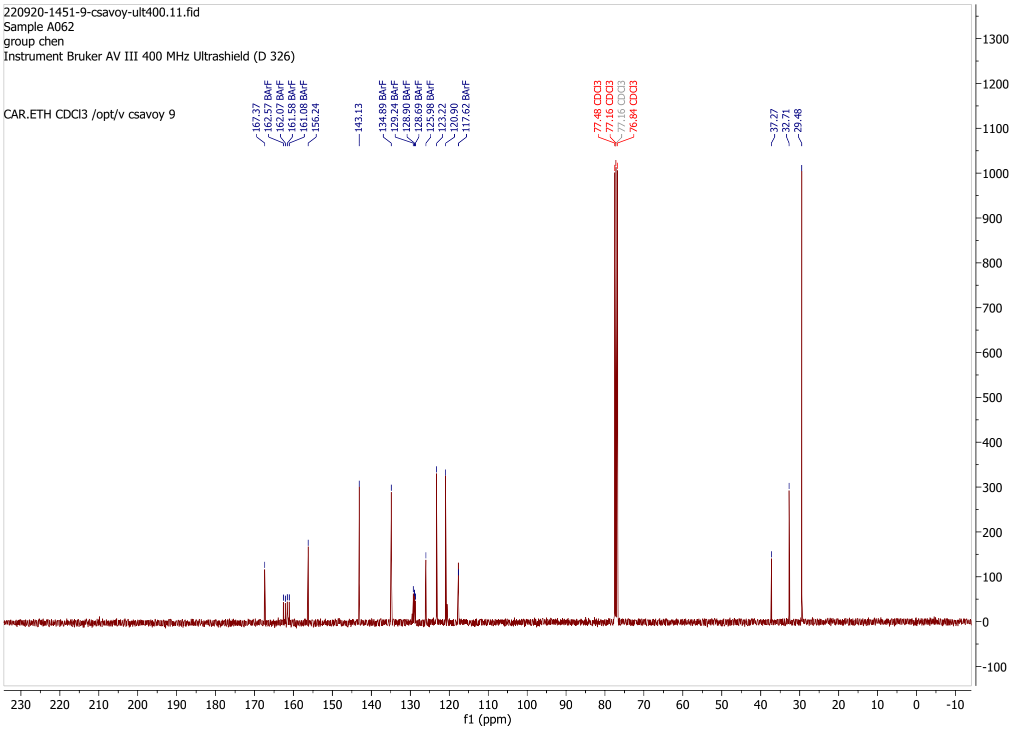

^{13}C-NMR of **13b** in $CDCl_3$

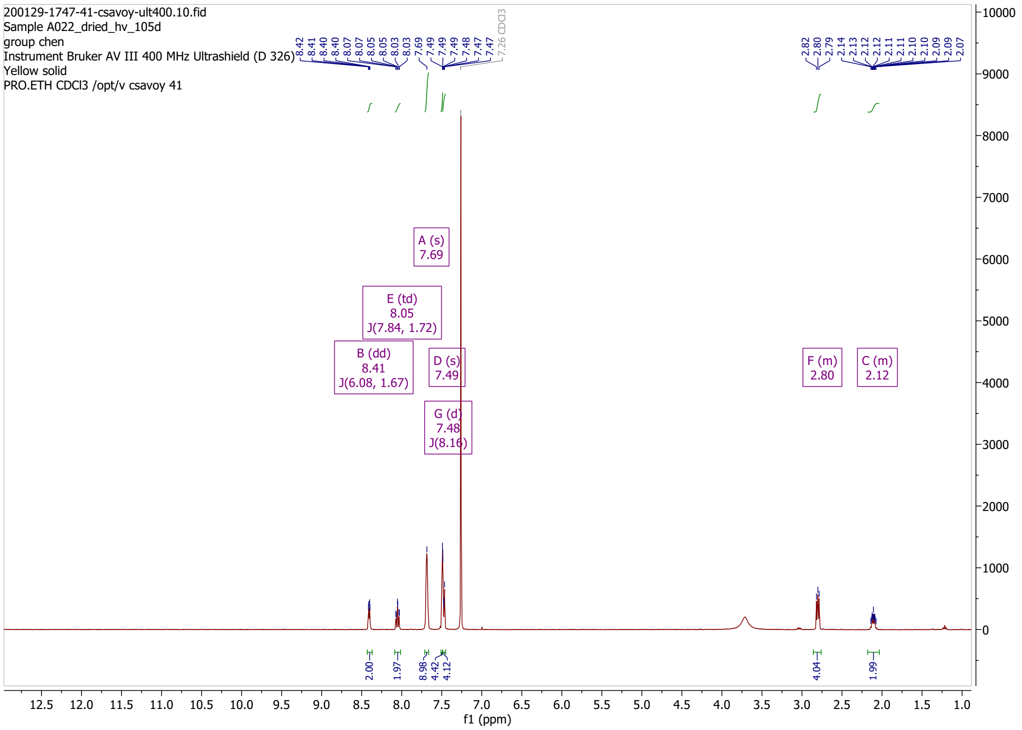

^{1}H-NMR of **14b** in $CDCl_3$

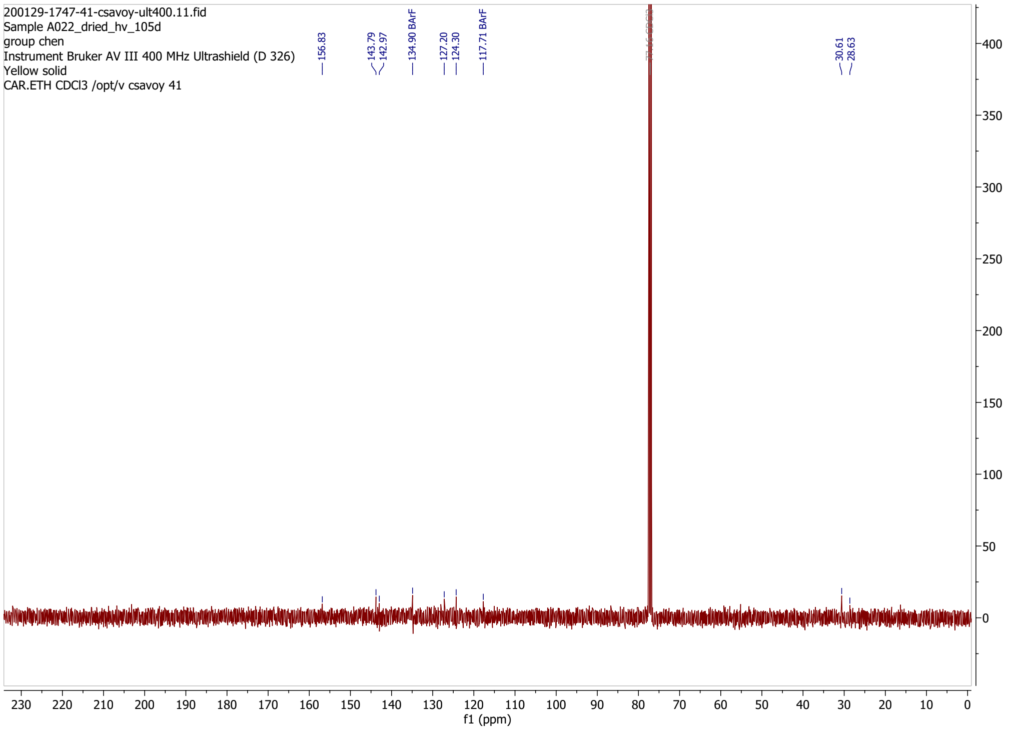

^{13}C-NMR of **14b** in $CDCl_3$

3.4 NMR spectra of ^{15}N tagged pyridines and bipyridines

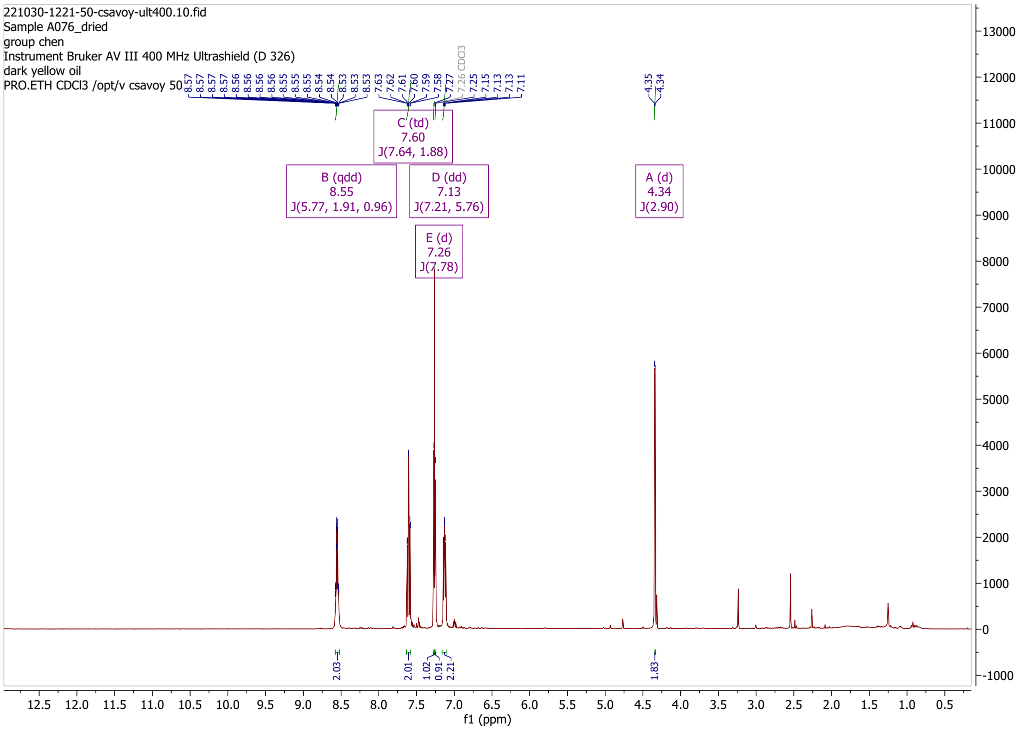

^{1}H-NMR of **t5a** in $CDCl_3$

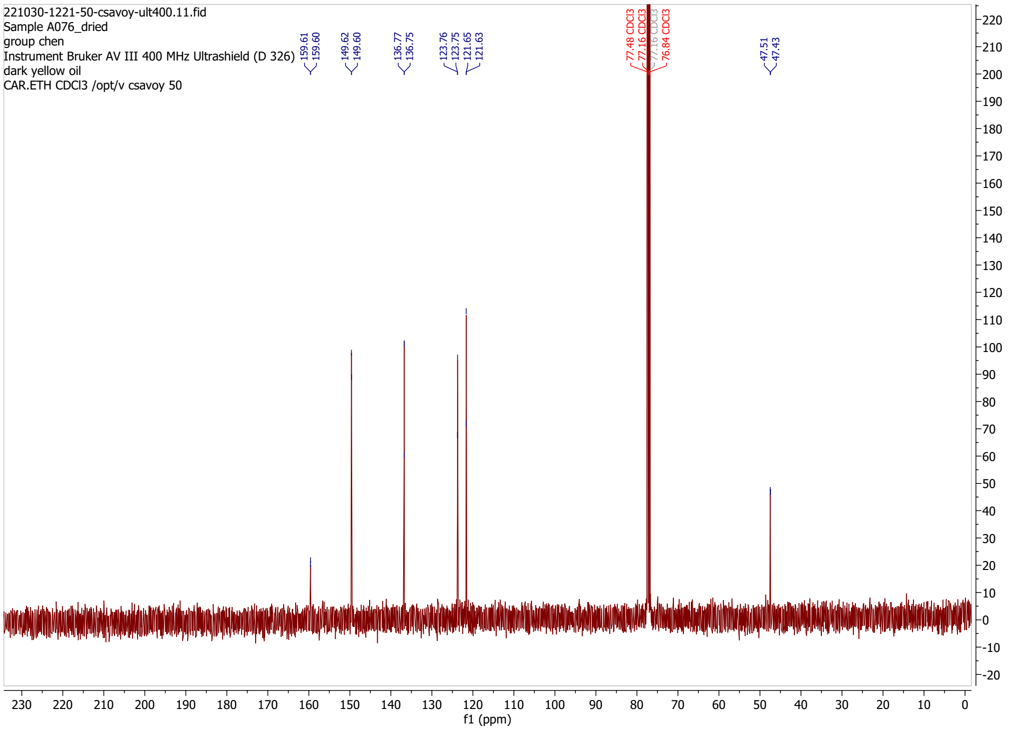

^{13}C-NMR of **t5a** in $CDCl_3$

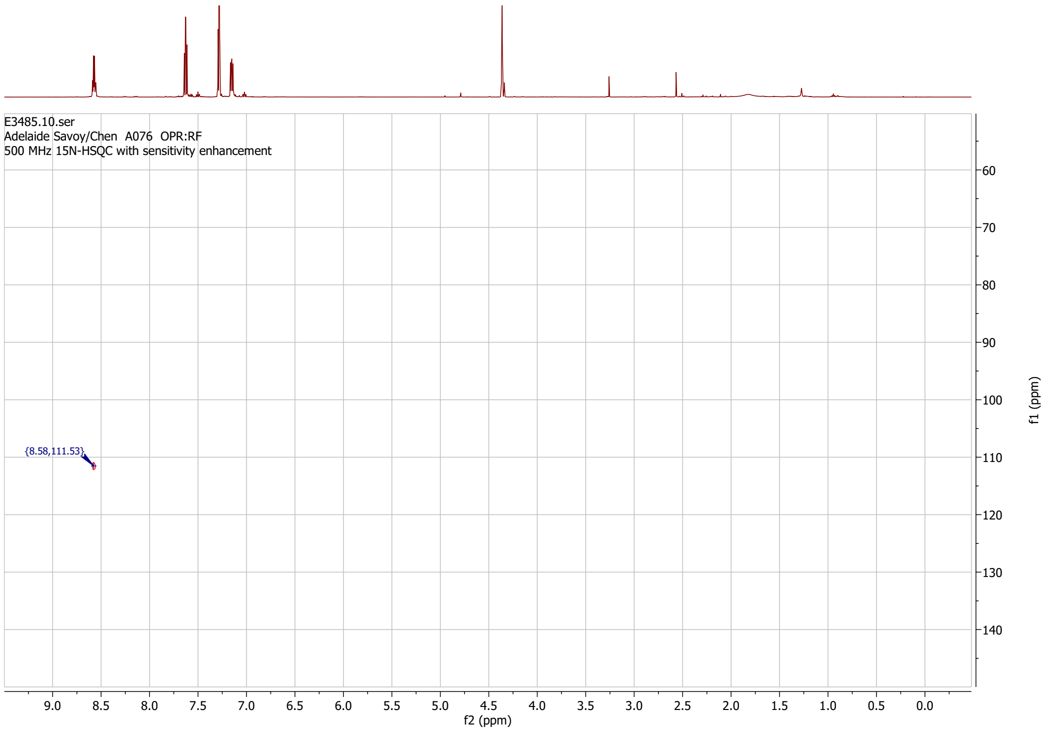

^{1}H-^{15}N-HSQC of **t5a** in $CDCl_3$

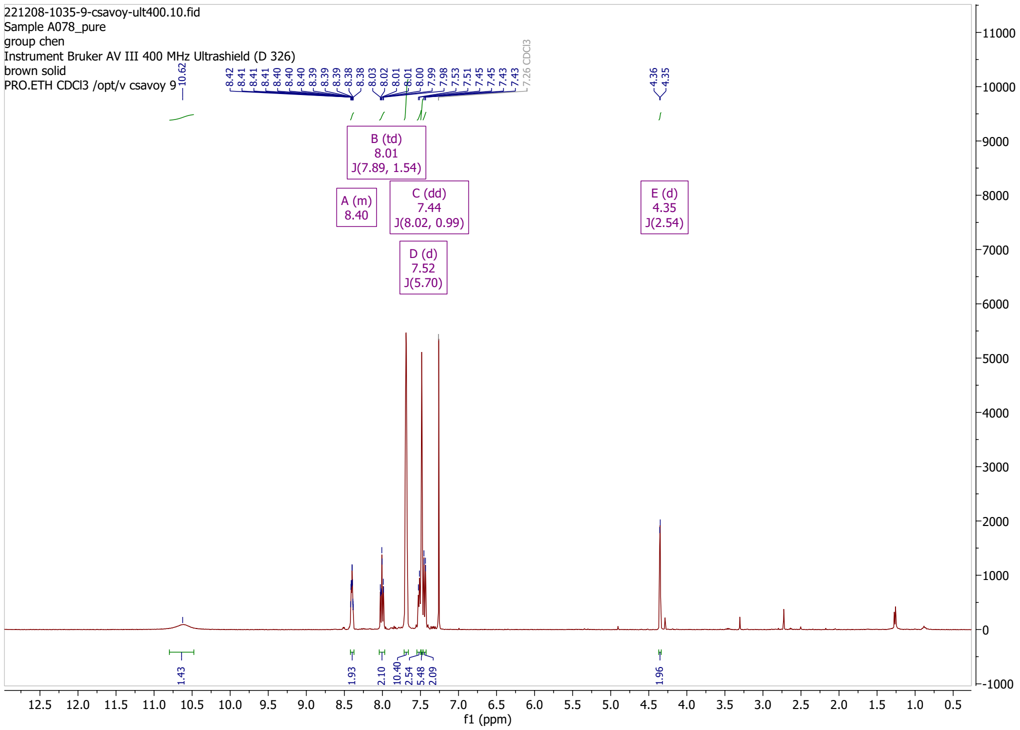

^{1}H-NMR of **t5b** in $CDCl_3$

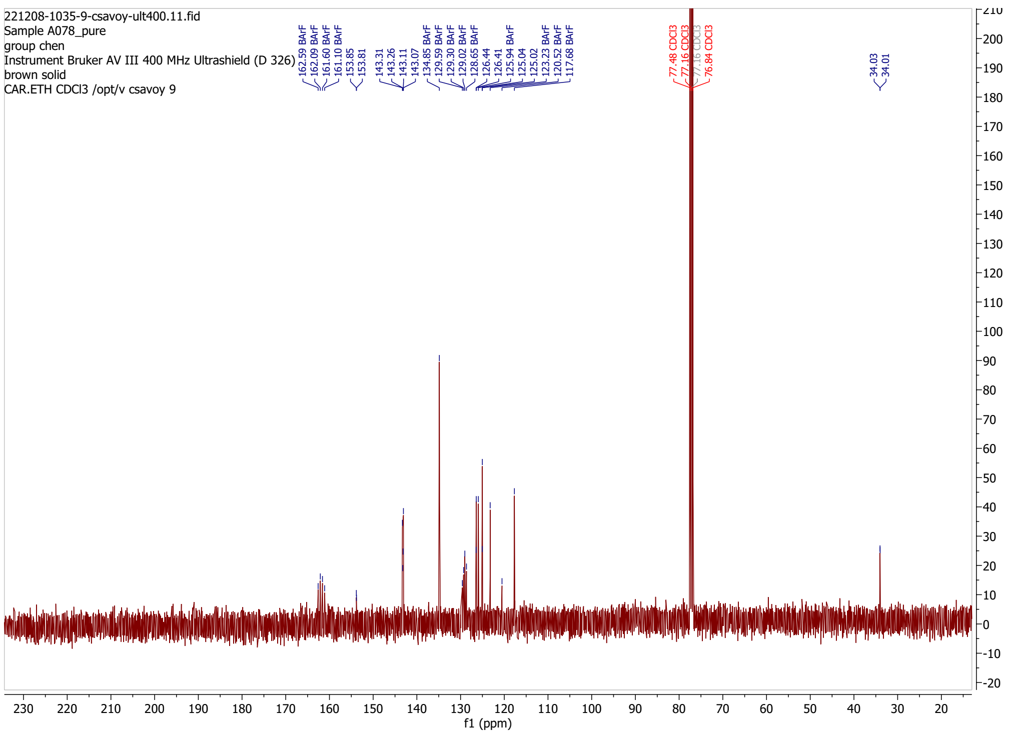

^{13}C-NMR of **t5b** in $CDCl_3$

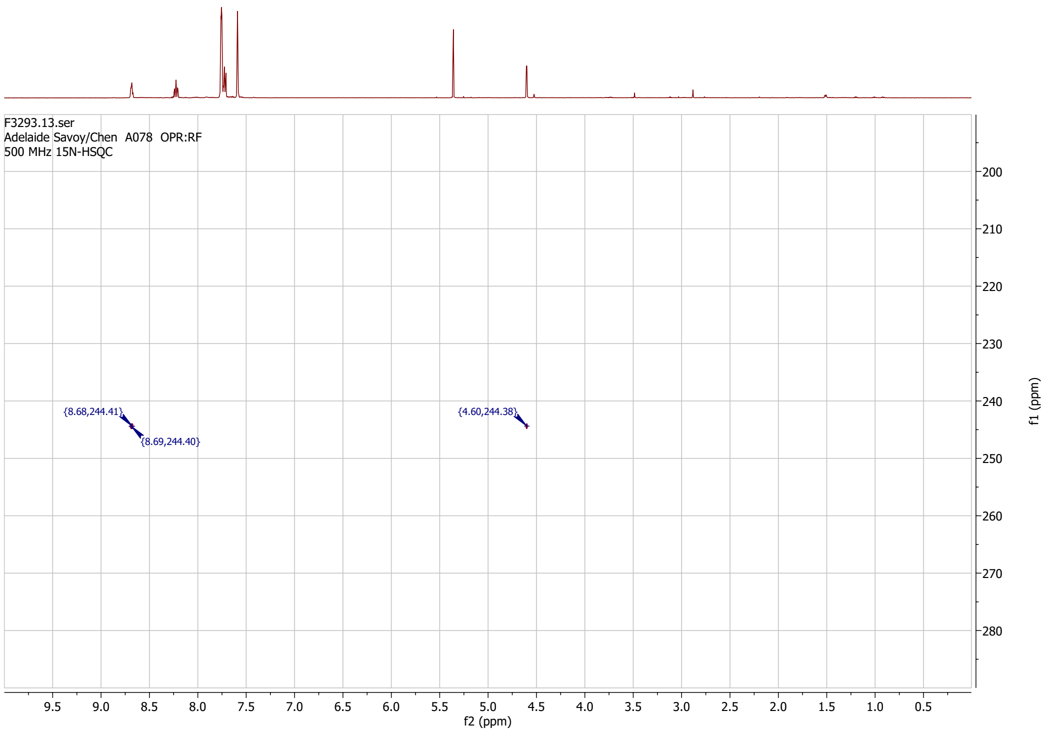

^{1}H-^{15}N-HSQC of **t5b** in CD_2Cl_2

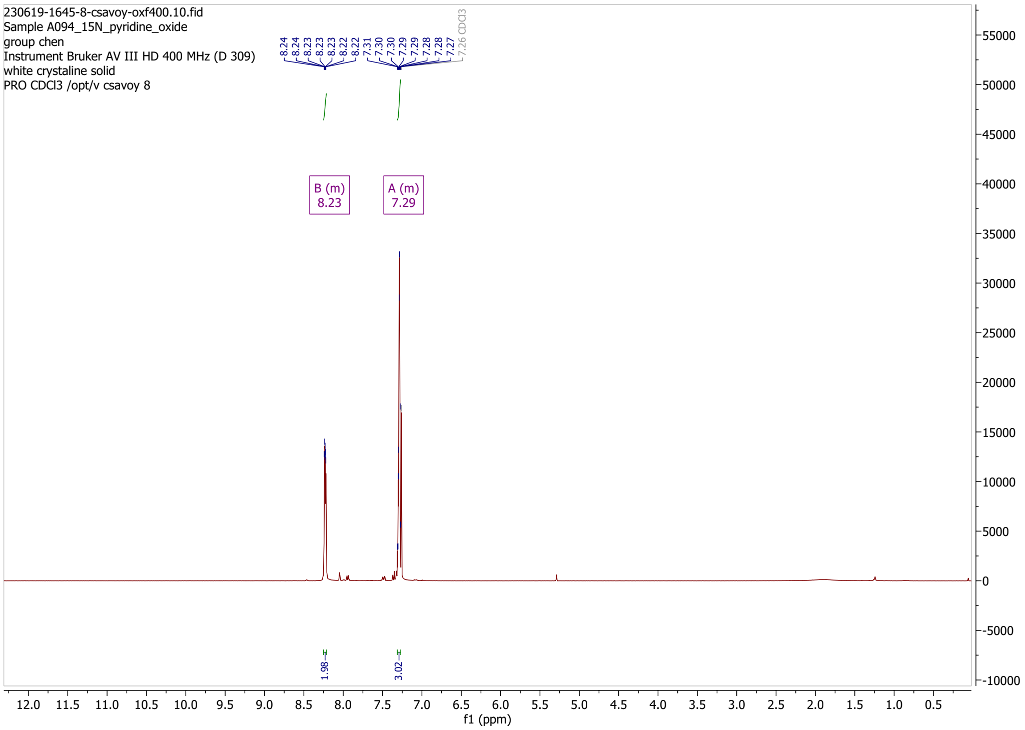

^{1}H-NMR of pyridine N-oxide in $CDCl_3$

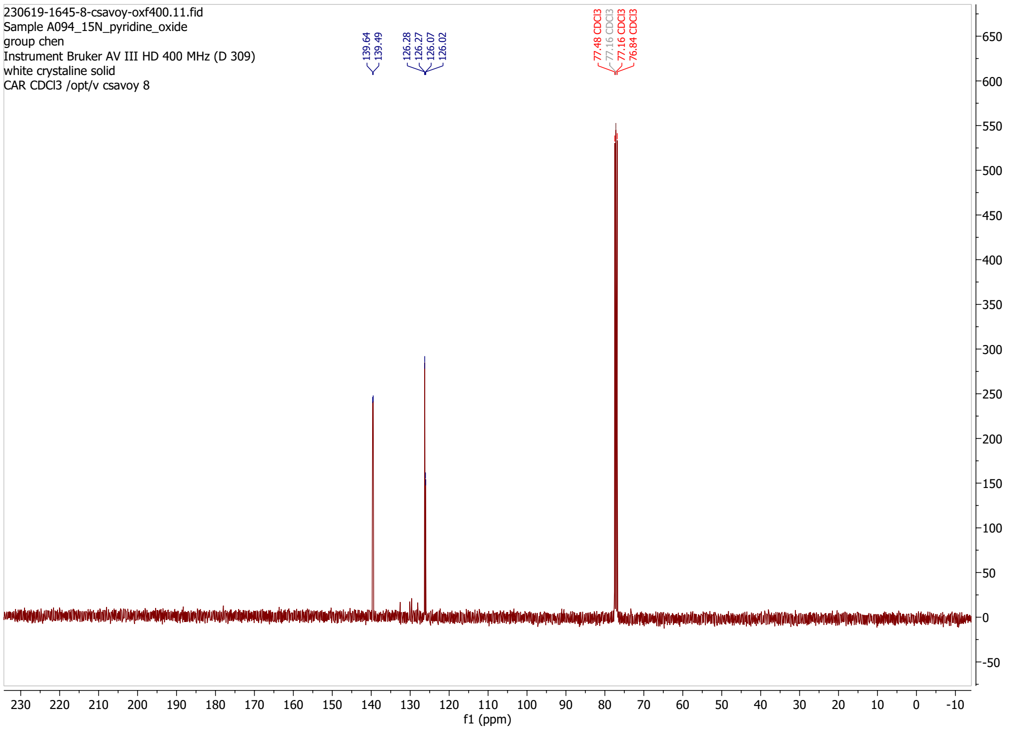

^{13}C-NMR of pyridine N-oxide in $CDCl_3$

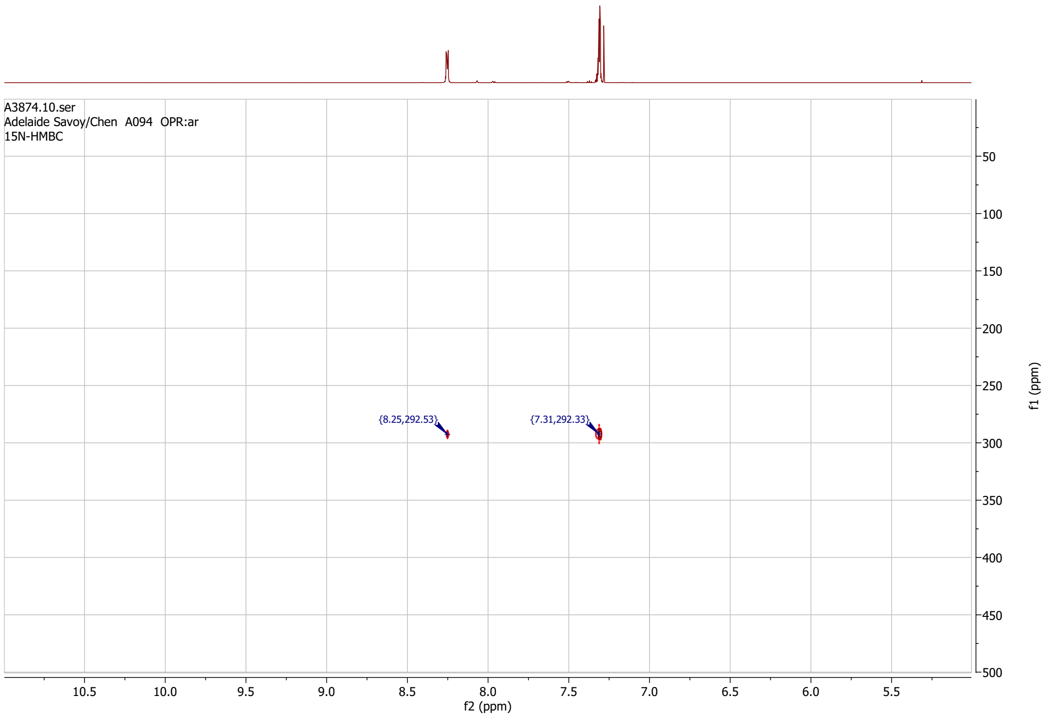

^{1}H-^{15}N-HMBC of pyridine N-oxide in $CDCl_3$

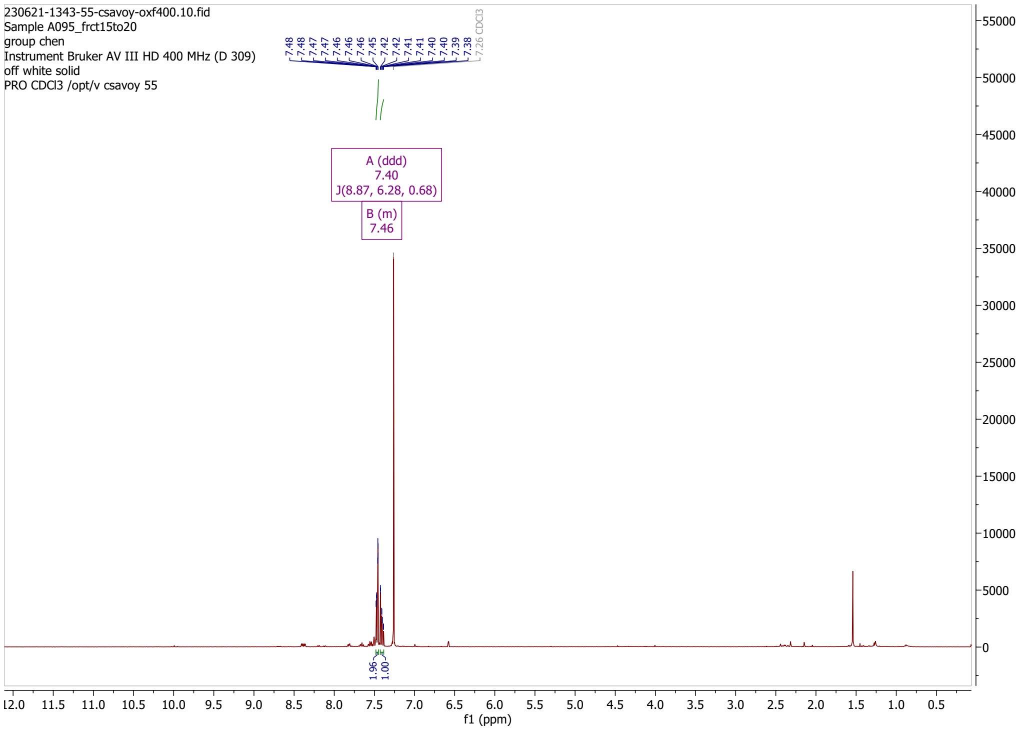

^{1}H-NMR of 2,6-dibromopyridine in $CDCl_3$

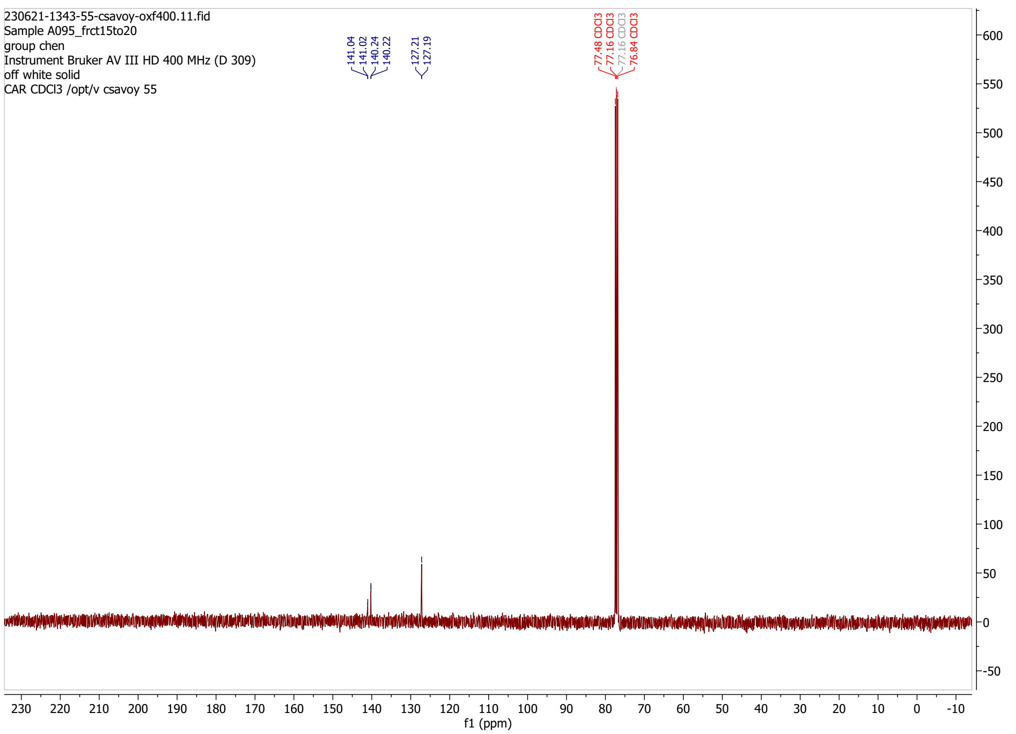

^{13}C-NMR of 2,6-dibromopyridine in $CDCl_3$

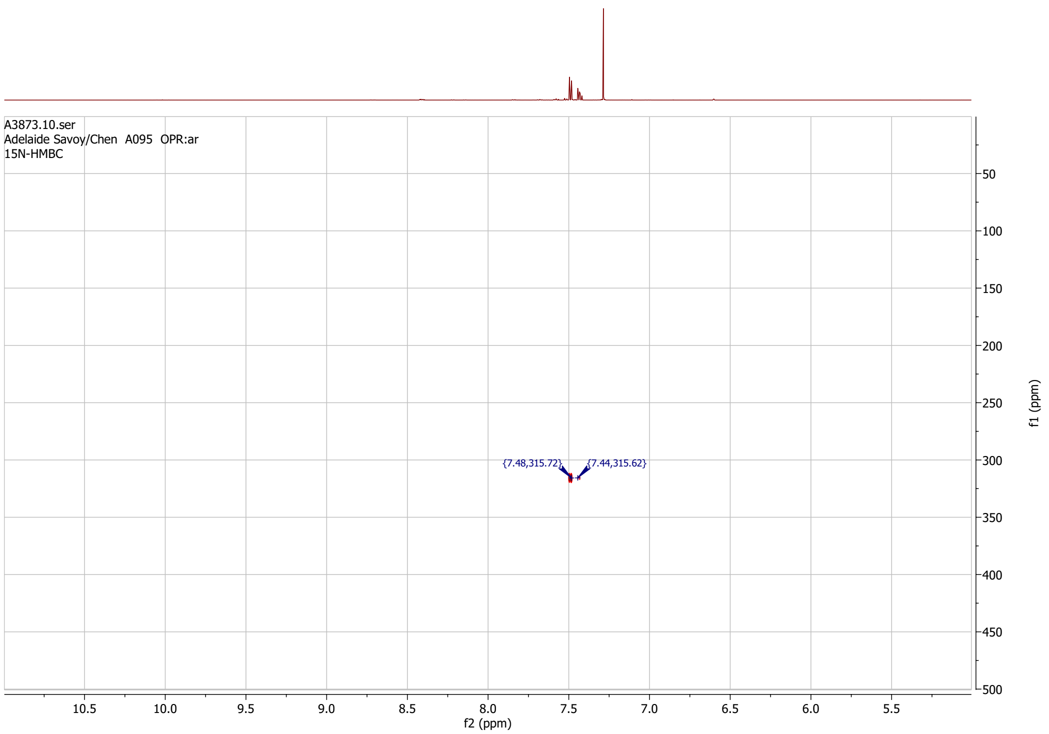

^{1}H-^{15}N-HMBC of 2,6-dibromopyridine in $CDCl_3$

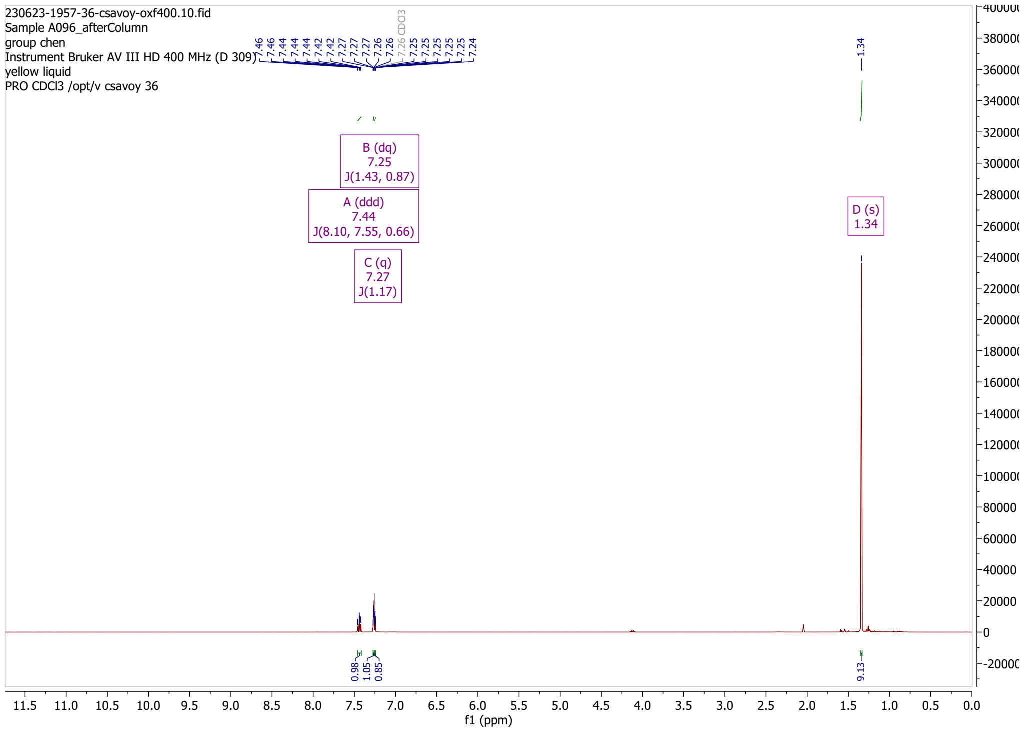

^{1}H-NMR of 2-bromo-6-(*tert*-butyl)pyridine in $CDCl_3$

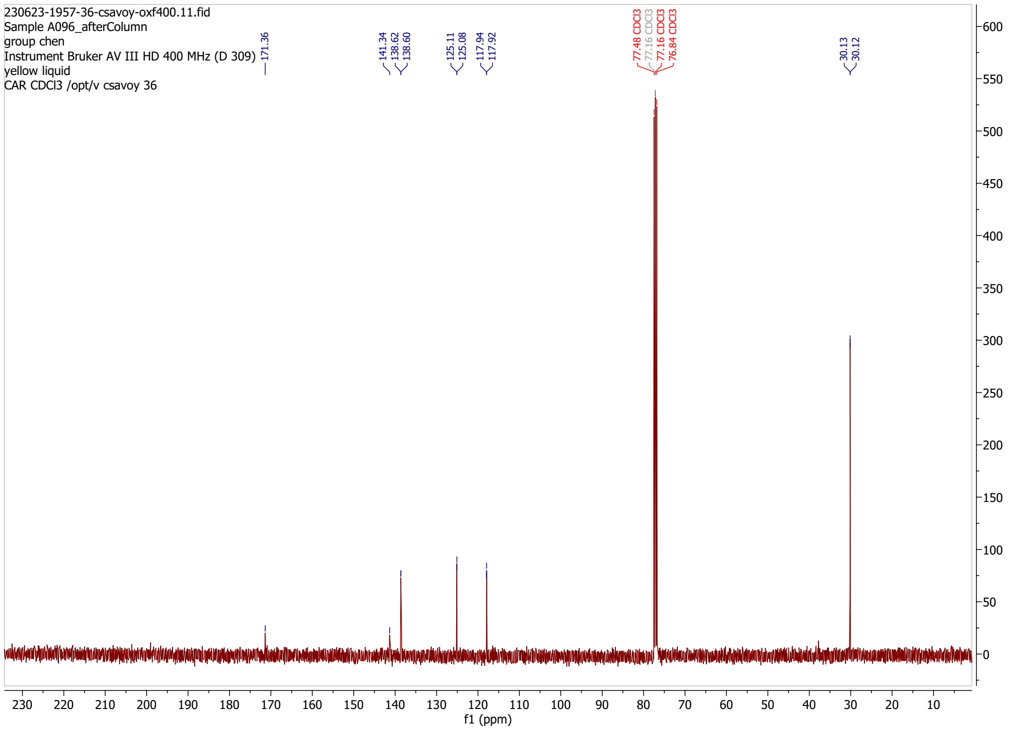

^{13}C-NMR of 2-bromo-6-(*tert*-butyl)pyridine in $CDCl_3$

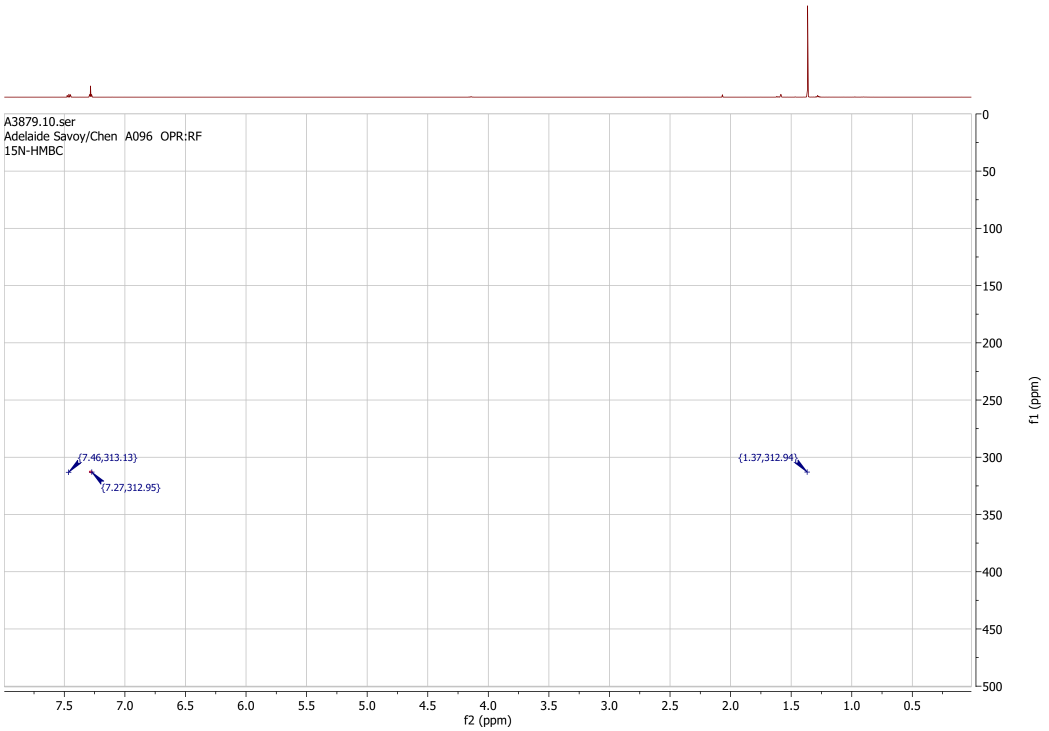

^{1}H-^{15}N-HMBC of 2-bromo-6-(*tert*-butyl)pyridine in $CDCl_3$

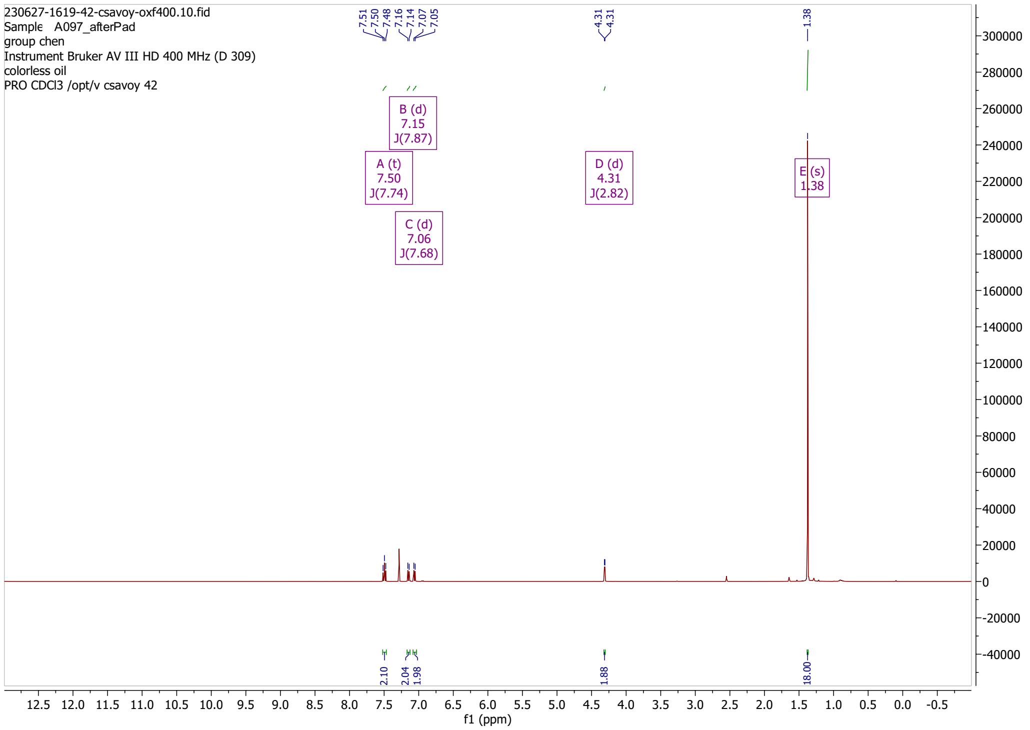

^{1}H-NMR of **t7a** in $CDCl_3$

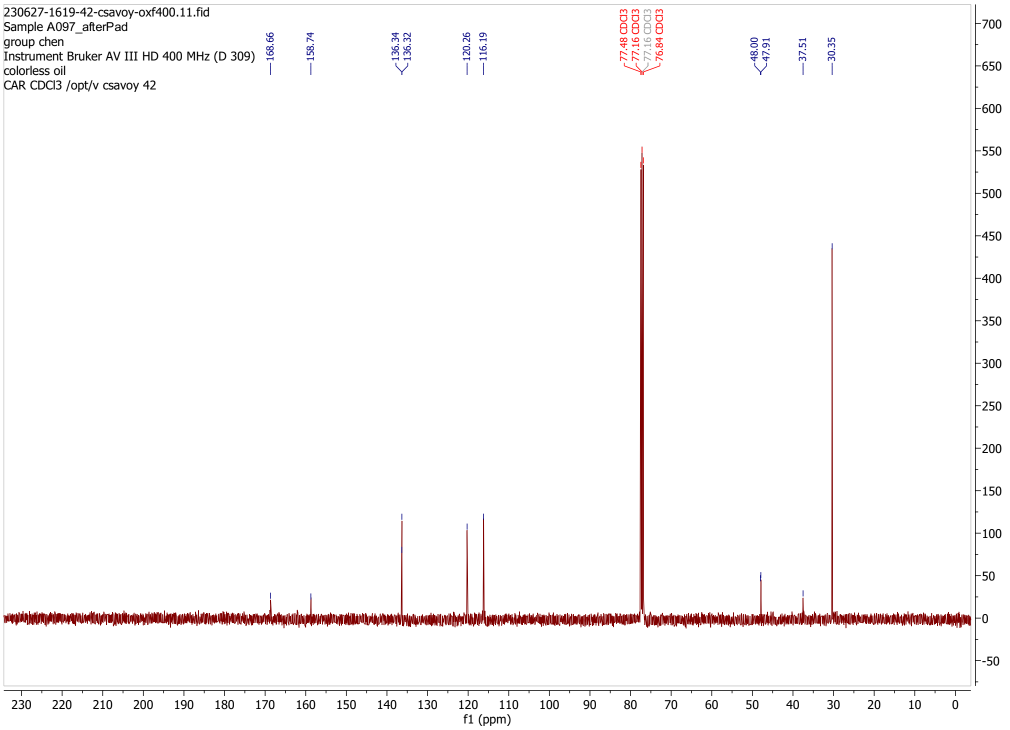

^{13}C-NMR of **t7a** in $CDCl_3$

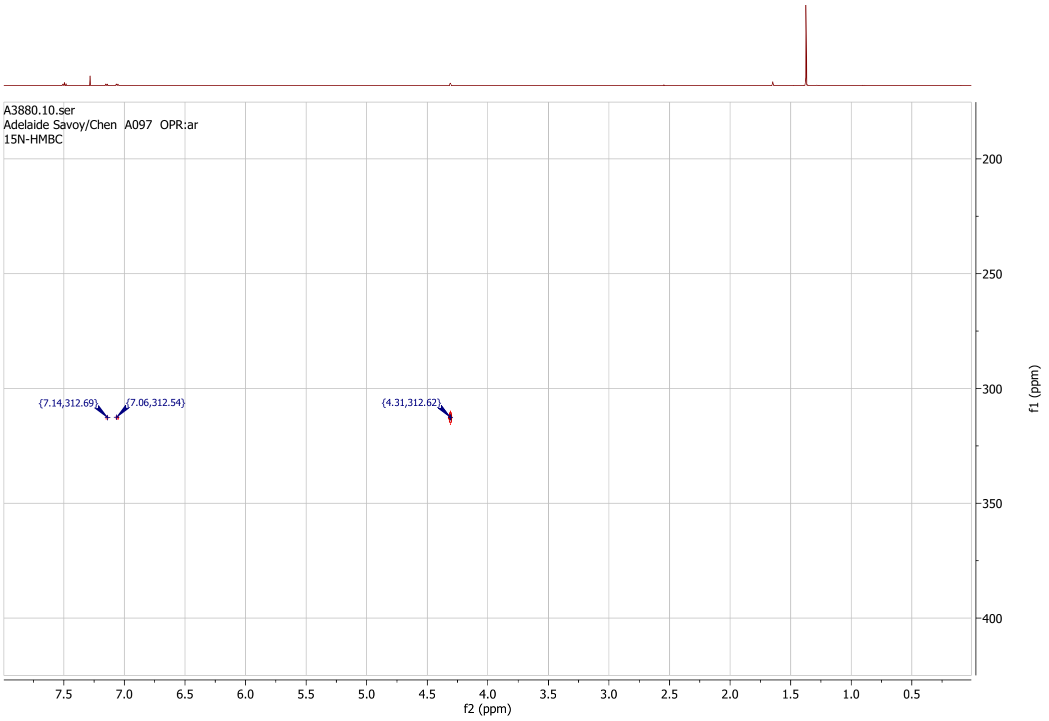

^{1}H-^{15}N-HMBC of **t7a** in $CDCl_3$

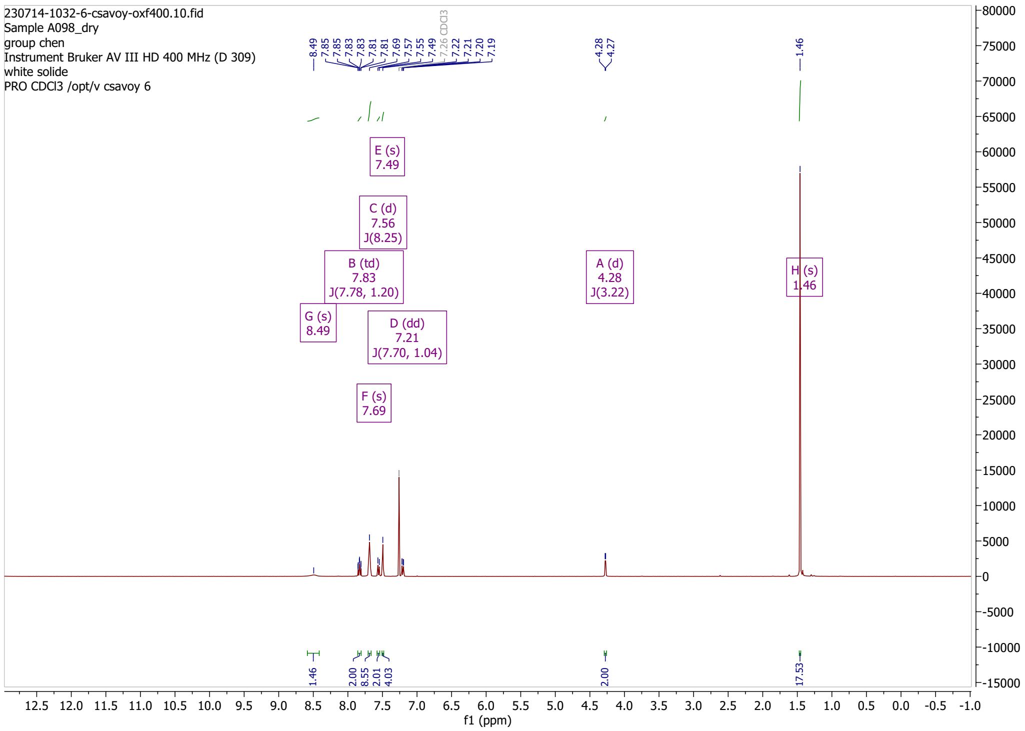

^{1}H-NMR of **t7b** in $CDCl_3$

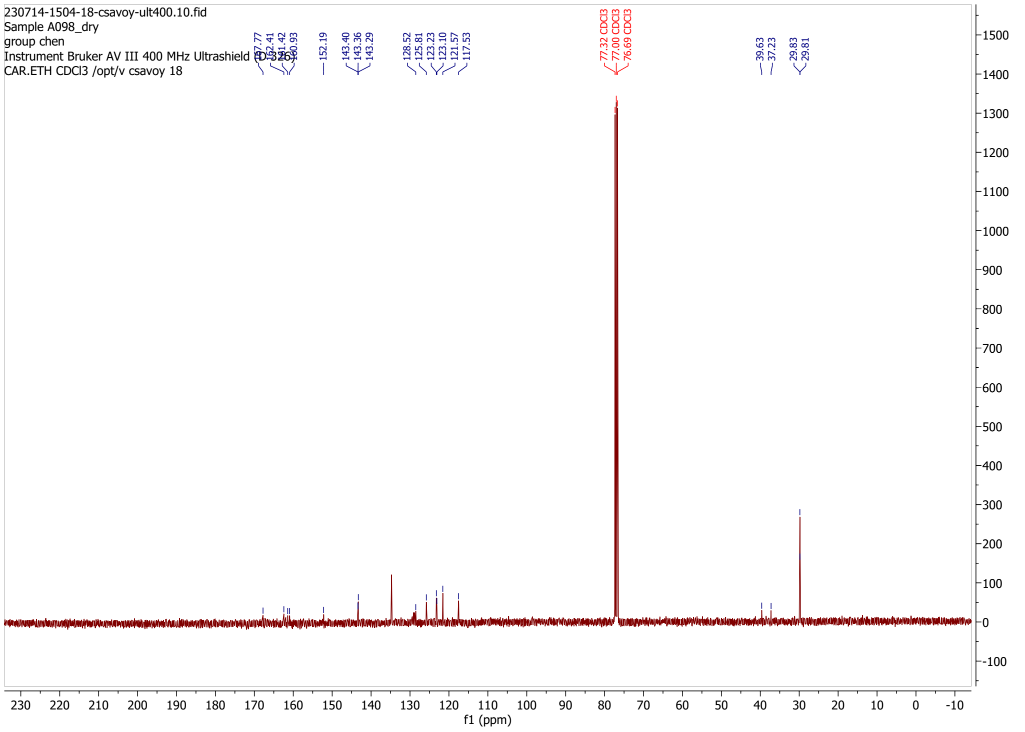

^{13}C-NMR of **t7b** in $CDCl_3$

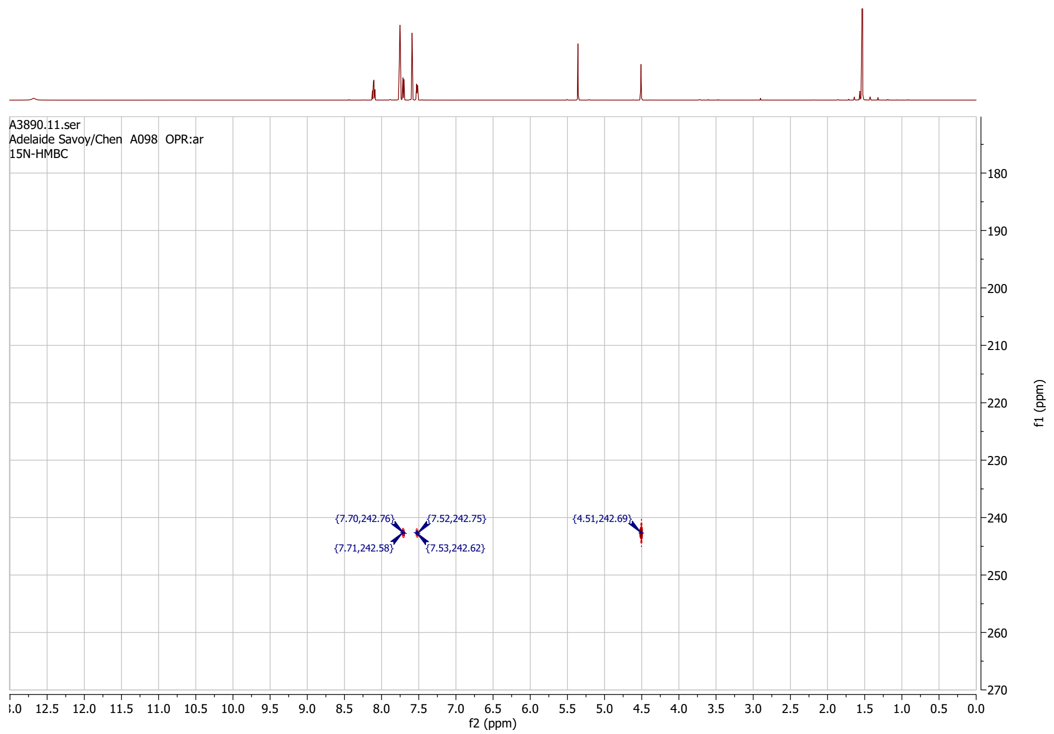

^{1}H-^{15}N-HMBC of **t7b** in CD_2Cl_2

3.5 VT-NMR

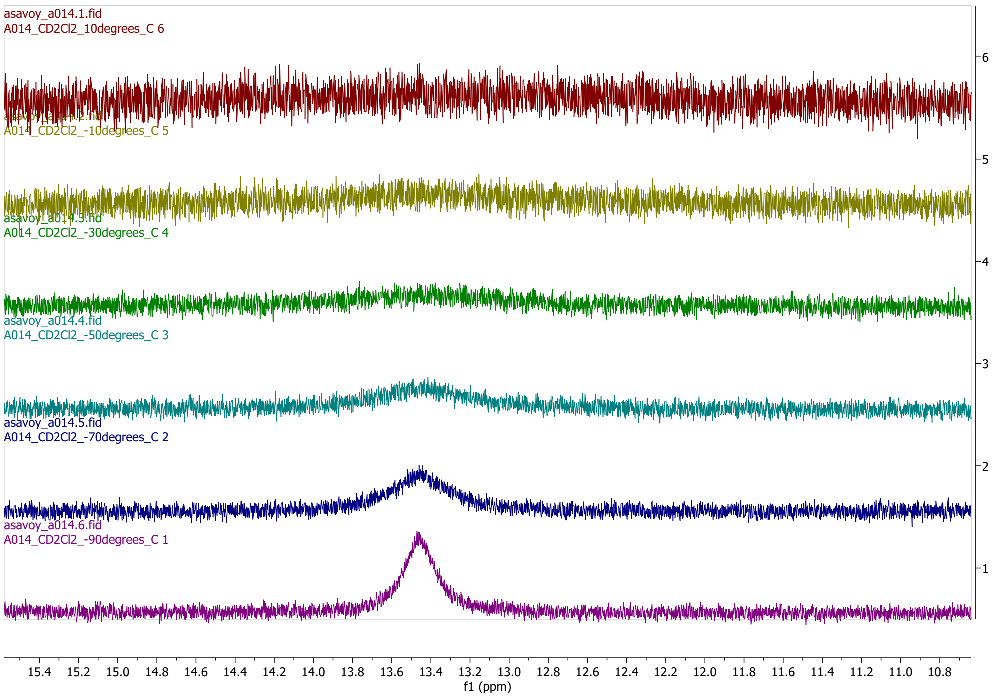

^{1}H-NMR of **1b** in CD_2Cl_2

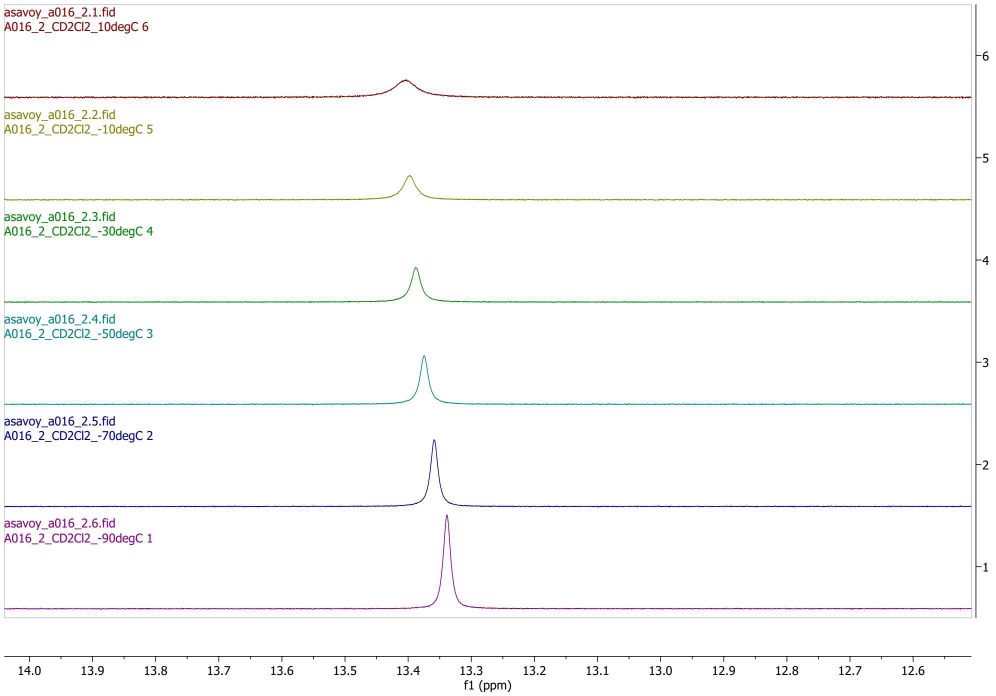

^{1}H-NMR of **2b** in CD_2Cl_2

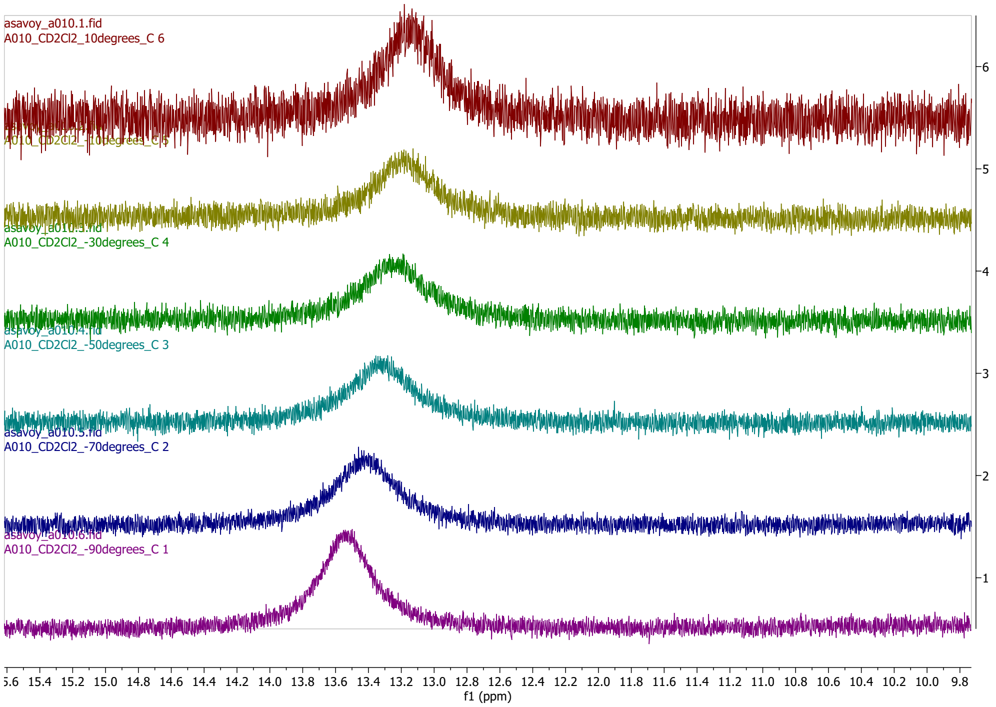

^{1}H-NMR of **3b** in CD_2Cl_2

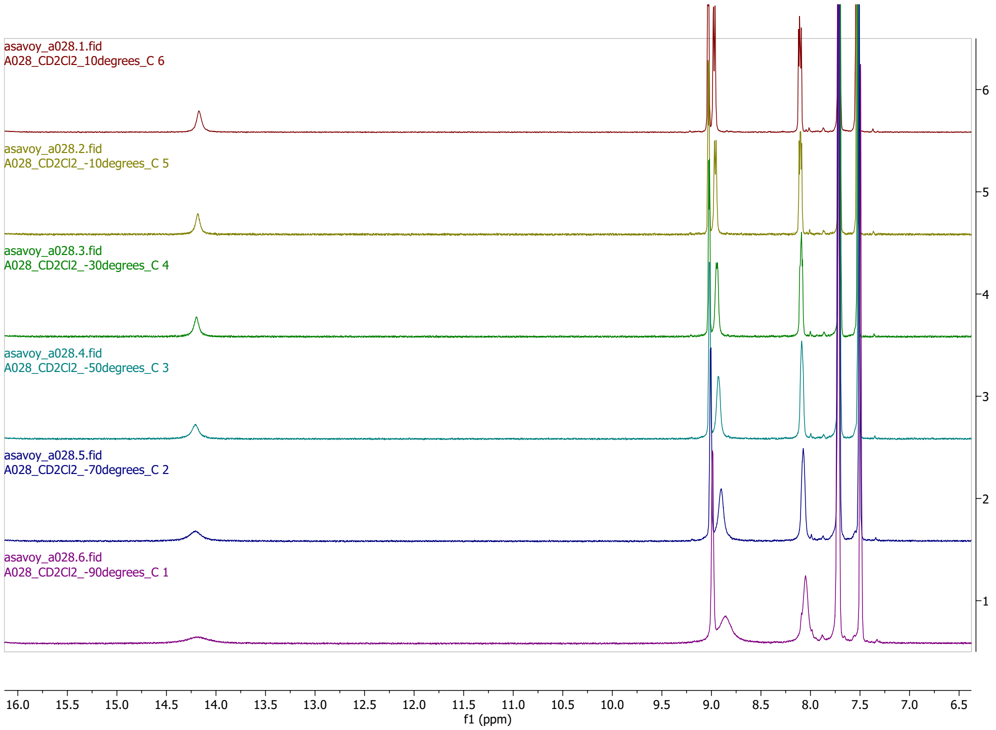

^{1}H-NMR of **4b** in CD_2Cl_2

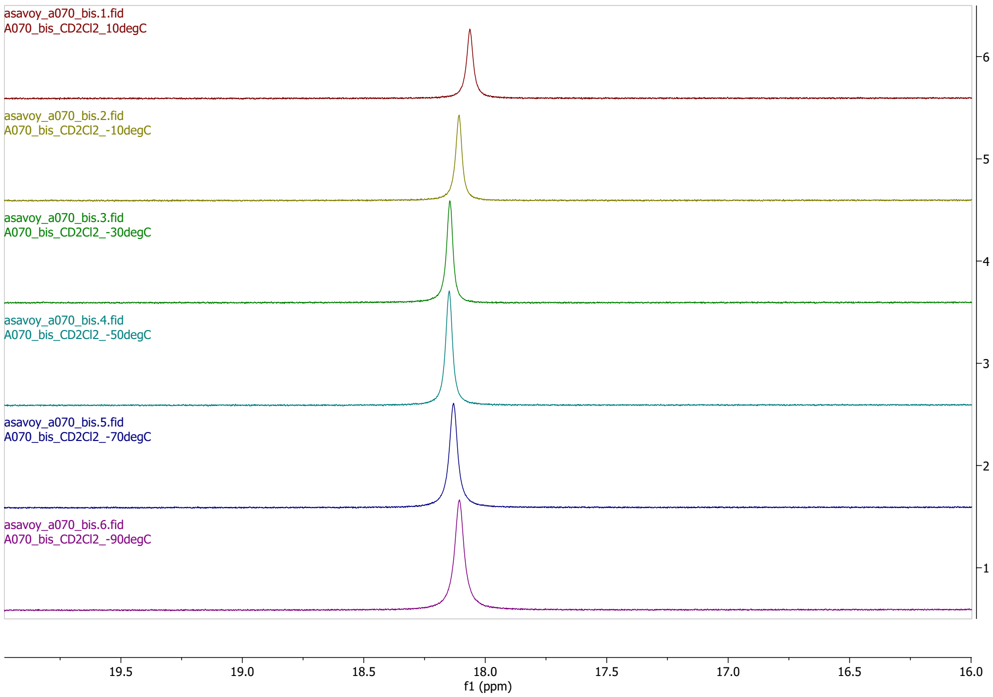

^{1}H-NMR of **5b** in CD_2Cl_2

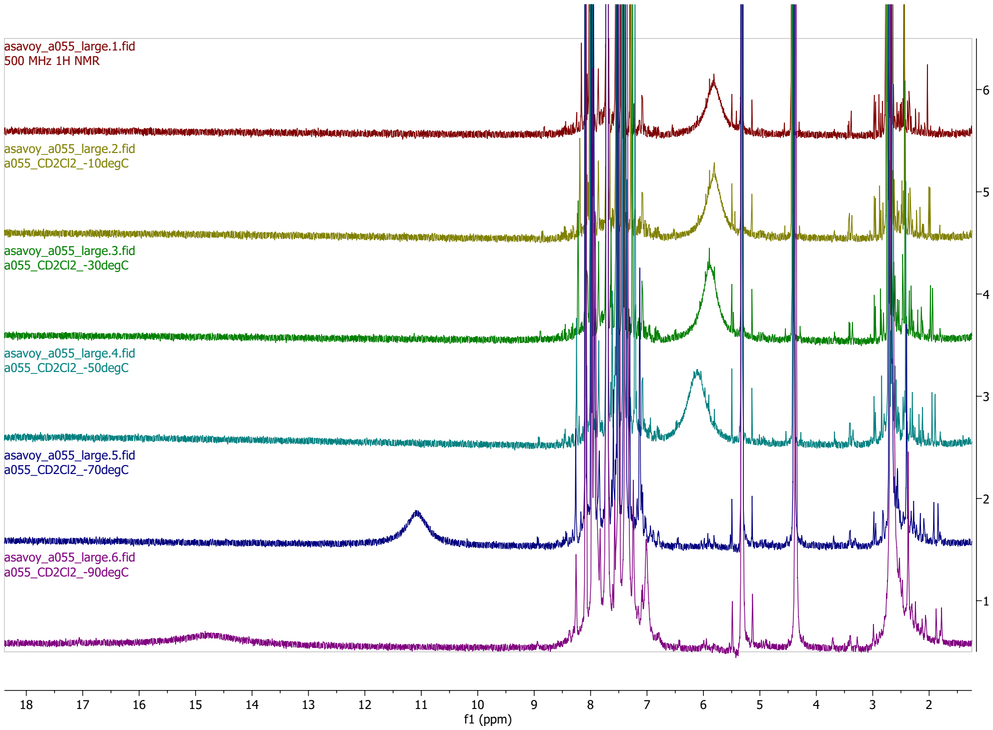

^{1}H-NMR of **6b** in CD_2Cl_2

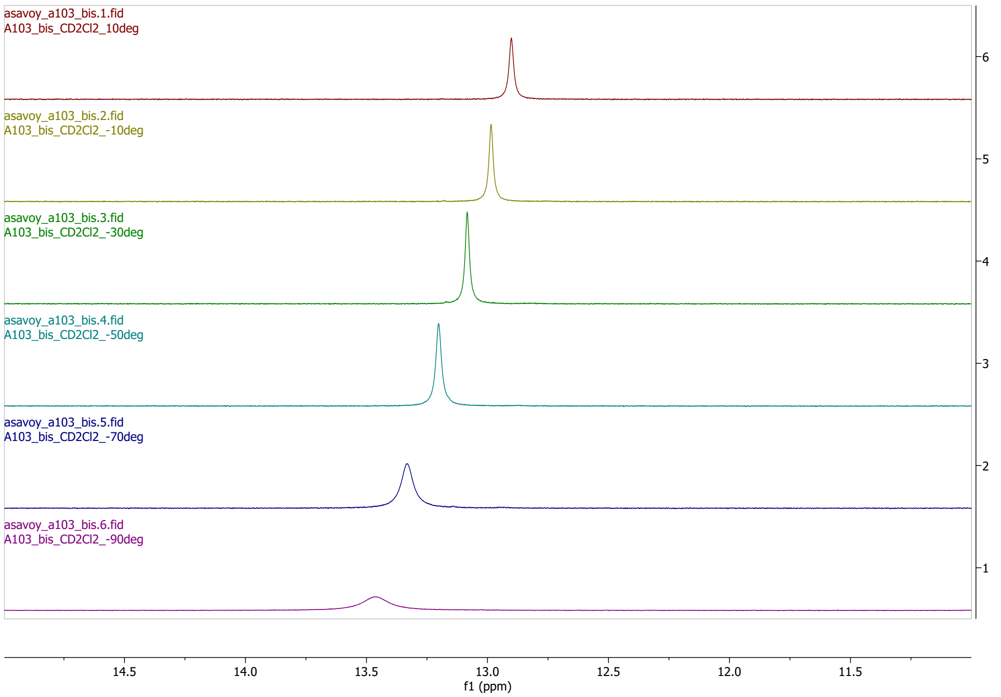

^{1}H-NMR of **7b** in CD_2Cl_2

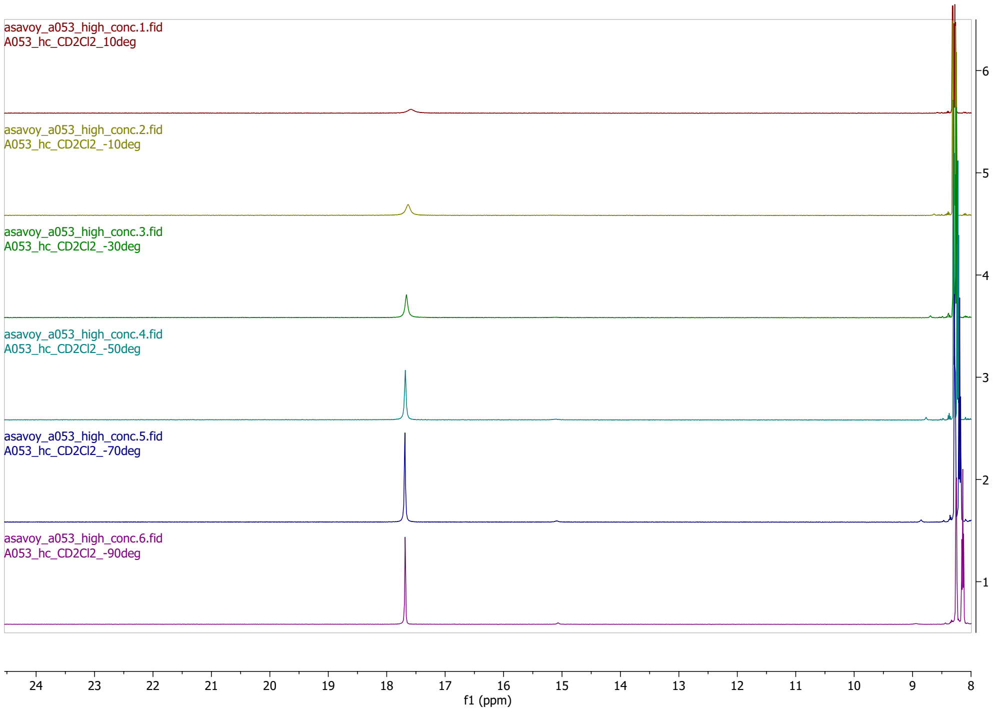

^{1}H-NMR of **8b** in CD_2Cl_2

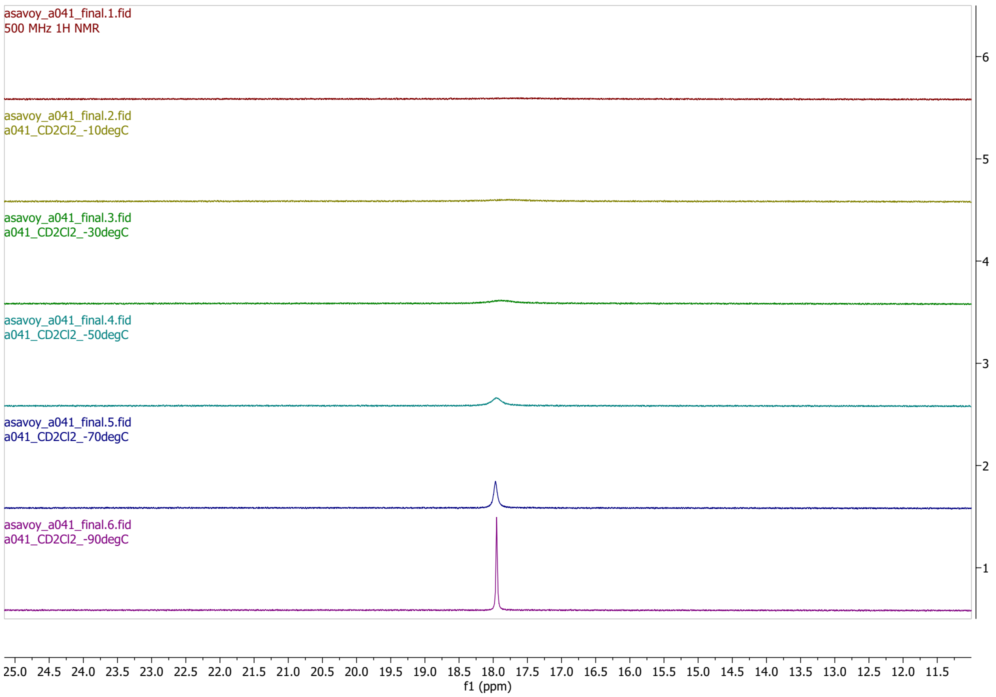

^{1}H-NMR of **9b** in CD_2Cl_2

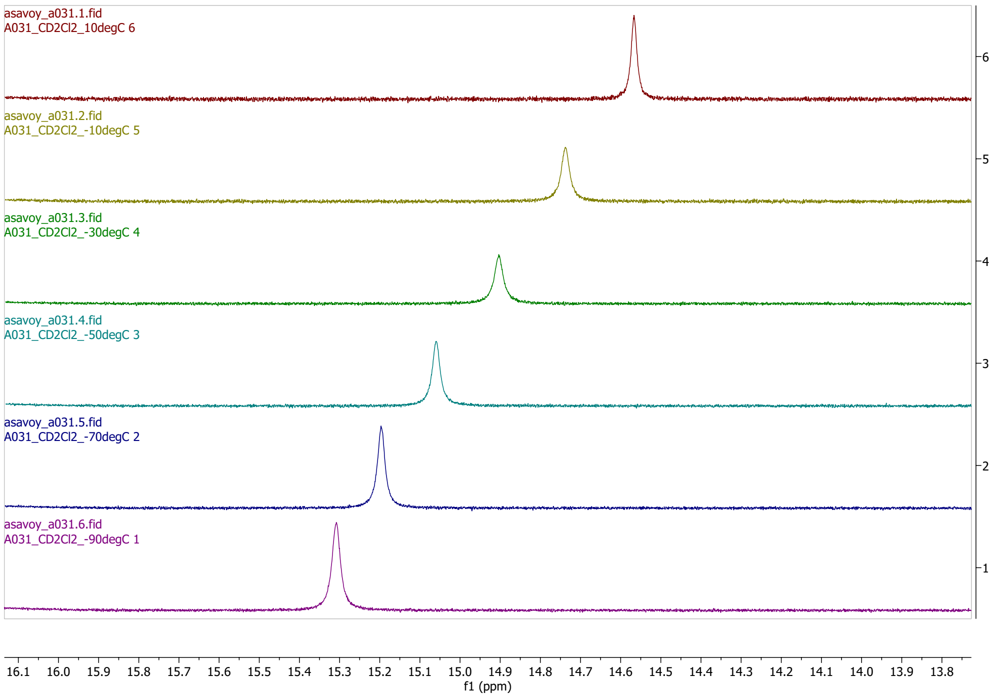

^{1}H-NMR of **10b** in CD_2Cl_2

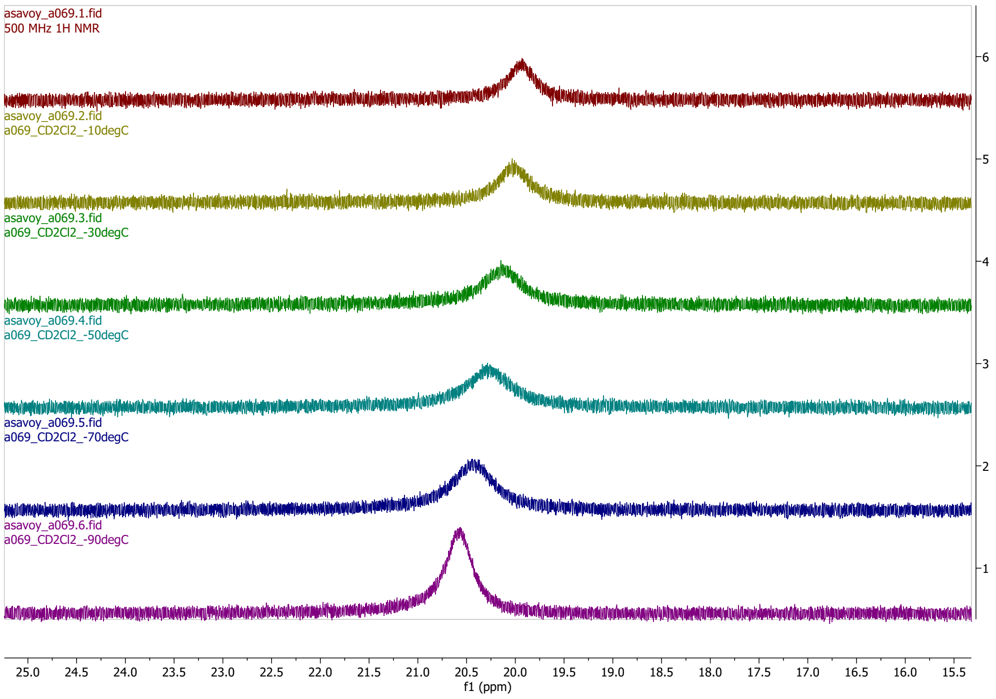

^{1}H-NMR of **11b** in CD_2Cl_2

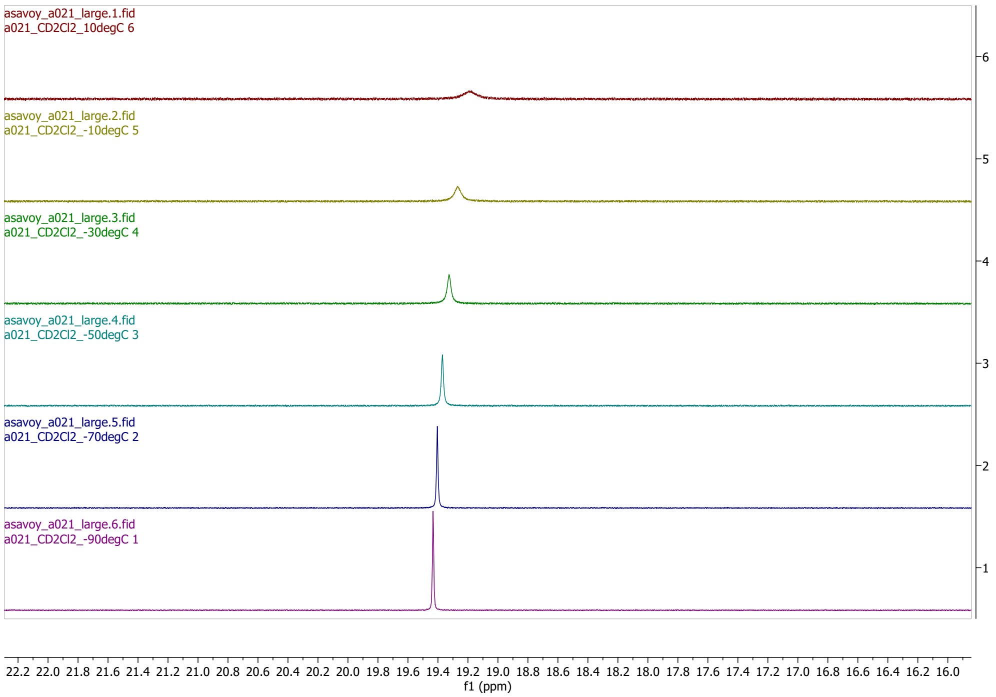

^{1}H-NMR of **12b** in CD_2Cl_2

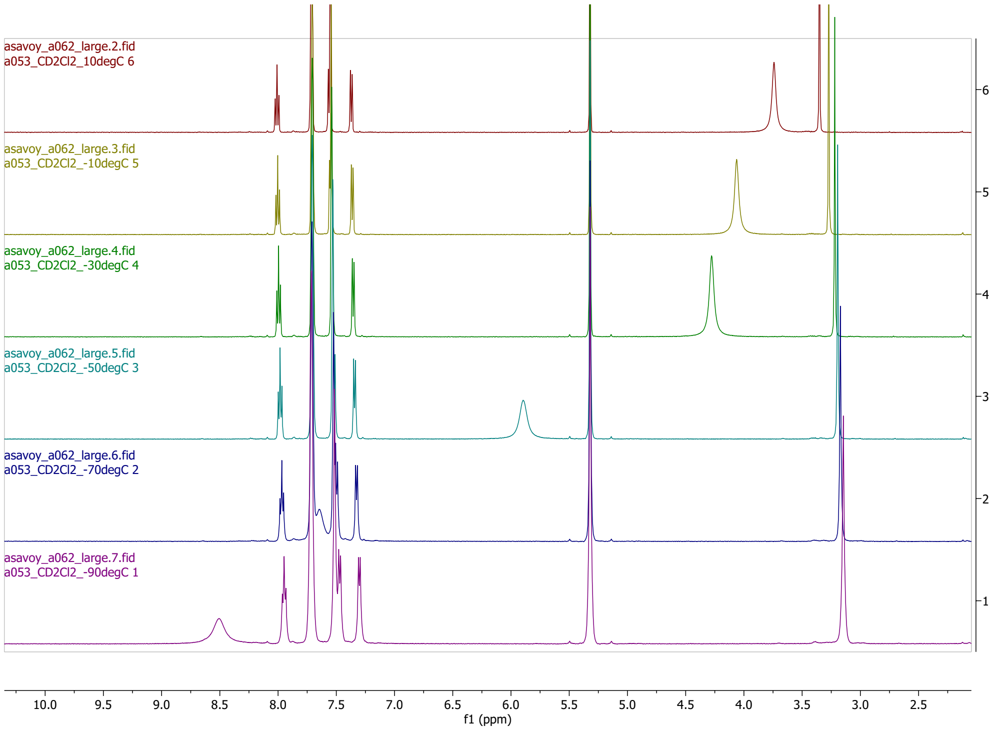

^{1}H-NMR of **13b** in CD_2Cl_2

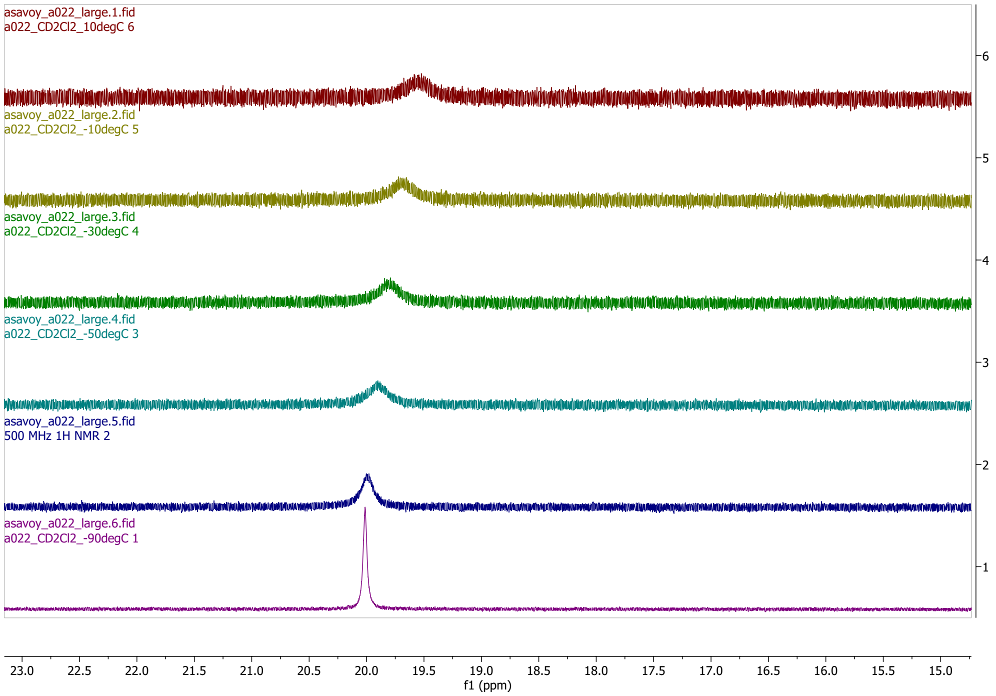

^{1}H-NMR of **14b** in CD_2Cl_2

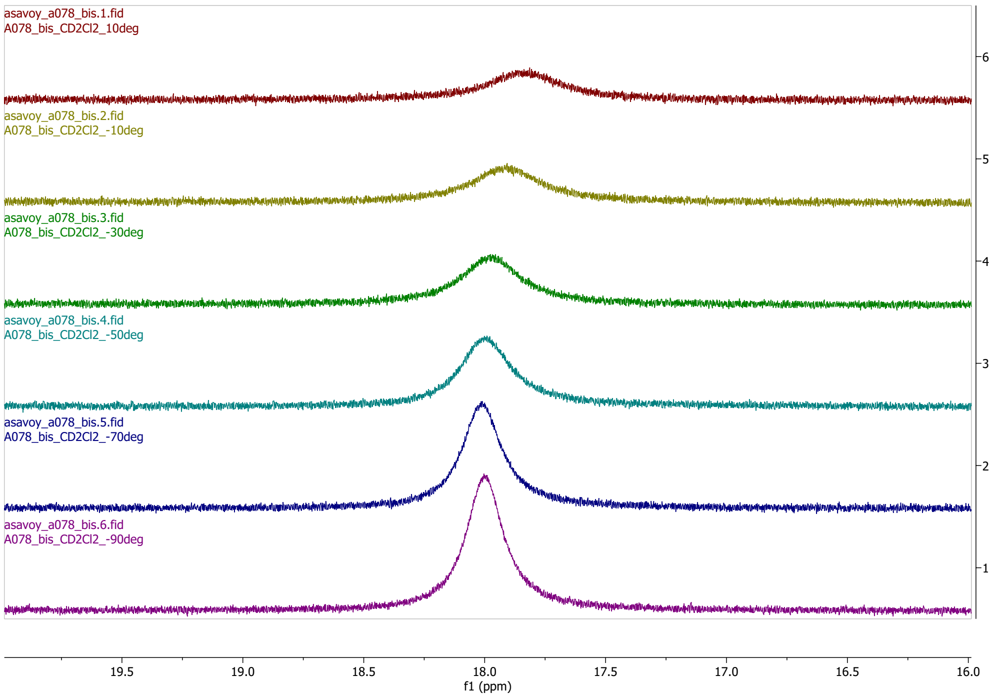

^{1}H-NMR of **t5b** in CD_2Cl_2

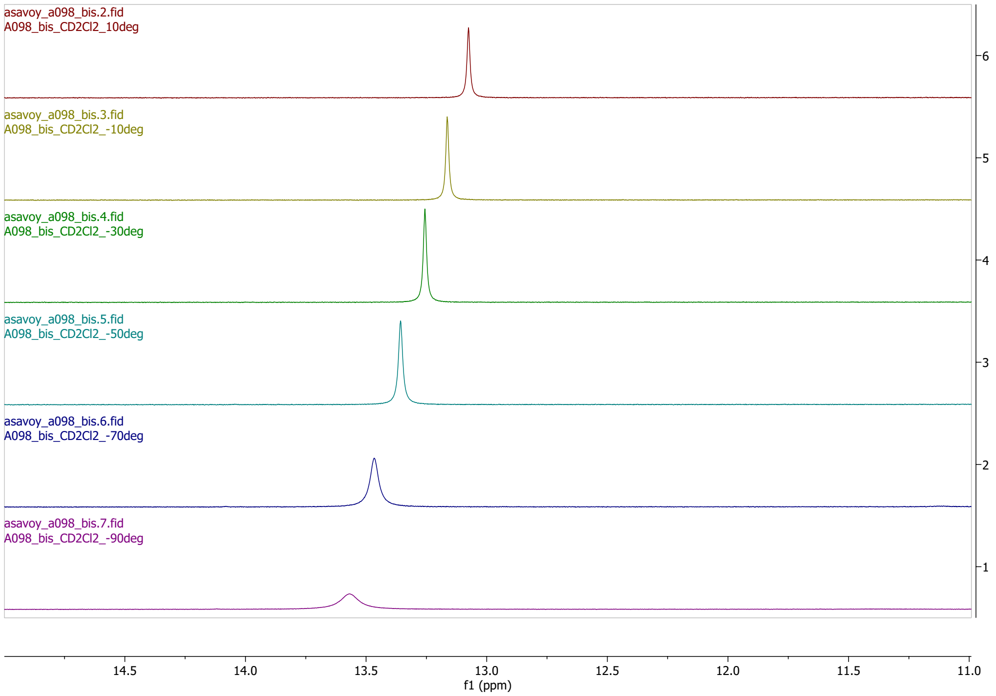

^{1}H-NMR of **t7b** in CD_2Cl_2

XRD

Compound 1b

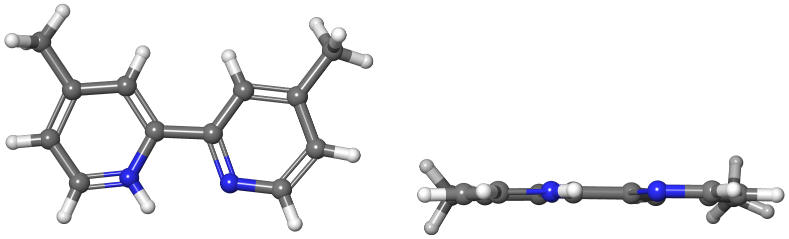

(a) front (b) side

Figure 4.1: XRD structure of compound **1b**

Identification code	c041219_2_1
Chemical formula	$C_{44}H_{25}BF_{24}N_2$
Formula weight	1048.47
Crystal system, space group	triclinic, P-1
Temperature (K)	100.0(1)
$a,\ b,\ c$ (Å)	9.34770(10), 15.6602(2), 16.1982(2)
α, β, γ (°)	73.8830(10), 76.1990(10), 75.6590(10)
V (Å^3)	2169.99(5)
Z	2
ρ_{calc} (g cm^{-3})	1.605
μ (mm^{-1})	1.503
F(000)	1048.0
Crystal size (mm^3)	0.149 × 0.056 × 0.037
Radiation type	CuKα (λ = 1.54184)
2Θ range for data collection (°)	5.776 to 159.598
Index ranges	$-11 \leq h \leq 11$, $-19 \leq k \leq 19$, $-20 \leq l \leq 20$
Reflections collected	60128
Independent reflections	9225 [R_{int} = 0.0519, R_{sigma} = 0.0296]
Data/restraints/parameters	9225/358/679
Goodness-of-fit on F^2	1.085
Final R indexes [I ≥ 2σ (I)]	R_1 = 0.0585, wR_2 = 0.1650
Final R indexes [all data]	R_1 = 0.0690, wR_2 = 0.1742
Largest diff. peak/hole (e Å^{-3})	1.19/-0.44
Deposition Number CCDC	2352861

Table 4.1: Crystal data and structure refinement for **1b**. Measured on a XtaLAB Synergy, Dualflex, Pilatus 300K diffractometer. Using Olex2[10], the structure was solved with the SHELXT[11] structure solution program using Intrinsic Phasing and refined with the SHELXL[12] refinement package using Least Squares minimisation.

Compound 2b

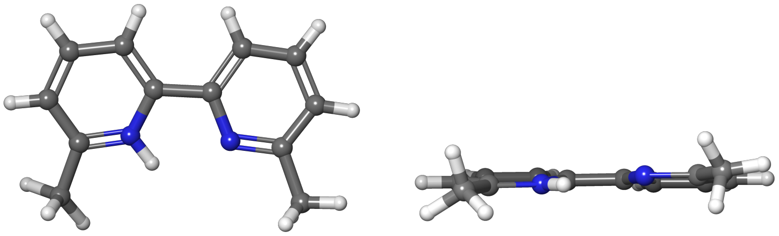

(a) front (b) side

Figure 4.2: XRD structure of compound **2b**

Identification code	c030620_1_1
Chemical formula	$C_{45}H_{26}BCl_3F_{24}N_2$
Formula weight	1167.84
Crystal system, space group	triclinic, P-1
Temperature (K)	100.0(1)
a, b, c (Å)	13.3366(15), 14.1573(15), 15.5099(15)
α, β, γ (°)	66.246(3), 89.877(4), 64.036(3)
V (Å^3)	2355.2(4)
Z	2
ρ_{calc} (g cm^{-3})	1.647
μ (mm^{-1})	0.327
F(000)	1164.0
Crystal size (mm^3)	0.17 × 0.15 × 0.04
Radiation type	MoKα (λ = 0.71073)
2Θ range for data collection (°)	2.936 to 55.254
Index ranges	-17 ≤ h ≤ 17, -18 ≤ k ≤ 18, -20 ≤ l ≤20
Reflections collected	35730
Independent reflections	10885 [R_{int} = 0.0569, R_{sigma} = 0.0717]
Data/restraints/parameters	10885/545/808
Goodness-of-fit on F^2	0.973
Final R indexes [I ≥ 2σ (I)]	R_1 = 0.0469, wR_2 = 0.1032
Final R indexes [all data]	R_1 = 0.0929, wR_2 = 0.1198
Largest diff. peak/hole (e Å^{-3})	0.39/-0.50
Deposition Number CCDC	2352862

Table 4.2: Crystal data and structure refinement for **2b**+ $CHCl_3$. Measured on a XtaLAB Synergy, Dualflex, Pilatus 300K diffractometer. Using Olex2[10], the structure was solved with the SHELXT[11] structure solution program using Intrinsic Phasing and refined with the SHELXL[12] refinement package using Least Squares minimisation.

Compound 3b

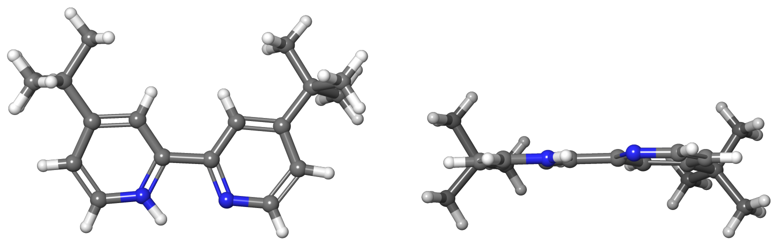

(a) front (b) side

Figure 4.3: XRD structure of compound **3b**

Identification code	c031219_2_2
Chemical formula	$C_{50}H_{37}BF_{24}N_2$
Formula weight	1132.62
Crystal system, space group	triclinic, P-1
Temperature (K)	100.0(1)
a, b, c (Å)	12.98390(10), 13.14960(10), 16.88170(10)
α, β, γ (°)	97.5750(10), 110.6430(10), 108.0880(10)
V (Å^3)	2467.80(4)
Z	2
ρ_{calc} (g cm^{-3})	1.524
μ (mm^{-1})	1.366
F(000)	1144.0
Crystal size (mm^3)	0.293 × 0.224 × 0.139
Radiation type	CuKα (λ = 1.54184)
2Θ range for data collection (°)	5.812 to 160.502
Index ranges	$-16 \leq h \leq 14$, $-16 \leq k \leq 16$, $-21 \leq l \leq 21$
Reflections collected	68819
Independent reflections	10503 [R_{int} = 0.0383, R_{sigma} = 0.0209]
Data/restraints/parameters	10503/2008/995
Goodness-of-fit on F^2	1.075
Final R indexes [I ≥ 2σ (I)]	R_1 = 0.0389, wR_2 = 0.0970
Final R indexes [all data]	R_1 = 0.0463, wR_2 = 0.1037
Largest diff. peak/hole (e Å^{-3})	0.33/-0.28
Deposition Number CCDC	2352860

Table 4.3: Crystal data and structure refinement for **3b**. Measured on a XtaLAB Synergy, Dualflex, Pilatus 300K diffractometer. Using Olex2[10], the structure was solved with the SHELXT[11] structure solution program using Intrinsic Phasing and refined with the SHELXL[12] refinement package using Least Squares minimisation.

Compound 4b

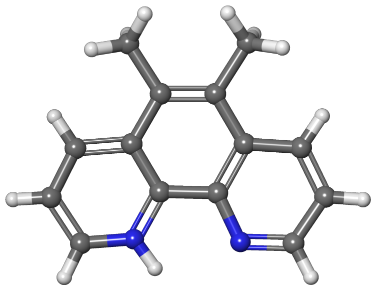

(a) front

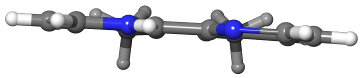

(b) side

Figure 4.4: XRD structure of compound **4b**

Identification code	c080620_3_2
Chemical formula	$C_{46}H_{25}BF_{24}N_2$
Formula weight	1072.49
Crystal system, space group	triclinic, P-1
Temperature (K)	100.0(1)
$a,\ b,\ c$ (Å)	12.3378(2), 13.3787(2), 13.4037(2)
α, β, γ (°)	92.0660(10), 104.8350(10), 90.4690(10)
V (Å^3)	2136.98(6)
Z	2
ρ_{calc} (g cm^{-3})	1.667
μ (mm^{-1})	1.543
F(000)	1072.0
Crystal size (mm^3)	0.327 × 0.279 × 0.092
Radiation type	CuKα (λ = 1.54184)
2Θ range for data collection (°)	6.828 to 146.442
Index ranges	$-15 \leq h \leq 14$, $-15 \leq k \leq 16$, $-16 \leq l \leq 16$
Reflections collected	56609
Independent reflections	8190 [R_{int} = 0.0442, R_{sigma} = 0.0242]
Data/restraints/parameters	8190/860/784
Goodness-of-fit on F^2	1.035
Final R indexes [$I \geq 2\sigma$ (I)]	R_1 = 0.0341, wR_2 = 0.0858
Final R indexes [all data]	R_1 = 0.0385, wR_2 = 0.0884
Largest diff. peak/hole (e Å^{-3})	0.25/-0.29
Deposition Number CCDC	2352865

Table 4.4: Crystal data and structure refinement for **4b**.Measured on a XtaLAB Synergy, Dualflex, Pilatus 200K diffractometer. Using Olex2[10], the structure was solved with the SHELXT[11] structure solution program using Intrinsic Phasing and refined with the SHELXL[12] refinement package using Least Squares minimisation.

Compound 5b

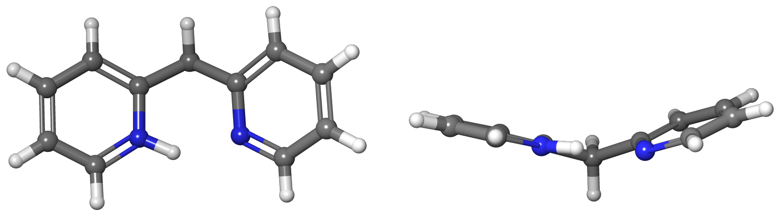

(a) front (b) side

Figure 4.5: XRD structure of compound **5b**

Identification code	c231122_1_1
Chemical formula	$C_{43}H_{23}BF_{24}N_2$
Formula weight	1034.44
Crystal system, space group	monoclinic, $P2_1/c$
Temperature (K)	100.0(1)
a, b, c (Å)	22.5581(2), 13.06430(10), 16.51850(10)
α, β, γ (°)	90, 90.2900(10), 90
V (Å^3)	4868.03(6)
Z	4
ρ_{calc} (g cm^{-3})	1.411
μ (mm^{-1})	1.333
F(000)	2064.0
Crystal size (mm^3)	0.304 × 0.223 × 0.187
Radiation type	CuKα (λ = 1.54184)
2Θ range for data collection (°)	7.82 to 159.404
Index ranges	$-28 \leq h \leq 28$, $-16 \leq k \leq 16$, $-21 \leq l \leq 19$
Reflections collected	72349
Independent reflections	10464 [R_{int} = 0.0462, R_{sigma} = 0.0294]
Data/restraints/parameters	10464/844/770
Goodness-of-fit on F^2	1.040
Final R indexes [$I \geq 2\sigma$ (I)]	R_1 = 0.0479, wR_2 = 0.1216
Final R indexes [all data]	R_1 = 0.0528, wR_2 = 0.1250
Largest diff. peak/hole (e Å^{-3})	0.40/-0.31
Deposition Number CCDC	2352874

Table 4.5: Crystal data and structure refinement for **5b**. Measured on a XtaLAB Synergy, Dualflex, Pilatus 200K diffractometer. Using Olex2[10], the structure was solved with the SHELXT[11] structure solution program using Intrinsic Phasing and refined with the SHELXL[12] refinement package using Least Squares minimisation.

Compound 6b

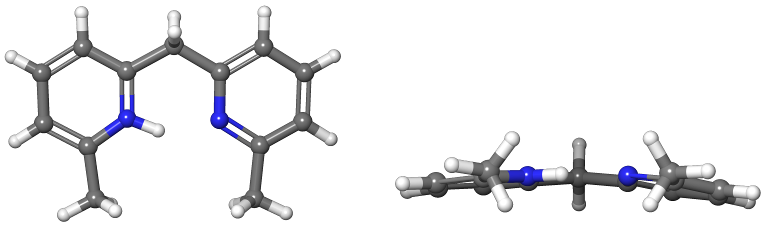

(a) front (b) side

Figure 4.6: XRD structure of compound **6b**

Identification code	c280222_1_1
Chemical formula	$C_{45}H_{27}BF_{24}N_2$
Formula weight	1061.49
Crystal system, space group	triclinic, P-1
Temperature (K)	100.0(1)
$a,\ b,\ c$ (Å)	10.1946(3), 10.3681(2), 21.4146(4)
α, β, γ (°)	78.922(2), 86.887(2), 83.404(2)
V (Å^3)	2205.41(9)
Z	2
ρ_{calc} (g cm^{-3})	1.600
μ (mm^{-1})	1.487
F(000)	1064.0
Crystal size (mm^3)	0.244 × 0.08 × 0.051
Radiation type	CuKα (λ = 1.54184)
2Θ range for data collection (°)	4.206 to 161.398
Index ranges	$-12 \leq h \leq 10$, $-13 \leq k \leq 13$, $-26 \leq l \leq 27$
Reflections collected	34397
Independent reflections	9327 [R_{int} = 0.0567, R_{sigma} = 0.0466]
Data/restraints/parameters	9327/1688/995
Goodness-of-fit on F^2	1.064
Final R indexes [I ≥ 2σ (I)]	R_1 = 0.0480, wR_2 = 0.1285
Final R indexes [all data]	R_1 = 0.0646, wR_2 = 0.1388
Largest diff. peak/hole (e Å^{-3})	0.45/-0.35
Deposition Number CCDC	2352870

Table 4.6: Crystal data and structure refinement for **6b**. Measured on a XtaLAB Synergy, Dualflex, Pilatus 300K diffractometer. Using Olex2[10], the structure was solved with the SHELXT[11] structure solution program using Intrinsic Phasing and refined with the SHELXL[12] refinement package using Least Squares minimisation.

Compound 7b

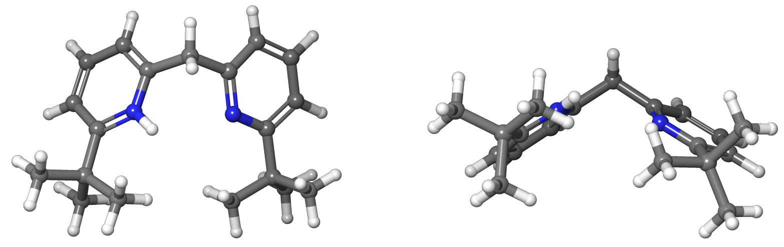

(a) front (b) side

Figure 4.7: XRD structure of compound **7b**

Identification code	c040620_1_2
Chemical formula	$C_{51}H_{39}BF_{24}N_2$
Formula weight	1146.65
Crystal system, space group	triclinic, P-1
Temperature (K)	100.0(1)
a, b, c (Å)	12.9518(7), 13.0314(7), 17.2798(10)
α, β, γ (°)	97.8190(10), 107.3230(10), 111.3320(10)
V (Å^3)	2493.6(2)
Z	2
ρ_{calc} (g cm^{-3})	1.527
μ (mm^{-1})	0.152
F(000)	1160.0
Crystal size (mm^3)	0.28 × 0.2 × 0.17
Radiation type	MoKα (λ = 0.71073)
2Θ range for data collection (°)	3.49 to 61.122
Index ranges	$-18 \leq h \leq 18$, $-18 \leq k \leq 18$, $-24 \leq l \leq 24$
Reflections collected	59674
Independent reflections	15264 [R_{int} = 0.0264, R_{sigma} = 0.0239]
Data/restraints/parameters	15264/447/804
Goodness-of-fit on F^2	1.035
Final R indexes [I ≥ 2σ (I)]	R_1 = 0.0451, wR_2 = 0.1145
Final R indexes [all data]	R_1 = 0.0598, wR_2 = 0.1238
Largest diff. peak/hole (e Å^{-3})	0.62/-0.34
Deposition Number CCDC	2352857

Table 4.7: Crystal data and structure refinement for **7b**. Measured on a Bruker APEX-II Duo (Mo) diffractometer. Using Olex2[10], the structure was solved with the SHELXT[11] structure solution program using Intrinsic Phasing and refined with the SHELXL[12] refinement package using Least Squares minimisation.

Compound 8b

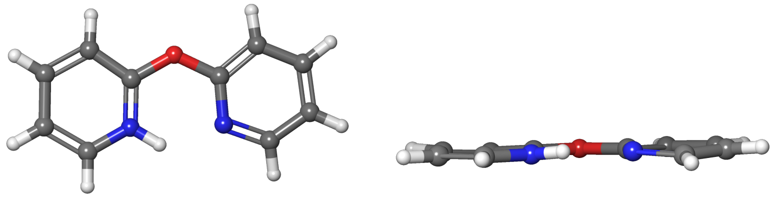

(a) front (b) side

Figure 4.8: XRD structure of compound **8b**

Identification code	c250322_1_1
Chemical formula	$C_{42}H_{21}BF_{24}N_2O$
Formula weight	1036.42
Crystal system, space group	triclinic, P-1
Temperature (K)	100.0(1)
a, b, c (Å)	9.76650(10), 12.6456(2), 18.0040(2)
α, β, γ (°)	106.5840(10), 93.7140(10), 98.8950(10)
V (Å^3)	2091.45(5)
Z	2
ρ_{calc} (g cm^{-3})	1.646
μ (mm^{-1})	1.571
F(000)	1032.0
Crystal size (mm^3)	0.284 × 0.164 × 0.043
Radiation type	CuKα (λ = 1.54184)
2Θ range for data collection (°)	5.156 to 159.544
Index ranges	$-12 \leq h \leq 12$, $-15 \leq k \leq 16$, $-22 \leq l \leq 22$
Reflections collected	57108
Independent reflections	8889 [R_{int} = 0.0456, R_{sigma} = 0.0250]
Data/restraints/parameters	8889/1595/992
Goodness-of-fit on F^2	1.083
Final R indexes [I ≥ 2σ (I)]	R_1 = 0.0421, wR_2 = 0.1096
Final R indexes [all data]	R_1 = 0.0488, wR_2 = 0.1141
Largest diff. peak/hole (e Å^{-3})	0.45/-0.39
Deposition Number CCDC	2352869

Table 4.8: Crystal data and structure refinement for **8b**. Measured on a XtaLAB Synergy, Dualflex, Pilatus 300K diffractometer. Using Olex2[10], the structure was solved with the SHELXT[11] structure solution program using Intrinsic Phasing and refined with the SHELXL[12] refinement package using Least Squares minimisation.

Compound 9b

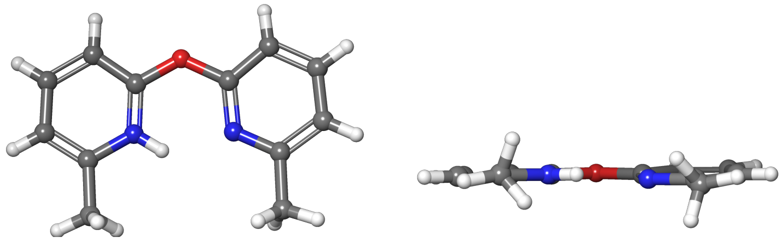

(a) front (b) side

Figure 4.9: XRD structure of compound **9b**

Identification code	c041120_1_1
Chemical formula	$C_{44}H_{25}BF_{24}N_2O$
Formula weight	1064.47
Crystal system, space group	triclinic, P-1
Temperature (K)	100.0(1)
$a,\ b,\ c$ (Å)	10.25310(10), 10.30780(10), 21.2778(2)
α, β, γ (°)	80.1710(10), 84.4690(10), 81.6490(10)
V (Å^3)	2186.36(4)
Z	2
ρ_{calc} (g cm^{-3})	1.617
μ (mm^{-1})	1.520
F(000)	1064.0
Crystal size (mm^3)	0.254 $\times$ 0.201 $\times$ 0.108
Radiation type	CuKα (λ = 1.54184)
2Θ range for data collection (°)	4.226 to 160.116
Index ranges	-13 $\leq$ h $\leq$ 12, -13 $\leq$ k $\leq$ 13, -26 $\leq$ l $\leq$27
Reflections collected	57226
Independent reflections	9276 [R_{int} = 0.0423, R_{sigma} = 0.0238]
Data/restraints/parameters	9276/718/870
Goodness-of-fit on F^2	1.054
Final R indexes [I $\geq$ 2σ (I)]	R_1 = 0.0463, wR_2 = 0.1167
Final R indexes [all data]	R_1 = 0.0544, wR_2 = 0.1225
Largest diff. peak/hole (e Å^{-3})	0.39/-0.37
Deposition Number CCDC	2352868

Table 4.9: Crystal data and structure refinement for **9b**. Measured on a XtaLAB Synergy, Dualflex, Pilatus 300K diffractometer. Using Olex2[10], the structure was solved with the SHELXT[11] structure solution program using Intrinsic Phasing and refined with the SHELXL[12] refinement package using Least Squares minimisation.

Compound 10b

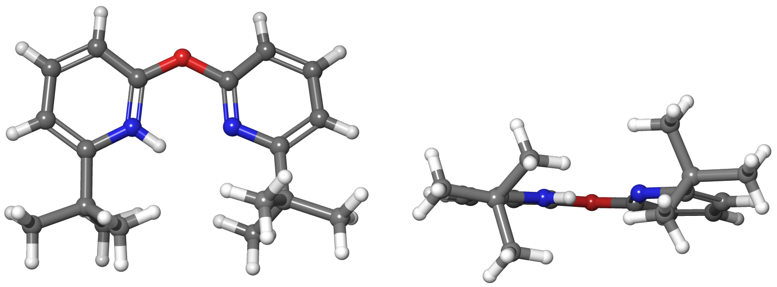

(a) front (b) side

Figure 4.10: XRD structure of compound **10b**. Measured on a Bruker APEX-II Duo (Mo) diffractometer. Using Olex2[10], the structure was solved with the SHELXT[11] structure solution program using Intrinsic Phasing and refined with the SHELXL[12] refinement package using Least Squares minimisation.

Identification code	c010620_1_1
Chemical formula	$C_{50}H_{37}BF_{24}N_2O$
Formula weight	1148.62
Crystal system, space group	monoclinic, Cc
Temperature (K)	100.0(1)
a, b, c (Å)	12.4372(4), 23.2521(7), 18.3176(6)
α, β, γ (°)	90, 107.9160(10), 90
V (Å^3)	2169.99(5)
Z	4
ρ_{calc} (g cm^{-3})	1.514
μ (mm^{-1})	0.152
F(000)	2320.0
Crystal size (mm^3)	0.24 × 0.16 × 0.15
Radiation type	MoKα (λ = 0.71073)
2Θ range for data collection (°)	3.504 to 56.798
Index ranges	-16 ≤ h ≤ 15, -28 ≤ k ≤ 30, -24 ≤ l ≤24
Reflections collected	21661
Independent reflections	11543 [R_{int} = 0.0227, R_{sigma} = 0.0376]
Data/restraints/parameters	11543/361/779
Goodness-of-fit on F^2	1.022
Final R indexes [I ≥ 2σ (I)]	R_1 = 0.0401, wR_2 = 0.0907
Final R indexes [all data]	R_1 = 0.0485, wR_2 = 0.0951
Largest diff. peak/hole (e Å^{-3})	0.40/-0.25
Flack parameter	0.11(17)
Deposition Number CCDC	2352867

Table 4.10: Crystal data and structure refinement for **10b**

Compound 11b

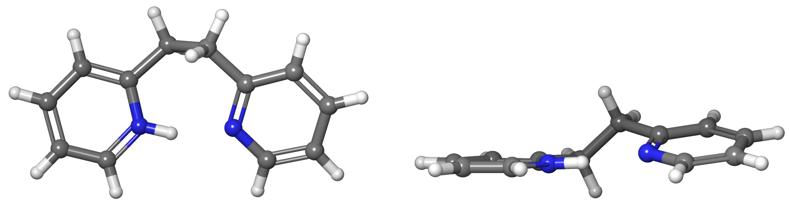

(a) front (b) side

Figure 4.11: XRD structure of compound **11b**

Identification code	c200123_1_2
Chemical formula	$C_{44}H_{25}BF_{24}N_2$
Formula weight	1048.47
Crystal system, space group	monoclinic, $P2_1/c$
Temperature (K)	100.0(1)
a, b, c (Å)	20.35010(10), 12.77770(10), 16.88380(10)
α, β, γ (°)	90, 103.7410(10), 90
V (Å^3)	4264.60(5)
Z	4
ρ_{calc} (g cm^{-3})	1.633
μ (mm^{-1})	1.530
F(000)	2096.0
Crystal size (mm^3)	0.321 × 0.249 × 0.188
Radiation type	CuKα (λ = 1.54184)
2Θ range for data collection (°)	0.321 × 0.249 × 0.188
Index ranges	-25 ≤ h ≤ 25, -16 ≤ k ≤ 15, -21 ≤ l ≤21
Reflections collected	149387
Independent reflections	9238 [R_{int} = 0.0349, R_{sigma} = 0.0118]
Data/restraints/parameters	9238/467/708
Goodness-of-fit on F^2	1.025
Final R indexes [I ≥ 2σ (I)]	R_1 = 0.0390, wR_2 = 0.0922
Final R indexes [all data]	R_1 = 0.0407, wR_2 = 0.0934
Largest diff. peak/hole (e Å^{-3})	0.47/-0.35
Deposition Number CCDC	2352873

Table 4.11: Crystal data and structure refinement for **11b**. Measured on a XtaLAB Synergy, Dualflex, Pilatus 200K diffractometer. Using Olex2[10], the structure was solved with the SHELXT[11] structure solution program using Intrinsic Phasing and refined with the SHELXL[12] refinement package using Least Squares minimisation.

Compound 12b

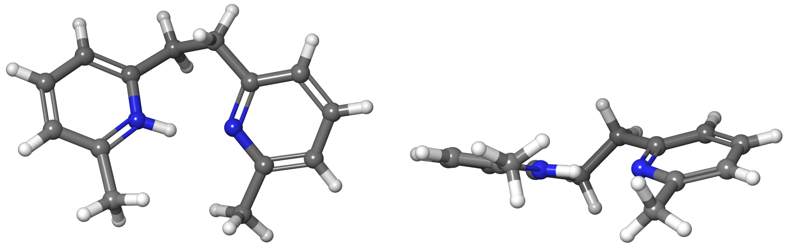

(a) front (b) side

Figure 4.12: XRD structure of compound **12b**

Identification code	c020620_1_1
Chemical formula	$C_{46}H_{29}BF_{24}N_2$
Formula weight	1076.47
Crystal system, space group	monoclinic, $P2_1/c$
Temperature (K)	100.0(1)
a, b, c (Å)	12.7494(14), 21.745(2), 16.4749(17)
α, β, γ (°)	90, 93.000(3), 90
V (Å^3)	4561.1(8)
Z	4
ρ_{calc} (g cm^{-3})	1.568
μ (mm^{-1})	0.160
F(000)	2160.0
Crystal size (mm^3)	0.23 × 0.19 × 0.08
Radiation type	MoKα (λ = 0.71073)
2Θ range for data collection (°)	3.104 to 56.686
Index ranges	$-17 \leq h \leq 17$, $-28 \leq k \leq 29$, $-21 \leq l \leq 21$
Reflections collected	85974
Independent reflections	11357 [R_{int} = 0.0370, R_{sigma} = 0.0210]
Data/restraints/parameters	11357/575/847
Goodness-of-fit on F^2	1.023
Final R indexes [I ≥ 2σ (I)]	R_1 = 0.0388, wR_2 = 0.0910
Final R indexes [all data]	R_1 = 0.0543, wR_2 = 0.0988
Largest diff. peak/hole (e Å^{-3})	0.35/-0.27
Deposition Number CCDC	2352863

Table 4.12: Crystal data and structure refinement for **12b**. Measured on a Bruker APEX-II Duo (Mo) diffractometer. Using Olex2[10], the structure was solved with the SHELXT[11] structure solution program using Intrinsic Phasing and refined with the SHELXL[12] refinement package using Least Squares minimisation.

Compound 13b

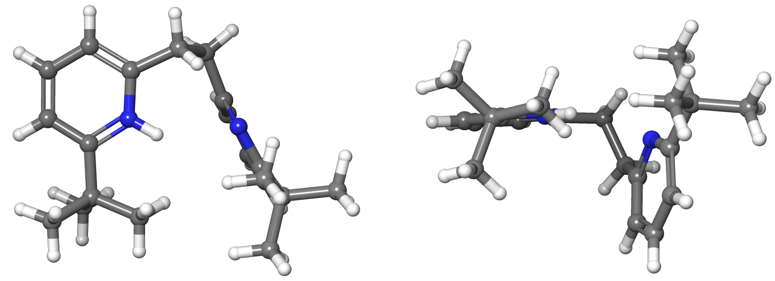

(a) front (b) side

Figure 4.13: XRD structure of compound **13b**

Identification code	c240322_3_1
Chemical formula	$C_{53}H_{43}BCl_2F_{24}N_2$
Formula weight	1245.60
Crystal system, space group	orthorhombic, Pbca
Temperature (K)	100.0(1)
a, b, c (Å)	21.3063(2), 20.9139(2), 24.3031(3)
α, β, γ (°)	90, 90, 90
V (Å^3)	10829.4(2)
Z	8
ρ_{calc} (g cm^{-3})	1.528
μ (mm^{-1})	2.188
F(000)	5040.0
Crystal size (mm^3)	0.28 $\times$ 0.155 $\times$ 0.061
Radiation type	CuKα (λ = 1.54184)
2Θ range for data collection (°)	7.274 to 159.532
Index ranges	-27 $\leq$ h $\leq$ 22, -26 $\leq$ k $\leq$ 26, -30 $\leq$ l $\leq$30
Reflections collected	88264
Independent reflections	11655 [R_{int} = 0.0359, R_{sigma} = 0.0209]
Data/restraints/parameters	11655/970/912
Goodness-of-fit on F^2	1.060
Final R indexes [I $\geq$ 2σ (I)]	R_1 = 0.0537, wR_2 = 0.1352
Final R indexes [all data]	R_1 = 0.0613, wR_2 = 0.1416
Largest diff. peak/hole (e Å^{-3})	0.87/-0.69
Deposition Number CCDC	2352871

Table 4.13: Crystal data and structure refinement for **13b**+CH_2Cl_2. Measured on a XtaLAB Synergy, Dualflex, Pilatus 200K diffractometer. Using Olex2[10], the structure was solved with the SHELXT[11] structure solution program using Intrinsic Phasing and refined with the SHELXL[12] refinement package using Least Squares minimisation.

Compound 14b

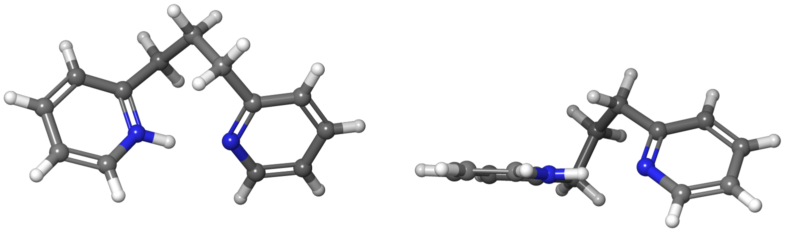

(a) front (b) side

Figure 4.14: XRD structure of compound **14b**

Identification code	c050620_2_1
Chemical formula	$C_{45}H_{27}BF_{24}N_2$
Formula weight	1062.48
Crystal system, space group	monoclinic, $P2_1/c$
Temperature (K)	100.0(1)
a, b, c (Å)	19.5780(15), 13.9630(10), 16.6113(13)
α, β, γ (°)	90, 106.838(2), 90
V (Å^3)	4346.3(6)
Z	4
ρ_{calc} (g cm^{-3})	1.624
μ (mm^{-1})	0.167
F(000)	2128.0
Crystal size (mm^3)	0.24 × 0.16 × 0.1
Radiation type	MoKα (λ = 0.71073)
2Θ range for data collection (°)	3.638 to 56.744
Index ranges	$-26 \leq h \leq 26$, $-18 \leq k \leq 11$, $-22 \leq l \leq 22$
Reflections collected	43270
Independent reflections	10838 [R_{int} = 0.0401, R_{sigma} = 0.0389]
Data/restraints/parameters	10838/388/734
Goodness-of-fit on F^2	1.010
Final R indexes [I ≥ 2σ (I)]	R_1 = 0.0450, wR_2 = 0.0955
Final R indexes [all data]	R_1 = 0.0749, wR_2 = 0.1082
Largest diff. peak/hole (e Å^{-3})	0.50/-0.29
Deposition Number CCDC	2352864

Table 4.14: Crystal data and structure refinement for **14b**. Measured on a Bruker APEX-II Duo (Mo) diffractometer. Using Olex2[10], the structure was solved with the SHELXT[11] structure solution program using Intrinsic Phasing and refined with the SHELXL[12] refinement package using Least Squares minimisation.

Compound B-H-B$^+$

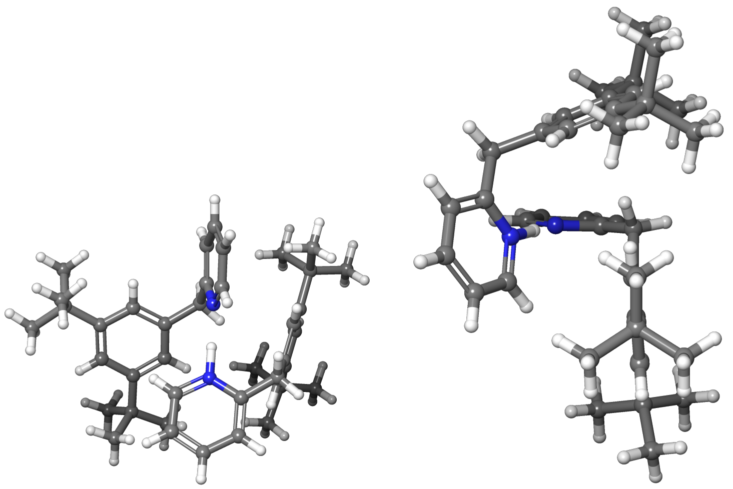

(a) front (b) side

Figure 4.15: XRD structure of compound B-H-N$^+$

Identification code	c280220
Chemical formula	$C_{72}H_{67}BF_{24}N_2$
Formula weight	1427.08
Crystal system, space group	monoclinic, $P2_1/c$
Temperature (K)	100.0(1)
a, b, c (Å)	17.9819(17), 18.4321(18), 22.181(2)
α, β, γ (°)	90, 110.091(2), 90
V (Å^3)	6904.5(12)
Z	4
ρ_{calc} (g cm^{-3})	1.373
μ (mm^{-1})	0.125
F(000)	2936.0
Crystal size (mm^3)	0.559 × 0.541 × 0.525
Radiation type	MoKα (λ = 0.71073)
2Θ range for data collection (°)	2.412 to 62.464
Index ranges	$-25 \leq h \leq 25$, $-26 \leq k \leq 26$, $-31 \leq l \leq 31$
Reflections collected	91951
Independent reflections	20844 [R_{int} = 0.0489, R_{sigma} = 0.0512]
Data/restraints/parameters	20844/286/1087
Goodness-of-fit on F^2	1.048
Final R indexes [I ≥ 2σ (I)]	R_1 = 0.0567, wR_2 = 0.1440
Final R indexes [all data]	R_1 = 0.0843, wR_2 = 0.1596
Largest diff. peak/hole (e Å^{-3})	0.45/-0.41
Deposition Number CCDC	2470444

Table 4.15: Crystal data and structure refinement for dimervova. Measured on a Bruker APEX-II Duo (Mo) diffractometer. Using Olex2[10], the structure was solved with the SHELXT[11] structure solution program using Intrinsic Phasing and refined with the SHELXL[12] refinement package using Least Squares minimisation.

DFT

The visualization of the generated .xyz files, their superposition with the measured XRD structures and the RMSD calculation between the two structure of the heavy atoms (carbon, nitrogen and oxygen) was done using Maestro (2025-2 version). The superimposed structures have been reprepared to ensure consistency. The nitrogen atoms and their neighboring carbons have been used for superposition of the calculated and measured structure using the “superposition “ tool with the “atom pairs” option as implemented.

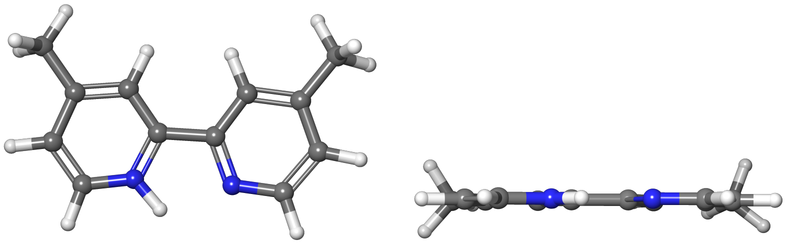

(a) front (b) side

Figure 5.1: DFT(BP86-D3BJ/def2-TZVP) structure of compound **1b**. N-H-N angle: 112°. RMSD (heavy atoms) to the XRD structure: 3.5992

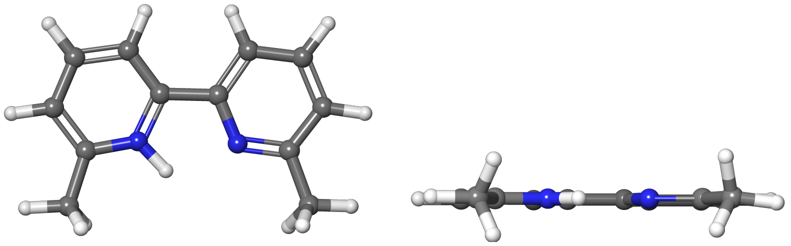

(a) front (b) side

Figure 5.2: DFT(BP86-D3BJ/def2-TZVP) structure of compound **2b**.N-H-N angle: 113°. RMSD (heavy atoms) to the XRD structure: 2.4013

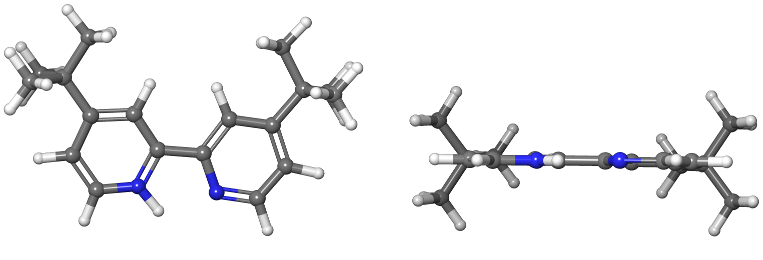

(a) front (b) side

Figure 5.3: DFT(BP86-D3BJ/def2-TZVP) structure of compound **3b**. N-H-N angle: 112°. RMSD (heavy atoms) to the XRD structure: 4.6224

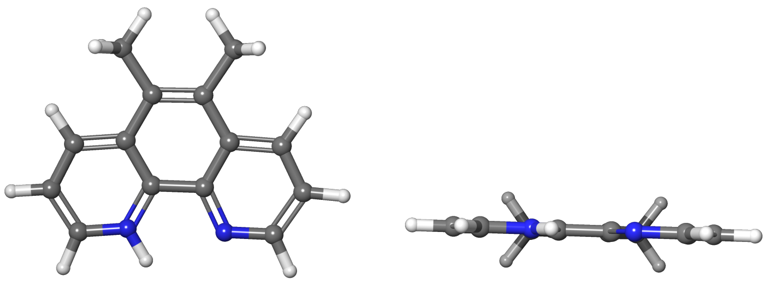

(a) front (b) side

Figure 5.4: DFT(BP86-D3BJ/def2-TZVP) structure of compound **4b**. N-H-N angle: 108°. RMSD (heavy atoms) to the XRD structure: 4.5400

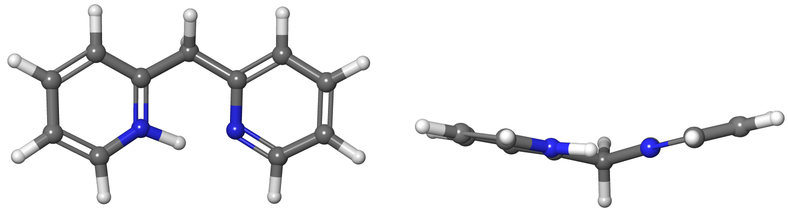

(a) front (b) side

Figure 5.5: DFT(BP86-D3BJ/def2-TZVP) structure of compound **5b**.N-H-N angle: 136°. RMSD (heavy atoms) to the XRD structure: 2.1248

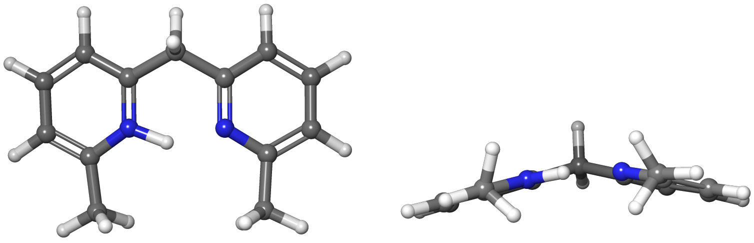

(a) front (b) side

Figure 5.6: DFT(BP86-D3BJ/def2-TZVP) structure of compound **6b**.N-H-N angle: 145°. RMSD (heavy atoms) to the XRD structure: 4.0123

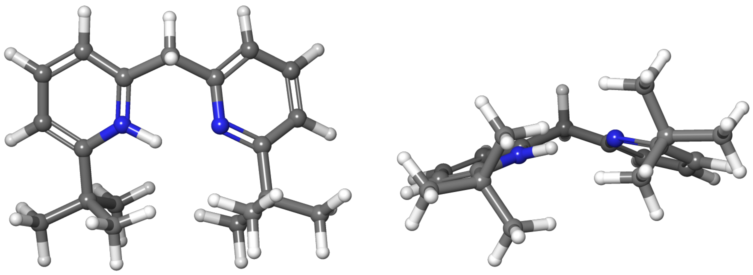

(a) front (b) side

Figure 5.7: DFT(BP86-D3BJ/def2-TZVP) structure of compound **7b**. N-H-N angle: 139°. RMSD (heavy atoms) to the XRD structure: 4.3532

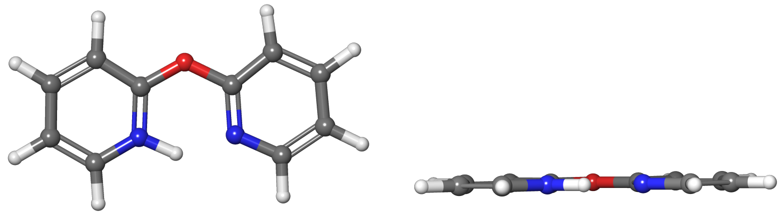

(a) front (b) side

Figure 5.8: DFT(BP86-D3BJ/def2-TZVP) structure of compound **8b**. N-H-N angle: 139°. RMSD (heavy atoms) to the XRD structure: 1.7897

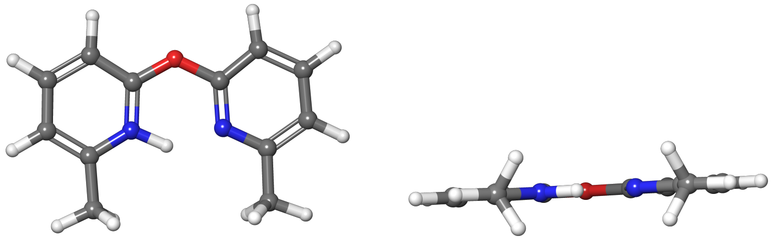

(a) front (b) side

Figure 5.9: DFT(BP86-D3BJ/def2-TZVP) structure of compound **9b**. N-H-N angle: 139°. RMSD (heavy atoms) to the XRD structure: 4.9474

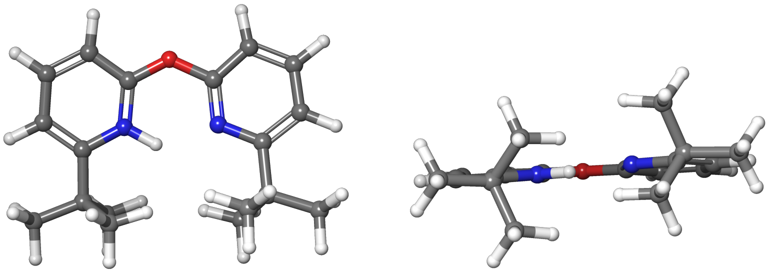

(a) front (b) side

Figure 5.10: DFT(BP86-D3BJ/def2-TZVP) structure of compound **10b**. N-H-N angle: 136°. RMSD (heavy atoms) to the XRD structure: 4.7548

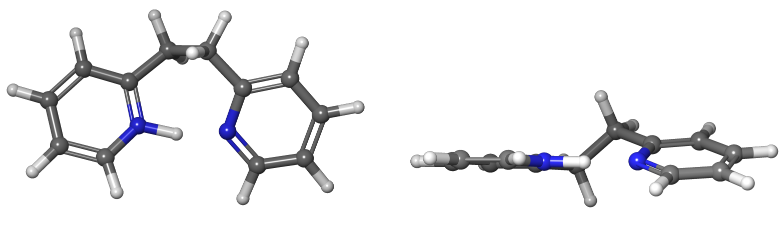

(a) front (b) side

Figure 5.11: DFT(BP86-D3BJ/def2-TZVP) structure of compound **11b**. N-H-N angle: 164°. RMSD (heavy atoms) to the XRD structure: 2.7037

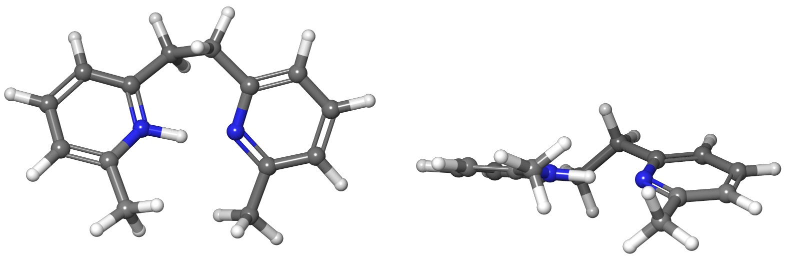

(a) front (b) side

Figure 5.12: DFT(BP86-D3BJ/def2-TZVP) structure of compound **12b**. N-H-N angle: 163°. RMSD (heavy atoms) to the XRD structure: 3.4999

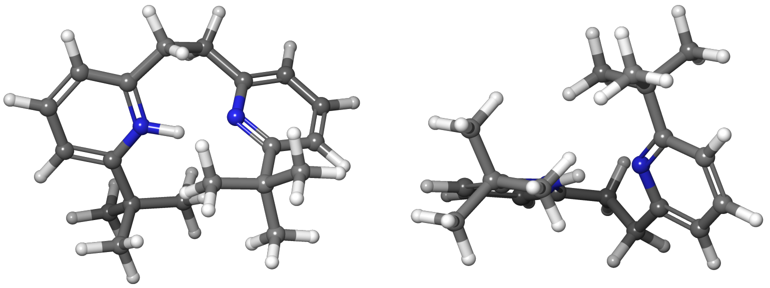

(a) front (b) side

Figure 5.13: DFT(BP86-D3BJ/def2-TZVP) structure of compound **13b**. N-H-N angle: 155°. RMSD (heavy atoms) to the XRD structure: 4.2603

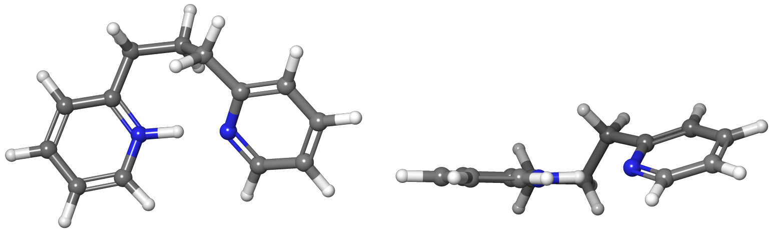

(a) front (b) side

Figure 5.14: DFT(BP86-D3BJ/def2-TZVP) structure of compound **14b**. N-H-N angle: 171°. RMSD (heavy atoms) to the XRD structure: 4.8878

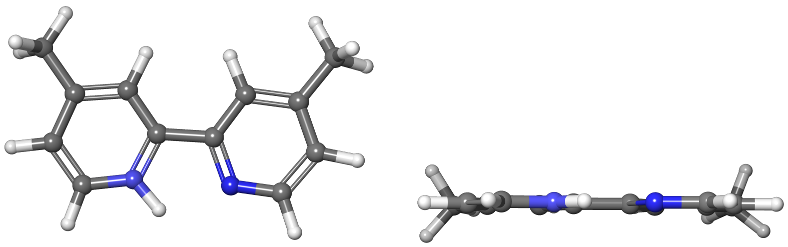

(a) front (b) side

Figure 5.15: DFT(BP86/def2-TZVP) structure of compound **1b**. N-H-N angle: 112°. RMSD (heavy atoms) to the XRD structure: 3.6017

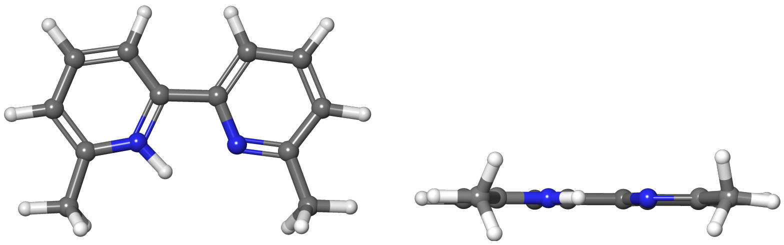

(a) front (b) side

Figure 5.16: DFT(BP86/def2-TZVP) structure of compound **2b**. N-H-N angle: 113°. RMSD (heavy atoms) to the XRD structure: 2.4037

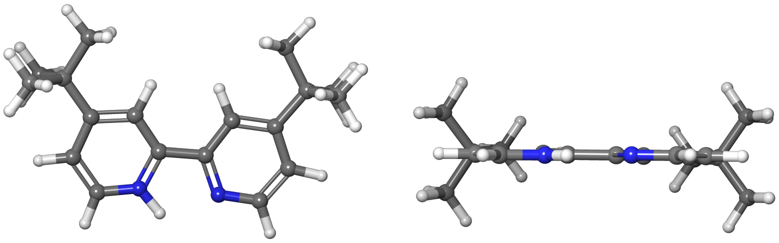

(a) front (b) side

Figure 5.17: DFT(BP86/def2-TZVP) structure of compound **3b**. N-H-N angle: 112°. RMSD (heavy atoms) to the XRD structure: 4.6325

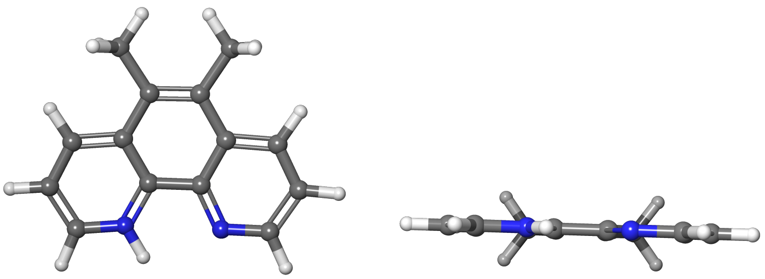

(a) front (b) side

Figure 5.18: DFT(BP86/def2-TZVP) structure of compound **4b**. N-H-N angle: 107°. RMSD (heavy atoms) to the XRD structure: 4.5421

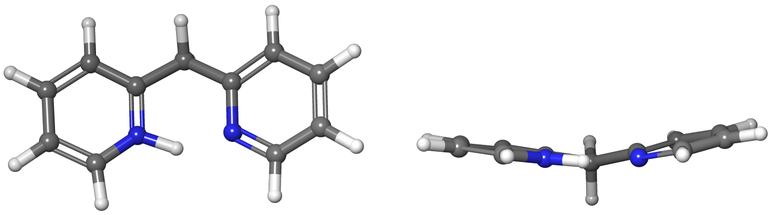

(a) front (b) side

Figure 5.19: DFT(BP86/def2-TZVP) structure of compound **5b**. N-H-N angle: 134°. RMSD (heavy atoms) to the XRD structure: 2.1282

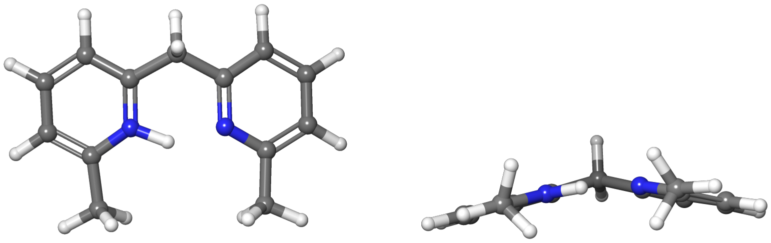

(a) front (b) side

Figure 5.20: DFT(BP86/def2-TZVP) structure of compound **6b**. N-H-N angle: 144°. RMSD (heavy atoms) to the XRD structure: 4.0220

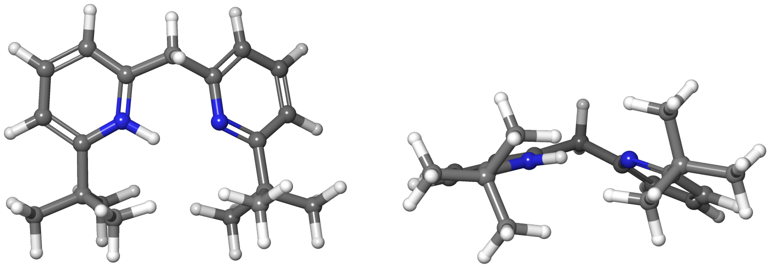

(a) front (b) side

Figure 5.21: DFT(BP86/def2-TZVP) structure of compound **7b**. N-H-N angle: 136°. RMSD (heavy atoms) to the XRD structure: 4.3992

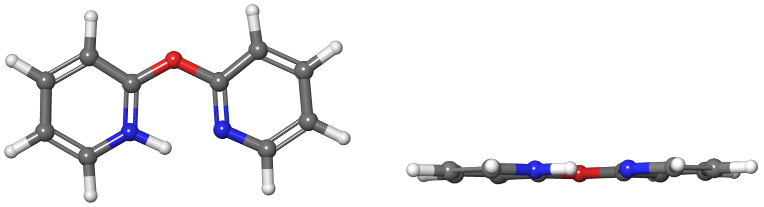

(a) front (b) side

Figure 5.22: DFT(BP86/def2-TZVP) structure of compound **8b**. N-H-N angle: 138°. RMSD (heavy atoms) to the XRD structure: 1.7893

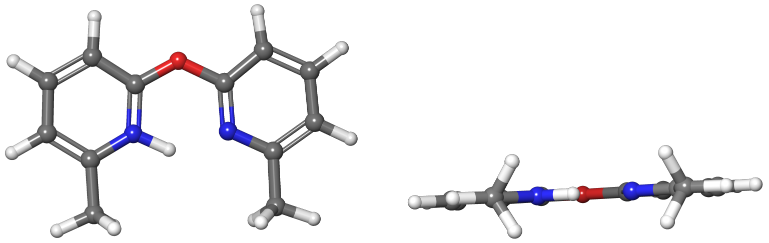

(a) front (b) side

Figure 5.23: DFT(BP86/def2-TZVP) structure of compound **9b**. N-H-N angle: 139°. RMSD (heavy atoms) to the XRD structure: 4.9549

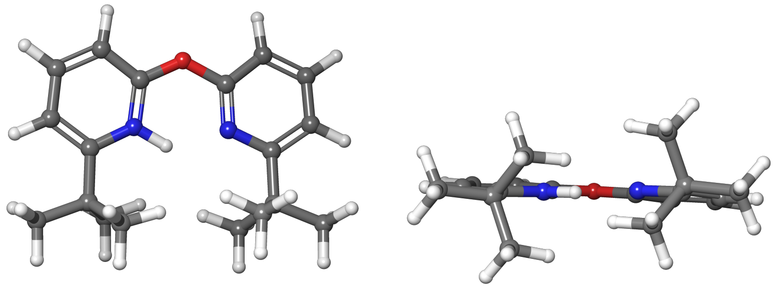

(a) front (b) side

Figure 5.24: DFT(BP86/def2-TZVP) structure of compound **10b**. N-H-N angle: 134°. RMSD (heavy atoms) to the XRD structure: 4.7876

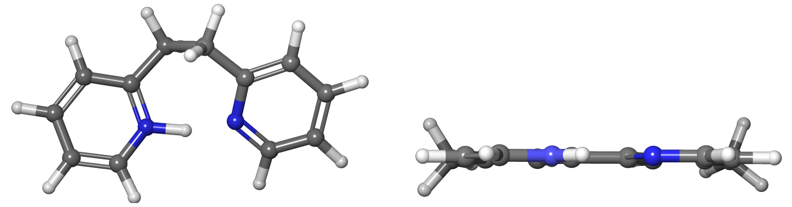

(a) front (b) side

Figure 5.25: DFT(BP86/def2-TZVP) structure of compound **11b**. N-H-N angle: 165°. RMSD (heavy atoms) to the XRD structure: 2.7079

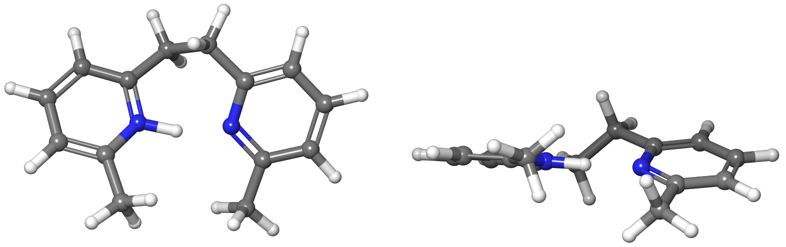

(a) front (b) side

Figure 5.26: DFT(BP86/def2-TZVP) structure of compound **12b**. N-H-N angle: 163°. RMSD (heavy atoms) to the XRD structure: 3.5082

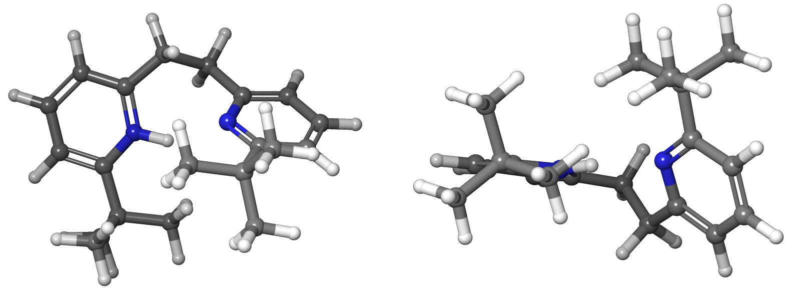

(a) front (b) side

Figure 5.27: DFT(BP86/def2-TZVP) structure of compound **13b**. N-H-N angle: 151°. RMSD (heavy atoms) to the XRD structure: 4.3144

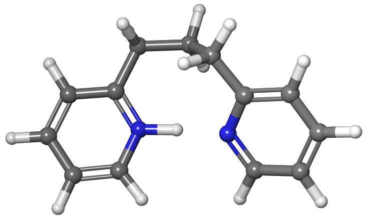

(a) front

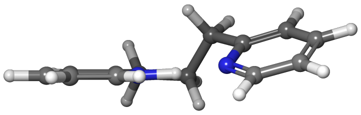

(b) side

Figure 5.28: DFT(BP86/def2-TZVP) structure of compound **14b**. N-H-N angle: 172°. RMSD (heavy atoms) to the XRD structure: 4.9052

^{1}H NMR Calculations and Conformer Analysis

6.1 Computational details

^{1}H NMR chemical shift calculations were carried out as follows: conformer ensembles were first generated using the CREST protocol [13–15]. These were followed by gas-phase geometry optimizations using the PBE-D3(BJ) functional [15, 16] and the def2-TZVP basis set [17] that were reported previously. [18] The resulting structures served as input for further geometry optimizations and ^{1}H NMR shielding tensor calculations with the PBE-D4 functional and the larger def2-TZVPD basis set [17], using the SMD implicit solvation model for dichloromethane (DCM) [19], as implemented in the ORCA quantum chemistry package, version 6.0.1 [17, 20–22].

To improve efficiency, the RI (resolution of identity) approximation was employed for the Coulomb integrals [23]. Tight SCF convergence criteria [24] and increased numerical integration grids (DefGrid2) were used to ensure accuracy [25]. Nuclear magnetic shielding constants were computed using the GIAO (Gauge-Including Atomic Orbitals) method [26], and chemical shifts were referenced to TMS.

A typical input file is given below. More characteristic data are discussed below, while all raw data can be found in the attached ZIP file.

```
! RKS PBE D4 def2-TZVPD def2/J RI TightSCF defgrid2 NoMOPrint Printbasis
    Opt NMR SMD(CH2Cl2)

%geom
  TolE=1e-6
  TolMaxG=1e-4
  TolRMSG=3e-5
  TolMaxD=1e-3
  TolRMSD=6e-4
end

%pal nprocs 16 end
%maxcore 8000

%CPCM
   SMD true
   SMDSOLVENT "CH2Cl2"
end
* xyz 1 1
...
...
*

%eprnmr
  Nuclei = all { shift }
end
```

6.2 Conformer space and Reference compounds

Table 6.1: Overview of the number of conformers considered per compound and their numbering in the previous work[18].

Comp.	# of conf. considered	Numbering in previous work [18]
**1b**	5	
**2b**	5	
**3b**	14	
**4b**	5	**1**
**5b**	1	**4**
**6b**	2	**5**
**7b**	76	**6**
**8b**	1	**7**
**9b**	3	**8**
**10b**	22	**9**
**11b**	1	**10**
**12b**	2	**11**
**13b**	81	**12**
**14b**	3	

* Reported numbers correspond to structures considered unique by the CREST protocol. In this and subsequent chapters, conformer uniqueness is re-evaluated using additional structural and spectroscopic metrics and clustering analyses.

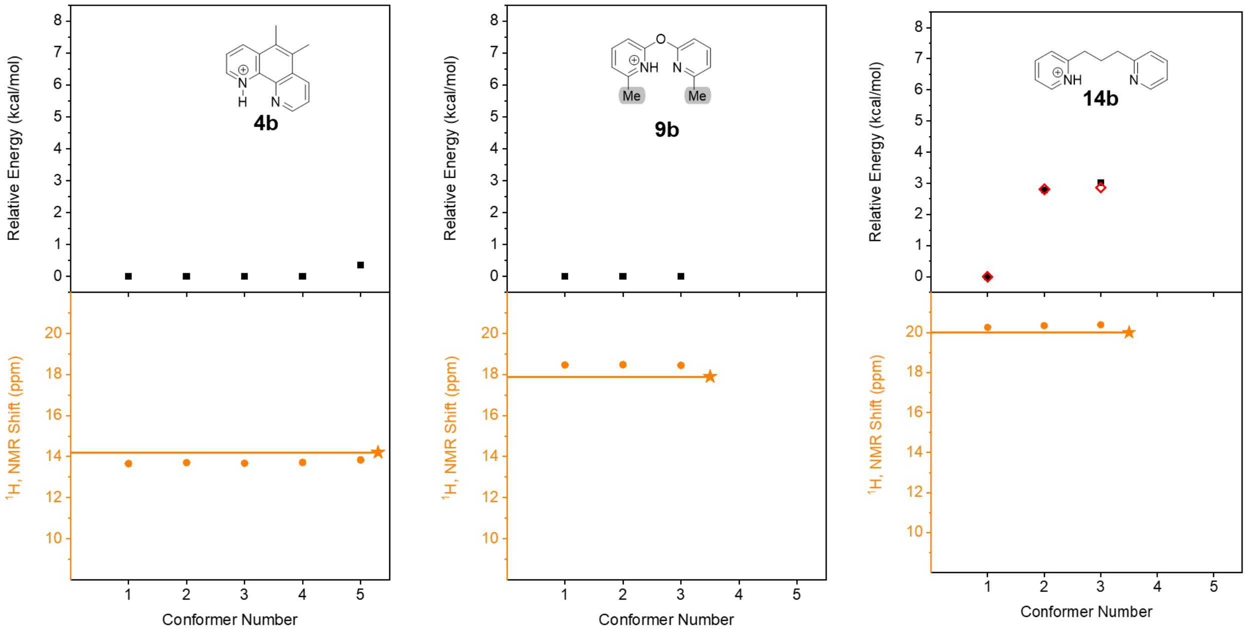

Figure 6.1: Comparison of relative energies and ^{1}H NMR shifts for selected reference compounds: **4b**, **9b**, and **14b**. For **14** relative energies in the gas phase (red diamonds) and solution (black squares) are depicted. Orange stars denote the experimentally observed ^{1}H NMR shift at $-90\,°$C.

Figure 6.1 presents the conformational ensembles of three reference compounds (**4b**, **9b**, **14b**), for which all structures considered-to-be-unique by CREST ultimately correspond to essentially one physically meaningful conformer. In the case of **14b**, the relative energies vary due to changes in the most flexible linker, the -$CH_2CH_2CH_2$- bridge. However, it is clear that even for **14b**, a single dominant non-covalent interaction determines the overall structural geometry.

For all compounds - except **6b**, which is discussed in the main text - the computed ^{1}H NMR shifts are in excellent agreement with the experimentally observed value, as shown in Figure 7 of the main text.

6.3 Conformer space analyses of 7b, 10b, and 13b

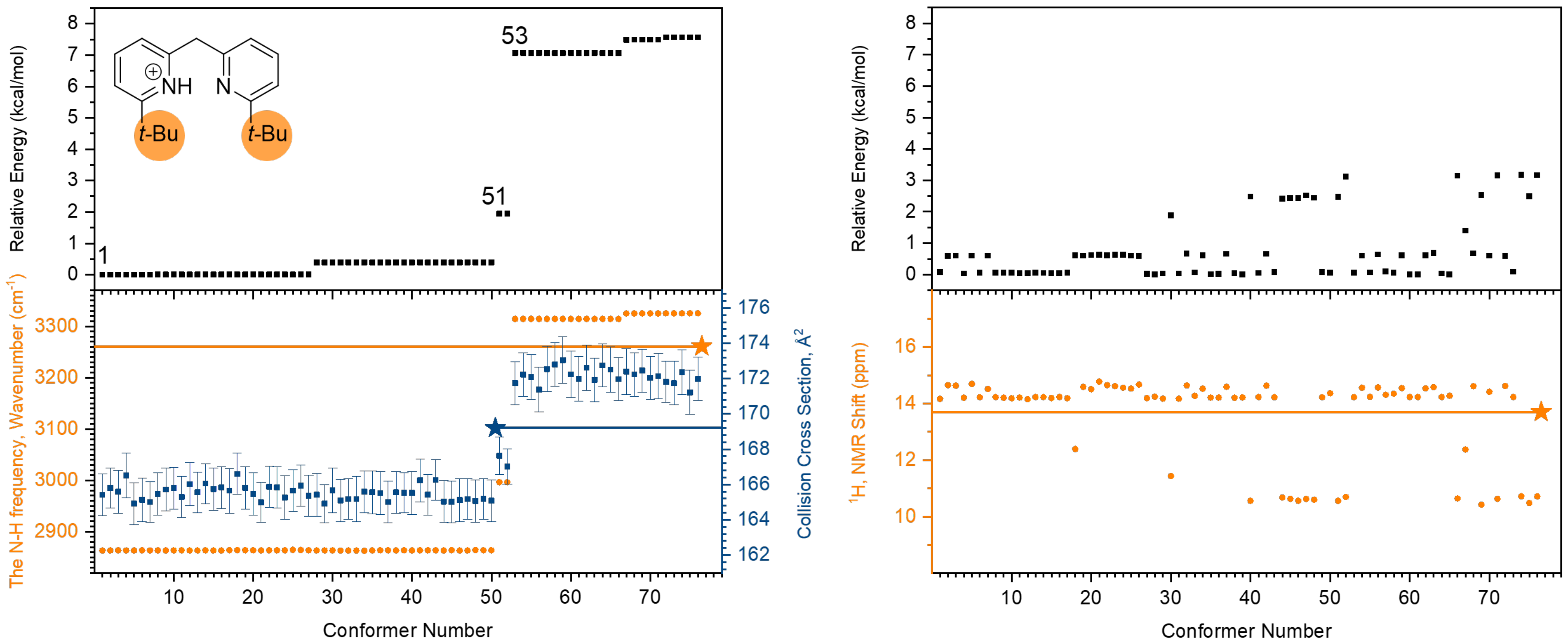

Figure 6.2: Comprehensive analysis of the conformer space of **7b** obtained in the gas phase (PBE-D3(BJ)/def2-TZVP) and reported previously [18] (left), and conformer space computed using the PBE-D4 functional and the def2-TZVPD basis set with SMD(DCM) solvation model (right). The numbering in the right panel corresponds to the conformer order shown on the left. Inclusion of SMD(DCM) substantially reduces the range of accessible conformers in the computed ensemble.

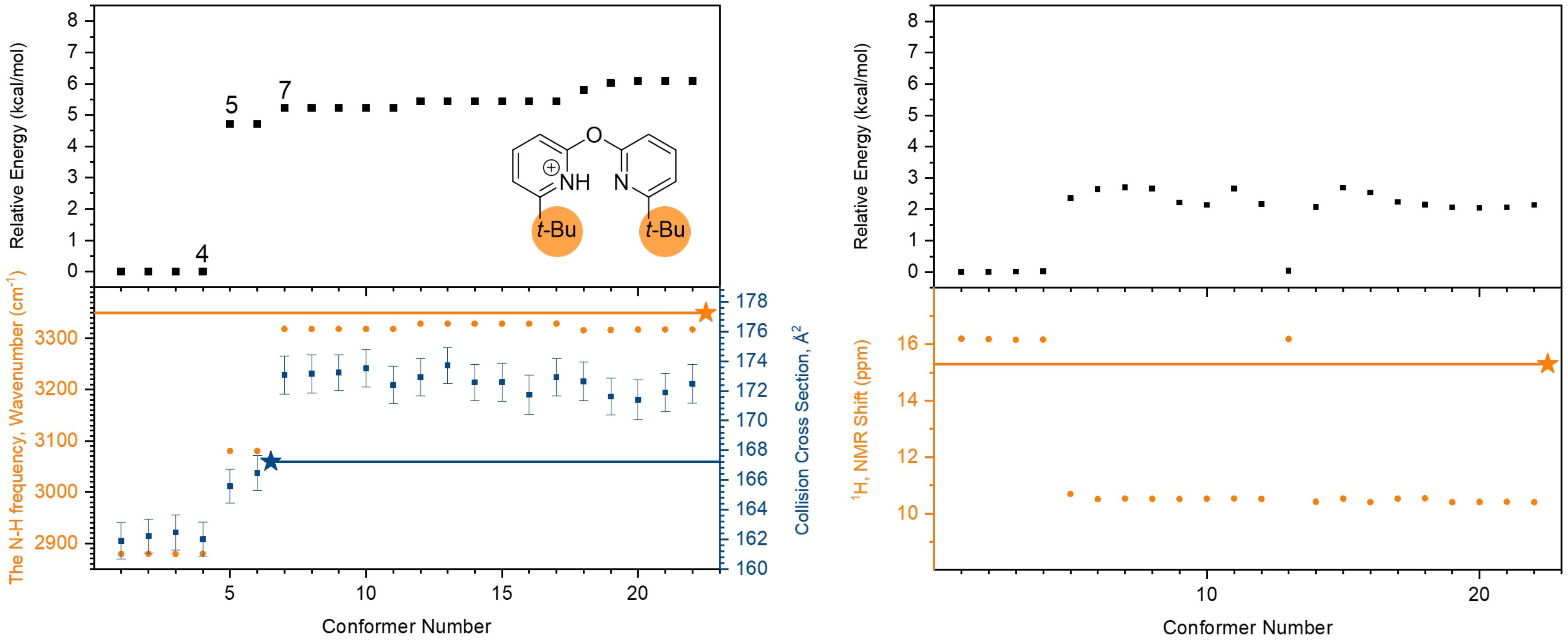

Figure 6.3: Comprehensive analysis of the conformer space of **10b** obtained in the gas phase (PBE-D3(BJ)/def2-TZVP) and reported previously [18] (left), and conformer space computed using the PBE-D4 functional and the def2-TZVPD basis set with SMD(DCM) solvation model (right). The numbering in the right panel corresponds to the conformer order shown on the left. Inclusion of SMD(DCM) substantially reduces the range of accessible conformers in the computed ensemble.

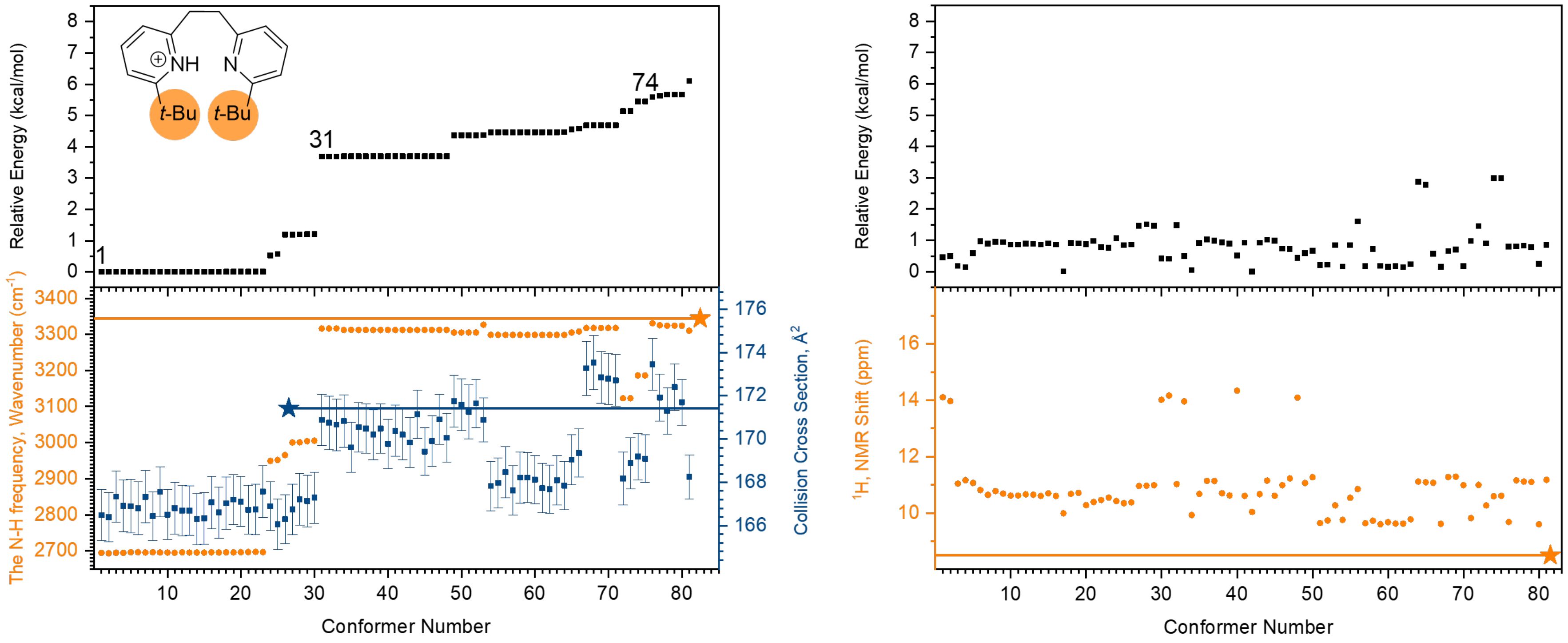

Figure 6.4: Comprehensive analysis of the conformer space of **13b** obtained in the gas phase (PBE-D3(BJ)/def2-TZVP) and reported previously [18] (left), and conformer space computed using the PBE-D4 functional and the def2-TZVPD basis set with SMD(DCM) solvation model (right). The numbering in the right panel corresponds to the conformer order shown on the left. Inclusion of SMD(DCM) substantially reduces the range of accessible conformers in the computed ensemble.

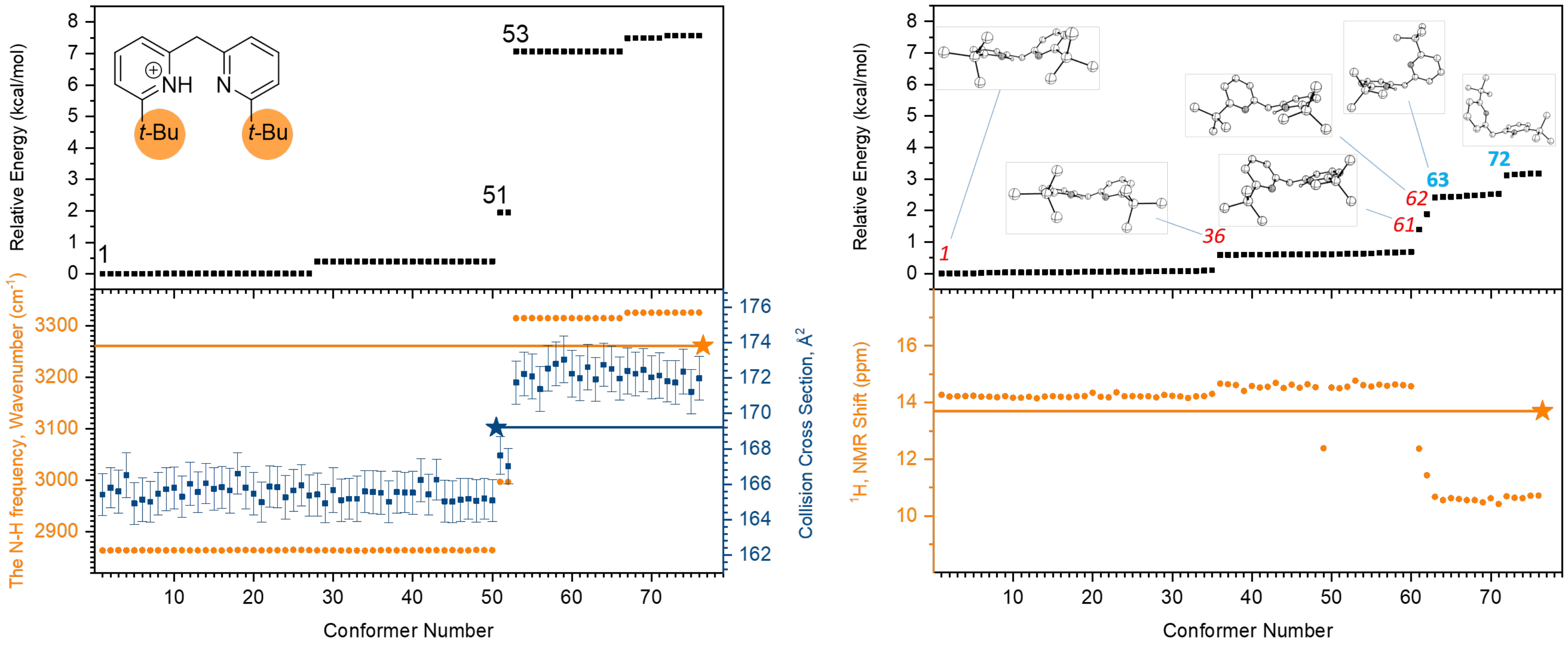

Figure 6.5: Comprehensive analysis of the conformer space of **7b** obtained in the gas phase (PBE-D3(BJ)/def2-TZVP) and reported previously [18] (left), and conformer space computed using the PBE-D4 functional and the def2-TZVPD basis set with SMD(DCM) solvation model (right). The numbering in both panels corresponds to decreasing relative stability. Inclusion of SMD(DCM) substantially reduces the range of accessible conformers in the computed ensemble. Selected conformers are shown in the right panel.

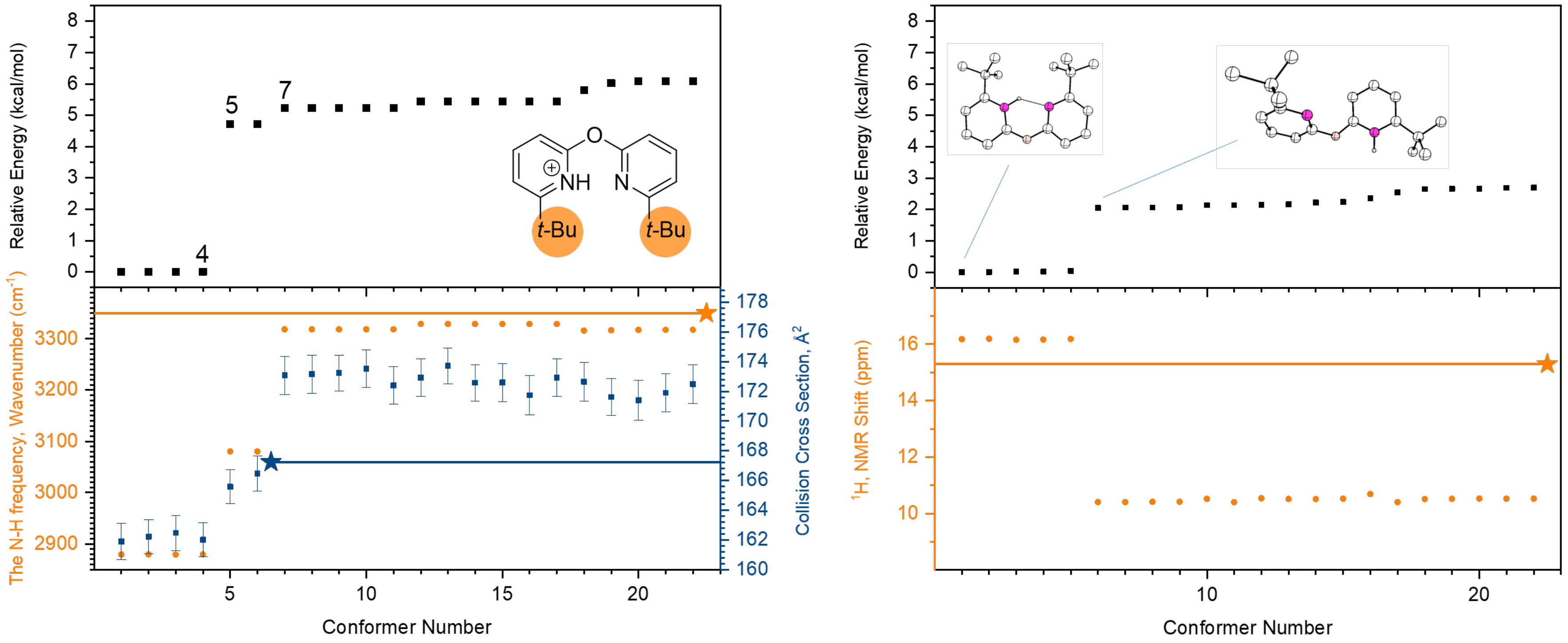

Figure 6.6: Comprehensive analysis of the conformer space of **10b** obtained in the gas phase (PBE-D3(BJ)/def2-TZVP) and reported previously [18] (left), and conformer space computed using the PBE-D4 functional and the def2-TZVPD basis set with SMD(DCM) solvation model (right). The numbering in both panels corresponds to decreasing relative stability. Inclusion of SMD(DCM) substantially reduces the range of accessible conformers in the computed ensemble. Selected conformers are shown in the right panel.

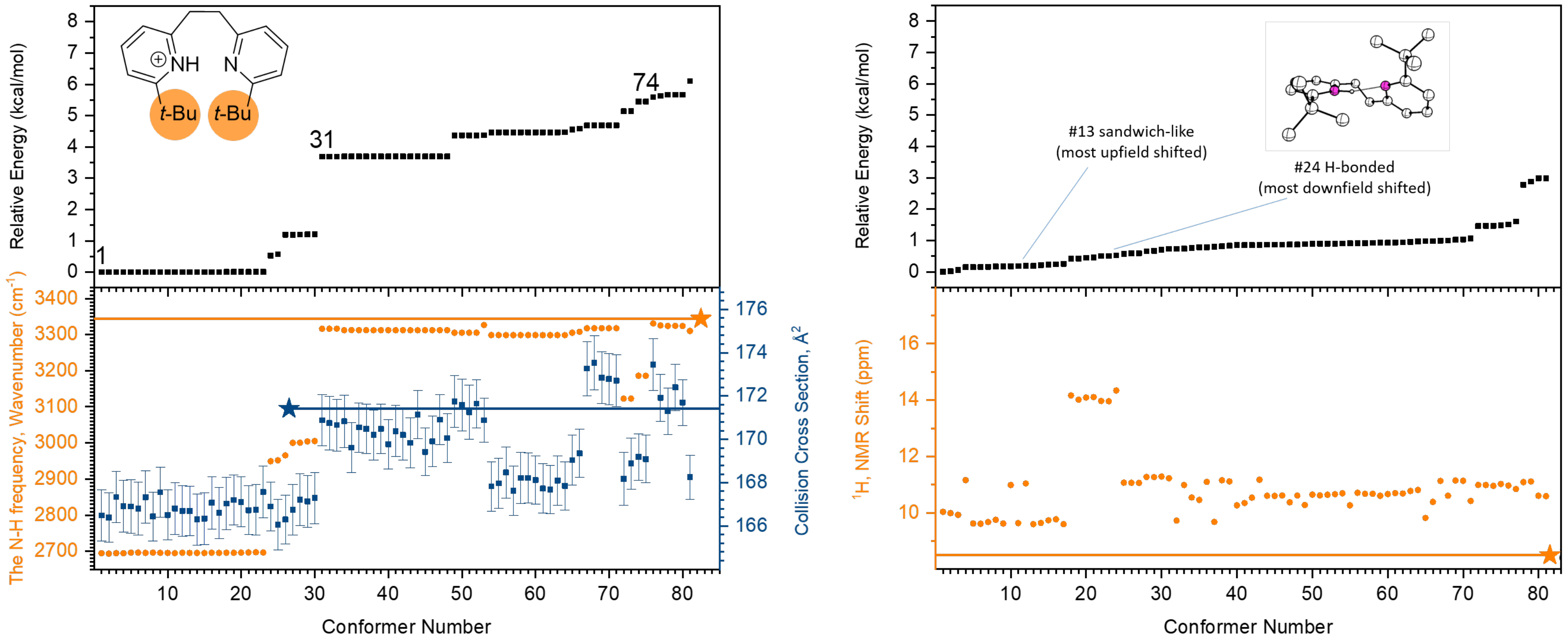

Figure 6.7: Comprehensive analysis of the conformer space of **13b** obtained in the gas phase (PBE-D3(BJ)/def2-TZVP) and reported previously [18] (left), and conformer space computed using the PBE-D4 functional and the def2-TZVPD basis set with SMD(DCM) solvation model (right). The numbering in both panels corresponds to decreasing relative stability. Inclusion of SMD(DCM) substantially reduces the range of accessible conformers in the computed ensemble. Selected conformer is shown in the right panel

Manual and Unsupervised Clustering

7.1 General notes

As all accessible conformers lie within 3 kcal/mol of the global minimum, even the highest-energy conformer may contribute meaningfully to ensemble-averaged properties (with a Boltzmann factor of approximately 0.6% at room temperature).

Furthermore, Figures 6.1–6.7 suggest that several conformers likely represent the same physical structure, as indicated by their similar ^{1}H chemical shifts and relative energies (as well as RMSD values compared to XRD geometries; not shown).

This raises a key question: how can one meaningfully determine the number of unique conformers to use for computing Boltzmann-weighted ^{1}H NMR shifts?

To avoid subjective bias, a custom Python script was developed to apply the hierarchical density-based spatial clustering of applications with noise (HDBSCAN) algorithm (Section 7.2). A comparative analysis of ensemble-averaged NMR shifts, based on both manual clustering and HDBSCAN results, is presented and discussed in Section 7.3.

7.2 Python-Based Protocol for HDBSCAN

```python
import pandas as pd
import hdbscan
from sklearn.preprocessing import StandardScaler

# === Step 1: Load CSV ===
df = pd.read_csv("input_file.csv", header=None)
df.columns = ['Conformer', 'Relative_Stability', 'H1_NMR_Shift', 'RMSD']

# === Step 2: Select features and standardize ===
features = df[['Relative_Stability','H1_NMR_Shift', 'RMSD']]
scaler = StandardScaler()
X_scaled = scaler.fit_transform(features)

# === Step 3: Run HDBSCAN clustering ===
clusterer = hdbscan.HDBSCAN(min_cluster_size=5)
df['Cluster'] = clusterer.fit_predict(X_scaled)

# === Step 4: Show results ===
print(df[['Conformer', 'Cluster']])
print("\nNumber of clusters found:", len(set(df['Cluster'])) - (1 if -1 in df['Cluster'].values else 0))

# === Optional: Save results ===
df.to_csv("7b_clustered.csv", index=False)

# === Optional: Save summary and cluster assignments to a file ===
with open("cluster_summary.txt", "w") as f:
    f.write(df[['Conformer', 'Cluster']].to_string(index=False))
    f.write("\n\nNumber of clusters found: {}\n".format(
        len(set(df['Cluster'])) - (1 if -1 in df['Cluster'].values else 0)
    ))
    f.write("Noise points: {}\n".format((df['Cluster'] == -1).sum()))
```

7.3 Influence of Parameter Selection on HDBSCAN Clustering

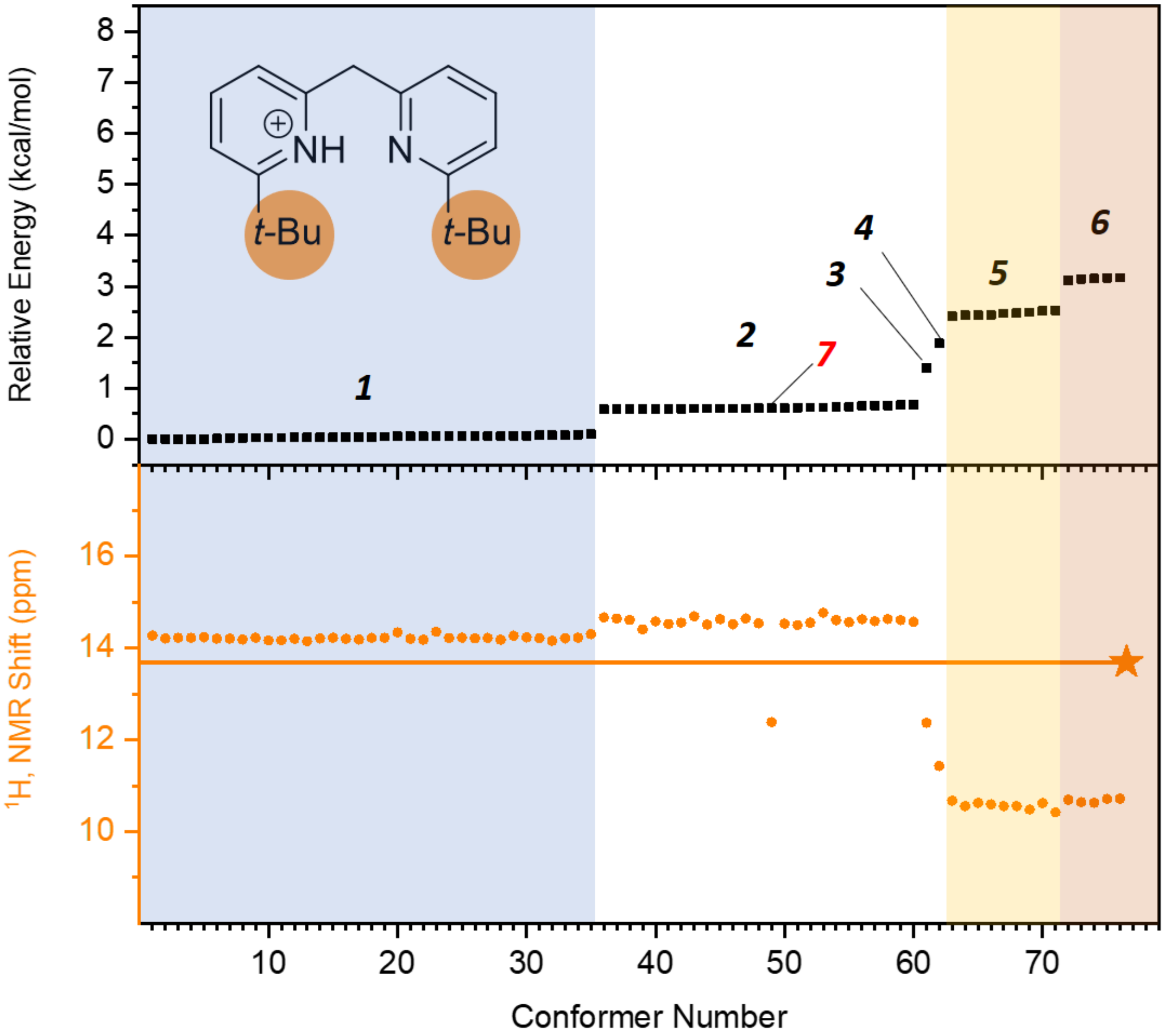

Figure 7.1: From the manual clustering analysis of **7b**, several groups of conformers were identified. Only one conformer, belonging to family #7, may be considered an outlier. Its inclusion or exclusion from the Boltzmann averaging has no significant effect on the ensemble-averaged outcome.

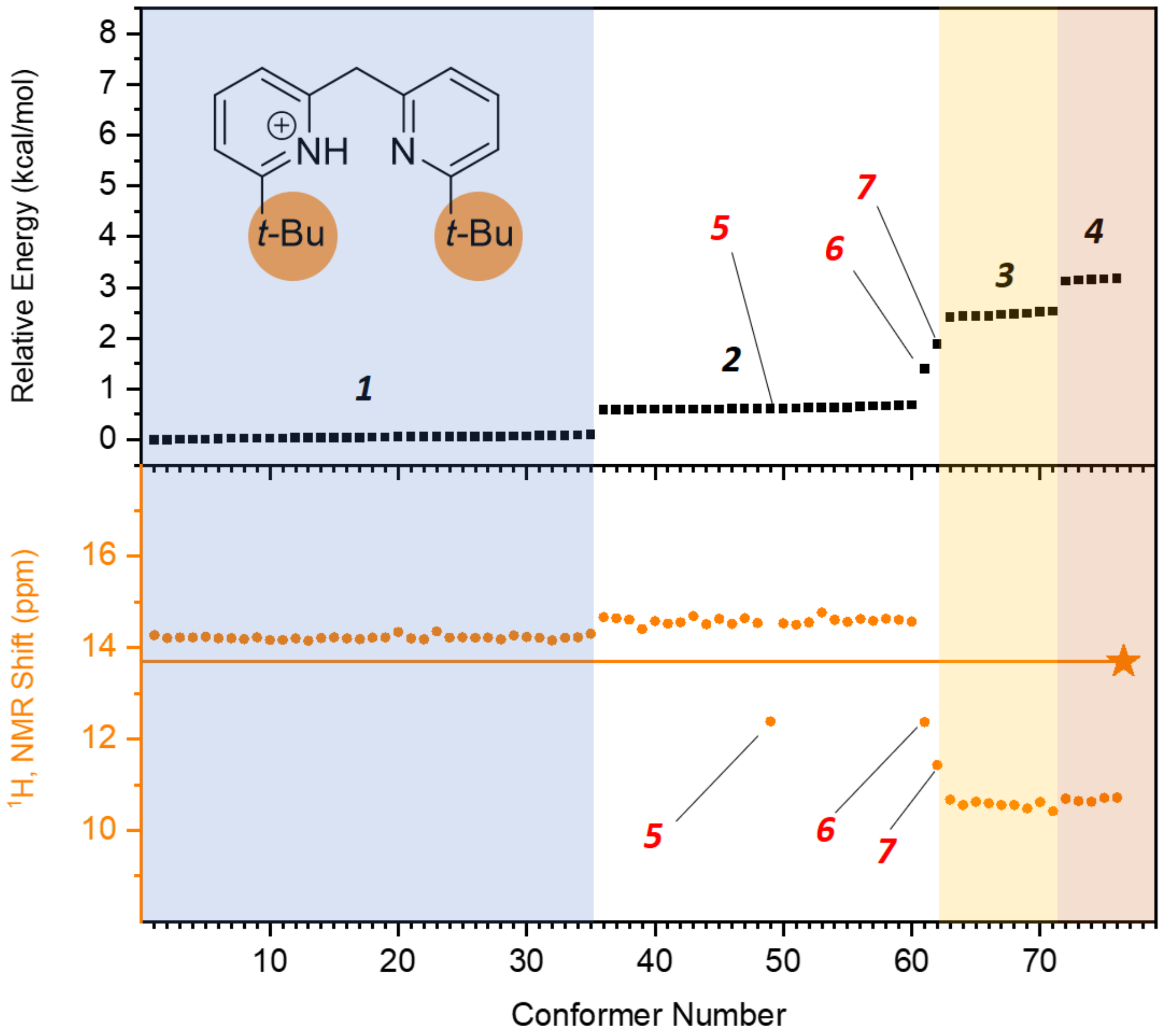

Figure 7.2: HDBSCAN clustering analysis of **7b** yields results identical to those obtained from the manual analysis shown above (Figure 7.1). Parameters used for HDBSCAN: minimum cluster size = 2. Features used for clustering: ^{1}H NMR shift and relative energy.

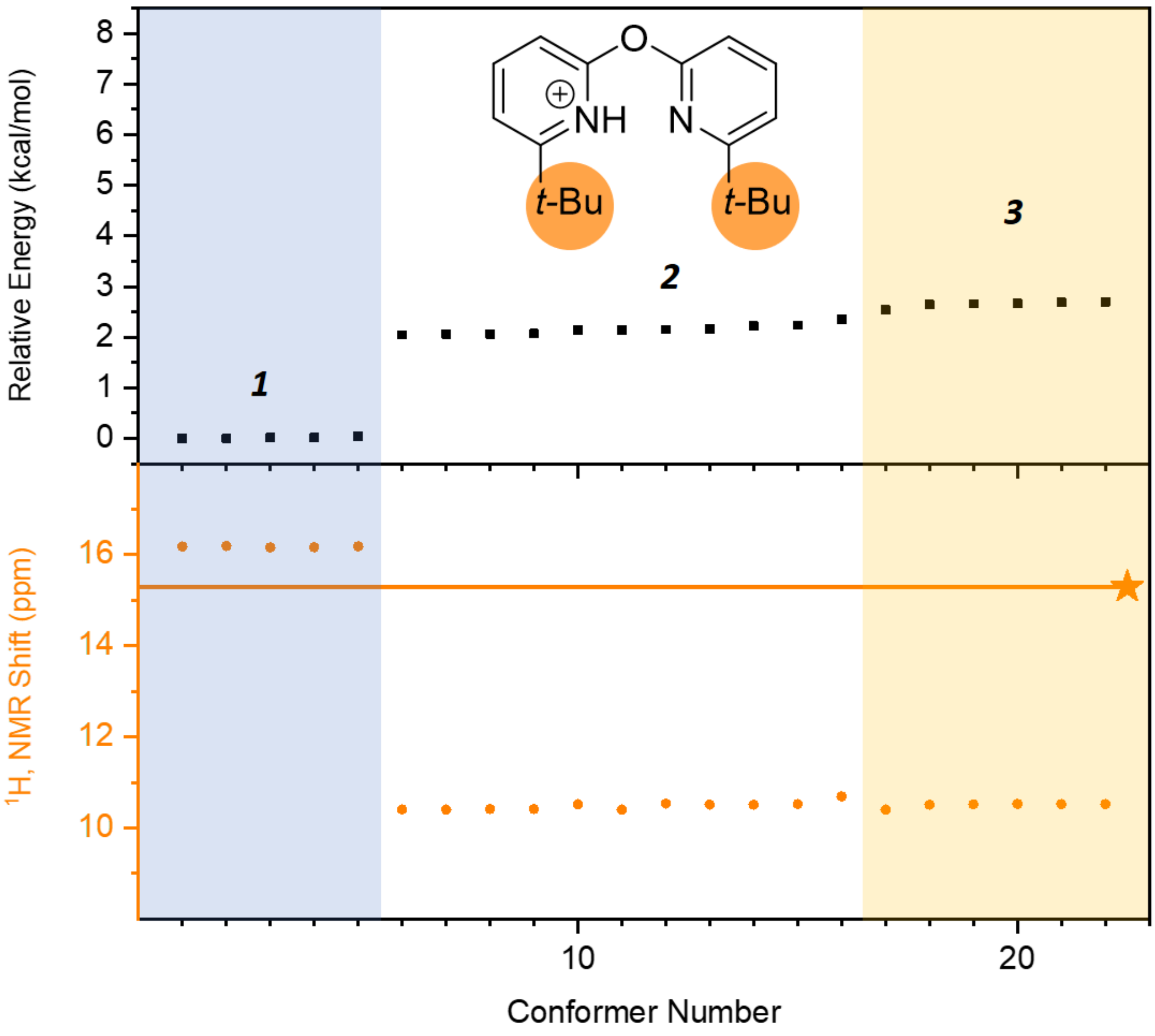

Figure 7.3: Both the manual clustering and HDBSCAN analyses of **10b** yield identical results. Parameters used for HDBSCAN: minimum cluster size = 5. Features used for clustering: ^{1}H NMR shift and relative energy.

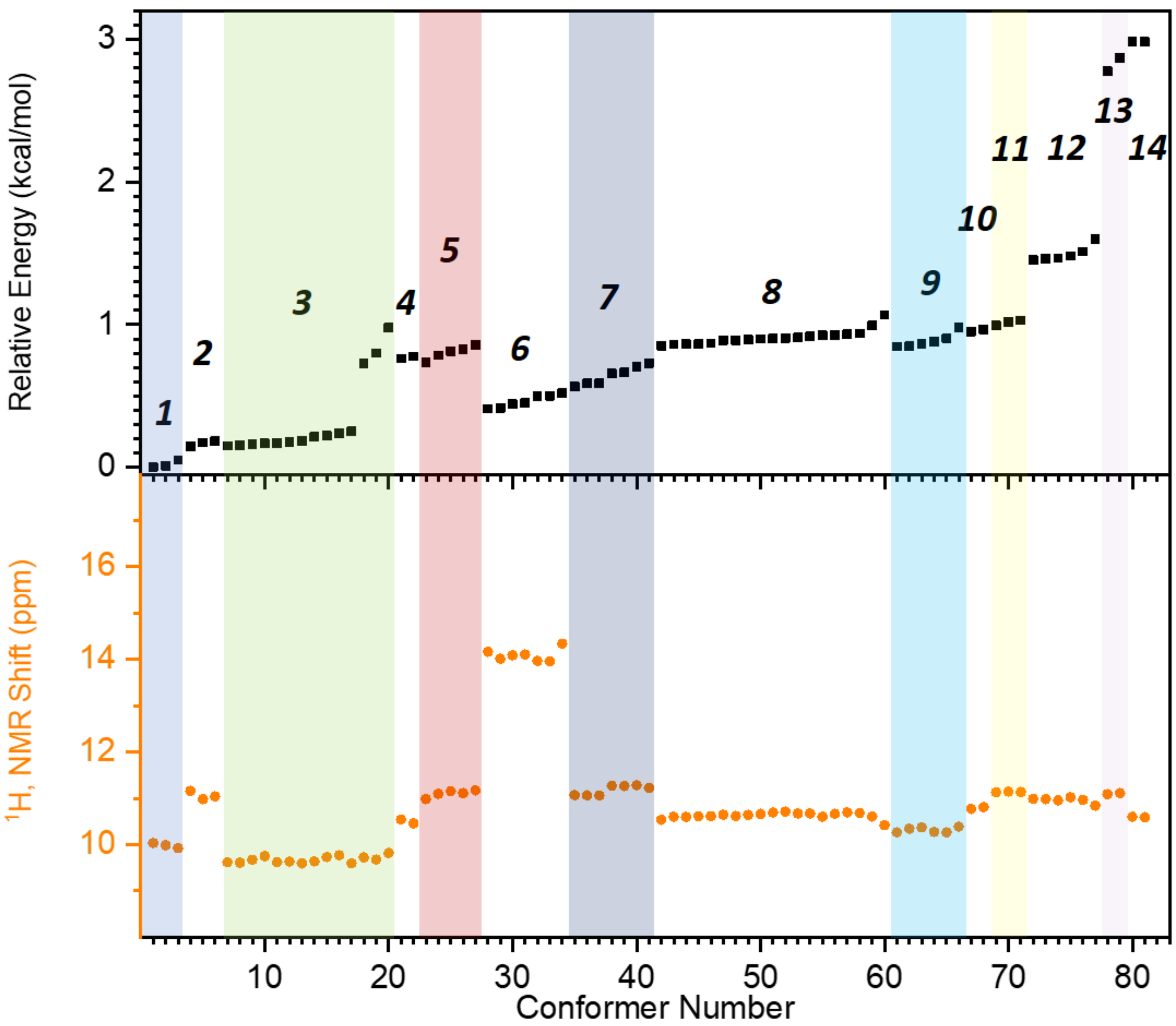

Figure 7.4: Manual clustering analysis of **13b** identified several distinct conformer families. Notes: (1) conformer numbering was adjusted to improve the visual grouping of each family; (2) Note that the vertical axis range here (0–3 kcal/mol) is significantly narrower than in other plots in this section (0–8 kcal/mol); (3) The computational analysis of **13b** offers limited insight, as none of the computed structures reproduce the experimentally observed upfield ^{1}H NMR shift (from 8.5 ppm at $-90\,^{\circ}$C to 3.6 ppm at $+10\,^{\circ}$C). Therefore, the choice of clustering scheme or conformer grouping has little impact on the final outcome.

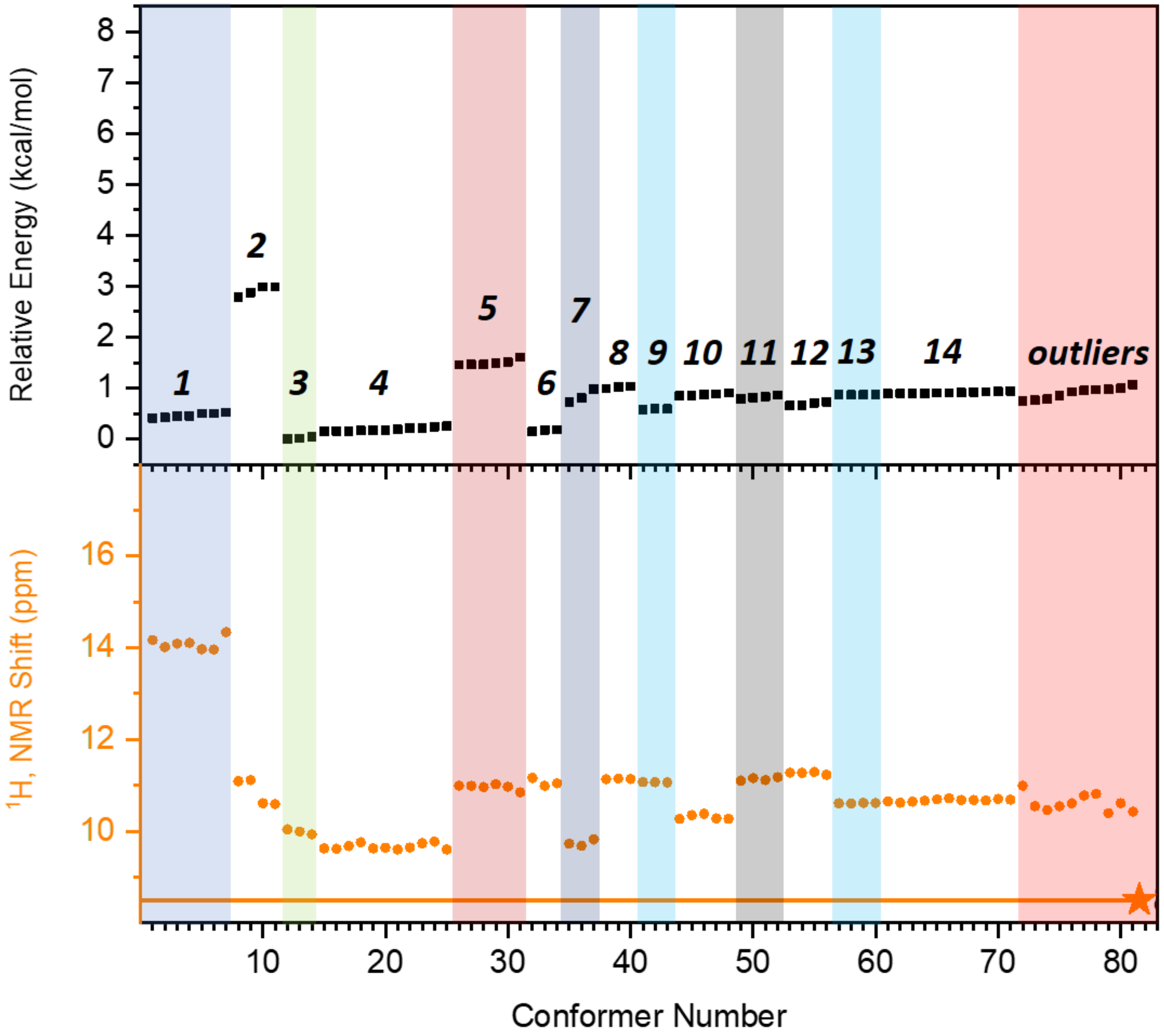

Figure 7.5: HDBSCAN clustering analysis of **13b** identified several distinct conformer families. Notes: (1) conformer numbering was adjusted to improve the visual grouping of each family; (2) the performed computational analysis of **13b** offers limited insight, as none of the computed structures reproduce the experimentally observed upfield ^{1}H NMR shift (from 8.5 ppm at $-90\,^\circ$C to 3.6 ppm at $+10\,^\circ$C). Therefore, the choice of clustering scheme or conformer grouping has little impact on the final outcome.

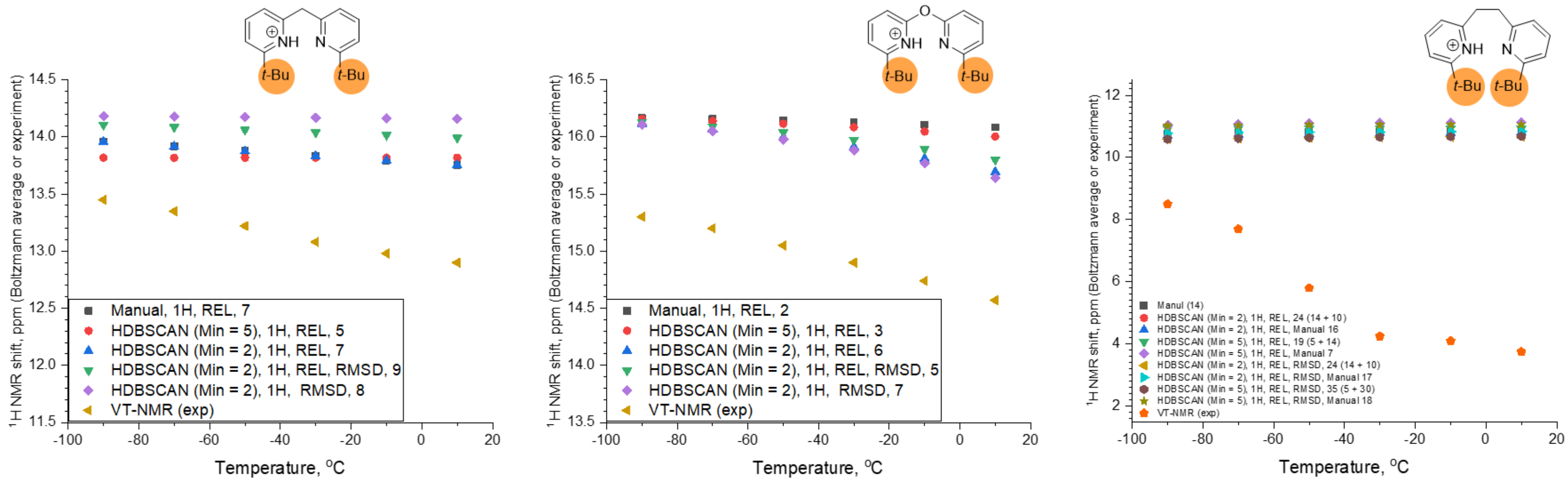

Figure 7.6: Temperature-dependent Boltzmann-averaged ^{1}H NMR chemical shifts for the conformer ensembles of **7b** (left), **10b** (center), and **13b** (right), compared to experimental values (orange triangles). Different clustering schemes—either manual or HDBSCAN—were applied to avoid overcounting structurally redundant conformers. For HDBSCAN, various clustering parameters were tested (for representative examples see Figures 7.1-7.5). In contrast to the near-quantitative agreement observed for the "simple" bis-pyridines in Figure 7 (main text), larger deviations are observed for all bis-pyridines containing *tert*-butyl groups. Note that the vertical axis range for **13b** is substantially larger than that for **7b** or **10b**.

References

(1) Tsybizova, A.; Fritsche, L.; Gorbachev, V.; Miloglyadova, L.; Chen, P. *The Journal of chemical physics* **2019**, *151.*

(2) Gorbachev, V.; Tsybizova, A.; Miloglyadova, L.; Chen, P. *Journal of the American Chemical Society* **2022**, *144*, 9007–9022.

(3) Zheng, Z.; Elmkaddem, M. K.; Fischmeister, C.; Roisnel, T.; Thomas, C. M.; Carpentier, J.-F.; Renaud, J.-L. *New J. Chem.* **2008**, *32*, Publisher: The Royal Society of Chemistry, 2150–2158.

(4) Stumpf, T.-D. J.; Steinbach, M.; Höltke, M.; Heuger, G.; Grasemann, F.; Fröhlich, R.; Schindler, S.; Göttlich, R. *European Journal of Organic Chemistry* **2018**, *2018*, 5538–5547.

(5) McKeown, B. A.; Gonzalez, H. E.; Michaelos, T.; Gunnoe, T. B.; Cundari, T. R.; Crabtree, R. H.; Sabat, M. *Organometallics* **2013**, *32*, Publisher: American Chemical Society, 3903–3913.

(6) Tlili, A.; Monnier, F.; Taillefer, M. *Chemistry – A European Journal* **2010**, *16*, 12299–12302.

(7) Quagliotto, P.; Viscardi, G.; Barolo, C.; Barni, E.; Bellinvia, S.; Fisicaro, E.; Compari, C. *The Journal of Organic Chemistry* **2003**, *68*, PMID: 14510538, 7651–7660.

(8) Palav, A.; Misal, B.; Ernolla, A.; Parab, V.; Waske, P.; Khandekar, D.; Chaudhary, V.; Chaturbhuj, G. *Organic Process Research & Development* **2019**, *23*, 244–251.

(9) Popov, I.; Do, H.-Q.; Daugulis, O. *The Journal of Organic Chemistry* **2009**, *74*, PMID: 19827765, 8309–8313.

(10) Dolomanov, O. V.; Bourhis, L. J.; Gildea, R. J.; Howard, J. A. K.; Puschmann, H. *Journal of Applied Crystallography* **2009**, *42*, 339–341.

(11) Sheldrick, G. M. *Acta Crystallographica Section A* **2015**, *71*, 3–8.

(12) Sheldrick, G. M. *Acta Crystallographica Section C* **2015**, *71*, 3–8.

(13) Caldeweyher, E.; Bannwarth, C.; Grimme, S. *J. Chem. Phys.* **2017**, *147*, 034112.

(14) Caldeweyher, E.; Ehlert, S.; Hansen, A.; Neugebauer, H.; Spicher, S.; Bannwarth, C.; Grimme, S. *J. Chem. Phys.* **2019**, *150*, 154122.

(15) Caldeweyher, E.; Mewes, J.; Ehlert, S.; Grimme, S. *Phys. Chem. Chem. Phys.* **2020**, *22*, 8499–8512.

(16) Wittmann, L.; Gordiy, I.; Friede, M.; Helmich-Paris, B.; Grimme, S.; Hansen, A.; Bursch, M. *Phys. Chem. Chem. Phys.* **2024**, *26*, 21379–21394.

(17) Neese, F. *WIRES Comput. Molec. Sci.* **2022**, *12*, e1606.

(18) Gorbachev, V.; Savoy, A.; Tsybizova, A.; Pollice, R.; van Tetering, L.; Martens, J.; Oomens, J.; Berden, G.; Chen, P. *Journal of the American Chemical Society* **2025**.

(19) Garcia-Rates, M.; Neese, F. *J. Comput. Chem.* **2020**, *41*, 922–939.

(20) Neese, F. *WIRES Comput. Molec. Sci.* **2012**, *2*, 73–78.

(21) Neese, F. *WIRES Comput. Molec. Sci.* **2018**, *8*, 1–6.

(22) Neese, F.; Wennmohs, F.; Becker, U.; Riplinger, C. *J. Chem. Phys.* **2020**, *152*, Art. No. L224108.

(23) Neese, F. *J. Comp. Chem.* **2003**, *24*, 1740–1747.

(24) Neese, F. *Chem. Phys. Lett.* **2000**, *325*, 93–98.

(25) Neese, F. *J. Comp. Chem.* **2022**, 1–16.

(26) Stoychev, G.; Auer, A.; Izsak, R.; Neese, F. *J. Chem. Theory Comput.* **2018**, *14*, 619–637.
